# Supplementary material for: Data-Independent Acquisition (DIA)-Based Proteomics for the Identification of Biomarkers in Tissue Washings of Endometrial Cancer
Source: Int J Mol Sci. 2025 Nov 27;26(23):11498. doi: 10.3390/ijms262311498 (PMC12691889; doi:10.3390/ijms262311498)

Figure S1. Whole-membrane Ponceau red staining for all samples included in the study. The figure shows the full membranes corresponding to the CTRL, low-grade, and high-grade conditions for each analyzed protein (APRC2 and SYDE2). Ponceau red staining was used to assess equal protein loading and uniform transfer across all samples and experimental groups.

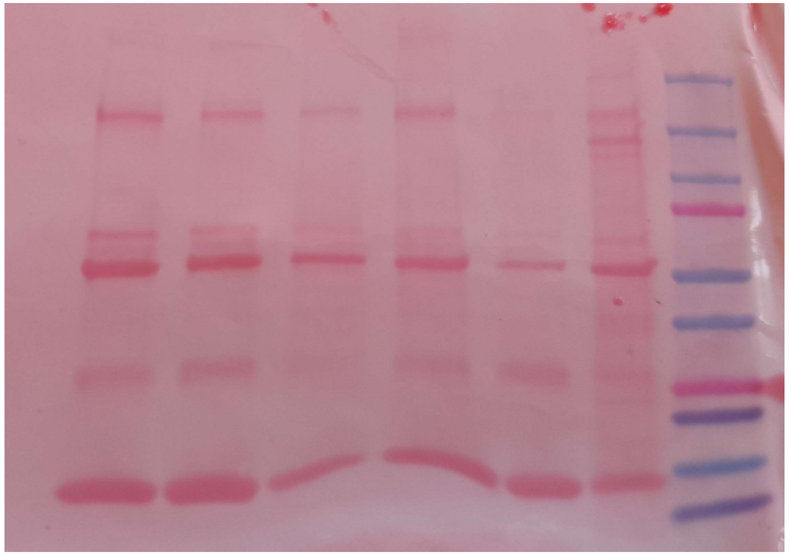

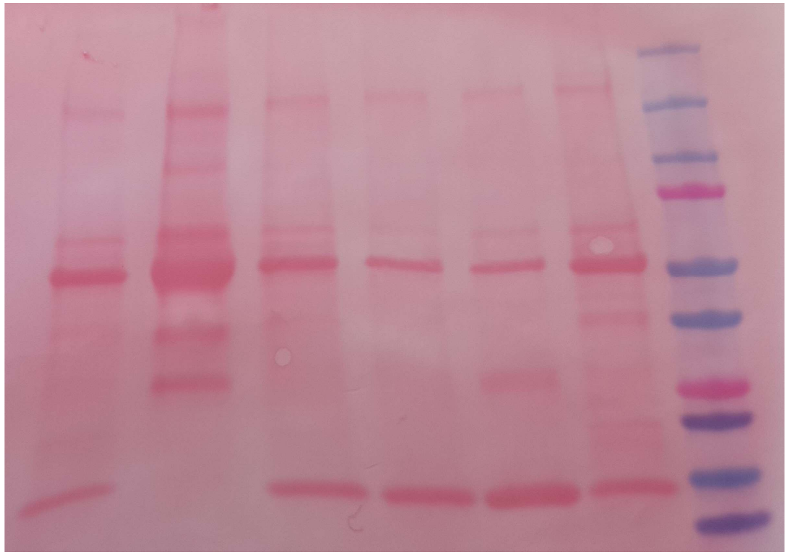

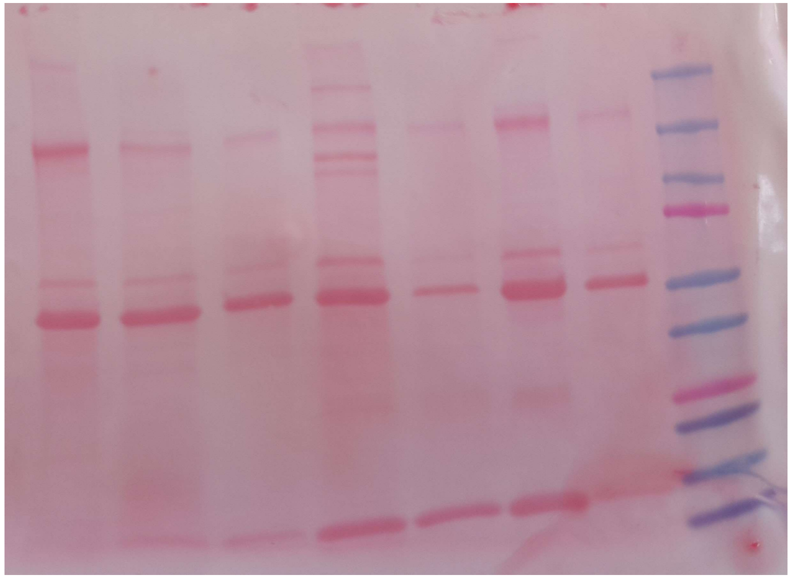

CTRL whole membrane stained with red ponceau

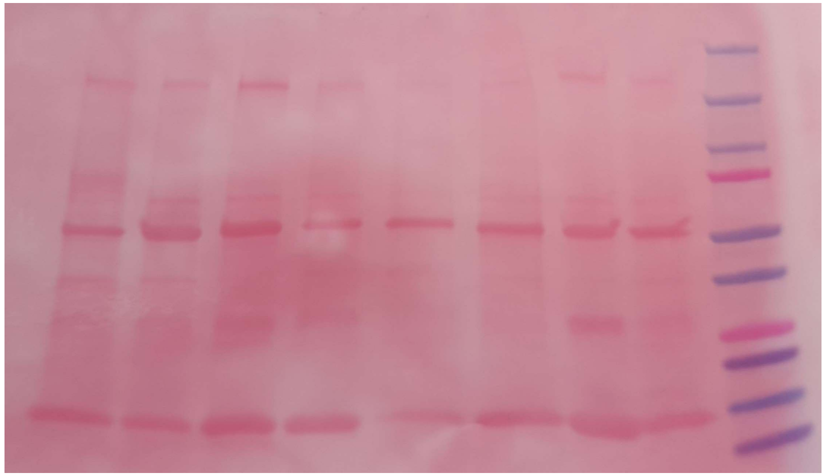

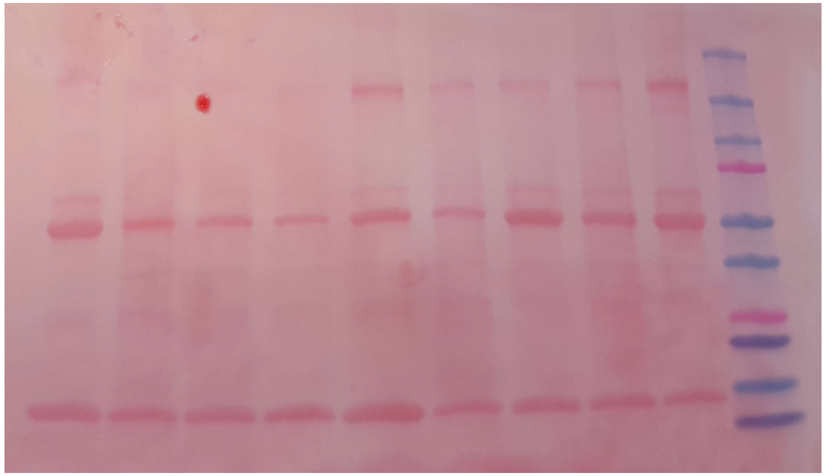

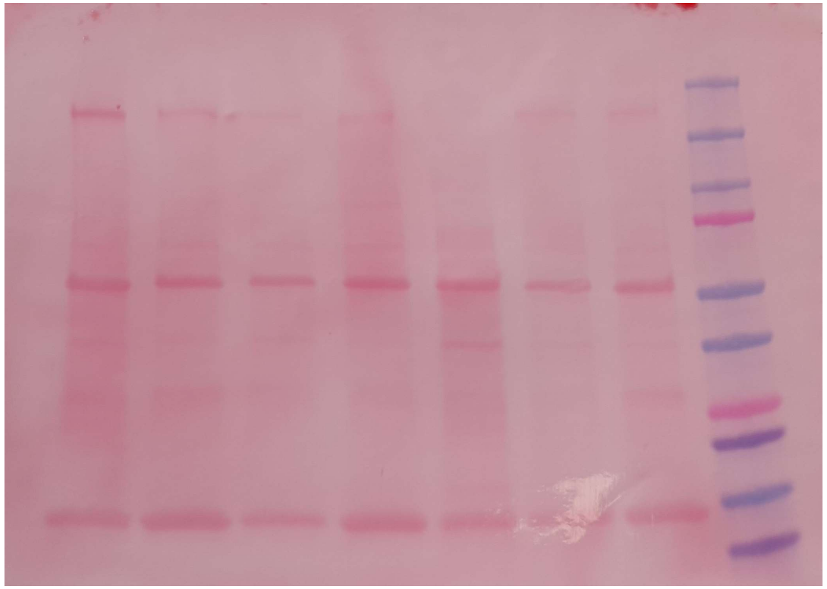

ADK low grade whole membrane stained with red ponceau

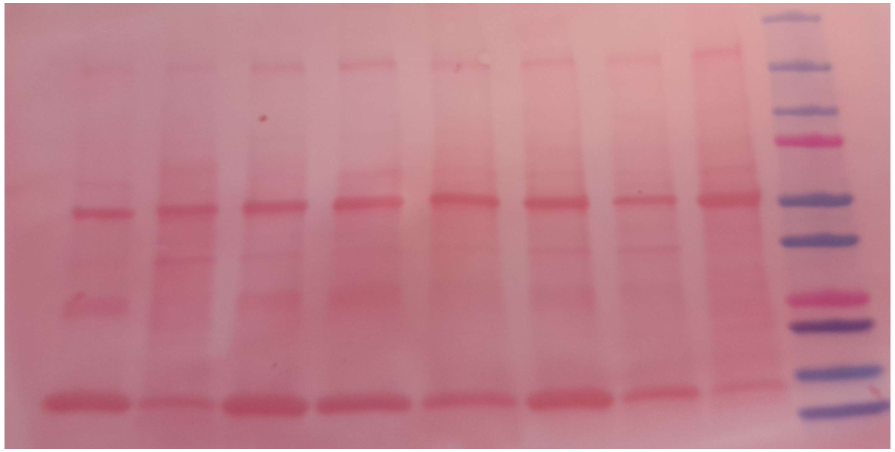

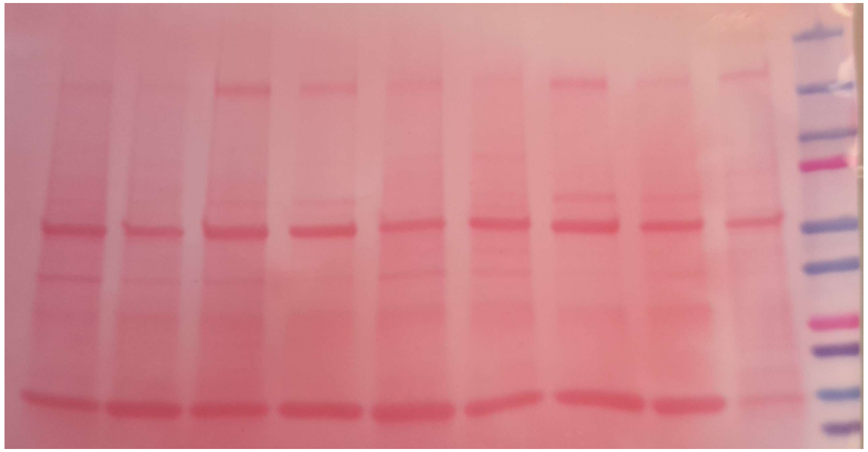

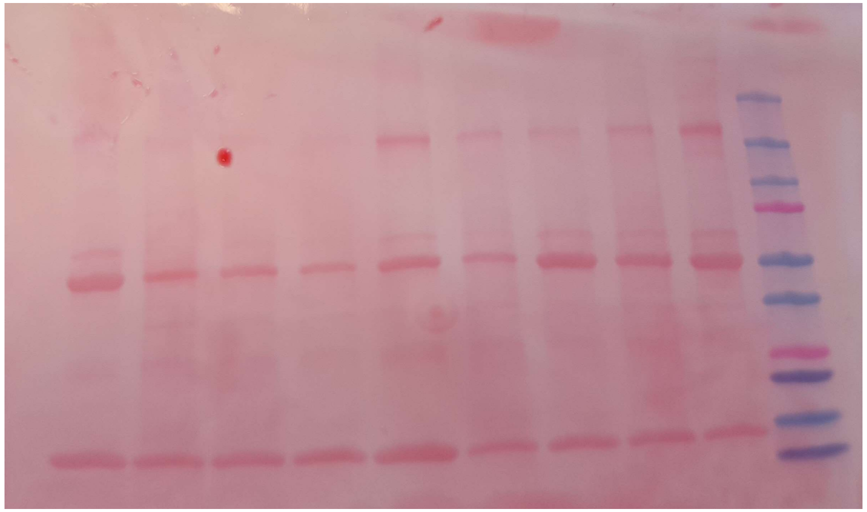

ADK high grade whole membrane stained with red ponceau

CTRL sample of the whole membrane of protein APRC2

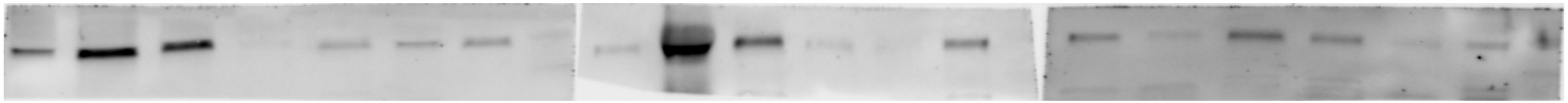

EC low grade sample of the whole membrane of protein APRC2

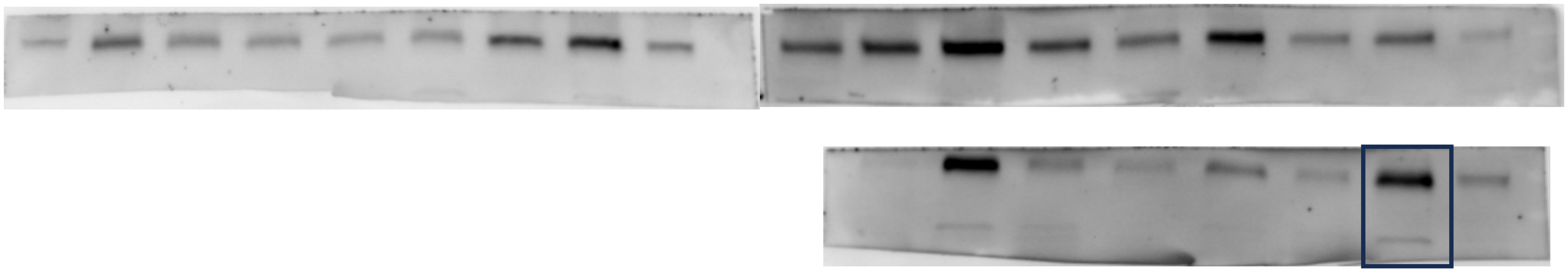

EC high grade sample of the whole membrane of protein APRC2

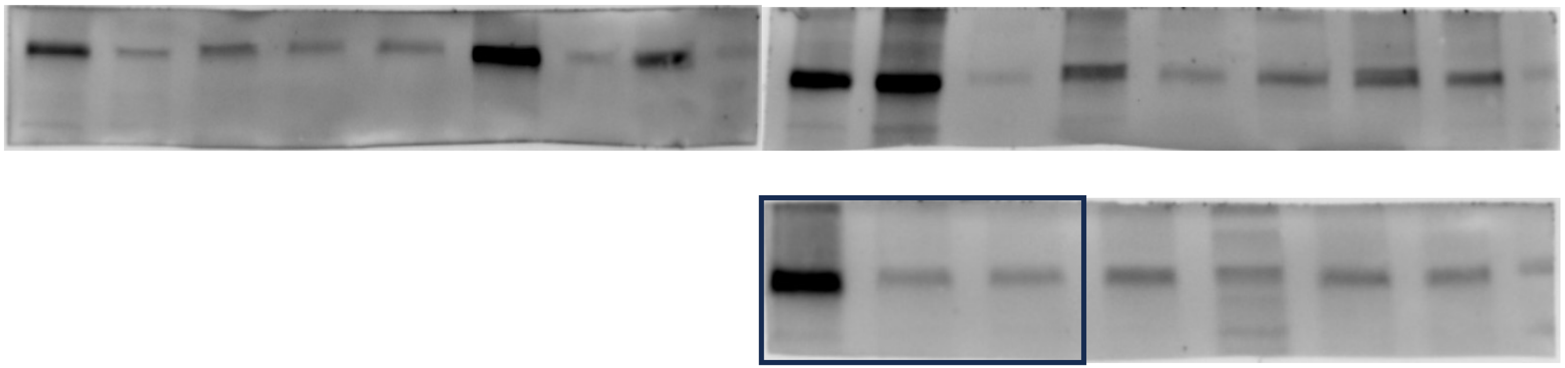

CTRL sample of the whole membrane of protein SYDE2

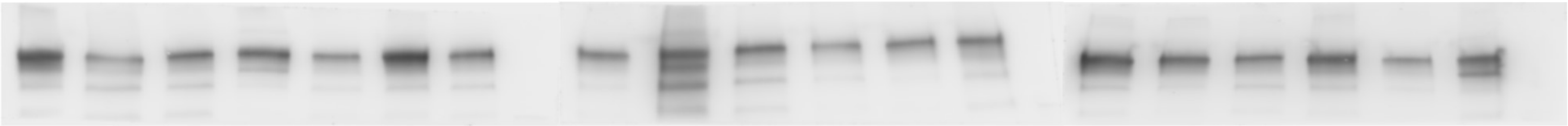

EC low grade sample of the whole membrane of protein SYDE2

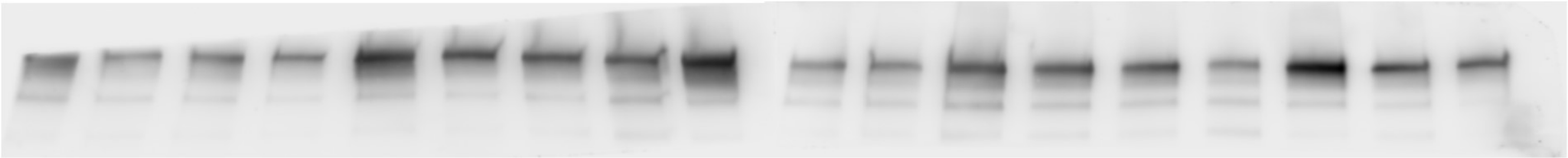

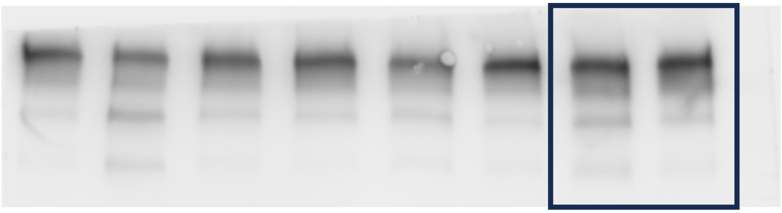

EC high grade sample of the whole membrane of protein SYDE2

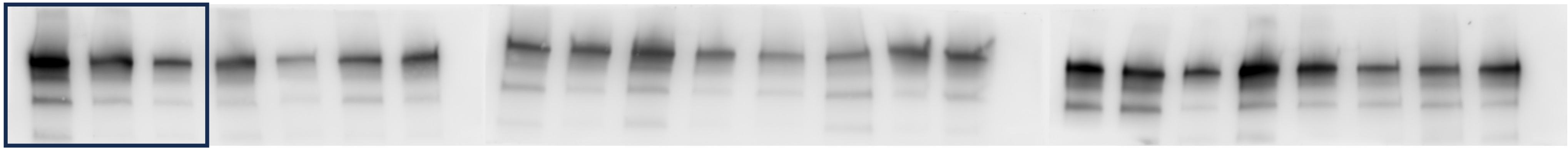

Supplement: Supplementary file 1 [file ijms-26-11498-s001.zip › Figure S1.pdf]
