# Supplementary material for: Data-Independent Acquisition (DIA)-Based Proteomics for the Identification of Biomarkers in Tissue Washings of Endometrial Cancer
Source: Int J Mol Sci. 2025 Nov 27;26(23):11498. doi: 10.3390/ijms262311498 (PMC12691889; doi:10.3390/ijms262311498)
Supplement: Supplementary file 1 [file ijms-26-11498-s001.zip › Supplemental file S2.pdf]

Descriptive statistics of all proteins identified with at least 60 % of values in the High-grade EC group (N=24)

PG.Genes	n	mean	sd	median	q1	q3	min	max
PPIAL4E;PPIAL4D;PPIAL4F	17	7797	3579	6456	5453	9991	3081	15995
TRAV17	18	7305	4486	6060	4518	8930	1801	17684
DPEP2NB	23	10226	13250	5520	4015	9748	2389	64382
CENPVL3	24	20214	5562	19274	17235	23995	9990	32484
TEX48	24	10607	6150	9755	7055	12472	1326	30580
CPHXL	24	6387	4165	5614	3292	9033	1416	19287
ERFL	24	13723	7831	11990	9253	15303	3837	37304
TRBV4-1	24	22805	30510	12083	8321	22590	4308	136262
RBM47	24	6481	6457	4432	3146	6794	1966	33452
UBA6	24	7877	3754	7046	5476	10622	2978	17298
ESYT3	24	29651	7134	28468	25579	32340	17058	44382
UHRF1BP1L	24	123511	46057	106236	101091	131436	69227	288735
SHTN1	24	20996	9010	19657	12508	28106	6901	36028
TLDC2	23	11579	6651	11313	6062	15764	2097	26267
BICDL2	24	32721	14771	27056	22256	38336	17600	70678
ARHGEF37	16	2318	1402	1724	1407	2989	787	6026
MEX3A	24	259176	164547	198360	158214	312036	79165	748248
ELOVL7	23	217098	103210	211246	153159	260004	48852	467250
SSC5D	24	262004	65403	239735	227153	274582	173185	455570
SH3PXD2B	24	17917	6567	16665	12302	21424	9061	33973
HYKK	24	5315	3469	4414	3455	5842	471	16219
CRPPA	24	20132	5693	19261	15484	24331	11894	33955
MEGF11	24	2615	1003	2470	1793	2980	1377	5026
FAM172BP	24	37083	13839	33266	27305	43951	18257	66464
VWA3A	24	52044	5593	52013	47931	56856	41305	61699

PG.Genes	n	mean	sd	median	q1	q3	min	max
C2orf78	24	44708	24810	37104	24987	55467	16128	108796
ANKRD33B	24	9404	3930	8956	7560	10368	3641	24306
IQCA1L	24	19672	4500	19067	17501	21565	11454	33128
PALM3	23	7183	2932	6542	5183	8971	3118	13800
SYNDIG1L	19	76705	56660	58331	46323	87535	20082	261376
PGP	24	130543	53409	117987	89896	162749	44155	226395
TRIM49B	24	19567	8747	18525	14815	21197	7062	47714
C5orf51	24	6913	2168	6673	5313	7215	4210	12392
SOWAHB	24	8066	2886	6903	6139	9864	3671	16028
MROH2A	24	16402	2303	16528	15054	18142	11418	20917
ANTXRL	24	19814	4899	19316	17294	21990	12156	29526
EFCAB10	24	14142	3484	12945	11253	15864	9954	21465
WIPF3	24	16420	5164	17118	13262	19330	7735	26592
PCARE	24	116105	21232	114698	103033	126441	83740	186760
ANKRD61	18	1216	571	1254	800	1570	381	2684
OTOL1	24	45547	19628	40587	33508	48931	16624	94875
FBLL1	24	85845	41556	67980	57485	110186	34275	194887
SMCHD1	24	4514819	733045	4395538	4067685	4798711	3354806	6342594
CASTOR2	24	7761	5721	6260	4814	8320	2171	29451
UNC119B	24	15625	6000	13003	11204	19674	9060	31551
LRRC72	24	53331	13247	53994	44360	60903	28174	89580
SDR42E2	24	15024	8167	14582	8337	17252	4796	38519
CPSF4L	24	5831	1725	5813	4934	6797	3241	11775
ANXA2P2	24	58251	42106	58350	27078	84214	6966	194723
NA	24	200294	138065	160322	120454	233435	40276	609269
FAM90A27P	24	23195	11729	20345	14869	26024	13049	61308

PG.Genes	n	mean	sd	median	q1	q3	min	max
ENO4	24	13047	3615	12466	10520	15072	7309	23547
C4orf47	24	7918	2326	7267	6538	8945	4770	15023
SRRM4	24	73889	23873	69610	63324	80380	35841	132939
A2ML1	24	68428	27967	59593	50760	79248	31330	153088
GABRR3	24	46200	16221	47536	36706	52250	16150	86390
NEURL1B	24	1143937	197921	1163823	1036888	1235659	793784	1550630
PRSS47	24	77751	25169	70594	57564	93603	40342	147135
NA	24	21165	6423	19775	17422	23210	11448	39962
BBIP1	24	7946	4057	6472	5748	8061	4033	19956
SERPINE3	24	14177	9754	10361	8775	17565	4591	49907
FADS2B	24	238788	70139	229211	187636	278814	137651	456659
WASH4P	24	9545	6957	6467	5452	11812	3393	29233
CAPN14	24	22529	7058	20856	17728	25100	14033	41928
NA	24	11883	5104	10436	8918	14689	4183	21547
NUDT19	23	1580	688	1406	1111	1859	637	3374
DCDC2C	24	11457	4402	10478	8752	11730	6408	21645
FOXO6	24	167119	101876	136983	102679	218792	54893	506027
MIF4GD	22	3593	1536	3351	2772	3803	1810	8653
ESPN	23	6775	2997	6885	4976	8698	464	12333
SNURFL	23	18037	6240	17637	14543	20771	8347	32923
ZNF487	23	11937	7712	9746	7100	13776	2887	32812
OR11H12	24	79480	63460	60028	50971	76532	23803	280588
ZNF732	24	10640	4011	9666	8202	11436	4985	20694
ANKRD66	24	26523	7177	25891	21960	31440	14232	40555
KIF28P	24	18346	3427	18126	16724	20386	12826	26996
C4orf51	24	20764	4795	19900	17319	23512	12278	31876

PG.Genes	n	mean	sd	median	q1	q3	min	max
HSBP1L1	24	33398	9491	32119	26237	38605	17445	56904
MCRIP1	21	5067	1609	5147	3668	5987	2501	9064
AKR1B15	24	9952	3773	9447	7202	11270	5003	20034
MCIDAS	24	24623	18430	17602	9031	37889	5022	68197
PROB1	24	47820	15720	45326	37537	52805	21635	81842
NACA	24	59885	6533	58787	54789	63483	50785	73676
C11orf98	21	2478	1423	2180	1271	2846	1169	5806
STMND1	24	21683	6980	21399	16746	24435	11266	42356
CROCC2	24	22224	6586	19330	17980	24193	16396	43359
TRIM77	24	81956	18060	80895	69805	91232	49857	128004
SLC35A4	23	4933	3125	4326	2605	6878	720	10816
ASDURF	17	5553	2094	5206	4249	7208	2058	9140
OVOL3	23	19247	9179	18640	15804	26748	1227	36534
DNASE2	24	13564	8083	11806	8022	18060	2085	30550
AGPS	24	9765	2697	9182	8167	11786	5518	15206
DDX39A	24	167828	62118	157928	131863	201845	64493	352506
PDLIM1	24	75166	30070	67378	51579	94312	34966	144172
AIP	23	4233	2038	3747	2573	5669	1265	8476
GTPBP1	24	41342	17000	38418	30106	52241	9671	78113
STXBP3	24	115222	40212	111658	95251	126259	61002	249711
SMAP	24	6503	2049	6432	5339	7215	2980	12829
LGALS8	24	176330	99010	150881	137018	174006	80813	584266
NFKBIE	24	7664	1658	7377	6869	8622	4945	10952
PSMD11	24	19706	9087	19989	14682	23609	5194	49371
PSMD12	24	16907	7338	16342	12756	20542	5977	38869
PSMD9	24	19156	4567	19523	16564	21835	8978	29083

PG.Genes	n	mean	sd	median	q1	q3	min	max
RNF103	24	33220	11315	32637	26000	37181	13312	64442
ATOX1	24	27269	14468	25339	15944	34413	4565	53777
MEN1	24	16808	5759	15662	13648	18799	8765	37920
PGRMC1	22	2662	1238	2216	2047	2985	680	6553
TAF4	24	412551	95158	412267	338976	464656	255482	639192
DFFA	24	11212	3717	12527	8426	13506	4942	18278
CLIC1	24	307581	123359	283459	221501	382905	115150	616187
EIF3F	24	18129	9934	15867	12769	22230	3866	39616
QSOX1	24	20217	14153	14357	10633	28234	7068	67294
DCTN6	24	6744	2358	6660	5116	8647	3068	10131
WASL	24	66867	18715	62175	53189	76749	45706	128210
PDE2A	24	393956	91199	357849	320825	468911	268190	571009
FOXN3	24	19124	3353	19689	16761	21551	12758	24416
POLRMT	24	151606	29862	149605	128791	163757	107066	243398
EEF2K	24	91797	23667	88378	72435	104561	56179	137132
EML1	24	34626	14977	31009	25277	44393	13107	65200
IGF2BP3	24	60750	16362	57773	52489	70609	36260	102038
GOLIM4	24	44475	25818	40771	27192	51213	12639	108643
EXOC5	24	56607	24721	45192	36925	73784	28273	118056
HMGN4	24	41535	41645	26352	22605	41080	16725	181871
PSMD14	24	19346	8706	19490	13688	22403	6530	48547
KPNA3	24	21645	11960	16912	15642	23495	11114	68235
USP9Y	24	53323	7589	53312	49181	57753	34456	68447
LAD1	24	227664	115770	184152	152894	297239	66846	559891
VWA5A	24	10937	6679	9073	6256	15405	2742	26697
NOP56	24	20583	5107	19909	17483	22550	13543	36630

PG.Genes	n	mean	sd	median	q1	q3	min	max
RNASET2	24	68442	55396	52753	28983	87279	7706	218042
GABRP	24	17295	9050	14797	11579	19624	3936	38146
PODXL	23	7980	5770	7266	3816	8910	1666	23757
FCN1	24	11421	3636	10919	8515	13738	6625	18979
PIR	24	11955	6386	10613	6896	14580	4267	26032
KPNA4	24	13898	6135	12412	10672	17058	5407	27971
NFIB	24	43768	35355	29613	25615	44688	19822	179101
PPP6C	24	3950	1515	3738	3002	4549	1675	8569
PIK3C2B	24	408820	129581	368991	317184	457332	226223	703596
FBP2	24	20534	9124	20495	14648	24576	5772	43016
UBE2C	24	36508	14482	34358	28496	38849	14651	74903
PDXK	24	24061	16868	22590	15028	29372	4849	86066
SCD	24	12000	5926	10997	8473	15344	1897	25995
ISLR	24	5538	3979	4083	3230	5715	1604	17814
NCKAP5	24	65455	16660	62698	54895	71022	41309	106246
CUX2	24	79693	19436	78003	68294	90414	43152	122884
SOCS6	24	166345	108872	122339	108722	213709	55529	557355
TRAFD1	24	27888	22423	18815	15432	32058	7979	102414
GAPDHS	24	9806	4077	8254	7480	11723	4712	20672
HSPB6	23	32695	63236	19184	14283	25782	1267	319686
NDUFAB1	24	19805	7336	20151	15321	23505	7927	36323
UBFD1	24	10388	3625	10830	8180	11987	3972	18687
COPE	24	50744	22102	46721	36244	56775	14804	114872
CCS	24	15648	3210	15500	14071	17529	9862	22810
ENPP3	24	111371	33419	112532	88483	143295	53476	167015
DNALI1	24	3607	985	3738	2876	3967	1864	5822

PG.Genes	n	mean	sd	median	q1	q3	min	max
ADAM10	24	9504	16888	5358	4421	7141	2418	87542
MAP2K7	24	188123	25946	185322	166358	206828	152096	253808
PDCD5	24	26276	12766	22991	17470	35690	5668	52654
SLC9A3R1	24	59354	33730	59382	35270	81140	10811	151198
TPP1	24	12222	6718	10972	8199	12775	5561	39445
TCERG1	24	314773	72909	299183	262389	347083	209701	485643
KIF3C	24	27670	9656	25105	21235	31972	11106	53829
UNC13B	24	20348	5086	20520	16052	21934	12992	32374
POLR3A	24	24350	6755	23371	20225	27503	12817	42118
PSMA7	24	95144	34391	87843	71580	110671	40193	180889
OPLAH	24	23176	6340	22038	18408	25771	15464	38369
SLC30A4	23	24357	11542	19664	17089	31322	9153	56141
IFIT3	24	278827	69974	263700	235733	331926	178116	443773
IRF6	24	25953	13919	22279	15003	31951	9253	55756
TAX1BP3	24	47553	21463	41316	35338	53256	23414	114601
GIPC1	24	10201	2934	10180	8028	11679	5519	16052
HAT1	23	5269	2506	4511	3796	6689	907	9909
MYL12B;MYL12A	24	22402	17426	17245	8504	36325	2462	58040
AURKA	24	16735	10815	14680	10670	18062	1341	53334
CLGN	24	34060	19063	31237	20608	43305	8276	79409
PPP1R12A	24	10408	6230	8684	7152	11296	5053	34515
AZIN1	24	189632	127865	179667	73076	295544	34837	520072
XPO1	24	57262	32947	50635	33455	74484	8737	128142
PLXNB2	24	135612	26144	135617	114847	150363	93375	200777
SEMA3E	24	155688	46231	145961	131042	164231	98591	310930
U2SURP	24	19994	4769	18978	17013	21590	12039	34891

PG.Genes	n	mean	sd	median	q1	q3	min	max
SETD1A	24	66989	20544	61507	53042	80073	32726	108464
KIF3B	24	26550	4345	26249	23722	29030	19254	36449
PFAS	24	47281	24895	38620	30161	65174	13303	100112
NACAD	24	110639	22062	114487	93448	123892	65673	154622
ADAMTS3	24	16548	5945	14314	12501	19322	10329	31549
LSM1	24	16703	6476	15434	11738	20136	7447	31475
ARPC1B	24	39899	14068	38442	32260	42954	18621	85517
ARPC2	24	87041	22408	80496	67776	101881	63748	149314
ARPC3	24	31181	10109	29739	23158	36879	18366	55206
TBXT	24	29043	14722	25406	20994	32681	1710	77124
PFDN6	24	21747	6734	22403	15845	25331	9521	34621
LAMA5	24	72161	13440	68436	65613	74444	56453	113945
CASC3	24	53475	35894	41876	30088	63166	18334	161907
CLIC2	24	10344	17446	5796	4352	8097	3059	89863
RTL8C	23	12308	6616	10729	8695	14341	4049	33833
MAPK13	24	42091	12697	39635	34131	48866	23645	79403
SPTLC2	24	92303	15746	87386	83648	97499	72211	141302
FANCG	24	10145	4926	8515	6381	13281	3131	21562
OGT	24	2980353	593558	2856425	2612277	3232864	2162640	4476495
PMM2	24	26706	12676	24664	19806	32858	6735	52492
INPP4B	24	41005	8969	41112	33361	48915	25605	55329
HMGB3	24	58054	34144	65304	34478	81120	7151	137877
PPM1G	24	155762	70296	150965	105010	212963	32416	280314
INPPL1	24	35611	8231	35215	29549	39705	22245	58525
EIF3H	24	24284	11547	21714	17693	28573	7965	49488
NVL	24	89172	21087	83592	76957	96959	62694	157841

PG.Genes	n	mean	sd	median	q1	q3	min	max
BCAT2	23	4956	2116	4302	3383	6101	2662	10996
BCAT2	24	46458	15882	41444	33808	53604	28397	89954
STX7	24	14092	6973	11611	8154	18141	6793	29771
SLC16A6	24	17216	5444	17427	13107	20773	7037	28185
CAPN5	24	14336	4715	12697	11207	17325	8878	25217
YKT6	24	19333	6113	17744	15259	24050	11493	30894
ARPC5	24	42395	11225	38810	34104	46165	29337	70350
POLR2D	22	11011	2609	10464	8862	13028	7200	15221
NKX2-8	24	15449	3829	15532	12226	18949	7941	20867
DDX3Y	24	16517	6575	16110	10460	20740	7877	31886
RGS5	24	827499	410545	774595	608025	892767	413831	2529582
DHX15	24	13886	6367	13766	9816	16930	3060	27021
ZZEF1	24	125089	16256	126688	115835	136039	83300	153131
FLRT2	24	8833	5614	7825	4805	12497	1957	24643
CYB5B	23	2471	1756	1942	1404	2543	1007	8047
CYP26A1	21	12013	4406	12584	9245	14569	4500	21298
PHGDH	24	89710	52922	68526	55215	117493	19858	206354
GPR39	24	24977	25692	18722	13715	21361	8281	124409
SEPTIN4	24	42862	8898	41610	37341	48273	28927	64276
DYNC1LI2	24	52983	9013	54403	49701	57439	29158	67830
KLK10	23	3544	2317	3478	2125	3700	884	11685
PSMD3	24	20613	8909	20323	15798	24752	8394	51194
PAPSS1	24	7772	2463	7261	5842	9582	3862	13092
SART1	24	73605	14494	69324	65344	85224	51452	106151
TGFB1I1	24	34503	31828	23637	16153	39000	5599	145554
SRGAP3	24	35270	8125	35198	29876	39835	19162	50937

PG.Genes	n	mean	sd	median	q1	q3	min	max
ZNF264	24	15238	3934	16031	12766	17202	7436	25660
HSPA12A	24	135487	44626	129299	115660	135355	93956	326238
MSI1	24	15636	3499	14599	13223	18162	10909	22360
WDR62	24	36403	7401	34048	31869	40555	23668	51320
PRPF3	24	9749	3060	8549	7650	11173	6141	17298
TXNL1	24	62927	22200	60762	48038	79983	23188	112917
ERI3	24	17005	5711	15495	13746	17200	10151	34387
FIBP	23	14249	3496	13390	11471	16723	10154	24396
EIF4G3	24	95915	19335	96730	81342	104994	69854	156251
PPIH	24	8470	4302	7783	5561	11288	1888	18434
HTRA2	24	21174	6292	20133	16428	25653	10732	32878
AKR7A2	24	13953	5285	13540	10309	15095	5851	28328
EPB41L2	24	68512	23881	64495	56310	70407	37726	136577
EPB41L2	24	132071	65195	130338	81779	150372	44023	272005
LAMTOR5	24	7615	3770	6954	5373	8548	3159	19478
WIPF1	24	78803	31834	65733	51100	101899	43698	148964
XRCC3	24	20669	10314	18067	14611	22049	8217	49731
DENR	24	13629	3707	13222	11557	16796	7165	20382
XPOT	24	55593	31999	44999	33520	69299	20167	147964
DNPH1	24	38255	9623	36355	30502	44137	25252	65573
DCX	24	9418	6023	8179	5743	9833	2844	25851
TIMM44	24	6183	1973	5569	4679	6838	4181	11220
TRAPPC3	24	78726	16920	80786	61506	91141	55740	111289
CHMP2A	24	18996	6707	17523	15835	23815	6858	34257
NCK2	24	8892	4519	7627	6435	10700	3838	25379
PSCA	23	10543	6016	11402	6116	13469	1625	28956

PG.Genes	n	mean	sd	median	q1	q3	min	max
TSPAN6	21	9124	11133	7015	4626	9222	2457	56336
PLRG1	24	13261	4680	13004	9371	15054	6079	26158
GET3	24	25460	9347	24064	20256	29340	11593	55695
ACTN4	24	132967	56432	116485	104960	158898	54310	294440
GSTZ1	24	56315	21557	52558	42240	69934	26760	113329
TRIAP1	23	7282	2035	7282	5462	8817	4022	10643
HTATSF1	24	5764	2567	5037	3682	7771	1626	9811
STX6	24	809025	118654	789672	730516	903271	607683	1007616
SYNGR2	23	16876	12316	12651	10401	18967	4978	65646
SYNGR3	24	9736	4352	9792	6360	11299	2190	19425
SGTA	24	21537	9101	22215	15147	24273	5581	42909
NARS1	24	53662	21719	55433	40335	69840	14128	100057
NUDT21	24	69244	32039	65582	44382	82368	29401	154132
LANCL1	24	23117	9633	22587	16995	25815	7219	52082
RRP9	22	3494	1697	3248	2532	4373	1153	8739
B3GALT2	24	25930	11807	25322	18113	29729	9610	64054
SLC37A4	24	18329	8161	17990	11786	20229	8447	38384
IDH3B	24	45404	15468	44104	36109	48212	28373	101062
AHCYL1	24	42974	10198	44788	33180	51151	22110	58459
CD5L	24	13257	12511	8863	6142	17142	1957	57427
NDUFS5	24	7991	3913	7421	5737	8698	2275	20380
PDE6D	24	5301	2203	4814	3798	6956	1440	10004
RAD21	24	22562	7199	21106	18180	24835	12640	43917
DHX16	24	12206	4179	11705	10112	13512	5249	22520
ZNRD2	23	3065	2324	2674	1653	3661	436	11984
GMFG	24	27678	11862	25696	22574	28611	11615	66667

PG.Genes	n	mean	sd	median	q1	q3	min	max
PPP1R12B	24	14871	3848	14539	11710	17614	9245	24005
BNIP3L	24	3596	1256	3673	2702	4569	1110	6162
ADGRB3	24	14344	4260	13917	11655	15443	7458	26304
PCDH7	24	41052	6998	40434	35740	43777	31370	59227
SMARCA5	24	45184	11930	39414	35724	55046	30719	66878
KIF5C	24	33508	4972	32176	29355	37395	26153	43496
NUAK1	24	27274	8151	25181	22268	32097	9835	50353
ZNF862	24	212821	52100	200487	181052	236384	147569	386243
SIPA1L3	24	42398	8994	40101	36607	46196	31134	65642
MAST3	24	165391	72225	148047	125372	182378	108985	449197
OPA1	24	107857	15123	104661	99159	111218	80908	155334
PPL	24	191463	42431	176010	165466	222465	108411	279330
GSDME	24	68327	22732	65078	56941	77170	36661	145967
PLXNC1	24	37632	11671	36232	31020	43392	18417	67222
ACSL4	24	63961	15587	61913	51248	74544	35344	92680
SNX3	24	12666	12101	8407	5878	16383	1509	57532
CUBN	24	21717	3890	20998	19326	23058	16508	32033
DOK2	24	20748	11576	18945	12529	24427	8318	60500
SORBS3	24	509183	484679	308865	259424	452173	152219	2352666
CDC40	24	43630	11040	43430	38901	51766	21532	68544
RANBP6	24	33721	8221	33257	28265	38442	16921	48740
CCNT1	24	548389	240090	509110	347115	660723	207103	1052714
PLOD3	24	6594	2751	6098	4354	8416	3268	12274
CCNT2	24	65068	13289	66013	55164	74424	40638	89313
TLR5	24	13862	6364	11651	9324	17187	6206	32478
TLR2	24	1472442	709130	1348926	996696	1790053	450899	3005113

PG.Genes	n	mean	sd	median	q1	q3	min	max
SELENOF	23	2888	1740	2519	1720	3189	1052	8037
TSPAN1	24	9275	5884	8537	5457	11408	1092	27036
EXOC3	24	47933	12277	44948	43057	51333	22233	89750
JAK2	24	88243	22769	83405	73849	94434	49920	150951
MAFK	24	217020	41751	219167	186242	249168	134832	276984
KPNA6	24	3884	2066	3603	2336	5450	413	7334
SRPX2	24	11854	5807	10337	8233	13613	4103	26448
UGDH	24	111554	66414	115491	64577	148037	14159	312307
SNX2	23	5305	2205	5200	3894	5958	2136	12170
SNX2	24	10959	3571	10783	8956	12348	3191	22455
HPGDS	18	2072	946	1814	1462	2547	621	4101
DPM1	24	15882	4511	15058	13093	18359	8459	26639
CCDC22	24	26314	6162	26336	23049	31331	15091	36560
DKC1	24	7685	2855	7216	5992	8453	3026	16339
EIF5B	24	11943	3948	11288	9419	14074	5906	23341
MMP20	24	12876	2376	13095	11478	14593	8129	17525
DNAJA2	24	28055	6233	28658	23719	32275	13187	37287
BRD4	24	8874	1937	8782	7360	9903	5826	12544
CUTA	24	9248	3465	8644	7315	11955	2984	15838
CTSV	24	13588	4169	12437	9838	14738	8817	24479
PFDN1	24	13754	3990	13859	10574	17094	6203	20672
PPP1R11	23	3888	3011	3032	1946	4935	1557	15210
NBN	24	94288	29000	90132	71845	116916	50416	157729
DTNB	24	47029	13951	46017	40501	54345	21095	76969
KIF21B	24	18781	3336	18288	16311	20651	12859	24736
SRGAP2	24	65596	32722	55375	44987	71990	24315	149179

PG.Genes	n	mean	sd	median	q1	q3	min	max
WDR1	24	91148	57968	84381	59415	96530	19375	274541
N4BP1	24	29228	5468	28492	25299	31043	22318	46253
ROCK2	24	113306	25753	110547	96209	132299	62811	169078
CLASP2	24	282798	82302	250903	229119	320704	199448	570856
CPNE3	24	19235	9732	17353	11114	25494	5658	41052
CLUH	24	18200	10439	14643	12920	19972	7729	50739
TSC22D2	17	2911	1586	2586	1693	3516	1097	6821
CNOT3	24	26691	6186	27146	22796	30244	13484	38419
GGCT	24	77850	36860	68072	46854	101725	21109	154601
ZPR1	24	12831	3150	11682	10795	14802	8119	19483
NIPSNAP2	23	27978	18247	23703	16765	34570	5381	86985
CILP	24	32588	4937	31235	28865	34323	26982	46194
PDCD6	24	11343	5374	9362	7375	15124	3504	23894
ZNF253	24	28219	6573	27980	24140	30378	20178	44164
TBCA	24	84823	32122	90222	60114	97981	35124	154070
ATP6V1G1	24	16097	5453	15813	13058	18946	4140	26491
VPS4B	24	15322	7326	13827	8934	20245	5790	29461
ZNF217	24	456027	173335	413789	340574	483222	242369	892206
SH3BGRL	24	171929	164453	115493	95895	171239	63695	848794
FLNB	24	604872	266349	599824	397692	702775	232106	1229552
NCOR1	24	540845	91527	519508	499226	545110	417067	862176
NDUFS6	24	41619	17444	36710	31634	48761	21557	101299
ULK1	24	102877	28858	96654	86851	114046	61660	197654
CS	24	71346	44956	52333	40275	102931	15999	208494
SEC22B	24	17743	10921	14350	10453	22968	4426	46123
POLQ	24	76532	12415	72809	69310	85360	59429	106580

PG.Genes	n	mean	sd	median	q1	q3	min	max
VPS26A	24	41847	11926	39166	33221	50612	21408	69999
PMPCB	24	19814	6578	17753	14885	24409	10869	34414
TECTA	24	35824	8988	36701	29386	42509	16972	53020
KATNA1	24	24923	11513	20690	17923	28505	12105	56947
RDH16	24	6190	1414	6330	5192	6710	3335	9646
ERN1	24	25319	10842	22224	17289	31433	12406	56162
PSIP1	24	19402	10938	16659	12485	23076	8275	60284
CLN5	24	13530	4708	12709	10563	16172	7076	28752
HSBP1	24	11454	3863	11158	9327	13152	5092	20009
KHDRBS3	22	14667	7694	12506	9187	18472	5062	35837
RBMXL2	24	38382	5275	38754	33962	42387	28716	46664
BANF1	24	109928	125463	74227	50820	130377	11664	616589
SF3B1	24	32233	13038	28596	25298	36486	14927	72446
WBP4	24	8214	7622	6047	4867	7761	2192	40075
SCGB2A1	16	9419	13305	4634	3347	7678	841	54339
SKAP2	24	41766	19881	40616	30354	52913	4186	89376
PGLYRP1	24	17662	37043	8877	5468	12467	2194	187963
NPM3	24	10636	6425	8709	5889	12031	4056	27571
LEFTY1	24	39801	60582	22707	14579	36879	1725	292273
CREG1	16	5265	3012	4471	3258	5603	1908	12895
SNRNP200	24	20472	5228	19451	17405	21400	13904	37850
TIPRL	24	74364	27428	72494	60496	84238	30105	166844
RFPL2	24	38344	7459	36956	33697	42388	20763	55720
PPM1B	24	12050	6817	11982	7211	14951	3125	33813
UTP20	24	98053	11991	96009	89416	106855	78336	126460
RP2	24	11522	9589	7765	6557	11861	4527	48338

PG.Genes	n	mean	sd	median	q1	q3	min	max
GJB3	23	12226	8961	9404	6476	14080	4069	43437
STK16	23	84394	68337	60244	41322	95204	23328	275050
CRTAP	24	12762	4332	11212	9865	15412	5593	22077
B3GALNT1	24	46330	25386	38538	33589	46988	20786	144146
TCEA3	24	24454	6200	23118	19824	28126	13559	40163
PALM	24	7075	2854	6213	5801	7286	3699	17696
RNASEH2A	24	71122	12896	67417	61739	81658	52594	102942
EIF3G	24	25439	8768	24835	20216	31509	11573	42310
EIF3J	24	15026	4499	15937	11130	18768	6918	21763
CBR3	24	32509	41198	20583	13538	31389	8821	207707
PSMD10	24	30370	10001	30692	21590	37546	15200	47919
ZMPSTE24	24	61156	23576	53853	47453	65334	35971	145969
PPP1R37	24	48593	14223	46877	40326	54155	24359	84150
IDH1	24	373952	206454	405109	214659	485513	80930	899168
GATB	24	6322	1377	6377	5198	7205	4064	8843
SCO1	24	9561	4060	9066	6407	12461	2245	17966
ATRN	24	23749	10408	22312	17781	26725	8157	52768
RBBP9	24	40511	18907	39498	24491	48788	13096	85719
STAM2	24	22966	5195	22269	19244	25936	13046	33019
SULT1C4	24	6249	2887	5953	4352	7596	2074	15373
ARL6IP5	20	3734	1661	3994	2155	4875	1234	6837
DCTN3	24	8243	4000	7675	6506	9366	2662	22646
DNAJC8	24	18010	6820	17391	13398	21122	7925	39191
ATP5PD	24	15859	4394	15454	12673	17711	8009	25286
SASH3	24	8378	3055	7996	6530	8912	3724	16796
GLRX3	24	43066	15190	43254	33291	50804	14471	78101

PG.Genes	n	mean	sd	median	q1	q3	min	max
RSL1D1	24	94769	35440	82022	72292	95218	58177	180153
WFS1	24	7278	2633	6524	5155	8717	4587	14477
SNCG	24	22737	21356	16175	11819	19907	10325	89694
CIAO1	19	2701	1579	2363	1407	3936	670	6167
DFFB	24	8515	6068	6959	5639	8967	1907	32024
SRP72	24	4880	1611	4686	3849	5467	2317	9768
DDAH1	24	84644	55469	67856	46260	119615	16349	251402
MTA2	20	3767	1802	3384	2510	4531	1075	7775
KBTBD11	24	52135	26047	40973	35160	53651	21300	113470
ATP10B	24	86714	22241	82835	74545	93986	48230	141556
TOMM70	24	10676	3253	10014	8659	12021	5695	20730
IPO13	21	7125	4668	5684	3873	10972	175	17508
TOX4	24	5504	1475	5237	4505	5950	3609	10267
SEC24D	24	7579	3442	6772	5023	9114	3067	16528
FCHSD2	24	27658	20453	22984	15895	26856	12709	96151
SASH1	24	289108	72056	280537	229801	321677	203994	434866
TMEM63A	24	56680	18162	59582	45475	67205	23591	97870
UBXN7	24	248244	107502	219199	162805	321358	120838	504450
SUN1	24	52263	18741	44154	40991	59451	28007	93913
PLPBP	24	14610	11280	11921	8334	19246	2844	55338
PCF11	24	67607	19910	60084	54393	80685	46890	129053
ENDOD1	24	40096	14079	35888	31610	45179	19087	72985
GLCE	24	27425	10392	25084	20062	33633	12220	48635
FBXO21	24	8334	3079	7762	5982	9714	4299	17237
RHOBTB3	24	41323	13533	36741	32372	49230	25535	73092
USP19	24	24310	8748	20802	18847	32463	12459	44438

PG.Genes	n	mean	sd	median	q1	q3	min	max
AP2A2	24	7043	3231	6568	5352	9391	1297	13871
CLSTN1	24	41506	30601	31477	18807	56020	13792	142233
INMT	24	12744	3944	12086	10483	13335	6005	24232
AGFG2	24	11732	6469	9560	7269	14577	2450	31961
NKX2-2	24	17034	6601	16239	12713	19488	6466	33755
ZFPL1	24	28545	13585	27283	23752	30880	5211	78859
ELP1	23	29891	17037	26400	18762	34076	5141	70859
NDUFA7	20	3068	1694	2509	1904	4017	795	7367
ZNF205	24	29058	6678	28886	25851	30832	17303	48540
STBD1	20	7833	5014	6446	4106	10419	2027	20694
ZRANB2	24	65964	17741	65106	53362	74418	33427	113072
SNX4	24	44248	12732	42586	37907	48823	21824	83357
OR6A2	24	228790	72958	221712	169470	269039	128558	435944
LUC7L3	24	59001	13787	56007	49919	68608	38864	91399
LRAT	24	8776	5049	8529	5145	10781	2531	23185
GOSR1	24	7782	2148	7595	6295	9219	3957	11945
KCNH1	24	14526	3353	14695	12250	16334	9200	22129
LYPD3	24	4773	1937	4584	3828	5904	1082	8750
VAPB	24	6576	2657	6292	4112	8518	2791	12402
PGLS	24	86040	30287	81542	63249	101515	43756	156158
PAPSS2	24	11602	2488	11143	10114	11685	8978	18532
ATG7	24	23754	8060	22315	18413	28943	11232	43423
LYPLA2	24	14270	4044	14413	12462	16185	4796	22527
IPO7	24	27815	11674	27348	17478	32304	11237	62840
ARIH2	24	25881	11895	21221	18537	29179	13702	64861
SLU7	24	49476	16504	48423	38742	58097	21214	83331

PG.Genes	n	mean	sd	median	q1	q3	min	max
CD2BP2	24	20155	5003	18950	16417	21527	14732	31227
BAG4	24	11408	5667	9894	8334	12547	4403	31653
AHSA1	24	33818	10570	33569	27620	41313	11241	50627
SLC34A2	23	5744	3168	5339	3678	6453	1427	13915
ABCA1	24	23413	5851	22328	19122	25889	15281	35835
H6PD	24	19019	9290	15792	13787	19493	9524	44349
SEC24A	24	33118	10499	28610	27697	37304	21238	70280
SEC24B	24	402805	157851	357938	278130	465812	200991	758088
VNN1	24	67692	29801	64046	45362	79865	14036	138979
PRAMEF12	24	293011	139960	264679	200070	366657	89804	649002
C1orf105	23	15671	9815	11531	9144	19361	5893	37716
ETHE1	24	17716	12791	13399	9954	21285	5222	57401
ZBTB11	24	75473	35933	64462	57388	88595	26072	203659
STAMBP	24	8316	3539	7635	6641	8291	4976	21681
ECEL1	24	53255	11881	51705	47233	59210	33798	78756
RAB3D	24	16436	9403	14257	10698	17175	8254	54079
OXSR1	24	42709	11473	45005	34400	52414	22599	58454
GGPS1	24	21269	17021	17678	11141	23135	6439	88158
LSM8	24	22496	8313	22528	15726	28978	8800	39056
AP2A1	24	47043	13624	43018	38891	55137	28467	81269
WIZ	24	22955	5505	22520	19572	24844	15019	42091
DDX58	24	18611	5521	17081	14745	21238	11684	34080
CAVIN2	24	33794	12632	31437	25290	39641	17374	79791
BAG2	24	8194	4935	7099	4224	10168	2328	21961
BAG3	24	5858	2152	5513	4307	6154	3406	13187
CRYZL1	24	6343	3138	5385	4791	6342	3973	18818

PG.Genes	n	mean	sd	median	q1	q3	min	max
AIFM1	24	10952	9057	7911	4749	12833	2053	38841
EML2	24	16830	10229	13905	11501	19440	5450	55485
NUDT14	24	7091	3454	6259	4852	8103	3282	18858
TSPAN15	24	11985	4981	10396	9281	13292	5238	26187
BPNT1	24	58119	29169	50458	41831	74193	12145	154192
DDAH2	24	77191	44694	71678	51733	92519	22175	234040
ABHD16A	24	5214	2132	4718	3610	6867	2022	9397
TXNDC12	24	26088	13072	23737	15936	35599	8747	54022
ECD	24	77096	18306	75828	63626	90684	36461	106867
EFEMP2	24	16278	17318	11366	7070	17285	2959	84046
SCGB1D2	19	6165	7614	4179	1064	6625	468	30860
RECK	24	12110	5156	10783	9170	13439	4730	25844
NUDT3	24	19761	8970	17826	13352	23543	8521	45919
AGR2	24	35065	31568	25242	16812	39891	4216	121458
PAK4	24	12724	4274	12406	9462	14110	7462	23430
APBA3	24	26036	14191	23632	13423	33297	9468	56210
ACTL6A	24	100064	58637	75970	58306	127023	32685	240552
MOCS2	20	2898	1228	2741	2265	3452	577	6455
CYB5A	24	7010	5172	5981	4632	7732	2202	28250
ADH1B	24	145478	206000	87212	62809	113719	35805	808961
ADH1C	24	53664	28659	45672	37042	59995	28125	161980
ALDH1A1	24	163156	219679	98097	58886	143376	19896	1064589
SOD1	24	273031	97376	241778	220800	301382	161070	594656
CP	24	115900	63003	100369	72466	139286	27874	316668
PNP	24	118379	39537	123262	88590	141163	28248	203739
HPRT1	24	66356	39319	54522	45692	64163	29749	175933

PG.Genes	n	mean	sd	median	q1	q3	min	max
GOT2	24	38180	18052	35329	23692	46389	15234	82075
PGK1	24	1152070	337959	1141819	920693	1406460	528546	1721144
AK1	24	73379	32465	70469	53621	84945	33159	201563
C1R	24	16314	8272	14511	10336	18618	5153	34359
CFD	24	8031	6684	5589	3827	8567	1263	29339
PLG	24	26446	21979	19335	10233	30024	3602	75490
PLAT	24	9346	2916	9375	7684	10482	3059	16635
CFB	24	131938	64227	124468	72903	170778	44629	275805
ADA	24	13601	7384	11838	7721	17209	4017	29775
CA1	24	1032600	766915	840442	526643	1313931	161460	3180321
CA2	24	240746	163633	195903	126865	306266	53385	671872
ASS1	24	46201	57424	27817	18248	43144	13215	291194
SERPINC1	24	23411	25294	12749	9042	29206	2965	118257
SERPINA1	24	1246636	831607	1037973	791296	1424336	87940	3924526
SERPINA3	24	178750	183387	88834	57127	206814	30646	679142
AGT	24	67291	34828	60212	46049	66775	17143	162876
A2M	24	490451	366774	392953	292671	482823	78966	1447319
C3	24	342336	176207	288981	218029	453282	106201	712666
C5	24	23446	16458	18272	14640	26631	3178	60816
TIMP1	24	21409	16012	15691	10580	30404	5013	74882
CST3	24	105013	74296	76184	47623	145146	29283	287960
CST4	24	5380	2788	4788	3797	6709	1894	15463
CST1	18	3018	1682	2939	1746	3866	845	7354
CSTA	24	41596	41539	30285	25028	44710	11053	226177
KNG1	24	12807	5430	11450	8728	15636	2259	23467
KNG1	24	149996	94336	124946	77139	187907	36737	402540

PG.Genes	n	mean	sd	median	q1	q3	min	max
HRAS	24	6770	2859	5761	5016	8193	3646	15292
KRAS	24	14219	6085	12858	9725	17380	5731	27655
JCHAIN	24	97332	110969	55378	32638	94443	8439	428240
CD4	24	43733	11967	43224	35798	50968	26903	74709
PIGR	24	766661	1416409	211612	38078	828048	3216	6337926
HLA-DRA	24	4868	2963	3618	3306	6122	1200	12886
HLA-DRB1	24	10335	4045	10161	7360	11992	5915	25287
COL1A1	24	90402	21495	85376	76167	99384	59501	135617
COL3A1	24	95583	14755	92443	84815	101977	75799	124956
COL4A1	24	102165	21054	96641	88611	108924	79413	160473
CRYAB	24	26746	12366	24625	16638	37183	2949	47127
LMNA	24	121219	208387	52130	36169	97718	27015	916572
APOA1	24	920687	702341	699557	447165	1067039	145610	2421268
APOE	24	29485	20060	22853	14577	41552	6262	74975
APOA2	24	392498	323271	296564	131734	536476	33820	1306622
APOC1	21	14151	15316	6844	3310	22745	1883	57692
APOC2	18	18320	25442	8291	3510	22100	1175	88625
APOC3	24	32870	38219	14792	7334	50914	1688	133614
FGA	24	153481	101616	130419	84558	177327	32145	403562
FGB	24	243389	206831	202892	93334	254128	40299	756826
PMP2	24	8177	4270	7069	4705	9284	4016	18104
SLC4A1	24	14355	25723	7934	5318	12032	3576	133052
CRP	18	3878	2721	3352	1900	4980	570	11741
APCS	24	133530	291166	45461	33820	74807	7927	1190268
C1QA	24	35941	23242	30844	20974	41737	5250	88031
C1QB	24	23111	13359	19131	13616	30906	7966	55731

PG.Genes	n	mean	sd	median	q1	q3	min	max
C1QC	24	19961	12748	16076	10069	28875	6318	49687
C9	24	56066	36276	48570	30789	66106	11460	170433
APOH	24	99940	66582	79922	54234	122155	12768	302903
LRG1	24	70772	36412	68503	40253	77305	16278	167324
FN1	24	15755	20101	9724	8351	13193	2724	90982
RBP4	24	36423	24803	28407	20848	38383	5811	106631
AMBP	24	85300	48524	73635	49277	101855	16082	227563
ORM1	24	490116	318089	417792	283127	584314	112001	1390251
AHSG	24	136079	78031	118543	93423	158746	32648	371079
TTR	24	252208	145862	212188	149360	316439	40330	589070
AFP	24	9778	4535	9478	6408	10670	4592	25449
PPBP	24	35801	15948	32009	25066	43322	9815	74071
PF4	22	7341	6461	5089	2973	9771	316	27192
TFRC	24	18284	7279	17815	13484	21653	9282	36636
TF	24	1830694	1091889	1560215	1008875	2199909	412046	5560686
LTF	24	545471	780415	360295	178920	591024	15843	3961989
HPX	24	388110	237963	328847	209720	430242	81934	1067423
FTL	24	30812	13533	27781	23051	36638	8715	59694
FTH1	24	9970	8257	7768	6274	11585	2801	44431
ANG	24	154789	108391	113135	95694	182876	73396	529915
KLKB1	24	88936	54798	72720	49385	103310	25853	212760
SLPI	24	34379	61941	6963	3482	35594	1827	217037
C4BPA	24	270768	265204	154544	85694	321844	34540	982125
VTN	24	45692	32786	33160	26240	58448	7732	111283
CAT	24	241649	163658	202093	140565	251229	77353	729317
FUCA1	24	124484	100986	84990	61835	143338	32172	459151

PG.Genes	n	mean	sd	median	q1	q3	min	max
PROC	22	2814	1029	2828	1918	3253	1349	4757
ALDOA	24	469277	152801	508253	331862	555672	193066	765535
CSTB	24	394516	347973	309252	250548	403230	89026	1841219
ANXA1	24	237106	154952	189470	142326	308735	24538	622829
APOB	24	53789	45396	45237	25796	62123	6563	184756
PRNP	22	4200	4346	2774	1851	4941	1262	19469
HRG	24	37999	30000	27549	19143	52549	6469	134259
THY1	19	2151	1199	1761	1370	2464	706	5114
A1BG	24	60474	38585	50833	33791	68294	11568	157825
CD74	22	3772	3720	2634	1474	3903	543	13493
VWF	24	19987	8152	17783	14145	24433	10173	36756
SHBG	23	4837	2641	3843	2792	6604	1565	10975
GAPDH	24	803862	390587	681713	542816	899665	303462	1789046
GAPDH	24	1425969	509731	1251614	998348	1781755	812632	2509432
CAPNS1	24	66764	18767	60566	56207	69204	46355	121297
HSPB1	24	37077	41147	20438	14673	40498	5178	155559
CYBB	24	25641	6385	23926	21388	28516	14015	42539
RPN1	24	43180	20277	37021	33374	47440	14617	95369
ATP1A1	24	12552	3699	12497	10097	13974	7048	22910
ARG1	24	25791	30406	18988	15127	25152	4472	163315
APOD	24	43998	39915	27269	19925	50821	5132	137860
ALDH2	24	46060	35534	32080	19857	57956	7545	141089
ITGB2	24	12153	4603	10536	8971	13339	7217	23601
S100A8	24	489846	986657	281628	142463	391832	21022	5024907
HMGN1	24	95967	33474	94813	72185	121547	40685	160467
SERPINB2	24	13026	7907	10660	8617	14688	4145	31289

PG.Genes	n	mean	sd	median	q1	q3	min	max
SERPINA5	24	12729	16284	5728	3814	16431	1971	75292
CFI	24	39565	16968	35848	28014	50707	20195	92344
ISG15	24	12207	10380	10403	3449	15854	2157	41349
PCCB	24	36303	11035	34935	27724	40587	20451	60449
ALPL	24	30153	8642	29021	26016	34071	13700	48748
ALPP	24	9948	8460	7421	3364	14702	1548	37762
EIF2S1	24	31808	11549	31018	26884	39136	10575	55744
ICAM1	24	20334	6081	19449	16871	23663	10378	36754
RPLP1	24	26332	16019	23730	18217	30138	2041	71681
RPLP2	24	62568	29896	55194	42834	83689	16667	135453
RPLP0	24	25938	14187	24058	15245	32426	4017	57560
FABP3	24	171599	263883	72608	51406	167945	37519	1271003
POLR3D	24	242741	60258	224466	197706	259794	171341	373440
REG1A	20	3555	2396	2954	2024	4228	543	10650
CLEC3B	24	19693	29708	11886	8350	15519	2774	149742
SSB	24	107116	45210	102710	72477	137354	29461	182364
SERPINA7	24	10683	5930	8735	7227	12990	2276	25751
SERPIND1	24	99508	63352	84099	50782	108708	20072	226575
ITGB1	17	2440	4087	1026	906	1464	264	14433
PRKCB	24	47399	11235	46110	40580	50173	32231	78766
C1	24	10808	4709	10306	7652	12904	3924	20277
COL5A2	24	182329	30946	174191	167078	204176	130702	253019
UROD	24	19580	6565	17890	15400	26317	8467	31322
BCHE	24	7016	2647	6111	5300	7310	3806	13758
GLA	24	7729	4796	6860	3820	9431	2382	20903
GSN	24	334877	212244	241800	199957	368789	124394	872458

PG.Genes	n	mean	sd	median	q1	q3	min	max
GSN	24	249369	116961	244724	151211	270582	101663	594082
RB1	24	20701	4200	18685	18315	21878	14637	31464
PGR	24	16235	5374	14799	12796	19917	6845	28565
PTMA	24	320684	218168	250306	174888	421638	69018	914646
ATP5F1B	24	70359	38681	59515	46547	99735	8977	158550
C2	24	14271	6748	12608	9895	17215	4114	30250
S100A9	24	983344	1984131	559874	285749	809861	55352	10122203
S100A6	24	386774	231165	323390	278867	427010	126150	1049135
APOA4	24	53806	74071	25696	13627	47329	4298	332702
EIF4E	24	37173	16849	33747	26075	44352	12551	81151
ENO1	24	3478417	1124775	3529175	2431113	4321268	1583154	5585988
PYGL	24	41664	31176	34048	26478	44101	10176	154810
GPI	24	213777	65956	201234	168596	250552	89396	372204
POLB	24	3861	1527	3695	2890	4717	1158	8065
TPM3	24	55665	22380	49116	42740	67676	17058	118526
HEXA	24	21160	12070	17170	12475	27806	4855	50837
EPHX1	24	11756	14150	5582	3998	13056	2185	58778
LDHB	24	438214	259381	325262	272918	485744	230726	1106412
GPX1	24	88371	35706	80218	63301	108344	37374	199229
PGK2	24	171915	38237	169416	149071	200224	90557	246540
PROS1	24	19565	13515	14662	10514	26884	5267	53410
P4HB	24	122921	71767	94963	76431	173390	28244	290902
H1-0	24	100086	248544	36373	20039	68889	7462	1239912
ACYP1	24	14756	5969	12888	10424	17354	7281	28408
CSF1R	24	21263	6790	18864	17107	24848	11781	42130
CTSD	24	170752	193771	126147	80948	175514	56463	1027316

PG.Genes	n	mean	sd	median	q1	q3	min	max
ANXA2	24	281343	167828	245566	166428	355447	46176	678234
C8A	24	137110	76113	125122	89974	160798	25487	281448
C8B	24	44541	29062	36650	28037	51654	6323	105665
GP1BA	24	4837	2636	4228	3447	4755	2284	13409
C8G	24	6285	3703	5378	4260	7811	966	14377
CAPN1	24	77198	29399	74810	60770	85772	32809	173262
TUBB	24	82840	31587	80389	66815	100889	29279	148230
CA3	24	25551	39875	10481	7594	24127	4027	199730
IVL	24	28929	11354	26550	21103	35754	12202	51943
DCN	24	10691	19210	5834	3790	8026	2366	97522
PSAP	24	105343	65435	86664	67579	108138	34179	261268
HEXB	24	58909	29459	54950	38761	78242	24314	153312
PFN1	24	889766	327318	881035	650057	1044454	451984	1840274
BPGM	24	73779	52296	61089	41264	93338	12248	217125
APRT	24	74670	25144	70697	59028	86244	31774	132816
EPRS1	24	28579	13613	25790	18740	35676	6960	55860
CTSB	24	260332	157209	254347	127753	361827	28068	579792
LDHC	24	62832	53246	46277	35144	75919	16413	278629
HSP90AA1	24	439259	162955	467559	313030	514042	139991	753312
GALT	22	3097	1587	3030	1981	4252	794	6809
GALT	24	12176	6257	10566	8160	14223	5452	31471
HNRNPC	24	203924	134009	169925	130620	214898	90281	716785
LAMB1	24	17568	6614	15322	14359	18606	11734	44421
YES1	24	22415	6507	22564	17060	25436	12853	39770
TPM2	24	22777	19061	17244	13060	21887	9222	91411
FH	24	20798	11466	19433	12995	23875	5634	50325

PG.Genes	n	mean	sd	median	q1	q3	min	max
THBS1	24	21779	11660	19543	15225	27932	4529	59093
RNASE1	24	8679	4395	8405	5636	11636	2050	16301
COL1A2	24	62973	19286	56834	50073	71805	36103	103907
ANXA6	24	59359	65945	41060	26785	57217	9987	324997
RHOC	24	146336	47510	137217	117801	165734	84057	295952
SERPINA6	24	26623	17863	22993	16448	25060	4601	68912
SLC3A2	23	5948	4031	4946	3845	6541	947	18326
GUSB	24	17396	15008	11870	7695	21768	4493	73293
PFKM	24	127889	72710	97072	76379	183430	60663	337346
HSP90AB1	24	833882	303024	887555	625321	958568	248037	1510978
ELANE	24	81146	86144	78820	31039	104499	3149	443573
MMP2	24	7361	3519	6425	5616	8346	3059	18664
SOD3	24	35496	72737	12379	5417	24516	1397	327912
CTSG	24	15062	19720	9196	5572	17214	863	97282
ITGA2B	24	31277	7927	28887	26220	35542	18050	47232
LPA	24	5918	1428	5623	4842	6912	4056	9366
PLEK	24	10527	6941	9010	6883	10514	3923	39128
CD14	24	55961	23865	51826	40463	58952	27521	133798
COL4A2	24	43529	5721	42404	38802	47813	33950	56356
SNRPB2	24	5578	1870	5437	3962	6745	3001	10418
CFH	24	51119	31247	42539	28006	68871	13185	114240
SNRNP70	24	13610	5923	12428	9728	15886	3917	27822
FCGR3A	23	4748	7876	3277	1827	4185	995	40312
ITGA5	24	37894	11003	35295	30396	39384	24017	63931
NFIC	24	102606	56843	87658	69187	112411	53854	332874
VIM	24	426199	438484	289420	138586	585718	47775	2004308

PG.Genes	n	mean	sd	median	q1	q3	min	max
SERPINF2	24	30607	18961	25352	17942	34258	7083	76594
RPS17	24	76583	42796	59547	56059	89657	32669	236185
GNAI3	24	14268	5471	13766	11069	15896	7174	32920
ANXA5	24	687935	484834	575806	353923	806859	295357	2379344
IGFBP1	24	41794	28987	35678	23207	49290	6534	132474
RPSA	24	96548	33753	94212	78417	110226	40811	168833
SNRPA	24	24762	12308	21438	15578	29411	8306	62460
ENO2	24	597655	300727	577296	384051	784996	163887	1247502
SRP19	24	7162	3052	6865	4743	8576	3242	14209
GSTP1	24	906916	387253	799888	561155	1234814	413541	1597694
SNRPC	23	8852	5790	7146	6584	9713	1104	26466
MMP7	24	35014	31957	24202	15633	39372	10663	136851
MMP10	24	10085	4704	8601	7256	11166	4212	21290
LGALS1	24	166292	90187	151309	99758	212385	26020	346883
QDPR	24	21274	11828	18265	12403	25973	9455	60951
HMGB1	24	233612	137219	240903	148989	303951	42915	618442
RBP1	24	43590	36551	27742	12209	77429	553	107468
FBP1	24	22870	18070	18888	7976	35797	2034	70837
SPARC	24	13391	4881	12020	10482	15556	7579	24796
GSTM1	24	42791	47070	21601	15456	56361	3867	215361
TPM1	24	19394	8749	17818	12737	20857	11150	39513
ANXA4	24	324733	252933	234135	164658	370683	44149	934627
ANXA4	24	4760	3584	4017	2714	5537	1245	18480
HMOX1	24	5394	1420	5122	4489	6258	3183	8105
DLD	24	19160	7653	17529	13179	21904	10938	38810
SNRPA1	24	27049	14951	23219	16739	31517	10778	77799

PG.Genes	n	mean	sd	median	q1	q3	min	max
CTSH	24	33372	24686	23961	16351	42803	8467	97795
COX6C	24	11373	6281	9986	7668	13311	2625	25137
TACSTD2	24	21551	3763	20949	19021	23635	15730	30588
C1S	24	10996	5600	9680	7156	12372	4686	22885
PARP1	24	401542	225227	350871	212562	529492	163390	912346
IFIT2	24	31703	15690	29009	23746	32977	15136	95332
IFIT1	24	17786	6878	15296	12654	24182	8104	30529
ALPI	24	10750	6612	7897	5989	13786	4333	26915
UCHL1	24	23325	34812	7328	4458	19601	1816	121463
ALDOC	24	58190	36354	47258	38610	65506	25677	199395
NUDT17	24	132134	71862	114447	92822	152828	38770	388567
C4A	24	125445	83127	114142	67014	171593	23661	328477
C4B	24	9048	5755	8074	5071	11039	2417	29408
H2AZ1;H2AZ2	24	71576	56044	52810	40040	64832	23197	266397
WEE2	24	67393	36071	55718	42267	81001	25826	166739
OR4E1	23	15848	9076	14025	11299	16096	5066	39728
ANKRD34C	24	27735	6825	28729	24142	32845	12267	37923
DCAF8L2	24	96304	26091	89525	77918	124003	49320	151123
PABPC4L	24	8129	4273	6615	5237	10142	2429	21688
POTEJ	23	7878	4547	7623	4068	10410	2439	16680
THEGL	24	25577	6239	24889	21653	27547	16655	47829
SAA1	17	2947	2631	1768	1445	2749	597	7818
SPATA31C1	24	16658	2602	16028	15367	18796	11795	22128
HSPA1A;HSPA1B	24	385252	229521	327077	265183	438811	70383	1145273
CBSL;CBS;CBS	24	42166	20251	40703	29157	49868	17220	110770
CALM1;CALM2;CALM3	24	419934	133726	401653	321163	511527	173713	704650

PG.Genes	n	mean	sd	median	q1	q3	min	max
SCHIP1	22	34031	15351	35842	24544	44912	2679	61525
AMY1B;AMY1C;AMY1A	24	10537	8400	6520	4650	15367	1326	29702
FDX1	24	14347	6484	13002	10070	17395	3300	29497
RNASE2	24	12296	23145	6092	3745	9589	1280	117089
RO60	24	21873	10165	21984	13473	26226	5587	44450
GAA	24	46067	33526	30416	21191	67507	8067	122985
RRAS	24	9580	11431	6116	4833	10189	3015	59951
HLA-C	18	2190	1140	2089	1256	3013	541	4479
H1-4	24	20125	21221	13665	9589	21737	4631	107012
SPP1	22	6450	6224	4272	3232	8342	1012	29495
SPP1	24	13481	13945	9027	4508	16087	3029	55199
DLAT	24	516302	138226	473380	462585	583937	206166	833516
NR2F6	24	77592	78341	52285	43599	73945	15846	368114
TXN	24	722905	228596	678383	583280	856645	320753	1407347
COX5B	20	6226	14000	2816	1715	4285	1140	65183
CTSA	24	18084	8584	15726	13209	20415	6865	42870
MAPT	24	48769	10503	46406	40544	55835	30890	66195
C7	24	14177	16269	8762	5819	14696	3683	79409
PRKAR1A	24	22700	14780	19156	13503	26233	7986	69068
CHGA	24	6955	5825	4846	3209	7441	1602	21796
ALPG	24	5024	2758	4497	2999	5985	1150	11779
UROS	24	15977	6127	15061	12050	20122	5510	28771
ESD	24	135567	78086	112369	97861	144411	65983	420257
HSPD1	24	63548	38786	54728	32871	87656	16190	145830
CLU	24	907797	946999	405636	289404	1222400	165798	3875784
HAPLN1	24	5264	1929	5556	3214	6788	1976	8921

PG.Genes	n	mean	sd	median	q1	q3	min	max
HSPA5	24	121373	70002	94039	72047	157327	33688	287448
LAMC1	24	142415	131599	100573	86378	127218	72937	711732
ACP2	24	12021	5179	11216	8480	15645	4074	22419
HSPA8	24	385955	157053	358073	260650	500744	142066	712378
EPB41	24	19968	6139	18924	15293	25038	10756	31071
UMPS	24	9806	4798	8510	6272	11629	4100	21871
DBT	20	9859	4446	9948	7336	13118	1300	17540
PYGB	24	47325	37044	30813	21943	69529	7196	141997
MBL2	24	395524	148699	370515	303841	498100	112703	650022
RALA	24	15680	6043	15027	11309	19941	6189	26428
NAT2	24	48907	34167	40710	29113	59106	6740	176130
SPTB	24	214487	70676	205923	162461	231043	131700	404586
LAMP1	24	92864	28601	83704	73743	108130	57318	155027
TOP1	24	61131	25065	54265	42276	80193	31543	140493
UBL4A	24	3867	1185	3904	3137	4369	1336	6112
GNAT1	23	7234	3646	7300	5219	8886	1547	16797
MTHFD1	24	26376	11836	25264	17689	32222	7358	52924
ADH5	24	132691	85636	105756	92071	123093	35573	417412
CDK4	24	69320	17659	64300	56569	81147	44186	104730
CRYGA	24	11456	7678	9850	6515	11962	1907	33704
PABPC1	24	85339	39383	87003	56636	109988	17393	191143
PCNA	24	34499	16086	38512	22121	44769	2891	62089
HARS1	24	31553	11880	29939	23564	37209	12573	57299
COL6A1	24	9872	4781	8057	6602	12379	5140	26589
COL6A2	24	43620	13283	39639	36699	48106	24356	68700
COL6A3	24	234930	132877	198555	173093	234439	113890	686704

PG.Genes	n	mean	sd	median	q1	q3	min	max
IMPDH2	24	15784	7343	15751	9253	21214	5293	26765
TPR	24	96643	26601	94823	80644	104601	57949	191213
PIP	16	3373	3207	2295	1160	4502	298	12277
CKB	24	78752	111428	45274	15708	73314	3379	417228
ANXA3	24	145270	131765	125451	94292	142277	13718	723068
BMP4	24	42721	12518	38391	35304	48773	28119	76801
BCKDHA	24	17344	3867	16667	13894	20700	11847	23846
RNASE3	24	10719	13223	6990	3078	11500	1450	65830
ACTN1	24	290019	166166	223377	186515	298688	96036	776571
CDH1	24	44404	21519	39047	28983	60035	14891	92796
MYH7	24	41019	6744	40461	37668	43423	32105	63872
SRC	24	101821	41389	96144	75519	125814	32561	192403
PEPD	24	42349	17621	38015	32219	44143	21121	100104
XRCC6	24	33647	19874	28968	19769	40201	11403	89059
XRCC5	24	72607	37309	66346	41230	96098	21029	164142
IFI30	24	14544	10542	12862	6896	20520	668	40827
RNH1	24	160796	80931	146667	123814	159778	67211	495307
SCG2	24	241844	56354	226735	200310	276124	146685	361892
EEF2	24	228966	62013	221212	188258	258681	118528	362176
PDIA4	24	114701	65897	103211	69443	149319	15307	280138
C6	24	11551	7111	9709	6620	14713	3137	25403
TPT1	24	32844	11814	34864	24280	41253	13359	49849
ALAD	24	100428	41411	91902	78642	111022	46989	210879
LCP1	24	244714	195667	193462	163774	257784	70134	1037290
PLS3	24	185210	79129	179204	133780	223933	59857	391144
APEH	24	85950	26097	87963	69717	96701	29437	140927

PG.Genes	n	mean	sd	median	q1	q3	min	max
ETFA	24	7929	4925	5927	4897	8923	1225	20874
GYS1	24	27465	9305	25618	22429	30717	8649	48605
GTF2F2	24	19552	8770	18219	14451	25173	6508	39726
MIF	24	1234661	519605	1136951	926075	1580050	240289	2445593
CD99	21	14334	12991	8881	7483	17118	3492	54855
PRKCSH	24	60986	33025	46637	35971	81371	25269	148861
HCLS1	24	285195	99836	259309	207390	376215	147767	514773
FDPS	24	98894	48203	92305	72084	109621	20349	204735
CPM	24	16513	6902	15845	12201	19086	6848	34894
NID1	24	74042	34575	58176	51317	86481	38314	185703
AKR1A1	24	61244	27505	61220	42675	76616	5123	116460
PKM	24	1324079	470998	1505770	988114	1610896	418510	2108734
PKM	24	299175	132384	309548	220609	390466	57145	550814
ACYP2	24	10530	5366	9778	7011	11451	4977	29773
HSP90B1	24	235462	153324	167706	151994	268191	77883	648643
CCNB1	24	48047	23955	39938	33137	54049	17885	95405
IDE	24	29751	11934	26543	21254	35558	18412	72715
MMP9	24	36440	88254	13672	8226	31392	2650	444663
COX6B1	24	12972	12996	8326	6174	14609	4045	65173
HNRNPL	24	48194	26670	37946	33603	54366	23501	147040
DARS1	24	49928	16018	50637	38672	57340	26170	89483
IDO1	24	54448	61555	21335	17786	73959	9940	224371
JUP	24	19925	13120	17476	9218	23670	4974	53661
CPA3	24	12527	4730	11041	8808	15449	7334	24756
FABP4	24	23474	24027	15959	7571	26716	2786	101640
GLUL	24	17628	11342	14716	11030	23736	5590	51950

PG.Genes	n	mean	sd	median	q1	q3	min	max
AKR1B1	24	188972	156400	128663	64357	314856	27806	626610
RAC2	24	49101	37030	39158	24444	67671	5903	161437
CPN1	24	18798	10870	15281	11795	19490	8234	50162
GSPT1	24	26089	8148	28707	22222	31047	7235	37684
PGAM2	24	82846	18651	76936	70963	92864	61334	124281
EZR	24	96176	48342	103324	57811	121604	10757	195771
FOLR1	22	9456	9072	5423	2770	11549	1727	28531
UCHL3	24	10602	3406	10179	8185	12605	5893	17531
NME1	24	59791	31079	59667	38167	80664	9639	119334
GNS	24	22100	12135	20978	13057	27750	7914	55253
ARSB	24	78149	48941	60068	50514	80082	25630	199509
RPS2	24	9725	8621	7795	3975	10463	2404	38219
ST6GAL1	24	59116	19450	58248	45644	66459	31975	110995
DSP	24	69194	16172	69299	60190	75616	39914	103522
GATA1	24	222392	65145	213317	194442	246396	111707	366299
TIMP2	22	2315	709	2300	1892	2575	1144	3904
NQO2	24	11083	6718	9200	7703	12559	1539	31224
SELP	24	5580	2088	5376	3880	6045	2652	10278
CBR1	24	49615	33610	39301	33172	59626	17411	181146
ACADS	24	10384	3920	9741	8014	11576	5036	24262
NCK1	24	3613	2385	2754	1886	4464	1080	9201
H1-5	24	136131	123548	94607	46360	175565	16729	525801
H1-3	24	453019	844472	237596	114450	425269	62491	4299350
H1-2	24	23102	28775	13469	8709	21132	4851	129181
EPCAM	24	69046	13462	67615	58071	78049	49081	99159
POR	24	18377	5575	17684	15164	20631	9974	33376

PG.Genes	n	mean	sd	median	q1	q3	min	max
MGMT	23	7364	3707	6318	4799	8833	1308	15091
PLCG2	24	29956	6701	27227	25776	34445	22788	50193
FAH	24	17530	10092	15578	10880	21433	3341	42111
NAGA	24	13556	4219	12302	10247	16681	6709	21689
HSPA6	24	113187	56960	102694	77417	135027	51221	318156
GOT1	24	47691	23428	38844	30493	57476	23019	95733
BPI	24	100223	65419	85391	59274	109248	50373	364685
JUND	24	7683	3108	6936	5473	9103	3864	17203
CAPN2	24	70253	35579	53439	47048	76005	36018	146715
DES	24	40117	28713	31372	28698	41469	20343	158210
CTPS1	24	6753	2657	6769	4325	8828	3053	12442
DDX5	24	59669	37783	50599	34489	75199	20304	189894
PFKL	24	130197	58187	107841	94890	156152	46706	247996
GM2A	24	242464	136790	201652	159813	250945	120388	725005
LGALS3	24	86761	48854	73026	60691	108509	6686	194404
FLT1	24	34493	3796	33857	32500	36362	25998	41830
PSMC3	24	27537	11544	27130	19848	34497	8942	60924
TCP1	24	85153	39451	85081	65931	92827	27576	217215
PTPN1	24	49884	14864	44931	41786	53020	34110	94514
IGFBP2	24	14832	7915	14539	9569	19418	2469	34357
RPL35A	23	7627	2809	6586	5744	9553	2179	12964
ITGB5	24	11990	21498	7251	6303	8322	4689	112493
ARF4	24	20957	12335	21217	9721	28674	2995	52351
RPL7	24	19791	14910	14577	11809	22509	3546	71201
VCL	24	17756	14159	14410	11064	16532	6517	66439
LBP	24	19757	11646	16588	11503	22436	6257	51288

PG.Genes	n	mean	sd	median	q1	q3	min	max
PTPRA	24	12785	5923	12620	8822	14772	3890	25848
NAT1	23	12818	7220	11281	7711	16000	3131	35351
PGAM1	24	404507	145620	364897	304489	424231	218498	792541
SDC1	24	9667	4583	8722	6626	11537	1934	18711
XRCC1	24	14566	4826	13364	11662	17623	7785	25695
TNNI1	24	7941	2857	7485	6545	8661	2694	16544
NCL	24	122753	59919	113977	84245	146530	45570	273836
POLR2E	23	6599	5645	5110	3930	6495	2832	30571
NDUFV2	24	8241	4581	7106	5494	8908	4217	24990
TNFRSF1A	24	12508	13707	10101	4897	12942	1500	61023
TRIM21	24	13967	12553	10009	8447	11645	6342	59509
EIF2AK2	24	33615	13916	29162	25132	35236	20251	82178
SRM	24	27683	13483	28540	18415	36473	5231	48798
ORM2	24	163260	91016	144482	98462	199014	37609	461265
CSNK2A2	24	4239	2202	3847	2701	4965	1196	9903
ITIH2	24	49612	32718	46146	28627	53544	11380	129896
ITIH1	24	43434	26214	41319	23682	49924	8382	106234
NFKB1	24	13718	5249	11732	10635	13960	8408	25332
NCF2	24	11645	11466	9136	5584	13749	3633	61486
PI3	21	3041	2273	2374	1608	4093	894	10528
TYMP	24	57941	42142	49655	30980	60468	12982	192995
EIF2S2	24	39887	14475	40509	27466	48764	16578	68250
TCN1	24	5585	3775	4628	3367	6511	2678	20579
ANXA7	24	42969	25996	35485	25843	43107	18423	113148
AZU1	24	92369	163305	49013	18883	82358	3100	802763
TPSB2;TPSAB1	24	83333	91511	55791	39663	81217	11133	454768

PG.Genes	n	mean	sd	median	q1	q3	min	max
BTF3	24	12633	3486	12511	10250	14401	5870	20263
RAB3B	24	11320	4510	11061	8279	12236	2780	24807
RAB4A	24	46125	22904	39754	33929	47822	25021	137297
RAB6A	24	23416	9197	21225	18749	24611	14035	54784
MX1	24	74603	47001	51178	44189	107931	22547	191056
PSMB1	24	70025	27477	68969	47952	83988	28517	138635
COX5A	20	2762	1589	2889	1544	3415	309	6258
LMNB1	24	117480	98845	88196	68401	119029	35238	496592
ITGAX	24	12807	1541	12713	11652	13769	10001	15446
GZMH	22	15892	13840	9892	7135	21214	3038	55901
PZP	24	192973	129843	162395	128013	185014	39965	551085
OGN	24	41033	58727	21178	17983	37216	12861	294817
CAST	24	57990	18846	50430	44398	73076	36509	104352
EFNA1	23	7487	5197	6307	3504	11157	1705	21106
C4BPB	23	8954	3207	7915	6981	10576	3485	15547
AGA	24	17039	7845	16857	11057	19495	8301	37364
RASA1	24	26272	8073	27034	20715	31878	8272	40991
PTMS	24	200798	176196	118460	64899	350369	16643	552932
PTN	24	29224	14140	24504	18171	33023	14689	59583
GSTM3	24	76442	85388	55264	15294	101645	7291	349591
ATP6V1B2	24	13759	4195	13897	11021	14991	7370	22739
ATP6V1C1	24	79333	25937	73674	66911	88479	39972	143778
CSRP1	24	199683	518263	47035	34766	66458	19282	2062338
ACO1	24	29896	14796	26593	20393	33194	9343	76808
S1PR1	24	19711	6489	17232	14975	24068	10763	33223
NT5E	24	15722	9130	10572	8751	24809	5016	32787

PG.Genes	n	mean	sd	median	q1	q3	min	max
EPHA1	24	16764	5851	15550	13119	18857	8770	35955
MDK	24	18458	10763	17551	8916	24761	3191	39597
ZP3	24	13485	3156	13032	11935	14414	9263	22842
VDAC1	24	22474	8370	20433	16898	27335	10804	45740
BGN	24	22857	6319	20870	19802	24283	15734	44916
CD9	24	13747	6036	13436	9420	16468	6327	31060
BCKDHB	24	9920	2731	9642	7491	12063	6366	15773
COMT	24	31693	24212	24814	17079	33963	3468	91004
TGM2	24	26963	22324	19179	13840	39380	3118	98272
MMUT	24	33696	8388	31726	28276	35996	24167	59444
OSBP	24	6333	1799	6287	4733	7591	3650	9493
PCMT1	24	20675	5842	19535	15993	23038	13272	37035
FBL	24	122246	49990	114354	90324	156562	26035	251179
GART	24	23948	9180	21640	18025	30492	10179	48487
PAICS	24	64875	39501	57828	39503	75222	14742	159165
GPX3	24	17805	14815	11122	7465	22581	4915	60553
NME2	24	500824	197342	471743	391701	575541	179422	1030031
ENPP1	24	63842	19288	58493	52582	67563	37704	107985
HNRNPA2B1	24	218163	128459	171511	137531	288639	47563	512496
RFX1	20	6667	4145	5968	3527	10161	1048	15140
C2	23	7307	2483	6681	5587	8301	4116	14004
CBL	24	29987	4801	30789	26876	33603	19878	38251
IGFBP4	20	18148	12979	17136	7739	25520	1322	53411
UQCRC2	24	9675	7535	6180	4498	14479	2580	32651
CPN2	24	9437	6129	7630	5486	11905	1975	25083
MMP8	24	16156	19181	11671	9431	14270	5953	103139

PG.Genes	n	mean	sd	median	q1	q3	min	max
FBLN1	24	76521	116233	40426	19914	54378	10147	554042
FBLN1	24	74216	147617	30310	17714	45928	5084	715407
TCEA1	24	18643	8590	19241	11532	25773	4286	35413
SFPQ	24	69662	36918	63814	43266	80534	30060	192174
PPIB	24	139142	82259	109454	85236	174125	35089	336929
S100A1	24	52653	34264	40403	32089	58992	13757	132096
HRC	24	49124	28760	39835	31364	54668	17532	112305
ME2	24	7968	4675	6665	4832	9876	1367	20234
WARS1	24	67376	80729	35903	25264	78572	14412	302369
RPS3	24	52338	23313	49561	40029	57560	17432	124505
GCSH	24	11794	5437	12633	7578	15329	2117	22554
SP100	24	23144	6154	21464	19052	24357	16136	40486
AHCY	24	225891	72816	216426	175023	254479	109503	369899
CFL1	24	672057	146784	640950	556252	770396	465119	1005965
ITPKA	24	54233	16223	47109	44373	58730	38631	98276
PAX7	24	200671	73633	188165	147549	243121	87979	344515
CPT2	24	18157	7526	15994	13184	21660	10528	47076
DTYMK	24	6548	2183	6069	5178	7444	3609	13157
RRM1	24	10456	4334	8941	7543	12935	4419	24155
CMA1	19	3670	2892	3025	1624	5236	619	13040
LAMA2	24	115861	23584	113181	103372	119420	80152	188210
PRTN3	24	39442	79100	24139	13249	36673	1775	404528
MMP11	24	18039	9970	13746	11867	20200	8287	48863
CHM	24	21161	16496	17131	15215	22506	8663	93898
NR2F2	18	1795	1991	1274	844	1631	560	9301
EEF1B2	24	80526	38299	71362	48749	111686	19405	164436

PG.Genes	n	mean	sd	median	q1	q3	min	max
IGFBP5	24	16458	6138	16856	11864	19224	6815	35318
ACP1	24	121560	56998	118084	78059	139245	46603	246206
ACAT1	24	12715	6759	10972	7450	14829	5462	33908
TNC	24	69499	49603	53938	35808	84334	16750	210768
POLR2A	24	142410	23701	140479	126027	153533	107487	197843
CDK2	24	33326	18832	27818	24445	34524	10978	98228
GRK2	24	8170	1287	8464	7021	8977	5918	10491
AZGP1	24	79120	52045	66027	41914	98610	12731	255823
MPST	24	37911	14543	38762	28089	48313	12167	68188
LAMA1	24	92142	22829	87810	75632	106580	57346	141987
RPS12	24	66320	25865	59077	49336	84837	19009	115744
YY1	24	4308	981	4245	3666	4851	2061	6193
DNAJB1	24	126520	43755	113725	98977	140955	56704	220129
DNAJB2	22	2998	1557	2621	1854	3305	1293	7065
ATP5F1A	24	38494	22199	31719	27696	44791	7855	95224
MT3	19	3636	3322	2968	1658	4147	570	14417
PSMA2	24	52611	25253	44877	34652	62660	17043	114901
PSMA3	24	83224	32200	75077	60183	97865	34600	163575
PSMA4	24	75715	29816	69220	53835	88544	29519	145879
S100P	24	76531	42788	73193	43114	85840	19434	191836
COL5A3	24	25454	3352	24455	23446	26506	21191	34530
PTX3	23	3321	1848	2906	1844	4396	924	8604
MSN	24	134924	60910	126349	85568	179054	49965	254850
DDX6	24	22762	8435	22796	16789	27707	9632	39941
U2AF2	24	50387	28382	44982	28950	64963	14756	125399
RPL13	24	12031	5026	11352	8106	14288	5382	23636

PG.Genes	n	mean	sd	median	q1	q3	min	max
CHML	22	6154	4857	4990	2655	7590	1014	19791
IVD	24	47563	14868	46482	37594	54057	23388	94291
S100A4	24	115411	50650	112260	77882	152019	42955	228567
MGAT1	24	15241	5508	14543	11491	16830	5537	28183
HMGB2	24	88553	54893	89374	54079	108572	20708	286065
PTBP1	24	46576	22783	40547	32080	62422	19187	114156
VARS1	24	44856	23341	43333	31842	52079	14135	107295
EEF1G	24	161416	69801	144318	114221	200256	33768	342211
FKBP2	24	52131	32914	46919	27613	58502	10444	141578
MST1	24	48865	31487	38321	25340	62248	19428	149158
ACVR2A	24	1848311	739911	1721938	1303753	2099845	715956	3624390
STOM	24	48101	13873	42540	38665	53814	32750	91735
AK4	24	2593	1088	2420	2003	3297	342	5013
PON1	24	14139	14221	7781	2754	21835	1808	49643
YWHAQ	24	142416	44540	133771	117391	166747	58177	257259
MAPK3	24	42709	17671	40101	33370	47395	18235	107167
CALML3	24	453547	123165	413739	366378	499179	302400	817276
DPP4	24	551313	259881	462178	374411	612464	290598	1210648
RPL10	24	6388	1294	6348	5286	7142	4487	9373
RPA1	24	36047	16347	38299	22536	45620	5674	72455
APEX1	24	191642	84275	172853	129818	223701	89453	415191
DCK	23	5096	2501	4431	3439	5699	2055	10728
CAD	24	36594	7621	38352	31936	42913	23267	49003
CALR	24	152035	85502	133302	90818	206476	40445	358785
MAP4	24	8211	2983	7687	6655	9239	4209	18351
CFP	24	9440	7800	7428	6058	9458	4276	44001

PG.Genes	n	mean	sd	median	q1	q3	min	max
ITPKB	24	177872	34182	176048	159261	201062	120235	256854
PSMA5	24	72185	28188	69336	53081	83917	32773	154389
PSMA5	24	81069	22348	79418	67489	92815	38143	135723
HLA-DMB	22	35276	34248	25297	19607	31571	12122	150572
PSMB4	24	75786	30914	70521	56863	85393	28081	154177
PSMB6	24	64819	27450	69581	38503	83733	20061	110526
GSTM2	24	19238	35166	9101	6637	11950	3844	167357
TMOD1	24	17777	6510	17416	12435	20422	8867	34474
TEAD1	24	7704	2337	7454	6410	8858	4407	13827
MAPK1	24	32374	10506	30642	26705	36717	19794	66534
GCA	24	36360	37225	29883	21228	35974	11306	199004
ERCC5	24	6939	2261	6250	5317	8097	4410	12135
GRN	24	6810	3830	5422	4089	8201	1918	17618
GRN	20	1925	2473	1067	848	1915	553	11324
PTPRM	24	28890	5991	27574	25164	29851	21372	47056
S100A2	23	54153	79455	30807	13941	47150	1169	352074
GTF2E1	24	45132	8875	44749	38623	54173	28066	60544
GTF2E2	24	18075	3475	18252	14936	20016	11972	26897
PCSK1	24	19488	8168	17849	15702	19076	10024	46196
TPP2	24	23717	8358	25283	17763	28315	10183	37986
IMPA1	24	31941	12639	31444	22601	38644	12403	62710
EPHA2	24	12408	3147	11599	10144	13592	8825	21048
EPHA3	24	11292	2081	11795	9536	12680	7132	14586
CRABP2	24	46878	43931	25170	16428	71125	7078	169080
ARID4A	24	46330	11819	43572	38494	49636	30910	76824
CASP1	24	16723	5091	15919	13053	20845	9362	26179

PG.Genes	n	mean	sd	median	q1	q3	min	max
SERPINB3	24	294911	351772	123349	92460	352872	41828	1353017
LMOD1	24	24435	5410	23990	20531	27661	14718	33650
RBMS1	24	10205	4824	9134	7324	11604	4951	23468
SERPINA4	24	17688	10708	13269	10274	21097	5265	40822
EEF1D	24	61072	30754	60919	38639	71999	14846	148735
EEF1D	24	26659	17241	25012	14507	32758	8097	91650
CRABP1	24	15662	9395	12547	9596	18525	6050	43012
MARCKS	24	57073	26903	54534	40411	70140	19489	124341
ALDH4A1	24	24906	13897	20906	18026	28296	12616	79136
PBLD	24	46873	27050	40244	29945	59459	10783	112586
ERP29	24	136630	70261	125748	78146	177566	27586	278006
PRDX6	24	698057	222442	646371	556766	779109	328100	1312782
BLVRB	24	147124	81939	127920	94525	181919	47150	357428
DDT	24	72778	29006	71328	54735	89786	8504	122463
PRDX3	24	43197	26988	33086	25605	58840	4789	107614
ATP5F1D	24	15004	6513	13789	9998	16736	7868	31395
RPL12	24	19537	11642	15336	12775	24606	7675	50422
ECHS1	24	23746	14741	18126	13322	31889	5700	58763
CMPK1	24	51072	25516	53934	34281	69253	10038	113469
PEBP1	24	389301	193131	346123	242732	455955	130543	860950
PDIA3	24	281472	206245	241369	147131	330422	48504	861778
PPP2R1A	24	46236	17899	43930	38593	49873	14865	104161
CDC27	24	139502	58366	131741	101551	167723	61501	314191
NKTR	24	241349	59768	242696	212659	261593	128570	382665
NMT1	24	6339	3315	5350	4260	7697	1392	13505
ADSS2	24	36167	14692	31557	28987	42790	14011	70750

PG.Genes	n	mean	sd	median	q1	q3	min	max
LRPAP1	24	14418	6064	12676	9560	18233	6019	25483
ADSL	24	6611	2480	6418	5099	7566	1669	13545
ADSL	24	15890	3703	16374	13307	17861	6836	22761
CLIP1	24	15996	3065	15067	14414	17405	10520	22052
GSTT1	24	35606	22620	28991	19112	45860	6365	89616
SERPINB1	24	132305	133884	100126	56946	148659	17691	652852
ALDH1B1	24	65627	27617	54551	48792	66781	40415	133700
ALDH3A1	23	9347	9332	5952	4304	8595	2010	35765
POLR2B	24	9725	1204	9724	8884	10643	6463	11515
SDHA	24	140963	40352	132726	110629	163053	89885	228004
CORO1A	24	127101	98507	107589	75474	131805	35423	538345
GDI1	24	40053	21712	36250	29935	44695	16260	121954
S100A7	24	20245	14285	15205	12412	24576	6109	74928
MAT2A	24	36243	13607	33761	26382	42197	17733	79883
PRKAR2B	24	7751	3156	7368	5666	9278	3241	18300
CPS1	24	15615	2468	15466	13585	16430	12168	21768
HIVEP2	24	256471	53432	238774	215131	283935	188214	405834
DNAJA1	24	10608	2819	10348	8921	11557	6147	17970
AKT1	24	76872	27079	67403	60970	89565	34554	157536
UQCRC1	24	7320	5072	5548	4169	7576	2864	21659
HIBADH	24	27327	18209	22412	15563	33568	9775	75654
ATIC	24	56880	19106	55770	48568	63173	10213	101632
HNRNPH3	21	12541	16067	6216	4744	14791	1666	75337
HNRNPH1	24	81072	50637	65250	51002	93838	31494	256866
CASP14	24	12839	9254	10943	9121	13570	5310	53761
SFN	24	37435	25609	33479	24721	39694	5753	124833

PG.Genes	n	mean	sd	median	q1	q3	min	max
STIP1	24	201141	59441	206601	146985	250213	97188	315361
S100A11	24	716137	307504	628056	550699	801007	275231	1471597
CEACAM8	24	14600	11147	10895	7189	16533	2919	50165
PRDX2	24	631291	236006	629796	471664	708738	274473	1249258
CDA	24	17239	32400	8643	6675	11661	1904	163726
DCTD	24	15826	11344	13021	8253	17922	2905	47010
GBP1	24	51980	54428	35792	21962	54141	9486	247475
GBP2	24	23179	16959	17139	13457	24489	7882	75147
RPL9	24	91982	32701	85967	73165	100003	54633	190706
KIF5B	24	15857	6539	14686	11976	19055	5343	29673
CSTF2	24	48051	11637	47918	38856	53335	32530	81044
DUT	24	8344	4722	7649	5149	9437	3178	25988
S100A3	17	4564	6910	3075	2600	3681	508	31029
MCM5	24	44674	13267	43900	34742	54397	23963	69200
GALNS	23	9295	7220	6922	5536	10344	2702	31299
RNASE4	21	4278	6762	1846	1346	3017	676	28909
NTF4	24	22738	10183	19819	15709	29131	9400	46753
SHMT2	24	61933	18276	60424	46548	76529	36192	104975
EPHX2	24	19542	8690	18597	13102	21916	9272	42360
HSPA4	24	98636	37923	100160	70910	125839	33690	189851
GRK5	24	137146	47787	127172	99963	168203	69333	238935
MPI	24	19477	7642	19511	13258	24286	7330	37556
GPC1	24	144977	40097	135713	118166	158646	90895	241676
PFN2	24	15130	10365	12090	8503	16342	2375	39966
PFN2	24	38154	19656	32072	24922	51609	7180	84204
CA8	24	13410	7896	13237	4978	20992	3397	27350

PG.Genes	n	mean	sd	median	q1	q3	min	max
CTNNB1	24	45506	27448	39425	30388	55374	14024	117122
NOS2	24	59866	11821	58389	52329	64448	43304	100348
SERPINB6	24	25048	15441	19454	14909	31547	11281	71337
RPA3	24	17024	9168	18878	8757	21290	3632	38568
RPL22	24	26586	15865	22952	19205	29848	4593	87977
GTF2F1	24	11715	3575	11481	9426	12853	5867	20724
SPR	24	16275	10084	15957	7949	21387	3445	40572
SPRR1A	24	137230	94867	119713	61409	192964	4406	408925
THBS2	24	9411	4124	7850	6828	12491	4537	19449
THBS4	24	6583	2379	6414	4695	7690	3055	13171
HOXD13	24	13079	11098	8920	5566	14371	2576	41139
IDUA	19	10268	34602	2043	1347	3404	783	153061
SAA4	24	6068	4104	4894	2943	8442	888	14271
FBN1	24	371241	54719	359240	337640	417889	265303	480022
PCK1	24	15201	6715	12537	11147	16838	8561	34187
AGL	22	8734	3380	8389	6833	9894	4053	19225
MYH9	24	110282	91478	57258	42873	164487	20067	303194
COPB2	24	310252	119482	308644	249441	405684	56043	491571
ADD2	24	14453	6555	12826	9705	16771	6778	31184
GRK3	24	514487	156960	509439	406600	557501	285195	839837
FUS	24	131989	50044	123497	94926	170311	39024	231123
DEK	24	33928	14743	29114	23829	40027	13761	74147
GLRX	24	24497	11616	20042	17723	29379	11047	58192
HMGCL	24	14965	9499	12002	9161	17533	4414	40307
PSMC2	24	12005	6663	11469	6827	15472	2230	33469
PSMC2	24	19110	10281	18544	12167	26357	5594	51170

PG.Genes	n	mean	sd	median	q1	q3	min	max
CHI3L1	24	13980	14692	9316	5941	15030	3355	67427
ARL2	24	10372	3341	9664	8262	11421	5221	19171
ARL3	24	19322	8211	19438	12311	23889	7236	37813
TRIM23	24	3864	1694	3740	2765	4949	667	7179
MAP2K2	24	7758	1784	8351	6469	8854	4327	10331
ATP6V1E1	24	21209	6144	21157	17722	23562	9500	34202
CPOX	24	68884	31029	66781	43262	87139	28288	154119
RPL4	24	47099	18388	43470	35281	55155	22290	96240
PGM1	24	77243	25472	77003	56300	90069	25519	135025
GNL1	24	5477	2182	4501	4036	6561	2715	10997
SERPINB5	24	18149	10067	15344	12145	19562	7439	49429
SERPINF1	24	35850	43687	24957	19634	28680	7156	219803
DLST	24	19802	9896	16115	12772	28500	7176	38062
GMPR	24	5925	2967	5759	3530	7572	2138	12463
CFHR2	24	8640	5141	7120	5381	10159	2590	20655
SRP14	24	79043	41833	71595	47285	108132	18035	149864
TGFBR2	24	6389	2415	6527	4315	7945	2209	11023
TALDO1	24	367807	137691	364674	273359	409393	195341	801859
SNCA	24	19076	14362	17296	8136	24678	296	57249
COIL	24	24339	16116	20574	16319	27134	9191	91830
HSPA9	24	154227	105025	107019	78643	241466	37394	416655
EIF4A3	24	22255	9407	21728	18765	26335	3963	44077
RPS19	24	123454	50677	104642	94911	132292	75948	293622
RPL3	24	27155	12376	25788	20287	31544	8877	62754
COL15A1	24	51212	26221	42950	39441	52344	22947	154035
ANP32A	24	168776	73712	162156	122599	196833	71818	326033

PG.Genes	n	mean	sd	median	q1	q3	min	max
FEN1	24	37798	14526	39862	24115	46788	13886	64572
CUX1	24	14863	3429	13925	12662	16475	9534	21368
MMP12	24	13025	2950	13000	11241	14962	6215	19686
CAPG	24	109086	49774	107079	71429	135219	38898	257722
CAP2	24	41391	27109	31170	26800	48157	21076	148445
CEACAM6	22	11670	16968	5536	3924	8206	908	74171
CD96	24	17905	4085	17703	15264	20593	9905	24880
TXLNA	24	37942	12661	34867	29970	42927	24136	82780
CCT6A	24	37610	18815	36145	29708	41401	13217	104585
NNMT	24	27350	19188	21074	14338	33697	6283	89288
PSMB10	24	19893	18686	13140	7820	23668	2258	80494
PBX1	20	6439	7610	4163	2103	6079	1105	29414
RPL13A	23	6695	4218	5839	4511	7521	1469	21489
ARL1	24	18960	9023	15084	12870	22302	8768	41073
STAT3	24	11128	4561	9516	7596	15079	5606	23923
USP8	24	20255	4478	19808	16982	24155	11533	27728
PEX19	24	14699	2322	13890	13463	15645	11239	20461
MDH2	24	223810	125492	175148	123640	358937	56693	511735
HADHA	24	23080	13545	21709	12811	26927	8566	62673
EIF2S3	24	32387	11607	31998	23165	39563	10411	53880
OPRK1	24	10202	3119	10216	8468	11322	1483	16442
CETN2	23	9206	6895	6913	5051	10299	2662	32216
ETV6	24	11360	2546	11151	9053	13293	8069	16473
MNDA	24	42457	44011	28934	22511	39065	10840	225238
RGS2	23	32461	29557	24979	18775	38761	4023	148466
PTGDS	24	8657	5371	7815	5066	10587	1312	24165

PG.Genes	n	mean	sd	median	q1	q3	min	max
UBA7	24	129296	51559	117179	94256	157952	64928	247365
NAA10	24	12078	4702	12760	8841	15103	2190	24594
PPP1R2	24	10308	6143	8622	7331	11747	2615	28565
CSK	24	6906	2193	7399	5604	8234	2830	10824
GARS1	24	27521	16519	22401	16509	30406	11337	74996
IARS1	24	97370	58583	72124	52464	135006	25364	222150
MAP3K8	24	9863	6902	8575	5643	10912	2661	33744
EIF1	23	9469	8046	8261	5016	11331	1600	41845
PRKCI	24	72710	24851	66236	57102	86230	34373	132908
ACTR1B	24	128614	48688	120102	99464	143200	56847	279595
TMPO	24	56017	23933	52561	33000	74691	25064	114198
STAT1	24	64910	31372	51947	40818	85439	23837	132757
STAT6	24	24298	4593	23103	21585	26855	16502	34172
STAT5A	24	34640	10515	31374	28804	37916	22291	67806
MTREX	24	36132	12930	32568	26901	41398	18675	65633
AKR1C3	24	33064	40278	16843	12373	41892	3784	179675
EPS15	24	42864	15643	38412	34934	46423	23068	90146
CASP3	24	16955	7515	15450	14294	18284	6144	46201
TEC	24	60439	29329	47227	41240	82974	26216	129850
NCAPD3	24	30541	8541	29798	23376	33627	20589	58400
RBM34	24	211336	67331	194113	167535	243056	107626	371101
LIFR	24	45771	17619	42734	38273	45733	30567	120643
LRPPRC	24	35215	16782	31406	22623	37378	19331	84214
ACAA2	23	45071	31909	38897	22559	52016	11803	147788
RPL35	24	7685	3420	6611	6107	8521	4275	21476
CDKN2C	20	9777	5272	7751	6457	11801	2067	22704

PG.Genes	n	mean	sd	median	q1	q3	min	max
PRCP	24	7274	2897	6324	5289	9164	3396	15313
HTT	24	245667	56802	229304	220751	257540	181593	452608
PAFAH1B1	24	28423	11056	28366	21389	34973	7510	51511
PTGFR	22	34102	36474	26699	19525	37601	10760	191624
MCAM	24	3878	2722	2922	2723	3410	1542	13231
CRAT	24	9345	1853	9064	8262	10518	6315	12866
MATR3	24	11358	9809	8729	6349	12071	3921	52317
ZAP70	24	2594427	496820	2528636	2272386	2844390	1866826	4252946
SYK	24	59578	19205	56015	46565	77029	13862	98655
NAMPT	24	29298	22570	22206	16630	37041	5473	91804
AFM	24	53064	30358	44007	35052	61738	8548	113818
PSMC4	24	490890	213847	471430	321544	656755	205700	1139101
ASPA	24	4946	2618	4160	3508	6026	2116	14525
PPIC	24	7624	4800	7151	5171	9413	410	20969
CBX5	24	11603	8163	8165	5881	14504	2125	30822
RANGAP1	24	93935	36638	86761	78441	105900	36837	182553
RECQL	24	48117	22351	47573	29796	67126	17218	88223
GPR4	23	20273	16696	14114	6717	31022	2566	55735
CRK	24	13082	5577	12639	10237	15971	3084	30831
CRKL	24	12314	6683	10572	8334	14395	5531	38011
BAG6	24	17874	5550	16868	13207	21521	10472	28065
GSTM5	24	29194	46417	14169	10386	25757	6849	230757
NSF	24	13406	6009	11528	9859	16835	4505	24224
CDKN1B	24	22801	18187	15819	10479	27542	7663	71177
RPL27A	24	15872	11691	11036	7048	21437	4019	45205
RPL5	24	20735	7933	21084	12753	27132	5348	34980

PG.Genes	n	mean	sd	median	q1	q3	min	max
RPL21	24	18754	13108	14850	11961	21647	6495	71474
RPS9	24	18116	16153	14091	8425	20103	2615	71780
RPS5	24	11721	6401	10261	7584	13267	5752	33686
RPS10	24	48511	14984	43945	39995	55715	27073	99137
MAP1B	24	20279	7959	18752	14481	21428	10282	40947
GNPDA1	24	42842	15357	39599	34282	54358	12707	81328
IQGAP1	24	103035	29854	94998	79997	114277	66818	171913
HAAO	24	12191	16371	7221	5478	12110	2088	85575
GYG1	24	16474	6455	15711	11611	18904	8198	32827
RABIF	24	8624	10003	5663	4542	7434	1203	49253
PLA2G4A	24	52348	11388	49718	45347	55149	38531	87334
RAP1GAP	23	5485	2039	5162	4092	5886	2594	11028
CAPZA2	24	100220	38388	97703	73169	112415	54431	214671
CAPZB	24	87218	30550	81176	63987	99965	42165	169528
EIF1AX	24	21859	8493	21173	16404	25518	6804	49838
ALDH1A3	24	13661	4907	12810	10069	16317	6472	24650
QARS1	24	16731	4804	16301	12116	19071	10391	28442
HTR5A	20	4353	2109	3701	3213	5062	1640	9570
CDX1	22	10904	6279	8843	7418	12623	3145	26593
RPL29	24	87620	33496	81835	67880	98569	52984	208292
XDH	24	1108781	243314	1016465	942199	1247538	801805	1667302
ATP5PO	24	6081	2173	5622	4495	7883	3324	11097
GRIA4	24	8723	6717	7694	4946	9082	3689	35977
LIMS1	24	14309	11219	11858	7726	16129	4591	58094
PREP	24	43762	13879	42314	34696	52073	15267	72835
ME1	24	48596	17006	43148	35940	58208	26735	82617

PG.Genes	n	mean	sd	median	q1	q3	min	max
IREB2	24	8258	2027	8474	6892	9341	4518	12036
RFX5	24	184558	69780	163146	147064	214438	92986	416910
ARCN1	24	33442	15983	31928	23566	38602	8269	72295
GCLC	24	23304	8622	21885	15762	31666	8227	37422
GCLM	24	6415	4211	5241	3511	8478	1293	16656
PCP4	24	14465	10159	11749	8410	19158	2631	40346
NRIP1	24	26078	6001	25031	22673	29210	16846	40792
PSMD8	24	80583	40443	83131	50994	95256	28013	227954
SERPINB4	22	11738	10533	8894	6645	13570	873	52098
SERPINB10	24	16347	14043	12672	10419	17387	7619	79052
PRRC2A	24	252044	47467	236649	221945	267108	180035	355575
GSS	24	109500	44004	95912	76213	138584	60583	217556
CCT5	24	51763	20093	48107	44304	60115	16056	114328
NES	24	21106	10546	17432	15157	22565	12317	55186
HSPA13	24	63301	18791	61496	51225	75453	34448	104701
IDH2	24	159322	99612	168051	74028	212795	30321	435277
LHX1	24	24736	5707	24868	21883	27163	11734	35422
CCN3	24	8185	3737	7717	5295	9668	4483	19269
ADGRE5	24	245038	57802	242055	217112	276139	132933	422274
POLD2	24	41557	9953	38609	36185	45920	30027	75460
MARCKSL1	24	44582	18276	47948	31801	57189	11045	69838
MAPKAPK2	24	22516	6429	21754	18887	23908	15098	44903
ALDH9A1	24	145044	75832	126838	105486	179449	28881	399957
RPL34	24	5591	3768	4856	3751	6366	1643	20700
RPIA	24	18065	9930	13846	11939	20164	8581	44631
NASP	24	58035	23164	57372	43815	72592	21253	104434

PG.Genes	n	mean	sd	median	q1	q3	min	max
FASN	24	17936	11527	18852	8009	23370	4415	43385
FNTA	24	291315	105895	264960	212196	341605	156178	592092
CCT3	24	30118	17546	26926	21988	31785	7681	88647
TUFM	24	41081	26503	34507	26760	43308	18935	145803
ALDH7A1	24	54172	27026	50237	29354	72256	20084	109663
SRP9	24	40899	20664	37214	26159	58752	14272	80157
UBE2A	24	10180	3976	9830	7298	11168	4907	19469
AARS1	24	13707	4887	14559	10797	17836	5021	21684
AARS1	24	27117	10385	24425	20159	31503	10583	59479
SARS1	24	56557	19623	54845	40487	72756	20465	96538
PPM1F	24	25274	18335	19545	16466	25765	10310	96443
PRIM1	24	6445	3044	5423	4789	6526	3420	16850
PSMB3	24	41174	18798	37555	27665	50487	15929	91714
PSMB2	24	54094	19554	50240	38052	62442	30706	99759
MCM2	24	25568	8022	23957	20140	30360	13475	45763
COMP	24	5162	2597	5004	3441	6002	2573	15192
ACADVL	24	105285	35454	99054	86986	122726	32859	200133
YLPM1	24	91150	23173	88037	74253	99705	62255	140812
ACOT2	24	108041	31801	101514	86407	115472	75568	196796
TMED10	24	23780	7514	22766	19566	27198	10036	45753
RBM25	24	21898	4676	20873	19202	24005	14375	36291
NUMB	24	15376	3887	14523	12543	16954	10647	25751
HINT1	24	34899	18422	32390	23910	44261	9403	79134
FHIT	24	6736	2776	6678	4506	7884	3203	13371
NUP153	24	56140	22049	50851	42614	61138	32918	135910
RANBP2	24	88160	17954	85829	73996	98045	60008	127808

PG.Genes	n	mean	sd	median	q1	q3	min	max
NDUFV1	24	9424	3814	9296	6566	11033	4170	19000
GSK3A	24	66455	23305	70937	46347	85115	27428	102089
SEPHS1	24	23840	11734	20383	15287	30328	8792	48267
SELENOP	24	9713	6599	6981	5989	11897	2736	29342
CAMP	24	27234	67122	11794	6249	19578	1598	338965
GMPS	24	27675	10240	28355	19823	35892	6681	44580
HNMT	24	19254	13002	17178	9363	23791	3136	55511
GNAQ	24	25537	22580	19103	11947	25840	5845	99139
IDH3A	24	4951	1388	5023	3874	5690	2523	8471
MEOX2	19	6550	3305	6204	3593	8608	1628	12283
SULT1A1	24	22563	5146	22920	18904	24724	14026	32747
CRIP1	23	62820	56400	48274	27018	87355	984	232752
MMP14	24	113313	24524	108195	96080	124843	72052	174217
GDI2	24	200120	68258	190031	146665	232486	101838	350756
EMD	24	25187	11651	23212	17322	27370	6218	53195
SERPINB8	24	77575	33569	64651	53343	98803	39573	153980
SERPINB9	24	15257	9191	12541	10686	17231	5841	49852
SERPINH1	24	71678	42910	61150	36957	110661	23663	166925
LHX2	24	79366	60073	66256	39047	92433	15023	243638
PDLIM4	24	26282	38466	15777	14031	21100	8489	198836
ST13	24	160146	53484	166585	121707	195529	52518	238920
ERF	24	14470	5359	13861	11664	15044	5555	33458
VASP	24	22010	10237	19005	15405	23827	11071	53066
METAP2	24	48777	19908	45862	32825	59313	26022	108446
METAP2	23	4765	3248	3846	2923	5278	2411	17767
NUDT2	24	9595	4108	8563	6422	11453	4044	19761

PG.Genes	n	mean	sd	median	q1	q3	min	max
TNFSF10	24	199353	46749	190670	169236	228190	101176	309956
HLCS	24	6794	1500	6778	5782	7897	3930	9361
RASSF2	24	16477	7114	17211	9820	21669	4868	29461
LRBA	23	13448	6007	11767	10414	15871	3116	27139
LRBA	24	500001	143675	518486	379334	613017	204316	731353
BCAM	24	75480	39398	58897	47540	105962	24155	174053
PPT1	24	19026	8770	16093	14411	21069	9636	53587
RPL14	24	29021	7242	29021	23089	33651	17560	49154
ANXA11	24	39747	28572	26240	18472	55100	6834	102227
PAPOLA	24	11415	4132	11191	7275	14695	5615	18957
FXR2	24	118637	80795	97060	64281	131594	39345	348086
RAB5C	24	64373	14014	63872	54625	73474	37676	91570
RAB7A	24	62308	27675	53091	44424	64822	35841	151460
RAB13	24	218225	44732	211250	194759	236302	147156	355727
PDE6C	24	47134	10156	46257	41271	52942	22488	68924
SCNN1G	24	12561	3466	11972	9929	15121	7287	20413
SCNN1D	24	4899	1121	4783	4288	5707	2571	6942
DAP	24	2754786	1232222	2686082	1865794	3520215	982746	5342764
DUSP3	23	9746	10057	7879	5297	9902	2632	53616
SMARCA2	24	44296	6470	44394	40317	47689	30160	57200
TPMT	24	20183	9510	19475	13296	27674	3602	37079
RENBP	23	3670	1919	3088	2389	3947	1708	9900
MECP2	24	42970	22103	35750	32827	40599	28469	118876
IRAK1	24	296562	178827	254926	178423	332485	53801	904164
ALDH5A1	23	3918	2603	3100	2342	4321	1778	13593
HSD17B4	22	9657	9790	7684	4402	9947	738	47185

PG.Genes	n	mean	sd	median	q1	q3	min	max
PSMD7	24	21581	7894	22140	17307	23973	9816	45397
SUOX	24	6916	4980	4932	3447	8629	1711	22632
SGSH	24	6716	2763	6428	4917	8908	3238	14084
STAT5B	24	13834	4163	13329	11998	14947	5893	21751
USP11	24	7146	1519	7002	6280	8431	4124	9525
KCNQ1	24	20595	10199	18513	13778	23227	7872	43642
PLXNA3	24	12774	6995	11145	8208	14847	4566	31948
DYNLT3	21	2204	1260	1870	1563	2736	578	5170
RPS6KA3	24	10155	2965	10085	8020	11638	5466	19339
GUCY2F	24	78014	21594	69773	61822	89415	54611	134896
HDGF	24	145676	73157	129335	102781	163889	54548	309464
LUM	24	89393	137219	39193	30882	46676	15746	597797
PRELP	24	68800	15857	66697	57819	80490	44029	110914
CNN1	24	120968	373799	14516	10751	27319	4595	1695963
CCNH	24	21273	4395	21019	17830	23223	15673	31248
NDUFA8	24	21886	13009	18152	13255	23630	8396	60255
HNRNPA3	24	63857	60525	46555	37641	55923	12403	267827
HNRNPA3	24	121394	81614	104893	75803	128634	23451	408325
HNRNPM	24	44193	35929	31815	23316	48027	15741	171340
KPNA1	24	14234	5260	14447	9568	17592	6290	25677
DGKE	23	8488	7068	5738	3964	10762	2811	31612
POLR2H	24	7582	2943	7995	5748	9606	1155	11968
MAP2K6	24	9893	4263	9365	7185	11338	4136	23397
ARHGDIA	24	121295	30398	119862	99047	137137	80850	206166
ARHGDIB	24	118589	81990	96202	75246	121315	54194	451706
AGFG1	24	10068	4634	9937	6829	12279	4395	21302

PG.Genes	n	mean	sd	median	q1	q3	min	max
HNRNPF	24	19803	16517	15259	10210	20916	7127	70789
GTF2A1	24	15605	9212	13110	10250	21199	357	43571
GTF2A2	24	7571	3445	6640	4602	10458	2887	15225
ZNF140	24	5189	1464	4754	4388	6106	2748	7866
ZNF131	24	7311	1700	7360	6312	8014	4357	11001
ZNF142	24	74868	30859	66107	54069	84881	32571	146145
RBM5	24	22751	6209	22412	19524	24522	13084	44520
RIDA	24	10985	7747	10973	4824	13393	1111	39145
SMS	24	28875	38931	15158	12111	17922	3844	134142
HK3	24	34513	30469	28763	21353	36412	9349	151746
MRPL12	21	8580	7028	5573	4338	10794	1743	28518
STC1	24	66624	40475	55075	35809	88077	21368	165737
NDST1	24	52052	14752	51227	42416	59259	26531	89049
THOP1	24	17045	7119	16495	10150	22075	6496	29680
AKR1C2	24	25626	7306	25226	21258	27313	14305	43300
CAPZA1	24	75834	29290	73178	55943	86244	25652	143099
CRIP2	24	61670	53101	44657	33066	77196	10256	241208
PDX1	22	6393	2085	6366	5082	7776	2621	10236
BLVRA	24	61613	26490	54125	43998	77881	23037	118307
PPP5C	24	26787	9403	24670	21095	32945	14099	50245
ARFIP1	24	45019	21505	40825	31180	58558	12114	94266
NUBP1	24	10417	3648	9394	8119	13309	5107	19348
ACLY	24	11531	7424	9673	6537	15751	1288	28469
METAP1	24	124552	48233	120969	77344	163791	49356	227423
SUCLG1	24	21758	12641	18219	13879	25779	6456	51547
MVD	24	14409	4765	13798	10666	19037	6896	23506

PG.Genes	n	mean	sd	median	q1	q3	min	max
COPB1	24	14984	7673	13977	8779	19875	3903	32251
COPA	24	87657	34472	94881	55439	113541	30411	147891
CTSC	24	8910	4019	8643	4958	12225	2482	15476
LIMK2	24	22397	7393	20932	17010	25061	11538	43019
AP3M2	24	5549	1909	5497	4588	6726	1443	9792
AP2S1	24	14762	5265	13854	11861	17908	4518	25308
TTC3	24	28280	4794	27215	25235	29529	19878	39467
SLC16A1	24	10484	2433	10190	9093	12167	6563	16803
SEC24C	24	15340	5922	13242	10067	21059	7258	25291
SUB1	24	114575	57961	93708	79950	144459	53063	256915
CLNS1A	24	21842	11339	20475	13711	26106	6544	49224
CRISP3	24	17836	24235	11574	7856	15571	5181	126388
BLM	24	24757	5464	24070	21760	28036	12289	39210
RARS1	24	20265	6338	18989	16262	24805	10764	35320
ATXN1	24	13592	4714	14150	10220	16037	5652	24740
YARS1	24	45169	22869	40798	28397	57303	9563	90473
HSPA2	24	407175	124460	372711	333335	469372	249803	759244
RAD23A	24	16328	4160	16634	13509	18264	8808	23466
RAD23B	24	55305	21001	47134	44507	59547	29990	123480
EPHB3	24	11853	2793	11996	10008	12789	7258	20798
EPHB1	24	190964	55965	180446	161327	221057	80827	318008
AK2	24	69485	43054	51529	40900	96911	21668	158796
GAS1	24	334246	194386	315275	199822	404307	120114	1073066
ALDH18A1	24	20253	6636	17900	14879	25736	11981	32994
NAPA	24	42768	19996	39398	31875	48839	16146	117804
ADPRH	24	5469	2297	5539	3828	6610	2394	12927

PG.Genes	n	mean	sd	median	q1	q3	min	max
AIF1	24	12832	7011	11199	8110	15310	5100	37117
EIF5	24	23216	10016	21403	17057	28075	9109	44579
PSMD4	24	9416	3503	8882	6663	11214	4017	17654
DRG2	24	12138	2857	12190	10468	14320	5538	16889
APOC4	24	145649	95368	123571	79112	182315	37986	452103
PLTP	24	15343	5720	14378	11613	19039	7524	29121
CSE1L	24	213121	123129	199708	118307	266028	60118	533803
VCP	24	135682	50976	139667	103418	167929	58414	270181
MFAP1	23	5201	1888	4704	4383	6150	1840	10042
HADHB	24	53515	19154	48933	40976	72018	21300	86336
MANF	24	74588	41535	70770	43537	96310	18804	180430
AFDN	24	5604725	1835478	5305009	4337065	6833659	2851436	10592363
CASP7	24	48803	13552	49163	40282	56483	23658	76813
CASP6	24	75878	30615	78403	59115	96432	19896	137279
ADK	24	47254	20316	44783	31318	52239	18543	98821
LAMB2	24	47806	14759	43795	37637	53649	26732	86398
CDH13	24	14999	3350	14201	13218	16013	9251	26307
FOXG1	24	28623	6278	28491	25478	31839	17893	42770
SNU13	24	43716	23100	39252	32334	49530	10913	114197
NPEPPS	24	87457	29048	84643	64252	104722	49222	150769
HNRNPH2	24	14601	10325	11891	9426	14642	7183	55106
XG	24	8287	7211	4954	3729	11314	465	24778
OXCT1	24	61515	35190	50118	40915	70126	31999	167109
EIF3B	24	31548	13589	27899	23723	37315	8903	62033
NDUFV3	24	12720	4259	12279	10736	14538	1689	22578
MARS1	24	18423	10942	13629	11477	23407	4941	45596

PG.Genes	n	mean	sd	median	q1	q3	min	max
ITGA1	24	36196	5790	36103	32655	39625	24702	48982
ARPP19	24	31876	10207	30248	25435	38873	14120	58470
CMC4	21	3446	2767	2990	1479	4471	466	12260
P2RX3	24	337487	245009	252273	195838	384665	82790	1260135
EIF6	24	50663	18481	44949	38471	56614	21983	100569
CTBP2	24	39718	10619	36284	32798	46154	22280	60118
RP1	24	100673	16639	98468	87755	113197	75330	134129
SLC37A1	24	17311	9098	14380	11515	21721	5439	39658
CFAP298	24	17385	5235	17972	15666	20261	2674	26624
GEMIN4	24	12757	2485	12154	11192	13701	9469	19839
EVC	24	41530	14206	37996	30897	50071	24469	78183
GSDMD	24	8146	5158	7216	3322	12947	1655	17817
EPPK1	24	160164	30301	152469	140599	169146	121100	244158
MTPN	24	48337	13009	46191	38157	56254	32338	78783
NLRP6	24	44337	16810	43592	30135	52700	21511	74870
TAS2R39	24	17073	9795	14486	9932	23338	2228	37205
DEFA1	24	263057	409314	150985	88187	255069	2835	2034920
GNG2	23	8176	4798	6543	4990	10054	3552	22354
LILRA4	22	24129	19299	18926	12210	23117	9084	84274
ZNF445	24	12602	4855	11781	9229	13909	7305	25669
ARPC4	24	159629	38573	152752	130826	168916	117249	272896
ARPC4	24	49319	10871	45533	41578	55960	34911	75658
CD81	21	4242	2747	3667	1993	5624	1153	10181
TPI1	24	810143	356836	714398	545425	943556	409426	1734605
EIF3E	24	21306	9074	18351	16152	24516	7333	40989
PTEN	24	42774	15138	39881	32281	52115	25244	80324

PG.Genes	n	mean	sd	median	q1	q3	min	max
PPP4C	24	10499	3951	10361	7842	12770	5027	20819
GABARAPL2	24	18336	3769	17710	15833	20920	12447	27934
MYL6	24	21532	6572	22258	18258	25145	5541	33040
PFN3	24	8283	2572	8247	6933	9347	3643	15479
ACTB	24	863604	286647	900918	688423	1059435	284440	1361782
EIF4A1	24	137997	50056	148332	97601	161916	36804	245304
RPS20	24	36208	18033	33902	24967	43872	11178	77957
PRPS1	24	8551	3636	7986	5883	9688	3970	17581
PSMA6	24	96863	37372	91377	70536	114372	39092	187105
S100A10	24	80771	34278	74023	59220	104926	23586	155118
CDC42	24	110506	38217	103273	94353	116098	78397	272913
DSTN	24	134540	154554	98639	79537	121141	40510	799182
GMFB	24	591536	123503	601317	500538	655884	341377	841425
RAB5B	24	84364	31908	74130	63448	93710	46525	167459
RAB10	24	22847	5801	21866	18913	25356	12538	36820
UBE2M	24	33447	12011	38158	24544	39802	13895	53069
UBE2K	24	53003	16862	50413	40418	67709	23046	84073
UBE2N	24	199332	82988	183071	137740	229025	98938	423873
RAB14	24	10241	5392	9419	7471	10511	3681	29458
ACTR3	24	103614	28739	97098	80140	111517	71075	200139
ACTR2	24	43750	10377	41834	38451	46152	31046	79608
ACTR1A	24	11748	6436	11246	6955	13786	2908	32467
ABCE1	24	15401	6020	14865	11513	19540	5047	27678
RAP1B	24	29872	14587	26103	21113	31582	15287	82415
RPS3A	24	24628	12244	21323	15408	32426	8302	65284
PSME3	24	87081	43688	78581	49471	113843	23346	165060

PG.Genes	n	mean	sd	median	q1	q3	min	max
RPL15	19	5424	4275	4335	2684	6102	1352	17168
MAGOH	24	5388	3740	4039	3374	5979	2153	18116
RPL27	24	25583	9481	21375	19406	29281	15333	52733
ATP6V0D1	24	4712	2248	4067	3145	5179	2477	10857
PCBD1	24	38450	22229	29755	23577	42700	17655	101610
RPL37A	24	44830	27099	45279	27111	59646	1840	107573
RHOA	24	16767	5096	17080	12477	19046	7296	27661
NCALD	24	6728	3749	5286	3864	8268	2735	17578
HSPE1	24	208913	112613	185790	128529	273052	64971	459694
LYZ	24	111102	153857	74686	49118	100513	10352	788886
ST8SIA6	24	22604	7782	21001	17141	28444	9724	39453
VBP1	24	17136	4887	17327	13530	20949	7456	24790
B2M	24	23779	14392	20013	16352	25983	8371	77509
NPC2	24	68366	36755	68815	36127	93397	13970	163227
COPZ1	24	32747	10203	31408	27026	37041	14229	57911
UFM1	24	72420	34834	64690	47934	82570	11854	170054
DCAF7	24	41631	41538	31400	21455	43519	14227	223967
WDR5	24	14956	6491	12851	11099	17220	6336	33171
AP1S1	24	45150	14822	45013	35588	56812	15989	67649
NUTF2	24	50825	20392	47315	35566	67098	20620	92302
HNRNPK	24	268593	121672	249716	197153	328562	91260	611162
YWHAG	24	57864	15063	56563	45604	65047	36241	93989
TIMM10	24	7754	2708	6807	5677	9482	4122	12942
RPS7	24	29558	16922	25234	18816	36272	8027	81422
PPP1CB	24	22449	7854	21086	17103	27360	8080	39985
PSMC1	24	8759	4351	8494	6436	9436	3804	26436

PG.Genes	n	mean	sd	median	q1	q3	min	max
PSMC5	24	14863	6988	13963	10492	18582	5031	36967
RPS8	24	39548	19912	31217	26635	47386	20965	98128
RPS15A	23	21091	16479	15112	12708	23290	7176	76570
RPS16	24	17883	11170	14433	11595	18700	8456	54124
UBE2G1	24	5021	2540	5417	3182	6563	1083	10466
UBE2H	24	10612	4904	9251	7787	11496	3973	27377
YWHAE	24	67741	28960	62142	44321	77808	27981	124845
RPS14	24	37558	20149	31060	23066	47396	13507	83373
RPS23	24	15041	11592	10682	7059	18085	4091	46722
RPS18	24	27279	16551	22340	19640	27048	11096	89631
RPS13	24	18983	14975	14167	11609	19537	7660	72228
RPS11	24	26545	20872	20826	14706	27470	8590	105773
SNRPE	24	35572	28283	28043	19991	38500	12770	129550
SNRPF	24	19681	8392	17332	12868	24575	10422	41971
LSM3	24	73399	24006	73937	59993	91753	33078	116472
LSM6	24	43259	15197	43548	33007	52585	19222	73690
SNRPD1	24	31173	13114	28443	21269	37696	12557	60881
SNRPD2	24	35910	20707	30531	20496	42957	15851	91708
TMSB4X	24	144285	53885	143861	115107	177659	35554	247338
ARF6	24	7117	3530	6695	4526	9559	1986	15253
PSMC6	24	9656	4216	9665	6491	12280	2953	20947
RPL7A	24	16008	9160	13937	9788	20355	3964	46920
POLR2G	22	4205	2478	3314	2387	5862	518	9068
ETF1	24	18130	6635	18106	13905	22114	4540	30531
RPS4X	24	141532	75683	116461	89710	182418	63066	377317
PPP2CB	24	6750	3497	5725	4921	6806	1464	14831

PG.Genes	n	mean	sd	median	q1	q3	min	max
ACTA2	24	1689377	553014	1591653	1333881	1993578	943757	3298919
RPL23A	24	215444	174078	157932	116549	224128	65465	821807
RPS6	24	36247	18864	30575	23133	44851	12799	77312
H4C1	24	68465	181225	18098	14105	23856	5830	852505
RAB1A	24	77806	44606	71643	56822	81516	37630	264148
RAN	24	285785	169876	261564	178829	338635	57796	750319
RPL23	24	37184	16271	32718	27517	43058	14758	93902
RAP1A	24	5953	4294	4513	3528	7430	1860	22866
UBE2D2	24	43253	13292	41468	32518	48818	25796	81917
RPS15	24	14669	21228	9069	6575	11244	2009	105201
RPS24	24	8173	4647	7226	5249	9439	2782	21746
RPS25	24	41481	25335	35764	24314	53922	17708	129800
RPS26	24	13716	18102	6077	3993	14134	1601	79177
RPS28	24	56704	23768	49778	40456	73984	24015	106767
GNB1	24	47925	11833	46529	40790	52198	23156	80586
POLR2L	22	5866	2791	4702	3727	7601	2811	11472
RBX1	24	32042	5977	31476	28957	34441	18803	46987
GNB2	24	17681	8262	17705	11423	21818	5445	36172
RPL30	24	19326	8876	17746	12241	22899	7038	47610
RPL10A	24	12447	7988	10544	7780	13889	3801	37544
RPL32	24	33125	14235	31657	28852	39121	5047	75375
RPL8	23	8021	5328	6154	4388	11105	1903	19204
PPIA	24	852509	241083	804955	684857	966653	537019	1633550
PPIA	24	824017	341612	798688	561091	989141	309094	1525532
FKBP1A	24	145011	57991	132400	107973	166547	61387	338741
GRB2	24	25211	8004	24229	20549	28578	11174	42729

PG.Genes	n	mean	sd	median	q1	q3	min	max
AP2B1	24	235172	63976	221335	185344	263569	149697	399026
PPP3R1	24	12965	4176	12809	10216	14648	6053	23255
YWHAZ	24	585615	195582	560106	431297	686824	309320	1077834
UBE2B	23	5711	1915	5579	4158	7281	2645	9121
SUMO1	24	14297	7551	12121	10030	18476	3963	32005
DYNLL1	24	22703	9286	21523	16805	25900	9838	49871
DYNLT1	23	4328	1632	4334	3134	5239	1202	6857
RPL38	24	23806	17061	18535	10614	32783	7855	65868
SKP1	24	22172	4635	22132	19560	25821	11881	29535
GNG3	24	13458	9581	10803	8717	15386	2479	47359
RPS21	24	26822	8977	23630	20613	33800	14950	44897
RACK1	24	41869	36542	25904	12012	66970	3552	112676
ACTG1	15	3170	4126	1924	1320	2506	522	16732
UBE2I	24	117878	49941	112601	79327	143404	41451	236507
SELENOW	20	3454	2245	3132	1762	4040	1360	10903
TMSB10	24	114147	39193	105915	92934	123845	62147	244721
PPP2CA	24	42730	11613	42034	36890	48259	20224	76214
YBX1	24	118212	43946	103733	90951	144109	62770	205518
CSNK2B	24	14936	17804	12336	6919	15085	1988	94694
TPM4	24	101419	46465	95213	67934	115939	33070	217334
TPM4	24	598610	462463	463778	345997	618204	248492	2241016
EEF1A1	24	5265366	1522881	5479412	4611306	5987915	1533226	7878420
FKBP1B	24	7802	4566	6320	5146	8213	3717	21731
ACTA1	23	8826	3124	8235	6929	10275	3826	17307
TUBB4B	24	153468	86807	137108	93486	212121	21987	308329
CSNK2A1	24	19834	7514	18672	13789	23467	8009	38025

PG.Genes	n	mean	sd	median	q1	q3	min	max
PAFAH1B2	24	243263	79364	242348	194434	284542	124234	477967
PSPH	24	15680	13336	11301	8855	15386	3944	49563
RPP38	24	33765	5943	33842	31295	36241	19088	48623
PIP4K2B	24	73959	19910	70453	63919	81979	35400	117142
CCT2	24	146990	65164	142379	122338	168645	36287	341474
RAE1	24	16083	7874	15610	9647	18151	5760	39554
DENND2B	24	10074	2757	9397	8251	12146	6330	17033
PRKDC	17	3904	2859	2578	2151	5853	1274	11948
ADAM17	24	28567	15008	22498	20335	30713	16264	84930
BLOC1S1	24	5466	1041	5498	4831	6027	3250	7765
GPLD1	24	35947	13583	33581	26058	47833	12537	59996
LCN2	24	371735	551870	166765	116314	365711	15099	2611203
NUCB2	24	34822	23139	24430	18167	48982	11773	91694
S100A12	24	53783	94984	35876	19746	48533	1719	484985
BASP1	24	55267	36777	42852	33844	67473	13968	182795
MRPS36	24	4729	2028	4143	3657	5750	1516	9336
MRPS15	24	11468	4797	11920	7560	15583	3371	20022
MRPS21	24	44208	19198	39359	30828	49171	24330	114998
MRPS34	24	54787	21281	54969	39722	63338	13412	119638
HMGN5	24	24433	9077	22284	16825	30744	10825	44142
SARNP	24	45534	19179	41632	33315	47416	20652	95004
RBP5	19	4675	3987	3326	2052	5316	1104	13527
LACTB	24	66914	27477	59833	48506	74989	33390	126825
COG7	24	78042	14308	80804	67508	87015	49637	110442
RPL24	24	93991	26363	90580	78444	111987	53356	142998
TXNL4A	24	8802	2524	7886	7540	10100	5296	14695

PG.Genes	n	mean	sd	median	q1	q3	min	max
CBX1	24	50873	27184	40634	35786	56534	25756	136121
SMAD3	24	19394	12626	15334	12564	23118	7756	61129
ARF5	24	24473	10217	25030	17135	31154	8062	45769
ERH	24	56589	28139	53281	35462	69251	23690	133037
RHOG	24	10988	4983	9401	7179	13363	5379	22020
RPL19	24	9651	15062	6037	3611	9502	906	77305
SRSF3	24	49161	24892	44439	30329	57642	25130	120657
MXRA7	24	11010	9711	8371	7017	10845	3218	51433
FOXK1	24	14642	3182	14406	12615	16282	9137	21067
CCZ1B;CCZ1	24	21080	4697	21169	17389	24020	13117	29815
TMPRSS15	24	54081	56080	34704	26215	59469	13340	280751
MUC5AC	24	54226	22895	47985	40649	56228	30575	109251
FBLN2	24	18835	4056	18481	16046	21516	9849	26931
VLDLR	24	60909	23904	56021	47242	66696	26805	124654
HSPG2	24	95977	71161	69256	54252	110766	36133	345551
RBM3	24	11788	6139	9811	8583	13347	2670	29668
CYCS	24	79941	50738	64977	39663	124598	25217	188419
TFAM	24	7584	2757	7179	5143	9469	4160	14025
PITPNA	24	22022	7864	20826	16879	24043	9562	40847
HDLBP	24	8735	4165	6977	6233	9081	4032	21145
HDLBP	24	19332	5827	17466	15554	23063	10528	34232
GTF2B	24	15133	4664	13982	12351	16013	9128	29498
CDK6	24	10091	4449	9069	7393	11259	4010	23972
PURA	24	9190	6790	7691	5513	10652	1002	33648
CLTC	24	42359	22666	38668	23750	58957	14971	91851
HSF1	24	26906	23529	19780	9878	36786	5824	92572

PG.Genes	n	mean	sd	median	q1	q3	min	max
FKBP3	24	60648	26746	58896	43548	79983	10604	113387
REEP5	24	6410	2813	6333	4798	7537	1703	16762
SORD	24	69829	68519	53223	31225	72458	8586	283832
HNRNPU	24	185637	115276	162662	114794	206864	42929	471709
SPTBN1	24	21256	12305	19611	12506	26866	6628	62533
TIAL1	15	2295	1781	1770	1320	2464	427	7339
INSM1	24	26560	9489	24447	21343	28815	11783	51636
SET	24	128488	50757	116193	95856	176197	55575	233024
SRSF2	24	20118	12342	15374	11581	24796	6069	53673
CTBS	24	9515	4598	8106	6060	12226	3768	20849
FABP5	24	62455	70722	41869	29064	63655	9324	345877
ANK2	24	51459	11400	45939	43954	56489	36845	83171
CAP1	24	168809	59124	157006	131657	180033	95081	333136
PFKP	24	198948	107466	163009	140291	247153	61105	503026
EWSR1	24	65644	17884	62722	55892	78526	34075	117857
OCRL	24	20802	4876	20273	18016	21828	13313	38758
TAGLN	24	548391	1604459	100829	62761	157802	40552	7496279
OGDH	24	213126	65337	201699	167615	272920	100392	334698
COX6A2	24	14877	8399	14563	10012	18307	2391	47100
ALDH6A1	24	15430	8640	12776	8793	19657	5197	38742
BDH1	24	26751	17170	23515	18005	30141	6847	88843
DSG1	24	50579	17251	47719	38798	57441	26072	102295
DSC2	24	40526	9129	39969	33042	45676	26881	64632
H1-1	18	10959	17333	4232	2935	11442	1245	76139
RPL18A	24	4727	2742	3951	2696	5425	2069	13005
GHRHR	23	8512	4962	7273	5857	9697	949	19527

PG.Genes	n	mean	sd	median	q1	q3	min	max
GCNT1	24	81840	40570	75953	53559	102618	23788	177336
MAP2K1	24	19437	5620	19453	16759	21305	6639	34953
FKBP4	24	68663	37779	59595	44168	84142	13885	148165
NUCB1	24	11475	7623	10076	6592	14320	3316	39071
RPL6	24	22440	10001	21581	13367	26838	11078	44853
TOP2B	24	31884	7816	30123	26618	35276	21982	55096
CREB5	24	483804	150050	457749	384476	549512	232200	862821
AKAP12	24	24105	24649	16473	11602	23272	7967	108410
DST	24	72706	15776	68841	60760	79523	51898	107235
CAV1	24	116736	70160	99122	73785	162102	17845	335902
TGFBR3	24	13459	6241	11889	10011	14095	7625	34623
TNFAIP2	24	41573	14962	37455	32549	46442	15431	80839
LMNB2	24	11763	5099	10673	8735	13892	4943	22598
PTS	22	16583	16287	11189	7908	21091	4738	80658
CFHR1	24	14331	11077	11307	7993	14946	1981	39128
EVX2	22	18277	16881	13615	5667	24698	726	63328
GBE1	24	19572	14502	16970	9882	23967	3890	70771
NOTCH2	24	208049	29235	203034	188523	228266	159403	278001
HGFAC	24	17590	5846	16326	14123	21173	8519	36827
SSBP1	24	17089	12274	14562	8989	19798	4179	55680
YWHAH	24	123630	48513	115743	92429	132713	61686	289940
PLP2	16	4162	3155	3024	1623	5405	1123	13147
CSTF1	24	18888	5053	19406	15893	21685	10307	29409
PTPN12	24	393327	54996	386246	360500	420370	297334	501982
CLC	24	22814	25922	14993	8977	25114	3820	132671
SRSF11	24	174483	61241	164986	137011	201143	59266	376578

PG.Genes	n	mean	sd	median	q1	q3	min	max
EEF1A2	24	19345	8584	17729	13805	24777	5874	45228
CALD1	24	102637	210128	44295	28193	57978	13765	924545
CALD1	24	16230	10719	13640	8969	19890	3004	43935
EML5	24	193182	30830	188898	172626	202848	145604	285798
PTPN11	24	17772	10776	14742	10103	21145	8165	44023
PPP2R3A	24	86685	16730	85245	75969	96675	53030	135152
PPAT	24	15994	9767	12972	8640	20992	4702	38076
EXOSC9	24	16964	7199	15514	13274	19334	9626	44824
EXOSC9	18	4662	2089	3798	3319	5989	1876	9191
PSME1	24	311508	163103	281420	202896	402706	105389	815184
GABPA	24	19506	5793	19022	15768	22932	11582	34163
RING1	24	7154	2533	7310	5147	8640	2631	12053
FMOD	24	16043	8235	13699	10594	19840	5186	36013
PRDX1	24	546719	169705	523733	417666	660550	180952	906020
RPL18	24	8352	5428	7099	4968	10071	1044	26287
C1QBP	24	30585	17196	26633	20047	38766	6716	80524
CKAP4	24	49456	15158	44883	37850	59313	28301	83101
TJP1	24	53599	22223	45683	39347	57813	33018	122873
TCHH	24	175647	48756	160362	140955	191251	123288	331736
TFF3	24	150715	190874	79162	29506	191855	4410	860511
KHDRBS1	24	29876	16784	27115	19101	36889	10688	82395
SOS1	24	47251	9204	45660	41667	51226	31612	67830
LRP1	24	102002	23973	98127	85172	105651	69622	154585
SRSF1	24	63051	37506	49452	40967	79537	21775	168261
ARHGAP1	24	24078	19819	20732	14963	28786	3286	101764
SRSF4	24	13942	6831	11421	9297	15153	6567	38417

PG.Genes	n	mean	sd	median	q1	q3	min	max
TGM3	24	27614	5889	27346	23208	29878	19462	42225
DHX9	24	42019	21203	39684	29536	46463	12083	101093
LGALS3BP	24	41368	20914	34759	29672	56688	6844	95353
EHHADH	24	6528	1867	6380	5559	8060	2949	10055
PPID	24	35074	16202	34021	22337	40930	15965	88735
SSRP1	24	14702	4990	13499	11144	17846	6555	26390
SLFN5	24	29835	8916	28775	22814	33338	18324	53228
VAC14	24	68484	13911	69217	56605	76495	45427	105630
ZNF616	24	17902	6040	17273	13488	19126	9339	32054
RBBP4	24	38243	21116	32633	24935	45419	10372	84796
NCBP1	24	14351	5408	12802	11068	17422	6645	26296
AHNAK	24	156844	184676	101913	65833	138877	46007	742488
EGFEM1P	24	51335	23518	52895	41561	58193	7732	117590
ABHD18	24	59330	23421	53443	40863	74091	26594	107840
LRRC74A	24	225396	63990	220493	178852	288524	90328	306990
HSPA14	24	68292	18527	64335	57765	74635	40051	113672
SCRN3	24	4033	639	3997	3552	4589	2986	4953
CGNL1	24	499905	102884	475360	446831	518424	383669	760627
CCDC173	24	53306	11244	50742	48160	57657	37063	92213
FABP9	24	16789	8987	13582	11983	17860	7976	43650
NEXN	24	40422	9200	40529	36312	46074	18087	53648
GALNT2	24	11770	2452	11273	10275	12803	6350	16769
GALNT1	24	40743	24516	33664	24519	47648	15508	129654
AP1B1	24	30438	8993	29938	25024	32554	15202	49447
CPSF1	24	56575	12910	53636	49777	66159	34972	84662
HMGXB3	24	67670	14207	66013	59156	73054	42836	110849

PG.Genes	n	mean	sd	median	q1	q3	min	max
WASHC5	24	7810	2824	7457	6147	8241	4268	18408
NUP160	24	27607	7526	25774	23566	30134	18623	53501
SCAP	24	22828	8214	22108	18855	25671	9625	46813
SREBF2	24	3344	1158	3363	2594	3897	1485	6333
ARHGEF5	24	53060	19950	48132	44691	51835	34231	132445
GTF3C1	24	1524581	326887	1467522	1350698	1669674	998037	2315969
HYAL1	24	21586	12576	19592	14761	23006	9184	62674
SMARCB1	24	17954	10285	15054	12133	18997	8888	50174
FSTL1	24	6528	4572	5622	2784	8186	1088	19175
SF3A3	24	13730	6286	12837	8965	16927	3104	27890
DPYD	24	44437	24177	38393	30227	46624	16379	115139
ILF2	24	62388	30806	56614	38821	71082	27004	129384
ILF3	24	55811	27248	54389	30362	62099	23629	115041
LMAN2	24	35444	12043	31221	28203	39125	23367	75655
IRAG2	24	35297	8001	33542	30161	42060	20449	50138
EPS8	24	6737	4514	4713	3949	8439	2561	19598
FOXF1	24	20972	9953	19310	16277	24510	2039	40128
FOXD4	24	42522	28479	33467	27041	45760	6632	113708
ANK3	24	75830	9618	73703	68955	78436	62921	103967
ANK3	24	156209	91680	125837	114071	143240	96550	463005
TAF10	24	10376	4530	9916	7496	12221	1897	24475
MYO1E	24	163491	28042	155264	147794	173194	128379	256189
PPP1R8	24	6083	2144	5794	4083	7236	3510	9978
PTP4A2	24	9650	3596	9697	6930	12084	3084	17418
ABR	24	14548	2904	13883	13187	17044	8454	19326
CSTF3	24	5407	1972	5263	4309	6202	2606	11191

PG.Genes	n	mean	sd	median	q1	q3	min	max
ECH1	24	23366	13702	16427	14543	30438	8421	49837
STRN3	24	18611	35483	10550	8757	13204	6760	183864
FLII	24	95303	24353	94249	80626	103018	39537	169689
LCP2	24	35965	18849	30275	22947	40768	17441	97999
USP4	24	31003	7859	30205	25704	36083	18668	49581
DUSP4	24	108581	39268	113645	82522	135033	44313	181749
KLF10	24	16826	5422	15500	13649	18114	9406	32203
IK	23	8738	10175	4554	3955	7049	2709	46079
EIF2B5	24	18866	7760	16605	13755	20441	9379	37010
TARDBP	24	11859	7432	10144	7229	13548	3533	37192
HNRNPA0	24	15639	13799	12231	5422	21724	1849	57786
PAK1	24	15634	3778	14526	12823	18351	9854	23258
AIMP2	23	4763	3356	4069	1687	6513	874	11753
FADD	23	5601	2973	4697	3492	6357	2361	12451
PRDX4	24	14426	8431	10576	8470	20149	3201	33754
PAK2	24	18528	8887	17007	11592	20406	8321	41030
CBX3	24	30057	12071	25428	22408	37253	13668	66412
STK3	24	33902	10786	29843	27542	38378	20987	68144
PSMD2	24	104439	62343	87713	66577	134073	26779	334211
MMRN1	24	29292	4908	28461	25132	33101	22666	39218
DDX10	24	883135	261377	878452	765150	954181	179970	1427013
DNAJC3	24	19990	5619	18615	16525	21754	11432	33119
NME3	24	10730	4888	11524	6894	14185	2356	18845
SRSF9	24	107687	70173	72445	58734	146382	24278	267661
SRSF6	24	70624	33002	60783	51991	84822	29301	161312
TRIM28	24	21883	20362	18189	8237	26163	4552	86288

PG.Genes	n	mean	sd	median	q1	q3	min	max
G3BP1	24	24995	11877	23920	15271	33463	7451	53418
NMI	24	35535	15273	35407	26579	40581	14434	87664
SLAMF1	24	31725	4997	30580	27848	34214	24985	41782
EIF3I	24	20067	9563	16820	14266	24473	5918	41508
ILK	24	34666	38272	26841	16090	35839	13519	199420
MSLN	24	17998	4629	16964	14699	21487	10865	26929
NNT	24	35553	8299	33162	30452	41107	21649	57198
SNTB2	24	42912	19137	35676	31697	44929	20621	109242
PPIG	20	11061	18592	4180	1415	9170	737	75856
TCOF1	17	2047	1119	1814	1358	2769	391	4536
TCOF1	24	50259	13757	48847	42845	56615	21657	81421
SF3B2	24	90195	35184	84774	60258	114731	45118	170319
GOLGA4	24	90478	11980	88055	82099	94946	76704	129217
PDAP1	24	28691	7165	29254	23840	33891	14805	40342
ADAM9	24	263996	102873	255642	187856	323726	114148	566523
LSAMP	24	23632	11888	21898	15059	27655	9322	59895
FKBP5	24	53705	25608	41299	35971	60223	30724	116089
MYO9B	24	19139	3064	19161	16962	20109	14987	28267
ROCK1	24	13212	2286	13193	11759	13742	10200	21216
IL18R1	24	21463	13374	18980	15294	22609	8974	75535
SMAD4	24	7126	2798	6352	5300	8513	3188	14827
SNAPC2	24	15120	10834	12005	8044	16576	2492	39740
TUBB3	24	7357	4099	6391	4675	9165	1959	21128
PPP1R1A	24	27495	9263	27301	22665	31054	8835	52384
PRPF4B	24	119833	23359	114392	103611	136767	87359	195723
PIN1	24	11720	7139	10237	6655	13477	2629	29173

PG.Genes	n	mean	sd	median	q1	q3	min	max
EIF4EBP1	23	13249	6704	11115	9313	15475	6277	37043
HDAC1	24	265801	111754	250220	193242	303860	82406	552761
DCTN2	24	24463	9981	23524	18119	27674	7570	48611
ITPK1	23	5061	2664	4819	2607	6625	1675	10236
SNW1	24	12058	2783	10951	10258	13494	8159	20589
IQGAP2	24	44797	14582	40415	35003	54015	23159	75950
GPR50	24	65436	10686	64136	62296	70587	40464	87135
STIM1	24	52902	17664	45942	39863	58905	32130	92173
TRA2A	24	12713	4087	11569	9891	15073	7191	23715
SNX1	24	16087	6033	15999	12760	19241	6798	33296
KRR1	24	23902	11191	20280	16163	30353	4385	54405
CUL1	24	11847	4886	10664	9348	14032	5393	27025
CUL2	24	41136	18806	36953	25955	51865	13959	79838
CUL4A	24	45134	11799	43976	37778	51959	25844	70853
GFUS	24	27991	13955	28208	15820	35887	8383	53635
RAB32	24	32235	12892	29405	21301	42742	12606	59502
FHL3	19	4045	1082	4200	3623	4612	748	5970
AAMP	24	744606	407749	638353	433030	898311	287201	1569290
ALCAM	24	63241	16946	57708	52080	70888	45844	112803
LAMB3	24	46557	12438	43847	39660	52746	26758	78678
THOC5	24	13856	5931	12240	9715	16999	5905	25798
APOF	24	23015	10877	21757	16522	26617	7915	62612
AUH	24	8234	2413	7987	6760	9423	4086	14677
DDX39B	24	79402	24942	77076	60912	95320	30965	138234
BLMH	24	34884	12388	32544	26357	37167	22475	76568
EXOSC2	24	18479	5485	17877	13735	22201	10513	30013

PG.Genes	n	mean	sd	median	q1	q3	min	max
SNTB1	23	3019	1550	2421	2013	3194	1127	6887
TUBB2A	24	17889	9879	16504	11633	23851	2390	42410
BYSL	24	48290	19528	41300	34110	54932	18365	87133
RAPGEF1	24	48123	25828	41152	30338	54068	7417	106645
CAPS	24	163855	118337	158304	59795	231366	10458	406458
CBFB	24	10376	4341	10132	6618	13820	3929	19473
IL16	24	15797	3707	15478	13271	18000	9921	22953
CAMK1	24	7647	4792	7120	5842	8333	1311	25083
COTL1	24	62554	25956	58725	43439	72350	27920	125134
CYLC2	24	47946	17979	42962	36998	51153	31232	118810
HNRNPD	23	4905	2730	4492	3200	6224	707	14129
SCARB2	22	2324	1050	1950	1561	3053	697	4513
IL18	24	76664	36392	66760	53948	90481	17623	181021
DPYS	24	70480	24880	65467	52528	76282	39780	143925
DAG1	24	20698	9177	17938	14712	25678	8174	47132
VEZF1	23	6642	2360	6187	5335	7723	2548	12363
DSG2	24	149351	46979	145810	115095	170668	67105	234405
SEPTIN6	24	6423	2260	5995	5091	7797	2588	13098
MORC3	24	69073	8925	68173	63383	75644	53710	92019
SAFB2	24	14293	4619	14326	11342	15807	7465	24006
EIF3A	24	159616	68792	140900	122200	185463	62569	303366
MLEC	23	7125	4013	6257	4351	8563	1815	17792
TTLL12	24	26753	14202	19585	15684	38959	11089	63472
DOC2B	24	851470	295673	764350	623485	974271	525028	1698845
DOCK1	24	12368	3514	11516	10303	13189	8712	25599
WRN	24	109591	41670	94147	81762	127993	53223	197746

PG.Genes	n	mean	sd	median	q1	q3	min	max
FHL2	22	2987	2610	1842	1320	3055	927	9865
CRMP1	24	63766	19318	58807	51377	67848	36336	105632
DPYSL3	24	43008	53853	28794	16454	37592	10391	240368
MRPL58	24	42531	18351	37633	28446	53655	17848	82630
DYNC1H1	24	102239	37491	93791	69421	133673	35580	170845
NPAT	24	478670	82552	465325	450490	531966	317211	716701
EIF2B1	24	94491	51805	87194	52645	108720	26510	211334
ADGRE1	24	67025	30077	59480	44702	78786	33977	145930
CTTN	22	11596	8750	8848	6734	10940	3858	38083
FLOT2	24	43786	16989	39824	33112	49868	19068	86553
TRIM25	24	13677	6955	11508	8781	17287	4646	30631
PTK2B	24	92763	27709	89906	71641	106004	58595	182275
FGL2	24	9777	2093	9555	8498	10991	5368	15970
FAM50A	24	6099	3024	5040	4413	6888	1821	16214
FRG1	24	17459	6636	15930	13395	21014	5330	31776
GAMT	24	33901	22627	25960	18644	45406	6176	109722
LRRC32	24	21901	11704	19195	14434	25591	7168	59485
GK2	24	4742	1628	4557	3440	5704	2467	8716
PDE3A	24	34691	6556	33562	30399	37937	25557	53136
SLBP	22	12307	7147	11115	8209	13966	1185	32527
RBM39	24	154709	90204	127403	105602	174493	46134	459755
WFDC2	23	140550	140509	102143	47876	171887	646	523462
SPARCL1	24	93146	58678	87932	52848	128339	9244	212398
HABP2	24	22585	8650	21922	16813	26146	9762	47229
HNF4G	24	10756	3759	9072	8134	12209	6655	19366
PDIA5	24	53555	36229	42188	31815	57244	20705	180676

PG.Genes	n	mean	sd	median	q1	q3	min	max
PRPSAP1	24	5703	2417	5132	4336	7031	2161	12078
MCM6	24	7362	1905	7183	5988	7929	4981	11790
ITPR2	24	30324	10200	28684	24043	32024	15892	59675
ZNF268	24	78914	25828	72509	64764	81066	46960	152752
IHH	24	21033	9858	18892	13742	25246	8177	47809
ITIH4	24	72348	41569	59410	48000	91020	21519	181795
PLS1	24	54141	21898	51436	36501	71411	22311	105573
LAGE3	24	10057	3912	10139	7681	12811	1726	16420
KIAA0100	24	146256	17859	143572	135855	155331	115924	183594
MDC1	24	2139932	381036	2106224	1834223	2388845	1535804	2958355
KANK1	24	43617	15882	40464	36207	42662	24832	93555
SMC1A	24	265866	53157	255943	224720	281450	212189	424358
RRP1B	24	24005	6569	22894	20677	26770	14122	40578
DIP2A	24	150154	22731	144789	139325	167447	102569	201421
BMS1	24	14215	2597	13451	12469	14990	10424	20607
USP10	24	71682	15361	67116	60075	79671	49489	105248
MESD	24	14783	8178	12955	8692	20055	3685	32915
GANAB	24	46235	28800	37409	29020	55160	11022	145275
KCNAB1	24	92325	35281	83880	66229	126334	39810	152581
LBR	24	10126	4219	9082	6921	13157	4138	18733
MVP	24	16595	11813	14002	8535	21252	4619	61626
LTBP1	24	29187	11779	24794	21940	31114	18152	65867
LTBP2	24	18429	4711	16601	15687	22015	10703	28766
CHD4	24	26136	7061	24450	21494	29490	17511	45244
CRYM	24	34764	11933	32135	24416	46468	16646	56245
KIR2DS1	24	52271	25399	43264	31526	70075	19464	115231

PG.Genes	n	mean	sd	median	q1	q3	min	max
KPNB1	24	57583	19081	56819	46058	66835	23703	96248
PSME4	22	4306	2684	3944	2379	5863	594	11597
NAA25	24	14181	3134	13793	11895	16172	9053	20637
NCAPH	24	55064	17038	52438	42517	67930	27812	96763
PCLAF	18	2022	1594	1881	602	3347	164	5730
WTAP	24	33171	6646	30311	29396	36934	23308	47844
PSMD6	24	9480	2654	9644	6888	11114	5533	15981
MAD2L1BP	24	27510	13529	23885	17604	32606	12081	64966
ABRAXAS2	24	8541	2841	8167	6651	9880	4420	17506
SART3	24	5551	2675	4691	3669	7463	1903	10955
NCAPD2	24	59562	10156	57147	53252	65429	42000	86084
ACAP1	24	21022	5917	19470	17842	23394	10607	33560
SNX17	24	8050	3661	7254	6407	8602	3184	22103
KARS1	24	28589	12539	27257	20531	33655	8084	63693
LRRC14	24	5861	1821	5582	4689	6426	2614	10223
ARHGEF6	24	153471	80196	134244	104945	187451	75319	457158
ACAP2	24	46503	14191	43245	39710	48322	28864	91157
WDR43	24	11550	3549	10987	8837	14079	5717	19287
POSTN	18	5335	3678	4050	3212	7069	845	14840
POSTN	24	14908	7490	14055	10659	16952	5855	39224
EEA1	24	150897	52165	138129	124420	156143	77398	303093
NCF4	24	5769	5199	4281	2093	7941	950	23854
PAFAH1B3	24	73871	36962	64108	49594	87053	23371	163506
PCOLCE	24	7369	5336	5098	4393	7599	2798	22517
PGM5	24	77239	156551	28269	20168	47219	11592	694043
PMVK	24	118916	60433	123626	65429	145250	32675	263502

PG.Genes	n	mean	sd	median	q1	q3	min	max
PRKD1	22	6056	3225	4797	4190	8711	2120	13188
PLEC	23	8333	2921	7614	6365	9441	4539	14759
PPP2R5A	23	5163	2630	4762	3685	5641	1822	12913
PPA1	24	204929	143834	168861	107772	229122	30145	540270
PDGFRL	24	15841	7833	12458	10676	20337	5928	30725
NONO	24	16969	10488	15773	11715	18365	5450	56441
QPRT	24	17196	11420	15222	7756	23351	2384	47216
RABEP1	24	31563	7825	31814	28332	35309	16108	55554
RAB35	24	10204	4570	9347	7645	11964	3276	22093
RCN1	24	11441	7508	9944	4751	16721	2916	24754
RP1	24	20045	5466	19677	17187	20716	11895	34710
ANKRD1	24	9655	2327	10016	7724	11072	5400	13877
TTF1	24	26736	7204	25486	21543	28049	18732	45781
TMED2	23	5208	6062	3216	2663	4268	997	29347
PCBP1	24	241727	89270	229313	205241	289680	83932	432194
PCBP2	24	221550	79267	216738	184932	261902	75435	392932
ELOC	24	9896	4398	9513	6364	11727	3348	19329
RHEB	24	4329	2075	4090	2576	5935	1390	8445
UBE3C	24	19843	7060	18746	15395	22497	8865	37336
SF3B3	24	198564	107867	181822	114180	246759	63845	517599
RSU1	24	20801	21567	14890	11699	17916	10525	114461
CNN3	24	46940	40310	38175	30068	50241	13980	215457
SAFB	24	19639	8237	17569	14452	23782	9432	43387
SF3B4	24	12366	3629	12234	8776	14118	6237	20106
SF3A2	24	86830	21055	79025	73843	94956	58399	138510
SEC23A	24	9429	5241	7537	5654	11731	3532	22638

PG.Genes	n	mean	sd	median	q1	q3	min	max
SEC23B	24	26013	10114	26519	17506	29763	7312	51413
SF3A1	24	33079	15611	31711	22638	44369	7668	75194
SKIV2L	24	46542	16307	41703	36293	52894	22754	83387
RGN	24	8327	7776	6391	4481	8317	2802	41189
CDSN	24	28062	14009	25431	19629	31138	6017	63330
TCEA2	24	26075	16939	23625	20463	26012	10767	100602
TGFBI	24	35416	29506	25392	14050	44576	7942	119089
DIXDC1	24	28921	5147	28084	25801	30155	19833	43302
TRADD	24	7298	2118	6708	5751	8031	4706	11588
TSN	24	73532	34916	60629	47760	82916	41758	155187
TRIP10	24	7509	3747	6494	4950	9162	2705	18281
TRIP4	24	10960	3083	11120	8762	12896	4222	17170
TRIP6	24	25980	13153	22279	19491	31274	10034	72483
MAPRE1	24	27767	8650	27285	20226	33358	15120	44103
TSC22D1	24	14470	5006	14059	11275	17019	5725	25831
ELAVL1	24	63430	34556	55520	37765	80875	18972	157485
INPP5J	24	33162	11625	30429	26559	36815	19405	65018
HERC1	24	970109	123159	959083	911076	1027578	688848	1283812
TOMM34	24	15959	6430	15300	11055	21006	3322	26203
TBCC	24	18286	3721	17886	16255	19493	12492	29065
UBE2V2	24	55333	18595	57212	41662	65701	20456	90937
CST6	19	15014	11825	11592	9528	18015	2105	49974
VAMP3	23	2318	2243	1559	1250	2176	897	9653
NEDD8	24	51909	16665	49859	44558	58973	21455	100385
ADIRF	21	13835	27697	4546	3823	5874	1611	100998
ADIPOQ	24	169100	124922	122494	86920	197134	38747	536435

PG.Genes	n	mean	sd	median	q1	q3	min	max
ATP6AP1	24	13666	6103	12383	9372	17445	5317	27618
RAB11B	24	57081	24678	51022	40836	73348	25523	128691
ZYX	24	22632	17476	18123	14138	22064	6116	83647
ADRM1	24	7191	2942	7143	5183	9080	2166	12612
CCDC6	24	8953	4327	7580	5366	11889	2902	19947
ENOX2	24	6743	2524	6450	4883	7810	2120	14546
LAMA4	24	30883	5624	31260	28530	33610	18313	43996
SSX1	24	6387	6125	4650	3616	6745	2680	33497
CSRP2	24	17531	17967	11877	5646	24588	2020	70668
DDB1	24	45208	16373	41063	32571	60495	22201	86353
MAPK14	24	5026	1198	5216	4392	5782	1428	6753
CDC37	24	41155	11657	38685	32598	47776	23118	75578
DPYSL2	24	698385	533194	574209	311652	925965	137659	2280772
SYPL1	24	23671	8126	21125	18109	28956	11037	42666
RBBP7	24	7730	2503	8117	5156	9031	4044	12980
FXN	23	9289	5237	7781	5055	12780	2758	20301
ZNF239	24	133533	67314	110569	93567	165222	34475	280253
CALCRL	24	26135	17861	21972	18068	25914	3587	82193
SRSF7	24	17572	10164	14383	10970	18912	6196	49186
PRSS8	24	19033	9199	16018	12422	22250	8712	44247
FSCN1	24	62806	46507	43789	34174	77095	13780	172669
IFI16	24	46884	41703	30858	21161	55351	10198	199446
DECR1	24	143522	91444	114321	85294	160435	46359	369243
MAN2A1	23	3912	1464	3488	2988	4243	2216	8150
CLPP	24	20074	7161	17584	14107	25905	11350	35161
TST	24	20433	17846	14258	8695	24378	3797	65555

PG.Genes	n	mean	sd	median	q1	q3	min	max
RTN1	24	4184	1650	4058	3061	4526	2666	10469
UPP1	24	15581	3193	15470	13623	17022	9217	24461
UGP2	24	6895	5024	5877	4059	7897	1644	26470
UGP2	24	69764	32555	56361	50399	88374	24747	134134
TXNRD1	24	37103	20438	33277	21722	41675	12412	78384
IMPG1	24	61989	13048	62112	53812	69807	25511	83872
LONRF1	24	242379	45386	228310	204619	259623	192742	360149
NKPD1	24	7086	3823	5773	4675	9078	3153	20303
HNRNPUL2	24	52886	39907	40780	31711	53907	15331	177413
INF2	24	32849	9590	31056	25337	37285	20284	55075
PDS5A	24	98914	41390	87629	79810	101033	49014	259201
C5orf64	24	19926	9186	16298	14004	24389	6870	49191
CCDC96	24	59334	25223	52443	38411	65776	28195	110801
SHROOM1	24	39465	9301	35682	32418	47240	26843	59138
TSR1	24	61855	25269	55324	42315	66339	29420	135958
IAH1	24	16706	8038	15649	10765	21001	5924	37184
SMU1	24	9346	2910	8784	7616	11026	4334	17973
SMU1	24	111436	55330	93483	85125	128190	47636	310352
ASTE1	24	41089	28961	31862	24823	42824	13947	129555
HKDC1	24	13775	5629	12910	9916	14786	8216	29879
PRTG	24	12549	3769	11710	9544	15667	6509	19458
DEFB115	21	14918	13453	12026	7607	17278	1821	61475
MYLK3	24	314750	102277	310479	264114	348089	128508	572087
LRRFIP1	24	37513	11298	37259	29514	41436	21290	68050
LRRFIP1	24	30349	7939	27186	23310	36955	19973	43956
P3H1	24	36322	7129	35980	31482	41784	23593	53453

PG.Genes	n	mean	sd	median	q1	q3	min	max
TRMT5	24	6917	2218	6234	5664	7600	3913	12817
C2orf76	24	15185	6352	13869	11570	16688	9058	38582
TKFC	24	5033	2610	5047	3464	6986	663	10659
OTOGL	24	39207	7324	38378	32977	45049	24604	52547
LGALSL	24	37416	10872	36975	30741	42620	11336	56983
AMZ1	24	38153	15640	34546	26486	44502	20218	75550
SV2C	24	5697	2154	5329	4159	6668	2186	9925
ZFP69	24	161365	35276	158082	134309	182886	108967	239299
MAP9	24	21918	5206	20885	18213	26288	13306	34568
VPS26B	24	9133	2362	8636	7885	10275	4212	14379
HYDIN	24	355496	64620	343357	310395	373107	286348	555194
TBC1D10B	24	46532	20274	42507	30859	65049	19082	86350
PLCH1	24	221709	44037	216262	192254	244302	134560	321002
FILIP1L	24	64765	13155	61929	57640	68131	46466	109187
CCDC58	24	11251	6497	8776	6668	15564	2983	22856
AMOT	24	35901	17878	30236	24563	43808	19279	98718
ATP13A5	24	13525	4053	13651	10880	15044	4796	22203
TIGD2	24	11070	4416	9821	8875	11568	5714	23741
GREB1	24	10868	2043	10790	9066	12021	7674	15518
TMEM259	24	7659	1558	7333	6676	8499	5120	10616
CCDC38	24	16393	4955	15756	12379	18374	10205	32209
ATP6AP1L	24	127842	83132	102076	83779	146715	37992	407723
CCDC184	24	50826	35749	37508	24576	64638	15768	150773
SBK1	24	8068	1341	7789	6880	9002	6222	10523
PDCD4	24	21171	12288	16787	13012	26961	7750	58341
FNDC3B	24	459800	262494	419342	276735	492731	188640	1232094

PG.Genes	n	mean	sd	median	q1	q3	min	max
NCBP3	24	15387	7309	12270	10425	21155	7072	30800
TP53I3	24	59291	46258	45540	29149	65958	11663	183180
HIKESHI	23	6228	2277	5546	4814	6822	3672	12476
SLC44A4	24	13355	3417	13412	11187	15670	5649	20210
SLC44A4	24	186214	58598	175365	136026	217842	115503	320458
PDLIM3	24	11043	21240	4176	2200	5787	1110	78956
LACTB2	24	11072	4502	10912	7226	14254	3896	19792
EIPR1	21	5055	2206	5055	3802	5875	592	9156
SMUG1	24	17622	8306	16482	13227	21507	4161	35669
SOWAHC	24	26870	9477	24421	19324	32936	16263	56202
LBH	22	3720	1498	3977	2860	4720	394	6699
ARHGAP15	24	60940	11643	60734	54493	67894	41045	87570
FASTKD1	24	72431	14962	71346	64131	78761	43121	109296
ASPRV1	24	104333	53764	90972	75866	122149	33420	236400
HS1BP3	24	19032	13818	14982	11959	21818	7446	74861
INO80D	24	38355	10431	39908	30343	44669	14737	58219
ACTBL2	24	25056	8780	22599	19983	28366	13028	48556
CCDC93	24	113364	32234	107581	88992	123944	70810	191393
OCIAD2	24	64853	30164	60875	44400	71810	20452	154945
HSP90AB4P	24	197441	35341	197592	168525	213933	145091	275347
HSP90AA4P	24	38251	6814	36443	34385	39197	27939	54222
TTMP	24	26480	11888	23975	19914	28460	12557	62909
NOM1	24	53899	13236	51960	44185	61519	30907	90086
ZC3H12A	24	48543	27720	38440	28417	64623	20303	114423
PDZD11	24	64919	23291	61161	46886	73937	36554	133609
CMPK2	24	33482	9958	33363	26980	37396	20804	61175

PG.Genes	n	mean	sd	median	q1	q3	min	max
FBXO48	24	17827	5470	17528	13855	21215	9390	29487
TNFAIP8L3	24	31969	9549	30224	27129	35663	18046	63276
FREM1	24	120563	14827	121137	107155	132884	99062	148356
RTL5	24	168544	44574	172593	137347	185061	94084	301567
PABPC1L2A	24	5958	1789	5917	4886	6426	3365	12559
PRRC2B	24	12384	7188	10903	7111	16689	2607	33365
KLF17	24	16814	12572	13828	7284	18660	1675	46272
COA6	24	5810	2888	4830	3254	8694	2647	11700
AARS2	24	13299	7043	11906	8224	16109	3125	31747
SPIN3	23	9381	7088	6639	5030	11203	1615	33161
GNAS	24	19862	6439	18671	15306	24598	9555	35535
KIAA1755	24	51390	14527	48244	40764	63157	33133	92942
PRSS36	24	7323	3911	6440	5379	8262	2266	20724
SAMD9	24	234392	55495	216073	200731	241482	185775	405360
DGKK	24	151581	30965	146495	129424	161884	117276	251184
SPECC1	24	137235	37567	127155	114450	151115	79933	226687
WDR45B	24	11521	3586	11286	9795	12200	6526	22920
EOGT	24	65976	19648	63951	53667	74020	35434	117979
NEXMIF	24	105725	23315	97544	91876	112589	70716	153924
TBCEL	24	8208	4159	6756	5551	10801	2035	18646
ELFN2	24	128528	19296	126866	115234	136428	101424	181192
TTC38	24	21376	16502	15964	11364	24370	4955	79415
EXOSC6	22	4225	5006	3210	2126	4279	947	25660
RBM48	24	11996	3833	10986	9769	13248	6715	21665
LRRK2	24	84829	11522	83350	78428	91056	61400	107831
PHYHD1	20	6659	3870	5407	4292	8582	2293	17806

PG.Genes	n	mean	sd	median	q1	q3	min	max
HP1BP3	24	251516	267404	180521	126392	239156	71578	1401639
FRMPD1	24	91259	17628	92654	82660	100175	60903	141251
C6orf141	24	24056	4231	24426	21274	26383	16385	34757
GLYATL3	24	82681	23762	81800	73253	100926	20935	121844
FAM120AOS	24	22398	29574	11730	6413	28403	5201	148224
ZC3H13	24	28857	4370	28275	25844	31936	19833	38210
TRAPPC3L	24	6811	1713	6344	5738	8623	3489	9777
ARMH3	24	44477	11085	41862	37115	47432	26513	71886
IBA57	23	7397	6348	5137	3218	7738	1526	25324
HECTD3	24	17123	5913	14681	12258	20685	9772	30536
SFRP5	24	164267	94391	142035	119366	186081	18903	421102
UBR4	24	10821	3949	10179	8632	13305	3857	18368
UBR4	24	229007	45363	227918	195830	256448	139812	339056
ZNF684	24	37672	11079	36423	29171	47208	18531	58657
KIAA1217	24	57654	12661	53637	46059	67417	38451	84899
ADGRF1	24	42222	62923	28784	20905	36971	10866	330571
UBAP2	24	12813	5508	11016	9352	15153	5868	32695
C9orf64	24	13552	6682	12851	7716	18343	1994	24807
KPRP	24	53701	18817	48412	41286	66248	20622	89207
L1TD1	24	325643	95348	291990	266942	361057	215005	558187
SYT6	24	55830	16126	57595	44817	64429	28043	97012
RNF187	24	21834	6496	20108	17363	25156	10824	37718
TTC22	24	10231	1548	9732	9304	11454	7160	12861
DCAF8	23	7316	4272	5896	4605	8279	2999	22443
RNASEH2B	24	5228	1341	4973	4440	5664	3263	8384
RC3H1	24	20582	5150	20727	15961	24426	12820	30821

PG.Genes	n	mean	sd	median	q1	q3	min	max
OGFRL1	24	8863	2813	9290	7551	10148	2952	15126
MAGI3	24	47364	7100	46879	43301	50097	38498	70636
SH3PXD2A	24	32406	7815	31082	27657	34284	21249	56265
RSPH4A	24	53164	26909	49967	32624	64435	21560	128711
DDI2	24	15599	5626	15364	11993	17263	5931	29006
C20orf194	24	15128	5088	13503	11978	17894	9433	28947
H3-2	24	14724	39798	3285	1779	4634	945	181918
C6orf163	24	44530	12259	42669	34869	53230	26337	70159
NT5DC1	24	69788	31518	66168	43765	96446	22420	133976
C1orf195	24	17951	8133	15962	12123	20311	6339	40791
AHDC1	24	191679	34774	192807	179328	202057	125462	300340
VPS13D	24	556728	99869	518236	490151	617979	454481	817267
CROCC	24	108974	14931	110420	97986	118692	83783	138161
AGBL2	24	57529	15127	53238	48583	62501	35761	96416
ATF7IP2	24	16347	7373	15380	10954	18851	7187	38930
RASIP1	24	26805	5476	26535	23562	29021	15060	39339
STRIP1	24	36600	9889	33264	29782	40883	25943	68299
SYDE2	24	49349	10613	48434	44036	53186	31232	77354
RNF220	24	10661	2553	10846	9047	12336	6503	14872
PRPF38B	24	78025	30427	74610	53065	91656	39988	164448
RNF20	24	37644	6577	36462	33591	41005	26728	50053
ZNF318	24	123181	19702	120758	109023	135027	91389	170108
DIPK1B	24	29198	6665	27249	25640	32708	19571	41436
ATRNL1	24	63569	15542	62165	56516	68911	34353	105535
SPATA31A6	23	16842	24939	9147	6359	14789	1472	122318
BROX	24	22516	12142	19968	12443	31249	7078	57445

PG.Genes	n	mean	sd	median	q1	q3	min	max
FOCAD	24	169702	32084	163966	148563	198301	117910	215600
TAF3	24	108471	32487	102372	94577	115616	64692	233228
PRAMEF8;PRAMEF7	24	13006	4531	12483	10338	14780	3800	23984
RSBN1	24	7328	2990	6340	5428	8198	3873	15282
LYPLAL1	24	13865	8809	11371	8990	14955	7248	49779
ECPAS	24	7813	1742	7996	6608	8700	4720	11703
TSHZ3	24	18673	4566	18025	15225	20423	13373	32142
AFMID	24	14433	4498	14233	12912	16222	4542	26355
KANK2	24	76655	32498	67557	53730	94381	42706	159562
WASHC2A	24	19389	11132	16834	12827	20059	8419	49465
EEF1DP3	24	21559	10038	19733	14606	25258	9799	57835
FAM91A1	24	115985	69555	92270	73792	122373	48338	302857
LARP1B	24	11572	4948	9909	8335	12274	6264	24735
CEP135	24	43377	4858	43908	39299	46097	35194	52072
TMEM198	24	227174	150300	168544	131753	250373	71326	687480
MAP1S	24	26379	7522	24470	22832	27351	16561	53453
E4F1	24	20572	10789	16969	13431	26549	6092	46090
ATG9B	24	91695	39973	77066	65674	110131	41733	198645
NUGGC	24	270492	79340	252449	221742	286339	177519	523536
TNS3	24	167972	39672	159691	147104	183345	105823	283690
ANKS6	24	789992	208814	781717	702679	900833	451537	1484496
LMBRD2	24	39459	11181	36892	32581	50404	13134	57945
ZFYVE26	24	43719	8143	41690	38575	47420	34186	68259
MSL1	24	13366	3833	12533	10652	15496	8429	22111
C18orf63	24	32833	8877	31249	24997	39515	22084	54257
CWF19L1	24	218664	98044	194512	153317	245421	94214	477907

PG.Genes	n	mean	sd	median	q1	q3	min	max
CYB5R2	24	19066	14122	14588	9451	21031	4436	58960
UHRF1BP1	24	30413	5950	28144	26138	33890	20391	43429
SP5	24	8889	2891	8325	7454	10486	2676	16471
ATL3	24	32964	8359	31420	27580	36027	17567	52692
NXN	24	12863	5855	10632	9585	13306	7563	33122
ZNF470	24	23556	5167	22166	20590	26475	17082	41935
VASN	21	5890	8225	3087	1997	4632	1079	32251
SFRP4	24	282130	135732	271341	220501	298389	152265	826964
CIAPIN1	24	77066	30307	77101	57038	92098	18988	129029
SMYD5	24	3963	1302	3775	2971	4769	1892	7029
PTRHD1	22	9337	5918	7386	5645	11457	2771	24175
NADSYN1	24	5411	3051	4283	3240	6433	2437	13715
LAMTOR1	24	37500	18350	34001	26921	43253	12015	95218
TWF2	24	12767	3447	12219	10423	14814	6662	22108
PHETA2	24	15152	13531	12947	7682	15716	1463	65922
TANGO2	24	22161	9714	19940	15286	27081	9536	47351
SPOPL	24	12607	5474	11521	9820	13105	7535	34744
RAB12	24	50958	18129	46062	36975	64441	24937	89289
KLHL10	24	12140	2465	12156	10604	14063	5581	17792
KRT80	24	12462	2159	12921	10883	13599	8594	16207
NIPBL	24	77058	15165	71427	65313	88655	56005	109677
TET2	24	101671	32248	100258	77667	118929	49912	169493
NAA16	24	212543	39788	215887	186357	226881	140692	319640
CHADL	24	33541	15906	28459	24677	41866	14789	79141
PSAPL1	24	143354	40700	139853	118827	165531	59987	256094
TYW1B	24	7322	3160	6534	5759	7903	4479	20273

PG.Genes	n	mean	sd	median	q1	q3	min	max
DPY19L2	24	17316	3553	17283	14303	19456	11355	24917
CRACDL	24	77862	9277	77341	70333	85273	61995	91469
NIPAL1	24	4988	1816	4323	3736	5737	2801	9860
HIBCH	24	609520	365279	510636	331236	746276	162034	1723305
ZNF774	24	56365	25220	56051	36534	72006	14920	101590
ANKRD54	24	324075	205784	274360	163294	488534	87989	717658
PPP1R18	24	8204	2904	7396	6167	9467	3839	15769
CAVIN1	24	107773	93571	81289	66123	92082	54357	430248
ZCCHC8	24	3110	892	3036	2475	3618	1966	5657
DHX57	24	69682	7364	69610	64110	73713	57653	89737
MRPL54	21	6081	4267	4202	3206	8258	2013	18869
CDC73	24	26238	13050	21824	18860	27603	14333	71650
TATDN1	24	199201	66514	208232	145284	232226	96770	334705
METTL2B	24	6940	2637	6258	5399	7705	3472	16302
C8orf82	23	3373	1100	3361	2862	4077	804	5554
EDC4	24	4097	1331	4123	3154	4481	2308	8641
PRPF8	24	5740	1310	5624	4950	6165	3404	9954
NEK5	24	3569199	1477198	3066016	2731713	4295766	1737792	6985820
SCYL2	24	55012	12286	51558	45562	59894	35689	86124
TTC27	24	12947	2247	12591	11431	14165	9422	18412
PLBD1	24	47700	28303	43368	23393	69240	11924	103983
LMOD2	24	90615	63732	82456	48875	119348	18857	268197
NOTUM	24	8017	2947	7164	5667	9103	4269	16151
GIMAP6	24	32567	8131	30512	26890	38464	23092	56960
VWA1	24	115722	60830	106075	81063	124468	47119	314485
PGM2L1	24	26019	16191	20256	14551	31574	10445	72476

PG.Genes	n	mean	sd	median	q1	q3	min	max
CTR9	24	42798	12877	39588	34195	47663	26334	69471
TEX38	19	8234	5868	6015	4698	11206	412	24967
TTC37	24	78035	11839	76960	68211	86077	62696	104922
DARS2	24	63629	14076	66151	54490	69414	41001	97675
FBXO46	24	11346	9204	9472	7404	11775	4609	52808
LARP1	24	61435	16866	56040	50219	66816	40352	101420
ATAD2	24	30665	8960	29044	25470	31776	16959	57224
CPLX2	24	10479	2674	10239	8872	12496	5918	14875
SLC25A47	24	90115	18287	92361	80809	102433	58460	129420
SPAG17	24	168456	33364	160333	144731	182858	125451	247042
SPATA8	22	2302	1365	2050	1302	2810	554	5817
MTHFD1L	24	8691	2295	8506	7512	9444	3756	16096
CSPG4	24	3958736	622620	3947071	3531518	4173376	2823651	5488224
DHRS11	24	21595	5216	20702	17807	25675	12065	37816
SBSN	24	67455	16219	66527	53418	75748	47123	111652
ENPP7	24	17961	3861	17806	15951	20094	7681	24225
OLFML1	24	18006	5967	16496	14144	20881	9945	33203
CWC27	24	53841	14334	58638	46384	65948	28108	71177
OLFM4	24	78245	71926	52421	37201	65327	23679	294478
LAYN	24	66726	45750	53701	32791	90633	8421	162248
PLXDC2	24	36286	14765	33832	29219	38189	15712	89539
WDR82	24	7968	2360	7929	6118	8793	3463	14275
MUC6	24	3833	1411	3455	2783	4918	1901	7198
RAB11FIP1	24	14145	4808	13080	10969	16504	5599	29092
ASCL4	24	6477	1583	6465	5089	7491	4180	9924
NAPRT	24	23434	9700	21393	17082	26442	11383	45651

PG.Genes	n	mean	sd	median	q1	q3	min	max
GIGYF2	24	108875	24459	103370	92600	112809	83773	178184
CD109	24	18781	8102	16447	13401	19324	9421	40652
HSDL2	24	32048	11916	29231	22070	42053	15048	54971
KYAT3	24	21266	6309	19870	16277	26032	11936	36296
PPP1R21	24	4468	937	4471	3815	4897	2378	6173
GLDN	23	9427	3064	9309	6854	11557	4681	16115
THSD4	24	46533	12476	45311	35893	53039	33296	85498
TMPRSS11A	24	7989	3298	7257	6088	8459	2763	17856
ZNF783	24	34675	17375	30936	22999	47706	10439	87868
TRIM72	24	11976	2835	11993	9504	13874	6403	17055
LEKR1	24	14271	6463	12831	9878	16048	7460	36004
ZNF782	24	11898	5830	10306	9313	11915	3932	31228
SPOCD1	24	47711	19023	44227	35515	54185	29051	121159
MEX3B	24	62321	20669	63637	50468	72779	28453	106005
BNC2	24	132914	55887	138077	88421	179053	38051	248364
GBP6	24	397977	120832	374441	315843	441256	199723	702954
CCDC81	24	107337	22267	100433	91173	127282	67675	146661
ZNF836	24	2730	886	2440	2238	3193	1164	4718
RNF111	24	185614	66444	171314	151226	217206	64956	336775
FRRS1	24	351181	534369	214507	85777	273721	43773	2584029
FGD5	24	26781	11551	22530	18356	35086	12497	49126
RBM44	24	98145	23027	98781	82538	108830	55880	149331
SLCO4C1	24	24698	5998	23416	20446	26405	18402	39861
WDR87	24	54109	11204	51391	47951	59000	40406	91600
CATSPERG	24	55236	27468	48391	39432	68652	11471	121391
FAM83H	24	12280	3476	12071	10369	13612	6293	22761

PG.Genes	n	mean	sd	median	q1	q3	min	max
LINC00696	24	4278	2637	3611	2873	4492	2077	14741
ZNF662	24	65158	32046	51815	45746	89100	27356	143180
C1orf122	24	6675	1976	6323	5305	7876	4208	12069
LCNL1	24	27553	11072	28027	19253	36638	4035	51595
CDHR3	24	92848	34942	81925	74691	111182	40807	173800
CFAP47	24	161179	35537	148622	138886	168459	115358	270442
MSANTD1	24	6681	2199	6924	5301	8065	2225	10936
UBN2	24	123854	26827	122100	99688	144408	70163	171111
FAM205A	24	52353	15794	47854	41158	57543	36442	101861
CEP128	24	46488	7540	46343	41038	50055	30797	60891
MROH5	24	13407	3767	12420	10415	15087	9387	21895
SPATA31E1	24	37200	7977	37115	29850	41834	24398	57161
CCDC121	24	56553	20227	50769	45366	66398	22776	121984
GPRIN3	24	16157	4088	15383	13393	18880	8048	24620
CFAP20DC	24	8292	2664	8153	6755	9733	3083	14272
NCCRP1	21	8806	3284	8673	6503	11026	2402	16753
MICALCL	24	129002	36704	118608	102843	149301	81183	251915
TMPRSS11F	24	70962	15439	67417	61187	83077	45423	100729
USP31	24	38229	9119	36974	32290	43105	24382	64004
RAPH1	24	22175	6743	21109	17351	24647	11784	36408
USP43	24	71694	24609	63387	56667	78692	44262	147737
UBE2R2	24	11954	3832	12432	9571	14690	5543	20952
CBLL1	24	10714	3358	10204	8822	11854	4257	20329
FBN3	24	119234	42011	107859	90835	127455	60974	220129
MTSS2	17	2375	1518	2099	1524	2879	676	7052
BRINP3	24	71132	18247	71024	60756	80545	38954	104604

PG.Genes	n	mean	sd	median	q1	q3	min	max
SSH2	24	27228	13386	25199	18393	34109	8801	59701
SUPT6H	24	151265	43778	135612	122151	161250	101165	262485
SND1	24	34699	19833	29933	23446	40680	9154	107231
DDX46	24	134293	51732	126305	100425	168215	79164	309390
TRIL	24	427372	132250	435243	313994	534695	207102	669998
TRMT10C	24	498960	132745	469805	422948	523110	308760	854909
CHST9	24	7664	4497	6391	4921	9206	1589	22160
ASRGL1	24	77355	61262	66967	25536	123138	979	207287
KCTD9	24	205518	103515	186470	141363	253975	57682	515891
EIF3M	24	8215	4267	7380	5284	10017	2780	18102
MEPCE	24	14281	6252	13698	9488	18905	4124	26316
PARS2	24	112131	44747	104552	87082	116126	50520	206300
CYFIP1	24	29924	9121	30149	20964	37373	16773	49671
COPS6	24	83981	31481	80594	61464	102483	38219	162016
EPM2AIP1	24	3050	661	3125	2696	3410	1328	4020
TAOK1	24	23752	8219	22821	19206	26486	10115	51907
KDM3B	24	26432	5897	24978	23494	28111	14745	43370
CHMP1B	24	60834	18972	62627	52094	74374	12475	95716
CHST3	24	20973	8316	19340	15805	23315	8086	46341
OTOP1	24	7008	2724	6391	5394	7513	4307	15430
MICAL3	24	166031	25527	165463	146129	185252	127174	215303
PHF5A	24	8646	4611	7833	5743	11383	2510	22164
OVCH1	24	9213	2105	8771	8071	10164	5594	14041
OVCH2	24	8096	2636	7888	6193	9260	4300	14391
TRMT1L	24	31007	18631	26087	21406	34359	12525	102291
ZC3HAV1	24	23856	10545	19554	16733	27782	14114	55495

PG.Genes	n	mean	sd	median	q1	q3	min	max
GVINP1	24	114434	20382	111047	99910	120881	75932	164771
NUP54	24	9586	3129	9220	7832	10295	4889	19278
DGLUCY	24	13279	4048	11972	10623	15617	7501	22642
CCDC186	24	129527	40342	118266	100837	143406	81996	219946
VPS35L	22	5378	2759	4734	3546	7316	1598	10698
ZFYVE16	24	32583	8563	29606	26371	35595	21605	54112
MYH14	24	83494	16243	82516	71187	93399	58720	116026
NUFIP2	24	36895	7335	34185	32515	41865	27216	52826
SZRD1	24	28217	10683	26335	20193	32515	11316	62223
MAVS	24	10686	4013	10997	7485	13152	3609	17463
PKD1L3	24	41132	12891	37936	33620	46098	25757	89497
DHX29	24	206428	91320	188634	151548	221154	105440	537434
NPHP3	24	15167	2320	14627	13576	16130	12447	21952
HDDC2	24	12586	5893	10531	8918	15911	5546	27667
POGLUT3	24	1712163	816431	1554529	1226724	1876951	894743	4953718
LIMS2	24	22381	7963	20557	18237	27330	9471	38049
DCXR	24	54305	18463	51430	38656	66655	22781	98800
AKNA	24	14521	2740	13977	13106	16066	9588	21502
TAFA1	24	81424	28665	80593	64521	95539	14204	129709
POLN	24	56888	9916	56232	49490	62431	38998	81412
PPP1R32	24	72185	21421	65410	59883	76958	52334	151084
SNX20	23	135961	61953	128819	85045	174716	52663	310994
SPRED2	24	28045	15578	24901	15914	36056	3970	67238
ARPIN	24	16991	10188	13994	11560	18142	7083	47360
HUWE1	24	66228	18972	61479	50995	77530	45187	111797
YTHDF3	24	16423	5297	15040	12688	19431	8169	28876

PG.Genes	n	mean	sd	median	q1	q3	min	max
CTU1	24	25378	14310	21405	16715	25802	9634	63233
ABI3BP	24	38618	9442	36042	32362	46000	24474	62149
ZNF467	24	76031	22909	72875	62790	83875	45025	157422
C11orf96	24	50145	28470	42145	31347	54571	19224	125375
MEGF8	24	17194	3579	16392	15604	19895	10899	23989
GALNT5	24	164253	25582	161157	151906	171913	120189	217651
GALNT7	24	37188	10037	36224	28569	44837	22930	55867
PHLDB2	24	20955	6042	18937	16852	25435	12556	34079
WDR86	24	156653	49465	152219	123310	189963	39731	245364
SETD3	24	38586	19357	34046	29650	39362	15195	120966
TTC7B	24	11847	4143	10562	9591	12522	6994	21735
ADCK1	24	113354	19882	112304	103081	129016	65780	146473
ACOT1	19	2773	2131	2102	1565	3052	689	8647
NOP9	20	3262	1163	2808	2327	4400	1251	5430
METTL3	24	29493	11965	26457	22162	35591	15142	64192
PRPF39	24	40479	20010	34412	31147	46765	10865	113729
SAPCD2	24	37767	8350	37859	34266	42426	15878	58891
OAF	24	4855	2187	4549	3371	5950	1618	10589
ZNF546	24	21303	4155	21423	18535	23462	14565	28902
MTDH	24	141022	70823	117833	91352	162379	55859	344343
LRRTM1	24	7257	3054	7044	5560	8418	2826	16125
ABCA12	24	66006	6171	66021	62422	70010	55164	77209
RTN4RL1	24	30761	18933	26442	20953	35105	4175	99699
RTN4RL2	24	10998	7024	8249	7447	10473	5173	29604
SMG6	24	47121	15312	43387	39328	49208	28333	101318
BCL9L	24	279136	53971	280452	232708	323016	181176	363398

PG.Genes	n	mean	sd	median	q1	q3	min	max
ITIH5	21	24110	4222	23908	22205	26613	12696	31353
FERMT3	24	31333	16054	28539	19317	39652	11862	74356
LUZP1	24	8882	2493	8174	7394	9856	4855	16907
ALYREF	24	20007	10135	17893	13334	26495	5894	47807
FAM160B2	24	22740	8901	21015	16181	24499	12375	44170
ZC3H18	24	11507	3263	11795	9098	12825	6301	20258
VPS36	24	21159	8186	19070	16817	23364	8465	41100
CAND1	24	92715	45679	77903	58782	114178	33434	194231
GLCCI1	24	9671	4027	8239	7267	10908	5748	22555
TXNDC2	24	289434	127606	232287	207915	309747	175800	634940
HOOK3	24	116319	34084	114176	91988	127160	73151	217534
COMMD7	24	2941	1126	2526	2223	3501	1207	5754
METTL16	24	15035	3279	15115	12514	17263	10063	20612
ZC3HC1	24	6047	2834	5309	4529	6399	2814	15302
PKHD1L1	24	27452	6392	24886	22643	30606	20158	44471
CCDC25	24	18881	5993	18775	12998	22200	9764	32269
CARM1	24	10246	5226	8906	7682	12861	2677	22637
CIR1	24	5572	3382	4613	4062	6524	1408	18591
FAM131B	24	10708	6351	9652	6115	11981	2013	25099
MICU3	24	33791	7706	33834	30824	37152	19552	51365
ZNF575	24	10075	3564	9151	7340	13117	4931	17912
PPP1R3B	24	87776	43070	78938	59824	106799	23871	187309
ZSWIM9	24	21080	4555	20278	17699	23010	14453	30186
ANKLE2	24	104834	25095	100642	85929	116920	59240	171842
DZIP3	24	26601	8150	24277	21060	29974	16150	50878
STX12	24	6149	4019	5075	3070	7243	1994	15553

PG.Genes	n	mean	sd	median	q1	q3	min	max
ZGRF1	24	47790	13411	44781	42061	51371	30691	86050
ERO1B	24	30769	7149	29671	25795	36636	15387	44205
ZNF280B	23	3674	1932	3385	2322	4452	924	7795
MARCHF9	24	19112	5486	18643	16722	21137	8707	33710
ANKRD13B	24	309949	91209	305591	242590	360551	121648	490368
TMPRSS6	24	75875	26331	68796	56609	87287	41234	137519
IRF2BP1	24	344697	291491	255443	174418	347785	122861	1346881
RHPN2	24	69061	37743	63028	49519	80727	14608	176414
TGIF2LX	24	7371	2623	7090	5469	8504	4058	16238
H2AC21	19	21121	24308	12946	9291	18739	1537	96981
AEBP1	24	2172706	1329319	1803273	1254251	2563521	794680	5996562
PHYKPL	24	371486	155493	330239	282966	371700	218224	808963
PLD3	24	14292	9355	11239	8849	17260	4458	45746
LIX1L	24	244486	75298	251526	185271	286138	140170	419882
ZNF584	24	240771	100539	214144	182891	289487	102348	474210
NUDCD3	24	26831	7829	26071	20942	32175	14409	41953
P3H2	24	29568	16055	26390	20816	30636	9517	69180
P3H3	24	7449	2969	7009	5290	9392	2611	12932
MUSTN1	23	3574	2174	3032	2235	4241	1119	10437
MISP	24	7037	1829	6678	6360	7669	3849	13118
IGDCC3	24	94602	13270	93255	85925	104737	68298	115679
CDKL3	24	15978	6688	14747	12267	17406	7409	34194
APLF	24	27811	11049	23844	20566	33750	12032	57112
MAPKAPK5	24	81770	37975	78086	52687	106087	19782	157184
NAXD	24	4861	2450	4194	3197	6035	1338	10590
NAXD	24	44421	36083	34528	29321	43664	13349	175180

PG.Genes	n	mean	sd	median	q1	q3	min	max
WDFY1	24	17139	3819	15832	14536	19205	11041	26731
TEX2	24	66406	17077	63273	54900	70878	40311	106483
FAM114A1	24	24932	7925	23683	21216	28341	9350	46191
GCC2	24	52313	11610	49724	43136	59808	34093	74557
HSCB	24	28804	11670	24961	21690	32534	6264	56446
CUL9	24	2953277	403363	3021259	2663893	3209557	2094879	3755502
LMTK2	24	65463	17280	64592	50994	74284	41623	101748
SULF1	24	836208	449569	699565	616471	885257	181959	2386583
UBR1	24	10219	2761	9324	8296	11338	7096	17051
CHERP	24	31516	6539	28452	27293	35707	21191	49581
SCUBE1	24	6072	2204	5782	4825	6986	2421	11982
TRIM42	24	9103	3455	8213	7275	9235	3976	20084
SUGP1	24	439720	94174	425189	372135	496455	281350	641907
UEVLD	24	13991	3706	13294	11962	15894	6613	23123
CCAR1	24	56904	11412	55935	49390	61191	33699	87047
DNAJC10	24	56973	24072	50057	37308	68777	21500	118150
NELFCD	24	48433	20091	43008	38864	52318	28446	126991
ASXL1	24	249614	107424	199539	175618	292510	117057	511297
PHC2	22	61282	63519	39287	22265	80307	1778	232453
MSRB3	21	7009	7909	4539	3525	6057	1002	34998
IQCF2	24	235014	91667	228539	164056	300188	62113	425812
MRPL41	24	17010	4373	16334	14097	18773	8981	26858
ETFBKMT	24	688701	311741	620125	504385	812484	336015	1747497
FAM217A	21	41845	84568	18776	16996	33934	673	406632
RBM12B	24	89820	30013	82363	69504	96191	50664	157490
ZC3H3	24	8851	4091	7131	6369	9442	4340	20846

PG.Genes	n	mean	sd	median	q1	q3	min	max
DDX60	19	7385	5789	6321	4304	7087	2922	26977
MICALL2	24	19120	4186	18542	16350	22284	11060	26189
RAVER1	24	8700	3424	8315	6184	11757	4000	18090
FTSJ3	24	5442	2293	5836	3901	7037	855	10530
MKX	24	48676	8414	47184	43407	52492	37657	77436
DIS3L2	24	15645	4504	14774	13453	16428	9256	28141
ZNF595	24	22094	6993	21309	17569	24135	11561	38171
LAS2	24	31164	6325	29746	27084	35738	20727	46946
EXOC8	24	37367	22513	32598	28151	36589	22564	136656
TRMT44	24	10600	12470	6544	5555	7858	3868	58761
THNSL1	24	17813	3364	16817	15819	18996	13424	28079
CFAP206	24	29790	13994	24948	22330	34509	17276	77771
PM20D2	24	6060	2362	5384	4588	6946	3467	14113
HACE1	24	30850	9191	30982	25383	32603	18843	68439
MICU2	24	8715	4235	7794	6001	9048	3779	24007
CHPF	24	103691	24470	97823	86670	120144	68994	168042
NRSN1	24	10165	9730	7033	6248	8738	2801	48534
ALDH16A1	22	12865	11175	9747	7450	15582	1822	57230
RTKN2	24	141394	80032	118280	94998	178161	31454	293268
ADGRF3	24	16057	6282	16546	12822	19396	4129	27392
PELP1	24	12493	24807	5529	3733	7539	2838	124848
ZNF654	24	10776	5202	9267	7611	12229	4664	25683
C1orf87	24	25812	7338	26310	21090	30913	11935	41097
SPART	24	75327	15942	69699	66062	81535	51055	117170
PGAM4	24	17719	8042	17918	12811	21364	1240	40595
CNPY4	19	4577	2843	3962	2887	5772	849	12963

PG.Genes	n	mean	sd	median	q1	q3	min	max
ADSS1	24	9462	6339	8107	5687	11389	3964	34806
AHI1	24	94753	28452	85110	75182	100635	57484	163729
NAGS	24	30111	9396	27661	25084	33888	16816	54598
ZNF567	24	14685	3525	14390	11627	16017	8754	21370
CARF	24	47588	36640	35334	26793	61859	11146	189025
C4orf33	24	11055	4129	10986	7053	14802	5598	21253
C1orf158	23	5947	2895	5137	3861	7263	1606	13003
STK11IP	24	16914	2929	17514	15514	18545	9656	21366
LRRC47	24	29837	10436	30283	22505	33523	14690	63289
CRYBG2	24	50113	12827	48178	42153	57184	28018	80580
VWDE	24	9936	2123	9893	8272	11689	5575	13191
ZCCHC24	24	30157	15425	26854	18565	35805	11547	84359
GHDC	24	239357	128753	226837	162725	263149	96579	646444
OSR2	24	19315	7012	18444	15595	22098	8076	34577
LTBP4	24	208284	80741	182551	161322	244092	114534	426494
PIAS4	24	128454	41911	119106	102151	157540	56175	230929
GPD1L	24	56517	32397	46063	36154	65114	17029	158695
EHBP1L1	24	35683	5005	34868	32257	37271	29610	52710
MICALL1	24	66559	20769	59797	54010	76197	38897	132005
TXLNB	24	63828	17359	63414	49958	70803	27901	105836
PHTF2	24	47250	17988	39838	37454	51810	28395	107449
FNBP4	24	17100	7073	15156	12049	20307	7442	38301
GUF1	24	77142	16493	74741	64563	85836	50023	117463
RIBC1	24	10123	4927	8165	7009	12418	4204	22518
SFRP1	23	3058	1676	2605	2031	3449	1236	8592
LIX1	24	34966	15591	33813	27025	37766	10984	74625

PG.Genes	n	mean	sd	median	q1	q3	min	max
PNKD	24	925786	817412	754801	387612	1248108	19047	3728656
GALNT4	24	6213	1848	5708	5205	7106	3466	11872
BPIFB2	24	112083	94296	87602	46931	146584	11919	451736
MARVELD2	24	179931	77263	156421	128594	237369	69101	348622
CPA6	24	10291	3091	9738	8435	12597	5871	17034
CBR4	24	6995	2587	6690	5581	7648	3020	13707
AFAP1L2	24	107642	28756	104349	89773	120084	58739	194118
TTC39C	24	47880	10042	47446	42268	53248	28266	72812
FAM89B	24	139701	106205	105427	78066	153206	30297	482150
ARRDC1	24	2737	1753	2143	1481	3393	789	7557
SLC25A41	22	7867	11003	4739	3155	7436	971	52497
CPSF7	24	16957	7760	15400	11355	20546	7489	39758
ESX1	23	7758	3358	7077	6165	8476	2331	17664
ARFGAP2	24	121187	27915	112671	105009	137919	74072	201715
OTUD6B	24	15346	6255	15440	10986	19696	4871	29698
SIRT6	24	737662	321169	674889	486034	905546	303862	1647946
CCDC185	24	57356	11454	57100	50852	64507	31536	77786
SLC66A3	24	140466	135274	116114	80888	155576	25763	735332
ZNF525	24	8602	4945	7484	6711	8783	4940	30178
PAF1	24	9860	5109	8136	6078	12129	2000	22063
PAF1	24	53045	10781	53529	48926	57913	27889	73439
ZNF283	24	1199423	995193	1020927	601487	1208018	473534	5045665
ADGB	24	32620	9590	30905	25642	37692	17384	51766
ANKRD31	24	67570	11850	64381	61160	73893	47072	95433
UBR7	24	28664	14162	24839	22034	28245	15519	70609
ZNF614	24	56148	39787	48630	38879	61157	4889	218549

PG.Genes	n	mean	sd	median	q1	q3	min	max
DDX51	24	10937	2825	11170	8718	12242	5851	16410
KIAA1958	24	17847	5163	18461	14211	20308	6311	28386
PTGR2	24	8120	3725	7338	5688	10640	2530	16101
SLC25A29	21	17832	13383	17108	10037	18323	5474	70699
PNPLA1	24	28005	6802	26643	22781	31636	18559	44676
MAB21L3	24	27814	9238	24675	20040	34769	14827	45561
ANKFN1	24	10771	4140	9764	7780	14731	4060	20113
ZNF709	24	102005	19570	102557	89275	110241	69604	146232
IGSF22	24	64276	27672	56028	47108	66821	30031	132126
DTX3	24	35780	9671	35590	29730	40973	18739	57969
ASCC1	24	5632	1652	5244	4482	5868	3726	10415
LRRC57	24	6993	2578	6655	5526	7590	3906	16512
FSIP1	24	178685	70760	155788	132576	191444	108071	385740
WDR31	24	3504	924	3467	2951	4170	1797	5238
C12orf50	24	23884	7332	22157	18960	27754	13214	45207
TEX45	24	129381	29569	118999	112045	148633	72828	186297
FAM47B	24	5236	1485	5207	4095	5823	3061	9396
MARCHF10	24	39575	11311	35385	29472	47119	25158	61952
ZBTB38	24	109742	39537	102079	88389	118785	64185	253322
PRPF38A	24	24517	10980	23465	19143	28042	8628	61629
KDF1	24	58063	26288	52613	39355	71029	17264	126490
MLKL	24	21917	5070	21506	19690	24716	9461	35487
NHLRC2	24	12333	5235	11115	8368	17630	5474	23271
AVL9	24	34541	7541	32279	29392	39703	22450	50745
COLGALT1	24	17026	7697	14371	12460	17550	10898	45950
POGLUT1	24	1580637	2413876	999881	820832	1443193	621308	12796210

PG.Genes	n	mean	sd	median	q1	q3	min	max
SLC4A11	24	15945	7410	15878	12205	18568	4218	37036
TXNDC5	23	9433	5764	9036	5098	12783	1448	21663
RNF149	24	38651	44582	26154	21485	46385	6345	232814
LEMD2	24	6393	3607	5431	4044	6645	3397	17181
NOA1	24	23276	5847	24106	18617	27636	11535	34439
FAM98A	24	53085	13226	49868	43589	62365	35835	82661
MTMR14	24	28321	8847	25873	22668	32761	17236	56060
NFATC2IP	24	13471	6311	11491	9742	14908	5613	29653
GALNT6	24	202724	51742	205842	165905	233260	121227	295350
NAXE	24	33902	16036	33431	23406	39144	7833	75820
CARMIL3	24	44561	11958	43406	34499	53770	28304	74868
RNF214	24	39594	10592	38043	32024	42029	27277	69023
LSM14A	24	6508	3844	5431	4690	6833	3204	22329
C3orf20	24	13095	7188	11146	8081	16605	4146	31941
MROH1	24	9034	1464	8660	8294	9433	6503	12644
MAPK1IP1L	23	15455	9499	13598	12382	18586	3315	44882
TNRC6A	24	108561	19035	109233	94516	119480	79816	171800
ZNF738	24	89984	26414	84003	76854	96227	60081	191148
ABCF1	24	22630	6422	21001	18329	26671	11701	39229
ACRBP	24	15318	14959	11781	6715	18160	4430	76437
PIK3C3	24	842110	205063	800527	719421	891352	574453	1482474
CATSPER1	24	9090	4500	7202	6539	10830	4535	22064
TTC16	24	38022	7450	37867	31904	42647	24746	56804
NGDN	24	15726	9974	13133	10202	17324	4129	49261
RTL9	24	94434	13012	91009	84938	103277	77791	132178
SDR9C7	24	21431	4586	20897	18693	23301	12435	33812

PG.Genes	n	mean	sd	median	q1	q3	min	max
SPATA4	23	9038	5764	7493	5820	10905	3268	29895
FBXO22	24	12758	4793	12254	10261	14051	5283	24707
BOD1L1	24	139163	30809	130630	121570	143202	107462	239658
ANKK1	24	87724	24782	82497	73849	94711	40126	153595
NUP37	24	18258	4255	17871	15314	21748	11337	27017
NUP35	23	2337	1666	1927	1356	2514	743	8333
REPS2	22	3298	2178	2745	2063	4075	1292	11382
GPRC5A	20	4629	3547	3340	2329	5855	825	14260
GJC3	24	40232	35209	27159	21566	48734	11436	170781
MDGA1	24	7141	2394	6431	5705	7189	4906	14266
NBEA	24	210870	39745	203497	180597	238454	143834	287862
CCDC148	24	22995	5257	22981	20685	26089	8245	32308
DNER	24	46330	21248	46453	30648	64154	14468	105240
TSTD1	24	17243	9945	15027	10990	21948	2107	37907
TSTD1	24	23915	6264	23133	19397	26731	14196	42377
CMAS	24	21040	8907	20230	13808	27199	9153	45368
TRIM58	24	46774	28918	34453	26833	64400	12583	121557
KNL1	24	90621	22228	85216	75856	107916	52399	135332
OR56B2P	22	22668	16061	16295	11470	34411	2135	60580
OR13C9	24	12460	7420	11833	6707	16589	2474	31170
OR6N2	24	8230569	4401560	7564012	5356137	11062994	1234612	21767660
OR2T3	24	38418	17055	35763	30075	40062	16304	101858
OR51A7	24	12400	5704	11588	8858	13361	5725	27658
AKR7L	18	30302	21092	30102	13923	38084	1178	78467
PLBD2	24	16882	6426	15508	12601	20392	6611	28873
SPATA22	24	35384	13292	34737	25747	39520	20491	72463

PG.Genes	n	mean	sd	median	q1	q3	min	max
TDRD7	24	3459590	1105173	3003988	2715953	4051853	2071415	6166425
GIMAP7	24	6651	1766	6386	5741	7809	3234	10603
GAS2L2	24	61459	11626	59579	54197	64871	44093	94211
HUS1B	24	112184	46880	95934	80028	127158	67245	233138
THOC2	24	164452	31583	156144	148209	179070	116407	263497
WDR36	24	41401	10501	39372	34464	48572	24304	61069
COQ8A	24	165607	60299	144536	122330	186533	99420	335498
SLC30A5	24	9797	3064	9234	7937	10779	4742	19347
GADD45GIP1	23	3346	2823	2195	1637	4871	512	12961
WDR48	24	28031	5039	28541	24925	31296	17765	39683
TYMSOS	24	15661	3746	15055	13419	18249	8758	22347
SMARCC2	24	18472	5947	18084	14404	20695	10799	36714
NPLOC4	24	369106	111108	388262	295722	422703	169940	674046
NA	24	49051	14393	46290	37423	56479	30713	78753
ZNF519	22	3185	2543	2404	2018	3454	881	12835
PIGX	24	39910	21863	31746	27523	45171	12562	110277
TBC1D16	24	17747	5306	17842	16158	20424	4907	29443
CIRBP-AS1	24	14240	4277	13298	10537	17328	7295	22170
MDM1	24	8507	2265	8036	6771	10523	5253	14277
FAM71B	24	30521	8331	28679	25712	34035	17726	59071
ACTL9	23	10941	3843	10105	8511	12899	3849	19296
NT5C	24	29115	15517	23926	17029	42066	7217	58544
PNPT1	24	41444	10953	40638	34251	46584	25626	78056
AGR3	23	58460	94874	30634	11252	48415	993	370413
NEK9	24	9872	2687	9960	7781	12264	5463	16413
ZNF675	24	54156	8872	52635	50282	57791	40220	80997

PG.Genes	n	mean	sd	median	q1	q3	min	max
GPT2	24	9584	8743	6959	5560	9584	2789	40612
PLEKHO2	24	22046	7915	19965	17016	26001	11873	42057
MAGEE2	24	13439	7546	11063	7645	17570	5314	33038
ACTRT1	24	75748	39288	61785	49796	87801	27188	171422
BPIFB1	24	86868	24570	84995	69337	96748	43475	161808
BRIX1	22	9995	4438	9266	7262	12129	4191	18998
RNASEH2C	24	84089	47783	71498	53846	97062	24068	210168
GNPDA2	24	16850	6995	15798	11600	21860	5240	34227
FAT3	24	179365	24169	173872	162335	186962	152040	238980
IGDCC4	24	22377	6092	20676	18644	24844	13044	39478
MICAL1	24	137994	45152	128605	104887	162097	77158	232560
PANK1	24	8742	2113	8636	7741	10301	4785	12799
TAS1R2	24	25719	7512	25604	18990	32388	14262	37701
ADAMTS17	24	175850	53106	167397	142856	195500	87749	300712
ADAMTS15	24	197990	55357	183494	163267	215288	129328	347859
EPS8L1	24	38440	11843	37429	28227	45772	21807	64794
DNAH5	24	137392	24314	130093	122581	147654	91304	204220
SH3TC1	24	11046	2665	10806	9684	11465	7843	18441
NSUN6	24	119213	24620	115169	101215	132582	84359	188252
TBCK	24	10101	2990	9733	7985	12049	3615	15617
DTD1	24	9002	4169	8558	6038	10728	3005	20181
IPO4	24	35017	9263	33505	28728	40768	18808	60111
PNISR	24	36851	19636	34196	24281	40438	15593	102738
DYNLRB2	22	4534	1937	4256	3431	4965	2350	10573
WHAMM	24	13231	3154	13084	11343	14628	7311	20189
ZNF483	24	49450	10697	48670	39605	59106	32354	67187

PG.Genes	n	mean	sd	median	q1	q3	min	max
FNIP1	24	19707	3439	20112	16855	22284	13859	27186
SHROOM3	24	76686	15113	73940	66319	85388	51570	107117
SETD7	24	6190	4891	5074	3784	6481	1437	23866
DDI1	24	21704	9635	20874	16851	23207	7914	60119
STK32A	24	6099	2557	5603	4656	6701	3170	15425
C7orf33	24	8630	3387	8586	6261	10307	3669	17622
SCFD2	24	191671	32341	196999	166461	203524	149030	263254
ZC3H15	24	132726	51632	123321	103404	139379	69108	318653
PPIL4	24	67910	28199	59373	52916	77605	22355	143753
PPP1R13L	24	26827	4725	25857	22897	30139	19360	35889
TGFBRAP1	24	20255	5202	18891	16890	23541	11858	35304
NUP133	24	90344	44125	76211	65157	95455	43320	220649
PDCD6IP	24	93504	27731	97384	73907	107423	35426	149344
FBLIM1	24	63809	40201	53482	37293	85916	10961	167762
SDR42E1	24	221661	97221	204885	154793	281665	67406	499742
BRK1	21	3146	1095	2999	2376	4003	1212	6091
DEPDC1B	24	35650	13131	31728	24884	43978	20512	67884
LTO1	24	18291	11783	16137	9069	21092	5114	51699
PTCD2	24	1548791	554100	1489259	1246858	1833900	340186	2696936
AFG1L	24	12874	7039	10400	8122	15894	6096	35247
LEO1	24	423171	101151	412317	374794	435142	247953	694040
OSCP1	24	50124	19711	44007	39800	53072	19026	92214
NUDCD2	24	7064	4430	6411	4523	7932	694	21915
SCFD1	24	11509	3907	11124	8694	13326	6040	22188
C4orf3	24	23922	9579	22615	17291	28388	12805	54305
UBLCP1	24	15301	6496	13323	10493	18893	7454	28585

PG.Genes	n	mean	sd	median	q1	q3	min	max
TEKT4	24	39429	15511	36195	31634	45228	20848	95403
ZFPM2	24	45742	19386	38952	33849	48516	21014	93751
SPRYD4	23	4430	1938	3982	3083	4787	2094	8762
CAPSL	23	8467	2265	7751	6623	9565	5307	13011
CYGB	24	7938	5740	5936	4172	11383	1797	22380
PHIP	24	39230	6856	38453	33752	43362	26859	53142
SELENOM	23	5000	2328	4097	3371	6460	2339	10758
PRPF31	24	4931	2399	4139	3243	6198	1672	10149
PALLD	24	44032	54967	27791	22481	35115	16493	281421
SREK1	24	12534	4552	12249	10055	14391	5428	24184
METTL21A	19	2034	847	1996	1465	2531	529	3971
COPS9	24	6298	2065	6323	4389	8074	3626	9488
SCG3	24	15723	3598	15483	13715	17211	10269	24030
SRSF12	24	70477	28921	60739	53929	85598	32512	149930
PSPC1	24	15119	7172	14748	9158	17937	4789	32620
RSAD2	24	41052	15197	40355	32670	50246	18322	83441
JPH3	24	37040	9849	36353	31804	39764	24988	64327
MUC16	24	124944	18268	119105	111184	133959	102468	163252
DNAJC9	24	12532	7077	10209	8498	16777	2840	34721
THAP4	24	128201	31909	124553	108111	141315	88293	229829
TTN	24	1296064	159840	1230992	1209682	1362958	1096759	1750738
OVCA2	24	1685652	497153	1701142	1448132	2033352	702473	2751160
LZIC	24	19549	6243	17892	15920	22360	11228	39652
IRGQ	24	14979	5345	15106	11624	18263	5413	24903
ST8SIA2	24	5511	2941	4362	3574	6721	2295	12936
DDB2	24	96819	24037	93034	84730	109121	45949	158892

PG.Genes	n	mean	sd	median	q1	q3	min	max
SMPDL3B	24	3916	911	3856	3380	4564	2478	6078
DDX1	24	53709	34364	41329	30422	64035	8202	136669
HSD17B8	17	3729	1994	3357	2468	4123	1595	9759
PIEZO1	24	12859	2095	12267	11716	14376	9790	18580
FAM3C	24	13408	8625	10218	8785	13622	2967	37296
H1-10	24	33460	54496	19176	16135	29860	11259	283824
PSMF1	24	17501	5542	17499	12776	21438	9698	29115
GBF1	24	125614	23892	121363	114961	139740	85396	201673
NCSTN	24	42365	18272	42165	26294	52118	16704	97102
MRPS27	24	36244	12488	32592	29771	41330	16506	70104
ELMO1	24	29058	4862	29514	27479	31268	15900	38226
FIG4	24	33395	5935	32529	30419	35331	24916	54854
DCUN1D4	24	5179	1536	5381	4049	6510	2518	7644
AP3S1	24	11360	3689	11300	9037	12447	4660	22063
UBXN4	24	8796	2680	8310	6537	10827	4429	14820
CNOT9	19	1873	1072	1486	1031	2626	532	4710
DOCK2	24	43759	5810	41813	40190	47383	32294	55798
TBC1D5	24	10627	2189	10200	9181	11510	7778	15614
LARP4B	24	7430	2537	6345	5949	8245	3819	13800
GCN1	24	65454	7209	63825	60126	70402	54865	84912
ARHGAP45	24	16587	8248	14462	12869	16923	10661	52285
DHX38	24	311481	89815	298229	251974	345444	195058	558544
NUP205	24	20210	4488	19295	17415	22595	13202	30906
TTC9	24	22114	12949	20229	15860	23115	7558	69458
PXDN	24	74028	22026	67247	59518	88969	40046	130334
PIGK	24	40478	11133	40674	32404	48872	22061	68421

PG.Genes	n	mean	sd	median	q1	q3	min	max
GTF3A	24	21606	19681	16096	9882	23373	4137	93411
SORL1	24	32151	7558	31981	28436	34792	16591	53190
ANP32B	24	24994	11159	23076	17706	28836	9550	51228
RABGGTA	24	17174	6418	16071	13734	19585	6951	40429
USP6NL	24	27282	5758	26371	24113	29370	18131	41932
HTRA1	24	8423	12162	6076	4159	8129	2880	64755
ARPC1A	24	9117	3756	8834	5921	10927	3931	18017
ARPC1A	24	18813	7333	18102	12621	23062	8610	36666
TAF4B	24	36558	9694	34652	30409	40567	21244	63597
HDAC2	24	98715	33251	86573	73163	122220	53617	155754
DPF1	24	45206	18273	41584	32359	53790	21484	87793
STAM	24	27379	11169	26810	18847	33942	9779	55520
PROX1	24	67141	15141	67771	59023	77095	38072	101299
SYMPK	24	4512	1280	4382	3470	5326	2831	8088
SYMPK	24	10378	4596	8725	7449	11293	4858	23330
TAF15	24	21449	4564	20702	19004	24309	10836	31645
GGH	24	33145	26482	27756	13109	47131	3216	120781
DDX17	23	13066	9079	10304	7400	16059	3103	41783
NEO1	24	8490	2222	7845	6905	9521	5826	13764
APBB2	24	46850	10769	46347	40068	52581	29780	73499
OSTF1	24	46144	20036	41019	31359	55912	19608	102713
ABCC2	24	23435	4262	23035	19939	25665	17455	34002
ERCC4	24	45434	10149	45883	37200	50430	28857	75264
UFD1	24	13237	4753	13821	9326	16534	5259	22275
RPL3L	21	5356	2875	4690	3949	5941	1471	15281
COPS5	24	10355	3316	10249	8538	12288	4942	19058

PG.Genes	n	mean	sd	median	q1	q3	min	max
GPKOW	24	35679	7219	34989	32234	38249	26597	62784
SMARCC1	24	3734	841	3674	2881	4236	2583	5595
RAB8B	24	24227	9060	22259	18375	26771	14788	56231
BAD	24	92422	63158	71906	62132	101000	52001	362291
KHSRP	24	83403	37306	83919	54579	110316	14520	150050
KCNB2	24	13445	5471	13193	9234	17406	4402	24459
GLMN	24	7428	2799	7914	5719	9140	1052	12536
DVL3	24	150988	70638	148804	109778	185117	33483	373483
USP9X	24	11144	3597	10895	8758	12720	6233	24237
USP7	24	25164	9217	23296	18331	30322	11118	48447
CUL5	24	78727	26314	76436	56752	100109	37808	139346
LPP	24	35294	71384	13874	10722	18452	7237	270540
RBPMS	24	8599	5960	5940	4950	10117	1884	24372
HGD	24	13902	11354	8999	5610	22540	1531	40673
MR1	24	104527	50783	91806	71379	128968	47048	264414
TCEAL3	24	10988	8106	8794	6975	10656	3946	37013
TSR2	21	2145	834	1892	1475	2621	929	3983
CAVIN3	24	114412	65545	90992	70584	119252	52798	293747
NKD1	24	16323	10205	14540	8738	21049	2645	44097
CNKSR1	24	25596	6469	25906	20776	29485	12383	38703
MYDGF	24	35222	18961	27294	19474	54796	13474	75589
OSBP2	24	18689	6425	17934	13325	21868	11019	35311
WBP2	24	10151	2471	10187	8147	11491	5767	15686
NCLN	24	3189	1402	3308	2276	3677	635	5914
NXPE3	24	52769	32511	46894	31062	55572	17677	150438
MYLPF	24	89731	24336	88510	73083	102258	46351	139913

PG.Genes	n	mean	sd	median	q1	q3	min	max
SYAP1	23	38512	21908	33027	24209	43301	14122	92338
EXOC4	24	17782	4164	16907	14414	20882	9660	26487
NTAN1	24	39465	9917	38630	32559	41801	26754	69167
FUBP1	24	14483	15549	10157	7334	14110	4094	72936
FUBP1	24	62043	27570	64358	37984	78472	14557	109564
TTC17	24	31974	8315	30236	26384	34792	22159	52275
LRRC59	24	15123	6654	13983	11053	19028	3340	29030
CLUAP1	24	52177	30672	46116	28048	72352	17441	136196
ESAM	24	2298	1110	2322	1313	2785	646	4680
FKBP10	24	124542	34632	124516	101222	143815	56095	192754
AKT1S1	24	5054	2721	4146	3418	5803	2184	11911
ZNF428	23	9816	4409	10859	5268	13957	3696	16357
TMEM186	23	21211	18425	13240	9395	22553	3469	67923
TMIGD2	24	179203	54987	163034	147085	190409	97864	299186
RNF25	24	8419	2496	7745	6692	9826	5500	14901
AIDA	24	30555	19406	26743	19093	33384	11563	94780
ARL8A	24	15763	8834	13480	11244	16827	6621	50400
OTULIN	24	23401	8710	21771	18538	26672	9789	53149
CHCHD1	19	4322	3748	2781	1438	5638	379	12621
PPWD1	24	10314	2581	9858	8663	11487	6158	18449
COA7	24	106492	37764	98646	81207	119388	67727	223537
PTER	24	11300	7199	11089	6725	13372	2205	33787
MOB3A	24	19071	5538	17275	15982	20105	10743	35449
LENG1	24	27400	6842	27894	23121	32922	11053	39123
FAM136A	24	12111	7757	9521	6336	15795	2281	27691
MIEF2	24	8881	3246	8044	7103	10025	3350	17786

PG.Genes	n	mean	sd	median	q1	q3	min	max
DHX58	24	23466	4398	23029	20519	26905	14104	30587
EFHD2	24	61744	31825	48664	42937	73284	29967	165202
GALM	24	57686	38032	43880	29010	85527	13023	144149
SYTL4	24	129750	22782	123476	117644	137286	88645	203341
ULK4	24	107952	18497	104336	95349	117205	72009	143977
DCPS	24	15597	6256	15307	10732	17414	7490	37233
PPP1R14B	24	21372	10978	18864	13767	26998	7064	51925
FBXL8	18	3363	1161	3271	2739	4360	1611	5507
PPCDC	24	39184	13460	39178	27734	48890	20113	65831
ZNF653	24	27725	9906	27290	20812	33750	11506	54577
NXNL1	24	38848	29890	28484	24316	34204	16846	140001
ISOC1	24	52615	28623	47303	35855	59406	13974	143448
GCC1	24	54996	7933	55862	50125	60351	39958	70131
FLYWCH2	24	12014	5441	11828	7984	15321	2199	21837
FAF2	24	22910	13345	18865	14533	27722	4740	63518
CCDC124	24	33555	12767	33066	26897	37141	14942	69863
AP2M1	24	13203	4302	12773	10800	16053	4717	21502
KCTD12	24	24655	22889	16315	9932	33331	7229	98429
RCN3	24	21407	7202	19443	17608	24229	14046	46729
COQ8B	24	157628	41649	152868	136608	180368	72193	247638
ZG16B	23	8382	5046	6420	5429	10815	1881	22401
RMDN1	24	7416	4010	5517	4746	9209	3457	16071
LRRC39	24	34679	13355	31511	23233	43773	15813	60190
CMBL	24	43290	19071	39795	32678	49692	13368	95708
SNRNP40	24	7771	3574	6096	4986	10535	3406	15725
ARHGEF26	24	32131	12934	30086	25656	36670	6204	61646

PG.Genes	n	mean	sd	median	q1	q3	min	max
ATG4C	24	16132	4353	15564	14290	18247	3681	24952
ITPKC	24	22101	7276	21264	18157	26455	8592	39076
IQCD	24	70408	12735	69950	63606	77433	41184	95749
RBMXL1	24	346536	163436	298061	230435	446435	127336	826457
SIRT1	24	213574	53302	204497	182654	241392	130975	350478
HOOK2	24	19376	4385	18595	16845	21025	13532	32715
SEH1L	24	4345441	3755471	3528287	2780814	4359463	1612500	20680148
TCEAL4	24	12911	4401	11592	10913	14609	7041	27168
GNPNAT1	24	29848	21569	28303	12019	40630	3711	88753
INKA1	24	9580	5581	8358	5850	12739	720	23093
MRPL53	24	5877	4125	4731	3506	6347	1680	21114
L3HYPDH	24	27022	10462	24358	20066	31275	11543	61731
MOCOS	24	20175	10906	16624	12475	26482	6420	45782
DAZAP1	24	30300	17161	26662	20307	36940	8346	74274
SAAL1	24	12239	7684	9278	8185	11290	4306	32661
CCDC51	24	7607	2817	6790	5899	7712	5174	15801
RBM33	22	2056	1472	1901	996	2523	414	6832
MMAB	22	14405	23892	8171	5955	12142	3092	119037
ADAT3	23	5308	5468	3790	2167	5754	1860	24333
SAT2	24	8735	5869	6995	5045	9941	2818	29040
CCDC97	24	112432	58593	104288	74330	139138	34829	242871
DISP1	24	48263	11823	43891	41367	49653	32905	76880
CNRIP1	24	6211	10394	2969	2255	4458	1673	48118
PHYHIPL	20	4848	2864	4244	2510	6568	1154	12570
DYNLL2	24	6706	4029	5436	4717	7297	2961	22211
WDR89	18	3126	925	3296	2699	3753	1380	4751

PG.Genes	n	mean	sd	median	q1	q3	min	max
DTD2	24	58739	22085	57310	48008	64369	17087	132732
S100A16	24	13625	8961	11149	9172	14122	5434	45629
SIPA1	18	1654	954	1390	1044	2106	387	3390
LRRC46	21	4476	1829	4187	3021	5069	2199	9955
THOC1	24	19866	3405	20288	17726	21467	12151	26191
OTUB1	24	31540	11227	31578	24441	35437	16805	71467
TRMT61A	20	4441	2104	4187	3143	6362	1121	8270
HMCES	24	6313	2142	6026	5171	6847	3715	14574
PGM2	24	69288	29881	59119	53374	73516	40951	171874
KLHDC7B	24	4955	2220	4688	3504	5702	2105	12203
DUS3L	24	11081	3215	11003	8742	12588	6025	18138
SDSL	16	3193	1940	2921	1925	3834	984	9325
PDXP	24	4093	1035	3933	3316	4897	2385	5869
DCUN1D1	24	43517	12686	41675	35452	45481	24947	76441
FAHD2A	24	4322	2076	3506	2995	5262	2134	9203
APIP	24	11725	4389	11619	8839	12880	4664	22563
ZC2HC1A	24	13100	6158	11279	8834	15468	3540	30033
SNF8	24	23539	3445	23615	21197	25843	17865	32170
ZC3HAV1L	21	4541	3159	4017	2532	5188	1519	15547
PDLIM5	23	10445	8463	8893	5194	11660	1429	34790
PDLIM5	24	20656	24463	12736	10738	17515	3126	114056
PDLIM5	24	38243	61053	17418	12511	39605	2385	303568
ACY3	23	17038	15623	13102	8560	20370	5869	80412
ERO1A	24	35502	34671	32110	18313	35686	2395	170220
PRR11	24	6220	1134	6068	5474	6861	4403	8321
FMC1	24	79956	27976	76987	57178	95846	44449	152079

PG.Genes	n	mean	sd	median	q1	q3	min	max
OXNAD1	24	60235	20347	58586	50284	66259	18979	117668
DIRAS2	24	8834	3591	8022	6924	9346	4675	19774
INTS4	24	158658	47574	147739	128102	183779	81793	256434
DDRGK1	24	36610	38359	27023	14018	41399	5108	168881
SCLY	24	13109	4443	12697	9665	14854	7707	23245
FUBP3	24	15869	5135	14702	13157	17692	8987	32240
RBM17	24	13821	4657	13065	10076	17450	7459	25177
NARS2	24	59914	16565	56552	47659	66437	37370	93926
GMPPA	24	14151	7550	14207	9057	19783	1835	31301
ABHD14B	24	62728	41195	49688	39570	75647	25605	217393
NGLY1	23	93733	41222	90313	63921	120714	14534	180883
FAXDC2	24	18769	6971	17775	13873	23387	8459	37431
CPB2	24	21599	13192	17300	13345	24282	10810	68837
PAWR	24	19462	13631	15174	11954	18677	4915	60699
THOC3	24	36607	11262	34297	30049	42710	12746	61544
COG3	24	434722	166230	402002	323894	475922	192280	919528
HIC2	24	13759	3123	13914	11235	14984	7497	21493
ZNF333	24	49038	10808	48076	40694	57617	34339	73898
CHAMP1	24	24465	3572	23687	22583	26795	15678	32100
MYO15B	24	34153	6379	34959	28139	39073	22985	47161
DCHS1	24	127957	19980	128984	109739	138607	97266	166845
CLMN	24	27783	11221	24794	20668	30352	16740	64012
PDLIM2	24	26951	9758	26665	21036	36036	6848	45370
BTF3L4	24	14278	6969	12038	10396	16462	2744	34093
BTF3L4	24	18618	5806	18866	14279	21578	7666	37365
DNAJC1	24	17867	6976	15402	13027	20807	8424	38508

PG.Genes	n	mean	sd	median	q1	q3	min	max
CABS1	23	11869	3763	11457	10288	12465	4743	21508
BTBD6	24	12148	10087	9240	6819	13274	4083	52363
ZNF512B	24	64940	15412	63596	53677	70400	38317	102689
LRATD2	24	10454	1736	10420	9059	11510	7954	15158
CNDP1	24	106659	35961	97246	82336	125011	53746	206617
RPGRIP1	24	53069	12990	48535	43260	61693	38016	83689
EXOC2	24	11862	3013	11093	9878	13843	7934	17593
CNDP2	24	272332	126087	266243	199033	360574	60466	531193
ZFR	24	11515	2730	10822	9658	12769	7476	18309
WDR90	24	10308	4129	10016	7637	11275	5266	25897
CAPZA3	24	20257	6187	21252	16944	24161	2458	30521
KCNH8	24	3434986	938663	3213510	2964320	3760140	2073656	6299471
CAPNS2	24	137567	23461	134636	122032	149978	102102	188703
FCRL2	24	11042	5587	8892	8187	11760	4920	30346
PRMT6	24	12809	5681	11260	9234	15502	4709	26465
TRIM47	24	11120	3965	10588	8772	12490	5208	24547
SENP8	23	3505	1441	3304	2426	4622	944	6209
CXorf58	24	17331	4381	16605	13899	21521	10978	26867
C4orf45	21	3281	2111	3022	2048	3692	802	8693
C12orf42	24	6292	1615	6394	4975	7585	3487	9490
PPP3R2	24	15663	11143	13594	11559	15878	5488	61679
TEX55	24	30271	18272	27869	17083	33769	5686	67978
TBATA	24	16540	6277	15988	11978	18679	8438	34345
NA	24	17332	5548	16955	13799	20041	8642	31139
CCDC7	24	108356	16593	101052	99055	114600	84273	145187
DRC1	24	17461	4384	17447	13526	20402	11096	27543

PG.Genes	n	mean	sd	median	q1	q3	min	max
MFSD14A	24	49248	50761	35766	19372	68015	3517	243129
C5orf34	24	5649	4850	4613	4053	5212	2534	27557
COG8	24	19806	4758	19410	16926	21241	12142	31055
TTC14	24	78717	26435	72187	63068	92917	42181	139300
PWWP2A	24	60852	11349	58703	55431	65094	43762	86648
RILP	24	10041	5156	10128	6208	11744	3590	25260
SCLT1	24	70283	13787	69700	62600	74196	42868	114773
WWC2-AS2	24	12498	8631	9578	6973	15910	3241	44435
SLC46A1	24	10575	5615	9269	6757	12248	4625	28576
CLIC6	24	16963	4429	17631	12718	20390	10490	25462
FOXN4	24	8071	2284	7877	6864	9110	4294	13999
IPO9	24	20190	13689	14571	11283	25652	3946	51831
ARHGEF17	24	109859	34707	96371	87306	123414	74019	189258
MS4A10	24	5810172	3638258	4548096	3528142	7387192	1201774	18092310
RBM14	24	37473	14548	37225	28297	46902	14197	68293
ADCY10	24	20238	3319	20319	18259	22690	13901	27316
GBP5	24	25359	7321	24814	20213	28843	12907	41377
GBP4	24	98817	29377	91092	78141	106068	67808	177130
FANCD2OS	24	161568	115755	127543	86958	181752	58974	496141
PSMG3-AS1	24	10332	3699	10077	8381	12116	3894	18052
LMTK3	24	17686	2994	17690	16257	19120	11899	24997
TRNT1	24	27481	8902	29504	20451	32633	12451	46271
FLACC1	24	91203	43412	74354	63224	98351	42461	193601
TMEM237	24	25202	8564	25833	19363	31114	9416	41476
GSDMA	24	23916	4757	23509	21190	28264	14895	31235
PPP1R10	24	9684	1280	9483	8707	10237	8100	13042

PG.Genes	n	mean	sd	median	q1	q3	min	max
PRAM1	24	10891	5897	9157	7281	10956	4836	28487
VPS35	24	54811	15673	54763	47793	64770	20462	83279
PURB	24	5228	2497	4783	3223	6954	1906	10594
PSKH2	24	11071	3798	10629	8174	12856	4798	20006
PHF12	24	64677	17078	60939	54315	70795	47201	122309
RBP7	24	746172	313063	726498	482384	941755	58734	1516169
PANX2	24	26131	5569	24796	23347	27151	15711	40871
NACC1	24	185064	81051	162625	144218	199115	102392	498926
ZNF300	24	49847	16810	44784	39983	57340	19196	91164
SNX18	24	269134	114621	245338	194746	306541	135016	578723
CIC	24	56231	9938	55961	50933	60351	42383	91876
VPS13A	24	74345	24303	65109	58076	87936	49033	150987
METTL26	24	25697	8633	23918	20203	30124	11786	44832
TP53RK	24	11023	4202	10182	7459	13639	2224	19772
HAPLN3	24	124858	47570	118962	88732	142502	39778	238305
PLEKHF1	24	22634	11866	19119	14841	27499	9504	65078
PPP1R9B	24	21574	5184	21245	17891	25420	12369	32173
SRPK1	24	34146	7801	33103	28268	40566	23487	50716
CPXM1	24	4289	1660	4047	3147	4734	2284	8932
CYP2S1	24	35450	57313	17832	12477	26435	2774	273251
IWS1	24	28576	6865	28459	23010	33684	15785	41045
SIN3A	24	37032	3786	37158	34507	39041	29513	44313
ADO	24	8258	3242	8121	5808	10909	3402	15160
RUFY1	24	161389	19604	164001	149088	174964	115807	191807
PNKP	24	4320	1513	4554	3539	5119	1265	7441
NIBAN2	24	43717	17555	40540	33738	50439	14378	87262

PG.Genes	n	mean	sd	median	q1	q3	min	max
RMDN3	24	31261	18216	26672	20088	33019	8221	77389
TCF12	24	115174	46006	107516	84662	139522	47287	238502
GAD1	24	11002	2866	10323	9050	12955	6008	17493
MYCBP	24	8077	4084	7384	5329	9186	1685	18926
TBCB	24	106530	56974	89773	70204	119058	50611	280102
PSMB7	24	23611	9460	22405	18318	28620	8652	39624
CNN2	24	23372	11730	18531	14943	32003	8616	47672
PCYT2	24	5719	2815	4736	3711	7108	2247	13366
CDC5L	24	40058	9660	38043	33042	45198	26367	62194
PSMD1	24	15876	10979	13119	8601	21047	3349	56233
PFDN5	24	51759	14670	50204	40577	62255	18645	78408
PARK7	24	257852	96142	242818	196680	300874	94274	521089
SORT1	24	19784	7875	17578	15305	21049	10897	47248
VAT1	24	63803	51301	46417	31958	66686	27805	248978
NUP88	24	4409337	1730803	3972965	3345624	4677082	2386985	9792115
PKP4	24	9449	4055	8310	7188	10095	5358	24280
POP1	24	46953	16395	43094	37638	51447	23325	105551
S100A13	24	60832	41323	47734	29107	94234	7175	181654
SCAF11	24	27945	6946	26022	23848	31611	16882	43765
TSNAX	24	12127	5317	10918	7517	14127	5998	22287
SEPHS2	24	93303	35785	80533	71003	113591	43083	175938
TTC1	24	7436	3401	6756	4886	9331	2832	17196
DNAJC7	24	44840	12214	44286	38000	50465	22464	68003
C12orf57	22	3840	2045	3389	2698	4076	1424	9469
COPS8	24	17776	3975	17413	15469	20538	11227	27127
CHP1	24	21399	8308	21356	15527	26439	5798	38921

PG.Genes	n	mean	sd	median	q1	q3	min	max
MAP3K5	24	85364	17188	84906	73052	94062	58638	133069
MGLL	22	2723	1927	2018	1586	2790	619	8192
KIR2DL4	24	25875	6541	26339	21432	28884	14101	39862
HSD17B10	24	13876	9814	12693	7251	17616	2251	49983
SEPTIN5	24	6937	2367	6184	5342	8604	3739	13243
CCL19	19	13034	15647	7856	5836	11770	4237	72614
NAP1L4	24	37683	9675	37909	32202	41671	20177	56689
NPAS1	24	19048	9921	16888	12462	23093	7210	44691
NAPG	24	16154	5049	15286	12519	18378	9015	29668
TXN2	24	5928	1719	5635	4728	6816	3313	10333
ABCA3	24	9794	1858	9470	8620	10436	7373	16594
MIPEP	24	42372	5211	43582	39317	44805	26151	49767
ACO2	24	52095	31063	42485	33403	63469	14283	143630
TM9SF2	24	36878	13339	35257	25653	49740	17236	57661
TSG101	24	20900	8559	19682	15111	24581	9088	43229
CPNE1	24	25205	12738	23235	17016	35590	3019	49431
CCT7	24	43912	18300	40872	35802	50966	14566	96856
SMO	24	31389	4268	31102	28729	33823	21200	39081
EBNA1BP2	24	29959	16500	25422	17594	38507	5937	65276
PKP2	24	65302	19079	60417	50677	74657	45198	130413
SH3GL1	24	22507	8060	21705	16623	28158	9199	43770
TEP1	24	28871	8174	27436	24476	33228	9359	47182
OMD	24	45168	15835	41806	35089	50001	29237	105277
SEMA3C	24	13992	6409	12567	9747	17433	3681	32496
VRK1	24	23954	13251	23171	14483	28150	8773	69940
GDF15	24	76182	35931	62231	55796	88578	38327	189827

PG.Genes	n	mean	sd	median	q1	q3	min	max
DPYSL5	24	13956	3192	12948	11709	15554	10213	22709
NIPSNAP1	24	13631	15130	9770	7313	12587	3078	79440
HSD17B14	24	15088	13307	11994	10852	13769	9350	76581
ARPC5L	24	15209	7230	13956	10706	19253	4098	31625
FAM118B	24	12652	4468	12046	10145	15808	5098	23977
KCTD14	24	96003	33549	92674	74291	111843	38203	173858
TRIR	24	15267	4607	14612	12152	18654	7797	26382
MACROD1	24	361329	142939	326848	270614	430165	170791	834928
KLHDC3	24	99948	42434	89057	72537	121744	34402	196085
WDR77	24	18936	9046	17688	13516	22994	7503	46657
KXD1	24	84114	54125	64724	44702	104107	24785	203615
ANTKMT	24	16848	6579	16182	10222	22796	8270	31240
TUBA1C	24	10338	6481	9836	4831	15018	1464	27030
APOL2	24	18794	10474	15207	13691	21274	9342	57363
PSD2	24	165510	50925	153123	135864	199087	77004	251564
NRIP2	21	28412	22434	18927	16505	28789	3224	90652
HEPH	24	31828	6093	31022	27310	36334	22270	46409
ACBD6	24	35346	44836	21800	15247	34790	7417	230788
CORO1B	24	78873	27975	77159	63004	85284	29359	168253
CCDC77	24	90785	29950	86150	76399	109319	37513	145166
NAA38	22	2755	1323	2548	1802	3370	1087	6891
TXNDC17	24	62954	22216	65700	47608	78738	21463	106356
PLCD4	24	12169	5907	11830	7195	16547	3315	23178
CPPED1	24	21764	10704	21021	13569	28320	7468	53160
VPS25	24	5818	2005	5852	4188	6689	2510	10897
NUDT16L1	21	3897	5203	2502	1435	4545	396	25170

PG.Genes	n	mean	sd	median	q1	q3	min	max
ERP44	24	312435	221845	231709	198315	343132	73468	1014330
LXN	24	29258	12726	27920	22326	31359	11316	69341
NTPCR	24	11769	3850	11766	8772	14740	5290	19528
HDHD3	24	3901	2341	3314	2274	5286	376	10407
TUBGCP2	16	5359	12194	2273	1198	3239	715	50830
ESYT1	24	21140	11123	16237	14214	23963	11929	60923
UBAC1	24	5921	4087	4857	3104	7162	1649	15917
CNPY3	24	5498	2903	4857	3597	6386	1447	13989
PSMG3	24	9066	3106	8739	6645	10941	4385	17460
COPS4	24	18093	5751	16206	14371	21355	9639	33706
WAC	24	25722	7862	24714	18775	30678	15069	42908
DIDO1	24	72438	10994	71169	64751	78479	54693	105222
RAMAC	19	4604	2117	4434	2915	6521	1485	7804
FUCA2	24	3111	1664	2627	2048	3770	1042	7399
HGH1	24	63812	13485	66478	53878	74221	21916	80372
THTPA	24	48304	15931	44742	40476	55258	25981	100889
DOHH	24	12100	4800	11350	9055	13056	4883	23895
SPINDOC	24	19686	3925	19436	16440	21052	14256	30337
TUBB6	24	33637	19803	29086	25300	33517	16397	119759
PAXX	24	23252	11782	22976	14487	28530	5673	53349
HNRNPUL1	24	45874	26645	40972	28419	53993	15454	136264
PDCD10	24	15850	4002	16438	14425	17949	8204	24039
EFHD1	24	20687	7949	18444	14798	25202	11085	43444
DDX23	24	75896	18819	70178	62181	84348	52924	123796
BDH2	24	50587	21824	46949	37687	63009	12715	93450
BDH2	24	107747	102023	73253	37641	147896	20185	428393

PG.Genes	n	mean	sd	median	q1	q3	min	max
ALG12	24	20036	7640	19950	14950	23095	9436	42349
C1orf50	21	3944	1870	3538	2365	5325	1113	7801
MRI1	24	29544	12713	24230	21277	34525	12525	61114
CCDC32	18	1897	1759	1604	907	1946	177	6509
THUMPD3	24	17717	7345	16580	11975	23041	5604	32650
ADI1	24	14522	9930	10874	7925	19639	1665	38348
RNF126	24	235316	96246	231149	179496	259526	111773	497509
KATNB1	24	13603	3344	13962	11397	14797	7162	23040
TMEM109	24	4279	3728	3075	1905	4610	572	14731
PBDC1	24	19592	7322	18532	16145	22699	5215	37753
DUSP23	24	6544	3569	5924	4180	7352	2360	15333
NUP58	24	14522	3540	15077	11104	16717	8358	23440
SELENOO	24	10936	3811	10281	8489	12516	5688	20814
DPCD	24	33752	13764	29576	26867	35833	21028	89965
GNL3	24	32897	8624	32289	29048	36813	19068	63296
TPPP3	24	83080	115275	37116	18435	81353	9196	482061
HIRIP3	24	223570	67305	213789	193604	253718	91508	337281
RBM4	24	10034	5217	8100	7197	11151	4930	27874
SF3B5	21	2601	1836	2438	1363	3328	237	7356
PRR14	24	4459	1723	4049	3320	5007	2113	10228
SSBP3	20	3584	1661	3384	2500	4359	1170	7539
SCRT1	24	35195	6622	34459	29144	38402	26404	48939
SYCP2	24	294434	43514	291047	267091	316485	221836	389373
LSM14B	21	3276	1915	3047	2242	3617	1005	10280
SORBS1	24	12048	4235	10704	9146	13066	8235	27111
HINT2	22	6580	5972	4798	2836	9409	1545	28550

PG.Genes	n	mean	sd	median	q1	q3	min	max
ZNF471	24	287092	95533	264097	245408	305811	52096	511363
PLVAP	24	25385	5739	24789	22330	29451	16921	40214
SPATA16	24	11151	3040	10780	9322	12178	6531	19027
RAB11FIP5	24	8053	4923	6171	5404	9440	2638	20601
C1QTNF5	24	48842	11377	47576	41656	55359	31683	73809
TMEM120A	24	70122	17868	73451	55521	82414	42308	113083
NAA15	24	20353	5376	20383	17125	22395	11994	34772
CDCA4	24	23584	6098	21769	19860	26826	15010	39644
PAPPA2	24	117930	54423	103927	82632	145221	42954	253677
QTRT1	24	7465	2311	6993	5910	8710	4394	14595
CFHR5	24	242701	134201	236834	138727	281058	81691	709806
CACNG6	24	13847	5990	13192	9982	16268	5156	32048
TEX12	24	56923	29635	53857	41170	67473	2996	128680
GON7	24	3069	1598	3370	1767	4055	615	6188
OSBPL1A	24	71599	20671	69121	59623	78160	41842	142395
EMILIN2	24	15531	3828	14929	13108	18375	8746	21740
ANKRD30A	24	55828	10289	51977	49368	63070	35888	74813
PACSIN1	24	74992	22379	67938	58903	86781	41950	134524
GPR87	23	23084	15931	18276	14120	30485	3234	76764
ITPA	24	14231	7232	12876	8756	21829	4136	26798
EIF2A	24	33504	18596	30177	19811	39672	12975	86809
CADM1	24	119642	55715	100395	77319	167651	49224	236408
POLDIP3	24	19405	3993	19504	17368	21598	9340	28746
GNB1L	15	1540	897	1469	809	1740	503	3488
PCGF6	24	12312	7483	10169	8378	14689	2216	39613
YTHDF1	24	9179	2478	9199	6874	10998	5206	14191

PG.Genes	n	mean	sd	median	q1	q3	min	max
STK33	24	18726	11288	15735	11942	22516	5794	51043
NLN	24	16142	5534	15453	10906	21450	8906	25905
CEP41	24	9842	5181	8681	7634	9670	3375	29996
POTEKP	24	240172	95170	214822	175207	283522	132301	522796
NUF2	24	23498	6496	22420	19212	27650	11380	37990
ASPSCR1	24	195220	55266	186641	159460	212694	116508	342802
DPH1	24	59786	14238	60825	48213	66684	39581	94841
IRX2	24	10112	6309	9081	6415	11445	1687	28433
UPF3B	24	104950	47479	94873	80632	130316	30379	243113
TBL1XR1	24	12163	7357	10093	7030	15910	3312	32411
UBL5	23	7069	6052	6563	2872	8330	807	27320
GSX2	24	325805	114664	323153	266280	384400	41369	566690
NIBAN1	24	16597	11511	11313	9313	21126	5385	52819
UBXN6	24	22110	10305	20498	14635	23288	8831	46407
TM6SF2	23	11436	6360	10846	7564	12555	4630	36218
DPY30	24	18862	11144	13592	10744	28845	6931	42305
TRIM2	24	9579	4564	9510	6079	10769	3332	22803
SPEF2	24	50051	9376	47998	43164	54391	36110	71052
FTO	24	23617	7760	23457	16663	30656	11119	38323
CFAP74	24	76809	14429	75668	67828	82285	49700	119874
TNKS1BP1	24	19302	8160	17109	12915	21692	10724	44798
UBE2O	24	6630	1880	6896	5024	8316	3472	9327
ZNF518B	24	18379	3684	17622	15637	19779	13580	28486
XPO4	24	23816	6518	21490	18944	27242	15735	38563
SRCIN1	24	262320	39905	248883	238734	275935	211155	371563
WDR33	24	26673	6025	25137	22663	31028	16957	43504

PG.Genes	n	mean	sd	median	q1	q3	min	max
WDR12	24	18223	4616	18693	16385	19439	5978	28418
C20orf27	24	14171	5796	12428	9713	17221	6756	29452
PDGFD	24	7894	2140	7635	6342	9060	5358	13664
PITHD1	24	27649	8527	26425	23092	33735	12362	45023
COMMD5	24	23080	7055	21920	18886	25962	11636	46983
DNAI2	24	15580	5785	14716	11915	18414	5611	31352
WDR61	23	3961	1708	3781	2801	5045	813	8480
NIF3L1	24	16296	6964	14357	12785	17935	4689	34373
EGLN1	24	19249	8038	17548	15596	20503	8927	49233
NYX	23	125651	50794	112363	96255	139036	33389	250208
NAA50	24	11255	3861	10967	8595	14310	4343	18376
UBA5	24	15328	6023	13718	10400	20538	7153	24777
LHPP	24	14755	7331	14313	9450	17047	4781	37466
PAIP1	24	32416	10925	34057	22369	41700	16362	50027
NAT10	24	9119	2922	8591	7129	11657	4038	15027
COMMD4	24	3409	1042	3371	2797	3814	1476	5682
IQCN	24	27288	4900	25415	23985	29820	21854	40401
KLC2	24	199066	81837	207329	137897	240657	42561	385260
ILKAP	24	15636	6578	15760	10722	19451	1468	26571
XRN2	24	9819	3290	9133	7332	11622	5117	17477
TOLLIP	24	8487	3381	7780	5633	10715	4048	16817
INTS2	22	10614	8754	7240	4771	12584	1582	32022
KLHL25	24	116142	64205	108196	84155	121730	51302	389789
TKTL2	24	9215	3215	8478	7373	11205	4905	17662
QRICH2	24	38762	8479	38745	34335	41858	22379	66723
PARP12	24	274340	96688	249516	219504	314693	116289	533462

PG.Genes	n	mean	sd	median	q1	q3	min	max
CSTF2T	24	37110	11092	33822	29442	44434	20630	65171
PCBD2	24	26032	7905	25106	21996	29652	12027	46875
CYRIA	23	3770	1171	3694	2928	4199	1503	6393
HDHD2	24	43273	15560	39369	31833	53455	16711	78956
RAB1B	24	9814	5360	9126	6963	10875	4172	31406
MRPL18	24	16664	24444	10876	8504	15804	4298	130028
C11orf54	24	31303	13310	31177	21115	37434	13640	63830
FAM234A	24	49730	15578	49546	38170	57142	28888	87309
NAPB	24	2352368	827785	2013068	1886007	2539131	1472177	4276504
CDH19	24	9015	2739	8427	7481	10733	4882	18112
SIL1	24	21390	5655	20591	16698	24139	13159	33862
IRF2BPL	24	13466	4422	12543	9871	15648	7222	24694
UNC93B1	24	79770	26426	73398	64756	104468	26446	125475
CYSTM1	18	2793	1353	2663	1945	3269	864	7112
POLR3F	22	4596	1492	4686	3765	5722	1758	6808
NUCKS1	24	42507	26860	37847	23813	49887	8650	126774
TMX4	24	9747	5463	8406	6101	11783	3433	24990
MEGF9	24	3813	1663	3413	2719	4375	1750	8480
ATG5	24	10292	6835	8151	7278	10269	4172	38192
WDR13	23	5967	2409	5367	4509	6815	3271	14564
TSPAN10	21	11536	8390	7616	6472	15736	2961	38879
EPN3	24	61061	16251	59555	48108	73709	25737	87383
OR10A2	24	80177	28681	87882	59397	95807	28254	132179
EHD4	24	16717	16058	12020	8624	19061	5447	84115
GBA3	24	19408	8242	19051	13329	24150	7447	35293
MMP28	24	5811	2188	5430	4317	6724	1965	11112

PG.Genes	n	mean	sd	median	q1	q3	min	max
SPTBN4	24	120994	29782	119648	100106	128231	73515	201270
OR51E2	24	219686	130624	192580	120260	286409	51397	589719
SH3BGRL3	24	100407	30689	94516	78727	118772	57349	159505
SLC25A32	24	16893	3814	17475	14333	18765	9167	25575
HSD3B7	22	16041	8028	14115	11653	16911	4308	38233
TSPYL2	24	7053	4116	5967	4847	7596	2746	18265
BLZF1	24	57823	12314	58552	52769	62692	26263	81982
PPIL3	24	7962	3245	7106	5617	9470	3540	16078
SLC38A1	24	19944	8610	16761	14834	21916	11231	49882
PDCL3	24	47448	13079	47615	39440	54532	17895	73858
RAB3GAP2	24	87787	21685	81900	76404	93945	54151	157705
ADNP	24	25016	5275	26031	21488	28475	12256	33399
MRPL46	24	9665	3938	9815	8057	12302	2649	18213
ZNF106	24	28187	3553	28476	25906	30587	20247	34233
PNN	24	15914	4026	15054	13501	17810	9927	28059
OR52D1	24	31110	19044	25603	20790	32121	5134	76542
GGNBP2	24	20176	8515	19452	13421	24277	8918	45676
CPVL	24	60325	30206	53055	42000	67262	19261	164597
BOLA2	24	20100	7582	18588	14294	26193	7397	33156
TMX1	24	121005	65515	98659	89330	117910	38256	322531
ACBD3	24	7762	3218	7557	5036	9735	3032	14555
PTPN23	24	35689	8666	33475	30029	40562	23217	57256
UNC45A	24	119819	36453	109278	93406	144054	66708	191945
CHMP4B	24	15395	5432	15233	13231	18866	3285	27339
FN3K	24	10679	3455	10447	8379	12742	5434	17530
POFUT1	24	19334	7155	18146	14644	25069	6343	33622

PG.Genes	n	mean	sd	median	q1	q3	min	max
RNPEP	24	63699	20529	60456	51968	73362	28511	107924
GOLPH3L	24	6074	2976	5022	3624	7981	3108	13427
GOLPH3	24	9406	2671	8871	7939	10296	4538	15841
TUBB1	24	19778	5720	19698	16837	21977	7558	34241
SMOC1	24	8724	5448	8267	5138	10707	1892	24799
EPB41L1	24	48935	20975	44986	34594	53247	27765	115550
GLIPR2	23	5686	4398	3585	2814	7519	1774	17952
PLEKHA4	24	53385	12770	51972	47177	60946	28255	76754
EHD1	24	23937	15049	21420	15726	24931	9820	82963
PCIF1	24	42505	24399	35830	23213	55016	13515	110306
MROH8	24	1023061	602094	878248	711697	1129295	282836	3252302
ELOVL6	24	64178	35274	51255	47437	66925	27897	164345
CIAO2A	23	3729	1329	3695	2718	4100	2057	7977
SLITRK6	24	8251	2406	8573	6294	9885	4239	12635
STN1	24	5638	2086	5219	4246	6104	3183	10723
OPA3	24	35625	25398	29847	18307	47002	5827	101790
ACSS3	24	391437	110685	378475	297324	448681	204712	617112
YTHDC2	24	34598	11694	30698	28423	36720	19262	64379
CWH43	24	13097	3970	12355	10486	15967	6985	22314
SPRING1	23	51145	27754	49258	36656	59334	7483	122114
DCTPP1	24	24183	12832	24584	13912	33521	1990	50634
SMYD3	24	49642	10402	48249	42411	55368	31846	70633
AAMDC	24	24313	17672	23287	16333	24536	1825	94027
DOCK5	24	150988	43207	138646	116804	167637	105088	279507
ATP13A3	24	36927	7872	36487	31994	40025	21115	59098
SCAF1	24	22963	4547	21864	19691	26496	15811	32770

PG.Genes	n	mean	sd	median	q1	q3	min	max
UBE2Z	24	11529	5842	10992	9153	12926	2034	34913
NOL11	24	18730	14149	14263	11306	20341	7868	76986
METTL7A	24	75657	43156	62379	53115	99029	20471	218367
MMRN2	24	39201	5888	38536	35121	42626	31316	55566
AKTIP	24	29398	6836	28901	24280	33972	15107	40765
ANKZF1	24	86955	22904	85066	69036	97175	59650	165100
RNF121	24	16900	6482	15515	13535	17836	8866	35529
SLC25A22	24	60227	27262	53042	44082	75444	17364	128782
MED20	24	25331	7965	25974	19441	30570	11934	45973
QTRT2	24	14420	3823	13928	12033	16358	6761	25024
ARMT1	24	13945	7951	13771	8163	16072	2361	34777
PANK3	24	409868	546916	278910	216020	415788	17129	2859678
LRRC40	24	6333	1757	5994	5270	6692	3609	10884
AGO3	24	40669	15865	37581	29120	44896	21990	89192
CHODL	24	8079	3257	7603	5980	9308	3535	18655
NHEJ1	24	9472	2555	9368	7750	10492	5606	17010
CAB39L	22	9466	4777	8280	5998	12971	2727	20886
FN3KRP	24	12620	4320	13258	9964	14933	4484	23353
CARS2	24	34213	19285	28203	21327	40349	12728	82167
PPCS	24	100817	39260	108566	68235	125415	24463	159365
NMNAT1	24	51221	25099	47101	39220	52741	21745	139974
MLXIP	24	61041	16439	57244	49023	67865	39580	94113
C17orf75	24	22733	7896	21126	17741	26876	11254	41536
SIAE	24	14601	6075	13034	10298	18962	6407	27221
PLEKHA5	24	12490	3275	12905	9924	14647	7542	19566
UPF2	24	513946	86625	494284	465071	540779	338070	697477

PG.Genes	n	mean	sd	median	q1	q3	min	max
RNPEPL1	24	9282	3256	8697	7656	10143	4726	17494
GRPEL1	23	4987	4278	3236	2554	5204	771	16596
ELOVL3	22	3512	1272	3180	2794	4185	1645	7277
MYG1	24	548800	405177	432571	272151	607627	92131	1539241
KCNK13	24	13711	8002	11833	7937	19430	2877	28471
SEBOX	24	54013	30312	43502	38576	59567	18742	132859
SCPEP1	24	19867	12224	16427	12785	26978	4071	49589
CYP3A43	24	4328251	1440589	3786916	3339162	5211549	2801795	7335091
PIDD1	24	5461	1451	5587	4466	6514	2358	7947
PARVB	24	10582	7330	8481	6409	11566	4515	37545
TNS1	24	105407	71392	87459	75088	97263	62763	405644
NMRAL1	24	38802	14605	37210	29504	48728	12999	67889
SLC38A10	24	10852	1789	10988	9673	11841	7696	14515
ZNF287	24	47254	12165	48202	37960	52263	27154	84068
EML4	24	156868	74132	144306	98229	201185	60849	345870
GLOD4	24	177052	64790	164235	129955	225746	78768	299345
CBX8	23	6543	1915	6568	5634	7622	2056	11106
MUC5B	24	15944	13294	11709	6458	22244	2024	43464
NEK6	24	24102	15148	17878	13817	28540	9577	60760
SPON1	24	14605	7406	12407	9299	17905	5072	35363
MCCC2	24	44979	19362	37454	32981	56876	10462	100019
NCOA5	24	10184	5676	9077	6851	11230	4781	33572
MOV10	20	4338	2190	4042	2991	5313	1223	9907
ANKH	24	26150	9672	23208	20618	31386	12397	48456
VAT1L	24	27093	7135	25009	23046	30181	17686	45506
GPAM	24	33670	7448	32952	28836	38140	19166	54381

PG.Genes	n	mean	sd	median	q1	q3	min	max
RESF1	24	232720	50063	218356	203869	250464	168033	417948
PLXNA4	24	30561	4582	30922	26355	33048	21357	39999
PGAP6	24	7352	2084	7464	5680	8683	4027	13409
EPB41L4A	24	367011	63163	363901	331851	402518	280249	545748
XAB2	24	92481	26670	84095	74821	96925	64474	182526
CD248	24	65615	42765	55950	39569	74793	9302	188137
PREB	24	12834	7670	10713	8123	14715	2992	31552
CHMP1A	24	9897	4063	9612	7030	12272	2829	17630
RETN	23	9564	13812	6650	3656	9536	919	69363
APMAP	24	16625	5496	16400	13503	18260	9395	36251
TXNRD2	24	66323	26039	60960	44273	73880	37399	120489
SINHCAF	24	491421	200868	435405	358631	610878	151777	903652
ARFGAP3	24	27646	13598	24321	16986	36091	6945	60701
PALMD	24	19762	7916	17469	14147	24699	10296	39927
SSU72	24	5693	2921	4776	3675	6905	2478	15502
ABCB9	20	2928	1331	2752	1945	3396	1233	6236
VTA1	24	15395	5982	14254	10459	17920	5430	30446
DYNLRB1	24	12447	3619	12087	9972	14349	5060	19062
PARD6A	24	23253	12584	20979	16721	24102	12590	74028
GPCPD1	24	66098	19928	59665	54429	78472	28424	106758
CCNB1IP1	24	16577	3547	16347	13613	19063	9597	23494
A4GALT	24	15208	4391	14626	13273	16745	6633	25020
MYNN	24	11356	6795	9098	7193	14693	1901	28736
OSGEP	24	13242	5014	13513	10007	16049	4719	24743
ACP6	21	8092	4882	6311	4796	12246	2482	19854
ISYNA1	24	42020	37381	24473	15353	56555	7126	133749

PG.Genes	n	mean	sd	median	q1	q3	min	max
OBP2B	24	83488	86049	52973	32738	88527	1419	347983
NXT2	24	12413	2839	12384	10999	14060	6820	20044
CD93	24	123215	79193	103946	62664	159172	34733	346523
LZTFL1	24	80960	35993	78405	54858	94374	31876	179636
MEPE	24	21942	4878	21533	18296	25044	12073	32858
TIGAR	24	25967	9646	23881	20058	30326	11223	44608
RTN4	24	28290	10552	24237	21263	31369	18276	64855
CYLD	24	8334	2501	8018	7108	9095	4539	17677
HINT3	24	6881	2402	6104	5066	8740	3452	11446
RPRD1B	24	89523	58718	70238	57928	101967	42952	323686
RRAGD	24	11533	4873	10555	8777	13418	5389	25194
PFDN4	24	8467	2376	7984	6611	10594	4413	11882
NIT2	24	43772	13246	44756	31438	53216	20102	62894
AVEN	24	18320	4804	18022	15022	20818	10646	27831
EXOSC5	24	22080	8208	21386	16682	25687	10124	47825
FSCN3	24	11018	12240	7595	4911	10783	3451	61766
KIF13B	24	25929	9931	25356	17199	33476	12368	50252
PAK6	24	13977	2188	13629	12423	15542	10568	17961
ANLN	24	8726	3537	8007	6375	9787	3887	18144
XPNPEP1	24	33505	20424	27913	21255	36164	6340	81982
GPHN	24	36679	11230	35038	26835	46064	21453	60153
MYO5C	24	62193	10826	59340	55325	65932	49443	90793
ITM2C	24	16917	10808	14469	9221	20963	4784	53588
BIN3	24	33811	22038	31014	19230	38341	7178	111488
PDLIM7	24	68465	125553	29673	25588	38253	13579	504240
DDX21	24	107545	34918	100845	82897	124837	55519	189258

PG.Genes	n	mean	sd	median	q1	q3	min	max
MAN1C1	24	26794	8720	25797	19996	29615	16988	48512
NANS	24	131904	80484	99045	75084	185824	31942	361454
SH3GLB2	24	8110	3746	7427	5739	9628	3038	18798
EIF2B3	24	10887	1844	11257	9839	11598	7679	14794
FBXO6	24	30036	9009	29052	26423	32252	12499	50936
PICK1	24	19627	6353	18632	15833	21827	10816	36490
CTPS2	24	10939	4290	10334	8585	12283	5202	23931
CHRAC1	23	6159	6064	4812	4004	6206	1655	32833
PAPOLB	24	14455	9886	13668	8768	16508	5460	55662
ENAM	24	57567	12105	53456	49261	60997	44944	90709
AASDHPPT	24	15439	3425	14903	13056	17635	8590	21619
UBQLN4	24	25091	12794	20421	17478	29456	10847	63687
HEBP1	24	62848	29751	54263	43498	70392	27935	161140
RAB6B	24	12092	4968	10456	8755	15949	5439	24804
APOBEC3C	24	4364	1917	3812	3305	5222	1652	9869
PHPT1	24	18563	7530	17966	12761	22502	8196	39773
ARHGAP35	24	180582	25601	177527	163862	186403	145644	234164
FAM114A2	24	6209	1543	5909	5170	7070	3459	9349
LTBP3	24	18983	8475	16465	13009	23428	8852	38541
RGS18	24	13249	3359	12843	11003	14927	8031	19963
LANCL2	24	24082	6786	21890	18503	31148	14370	36485
KCND1	24	7052	2602	7170	4707	8858	2618	13065
CTNNBIP1	24	29777	7762	28260	25342	34353	13471	48196
ST6GALNAC1	24	20464	8555	18850	13640	24928	8972	41598
FARSB	24	160703	61992	173966	105699	190023	48025	285346
IARS2	24	83961	27049	74198	64341	93645	56993	166980

PG.Genes	n	mean	sd	median	q1	q3	min	max
STARD5	24	120939	75500	92694	73928	138878	43486	343713
ATG3	21	3578	2235	3652	1887	4286	651	10689
PDS5B	24	47776	10448	45860	41931	49947	31694	69116
OLA1	24	75226	39565	67071	55551	74991	26977	188073
CUTC	24	23219	9655	22689	18364	28534	7766	54453
RBM12	24	40671	16440	38347	30880	47797	14328	71983
ANKEF1	24	29175	6084	28559	26091	30322	20782	50725
ZCCHC3	24	18926	5443	18407	14888	20227	10769	35745
ABHD10	23	6328	3632	4830	3800	8110	2625	16633
STAU2	24	38451	10398	35514	32641	41270	18433	71399
TMEM106B	21	4988	2484	4638	3081	5860	1320	10646
LIN7C	24	11135	3478	10700	8953	12852	5200	19309
CYRIB	24	32424	11374	30245	26632	39411	11032	59846
DDX19A	24	15336	5207	15069	12343	16763	7494	29267
GIMAP4	24	6814	2671	6069	4616	8653	3745	14692
TDP1	24	6159	3119	5403	4688	6334	3670	19512
TBC1D23	24	2402	1086	2122	1755	3164	613	5075
MRGBP	15	3321	2650	2385	1520	3933	1077	9528
SEPTIN11	22	3771	2627	3434	1831	4907	456	9403
SLC38A7	23	16178	9502	15238	7659	19987	5114	42734
MED17	24	34461	5277	33394	32243	34544	25642	48228
PARVA	24	45704	71040	26986	21659	33107	14223	339553
PANK4	24	74344	16070	70904	64543	82362	50882	111949
FBXO28	24	6855	2076	6572	5623	7610	3886	12356
TBC1D13	24	23541	8637	22460	16962	28816	11493	40094
ARL8B	24	146190	57410	125299	105281	180692	73713	290738

PG.Genes	n	mean	sd	median	q1	q3	min	max
DNAJC17	24	14236	8951	12326	10819	14176	7080	54605
DDX18	24	23170	6389	23219	18497	26010	13840	38262
ASF1B	22	3918	1329	3471	2926	4542	2281	7321
TBCCD1	24	54881	18376	48619	44077	61895	26641	109835
ARMC1	23	4601	1950	4282	3705	4774	2054	11565
MTPAP	24	16414	9195	14261	11429	16928	5807	50026
RLIM	24	39044	8839	39691	33072	42509	23316	60973
RBM22	24	32511	15800	27963	23900	32909	18095	88865
WDR70	24	56976	9734	55832	52139	60636	37019	81436
SLTM	24	32000	5733	31651	28967	33213	23850	48082
PAG1	23	11459	5367	10489	8640	12530	5556	27700
PARPBP	24	5324	5883	3870	2866	5551	1595	31278
PAK1IP1	24	15594	6191	12697	11017	20268	7976	32398
HIF1AN	24	7152	3677	5657	4906	9234	3264	17714
CZIB	24	120634	59772	111492	89229	133701	51601	369224
BABAM1	24	12186	5422	10474	9414	12022	6547	27164
CLN6	24	17181	6176	16092	13735	20776	7288	33380
C2orf42	24	29668	13695	25633	21162	34242	8620	69307
THG1L	24	12520	3156	12020	10618	14952	6272	21082
IRAK4	24	32002	9704	29554	27245	37202	15198	58268
COMMD8	24	28183	31610	20661	14804	27774	6871	167394
NHP2	24	6475	2812	6463	4259	8119	1233	12872
OCIAD1	24	12567	4500	11468	9500	15204	5766	24256
ADPRS	24	10222	4491	9780	7104	13311	3075	17699
HYPK	24	7283	1784	7479	6239	8610	3340	11135
CHCHD3	24	21094	9362	17614	15604	26258	7709	43318

PG.Genes	n	mean	sd	median	q1	q3	min	max
ZSCAN32	24	274711	160313	245374	182313	302657	90995	758596
SIRT5	24	6933	2557	6066	5275	8602	3736	12711
THUMPD1	24	26015	13131	25980	17020	31664	6960	73929
PGPEP1	16	3435	1421	2925	2226	4514	1606	6351
FBXL12	24	11740	6205	11990	7272	15543	3941	31453
GIN1	16	1887	955	1800	1321	2230	650	4615
NDE1	24	16592	3803	16130	13948	19083	10760	25163
BABAM2	24	10892	2467	10808	9219	11710	7227	19125
CDKN2AIP	24	6826	1641	6514	5792	7483	4476	11136
GAR1	24	9839	4311	9022	6344	12356	4371	21561
STAB1	24	229096	39891	230131	206435	257861	149218	294842
PPP4R2	24	64317	15602	64729	53792	76750	33814	88653
SLC5A4	24	18879	8219	17453	14351	19978	8683	42134
TERF2IP	24	15080	7583	12678	10224	18639	7911	37156
BCLAF1	24	58907	22051	52569	45007	69997	37293	139228
COA4	24	9203	7741	6356	4198	12917	1588	35630
TLR7	24	32966	23353	25489	20332	35060	14060	112960
MAP3K20	24	4366	3607	3141	2247	4638	1487	15082
TMOD3	24	11337	5475	10052	7405	13530	3901	24854
CELSR1	24	80069	25285	75171	62819	87004	45370	135171
FAT2	24	1328242	154891	1336947	1274684	1426321	958186	1548298
NKIRAS1	24	17445	8214	16021	13571	18215	7410	42478
UGGT2	24	43260	11828	39306	35085	47960	32092	83150
UGGT1	24	11161	4753	9609	7540	13845	6078	21184
TAS2R5	24	8298	3151	8095	5772	10611	3329	16367
RBAK	24	30139	11769	27220	22462	32529	15446	61890

PG.Genes	n	mean	sd	median	q1	q3	min	max
ERAP1	24	22772	15215	16402	12072	35949	6046	54150
ACTR10	24	19034	5475	17381	15866	20440	12956	37246
C9orf78	24	59368	34267	45640	38233	65716	22326	146934
FAM120A	24	24877	6633	23056	21052	27110	15766	45234
WWOX	15	5039	2714	4260	3165	6183	1130	11056
SMARCAL1	24	12127	6282	10916	8133	14115	3470	29184
GLTP	24	39681	24015	33721	24242	43127	5676	105122
AHSP	24	68107	65448	39111	28226	90298	9785	246450
NUDT4	24	9330	4859	9084	5190	12765	2782	20501
EHD3	24	1633661	1195224	1483282	996825	1815897	540454	6330590
EHD2	24	15013	24395	6957	5429	8516	3101	104434
CNOT2	24	48076	31235	41752	28067	61340	700	111975
C1RL	24	5280	2197	4627	3794	6862	2366	9804
TMOD2	24	25743	7023	26300	20377	28837	12256	42119
CALML5	24	5604	2113	4750	4119	6838	2770	9943
OGFR	24	5684	1221	5214	4891	6789	3810	7732
LMCD1	24	7171	7570	4692	3926	8052	2599	39122
COMMD9	24	15523	8555	14359	10545	16541	3506	36015
THYN1	24	11814	8227	11638	6000	13367	2658	35070
NDUFAF4	24	49274	9153	49632	41886	58219	32944	63481
HACD3	24	2290	1793	1575	1085	2979	507	6418
PDP1	23	9470	3493	10291	6714	12016	3171	14217
VAPA	24	39130	17089	38599	25074	48391	17328	82502
MACROH2A2	24	16984	13999	13470	10639	16738	4981	74878
RNF181	23	5190	2231	5192	3851	6028	1030	10717
MTRES1	24	6456	2409	6368	4723	8256	2869	12494

PG.Genes	n	mean	sd	median	q1	q3	min	max
GSKIP	23	4004	2291	3639	2311	5758	375	10173
ZNF581	23	15506	15470	10080	6851	15175	5191	74919
SPATA7	24	26125	4816	25360	23019	27457	19217	41863
ABRACL	24	53443	27455	52881	32607	71348	8519	101457
MDFIC	24	28466	5170	27838	24741	31121	19871	38702
ACTR3B	24	15897	2643	15297	14098	18044	12034	20781
HCN3	24	16917	5406	17531	12078	19836	10456	30925
WHRN	24	17164	3060	16440	14588	19019	12648	25641
PLCE1	24	1660931	830730	1408686	1047886	2234161	603561	3491920
VPS18	24	7705	3756	6717	5770	8376	3306	21546
RCC2	24	48685	27092	49473	26098	58753	10873	105190
SLAIN2	24	6455	2141	5839	5119	7443	3826	13509
USP36	24	186849	74711	171651	149006	196215	96163	470994
FNIP2	24	38147	14560	34910	30892	41430	19945	84427
STK26	24	128557	37106	126344	100594	142746	80544	247065
PTGFRN	24	139956	42753	127488	118192	153848	75942	229550
RRBP1	24	35256	11291	33008	28771	44405	17648	62027
MAP10	24	26444	9844	27982	19348	31566	10168	50284
KLHL8	24	10993	5631	10500	8218	11208	4129	33398
CEP126	24	26747	5925	25060	22411	29011	18824	39006
CPSF2	16	14755	7728	12344	10609	16679	6012	32877
LARS1	24	18025	7378	14887	12040	22476	9491	32963
CC2D2A	24	356238	119884	319799	275893	381749	155023	652972
RBM27	24	52835	18966	48651	39544	58682	25046	97548
RERE	24	27026	8334	25646	22401	31047	16537	46730
SUCLA2	24	366035	116353	364230	288280	433209	138226	608744

PG.Genes	n	mean	sd	median	q1	q3	min	max
WRAP73	24	17136	5017	17134	12962	20730	9763	25355
STX18	24	28401	6919	26024	23903	33484	20345	45319
DELEC1	24	32112	8838	34731	24965	38239	16490	47205
ATXN10	24	1848794	770281	1676851	1339666	2150783	665449	3683829
TFIP11	24	293147	53850	287322	257587	330188	156724	385645
MYO1A	24	105019	15528	106729	94876	114722	77688	146131
SPRR3	23	9620	13772	4998	3895	8758	2666	70153
HSFX1	24	75064	48236	64086	44626	88475	6043	201395
SAE1	24	43315	17530	39346	33470	56041	13333	85481
NLK	24	10956	6374	9285	6824	13207	1566	25696
COPG2	24	86544	24471	83194	71784	101019	46579	135169
MRC2	24	43788	11005	42300	33393	54203	27860	61884
IL36RN	22	3839	3311	2931	2178	4442	794	16766
COMMD3	22	3030	1671	2686	1900	3721	1078	8097
GNG12	23	9580	5310	8312	5156	13061	3027	23319
HCST	24	30039818	18124064	28792908	14311498	42130164	6850172	73186056
CPNE7	24	20606	7802	18275	14544	27146	8795	38034
DKK3	24	17215	4529	16072	14086	19686	11435	26372
VPS29	24	9814	3212	10161	7367	11609	4453	14714
GRHPR	24	269989	85040	279904	212687	300004	110438	434517
CTSZ	24	38236	27244	33453	22696	44079	5597	145210
UBA2	24	15975	5772	15231	13621	19403	4638	29792
DKK4	16	8415	5230	5849	4404	9734	4084	18811
NXF1	24	124185	63866	114799	91580	131675	64861	397697
PEF1	24	9380	2594	9372	7272	11286	5184	14636
BIN2	24	38737	12579	38541	28113	41954	18221	72768

PG.Genes	n	mean	sd	median	q1	q3	min	max
COPS7A	24	16054	3521	15147	13752	17855	10972	24237
FBLN5	24	5432	4695	3908	2191	6883	1178	19279
APEX2	24	745973	359224	662860	546395	820981	207801	2025804
PPP1R1B	24	11227	5108	11388	7078	13134	3293	21644
AASS	24	44513	13375	40388	34915	54892	24050	72579
ZNF212	24	22651	4241	21291	20079	25019	15918	31241
CFDP1	24	5748	2362	5878	3525	7138	2460	11865
VTI1B	24	16961	4732	16364	13639	18510	10521	27554
STK39	24	8638	2090	8483	7216	9777	5354	13306
LRWD1	24	14590	7347	13252	10408	18720	5668	33476
NIPSNAP3A	24	32706	23596	25048	20881	40526	13601	128278
ABCF2	24	4902528	967084	4773928	4241548	5345595	3319602	7199780
TES	24	9211	8869	7158	4985	9390	1524	40529
DMBT1	24	36035	12553	32995	28828	39431	19666	70339
FETUB	19	3616	2200	2830	2034	5243	603	8093
LIMD1	24	31224	6540	29939	25781	35874	20571	42881
HMGXB4	24	12994	2277	13179	11536	14513	7728	17003
APOBEC3B	21	9858	4947	8386	7294	11160	4691	25742
SWAP70	24	306717	77755	315902	253339	356848	179269	431936
FBXO40	24	255461	128031	218151	198126	259434	110806	693960
LAMTOR3	24	13384	7843	12285	8316	14498	5922	40750
LIMA1	24	61765	19150	58928	46602	74232	32577	105403
SRP68	24	33443	9002	32214	26243	42379	19614	48805
CNTNAP2	24	8395	2244	8336	7549	8687	5743	17663
NPC1L1	24	475363	220059	436257	321888	499287	163396	990744
CHORDC1	24	19600	8383	19243	12516	26688	6938	34378

PG.Genes	n	mean	sd	median	q1	q3	min	max
UBQLN2	24	4828	2427	4511	2950	6859	1422	10286
EGFL7	24	3787	1810	3483	2926	4414	715	8333
IL20RA	24	7897	3200	7645	6276	8930	2291	14756
ADAMTS1	24	58982	28473	50858	39289	70057	24794	134391
SHPK	24	6532	2727	6201	4658	8310	2210	12279
DPP7	24	53537	38641	43894	30150	62091	13962	191925
SAP30BP	24	31265	8642	27614	25351	34735	21853	59673
ZNHIT2	24	1927784	848885	1570328	1393664	2122500	938490	3773408
PFDN2	24	143582	34268	140715	119946	162109	73244	212473
PUF60	24	21080	8482	20507	16116	25936	6572	40502
NRBP1	24	18113	8303	15512	12726	22207	8595	36926
ENOPH1	24	30803	11300	31461	22950	37371	12194	53169
ATP6V1H	24	4828	1653	4400	3704	5754	2442	8610
TAGLN3	24	10082	4816	9057	7632	10728	3666	22933
TRMT112	19	7084	4256	5902	4081	8436	1940	17142
CPA4	23	7612	6236	6611	4492	8812	1850	33863
XPO7	24	17844	7498	17140	13194	19533	5690	37088
BAZ1B	24	70780	9909	71211	63635	77239	55214	96129
ATP5IF1	24	5699	2227	5502	4028	7233	1794	11794
AK3	24	16068	10378	13022	7352	19591	4890	45315
CNOT7	24	9083	3715	7991	6977	9970	5602	22649
SERPINB13	24	14697	4330	14868	11056	17126	6740	24908
GGT7	24	40288	14107	36054	34184	45236	12104	88721
RABGEF1	24	18920	3888	18951	15375	20788	13242	29717
MAGEL2	24	8299	4179	7154	6096	8826	4501	22555
NAGK	24	39556	14248	38773	33235	42669	11885	88012

PG.Genes	n	mean	sd	median	q1	q3	min	max
CRLS1	24	14185	3828	14692	10886	16754	5969	20770
SH3BGRL2	24	10986	5321	11356	7302	14599	3215	22369
RASAL2	24	62893	13282	61504	52798	68384	45878	98436
DBNL	23	6413	2210	6534	4532	7829	2211	10178
DCTN4	24	8964	2987	8164	7205	10873	4269	15333
ZNF229	24	32831	7724	32880	28398	37189	16075	47937
CDC23	24	25636	8099	24250	20194	30202	14194	40479
ANAPC2	24	15321	5688	13812	11929	17404	6652	28617
LSM7	24	32183	7886	33143	26846	36610	17079	47307
SERPINA10	24	8267	1408	8129	7136	9005	6274	11372
DBR1	24	7296	2632	6980	5810	9013	2300	12499
TASOR	24	27883	8108	25606	23828	28591	16726	54401
FEM1B	24	17570	3893	17555	14925	19597	10510	27806
JPT1	24	30644	11767	29709	23528	37511	8113	51180
FBXO3	24	7719	3193	6654	5949	8187	4333	19457
AKAP11	24	104146	11407	105187	97809	109483	76278	128602
DNAJC12	24	50849	24281	42377	38901	60441	20208	113269
CPSF3	24	10165	2500	9828	8607	11383	6104	15356
PITPNC1	24	12216	4960	11708	9321	13263	4632	31094
APPL1	24	9006	3009	8878	6530	10644	4826	16317
PARP4	24	29631	7093	27984	24747	33129	21403	53692
NUDT5	24	35149	17138	31900	20900	45080	10614	68687
RCOR1	23	5521	2058	4968	4154	6896	1990	9636
MAN1B1	24	65981	12338	68959	59960	77235	39423	81473
MYO15A	24	156256	29114	148524	135497	167459	119339	234503
GTF3C4	24	14761	3950	14322	11981	16090	9188	24041

PG.Genes	n	mean	sd	median	q1	q3	min	max
ADAMTS6	24	81912	42421	71066	61797	89846	40264	259679
PACSIN3	24	26132	12953	21872	18120	30224	9924	58238
FBXO4	24	10628	5037	9009	8072	12534	3557	28763
FBXL21P	24	78428	22507	80075	62548	92483	43574	126720
ACAD8	24	389183	148123	333621	288268	462790	226842	763466
ACIN1	24	9029	2234	8794	7598	9415	5047	14403
AGO2	24	67536	18377	63118	53061	74939	44777	113368
MYH2	24	338342	84469	329763	286289	374004	219013	547980
MYH13	24	88387	14700	84304	80567	92712	63342	133739
NUP50	24	147686	35105	138549	127360	168663	89245	213255
ZHX1	24	108213	28964	100347	87904	130092	63240	170752
DSE	24	8028	2230	7488	6537	9552	5092	12827
AGO1	24	10341	3648	9347	7917	11459	6088	20714
RAB21	24	18314	9902	15658	12748	19883	7545	39661
RAB22A	24	6992	2380	6988	4895	8940	3889	12303
PSME2	24	146722	79395	132588	101558	185026	35987	398130
RAB23	24	11413	18437	6045	4652	7662	2292	86237
MCTS1	24	32575	8945	33666	27808	37955	15268	49519
MTUS1	24	60805	15136	57473	50430	69418	31777	93274
PALD1	18	10282	5825	8859	7111	12049	2444	24874
SLC39A10	24	364182	179648	334040	208093	450076	154193	795705
ZBTB21	24	92217	52345	78689	60315	98581	37420	271101
KIAA1210	24	66734	15862	63377	58492	69549	49535	129633
PLEKHG1	18	5444	4267	3343	2664	7723	1651	14099
CNOT6	24	111588	49874	97690	79296	136298	62353	290201
TBC1D24	24	6809	2357	6786	4954	8217	2241	11614

PG.Genes	n	mean	sd	median	q1	q3	min	max
PPM1H	24	64074	20217	58896	51418	71846	36095	120914
KCNS2	24	15748	6888	13783	11518	17842	7074	29419
ZNRF3	24	23455	6571	22359	19748	25883	15558	42409
HECTD1	24	58952	19000	55394	49929	62529	29563	108787
MYO5B	24	95439	16624	94347	86865	98976	65548	133108
FZD4	22	5133	3253	4598	3221	5861	185	17170
CORO1C	24	232655	190389	199304	127094	260163	67870	1006620
NAP1L2	24	41774	18849	37926	29453	50585	8800	89063
PYCARD	24	30860	21559	24537	16173	33255	10075	83340
PADI4	24	86368	29865	79976	65346	94563	52847	175533
EPDR1	23	5541	3948	4606	2243	8199	655	12814
MYO6	24	20890	10391	17923	12693	28532	8350	43143
PPT2	24	34276	27922	23281	18806	36332	7680	108463
NFU1	24	40668	17940	35349	30841	42652	19640	85589
PRPF19	24	23996	10251	23049	15907	29509	8786	42108
SYNPO2	24	50699	20062	44512	41298	55497	25512	110140
NENF	24	11354	5790	10404	6914	15649	2440	22088
VPS4A	24	88172	24976	81328	74017	97961	50079	147663
ARHGAP26	24	91861	21522	86170	75422	107426	57705	134851
STUB1	24	99038	23048	99204	82127	112496	67542	170762
CDC14A	24	17350	5131	16228	15077	18792	6702	28558
SNX6	24	32079	8783	30430	26341	37697	14185	52725
PSMD13	24	22872	9484	22683	17292	25634	10037	55428
FAF1	24	23032	7388	22447	18854	24682	13806	47311
PROCR	21	3032	2689	2334	1472	2896	665	11911
DIMT1	23	4099	2225	3922	2659	4629	1565	10480

PG.Genes	n	mean	sd	median	q1	q3	min	max
TIMELESS	24	9638	2739	9344	7943	10362	6214	18285
WDR3	24	11541	5367	10587	7709	13589	3991	24885
NSFL1C	24	39351	15062	34166	28814	46514	20211	86497
ADAMTS8	24	11377	3656	10524	8764	12724	7398	22697
COG5	24	35181	8241	34247	30095	36676	24383	57787
MACF1	24	152272	12387	151451	142690	162227	132105	176523
SCAF8	24	28348	7695	27904	21516	31326	18768	48483
TRIM35	24	18423	5847	16994	15340	19905	12027	33360
ZC3H4	24	12382	1721	12183	11564	13450	8901	15808
SORCS3	24	77911	15777	73253	65593	89972	58600	112411
USP24	24	78208	25334	71632	64041	81830	48597	156079
SAMD4A	24	6900	2092	6553	5531	7929	4132	13284
PHF24	24	16099	15550	9456	6441	19131	4618	71120
TRAK1	24	43134	11423	43868	32724	48405	24070	64910
SHANK2	24	15962	6060	16298	10787	19448	5637	27572
SRRM2	24	43093	7310	42369	37984	45804	32664	60742
CNTN6	24	15098	3927	13687	12090	18207	9630	23089
PA2G4	24	65142	30541	55832	40792	81988	25391	141910
SPG7	24	9821	1875	10046	8166	10890	6908	13978
CLCA2	24	29275	11753	27836	25195	32513	9841	64062
ERVW-1	24	154595	56208	139102	115426	194021	71911	288410
MAPK8IP1	24	13344	5868	11180	9129	16298	6128	27716
ZNF148	24	21965	8470	20158	15599	24397	12337	46748
RTRAF	24	19028	10273	15145	11421	25956	5563	41612
RUVBL2	24	50377	28625	41033	26651	71617	18331	114795
LIPT1	24	11644	11374	7320	4853	11793	2653	46350

PG.Genes	n	mean	sd	median	q1	q3	min	max
NOD1	24	26335	10042	26057	18602	34135	10523	46321
CLEC11A	24	8120	1233	7798	7285	8916	5678	10310
AKT3	24	22584	7051	20857	18561	23696	12784	44986
EIF3L	24	16804	8712	14017	11423	19550	4618	35755
PLAA	24	42433	14318	41830	34358	51612	18832	74169
RUVBL1	22	6198	5897	3909	1712	9209	377	22189
NUDC	24	375822	164699	386507	233190	499376	113937	679155
ST3GAL6	24	17093	6641	15818	12130	20535	8126	33602
HS3ST2	24	36374	10980	32611	30140	38558	20935	64270
CFL2	24	463845	229989	431544	323510	484705	230983	1402231
FARSA	24	23087	6401	22170	18752	26791	13004	40847
ASF1A	16	2088	1003	1754	1422	2879	574	4384
DRG1	24	6305	3561	6218	3197	7234	1739	16153
NCKAP1	24	20054	5998	19562	16649	23087	10986	34276
CNPY2	24	90423	54490	81887	52477	118765	25632	217608
DTX4	24	36402	8913	37831	32138	40455	19562	50640
STK38L	24	27731	10001	26535	20500	32469	15741	58358
INPP5F	24	23404	3757	23575	20711	25694	16118	30689
PLEKHA6	24	42072	6139	41804	36822	45352	32228	58584
WDR37	24	9884	4387	8677	7041	10375	4049	20142
PADI2	24	20390	8901	18264	14136	24436	11783	52861
ZKSCAN5	24	154317	39855	162253	118383	177340	84021	239993
SLC27A6	24	17897	8758	15449	10991	23851	6416	40679
LAMTOR2	24	33569	13307	29822	25100	35406	19269	72581
TMA7	23	60421	33154	46902	35385	87470	21569	124055
AP3M1	24	5438	2041	5166	4524	6141	2202	10617

PG.Genes	n	mean	sd	median	q1	q3	min	max
CARHSP1	24	39484	20347	34556	25136	52114	6472	81398
THRAP3	24	18458	7955	16488	14268	20175	9492	48109
WBP11	24	14741	4280	14227	11117	17997	9354	25511
NOP58	24	15732	7297	14524	11195	18455	7310	44289
DERA	24	24644	14835	21903	16538	28084	10271	86022
LSM2	24	27593	11596	27307	17389	34980	8182	49219
STARD10	24	15195	10474	13297	9662	17471	4362	56720
CAB39	24	33389	11003	30587	27373	39848	15759	55110
LUC7L2	24	7448	2580	6939	6401	7907	4424	17999
MRPS2	24	26376	6529	24987	22196	30549	15394	40013
SBDS	24	34909	25144	31390	22089	38195	7989	136854
EXOSC1	23	7474	4002	6969	5107	8806	2604	20581
SF3B6	24	8977	5237	8693	5435	11147	1127	27199
REXO2	24	11071	3160	10695	8933	12223	5074	20355
WASHC3	24	38814	27835	30936	27174	35184	5834	143544
PPIL1	24	66315	24293	61303	52209	74189	40185	161033
UFC1	24	36659	26127	29303	25586	40832	10760	149325
FIS1	24	23397	10994	23858	14489	30195	7635	47732
AK6	24	31655	17943	25623	17806	40662	12858	78770
HDGFL3	24	15131	26568	8338	4028	11522	663	131073
BOLA1	20	6616	3887	5451	4025	8587	1490	16252
CHMP3	24	68977	25923	63438	51508	87469	23264	119857
CHMP3	24	6708	2161	6099	5336	8087	3404	12680
STRAP	24	17459	6676	16721	12748	20585	7018	30301
RTCB	24	24106	9231	21728	18601	31241	9117	43838
RABGAP1	24	37474	8427	36207	32309	39510	26264	56795

PG.Genes	n	mean	sd	median	q1	q3	min	max
TSC22D4	24	31241	11321	30533	23331	35627	16835	66048
SAMHD1	24	31861	18101	26014	23303	38791	9201	95929
HBS1L	24	12666	5387	10825	9629	14227	6042	28587
SALL2	24	5249	1317	4883	4467	6092	3345	8681
PRKAB1	24	13502	2358	13335	11872	15608	9650	18019
TLN1	24	69773	49139	55798	45016	71869	33571	278681
MFHAS1	24	147567	53440	142613	104709	185913	65350	266825
ZNF451	24	60170	22674	57114	46216	64998	25997	141264
USP15	24	290477	77653	265492	239700	342817	188845	457388
TOGARAM1	24	37032	7976	37474	31372	42409	22095	56420
RIPOR2	24	85844	15733	82241	74660	93113	68401	121733
TLN2	24	69583	10863	67857	62793	77794	50673	89393
IRS2	24	1388941	319779	1341997	1176311	1561631	942664	2086938
LOXL2	24	292961	46265	293404	263431	319899	196191	398246
CRYBG1	24	35588	15941	31279	24282	42578	12784	77616
MAP4K5	24	145565	50228	133406	117715	174242	35817	229823
HYOU1	24	26443	14963	20469	17712	31850	10228	66564
TBL2	24	2199942	858629	2002690	1752895	2201216	754806	4620120
TELO2	24	233809	62408	223697	201012	251959	116982	431449
ARIH1	24	7868	2688	7716	5811	9715	2885	14408
LSM4	24	25642	10403	24833	17259	31546	9339	47427
OARD1	24	7319	4849	6210	3850	8263	2950	24009
HSPB11	24	12628	5350	12472	9178	15189	4025	26003
SUPT16H	24	18337	4109	17929	14784	21435	11828	27480
PCDHB2	24	6907	3364	6499	4611	8159	1096	15802
PCDHB14	24	21508	10096	21325	13597	28869	5638	46760

PG.Genes	n	mean	sd	median	q1	q3	min	max
TIMM9	21	2025	1449	1691	776	3185	426	4675
TIMM8B	24	70936	29752	63417	52066	79196	35522	163246
PCYT1B	23	3161	1535	2906	1819	4373	1166	5915
CD2AP	24	21955	10303	20749	14476	26862	6968	46456
ATP6V1D	22	3555	1474	3229	2516	4648	1121	6422
TIMM13	24	13347	4890	12845	10297	15951	5369	27721
PPP2R3B	24	21064	5114	20600	16853	23579	13938	34937
DMRT2	24	34268	16106	27505	24855	35618	16029	72897
TRAPPC1	24	40577	13079	40824	32613	46132	21213	76115
CDC42BPB	24	198480	33930	190362	180371	215618	134380	293438
RBM8A	24	25451	13515	22388	18963	26380	8224	66080
ZNF706	17	4507	2391	3911	3466	5074	1189	12632
WIF1	21	7195	3920	6705	4070	9081	2310	15945
SNX13	24	124169	16784	119407	113413	137731	97223	161774
SNX9	24	7930	6509	5359	4895	8278	1006	31935
SNX5	24	12375	3958	12318	9092	15024	6210	24155
NUBP2	24	11831	32736	4224	2545	6228	1868	163799
ST14	24	6595	1855	6366	5182	7946	3704	10106
LYVE1	22	5189	5120	3799	2156	6026	577	24940
HEBP2	24	56904	22257	49673	41326	72984	17854	111843
HEBP2	24	57009	20443	54318	44929	68234	27335	118948
PUS1	24	55023	23605	50699	39452	58663	20587	119230
LRRFIP2	24	19742	3892	18706	17441	20519	15035	31180
PSAT1	24	40017	29671	28908	18183	56717	3801	104979
F11R	24	4886	1851	4349	3758	5911	2581	10569
CPQ	24	20417	15147	13059	10467	23836	4764	58960

PG.Genes	n	mean	sd	median	q1	q3	min	max
SPIN1	24	1110537	515971	904218	767824	1372564	482010	2795163
COPG1	24	21582	9740	21079	15160	29449	5809	39767
CLIC4	24	56259	49338	48182	31063	63875	16857	265365
CFAP20	24	38813	19752	34368	24211	46994	15047	90489
SAR1B	24	6039	2591	6184	3897	8095	2536	10619
EMILIN1	24	26056	7248	23904	21695	27499	16995	44771
ARFGEF2	24	11112	3185	11231	9106	12772	5913	17375
ARFGEF1	24	21393	6096	19512	17705	25445	13601	42869
STK24	24	7658	2152	8118	5910	8989	3882	11367
BZW2	24	14311	5272	14108	10104	17918	5795	24807
COMMD10	24	29357	29328	25282	13031	30389	7819	152383
DYNC1LI1	24	12426	3124	12648	10161	14453	7233	19539
CHCHD2	24	15781	8807	13384	9191	23186	3263	31780
TEX264	24	630867	365581	509525	447710	645388	339081	1891093
DNMT3A	24	93489	34231	85562	76617	103428	45528	182711
OAS3	24	10720	3028	9855	8945	12938	4894	16372
AK5	24	38944	6537	36987	33969	44571	29215	49835
SQOR	24	15053	4742	14071	11718	17756	8407	27255
LAMC3	24	231652	60857	226958	190733	267622	114985	365251
CAPN6	24	18996	3412	18570	16823	20786	13386	26284
FCGBP	24	67296	67227	44603	18715	70937	10959	208708
CAPN7	24	138508	23042	140298	127701	156109	94193	176376
WASF2	24	22265	5393	21546	18670	25806	14561	33787
FAM169A	24	14767	5826	14029	10221	17188	8134	32166
IVNS1ABP	24	17664	4227	17104	15199	19456	11421	29983
SEC23IP	24	61350	12516	58071	52246	65459	46644	94056
