# Supplementary material for: Data-Independent Acquisition (DIA)-Based Proteomics for the Identification of Biomarkers in Tissue Washings of Endometrial Cancer
Source: Int J Mol Sci. 2025 Nov 27;26(23):11498. doi: 10.3390/ijms262311498 (PMC12691889; doi:10.3390/ijms262311498)
Supplement: Supplementary file 1 [file ijms-26-11498-s001.zip › Supplemental file S3.pdf]

Descriptive statistics of all proteins identified with at least 60 % of values in the Low-grade EC group (N=25)

PG.Genes	n	mean	sd	median	q1	q3	min	max
PPIAL4E;PPIAL4D;PPIAL4F	25	11208	10772	8363	5437	9948	3383	50826
TRAV17	24	13474	10443	10957	6986	16933	936	44148
DPEP2NB	25	3281	3485	2478	1461	3180	411	17131
CENPVL3	25	37960	26811	29655	21202	48806	7694	132094
TEX48	25	14188	17277	9294	7803	14633	5813	94151
CPHXL	25	8148	6184	6089	4833	8782	405	27066
ERFL	25	45372	198830	4121	2987	6103	2188	999478
TRBV4-1	25	25313	25341	14885	8698	30656	4150	104235
RBM47	25	7249	2958	6040	5020	9587	3228	14064
UBA6	25	73272	29367	63057	51720	100519	30609	138663
ESYT3	25	14922	5742	14339	11235	17944	6689	32207
UHRF1BP1L	25	31753	7862	29224	25321	36302	20631	53184
SHTN1	25	55058	18625	52320	41306	63323	30452	107584
TLDC2	25	62094	17892	59900	50169	71501	33447	100552
BICDL2	25	40049	14186	37520	32574	40587	27674	98469
ARHGEF37	25	28834	9156	27965	23816	31154	10495	58484
MEX3A	25	41938	16883	38067	29057	49153	17560	81475
ELOVL7	25	404643	317261	336454	189817	442572	20046	1488903
SSC5D	25	514310	254301	511910	300798	681798	138721	951118
SH3PXD2B	25	195469	48853	187937	160802	212436	139366	322720
PIPSL	15	4529	2962	3361	2954	4996	1153	12990
HYKK	25	6260	2139	6030	4848	7009	2954	10749
CRPPA	25	20242	18278	15410	8628	20782	5190	81468
MEGF11	25	14851	7476	11447	9510	19846	6595	33224
FAM172BP	25	25382	12482	23081	17443	29983	8951	64629

PG.Genes	n	mean	sd	median	q1	q3	min	max
VWA3A	25	33777	8124	33413	28914	37877	17680	50651
C2orf78	25	23756	12527	19288	15647	27431	12907	69702
ANKRD33B	25	76430	37102	73699	46418	98811	23767	153943
IQCA1L	25	14870	5067	13189	11367	18233	5580	26147
PALM3	25	3708	1983	3086	2586	4348	1731	11108
SYNDIG1L	25	68817	40789	63227	35705	79672	16064	176939
PGP	25	11119	5629	9844	5838	13711	4749	23544
TRIM49B	25	14877	10484	11293	6542	19300	4715	47317
C5orf51	23	3086	936	3356	2492	3802	1063	4513
SOWAHB	25	81562	49281	68516	55670	81806	41510	262762
MROH2A	25	27593	4525	26927	23973	29843	20484	38183
EFCAB10	25	15920	7942	14146	10300	19643	7431	35279
WIPF3	25	15731	12566	13494	8335	16585	3647	66256
PCARE	25	57727	18265	51270	43748	69372	33866	98036
ANKRD61	25	67023	25379	61645	56310	71635	19273	155481
OTOL1	25	53210	35810	41321	33133	59076	22048	187955
FBLL1	25	7760	3413	6433	5370	9387	3803	17548
SMCHD1	25	3911889	857738	3610741	3372082	4147031	3009934	6657894
UNC119B	25	18469	6964	16503	13551	21008	10746	34313
LRRC72	25	10191	5860	9129	6496	11647	4374	31381
SDR42E2	25	50065	21336	46081	37456	59135	20530	106510
CPSF4L	25	15394	5020	14312	11950	17494	8636	30661
ANXA2P2	25	75439	44215	58848	43522	94326	24501	180474
NA	25	327542	268077	257751	151676	430994	15636	976720
FAM90A27P	25	20671	6876	19429	16217	24842	9047	35800
ENO4	25	97086	35456	91442	69003	105393	46015	185524

PG.Genes	n	mean	sd	median	q1	q3	min	max
C4orf47	25	7763	2413	7731	6538	9225	2391	11831
SRRM4	25	88694	30115	92130	74773	105690	31443	159693
A2ML1	25	58540	34885	48255	36386	66381	28556	189208
GABRR3	25	7871	5154	6764	3969	9857	2265	20735
NEURL1B	25	792434	262322	707771	654952	912493	373018	1471648
PRSS47	25	35885	15663	31524	26103	45380	16376	72768
NA	18	7373	11214	4083	2783	5955	192	49847
BBIP1	25	8530	4793	7012	5311	11120	3046	24509
SERPINE3	25	23376	5886	22340	19705	27023	11896	37615
FADS2B	25	34221	11707	32229	27728	37718	17885	75661
WASH4P	25	7130	3312	5869	5150	8162	3429	17574
CAPN14	25	13348	4705	12082	10898	14288	7148	29833
NA	16	2348	1647	1997	1035	3105	687	6898
NUDT19	25	8754	9199	6957	4587	9335	2655	49836
DCDC2C	25	12212	3530	10926	10108	14638	6207	22185
FOXO6	25	7001	2366	7186	5209	8324	3546	14342
MIF4GD	17	2965	804	2978	2654	3147	1696	4663
ESPN	25	61948	24928	56584	43766	74958	28216	130660
SNURFL	25	12789	6539	12306	8098	15949	4822	33636
ZNF487	25	135558	75806	109830	84324	154292	32948	327823
OR11H12	25	45946	22004	44018	36000	48823	12006	107486
ZNF732	25	6990	2126	6633	5242	8200	3483	11257
ANKRD66	24	3559	1497	3478	2698	4137	820	8403
KIF28P	25	67841	15169	64417	59561	68950	49928	123199
C4orf51	25	23244	13429	20423	15110	27833	6691	62436
HSBP1L1	24	44516	18526	45273	29539	53774	13849	75842

PG.Genes	n	mean	sd	median	q1	q3	min	max
MCRIP1	25	10837	3089	10559	8754	13539	5047	19368
AKR1B15	25	49080	11146	48985	42201	58825	31052	68359
MCIDAS	25	8813	4293	7175	6089	10744	4717	24579
PROB1	25	7141	1936	6801	6108	7664	4254	14134
NACA	25	170217	33684	168163	149433	178398	118364	268803
C11orf98	22	2610	2233	2316	1416	3013	829	11828
STMND1	25	20186	5899	20055	16344	24763	10207	33643
CROCC2	25	29081	9517	25891	23890	30462	18990	62947
TRIM77	25	10763	2931	11461	8942	13041	4295	16046
SLC35A4	25	8684	5214	8456	5254	11063	743	22839
ASDURF	17	26790	38282	9427	8161	27418	3935	156141
OVOL3	25	24459	15507	19988	17645	25323	12731	93032
DNASE2	25	12733	8386	11922	6074	17092	1750	33598
AGPS	25	23250	7925	22641	19250	25166	10554	45122
DDX39A	25	179923	70845	169529	134897	239924	46012	306757
PDLIM1	25	88766	46722	77962	61454	114546	20953	199981
AIP	25	28668	10117	27139	21011	33712	12881	51342
GTPBP1	25	63702	28686	53882	43217	81871	26137	137983
STXBP3	25	12174	3428	11430	10044	13838	7316	24967
SMAP	21	3892	3643	2969	1897	5220	232	17137
LGALS8	25	142077	38843	142728	119847	158010	80808	231102
NFKBIE	21	2016	1415	1847	1137	2077	525	6617
PSMD11	25	20586	9859	21445	11715	26817	6049	43884
PSMD12	25	24309	10954	24927	14713	33074	7921	49139
PSMD9	25	16227	4841	14713	12852	18243	9754	26990
RNF103	24	7397	3737	6763	4199	8582	2891	17465

PG.Genes	n	mean	sd	median	q1	q3	min	max
ATOX1	25	17480	10832	16309	10426	18406	3929	59015
PGRMC1	23	8722	14619	2989	1882	5155	1034	49268
TAF4	25	400719	129781	365727	328317	443741	233192	870213
DFFA	25	34022	28786	28081	22782	31604	12565	162814
CLIC1	25	230381	84652	214299	186739	265089	73546	452604
EIF3F	25	19149	9311	19985	11785	25023	3243	40545
QSOX1	25	14486	10485	10385	7568	16229	4857	46922
DCTN6	24	9509	4788	8794	6618	11324	3023	21230
WASL	25	59398	17216	61788	43732	69926	33727	100447
PDE2A	25	559012	293051	481631	440630	541703	331879	1845667
FOXN3	25	18902	6057	17390	15464	20221	11649	34276
POLRMT	19	26487	12318	24558	19485	32746	4633	56525
EEF2K	25	201305	50461	217072	159689	241115	79194	275135
EML1	25	4070	2263	3732	3090	4323	1479	13541
IGF2BP3	20	4460	4181	3234	2194	5696	787	20410
GOLIM4	25	77982	28366	73844	54035	94084	44369	146227
EXOC5	25	6866	3307	6204	5307	7476	3536	21151
HMGN4	22	12578	16010	7020	2836	16002	640	61020
PSMD14	25	30740	10474	30053	23959	38556	13981	58169
KPNA3	25	22077	9233	21896	15868	28713	5365	44577
USP9Y	25	18344	5054	17120	14244	21480	12454	30948
LAD1	25	19279	7477	16689	14312	22589	10662	44798
VWA5A	25	243132	152798	211139	159617	253287	55410	608654
NOP56	25	30860	7972	31119	25197	35862	18777	47692
RNASET2	25	84476	73663	71826	26847	99370	6422	336829
GABRP	25	99852	41269	92375	67553	125799	38690	198054

PG.Genes	n	mean	sd	median	q1	q3	min	max
PODXL	25	162618	306887	73844	35117	159688	16325	1566333
FCN1	25	12914	6347	12365	9741	14388	4235	31832
PIR	25	76995	56316	63437	45863	88432	11020	238771
KPNA4	23	6169	2466	6109	4751	7808	2368	12465
NFIB	25	98974	32952	103796	65610	118181	53114	165947
PPP6C	23	9804	5250	8745	6941	11993	2211	25512
PIK3C2B	25	54433	19337	50614	43052	59488	32532	109409
FBP2	25	24721	18989	20021	11932	35482	1487	80154
UBE2C	16	18717	22608	4267	2498	28692	1104	79373
PDXK	25	32000	18811	29198	18911	36714	4938	87457
SCD	25	12664	7323	12901	6178	16684	2924	29134
ISLR	25	7884	5656	5357	3752	9552	1688	22771
NCKAP5	25	134011	49762	122230	107750	141349	90846	340090
CUX2	25	65706	14457	64697	55225	68794	50885	118538
SOCS6	25	198733	45815	195260	167379	224775	123597	306879
TRAFD1	25	4451	1945	3964	3034	4951	2258	8954
GAPDHS	25	38540	17120	37070	28747	41806	14606	93777
HSPB6	17	17955	25417	5198	1790	16399	341	75623
NDUFAB1	25	21376	30594	13687	8533	19677	2217	152708
UBFD1	25	12136	5446	11302	8777	13702	4484	30116
COPE	25	31593	30842	23944	13684	34168	8086	148352
CCS	25	16265	5231	16189	12338	18448	8392	29876
ENPP3	25	47637	26087	46426	29558	54055	17025	139797
DVL2	25	60581	24775	57135	43725	72005	32953	125616
DNALI1	25	17435	6895	15527	11761	20804	8060	34420
ADAM10	25	184422	82752	171326	133066	220292	90046	479202

PG.Genes	n	mean	sd	median	q1	q3	min	max
MAP2K7	25	112444	37599	103752	83862	142496	66698	197439
PDCD5	25	24606	14224	23158	15695	31785	3273	70355
SLC9A3R1	25	74898	45893	57654	40835	97767	17237	205352
TPP1	25	11583	5391	9701	7851	14748	3707	25302
TCERG1	25	138072	46829	134753	107529	145979	74359	285119
KIF3C	25	17903	9536	16403	14453	18793	8251	58751
UNC13B	25	70624	13256	71460	62811	82121	49303	94571
POLR3A	25	84426	39765	74941	70949	83613	53120	262503
PSMA7	25	44447	18994	43541	35636	53831	11343	96391
OPLAH	25	14198	8504	12525	10754	14944	7950	53143
SLC30A4	20	8863	7670	5680	3101	13983	819	28887
IFIT3	25	32146	14136	30706	25453	35230	12890	83518
IRF6	25	13768	7721	10497	8950	16745	5186	34439
TAX1BP3	25	42947	20474	39243	30357	52612	12789	98794
GIPC1	25	9400	3920	8524	7308	10020	5457	22793
HAT1	25	11369	6129	9936	7639	11932	4465	31233
MYL12B;MYL12A	25	31424	19136	24181	19188	33845	14863	84321
AURKA	25	39722	10175	38260	32135	47493	22406	63465
CLGN	25	18301	12971	16207	8849	22593	1277	52863
PPP1R12A	25	58415	17182	52871	47837	66520	26784	94212
AZIN1	25	168487	57121	169829	136495	207405	54532	275474
XPO1	25	38223	21703	34520	24773	47796	9236	118325
PLXNB2	25	17446	4037	16786	14849	18623	12197	30492
SEMA3E	25	104504	25670	102448	90424	114225	48498	150384
U2SURP	25	35615	11244	34474	27183	40134	21751	73978
SETD1A	25	31145	8978	29321	26256	32012	19478	60770

PG.Genes	n	mean	sd	median	q1	q3	min	max
KIF3B	25	48633	27860	42433	34420	50502	25759	165685
PFAS	25	23773	10405	22542	13867	30605	10500	51027
NACAD	25	9770	2906	9231	7939	11737	5221	18243
ADAMTS3	25	49608	14338	44355	39817	58974	31253	77074
LSM1	24	12355	6277	10729	8081	14675	4220	30088
ARPC1B	25	42979	22585	36662	29093	56818	6721	85782
ARPC2	25	88827	37645	73940	66755	116141	38796	183577
ARPC3	25	79565	35320	73635	59037	91019	21817	187667
TBXT	25	17701	6850	17076	13354	22228	2624	29773
PFDN6	25	9003	3755	8379	6848	9797	4904	21515
LAMA5	25	33575	6263	32045	27982	37599	25858	48276
CASC3	25	131623	31022	133618	119916	138695	86443	256473
CLIC2	25	31112	20490	25796	14907	38275	8789	75675
RTL8C	25	4649	8658	2141	981	2724	392	42938
MAPK13	25	95308	29137	83876	78190	104822	58232	157791
SPTLC2	25	78858	21498	78831	64016	86854	40035	132526
FANCG	25	12121	4407	11382	9310	15579	4112	20628
OGT	25	70315	22226	63986	54265	82665	41464	133403
PMM2	25	26447	13796	27466	14455	36469	3523	55922
INPP4B	25	39336	22300	36352	28643	41635	24563	140364
HMGB3	25	41039	23496	39686	25568	55169	8416	99195
PPM1G	25	17272	9646	15407	9316	22596	4526	42961
INPPL1	25	13258	4234	12076	10459	15676	7322	24124
EIF3H	25	8947	3020	9634	6598	11266	3891	15788
NVL	25	78818	20480	73530	66269	93619	34071	116932
BCAT2	22	5898	5050	4143	2500	6929	1196	23809

PG.Genes	n	mean	sd	median	q1	q3	min	max
BCAT2	25	46918	12645	44043	37596	56072	28255	72511
STX7	25	39095	19297	33494	25025	44779	12646	92398
SLC16A6	25	48943	23484	42812	32754	56429	14322	110714
CAPN5	25	9711	5626	8000	6129	10772	4660	26700
YKT6	25	32322	12241	31084	24219	39729	13179	57497
ARPC5	25	50808	21845	44815	39586	57891	19895	97948
POLR2D	25	3602	1719	3402	2606	4438	1089	7883
NKX2-8	25	5343	3612	4400	2916	8133	1171	15002
DDX3Y	25	15982	6280	15380	11508	20237	4727	30744
RGS5	25	367378	203506	301764	260537	354982	214508	1037296
DHX15	25	170647	69654	162584	137210	199193	41081	314202
ZZEF1	25	289226	73418	263945	237718	336119	197288	545310
FLRT2	25	21181	12123	16504	14133	25116	7941	56821
CYB5B	23	2080	1590	1615	1080	2311	407	6818
CYP26A1	25	105475	85708	87909	68598	96932	57696	492143
PHGDH	25	49703	37993	39309	20421	73376	4825	171982
NDUFS4	19	8841	19764	3543	2684	4692	1948	89718
GPR39	25	19670	9030	17516	15669	21540	10645	56118
SEPTIN4	25	66244	25868	60704	49501	70041	36614	137652
DYNC1LI2	25	9369	3505	9055	6994	12141	3595	15324
KLK10	22	7820	9966	4807	3636	7754	652	49525
PSMD3	25	38506	17000	41229	25503	51818	10107	69366
PAPSS1	25	17063	4366	16530	13646	19902	10597	26655
SART1	25	35380	11200	34512	25120	46561	19345	53208
TGFB1I1	25	103322	71623	82585	57926	132196	22808	324981
SRGAP3	25	133025	41027	126451	109754	139264	91008	297320

PG.Genes	n	mean	sd	median	q1	q3	min	max
ZNF264	25	16178	3917	15829	13597	17798	9279	26659
HSPA12A	25	13076	8281	10704	7929	16027	3877	44171
MSI1	25	21660	6847	19848	16068	26000	11422	36584
WDR62	25	35609	12488	34464	23777	43325	20545	65666
PRPF3	25	4910	1922	4380	3886	5134	2843	11410
TXNL1	25	39809	15000	38328	31432	46633	15558	68384
ERI3	25	9464	3030	8836	7971	11104	4904	17255
FIBP	25	9468	7930	7006	6358	8379	4655	42850
EIF4G3	25	71994	27636	62561	57467	73783	53104	189780
PPIH	25	24565	11454	24552	17061	31667	7843	53825
HTRA2	25	15658	6602	13337	11364	17417	5858	33897
AKR7A2	25	20005	5889	18866	16746	22908	9832	31902
EPB41L2	25	28161	13019	25736	18552	34686	10525	61690
EPB41L2	25	136780	103064	90271	69610	179534	33483	454257
LAMTOR5	25	8814	8404	6785	4173	9278	1315	37908
WIPF1	25	18574	19029	11982	10186	18210	6247	97407
XRCC3	25	25629	10500	22662	17889	28987	11977	52593
DENR	25	22565	7354	23553	17264	27354	6524	42185
XPOT	25	55991	19781	55450	42277	64730	26056	129208
DNPH1	25	22706	10432	19149	13845	30632	9293	40883
DCX	25	21189	15322	16124	11196	21979	6738	64022
TIMM44	25	13619	6859	12804	9764	15027	6042	41274
TRAPPC3	25	10754	3413	11206	8466	12473	3877	18497
CHMP2A	25	18233	8726	17587	12104	21461	9208	49330
NCK2	21	5966	1879	5595	4314	7213	3110	9798
PSCA	25	16231	6370	14229	12130	19943	6183	33425

PG.Genes	n	mean	sd	median	q1	q3	min	max
TSPAN6	18	3024	2562	1852	1483	3181	542	9847
PLRG1	25	9234	4549	8212	6952	10184	5350	28231
GET3	25	20887	6670	19793	15271	23647	13954	40750
ACTN4	25	116118	48652	103230	87691	132838	38418	247900
GSTZ1	25	29704	17373	26686	17927	40605	6560	70294
TRIAP1	25	13489	4198	12957	11509	15631	5070	22841
HTATSF1	25	16832	4958	14808	13535	20494	8761	27887
STX6	25	10433	3757	9147	7586	13067	4757	19425
SYNGR1	23	2278	1448	1893	1351	2581	818	6641
SYNGR2	25	864703	529247	775570	550874	921779	17696	2335157
SYNGR3	25	84061	56051	73945	62863	88097	23717	331641
SGTA	25	13788	5799	13144	8866	16606	4811	26754
NARS1	25	37107	15001	39076	29861	49643	7368	58315
NUDT21	25	26556	11992	22854	19164	31451	9149	60247
LANCL1	25	44819	14938	41348	34694	54980	21335	80696
RRP9	24	13125	27237	7496	6152	9256	4615	140618
B3GALT2	25	4226	2247	3557	2881	4734	2195	12926
SLC37A4	25	8393	2996	7799	6661	9112	4089	16042
IDH3B	25	75494	22713	68034	60401	82389	40988	130219
AHCYL1	25	14559	5902	13993	10987	16180	4784	26073
CD5L	25	13091	13943	8936	5572	13826	3218	69078
NDUFS5	25	24614	16494	21375	14387	28615	7074	84514
PDE6D	24	7506	3868	6917	4874	8437	3492	22285
RAD21	25	6048	1620	5785	5306	6855	2895	9393
DHX16	25	33891	7605	32225	29395	36472	25661	62172
ZNRD2	25	11444	3935	11623	8179	14367	3204	21381

PG.Genes	n	mean	sd	median	q1	q3	min	max
GMFG	25	26593	13071	23607	19942	28816	7547	70539
PPP1R12B	25	16127	4773	15141	13386	16511	11148	34440
BNIP3L	22	9024	3903	8898	6815	11628	2907	19018
ADGRB3	25	14344	3140	13777	12152	15997	9275	21208
PCDH7	25	54962	28346	49397	36672	62962	21990	136146
SMARCA5	25	17527	4014	16122	14403	19881	10969	26427
KIF5C	25	34122	20425	29748	27619	33685	20055	128815
NUAK1	25	56393	25274	49461	43551	57659	27230	149302
ZNF862	25	77128	10886	79486	65979	85476	55895	94127
SIPA1L3	25	1211946	212125	1167598	1047374	1337662	898322	1761886
AQR	25	8267	1965	8174	7026	8941	5017	13485
MAST3	25	151548	45818	144969	118620	167591	100893	277881
OPA1	25	292533	121153	267078	250085	318001	144946	801333
PPL	25	222207	90545	201688	152571	247270	128868	441262
GSDME	24	3024	2306	2801	1613	3479	716	10930
PLXNC1	25	32655	13519	30865	26119	34501	19704	90580
ACSL4	25	310896	230843	220835	198039	338876	122006	1288645
SNX3	25	18092	11437	13483	8479	22806	4328	45964
CUBN	25	9999	1676	10032	9073	11047	7085	14890
DOK2	25	44963	9522	45105	38441	49850	27981	62174
SORBS3	25	58364	16423	56659	45282	68009	21536	91242
CDC40	25	28274	11679	26966	22169	33266	9841	69691
RANBP6	25	22167	6388	21858	17550	25436	11403	38101
CCNT1	25	8433	3892	7482	6456	9114	4069	24127
PLOD3	25	94144	52611	75855	64235	105485	35841	298665
CCNT2	25	3675	677	3681	3297	4235	2253	4904

PG.Genes	n	mean	sd	median	q1	q3	min	max
TLR5	25	12334	6152	10205	8323	14495	4150	32577
TLR2	25	428661	199126	387803	319298	519339	97418	862695
SELENOF	22	4276	3492	3439	2565	4352	1028	17748
TSPAN1	21	15510	20899	7921	4273	16328	1218	84836
EXOC3	25	172447	47623	165135	140528	190700	108251	346982
JAK2	25	8485	1288	8702	7536	9304	6470	10526
MAFK	25	186516	69605	169200	139698	243088	81203	314722
KPNA6	23	6089	3488	5286	3447	8310	1297	12110
SRPX2	25	42038	21985	33918	31232	47570	15084	113116
UGDH	25	71581	43480	61404	38496	99254	10634	151111
SNX2	23	6370	2978	5562	4900	7223	2567	16589
SNX2	25	39239	10862	35805	31692	47173	21801	62456
DPM1	25	69453	15070	68929	55814	77197	43467	94529
CCDC22	25	31435	12661	29274	25948	33048	18538	84647
DKC1	25	3400	1188	3430	2674	3956	1386	6608
EIF5B	25	14269	4905	14312	10152	17136	7241	22845
MMP20	25	22143	14686	18250	14218	23443	5790	62079
DNAJA2	25	11601	4322	11370	8523	13830	5871	25606
BRD4	25	8419	2126	8105	6709	9773	5285	12556
CUTA	25	64189	37086	51067	39797	77969	16397	171070
CTSV	25	5383	2663	4806	3862	5832	2252	14523
PFDN1	25	12544	6920	11577	7521	16258	4641	35123
PPP1R11	22	3006	1883	2608	1718	3721	897	8250
NBN	25	94260	23262	92684	81590	103827	57811	163136
DTNB	25	19061	11704	13726	12235	20027	9123	53812
KIF21B	25	31226	15490	27386	24714	30876	19048	91890

PG.Genes	n	mean	sd	median	q1	q3	min	max
SRGAP2	25	100195	47164	86606	70927	108053	51313	261765
WDR1	25	127937	58304	117620	91702	152311	38673	268025
N4BP1	25	16935	5120	17575	13376	19955	7525	29876
ROCK2	25	10859	2263	10567	9634	11657	6359	17796
CLASP2	25	61448	19015	58436	51850	72036	29718	108656
CPNE3	25	17358	8813	16314	10859	21338	3091	38716
CLUH	25	14976	4312	14123	12106	16945	8192	26237
CNOT3	25	29813	11226	26539	23415	37326	8040	54278
GGCT	25	42980	22725	42548	23332	56134	10885	96257
ZPR1	25	9995	5718	8376	7387	10342	4525	31063
NIPSNAP2	23	15291	14151	13058	5795	18019	2538	68501
CILP	25	26118	12626	22004	18310	26495	11471	68827
PDCD6	25	16695	15615	12726	9640	17769	4575	68359
ZNF253	24	3197	1425	2931	2298	3868	1178	7119
TBCA	25	29336	18668	31499	14710	40127	2811	74808
ATP6V1G1	25	13726	6590	12753	9528	17030	3017	30159
VPS4B	25	16066	6720	14699	11099	21207	7094	30664
ZNF217	25	356739	68117	338140	314912	394764	249552	531090
SH3BGRL	25	178822	89619	164279	110699	234411	40658	343019
FLNB	25	10476	10568	8021	3634	10597	1082	50844
NCOR1	25	87006	43102	76126	63746	95590	48654	276126
NDUFS6	25	27768	12835	23920	19774	34345	10501	65580
ULK1	25	72677	16984	66461	62761	81456	45813	114446
CS	25	58502	50045	45221	31148	58350	14486	253109
SEC22B	25	36198	22084	31695	26903	40283	6849	117898
POLQ	25	88181	12283	85162	79825	96501	64980	119955

PG.Genes	n	mean	sd	median	q1	q3	min	max
VPS26A	25	41937	17758	41245	31337	51103	11918	79646
PMPCB	25	11180	10182	9853	6522	11987	2601	56857
TECTA	25	93401	46388	71011	64550	118722	43600	226799
KATNA1	25	28843	14984	26617	19810	34312	11362	79989
RDH16	25	10309	9927	7129	5151	10407	3962	43506
ERN1	25	191726	93826	158210	130798	222096	61867	507017
PSIP1	25	12914	5770	11932	8643	14410	5897	27216
CLN5	25	25288	8678	22705	19811	29559	8522	48241
HSBP1	25	8894	5213	7588	5427	10252	4262	24565
KHDRBS3	18	4050	2516	3783	1567	6280	1137	8442
RBMXL2	25	36334	11368	37056	31929	41484	11813	64842
BANF1	25	109740	68375	109172	57171	146408	16754	291409
SF3B1	25	416631	113528	402172	349618	459912	264511	684316
WBP4	25	8141	4846	6656	4217	10932	3526	20884
SCGB2A1	25	22721	54251	10274	7181	15268	2575	280552
SKAP2	25	646240	329052	567926	427900	761694	257378	1840029
PGLYRP1	20	4693	5561	3037	1048	6281	319	24631
NPM3	25	30420	16188	26650	19311	43153	2396	59216
LEFTY1	25	35523	27916	25275	20003	39596	10603	118825
CREG1	24	29262	9880	30264	22997	36416	10527	49782
SNRNP200	25	237045	53649	240032	197867	267281	156416	368224
TIPRL	25	55752	25494	51900	43055	64214	9470	122874
RFPL2	25	22226	9831	19474	15805	27108	10767	50660
PPM1B	25	9246	14645	6311	3657	8447	1394	77233
RP2	25	3489	2322	2726	2028	4137	916	10652
GJB3	24	9262	3927	8349	6172	11680	3994	18285

PG.Genes	n	mean	sd	median	q1	q3	min	max
STK16	25	31981	15391	29082	23431	34800	18596	95895
CRTAP	25	9656	2913	9912	7427	11276	5353	15180
B3GALNT1	25	95884	37639	84075	71115	112451	47720	185141
TCEA3	25	5012	4661	4210	3054	4891	882	26301
PALM	25	66701	15770	66479	54370	76613	39480	98297
RNASEH2A	25	37901	13182	37535	26691	44648	14725	58788
EIF3G	25	32985	13820	30922	24618	41733	10210	64564
EIF3J	25	19114	11879	13962	9566	25425	5798	47990
CBR3	25	25804	17599	19890	13061	38459	9856	76416
PSMD10	25	17321	4116	16302	14668	20379	10301	25050
ZMPSTE24	25	47891	23238	48108	36979	51685	11258	112698
IDH1	25	231439	166994	169355	122550	323651	48688	700374
GATB	25	20755	6648	19206	16709	24105	8677	36188
SCO1	25	70078	34965	66644	47885	81484	29242	190441
ATRN	25	18638	34335	10709	7810	12809	6047	181563
RBBP9	25	30463	18328	26190	20360	34902	9746	96891
STAM2	25	7336	3235	6608	5408	8335	3691	19721
SULT1C4	25	8203	2588	8235	5856	9247	4784	14285
ARL6IP5	18	4305	2872	4252	2276	5104	1103	13287
DCTN3	25	10067	3889	10108	8312	12366	3171	16860
DNAJC8	25	23709	21719	17293	14663	24974	7914	122012
ATP5PD	25	10721	3343	10066	8464	12119	4579	18220
SASH3	25	107079	68399	89558	69262	124308	44028	373929
GLRX3	25	32838	13369	34179	22124	39429	11775	61592
RSL1D1	25	23093	8014	21288	17413	27847	10200	43110
WFS1	25	12572	10063	8428	5327	15056	3097	40327

PG.Genes	n	mean	sd	median	q1	q3	min	max
SNCG	25	19844	27571	12042	9056	16710	5436	144508
CIAO1	22	3409	2244	2722	2188	3885	920	11463
DFFB	25	7876	6010	6272	4990	7984	1960	32276
SRP72	25	46045	16876	45455	32245	58349	19368	76995
DDAH1	25	67322	40055	57352	43169	87824	8713	150527
MTA2	25	24374	7188	24919	18015	28846	11666	42751
KBTBD11	25	6409	3377	5213	4101	7325	3065	18453
ATP10B	25	17872	3566	17419	15501	19093	12626	28058
TOMM70	25	5676	2884	4561	4235	5531	2981	15568
IPO13	25	16956	7531	17240	12927	19922	2101	35749
TOX4	25	35791	13633	33599	28214	40595	18623	75915
SEC24D	25	8018	3416	7998	6162	10015	2337	13901
FCHSD2	25	105452	33036	98563	76862	131285	55278	184235
SASH1	25	45210	12125	43750	39155	47253	28499	80793
TMEM63A	25	45546	13675	45757	36250	53011	19059	77553
UBXN7	25	74345	22609	74277	61127	86358	35848	131591
SUN1	25	22554	11478	20794	16208	24242	6160	56420
PLPBP	25	27589	10643	27039	17937	33157	11978	47462
ERLIN2	25	8850	6968	7359	5867	8220	2167	39193
PCF11	25	15314	3073	14360	13226	17198	9848	21077
ENDOD1	25	36070	14677	32610	26685	39071	15533	70778
GLCE	25	20543	6903	19332	15189	27193	10820	37168
FBXO21	25	6441	3550	5169	3977	7530	2089	15171
RHOBTB3	25	59555	21150	52465	43426	66532	39364	123043
USP19	25	39265	9270	37869	32220	46120	26371	63474
AP2A2	22	6799	2775	7496	4724	8847	795	11427

PG.Genes	n	mean	sd	median	q1	q3	min	max
CLSTN1	25	16916	18120	11205	8228	18891	5644	96951
INMT	24	7685	8428	5172	2872	7870	404	36995
AGFG2	25	26694	12559	22835	17602	32938	8798	53742
NKX2-2	25	7397	2969	6478	5310	8875	4353	17078
ZFPL1	24	5000	2235	5270	3581	5872	936	11393
ELP1	25	24557	6284	22571	20293	28481	15217	40209
NDUFA7	25	9613	5564	8694	6176	11905	675	25414
ZNF205	25	20899	6472	19464	16290	26061	12204	36567
STBD1	21	4327	2402	4194	3351	5003	1293	11999
ZRANB2	25	11733	5182	10196	8524	11199	5192	23311
SNX4	25	10395	2548	10540	9065	11904	5623	16807
OR6A2	25	33496	31474	23916	15895	33903	7786	144609
LUC7L3	25	36237	12722	36153	29456	41212	10643	74730
LRAT	24	11085	8482	7826	6109	12650	1228	40521
GOSR1	25	7949	4726	6863	4983	8335	2150	24091
KCNH1	25	14513	2094	14214	13381	16035	11038	18335
LYPD3	25	4563	2061	4264	3172	4989	2050	10735
VAPB	24	4926	3121	4378	3578	4933	2193	17812
PGLS	25	61477	18734	62234	51544	77069	23463	97039
PAPSS2	25	1801132	1397804	1496801	1161665	1994618	817901	8033445
ATG7	25	16459	7505	15629	10691	20012	6813	32219
LYPLA2	25	26171	10562	24322	19741	32040	9762	58765
IPO7	25	43520	18539	40144	31173	55746	9563	81646
ARIH2	25	24007	20728	18311	11601	25092	9909	105573
SLU7	25	13561	4451	12999	11600	15205	7189	28908
CD2BP2	25	25309	8647	22867	19003	31783	13389	43354

PG.Genes	n	mean	sd	median	q1	q3	min	max
BAG4	25	23045	19364	18628	14311	21472	5789	93653
AHSA1	25	10688	4552	11342	7323	13177	3544	19037
SLC34A2	24	4649	2501	3877	3241	6564	842	11106
ABCA1	25	55062	23909	50684	38194	63428	27979	116913
H6PD	25	47326	21428	40255	30412	64422	16556	91353
SEC24A	25	17344	3674	16606	14879	19258	11629	24824
SEC24B	25	94976	32081	90142	77856	103838	57757	205271
VNN1	25	3920	1823	3644	2594	4362	1563	8381
PRAMEF12	25	499104	356843	375100	283882	610981	120930	1698231
C1orf105	22	4540	3292	3487	2278	5543	1343	14063
ETHE1	25	25068	19273	20710	14231	27360	7327	97346
ZBTB11	25	19749	7847	18904	14737	22257	11244	50984
STAMBP	25	48902	13543	46105	39980	52878	22445	84115
ECEL1	25	99585	27229	92780	80150	106706	62745	159236
RAB3D	25	22730	6529	21107	17912	28308	11306	34793
OXSR1	25	38514	13877	34563	29330	46666	15807	64407
GGPS1	25	12144	16269	7878	6382	13471	3193	87971
LSM8	25	22326	13704	20578	15546	25553	4190	76033
AP2A1	25	61929	26427	51104	41155	78374	31674	122523
WIZ	25	84481	15436	83782	72927	90533	64383	128291
DDX58	25	24855	8944	22006	18418	25004	16524	50002
CAVIN2	25	13324	6459	12527	8529	16957	3461	25844
BAG2	25	10289	4518	10096	7821	12914	2542	22181
BAG3	25	8782	3040	8171	6681	10001	4744	18823
CRYZL1	25	26595	7356	25040	23463	26779	14775	49301
AIFM1	25	22468	28408	14993	9178	21671	4581	119148

PG.Genes	n	mean	sd	median	q1	q3	min	max
EML2	25	24494	11777	21042	15619	28244	8867	54680
NUDT14	25	140257	177220	88550	77759	113259	48070	770955
TSPAN15	23	17228	8141	17747	10387	22328	5399	33718
BPNT1	25	26102	13408	22228	16901	40253	9507	53563
DDAH2	25	55516	28247	53027	33278	67774	17611	114648
ABHD16A	20	2971	1551	2801	1779	4177	400	5821
TXNDC12	25	38281	21733	32506	26967	45213	11547	115430
ECD	25	77179	34839	60521	56025	92892	23673	151360
EFEMP2	25	11706	15426	4633	1881	13329	1189	59252
SCGB1D2	17	106479	360550	14803	4951	33147	949	1504132
RECK	25	4537	4085	3349	2691	4352	957	22371
NUDT3	25	18506	6362	18394	14381	21888	6713	29010
AGR2	25	37462	42229	25110	15934	39899	4296	178966
PAK4	25	44889	19630	39855	33255	55223	23952	117791
APBA3	24	12811	7692	10924	8168	15188	3858	37228
ACTL6A	25	83425	50718	72819	49352	87698	29313	232220
MOCS2	23	3093	1379	2920	2016	3959	1253	6442
CYB5A	25	7901	11831	4474	3207	6245	1568	51784
ADH1B	25	96836	98537	61587	37716	90469	20681	363995
ADH1C	25	9678	3640	8419	7296	12386	4158	18102
ALDH1A1	25	196930	157062	157762	109696	209204	19228	754576
SOD1	25	258001	106711	238475	200351	316244	88359	551156
CP	25	226078	202136	148751	103285	278538	62625	981476
PNP	25	106569	49340	94762	86911	128521	26451	280584
HPRT1	25	53120	17734	52350	38906	66483	31426	93938
GOT2	25	33031	15799	25968	19887	43908	13889	61336

PG.Genes	n	mean	sd	median	q1	q3	min	max
PGK1	25	1071718	388692	1083774	776001	1314973	309932	1950067
AK1	25	85783	27757	86776	61825	105655	30277	144237
C1R	25	1154876	759303	915909	768155	1076818	441965	3332176
CFD	24	5674	4870	3855	2102	6854	1293	18541
PLG	25	48715	57719	27082	19055	35417	6518	221816
PLAT	25	13614	4906	12662	9977	16351	5840	23717
CFB	25	105776	77197	72047	60516	100331	42011	356339
ADA	25	20420	13505	17065	14901	21022	11097	79264
CA1	25	1678948	1248026	1187062	690679	2323230	323624	4764854
CA2	25	380802	232141	304094	228542	512866	66668	959948
ASS1	25	40888	102316	14030	8374	32128	2573	524511
SERPINC1	25	136731	153225	81073	40706	135987	16023	668247
SERPINA1	25	1429319	1306049	948222	684560	1562680	535046	6605363
SERPINA3	25	348035	333907	168926	114271	509579	35434	1180360
AGT	25	77248	43793	56317	46064	98824	40908	216110
A2M	25	378305	204613	316479	240265	516408	91687	998739
C3	25	551949	473219	371724	297587	558954	228123	2193669
C5	25	30980	27988	19496	15260	33404	8512	110818
TIMP1	25	10300	11822	5072	4054	13019	624	49039
CST3	25	28710	28809	14593	12770	31258	6136	118477
CST4	16	56587	143809	3390	1881	8270	571	538506
CST1	25	11215	8919	8550	4285	13494	2342	40642
CSTA	25	20810	8441	17230	15156	26305	9848	47636
KNG1	25	185719	153385	136015	96686	202899	48481	745339
HRAS	22	4199	2083	3842	3091	5093	1330	9198
KRAS	25	22794	8270	21637	17377	29524	8647	37981

PG.Genes	n	mean	sd	median	q1	q3	min	max
JCHAIN	25	112906	313800	21664	9066	52249	5673	1561585
CD4	25	40183	10532	38793	33854	44037	21799	62157
PIGR	25	642508	1005008	131366	48452	1122335	8270	4087712
HLA-DRA	25	9284	4313	7946	6713	11712	2225	19794
HLA-DRB1	25	8833	3223	8720	7018	11312	2737	16367
COL1A1	25	77021	43396	64506	56607	80436	45078	263476
COL3A1	25	24126	8937	21681	19308	23737	16600	60422
COL4A1	25	18784	6172	16439	14802	20245	11853	36921
CRYAB	25	36948	24215	30842	23658	48464	8597	127638
LMNA	25	28218	52367	7371	4649	18127	1569	225544
APOA1	25	1448824	1085385	1065137	740983	1755963	291646	5352343
APOE	25	25711	21548	21024	12442	28757	6881	114036
APOA2	25	310793	231543	261360	126137	401893	64967	895288
APOC1	25	12415	19513	5814	3914	10284	1176	81555
APOC2	23	18457	23289	8541	4155	21637	1405	83204
APOC3	25	44557	42703	27069	16434	62703	3169	168791
FGA	25	274262	252914	210107	145401	299583	78471	1328700
FGB	25	328665	296651	266316	182247	380392	31811	1556431
PMP2	25	105283	174849	53369	39664	89931	15267	869890
SLC4A1	25	105661	121549	65799	36902	149286	16805	586817
APCS	25	77747	88070	38961	21625	98575	12973	355194
C1QA	25	9371	8806	6743	5272	9481	2742	45663
C1QB	25	62770	86877	37802	27712	46478	19480	418983
C1QC	25	29578	54978	11025	6626	17746	3485	241084
C9	25	63949	51223	46243	32417	69539	18011	217800
APOH	25	143029	113166	91588	71238	191176	42657	428973

PG.Genes	n	mean	sd	median	q1	q3	min	max
LRG1	25	121049	119632	84848	59773	143027	41337	634300
FN1	25	19606	20171	12062	8484	22712	2372	82182
RBP4	25	37522	30488	27786	18656	42074	9530	151183
AMBP	25	105800	79784	71068	57459	120676	28519	347033
ORM1	25	548074	422495	338534	271500	729010	147619	1937428
AHSG	25	150029	167818	90778	67023	153616	37546	854677
TTR	25	174406	138273	132428	85959	204401	48927	711028
AFP	25	20723	6750	20161	16253	25071	9223	40574
PPBP	25	24895	12630	22857	18393	31271	6706	59859
PF4	25	11635	10908	6632	5681	15808	1454	46039
TFRC	25	71087	25539	70832	52372	85578	31334	127337
TF	25	2043917	1635703	1378945	1101320	2297334	717619	8319705
LTF	25	208992	233205	135731	55731	275906	6763	1050826
HPX	25	907746	809980	613203	442032	1066785	379270	4246500
FTL	25	17416	28283	7959	6815	10682	1471	126025
FTH1	25	23614	44818	11597	6837	15725	3760	220732
PRH1	25	50740	44334	37722	23645	54318	9075	206972
ANG	25	5895	5657	4002	2630	6359	691	27739
KLKB1	25	20384	18371	13136	11132	19999	4975	83759
SLPI	22	53607	82317	16566	6017	52447	1116	288532
C4BPA	25	56009	78360	31692	20430	39628	11220	293606
VTN	25	61031	90405	33819	22855	54493	9695	458551
CAT	25	205936	129293	160048	123226	242922	82508	570306
FUCA1	25	30101	25046	25138	18796	32623	4702	127964
PROC	25	291767	107811	284995	238151	343071	64467	544268
ALDOA	25	729996	273273	681698	580054	904387	270752	1242038

PG.Genes	n	mean	sd	median	q1	q3	min	max
CSTB	25	264299	109814	236676	184592	313437	88695	488011
ANXA1	25	131624	91095	91661	61328	182291	28987	365051
APOB	25	65754	59653	50957	31114	69507	8651	262235
PRNP	19	6132	3515	5224	3578	6970	2021	13761
HRG	25	105152	102704	64252	48849	115735	35956	444745
THY1	20	5783	5392	3381	1133	8517	520	17936
A1BG	25	76970	77833	48954	36613	84787	18178	389278
CD74	24	2675	1427	2233	1660	3606	673	5901
VWF	25	14824	3823	13362	11984	17016	9679	24855
SHBG	16	4019	2861	2674	2161	5957	726	11234
GAPDH	25	407105	231908	353091	306227	477576	143595	1278063
GAPDH	25	1496538	494947	1451962	1255813	1652858	740269	2622601
CAPNS1	25	47529	19448	43409	36830	54589	22741	117653
HSPB1	25	127457	87268	97407	47986	197163	16192	356275
CYBB	25	35395	22292	31242	24550	40013	15342	132911
RPN1	25	12150	7382	10243	8474	12542	3008	35755
ATP1A1	25	73024	26525	65673	56395	80763	42008	152958
ARG1	25	8792	4470	7298	6322	10339	4061	22482
APOD	25	48878	40794	31729	24286	63922	10043	182113
ALDH2	25	115270	147712	71870	51274	129680	9551	767561
ITGB2	25	2829	988	2611	2176	3139	1818	6333
S100A8	25	275476	286617	203280	107106	295104	27062	1175998
HMGN1	24	55485	50799	53366	11684	89435	637	203347
SERPINB2	25	18215	8294	17028	12241	20725	8601	46291
SERPINA5	25	152826	268620	28990	20696	69368	14159	1037491
CFI	25	62510	102660	33571	24007	52724	13079	538039

PG.Genes	n	mean	sd	median	q1	q3	min	max
ISG15	25	10570	12581	8412	3887	10670	1286	65487
PCCB	25	13191	7021	11389	8465	13567	5628	30105
ALPL	25	32673	64164	18033	16182	25064	11371	339403
ALPP	24	4124	3062	3503	1721	4877	983	11701
EIF2S1	25	30685	16438	28194	21389	38247	6943	74972
ICAM1	25	6672	2361	6051	5232	7651	3881	14358
RPLP1	25	42329	59420	27282	14237	37631	6835	266526
RPLP2	25	93316	116003	61592	44486	85937	12256	565492
RPLP0	25	36367	25675	32189	17602	44498	4118	119986
FABP3	25	20538	17258	13728	9621	32629	2290	77745
POLR3D	25	7470	2967	7370	5505	10640	2276	11749
REG1A	16	2540	1066	2512	1883	2878	811	5440
CLEC3B	25	26903	13458	23795	16607	31758	10906	68214
SSB	25	198097	97772	202164	125961	251149	42432	439918
SERPINA7	25	10181	7177	7640	5389	12006	2870	31055
SERPIND1	25	44713	43193	32730	19802	43495	13108	208309
ITGB1	16	11493	10831	9365	5560	12259	3418	48573
C1	25	11606	5814	11072	7307	14160	3834	25821
COL5A2	25	31687	13064	27588	25010	34284	16655	79338
UROD	25	48624	25831	45240	28166	57617	23472	149594
BCHE	25	97432	32973	84016	71330	120502	55429	178901
GLA	25	14312	3403	14017	12574	16962	6872	19703
GSN	25	252197	122899	205901	168787	281454	96422	598934
GSN	25	17439	9829	14398	10408	19879	7754	45439
RB1	25	8529	2265	8115	6767	9727	5418	14181
PGR	24	7046	4671	5444	3890	7900	1687	20489

PG.Genes	n	mean	sd	median	q1	q3	min	max
PTMA	24	162394	124220	155968	47927	243231	5319	427830
ATP5F1B	25	55601	55687	37937	27548	60272	9513	284651
C2	25	15541	17909	10372	7352	17937	4660	96255
S100A9	25	402589	380965	312514	155021	434367	49612	1486894
S100A6	25	239636	204778	165790	91050	325673	24325	936379
APOA4	25	57847	73890	31707	18137	59685	4872	361541
EIF4E	25	28822	14420	26195	19016	36089	8797	63181
ENO1	25	746821	310163	762439	495501	969860	139729	1266001
PYGL	25	9948	3696	9494	8326	12343	3422	18973
GPI	25	169216	59915	172748	116142	218186	46325	269059
POLB	25	6241	2121	5767	5126	6001	3351	11533
TPM3	25	294269	235913	267824	128078	376673	35585	1070416
HEXA	25	21355	13817	18946	11387	27993	4849	67747
EPHX1	25	37359	32986	26052	17892	35647	11547	148871
LDHB	25	363452	145318	365121	243013	452929	129561	681325
GPX1	25	79683	33630	83187	51488	101492	9428	162216
PGK2	25	251275	97200	240248	176978	275668	116335	541278
PROS1	25	20060	21549	12385	9248	19923	6240	89691
P4HB	25	121745	139977	83576	50260	144636	17524	733272
H1-0	25	45405	62434	32089	18634	44604	4064	317881
ACYP1	25	6752	2144	6258	5547	7995	3477	11628
CSF1R	25	6595	2506	6214	4904	7357	2775	12495
CTSD	25	316318	209762	273508	183915	338388	101225	1098784
ANXA2	25	215297	174766	173231	108139	256150	20216	619686
C8A	25	39223	34302	30016	21453	37780	10985	142678
C8B	25	15118	13826	11073	6465	15362	3578	60728

PG.Genes	n	mean	sd	median	q1	q3	min	max
GP1BA	24	9401	4149	8753	6615	11444	4147	20893
C8G	25	18185	16515	12990	8103	19674	4279	68343
CAPN1	25	73881	38150	62095	46824	83603	29935	169557
TUBB	25	129153	42655	124033	101571	157341	43998	210272
CA3	25	17344	12982	12103	6804	22448	4489	49945
IVL	25	21380	5713	19435	17720	25137	14025	36459
DCN	25	21930	17350	20460	6875	31059	5184	62362
PSAP	25	92821	52699	73824	58738	113429	42498	232057
HEXB	25	51449	35876	47508	26711	60702	13851	159870
PFN1	25	1022061	336870	1086506	754278	1228096	256022	1939979
BPGM	25	65655	48799	52191	30207	73130	8414	186584
APRT	25	113760	48657	110257	92684	146597	24792	194726
EPRS1	25	84353	27907	82652	70344	98112	35223	138695
CTSB	25	229100	134380	213001	131520	307939	23545	495310
LDHC	25	48404	40342	37890	24685	53159	13652	215265
HSP90AA1	25	370442	138328	358206	285092	470533	142097	698121
GALT	24	8543	2901	8824	6844	10091	2659	16364
GALT	25	10754	3363	9931	8858	12490	4609	19698
HNRNPC	25	201055	95243	181461	146444	245307	49140	393338
UQCRH	24	16321	22100	8540	6448	13116	3940	97470
LAMB1	25	35326	16905	32180	27394	37667	15698	105776
YES1	25	23436	24598	15597	13393	25160	7554	133619
TPM2	25	64114	73817	31768	18793	84151	10513	322797
FH	25	31997	33679	20308	14147	32228	10220	171463
THBS1	25	23837	16400	19157	13700	29122	7838	70363
RNASE1	24	8568	10503	4268	3035	8380	996	39368

PG.Genes	n	mean	sd	median	q1	q3	min	max
COL1A2	25	38855	14703	32767	30303	46375	22761	88726
ANXA6	25	118033	81674	91158	54654	166912	10912	303765
RHOC	25	124421	43588	126060	97254	158784	40644	224331
SERPINA6	25	40372	64457	21442	15665	37227	11056	341039
SLC3A2	25	7412	3643	7507	4865	9619	1953	18286
GUSB	25	15121	12579	11925	7117	20379	2276	63228
PFKM	25	81629	35791	67713	56840	92436	36099	172878
HSP90AB1	25	811857	379315	809304	475589	973523	307895	1917172
ELANE	25	63742	65484	40768	12744	99267	1150	269288
MMP2	25	8036	3940	6560	5613	8992	2623	19245
SOD3	25	31383	44939	11971	5416	30593	2530	186036
CTSG	25	16603	15165	11532	5329	22688	2651	65670
ITGA2B	25	80827	32664	86958	55972	91895	39317	178803
LPA	25	21827	17715	17037	13519	22258	9165	87911
PLEK	25	38952	11843	37552	31138	45372	21778	65366
CD14	25	12585	8876	9183	6729	16833	2507	36883
COL4A2	25	9654	2364	9040	8449	10424	5740	17603
SNRPB2	25	8987	3953	8579	7074	11013	2767	20490
CFH	25	283317	239452	194946	148067	262690	95245	1014947
SNRNP70	25	22914	12856	18407	15612	29254	3415	55886
FCGR3A	20	5468	4345	3554	2369	8223	1726	17017
ITGA5	25	8102	3378	7659	5491	9910	3446	15254
NFIC	25	4702	1708	4443	3887	5701	1616	8995
VIM	25	855539	867075	651806	284010	906701	52116	3990150
SERPINF2	25	43449	41696	30728	24291	43554	9761	221092
RPS17	25	63839	47204	58010	38890	82133	11443	247922

PG.Genes	n	mean	sd	median	q1	q3	min	max
GNAI3	25	8825	3677	9205	6035	10776	1619	15553
ANXA5	25	807962	396605	766249	490482	1046794	223369	1814438
IGFBP1	25	6529	4894	5488	2728	8907	1242	23192
RPSA	25	105895	49439	111580	64234	139076	19771	197184
MRPL3	25	113970	54199	97125	84522	131277	42742	283486
SNRPA	25	32518	19821	26972	19786	38100	6569	87339
ENO2	25	405711	163907	392835	297335	497703	109195	834615
SRP19	22	3664	2045	3528	2038	4761	637	7833
GSTP1	25	839301	257683	806507	716640	957577	314372	1506028
SNRPC	25	19126	12573	15532	13341	22155	7722	72296
MMP7	23	21410	29457	9767	3872	25100	990	123003
MMP10	25	12260	10215	8779	7465	11753	5062	55083
LGALS1	25	168895	106366	142774	98087	210740	48432	512512
QDPR	25	20494	8723	18099	13596	25658	8273	40641
HMGB1	25	61516	27148	66842	38968	71603	20628	143257
RBP1	25	47574	26106	42062	25035	64076	12363	114095
FBP1	25	172937	139568	138891	66136	207173	17135	544671
SPARC	25	12733	4061	12450	10632	15753	4751	24133
GSTM1	25	14679	17858	6564	4124	13831	869	61404
TPM1	24	10900	8313	8791	6622	12020	2489	40569
ANXA4	25	178153	182459	107606	95067	168729	20930	723005
ANXA4	19	7550	6599	5354	3617	10827	1292	25887
HMOX1	25	2355	1636	2030	1143	3065	352	6088
DLD	25	32901	25211	26522	17436	36224	10567	137250
SNRPA1	25	44709	21606	45554	26424	56347	10017	91087
CTSH	25	41066	37176	29949	23684	48739	9661	169735

PG.Genes	n	mean	sd	median	q1	q3	min	max
COX6C	25	16124	7248	15803	10958	19306	6862	37445
TACSTD2	25	26723	7378	23924	21004	34167	14342	40399
C1S	25	95636	64170	69055	61539	87359	42383	257834
PARP1	25	55851	21566	53148	41201	71772	21188	104375
IFIT2	25	43450	44579	23138	16542	66908	8498	207425
IFIT1	25	33752	21886	30304	23264	34575	16823	130313
ALPI	16	3094	1840	2702	1807	3520	1180	7659
UCHL1	25	324049	583689	70637	39799	274195	12789	2412778
ALDOC	25	28947	15956	28415	15322	37133	8503	63505
NUDT17	25	315557	179345	280180	228720	343864	198389	1126286
C4A	25	226044	198797	156962	127474	274794	80188	1025824
C4B	25	22427	21404	16755	12190	24241	8838	115791
H2AZ1;H2AZ2	25	67007	40451	58764	39893	79810	15222	206103
WEE2	25	91922	60871	65125	48276	132474	20355	271172
OR4E1	24	4547	4289	3532	2718	4850	445	22667
ANKRD34C	25	13611	4795	11942	9273	18039	6711	23518
ATXN1L	25	8989	3296	9286	6979	11398	1999	15563
DCAF8L2	25	11613	5729	9616	7576	15595	4731	26657
POTEJ	25	7833	2800	8057	6086	9828	1924	15123
THEGL	25	100295	33920	96756	83246	109268	55298	194192
SAA1	20	3910	3185	3150	1835	4037	871	13664
SPATA31C1	25	15844	6167	14544	12051	18988	6374	30294
HSPA1A;HSPA1B	25	357136	166264	335422	236763	404206	111042	893828
CBSL;CBS;CBS	25	68994	19672	67030	53467	80608	36028	109062
CALM1;CALM2;CALM3	25	346385	144861	341647	235257	423485	98172	790368
SCHIP1	25	35883	12229	34330	27187	43947	15586	63169

PG.Genes	n	mean	sd	median	q1	q3	min	max
AMY1B;AMY1C;AMY1A	25	27000	84885	4555	2825	11835	794	422847
FDX1	25	129658	42980	121130	102674	162359	69245	219990
RNASE2	21	3890	3390	3963	964	6332	136	13782
RO60	25	66100	31795	61190	42425	81751	24411	165006
GAA	25	27487	26144	18016	11522	31725	6714	125229
RRAS	24	9067	6634	6940	4453	11505	1076	26480
HLA-C	24	3000	2213	2298	1516	3259	718	7795
H1-4	18	28457	25853	22653	7582	39550	2579	107432
SPP1	25	10829	9628	7566	5400	10401	3102	43014
DLAT	25	28676	9155	25217	21662	32947	14477	47607
NR2F6	25	9584	4150	9339	7061	12858	2229	17551
TXN	25	608711	291119	572277	444407	720103	232523	1626679
COX5B	25	178994	141279	144726	69034	251629	35522	636789
CTSA	25	8851	5130	7481	6400	9750	2778	27369
MAPT	25	60301	18398	60082	46575	66144	26825	105640
C7	25	29534	22772	20224	13235	34925	4900	87687
PRKAR1A	25	28698	11095	28314	21034	38006	9230	48955
CHGA	23	4143	6704	2383	1547	4081	451	33694
ALPG	23	13007	14022	10208	3209	14084	956	58658
UROS	25	8026	3325	7634	5456	10318	2746	16113
ESD	25	96858	41473	92890	68837	107816	37361	215714
HSPD1	25	51052	50670	33856	22587	56016	7671	245722
CLU	25	890388	1239215	328905	230774	950064	182802	4885050
HAPLN1	25	7716	2309	6700	6203	8310	5330	13818
HSPA5	25	136776	137839	88181	69577	176233	19548	716928
LAMC1	25	29562	25159	25028	18618	31325	11452	143971

PG.Genes	n	mean	sd	median	q1	q3	min	max
ACP2	24	8250	5897	7547	4715	8815	1642	29195
HSPA8	25	526061	204212	529649	416450	667676	192777	879827
EPB41	25	16624	6416	15554	12327	19443	8411	38291
UMPS	25	7433	3145	7166	4736	8549	3604	16400
DBT	25	31745	17760	24838	18749	37086	11627	76885
PYGB	25	39430	17267	37504	22956	53411	10444	70980
RALA	25	5095	2076	4965	4119	5261	2178	13278
NAT2	25	45508	19129	40647	30564	61074	13778	78954
SPTB	25	166717	62196	148598	131167	190087	101932	346708
LAMP1	25	5206	5711	3283	2303	4082	1156	25449
TOP1	25	35451	14640	33014	25869	39028	15829	77097
UBL4A	25	7605	3608	7711	4823	9501	2222	19845
GNAT1	25	36563	22283	31805	20810	48777	7959	102512
MTHFD1	25	460944	310392	380986	277382	594083	138985	1589246
ADH5	25	92002	43988	92224	58863	111079	29313	197460
CDK4	25	12457	4038	12274	9904	15492	5823	21079
CRYGA	21	10089	5434	8985	6186	14628	2266	20744
PABPC1	25	99549	64640	87710	59778	128171	15499	248057
PCNA	25	41201	22444	31423	25333	51470	7675	86769
HARS1	25	11567	4443	12040	8399	14329	3719	20258
COL6A1	25	10835	10710	8284	5720	12356	3858	57752
COL6A2	25	31063	10924	29094	26868	33104	16925	78004
COL6A3	25	135586	43740	136270	104934	155961	80902	265014
IMPDH2	25	35835	19232	29969	20254	48275	12471	78125
TPR	25	183122	72694	156329	144151	182544	105591	423665
CKB	25	86754	79867	70358	24730	124286	7656	299056

PG.Genes	n	mean	sd	median	q1	q3	min	max
ANXA3	25	137585	78748	122000	98916	163708	21304	360158
BMP4	25	59980	36963	47037	35088	96571	13065	135140
BCKDHA	25	4090	1652	4362	2690	4945	789	7902
RNASE3	25	14535	11963	11847	4289	20619	1400	43923
ACTN1	25	147012	85570	130359	89817	196508	34019	400737
CDH1	25	36986	20823	33097	23464	42922	7441	104710
MYH7	25	14272	2692	13836	12609	14998	11262	23487
SRC	25	23057	4355	23650	19595	25565	16107	33990
PEPD	25	23421	8269	24398	16972	30783	10150	36129
XRCC6	25	96191	38203	96152	70726	118964	21647	171337
XRCC5	25	75344	31683	74493	52103	97023	24731	147597
IFI30	24	13549	10305	10683	6468	18503	1217	36755
RNH1	25	168770	55598	166433	119006	219170	71125	270262
SCG2	25	40352	23360	31107	27588	50015	15465	126528
EEF2	25	304621	121513	321743	196939	361074	108740	559172
PDIA4	25	154200	129066	110460	74659	227631	27696	648614
C6	25	16323	16377	10369	7488	15205	6228	71309
TPT1	25	46271	21934	41700	32576	57633	15162	97600
ALAD	25	83315	29975	84867	59968	92733	47454	154785
LCP1	25	163970	103811	135722	104221	183361	34661	538204
PLS3	25	121732	55673	109573	86086	138838	39946	250168
APEH	25	59662	22074	57435	44430	70399	22909	105218
ETFA	24	10340	6466	8431	6598	11719	1966	23805
GYS1	25	17280	8296	15866	12295	17536	6301	42319
GTF2F2	25	11321	4470	10060	8353	13642	5614	24899
MIF	25	1428138	630093	1222081	997536	1844360	522322	2660131

PG.Genes	n	mean	sd	median	q1	q3	min	max
CD99	25	10977	13344	7713	5210	12052	721	70755
PRKCSH	25	58231	61172	40366	27651	68607	15843	325576
HCLS1	25	3496286	1538529	3484686	2552405	4187474	1604714	8905937
FDPS	25	79122	42116	69750	57647	89250	27790	233585
CPM	23	8047	12254	3731	2062	7288	914	58389
NID1	25	33775	36097	27821	18552	31135	12312	201588
AKR1A1	25	124374	49980	131706	93191	148131	37534	272845
PKM	25	1077105	646125	1025188	759048	1140783	266905	3426088
PKM	25	355403	246994	289417	207708	401002	73148	1020388
ACYP2	25	6562	4943	5071	3868	7237	2552	26421
HSP90B1	25	263270	214449	189088	109862	355473	87034	1115987
CCNB1	25	335335	322755	250304	167519	393061	33611	1634636
IDE	25	55374	15493	51525	46059	63425	33300	93574
MMP9	25	11279	7609	9807	5550	14238	3352	37315
COX6B1	20	7877	15777	3160	1703	4571	885	62453
HNRNPL	25	61892	19812	59907	54014	74681	18741	111936
DARS1	25	37627	17455	36164	21199	52580	11977	73488
IDO1	25	19936	14196	16374	9399	26199	3576	58612
JUP	25	10551	7171	9740	3832	14631	2146	23303
CPA3	25	35722	28960	25565	18445	38596	10284	124462
FABP4	25	18311	34561	7003	4848	19419	798	178250
GLUL	25	22138	23911	12042	9324	18557	8067	93297
AKR1B1	25	127212	61679	120040	79692	152909	37471	300877
RAC2	25	33172	29068	26689	17507	42561	1230	139494
CPN1	25	47095	40216	37194	24567	54829	3640	201353
GSPT1	25	20019	8858	19560	12402	27158	5370	34667

PG.Genes	n	mean	sd	median	q1	q3	min	max
PGAM2	25	65253	27186	64304	52365	73824	22799	165685
IFNGR1	25	9062	4248	6988	6183	10901	4406	20466
EZR	25	94569	49171	90694	65517	119758	13946	230840
FOLR1	22	18801	40915	5708	2360	12793	625	190911
UCHL3	25	14036	6685	12898	8684	18512	1558	33253
FOSL2	23	13183	5054	12400	8724	18115	6366	22701
NME1	25	46416	21877	47773	32116	55686	10398	98883
ARSB	25	63136	27328	56030	47460	65335	40023	150183
RPS2	25	17830	13495	16175	8420	24676	2230	66136
ST6GAL1	25	93687	26374	94252	81793	108361	43201	146609
DSP	25	219838	138543	169298	132038	260109	103944	699013
GATA1	25	38802	12059	36004	31711	43216	16443	70132
TIMP2	25	36310	22886	28990	23188	41648	12480	119164
NQO2	25	9274	6595	7714	4819	11241	1528	27261
SELP	25	20534	8445	17043	15467	22749	12999	49113
CBR1	25	52283	22918	45751	35773	71406	12032	94697
ACADS	25	6357	1517	6216	5235	7169	4233	10371
NCK1	25	23094	7442	21650	18189	26660	10726	43435
H1-5	25	93096	44527	88902	57112	119629	17319	190487
H1-3	25	14928	11443	10212	7972	17899	2920	44440
H1-2	25	289083	120633	279837	195549	393823	37224	548271
EPCAM	25	15223	7836	12399	11572	15404	6243	41029
POR	25	85067	40743	74757	60045	87108	49561	243686
MGMT	22	5056	4283	3514	3044	5162	1629	20940
PLCG2	25	22018	8939	19758	17560	25052	11627	54975
FAH	25	24136	13740	19747	16952	28006	8594	65236

PG.Genes	n	mean	sd	median	q1	q3	min	max
NAGA	25	15617	3432	15773	13557	17100	8843	22981
HSPA6	25	116144	33608	115306	85741	144574	60528	183743
GOT1	25	33165	16194	29653	23148	37426	14583	77329
BPI	25	11147	5208	10607	8128	12921	4195	22947
PRKCA	25	52765	17660	50452	39852	62603	28629	108217
JUND	16	3385	1645	3001	2174	4557	1070	6792
CAPN2	25	67052	25065	62803	47434	85388	27310	118966
DES	25	8120	5613	6681	4758	9531	2301	27411
CTPS1	25	12416	7032	11018	7356	16954	3961	33612
DDX5	25	16925	7163	15294	11972	20817	4215	34128
PFKL	25	149111	62743	146610	111381	165598	46437	320960
GM2A	25	14962	8018	11958	9733	18364	5470	37228
LGALS3	25	97255	63125	80917	60430	110362	28238	300208
FLT1	25	121459	27307	111153	101382	138066	88962	179335
PSMC3	25	23191	9955	25225	11739	28797	8846	41458
TCP1	25	72141	32015	72036	49146	92097	23025	135401
PTPN1	25	12713	5382	11542	9250	13614	7319	31886
IGFBP2	25	13220	12419	9513	5088	13926	2365	43175
RPL35A	22	8545	6095	6184	4333	9885	2036	26448
ITGB5	25	31115	21925	23710	19824	36222	14865	123910
ARF4	25	63192	28489	61613	44849	78034	18129	117980
RPL7	25	50115	34228	33093	23157	73173	13273	147992
VCL	25	185481	97495	171764	113155	203259	40938	382966
LBP	25	7069	6871	4538	2564	8670	1645	31783
PTPRA	25	28633	11054	27058	22969	35230	8983	60107
NAT1	25	6722	2236	6527	5943	7355	1701	13411

PG.Genes	n	mean	sd	median	q1	q3	min	max
PGAM1	25	181411	54368	185060	159946	207935	47144	295762
SDC1	20	7678	7444	5465	2852	9758	1530	33642
XRCC1	25	27535	7435	25120	23630	30077	19155	56574
TNNI1	25	10184	6320	8841	6532	11819	4250	30994
NCL	25	206012	117265	183298	126568	238232	39696	522823
POLR2E	23	3896	1485	3622	2969	4966	1503	7409
NDUFV2	25	17613	9296	15689	12982	19263	8866	57508
TNFRSF1A	25	15837	21444	9882	4160	14247	1767	90950
TRIM21	24	8937	2918	8071	7086	9822	5058	16696
EIF2AK2	25	21626	10545	18147	14731	22951	10638	54577
SRM	25	27151	13828	30164	15064	36874	4766	56081
ORM2	25	151164	145985	104945	78606	158234	51968	793566
CSNK2A2	25	142395	46602	136186	116525	160970	56036	249779
ITIH2	25	142323	122577	105365	77282	142040	40892	574746
ITIH1	25	69013	56330	50661	38345	70085	20965	271031
CEL	25	16383	8200	13449	11698	18843	9050	46377
NFKB1	25	25990	8007	23010	19673	30715	15664	42583
NCF2	25	7188	4656	5196	4244	8575	3274	22752
PI3	22	3865	1520	3737	2947	4701	1326	7875
TYMP	25	64051	74546	51285	33882	64581	5537	394803
EIF2S2	25	17778	9037	16319	10996	21055	4749	40340
ANXA7	25	35277	29768	28206	18862	41844	5836	138041
AZU1	25	31016	27531	26562	8342	40037	3037	101423
TPSB2;TPSAB1	25	33877	17745	29211	24551	40792	7558	80900
BTF3	25	11992	6408	11223	8086	13308	4405	31975
RAB3B	25	12243	7240	11168	9473	13691	5721	43944

PG.Genes	n	mean	sd	median	q1	q3	min	max
RAB4A	25	30067	11761	26917	21066	35481	14599	61778
RAB6A	25	21751	5647	21198	18049	25366	9988	32583
MX1	25	23681	13899	19434	15726	27019	8267	79283
PSMB1	25	67861	25967	62093	52192	82356	24341	118490
COX5A	24	2794	4256	1798	1349	2598	232	21752
LMNB1	25	7758	9610	4301	3591	6139	2923	41187
ITGAX	25	38174	8495	35395	34366	43264	24896	63050
GZMH	24	22209	14160	18661	13596	30370	3546	50969
PZP	25	230595	153239	189821	158860	261984	62026	853089
OGN	25	34291	20976	26778	18779	54172	8558	70813
CAST	25	51106	22621	44481	36357	54098	29926	131760
EFNA1	25	27850	16635	22775	15320	34787	4365	78844
C4BPB	24	7652	5330	7089	4635	8936	708	25078
AGA	25	17295	15337	13386	8100	18801	3018	63342
RASA1	25	13800	3064	14102	11564	15716	8304	21207
PTMS	25	134058	91690	113391	64897	171620	12984	331787
PTN	25	2411957	1848761	1825794	1057694	3291518	501622	7053250
GSTM3	25	77235	58886	51081	33615	110995	11565	218198
ATP6V1B2	25	21889	9572	21084	16180	24347	7090	44112
ATP6V1C1	25	6181	3335	5372	4661	7374	1933	17376
CSRP1	25	83446	73409	51348	34239	128713	4487	267727
ACO1	25	23310	9799	22348	14912	30374	8982	44436
S1PR1	25	8136	7251	6825	5078	7670	3079	40376
NT5E	25	45926	26266	37204	29105	48747	20844	131015
EPHA1	25	115276	81609	92827	71818	143850	30062	433883
MDK	25	22382	19683	16893	8778	29880	1143	84071

PG.Genes	n	mean	sd	median	q1	q3	min	max
ZP3	23	107843	65590	105663	68810	120524	42796	343116
VDAC1	25	26892	13942	22598	17548	31791	6317	65622
BGN	25	4696	2284	4441	2656	6622	1048	10190
CD9	25	79753	58616	64487	41287	110645	18381	281190
BCKDHB	25	21556	7454	22436	14715	24482	9525	41379
COMT	25	13683	11046	10317	7082	17047	2688	52784
TGM2	25	129900	195344	33506	11456	127253	5565	719514
MMUT	25	12619	4613	11669	10178	14088	6723	27377
OSBP	25	25655	9572	23530	19259	30958	9981	44042
PCMT1	25	36748	10952	34666	29163	40872	21235	68906
FBL	25	35518	16938	31330	23840	40148	18318	78522
GART	25	17565	9120	14892	10771	24059	5063	43430
PAICS	25	48430	30124	42997	23807	66540	8467	114428
GPX3	25	16934	13083	11751	8644	22008	4373	52856
NME2	25	444948	193312	417523	343534	603466	126704	815345
ENPP1	25	197922	129783	161906	131823	200192	71764	729778
HNRNPA2B1	25	406776	280539	347962	239436	506866	57775	1327929
RFX1	25	13291	5315	11585	10139	13724	8733	32037
C2	25	102438	47843	89429	76589	109352	47182	242613
CBL	25	20459	6327	17924	16742	23387	10457	34031
IGFBP4	25	3749	1527	3435	2820	4193	802	7075
UQCRC2	25	3590	2216	2787	2402	3617	1498	10572
CPN2	25	108187	69576	91281	59739	128270	34023	344753
MMP8	25	86072	26801	80509	72978	93757	44110	164219
FBLN1	25	18932	23269	14466	7004	21358	2729	116923
FBLN1	25	101453	154704	48914	20522	93669	3856	617816

PG.Genes	n	mean	sd	median	q1	q3	min	max
TCEA1	25	62519	24366	58136	45688	72614	23346	123940
SFPQ	25	58049	37721	54937	28786	69523	7514	179067
PPIB	25	74999	55001	56160	37373	97507	12576	270126
S100A1	25	108152	117630	72937	43673	94793	8678	503482
HRC	24	4615	2160	4530	2991	5562	1932	10992
ME2	23	6813	4554	5487	4082	8312	2136	22458
WARS1	25	45932	38509	36583	27443	48443	9437	211243
RPS3	25	80217	38439	83518	45074	109334	16996	148040
GCSH	24	18843	10679	14139	11082	27312	5560	38976
SP100	25	141707	29015	141105	132767	155820	85013	224853
AHCY	25	220224	79590	212610	154107	275322	93354	425546
CFL1	25	858026	299614	836649	677895	1013102	206730	1404067
PAX7	25	60204	11493	59508	51073	70483	37784	83059
CPT2	25	23165	8185	22272	18497	26049	10338	44622
DTYMK	24	13216	12544	10364	6356	14363	2210	63186
RRM1	25	21358	11015	17123	12580	25117	11202	52104
CMA1	25	14467	13262	6702	5767	24255	3778	57760
LAMA2	25	68309	12507	65960	61543	68681	53705	113628
PRTN3	24	21068	19746	16870	3847	34607	839	73301
MMP11	24	5651	4146	3575	2846	8273	1771	17916
CHM	25	9187	6430	8178	6769	9393	3680	37806
NR2F2	25	27466	15867	24579	18656	33897	4118	64453
EEF1B2	25	74119	46228	65123	45239	103976	13852	170330
IGFBP5	25	17788	7116	16138	14231	19730	5955	42089
ACP1	25	85253	27224	84775	64808	106044	43201	145316
ACAT1	25	118565	58853	100051	82036	151214	45606	280541

PG.Genes	n	mean	sd	median	q1	q3	min	max
TNC	25	36215	19006	29483	23483	46963	10015	77488
POLR2A	25	265347	112959	244592	222749	271413	176591	784310
CDK2	25	7317	4108	7060	5331	7716	1225	22733
GRK2	25	14584	3100	14450	13147	15434	7010	22112
AZGP1	25	74915	64404	42342	39114	92332	27640	329584
MPST	25	36371	15049	36223	25889	44010	13100	66408
LAMA1	25	363104	131467	314720	298059	420112	199057	816799
RPS12	25	62073	32836	66763	36447	75425	14072	151885
YY1	25	8075	2309	7744	6775	9634	5015	13787
DNAJB1	25	183329	50759	193892	146781	216180	88986	276450
DNAJB2	25	40630	15394	37112	30132	48493	15802	86604
ATP5F1A	25	37276	24013	26466	21472	46682	10661	103048
MT3	20	56077	22714	57656	47818	62724	10406	119428
PSMA2	25	40338	17358	37391	30526	53252	12420	73172
PSMA3	25	72355	29410	69879	53605	90758	25632	141151
PSMA4	25	65821	30154	60060	48162	81416	19746	152698
S100P	25	79472	55640	66341	39072	121289	6505	209487
COL5A3	25	248042	76790	224303	209229	261426	184797	576371
PTX3	25	8789	4561	8466	5308	10088	2752	21736
MSN	25	183721	62563	163449	146056	227296	63153	304883
DDX6	25	21811	8633	21000	15875	25309	10517	42485
U2AF2	25	43695	30448	37428	29744	50018	4820	144468
RPL13	25	11978	11285	9579	5770	13252	811	58037
CHML	19	4875	1755	4709	3473	6105	2252	8568
IVD	25	140626	61976	133274	110477	152617	50114	292092
S100A4	25	96862	64545	69590	58354	117124	37778	284410

PG.Genes	n	mean	sd	median	q1	q3	min	max
MGAT1	25	4059	1957	3670	2705	4474	1957	9813
HMGB2	25	53016	28881	47297	34294	66188	7027	136620
PTBP1	25	46453	33625	39252	27807	52921	7612	147745
VARS1	25	21114	12655	21011	8717	29175	3501	50701
EEF1G	25	175416	98398	162942	122821	233837	42042	400453
FKBP2	25	68916	54781	53878	34611	78148	11655	286447
MST1	18	2757	1980	1881	1325	3792	1074	7971
ACVR2A	25	1268510	383538	1194199	1034172	1446487	832427	2604636
STOM	25	17052	6235	16243	12181	20126	8932	36092
AK4	20	10172	8192	6863	3774	16025	1094	30435
PON1	25	16652	16318	11876	6607	17327	2584	70751
YWHAQ	25	164230	51532	165484	134597	205945	42376	244496
MAPK3	25	235375	66220	232219	173033	282808	115214	347326
CALML3	25	452003	211801	409931	352868	458249	184306	1166331
DPP4	25	23630	10144	22465	17615	29388	7508	52424
RPL10	25	13936	8780	11643	7266	16588	4096	36567
RPA1	25	48331	22019	50059	29597	57344	14890	113075
APEX1	25	175261	75017	175708	130168	212075	41366	411755
DCK	25	20461	14652	17920	9557	25781	2810	66832
CAD	25	9009	2037	8561	8049	9498	6114	15422
CALR	25	189764	205314	136615	86304	207053	32093	1087938
MAP4	25	210125	105587	186092	156002	230283	80020	534113
CFP	25	31416	30156	21992	16501	28446	7523	125442
ITPKB	25	42811	9658	42613	37127	46410	26505	73666
PSMA5	25	42405	16930	41496	32786	46845	16337	89070
PSMA5	25	37716	16407	36630	25119	42599	17018	88625

PG.Genes	n	mean	sd	median	q1	q3	min	max
HLA-DMB	25	6390	2336	5946	4939	7284	3031	12842
PSMB4	25	37200	15928	37652	28971	45348	11795	82576
PSMB6	25	58445	35504	49785	35606	73838	20345	178061
GSTM2	25	12957	16537	6802	3692	14917	1869	70153
TMOD1	25	13124	8111	11508	8105	14641	2339	33987
TEAD1	25	12150	2479	12318	10085	13687	7566	16395
MAPK1	25	41128	12777	39083	32944	50517	13431	68258
GCA	25	23635	15375	24001	9480	35211	2051	63685
ERCC5	25	73747	20567	66516	63595	75776	51317	148462
GRN	24	5273	3068	4861	2806	6469	1646	14307
GRN	25	8183	6838	6517	5105	8007	2282	37276
PTPRM	25	1970734	538137	1768661	1555497	2093329	1365065	3045492
S100A2	23	36838	64535	25815	6805	36747	1902	322233
GTF2E1	25	2553	763	2234	1989	2802	1781	5158
GTF2E2	25	4972	2573	4489	3722	5236	2231	15636
PCSK1	25	28600	13396	23798	20621	28663	13566	63773
TPP2	25	15375	7108	14215	11446	17537	5280	37658
IMPA1	25	23500	11145	22250	16727	25672	9791	60177
EPHA2	25	43339	27226	34266	26932	57004	15959	135120
EPHA3	25	62357	11832	63368	55012	71801	37582	91079
CRABP2	25	26577	34714	12580	9968	21923	4737	138561
ARID4A	25	35410	11060	34613	27472	39727	22078	75461
CASP1	25	20618	15285	15857	13264	22927	9473	83085
SERPINB3	25	84401	99886	43026	30683	90671	15435	425221
LMOD1	25	10181	4103	8239	7111	12408	5055	24263
RBMS1	25	39883	8854	37835	34219	43689	25491	65104

PG.Genes	n	mean	sd	median	q1	q3	min	max
SERPINA4	25	19519	19743	13085	10163	23131	6392	103600
EEF1D	25	117358	80010	104300	62521	144052	17991	334294
EEF1D	25	36294	32597	25362	21485	44827	3943	166493
CRABP1	25	60593	39876	50129	36947	71240	25656	201687
MARCKS	25	19006	11902	18371	8009	26807	3988	48491
ALDH4A1	25	125639	52976	123966	94907	142435	64389	325984
PBLD	25	7907	3416	6613	5364	9305	2995	15945
ERP29	25	85609	87661	63532	43345	92658	20489	459236
PRDX6	25	791465	298165	764287	544156	954812	369113	1493437
BLVRB	25	217033	128781	163180	109572	276457	61353	489347
DDT	25	57463	31454	50839	34632	64103	20251	167897
PRDX3	25	40251	64515	20923	12570	31733	2405	283176
ATP5F1D	23	24059	46373	10818	7575	19181	4651	231874
RPL12	25	25463	24547	21160	11919	28213	2810	130214
ECHS1	25	37395	43817	18995	14496	38396	5229	199697
CMPK1	25	59138	35750	50935	35540	61086	14016	151961
PEBP1	25	372404	161648	374210	279012	383214	138365	735832
PDIA3	25	199647	169981	143587	98564	259297	34900	845976
PPP2R1A	25	38491	12968	40521	29974	47885	15042	62303
CDC27	25	956488	365858	814408	696654	1265006	537789	2060914
NKTR	25	248398	43027	238356	226943	268223	166215	372690
NMT1	25	8763	2720	8351	6183	10071	5234	14008
ADSS2	25	24949	13495	22290	15887	29388	8307	66837
LRPAP1	25	63816	26326	60138	45972	71321	38088	157498
ADSL	25	8370	3138	8080	6428	10331	3200	15532
ADSL	25	17859	5033	18531	14630	20426	8773	29596

PG.Genes	n	mean	sd	median	q1	q3	min	max
CLIP1	25	87360	17393	80505	76362	91567	65031	130181
GSTT1	25	15179	7806	13324	9269	18823	4287	35735
SERPINB1	25	131436	92231	119479	76657	181373	22795	424154
ALDH1B1	25	7582	4566	6521	4108	9087	2215	21350
ALDH3A1	25	42829	16900	38967	30691	47651	20180	90460
POLR2B	25	58808	13794	56045	46578	68873	36990	87886
SDHA	25	145695	41789	138524	118511	157832	94218	260775
CORO1A	25	98349	71199	80323	55128	113943	13719	328480
GDI1	25	45548	18164	42478	35125	58432	12543	80749
S100A7	25	34821	24547	25881	19166	42928	13356	100552
MAT2A	25	37926	13631	36673	30057	45254	16439	71004
PRKAR1B	25	4838	5426	3466	2440	4815	1377	29098
PRKAR2B	25	16046	5465	15518	12299	18091	10016	32392
CPS1	25	39140	9935	38354	30502	45427	27225	68022
HIVEP2	25	229947	52449	214166	191318	248767	158842	362236
DNAJA1	25	7378	4050	6611	5182	8515	2539	23151
AKT1	25	17912	4802	17311	13653	21164	9765	25869
UQCRC1	25	25371	17157	20332	16907	24910	9773	78913
HIBADH	25	18088	14163	12601	8373	22880	4906	70093
ATIC	25	83497	38007	85024	59138	107295	13644	145436
HNRNPH3	24	9254	4663	8957	5753	11487	3025	18207
HNRNPH1	25	82843	57477	76230	54824	97414	7996	249477
CASP14	23	3131	2510	2486	1649	3810	567	10491
SFN	25	23586	30374	14462	9332	23546	3018	153097
STIP1	25	155425	54638	138924	120727	196613	61063	254812
S100A11	25	401182	267620	362651	253030	485399	64274	1240391

PG.Genes	n	mean	sd	median	q1	q3	min	max
CEACAM8	25	9214	3035	8852	8010	11330	2106	14996
PRDX2	25	623981	264966	611428	400808	725810	244163	1340908
CDA	24	8646	6208	5660	4101	12451	1944	22554
DCTD	25	11247	8072	9674	7104	11600	4534	42053
GBP1	25	19485	16990	12360	8665	22641	3770	84036
GBP2	25	43899	23281	38213	27014	51967	11902	101351
RPL9	25	18999	8675	17751	12568	22680	5304	35170
KIF5B	25	70560	27692	67147	56372	89259	26561	141843
CSTF2	25	11704	4057	11098	8937	12724	6267	23059
DUT	25	14955	7239	13861	8448	18233	7079	31465
MCM5	25	114102	60939	99054	87747	132715	53291	354260
GALNS	25	202168	141769	170963	138328	231738	49704	806080
RNASE4	25	4317	2262	4170	2640	5135	1743	11925
SHMT2	25	82207	52053	70314	43748	98895	26066	224992
EPHX2	25	18669	10613	15331	11419	23661	7664	46675
HSPA4	25	49660	20710	48534	39134	67167	14200	87598
GRK5	25	13662	5295	12875	11259	16056	4909	26063
MPI	25	20953	10718	18533	15508	24685	6271	56123
GPC1	25	95581	21408	95817	79243	112198	55506	138561
PFN2	25	8637	4495	8344	5903	10643	1907	24169
PFN2	25	28848	14480	28510	16456	38075	6492	60291
CA8	24	14102	11751	12077	5052	17157	1795	49236
CTNNB1	25	36134	36514	26513	15012	42731	6111	176621
NOS2	25	18176	6247	16900	15534	18954	13049	45489
SERPINB6	25	27616	17131	20021	16828	39884	11434	74040
RPA3	25	16870	6740	15311	12945	19846	6604	37443

PG.Genes	n	mean	sd	median	q1	q3	min	max
RPL22	25	38691	22230	33083	28658	42284	13897	117949
GTF2F1	25	25220	7854	23331	18975	28691	16872	50861
SPR	25	250328	112043	232602	169549	310864	91378	505626
SPRR1A	25	111523	130664	70579	49919	126640	22926	566953
THBS2	25	8877	3170	8309	7330	9608	3774	18614
THBS4	25	153149	92039	129610	102112	170458	75609	501350
HOXD13	23	13632	4877	12334	10369	16628	7164	26078
IDUA	24	6860	3061	6198	4687	8298	3319	17541
SAA4	25	34116	42600	20869	14458	30597	10562	219992
FBN1	25	433588	91995	422872	350486	500819	300625	682135
PCK1	25	47302	35799	38863	25796	53581	13895	177868
AGL	25	17896	8440	16264	13744	18758	5081	41656
MYH9	25	79256	58451	60771	41472	88751	23754	253337
COPB2	25	58539	43825	44689	28839	68969	17704	198800
ADD2	25	42200	25489	36932	27556	42866	20483	133871
GRK3	25	56707	19951	56688	43193	68916	20743	95828
FUS	25	70431	35006	61549	49586	82786	33785	202107
DEK	25	115360	45884	128761	83016	145452	22648	190093
GLRX	25	34498	21974	26410	23182	37404	10102	107332
HMGCL	25	20710	13886	16836	11813	24180	5878	65898
PSMC2	25	14596	7207	11917	9988	21901	3451	28152
PSMC2	25	25103	11254	28690	17135	32926	5847	44887
CHI3L1	25	7872	10008	5422	3455	6580	1450	51863
ARL2	24	8471	4492	7490	6042	9568	3667	25786
ARL3	25	16310	10201	15852	7803	20499	3984	43392
TRIM23	25	9485	3968	8039	7025	10413	4792	21259

PG.Genes	n	mean	sd	median	q1	q3	min	max
MAP2K2	25	27905	7845	26649	22406	31816	12306	43174
ATP6V1E1	25	9602	3742	9249	7313	10807	4328	18826
CPOX	25	123618	28862	116884	101842	146503	78391	176034
RPL4	25	59244	27920	53724	35807	72760	23191	134741
PGM1	25	75079	33981	71981	42096	94729	17874	141065
GNL1	25	5788	2872	5258	4528	6049	1971	17264
SERPINB5	25	19034	7998	17388	13982	23776	7044	40359
SERPINF1	25	45768	28813	40087	24603	64780	14249	121929
DLST	25	54266	53706	40830	29627	52513	15871	288172
GMPR	25	10686	8202	7772	5735	11626	2856	34633
CFHR2	25	36026	22355	31088	19041	44860	7509	90337
SRP14	25	43334	17778	40281	32876	52294	5494	78866
TGFBR2	25	31818	9454	28899	24865	37572	18788	50622
HPCAL1	25	12995	5033	11802	10309	16140	5952	26998
TALDO1	25	369110	131174	363819	258811	476465	126987	624595
SNCA	25	27273	19740	21603	10598	42518	6175	81271
COIL	25	28637	10517	26756	22116	34149	10371	56191
HSPA9	25	47917	27370	40966	30278	57550	11302	119863
EIF4A3	25	20830	9129	18687	15304	27705	5894	41935
RPS19	25	24076	14268	22108	12187	32359	6485	64851
RPL3	25	25650	12982	23054	17892	32340	7328	63772
COL15A1	25	115806	24194	111969	101762	133940	73660	180970
ANP32A	25	133413	82851	120710	78456	152560	24917	417042
FEN1	25	22116	11474	18392	13385	28378	8751	50906
CUX1	25	4070	1019	3812	3248	4733	2571	6313
MMP12	25	49424	19155	48326	37277	57527	15074	110586

PG.Genes	n	mean	sd	median	q1	q3	min	max
CAPG	25	63062	54008	54445	37272	65798	20989	299971
CAP2	25	506531	219571	452949	320819	573429	260978	1063211
CEACAM6	18	6891	7411	4278	2744	8486	920	29559
CD96	25	31378	10315	31658	26953	40345	10072	50841
TXLNA	25	12266	4495	10773	9274	14189	6813	26122
CCT6A	25	129860	52911	126105	96105	178551	43490	221134
NNMT	25	35175	30312	25427	13899	45854	7634	119832
PSMB10	25	14085	10782	9869	6690	17462	1293	41756
PBX1	21	3358	2296	3521	1393	4447	251	9829
RPL13A	25	24517	16054	21154	15569	30088	8401	88674
ARL1	25	13917	8882	10515	7389	17380	4564	39231
STAT3	25	281874	178117	198936	162158	433733	99717	718555
USP8	25	10376	3848	9364	7975	11038	6740	22908
PEX19	25	9724	5191	8890	6226	11609	4057	27483
MDH2	25	179343	117155	138906	99196	247689	31708	578966
HADHA	25	19249	10657	15863	11381	21005	9004	48557
EIF2S3	25	35522	19596	34055	22708	42640	7831	87206
OPRK1	24	31293	32795	17108	11529	43080	3347	149928
CETN2	25	4613	1314	4578	4071	5225	1534	7084
ETV6	25	18572	7786	18036	14381	21347	7728	44992
EIF2D	25	12405	4186	12020	10179	14859	4692	21165
MNDA	25	18062	14917	16221	7519	20349	4253	71954
RGS2	25	17198	12365	13797	7977	21839	4931	50718
PTGDS	25	9287	7654	5587	3962	11977	937	30482
UBA7	25	9425	3360	8600	7257	11078	4468	18255
NAA10	25	4807	2282	4761	3670	6492	1219	10566

PG.Genes	n	mean	sd	median	q1	q3	min	max
PPP1R2	24	6753	3952	6518	3978	7910	2338	20174
CSK	25	28012	17152	24246	20149	30481	10538	100422
GARS1	25	33651	16451	32937	21470	40178	5972	64807
IARS1	25	26508	14294	24497	15261	39063	7210	53357
MAP3K8	21	3540	1929	3687	1884	5237	1120	7722
EIF1	25	5568	3682	4618	3191	7910	1352	17174
PRKCI	25	63405	43252	53587	30262	83269	8560	180505
ACTR1B	25	25845	8293	26585	20963	30489	10455	46292
TMPO	25	53698	28202	54804	30123	74763	6744	113075
STAT1	25	32559	51355	16624	10462	30036	6889	259446
STAT6	25	37816	31213	33179	20777	38432	16483	173039
STAT5A	25	27315	9213	26311	18842	34742	13650	41201
MTREX	25	7666	2666	7536	5759	9295	3839	14445
AKR1C3	25	37469	36063	23260	14367	49324	4999	150206
EPS15	25	12325	4632	11791	10065	13232	2295	23774
CASP3	25	83985	21676	85304	69856	99919	42234	124883
TEC	25	12259	6562	10007	8419	12970	6670	37245
NCAPD3	25	16394	3512	16104	13978	18823	9379	24275
RBM34	25	209808	142678	160425	114918	282405	51162	720483
LIFR	25	16703	5796	15357	13881	19810	7396	30850
LRPPRC	25	43763	17147	39928	32712	54285	18233	84125
ACAA2	25	16979	6291	14643	11894	22756	9002	28760
RPL35	25	12535	9363	9905	6838	16659	1445	44848
CDKN2C	25	8928	5260	7874	5257	13276	2713	21856
PRCP	25	18674	3740	18697	15964	20937	12677	26019
HTT	25	45303	18209	37856	34011	51127	30178	105952

PG.Genes	n	mean	sd	median	q1	q3	min	max
PAFAH1B1	25	35689	13112	36074	28294	42134	10315	69389
PTGFR	25	9038	6049	7449	5251	10158	3675	27726
MCAM	25	15149	7642	14146	10088	18784	4469	39842
CRAT	25	20094	8970	18976	15835	20836	7974	53964
MATR3	25	36356	20093	29961	26464	40217	7078	95157
ZAP70	25	21905	8500	21653	15128	27626	9954	40262
SYK	25	20696	8429	18028	16351	22292	12891	52020
NAMPT	25	41493	27840	31755	22218	50326	14150	123423
AFM	25	58151	49216	43603	29269	50987	14388	242005
PSMC4	25	12636	4850	13565	9166	14854	3627	23392
ASPA	25	11351	11498	6501	4923	10648	2685	46577
PPIC	24	8140	5047	7365	4641	10285	976	20492
CBX5	25	8842	5656	7935	4808	10707	2129	27217
RANGAP1	25	14523	4590	14394	10989	16521	7425	26518
RECQL	25	29785	17185	29795	16446	38785	6491	77021
GPR4	24	159503	114885	150727	47224	257649	2428	393021
CRK	25	8590	3546	8153	7066	10237	2873	19011
CRKL	25	13671	6612	12180	10156	15984	4272	28658
BAG6	25	6156	1807	5902	4905	7127	2920	9858
GSTM5	25	35957	16350	31574	27005	42872	15879	75734
NSF	25	23750	13205	17988	15456	28051	7846	61090
CDKN1B	25	48772	10179	46668	43085	54994	27565	68976
RPL27A	25	15041	10001	13724	8936	18570	3169	55587
RPL5	25	29509	11312	28648	23302	37572	6507	48626
RPL21	25	43754	29601	35087	20249	54708	9517	117577
RPS9	25	21463	14629	19663	10544	32700	2669	64245

PG.Genes	n	mean	sd	median	q1	q3	min	max
RPS5	25	11370	7923	9074	5824	15951	2180	36887
RPS10	24	15297	11786	12512	7178	22462	1829	54624
MAP1B	25	209000	89813	177711	163364	221045	124579	446572
GNPDA1	25	43437	18271	39933	29009	49395	21189	92753
IQGAP1	25	81679	22529	81784	67745	95888	27368	120090
HAAO	25	7886	6358	5202	4055	8853	1706	25635
GYG1	25	14018	6104	13014	10303	16569	2451	29137
RABIF	25	8418	3372	7648	6279	9323	4292	20567
PLA2G4A	25	138730	62106	134511	91942	165690	43036	351402
RAP1GAP	25	13586	9344	10705	8798	13708	971	38031
CAPZA2	25	102374	45995	95679	67962	139341	32508	207232
CAPZB	25	83786	37563	77524	55223	99012	28470	188477
EIF1AX	25	15327	5941	16615	10991	19053	2738	26806
ALDH1A3	25	15859	4994	13836	12461	17468	9324	27245
QARS1	25	96003	38521	91973	75973	109188	30258	205188
HTR5A	24	8540	4786	7101	5105	12123	512	22879
CDX1	25	21838	18727	15233	8883	30233	1924	82095
RPL29	25	21392	5264	21256	18350	25340	9092	29158
XDH	25	28980	23091	24067	19892	29453	11258	135016
ATP5PO	25	12941	3881	12711	10004	14308	7194	23872
GRIA4	25	44981	32187	36515	27274	47347	13728	182489
LIMS1	25	22001	11926	18715	13198	28703	7199	51824
PREP	25	35044	16967	32630	24026	40573	8010	82637
ME1	18	7353	2578	6744	5882	8390	4249	13484
IREB2	25	24354	8126	23741	19394	27586	14674	55527
RFX5	25	168812	65415	140370	124649	210753	82220	310486

PG.Genes	n	mean	sd	median	q1	q3	min	max
ARCN1	25	130349	75800	125504	65895	175571	29308	303758
GCLC	25	74177	56419	65055	52336	74447	15432	326624
GCLM	25	7809	2474	7959	6192	9346	3731	13677
PCP4	25	6386	6367	4325	3173	6340	1033	31370
NRIP1	25	49081	9938	47347	42341	51415	34000	77661
PSMD8	25	49429	17342	49653	34777	60020	17245	90308
SERPINB4	25	18024	7959	15745	14334	19182	8160	43761
SERPINB10	25	35290	19321	29838	23876	40173	16372	98058
PRRC2A	25	69380	13329	66871	61948	74301	48792	110319
GSS	25	70724	22834	68560	55547	86784	31256	111533
CCT5	25	44532	18891	44281	34046	53905	15197	78207
NES	25	17459	6952	15852	13799	19531	10165	46278
HSPA13	25	16488	9921	15312	9624	18542	6437	51585
IDH2	25	132566	96966	123548	70773	159322	25583	500938
LHX1	25	68703	76592	51995	37624	72129	30565	424619
ADGRE5	25	8509	4029	7403	6433	9611	2431	22462
POLD2	25	47801	11941	43461	41066	54887	31232	77538
MARCKSL1	25	13507	6356	11571	9451	15845	3271	32896
MAPKAPK2	23	5470	2130	4921	3898	6816	2347	9228
ALDH9A1	25	23176	23139	13884	10759	32162	3651	117835
RPL34	25	7152	2944	7620	5549	9345	921	13751
RPIA	25	12872	5078	12478	9271	16720	4753	24246
NASP	25	46590	23537	39401	28305	59066	12729	99202
FASN	25	107848	97610	89831	32824	140988	18858	439499
FNTA	25	8261	2796	8454	5705	10181	3351	13201
CCT3	25	92052	42115	93640	68369	118881	32732	176926

PG.Genes	n	mean	sd	median	q1	q3	min	max
TUFM	25	47890	28279	47031	31941	53460	12490	145052
ALDH7A1	25	36223	20177	35072	20773	44387	5354	77378
SRP9	25	68589	37690	56917	47655	83097	17185	179349
UBE2A	24	6717	3291	7201	3839	8127	1626	15154
AARS1	24	16411	8110	17204	9012	21511	2687	32499
AARS1	25	31245	11233	33414	23715	38576	9652	48780
SARS1	25	146945	57784	148099	107322	169444	39744	258239
PPM1F	25	19261	7948	16226	13856	23023	10557	41357
PRIM1	25	34623	20183	29726	20565	41781	13381	92798
PSMB3	25	17189	7361	17964	10671	22420	5096	29401
PSMB2	25	47698	19517	46084	33646	60945	17327	86634
MCM2	25	38255	17751	36863	25766	48152	14733	71934
COMP	25	6487	2556	6130	4984	7474	3389	13331
ACADVL	25	21663	8336	19646	14750	25404	11935	41393
YLPM1	25	116959	26596	109097	94606	128907	89833	170407
ACOT2	17	8652	9487	6868	2225	9465	139	38979
TMED10	25	25438	12536	23730	19908	26789	10169	70601
RBM25	25	76249	29552	68978	56602	89562	43367	183305
NUMB	25	397639	222198	343117	294354	407658	146198	1147452
HINT1	25	20353	9630	21101	12498	26071	8755	51707
FHIT	25	6637	3733	5066	4485	6714	2585	16821
NUP153	25	42154	16430	34089	32385	46829	24785	86502
RANBP2	25	48679	12168	46240	39140	58501	30732	76505
NDUFV1	24	5994	4182	4725	3734	6958	1206	16963
GSK3A	25	7725	2656	8226	5162	9670	3319	13919
SEPHS1	25	33550	17989	31282	22872	40025	8196	87938

PG.Genes	n	mean	sd	median	q1	q3	min	max
SELENOP	22	5028	3848	3566	2386	6667	994	16351
CAMP	25	12907	9360	10551	7113	15279	2716	37600
GMPS	25	102023	45192	97098	78389	113033	32385	236682
LIG4	25	80113	18284	73131	67223	86420	55947	130561
HNMT	25	17365	8337	16348	10903	24019	3956	34437
GNAQ	25	43863	21781	36843	30097	50877	19873	117849
IDH3A	25	28076	14522	25003	18826	35298	5802	72455
MEOX2	19	10149	4537	9644	7272	12473	3076	21094
SULT1A1	25	17114	8491	16357	10290	19507	6728	37075
CRIP1	25	51390	32311	44164	27684	59561	10974	158904
MMP14	25	58465	30462	48996	40704	66818	30872	178435
GDI2	25	219411	81925	224973	157280	251196	67834	390038
EMD	25	11619	14310	7918	6079	9835	3857	76034
SERPINB8	25	76399	39806	58723	51140	82491	34449	178703
SERPINB9	25	22345	13344	17511	14060	26159	9255	63122
SERPINH1	25	59743	37907	47584	26725	82588	16546	148641
LHX2	25	6690	3641	6779	4717	8772	1768	16293
PDLIM4	25	9447	4500	8024	5957	12131	4233	22931
ST13	25	150087	55088	138110	121687	156563	73054	345742
ERF	25	8059	2573	7589	6992	8901	4028	15439
VASP	25	12291	5307	11989	8265	14999	4066	30397
METAP2	25	38525	21283	30835	25068	45265	17126	101574
METAP2	25	10712	22123	5647	2066	9089	915	113733
NUDT2	24	6707	3156	6504	5014	8835	1097	12062
TNFSF10	25	279410	87928	279739	231639	328007	98417	471310
HLCS	25	31435	12619	29783	25647	31753	17364	78738

PG.Genes	n	mean	sd	median	q1	q3	min	max
RASSF2	25	48307	13720	47133	39527	60924	18207	71832
LRBA	16	2949	1713	2579	1383	4436	938	5884
LRBA	25	230054	90665	189586	175022	270293	119885	442962
BCAM	25	76282	77552	46586	40165	86287	18952	406173
PPT1	25	9427	3569	8994	6809	10958	2849	18977
RPL14	25	27223	15513	21816	15067	36164	8822	71101
ANXA11	25	22506	15322	21442	11678	27034	3091	71219
PAPOLA	25	8453	3370	7792	6428	9735	3594	17561
FXR2	25	7381	3188	6690	5703	8340	3819	20922
RAB5C	25	157214	45652	170639	132686	185814	53822	249295
RAB7A	25	56484	29164	50973	43099	59617	17317	149152
RAB13	25	274100	84788	258178	193745	307143	150332	485535
PDE6C	25	25142	4278	24979	23390	27306	14614	34303
SCNN1G	25	23242	7491	21747	19264	24077	13045	49805
SCNN1D	25	17651	5243	16844	14150	18443	11012	31068
DAP	24	6399	4412	4712	3656	7928	1758	18838
DUSP3	24	12991	7476	10717	8762	17635	3145	35347
SMARCA2	25	6266	1860	5759	4839	6958	4020	10819
TPMT	25	25665	9339	24625	19458	32444	8104	43260
RENBP	25	20844	14539	18686	15830	23136	1799	82651
MECP2	25	21446	15922	15993	10782	24291	6899	69550
IRAK1	25	373135	346494	272285	187258	349449	125552	1757423
ALDH5A1	25	4672	1224	4348	3731	5628	2948	7234
HSD17B4	23	11208	13860	9058	3600	11413	942	67382
PSMD7	25	146817	48974	140471	128737	182450	57851	248244
SUOX	25	7319	3814	6476	4978	7462	3758	21943

PG.Genes	n	mean	sd	median	q1	q3	min	max
SGSH	25	18425	7276	16294	13733	22753	6421	35864
STAT5B	25	13652	5740	12801	9001	14697	6726	30340
USP11	25	97633	51674	94980	57398	120242	31025	263925
KCNQ1	25	12800	3925	13006	9737	16055	6107	20204
PLXNA3	25	28488	15863	22631	20391	29182	14760	82392
DYNLT3	23	2756	1360	2524	1697	3218	830	5942
RPS6KA3	25	33926	10452	32914	25562	37907	16921	59067
GUCY2F	25	18876	3091	18984	16745	20660	13683	25011
HDGF	25	87523	71243	86227	45849	107618	16394	386361
LUM	25	181374	118260	143695	94387	221364	57779	457551
PRELP	25	34070	25965	24220	21071	38529	11098	118353
CNN1	24	23679	25775	13721	11951	19556	6087	109506
CCNH	25	11048	4491	10876	8378	12465	5065	29037
NDUFA8	25	9074	10354	5106	3323	9988	1786	47774
HNRNPA3	25	35820	25906	24121	13452	57727	2240	87967
HNRNPA3	25	80283	33329	85462	55967	107084	13697	124824
HNRNPM	25	29482	13280	26570	20759	36530	15042	70843
KPNA1	25	17266	5766	16772	13738	20351	9579	30109
DGKE	25	14567	5726	14895	11533	17766	4362	29923
POLR2H	25	12608	7977	10486	8864	13778	5242	44795
MAP2K6	25	93982	38798	77097	70303	106219	41333	190431
ARHGDIA	25	129468	43196	131225	102490	149395	36822	216725
ARHGDIB	25	84012	46322	73372	66507	93374	37950	268624
AGFG1	25	188248	57869	188267	141259	219178	84274	290788
HNRNPF	25	18482	13737	14389	10040	21963	3125	65103
GTF2A1	23	6034	2893	5078	4482	6761	2959	14739

PG.Genes	n	mean	sd	median	q1	q3	min	max
GTF2A2	25	18651	11861	14414	12273	20365	7447	63772
ZNF140	24	28884	32118	15950	11473	37003	6146	158389
ZNF131	25	17635	6265	17267	13967	18984	9056	34229
ZNF142	25	141379	35939	134133	116772	145418	98616	271426
RBM5	25	9843	7639	8048	6093	9332	3804	37679
RIDA	25	8799	6255	7769	4106	10583	1756	25691
SMS	25	27226	15791	23938	17930	32260	6520	69491
HK3	25	48200	21579	39709	34363	48859	30319	111472
MRPL12	25	6226	6094	4915	2473	5909	816	23444
STC1	25	218624	111628	207204	159002	250688	56744	560417
NDST1	25	7739	1960	7675	6612	8929	3623	12726
THOP1	25	11286	5206	10191	6460	14801	4253	23527
AKR1C2	25	9031	3018	8674	7417	10578	3359	17432
CAPZA1	25	57499	26332	53055	39715	71640	17652	122441
HMGA2	18	3518	2165	3127	2427	4293	1065	10721
CRIP2	22	22351	24932	15886	13228	20482	4345	126618
PDX1	17	2536	1727	1962	1587	2499	1084	7837
BLVRA	25	49374	11242	50825	42221	59689	21337	63081
PPP5C	25	22232	5380	21898	18223	26307	13553	33417
ARFIP1	24	12559	8290	10572	7241	15580	1915	33764
NUBP1	25	16118	10730	14295	12206	16696	8615	64986
ACLY	25	33106	19349	28669	21340	38616	9055	107695
METAP1	25	18666	7142	16538	13263	21732	10058	40702
SUCLG1	25	10298	4286	10450	6356	13688	4058	18185
MVD	25	209859	52895	199281	170549	244064	141465	354709
COPB1	25	227657	124770	227235	133540	328267	58011	471699

PG.Genes	n	mean	sd	median	q1	q3	min	max
COPA	25	21554	10860	19723	11962	29513	6759	43064
CTSC	25	12818	9440	12398	7213	14306	1906	48418
LIMK2	25	31929	5945	30901	28460	34817	23283	47943
AP3M2	25	18447	4575	17812	16047	20393	8861	29180
AP2S1	25	62187	24811	60977	43530	64279	22459	118660
TTC3	25	46983	8066	44636	42081	49827	30187	64025
SMTN	25	49370	10657	49595	42283	56076	27725	70061
SLC16A1	25	11007	6910	9709	8147	12577	2519	34486
SEC24C	25	48265	27759	41766	29740	57938	11993	119541
SUB1	25	70075	43528	67435	36062	95945	8425	205917
CLNS1A	25	110835	61257	99591	77505	124175	17175	321928
CRISP3	25	23089	27030	15503	9808	23876	1499	139205
BLM	25	190779	41439	203349	153016	216584	114126	269208
RARS1	25	10064	5867	9629	4980	15703	2009	20942
ATXN1	25	14985	5869	13363	12015	16291	7902	35072
YARS1	25	24957	12249	25908	15729	33788	7878	56590
HSPA2	25	352933	138245	343335	263473	420988	132667	803267
RAD23A	25	24079	6646	24882	17703	27996	14119	36119
RAD23B	25	33896	13970	31579	24894	39322	13942	71628
EPHB3	25	8682	3853	7389	6097	10248	4121	19854
EPHB1	25	24895	5024	24071	21218	28696	17420	37431
AK2	25	62018	51229	49998	32520	59836	21027	216408
GAS1	25	20597	8570	18237	14849	26678	7077	41068
ALDH18A1	25	9876	4447	9199	7121	11219	3787	22226
NAPA	25	11968	3452	10956	10064	14856	5588	18286
ADPRH	25	23528	11508	21051	17445	25730	9855	66178

PG.Genes	n	mean	sd	median	q1	q3	min	max
AIF1	25	17447	5515	16469	13572	21485	9498	28235
EIF5	25	19296	7715	18825	14093	26711	2329	32400
PSMD4	25	14493	6662	14037	9386	17165	4648	31055
DRG2	25	13924	5286	13512	11313	16560	4259	26995
PLTP	25	80438	114537	47026	32043	86628	24683	607529
CSE1L	25	14861	12674	13854	7939	16886	2072	69507
VCP	25	132816	87658	115942	76811	161400	38310	432102
MFAP1	25	19626	8564	17911	14091	24718	10000	50168
HADHB	25	21422	16495	15294	9533	30353	6066	74004
MANF	25	191263	146357	142858	94850	237618	44939	630301
AFDN	25	79913	45272	66433	47095	92405	39894	220985
CASP7	25	26023	8126	26396	21261	30083	10822	49226
CASP6	25	175971	133137	111953	87065	256631	18308	557986
ADK	25	32928	13064	34167	22022	36984	15548	68963
LAMB2	25	66245	27512	60623	46295	74371	37868	146172
CDH13	25	23346	12057	17974	14670	31458	8826	52074
FOXG1	25	38240	14361	37619	27062	47651	14121	80019
SNU13	25	58263	22432	55419	46553	75262	15161	102479
NPEPPS	25	72983	25864	76664	56466	85404	24799	118427
HNRNPH2	25	11948	6183	10287	6522	17674	3583	25410
XG	25	5076	6006	3167	2639	4973	1026	32126
OXCT1	25	24330	21002	18022	14078	25189	9644	109592
EIF3B	25	17741	7477	18775	12914	22850	5191	36415
NDUFV3	25	9051	3394	8715	6592	9691	4647	19585
MARS1	25	33002	13966	30533	21592	40160	13666	58981
ITGA1	25	31566	15490	27748	19750	40203	9813	64325

PG.Genes	n	mean	sd	median	q1	q3	min	max
ARPP19	25	13209	7021	11624	8873	15605	3611	36023
CMC4	21	2207	1492	2013	1209	2726	617	7171
P2RX3	16	2060	2823	1066	574	1983	217	10774
EIF6	25	43871	11926	45759	35065	50595	22139	67858
CAV3	19	21426	16530	21179	6593	30331	2114	58323
CTBP2	25	116041	27446	112849	104935	136783	63767	169300
RP1	25	53610	24756	50709	41498	53606	37884	163693
SLC37A1	25	14196	20623	9698	7128	12413	5118	111024
CFAP298	23	4439	3327	2997	2552	5136	1910	15904
GEMIN4	25	15773	4041	15676	13963	17746	7633	28766
EVC	25	149150	28362	148221	126860	160625	98938	207021
GSDMD	25	9193	3657	8259	6689	10257	3357	18263
EPPK1	25	76979	10827	76195	67153	84203	59187	98768
MTPN	25	49001	16049	46947	40207	56355	14696	101155
NLRP6	25	66942	27192	57958	48566	75564	41214	147750
TAS2R39	25	8214	7342	6133	4605	8361	1699	38167
DEFA1	25	494494	707099	301816	77921	640914	31369	3393968
GNG2	23	15283	12661	11347	7219	20086	2980	53855
LILRA4	25	6879	4543	5710	3893	7590	2633	19762
ZNF445	25	22964	11285	20986	18716	24035	10089	72648
ARPC4	25	170153	67536	159308	133551	198763	69109	362127
ARPC4	25	11684	4464	11265	8962	13380	4447	25497
CD81	22	7632	5176	5537	4163	9890	1856	19072
TPI1	25	968658	346576	984399	754182	1154086	270800	1658289
EIF3E	25	21188	10632	22242	13516	28987	4240	46656
PTEN	23	27238	29155	18220	10129	28872	3350	113201

PG.Genes	n	mean	sd	median	q1	q3	min	max
PPP4C	25	107177	78098	89739	76475	114131	42616	446585
GABARAPL2	25	15584	4313	15925	12714	18246	8963	24974
MYL6	25	21166	10366	20707	13005	27297	6376	48979
PFN3	25	15058	7163	13581	10721	16582	2475	29625
ACTB	25	1461629	507408	1473256	1226052	1693862	458674	2482836
EIF4A1	25	197508	108838	171522	124609	247924	84490	573565
RPS20	25	24989	17138	22451	11664	34935	2386	60197
PRPS1	25	25304	8567	23207	19263	28203	13298	48365
PSMA6	25	64721	27241	62230	48726	82582	22418	131031
S100A10	25	67844	32572	56883	42294	81229	29016	145276
CDC42	25	96325	29670	88518	79381	115630	34576	162742
DSTN	25	131891	63280	124624	93020	154940	36130	291724
GMFB	25	13094	5303	13445	9125	16418	3977	23750
RAB5B	25	11775	4227	12413	8446	14889	3717	20651
RAB10	25	21694	7817	21444	15846	26611	6831	37312
UBE2M	25	79924	31631	79592	64685	89858	20995	187235
UBE2K	25	32168	15167	29915	23502	42257	8462	59162
UBE2N	25	161385	47483	169963	140281	194886	53462	234445
RAB14	25	16035	5226	16247	12125	18999	5609	25037
ACTR3	25	108810	47655	99355	80160	123191	44542	257723
ACTR2	25	48106	21153	42930	32163	60663	18611	101504
ACTR1A	25	26923	9488	26661	19034	32517	11136	46472
POLR2F	15	2110	1242	1567	1163	2828	525	4840
ABCE1	25	7465	3353	6902	4857	9609	2756	16489
RPS3A	25	29298	13968	25340	19984	38645	5466	65420
PSME3	25	16845	8080	16862	11082	22132	5070	36615

PG.Genes	n	mean	sd	median	q1	q3	min	max
RPL15	25	11092	8998	8524	5155	13983	1189	39083
MAGOH	24	8497	4642	7375	5542	9998	2417	23545
RPL27	25	59537	34544	48842	37181	78660	10825	158683
ATP6V0D1	24	8919	3570	7943	6664	10467	4508	19578
PCBD1	25	45758	19897	50133	28626	59555	12087	75589
RPL37A	25	221222	224064	182441	84147	235035	15956	1023253
RHOA	25	14510	4771	13969	11547	18595	5023	22477
NCALD	24	5847	2996	4714	3747	7394	2765	13848
HSPE1	25	337595	260433	265301	152501	498774	90598	1258143
LYZ	25	43106	41244	29891	12578	52711	6056	148172
ST8SIA6	25	54445	18623	51159	43815	65645	19223	91432
VBP1	25	15044	7135	14745	10131	18728	4497	36555
B2M	25	34272	37757	18913	15514	29157	6766	164692
NPC2	25	74169	52834	62118	52762	84135	17642	284292
COPZ1	25	41152	28021	33399	23252	50981	5554	147332
UFM1	25	91811	43323	93333	58025	107638	19404	190679
DCAF7	24	8866	2749	9125	7155	9957	2867	15288
WDR5	24	16774	5388	18154	12544	20546	5836	25285
AP1S1	22	4619	2802	4309	2468	6651	927	11155
NUTF2	25	41783	12785	40270	35806	47468	17997	71416
HNRNPK	25	161214	77439	145377	118249	195627	35942	386339
YWHAG	25	58856	22172	58910	45165	72484	12394	109837
TIMM10	24	13727	16172	7739	5819	12675	2866	75006
RPS7	25	31323	11285	32369	23766	38661	6907	55109
PPP1CB	25	25517	6931	25588	21711	29132	7446	38779
PSMC1	25	12905	6356	12548	7193	18191	2895	22328

PG.Genes	n	mean	sd	median	q1	q3	min	max
PSMC5	25	15160	6334	16008	9017	19496	5374	25024
RPS8	25	16821	10199	13802	8187	23569	5041	47612
RPS15A	25	23626	16677	20402	12379	31284	6147	79936
RPS16	25	46927	29178	42514	26759	62403	18437	153747
UBE2G1	24	9635	4118	9462	7112	12145	3106	19272
UBE2H	25	8593	2784	8061	7071	9563	4549	18343
YWHAE	25	51658	34189	54156	19375	72774	5542	137834
RPS14	25	16000	6338	14445	12601	19011	6681	32001
RPS23	25	13338	8996	10910	8094	16079	2654	49503
RPS18	25	25485	19353	21298	11114	34264	3627	95483
RPS13	25	24935	21816	19909	9481	32050	4121	106709
RPS11	25	8985	6028	8397	5091	10840	1749	30486
SNRPE	25	30488	18274	28043	14098	42407	2949	76505
SNRPF	25	20132	16520	15300	9973	22176	3751	69581
LSM3	25	70954	32113	68966	51093	84748	19410	157145
LSM6	25	33403	15134	33261	24263	40173	9507	74586
SNRPD1	25	31742	22512	28040	18309	32766	5646	101110
SNRPD2	21	21966	16478	18052	8346	28001	867	56066
TMSB4X	25	270130	220235	281621	26494	388375	5871	739412
ARF6	25	20011	13118	17880	12210	26192	3675	64669
PSMC6	25	107159	48474	98472	67028	139834	37849	209339
RPL7A	25	13976	10210	12159	5802	18245	3024	49388
POLR2G	24	4766	2688	4427	2967	5426	1384	13448
ETF1	25	17273	8716	17446	13107	19677	3356	46490
RPS4X	25	29946	16951	28593	16979	39356	5341	80835
PPP2CB	24	7264	2685	7003	5514	9397	2354	13232

PG.Genes	n	mean	sd	median	q1	q3	min	max
ACTA2	25	180633	95076	153071	113595	234034	60160	395185
RPL23A	25	112378	142500	90495	70968	100434	31308	777015
RPS6	25	22727	13738	18415	11847	31396	6034	53850
H4C1	25	85198	74517	54622	37899	85264	18118	339353
RAB1A	25	39566	13011	41531	32346	48077	10885	69719
RAN	25	237402	125736	208375	176486	292901	20729	693419
RPL23	25	12362	8496	10767	6265	15525	3091	42076
RAP1A	25	46258	25879	46487	27339	52925	19345	145196
UBE2D2	25	42339	17283	38160	31797	51157	19308	84167
RPS15	25	10090	6358	8661	4602	14316	2128	26546
RPS24	25	8971	2435	9123	7288	9962	4803	14959
RPS25	25	38606	31468	26382	16661	54079	4106	141474
RPS28	25	41133	35431	38531	9462	67234	2613	121205
GNB1	25	30163	9819	27716	21792	39443	15404	52454
POLR2L	20	3250	1457	2902	2452	3668	1076	6387
RBX1	25	17432	6354	15379	14066	19797	10560	41966
GNB2	25	20410	6799	19726	15577	22364	11609	43789
RPL30	25	24582	19414	19375	13476	29698	2954	83163
RPL10A	25	11898	7494	8352	7242	14567	3188	34330
RPL32	16	3713	2638	3635	2123	4428	806	11893
RPL8	25	9774	7539	6523	4654	13461	1372	36829
PPIA	25	1055294	281063	1019088	952688	1288628	335002	1461720
PPIA	25	14100	4392	13519	11855	16407	6228	29148
FKBP1A	25	97350	32948	95023	80859	106713	29093	181245
GRB2	25	23039	11923	21205	15261	26652	7285	55174
AP2B1	25	22503	8864	21739	17365	28260	8512	38901

PG.Genes	n	mean	sd	median	q1	q3	min	max
PPP3R1	25	7394	4143	6897	4264	9014	1656	18651
YWHAZ	25	492145	191262	473857	428125	561717	74691	906568
UBE2B	23	5163	2030	5368	3561	6384	1551	9410
SUMO1	25	26049	18742	21011	12538	36031	5027	88544
DYNLL1	25	173507	83845	151366	123375	219968	63418	387064
DYNLT1	24	12579	37290	4865	3276	6749	1179	187313
RPL38	25	27509	22201	20920	12895	34487	2182	110502
SKP1	25	31824	12635	34909	23423	36593	8655	73484
GNG3	25	17516	12770	12987	10621	15387	4812	52275
RPS21	25	48998	24298	41941	24573	66003	15146	88685
RACK1	25	75127	41115	75293	44621	105877	9773	142963
ACTG1	18	15815	20127	6809	3869	18563	989	72452
UBE2I	25	84647	26720	91213	63901	100764	27373	136892
SELENOW	24	7961	6209	6108	4750	8658	3329	33897
TMSB10	25	107665	55186	111701	54841	141750	29495	210186
PPP2CA	25	39377	10487	40565	31572	46009	15633	59881
YBX1	25	76365	45967	65343	46827	92603	17478	208324
CSNK2B	25	11957	5095	12534	8294	14125	2295	23216
TPM4	25	55869	35498	48169	25687	73311	9782	139790
TPM4	19	16091	19171	9144	3173	19839	837	70203
EEF1A1	25	4139252	2155433	3748954	2564255	5398989	1484990	9373522
FKBP1B	25	4334	2056	4192	3213	5705	843	8410
ACTA1	25	60260	20238	56764	45549	75411	28677	100525
TUBB4B	25	144819	77879	141191	95563	180754	41404	419642
CSNK2A1	25	12570	4926	11959	8771	13884	5705	27655
PAFAH1B2	25	99637	71258	83218	68232	100797	17333	331180

PG.Genes	n	mean	sd	median	q1	q3	min	max
PSPH	25	25564	12085	21612	19030	26742	10217	56028
RBM6	25	76687	16018	73638	66790	80948	54948	118163
RPP38	25	83666	36103	78634	60828	98066	35783	215164
PIP4K2B	25	5772	12193	3635	2068	4460	723	63866
CCT2	25	71781	30860	71800	56772	91454	23812	138233
RAE1	24	7833	4663	6559	4394	8732	3049	22008
DENND2B	25	49929	12952	46765	43415	54053	35773	94859
PRKDC	25	128935	50593	132693	82303	167853	45805	244507
ADAM17	25	18399	10993	16471	13688	18530	10124	66287
BLOC1S1	25	20003	8216	18546	16935	21521	8208	52987
GPLD1	25	34524	24059	29986	21014	33678	15118	121916
LCN2	25	65139	63186	52951	21857	83081	3176	245736
NUCB2	25	31162	36319	19453	15857	30945	10455	190454
S100A12	25	23338	18575	16017	10405	34138	3944	72942
BASP1	25	61360	34232	53634	36186	85824	14539	135971
MRPS36	25	49195	19440	48429	34848	56302	28772	112607
MRPS15	25	16638	20374	10665	7821	14951	3563	102882
MRPS21	24	48393	26082	43249	27147	64627	11547	94059
MRPS34	25	56556	23811	49109	39148	65055	25991	124152
HMGN5	25	45457	29576	40006	23525	53194	13419	134569
SARNP	25	17507	7570	16866	12870	21749	3007	35362
RBP5	25	9560	3734	9124	6506	12824	4839	16763
LACTB	25	18981	6207	17599	14939	21039	11303	35696
COG7	25	189777	67272	193572	143832	212642	98841	385403
RPL24	25	106699	65451	85656	68512	120595	15021	315469
TXNL4A	25	16984	10106	13686	11049	17372	7815	53414

PG.Genes	n	mean	sd	median	q1	q3	min	max
CBX1	25	11750	6126	10741	8565	13035	5541	35455
SMAD3	20	16256	15879	9679	5803	23015	1134	55753
ARF5	25	15048	7340	15823	10849	17941	5284	39929
ERH	25	61371	41710	54133	36602	72325	10352	200471
RHOG	25	17171	4286	16704	14449	19451	9979	26179
RPL19	23	10659	7653	8357	5239	15740	752	32814
SRSF3	25	45642	22187	39808	36878	51829	12524	118482
MXRA7	23	2856	1192	2711	1882	3586	981	5497
FOXK1	25	10918	3660	10925	8270	12830	6066	20863
CCZ1B;CCZ1	25	24303	8510	24422	17289	28992	10489	45218
TMPRSS15	25	47773	13430	46139	42236	51782	25037	85078
MUC5AC	25	14065	3866	13083	12144	15362	8061	25139
FBLN2	25	289156	161040	243839	195553	336185	109723	803076
VLDLR	25	53048	23213	46461	44038	57149	23250	143670
HSPG2	25	226119	138706	186566	126476	260689	92798	550580
RBM3	24	8340	3452	7837	5417	10599	3567	17047
CYCS	25	72271	56051	58240	37474	88410	11738	253189
TFAM	25	18291	9807	16098	10915	22936	8547	44792
PITPNA	25	16405	6509	15163	12557	20319	5596	35405
HDLBP	25	15337	10631	11721	8312	22286	2313	45131
HDLBP	25	121032	56396	101297	79775	148011	56741	262202
GTF2B	25	561176	389896	455792	389680	539942	229921	2244656
CDK6	25	12667	3442	12091	10324	14574	7236	20823
PURA	25	14852	14638	10537	6552	15547	3908	70945
CLTC	25	79247	42984	78231	46446	107887	10072	168202
HSF1	25	48873	53135	34152	23825	51456	15096	283448

PG.Genes	n	mean	sd	median	q1	q3	min	max
FKBP3	25	66242	32544	64440	41762	78908	20321	156830
REEP5	25	7257	5543	5987	4575	7929	1761	30578
SORD	25	99572	123078	57675	36524	92153	12040	575460
HNRNPU	25	96848	39855	93978	68710	131205	29994	186298
SPTBN1	25	20849	11794	17756	14588	22332	5950	60610
TIAL1	15	3243	2566	2365	1578	3571	917	10799
INSM1	25	8850	2746	8906	6265	10685	3972	14364
SET	25	68995	36989	64067	39626	97109	7873	139820
SRSF2	25	38309	20239	35077	29185	42572	12852	117293
CTBS	25	7550	3030	7042	6193	8411	2820	16972
FABP5	25	170576	115176	142729	96087	195406	43128	587243
ANK2	25	178581	65230	159354	132043	196935	96833	389996
CAP1	25	105891	41812	105847	79765	125077	36336	207977
PFKP	25	19994	12722	17571	10621	23856	4088	48297
EWSR1	25	26819	9331	24899	21111	30051	16942	54579
OCRL	25	17877	9865	15001	12679	17571	10296	59434
TAGLN	25	591864	798960	175662	72072	819205	14745	2783086
OGDH	25	93484	28078	88737	69230	101170	62620	168185
COX6A2	25	9109	3622	8008	7251	10758	1910	15096
ALDH6A1	25	27225	23474	18089	14553	26942	7048	112221
BDH1	24	7492	3388	6762	5181	10207	1325	14832
DSG1	25	89235	48296	86185	49981	103052	38247	244960
DSC2	25	17570	16522	12564	8874	17284	1584	67307
H1-1	23	9666	7770	6650	4621	13096	1013	32963
RPL18A	25	19403	13714	13294	11058	25702	5743	57647
GHRHR	25	14370	13465	10000	7528	18692	2196	66371

PG.Genes	n	mean	sd	median	q1	q3	min	max
GCNT1	25	26811	9754	26415	21433	29490	11345	60348
MAP2K1	25	13240	10444	11770	8636	13552	4924	58951
FKBP4	25	59679	51844	48778	27682	65853	16087	279898
NUCB1	25	19793	25331	11576	9346	19035	3966	133396
RPL6	25	15736	11217	14525	8573	20254	2694	52150
TOP2B	25	12219	4732	11274	9717	13395	7705	32657
CREB5	25	42438	21203	39567	29903	49654	13739	111434
AKAP12	25	19582	8683	17860	13251	24554	7533	39868
DST	25	235837	57661	209633	200751	282607	156455	392788
CAV1	25	16541	8996	15407	10393	23246	3675	38798
TGFBR3	25	11262	2030	10868	10308	11931	7744	16386
TNFAIP2	25	7338	2194	7335	5795	8708	4166	12137
LMNB2	25	27770	27093	17084	15685	25919	6971	129491
PTS	24	2636	2188	2071	1275	2854	615	9765
CFHR1	25	7551	6063	5219	3708	9673	2054	25645
EVX2	24	12031	7225	10899	6736	15060	2641	29428
GBE1	25	35612	21295	26686	19553	48409	10151	89579
NOTCH2	25	185594	72232	165148	151696	187036	113695	479779
HGFAC	24	2434	1350	2133	1594	2805	938	7344
SSBP1	24	15792	13604	10009	7445	22395	5489	68586
YWHAH	25	81022	32234	78284	57579	100710	15607	147854
PLP2	24	4620	2984	4059	2954	5719	450	14114
CSTF1	25	24328	12125	21963	19135	26345	9164	77364
PTPN12	25	69182	9895	69345	65164	74600	49610	91239
CLC	25	11023	4382	11505	8173	12469	3165	23476
SRSF11	25	58567	41138	44744	40741	62403	25462	229109

PG.Genes	n	mean	sd	median	q1	q3	min	max
EEF1A2	24	30918	15951	28184	16771	42068	9054	74507
CALD1	22	5476	4873	3952	2841	5879	884	18698
CALD1	25	51817	36466	40664	26727	72067	11599	166451
EML5	25	129413	18848	124378	113128	141903	105439	172573
PTPN11	25	18685	7834	17850	11642	24281	8205	36506
REG3A	21	16904	32301	6623	4295	9666	781	137087
PPP2R3A	25	107348	26174	100498	89026	132237	70378	160630
PPAT	25	13032	8339	10426	6256	17499	3456	41232
EXOSC9	25	8599	3078	9133	6383	9818	3966	17732
EXOSC9	25	14024	9533	11644	10122	13383	5763	47945
PSME1	25	320961	137565	305437	232144	423384	107316	557694
GABPA	24	3650	1306	3426	2943	4049	1826	6910
RING1	25	3844	4197	2729	2142	3496	642	19025
FMOD	25	16687	17294	8564	5609	21973	2426	69718
PRDX1	25	708862	363608	656432	535023	746942	287674	2024737
RPL18	25	15164	12038	9887	5080	24463	2203	46896
C1QBP	25	44468	37548	35782	18640	54829	3713	184571
CKAP4	25	34821	8320	34527	30237	39867	15570	48435
TJP1	25	12452	4860	11163	9415	12594	7203	28781
TCHH	25	35361	13443	34464	27773	44368	12969	65303
TFF3	24	96462	117696	36726	12920	189648	1985	473080
KHDRBS1	25	58467	30227	52175	36272	73179	9804	129442
SOS1	25	28565	10504	27006	23676	30094	19028	74823
LRP1	25	106053	27678	101927	83809	126350	65607	175818
SRSF1	25	15226	10997	12770	10341	15649	3353	57351
ARHGAP1	25	32090	13928	27866	22423	39738	12082	64542

PG.Genes	n	mean	sd	median	q1	q3	min	max
SRSF4	25	15085	13309	12277	9386	14955	6505	71085
TGM3	25	9079	4434	8570	7467	9634	4431	28639
DHX9	25	53703	19030	53281	41830	67871	16386	87763
LGALS3BP	25	83721	203141	27914	15864	78707	6081	1037211
EHHADH	25	27462	7572	26225	22373	30943	15680	47703
PPID	25	31400	100789	13276	6917	14988	4052	514625
SSRP1	25	17186	14113	13605	10470	17636	9120	80389
SLFN5	25	91640	30318	83025	78431	95440	58682	220456
VAC14	25	40530	9528	39482	34735	42602	27690	72159
ZNF616	25	144814	53358	129603	113054	157191	90511	328507
RBBP4	25	26266	11804	25554	18646	32762	6159	46634
NCBP1	25	111424	43973	104611	84933	129382	66806	284312
AHNAK	25	102151	40405	98279	65581	131630	37420	170816
EGFEM1P	25	159673	58574	153228	115830	193082	54211	273376
ABHD18	25	44212	25897	39709	26448	58335	18058	138622
LRRC74A	25	49973	28306	41751	31805	63491	20488	132550
HSPA14	25	97945	36399	93053	68052	116986	56621	179394
SCRN3	25	16634	18199	12774	10536	15829	6796	102096
CGNL1	25	103656	21597	103255	90440	117758	68224	166407
CCDC173	25	40194	20067	33080	30891	44525	24295	128492
FABP9	25	10693	5345	9022	7798	11156	5311	28917
NEXN	25	29870	8160	30195	23666	32573	17570	55932
GALNT2	25	5210	4024	3988	2832	5459	1666	19370
GALNT1	25	7115	2480	7157	5176	8623	3625	11940
AP1B1	25	99617	34892	103111	81600	119635	34514	178276
CPSF1	25	80198	16355	80156	68048	93439	54571	111150

PG.Genes	n	mean	sd	median	q1	q3	min	max
BST1	15	3947	3608	2462	1712	4736	752	14822
BST2	21	4194	3973	2995	1278	5050	565	15976
HMGXB3	25	57831	9634	59047	53578	60757	39926	75625
WASHC5	25	16240	4573	16158	13034	18137	8573	27363
NUP160	25	47884	15323	44113	38279	56298	25497	95095
SCAP	25	14005	7919	11635	9520	16796	4572	43774
ARHGEF5	25	16330	4888	14032	12917	20738	10218	28711
GTF3C1	19	11412	5045	11074	8440	13077	4670	27182
HYAL1	24	16204	9871	14632	9907	22994	1215	37242
FSTL1	25	9622	14092	4626	3648	9902	2240	72012
SF3A3	25	17399	7718	16105	12554	19452	7289	41896
DPYD	25	6039	2133	5293	4594	7381	2832	11053
ILF2	25	45734	23708	41229	31528	59216	7940	108333
ILF3	25	21345	10918	21246	15251	26477	3553	51340
LMAN2	25	50821	44821	37478	30233	53062	24084	250688
IRAG2	25	20941	8092	19483	15999	23059	10834	52845
EPS8	25	13700	3585	13339	11610	14746	8742	24783
FOXF1	25	18389	14551	11771	9869	23183	2813	67083
FOXD4	23	52973	41963	37371	27996	58376	19732	193460
ANK3	25	76376	15353	72037	66726	86506	54901	132405
ANK3	22	34591	31525	25709	10335	53432	1113	122471
TAF10	25	411575	146885	375070	299717	475340	187308	816438
MYO1E	25	46563	13571	45271	39205	52308	20843	80330
PPP1R8	25	4632	1743	4255	3782	4894	2220	10382
PTP4A2	24	3855	1411	3835	3044	4743	1422	7573
ABR	25	54075	11630	52369	46446	57531	33705	80840

PG.Genes	n	mean	sd	median	q1	q3	min	max
CSTF3	25	74293	16011	73008	60687	80745	50662	113437
ECH1	25	27578	26730	19093	12537	31125	5508	135266
STRN3	25	4344	1273	3966	3452	5026	2509	7455
FLII	25	75936	27483	75565	56769	86406	35280	145625
LCP2	25	18280	12193	13156	10867	23097	6766	61355
USP4	25	23932	4874	23996	19903	26475	14869	33488
CHAF1B	25	38355	17484	37875	26805	53846	6372	70910
DUSP4	24	27584	26712	18387	13253	27205	2369	102135
KLF10	25	258701	119711	239873	184601	281040	127751	683933
IK	25	624481	397960	478067	430668	579228	326211	1853941
EIF2B5	25	77980	28077	80956	64811	96123	28553	148996
TARDBP	25	13574	5595	13545	8999	16448	5138	26780
HNRNPA0	25	27352	20457	22146	12580	32999	7992	89129
PAK1	25	16727	7429	15895	11176	22351	2748	29092
AIMP2	23	5212	3699	5092	2153	8057	435	14088
FADD	25	30095	20332	21617	18428	35679	9230	98324
PRDX4	25	30435	41245	22501	11396	30266	6472	215306
PAK2	25	32962	11248	34827	25024	39890	10310	50988
CBX3	25	24073	14615	22694	14846	26673	6058	70051
STK3	25	18738	8584	17481	14451	19816	9427	55041
PSMD2	25	6572	2793	6413	4229	9426	1694	11241
MMRN1	25	45437	9573	43083	38756	53447	28885	64518
DDX10	25	33770	14323	29780	25860	37610	17349	76876
DNAJC3	25	20995	9089	18840	15672	23685	9055	50918
NME3	25	10689	6945	8212	5922	14669	2722	29074
SRSF9	25	10379	4906	9193	6597	12796	3558	24103

PG.Genes	n	mean	sd	median	q1	q3	min	max
SRSF6	25	24369	8400	25246	18379	28892	10197	49751
TRIM28	25	47881	57138	36282	19464	56452	7271	304793
G3BP1	25	22994	15310	20742	11686	30694	3505	58689
NMI	25	12272	8007	11441	7401	15128	3125	40566
SLAMF1	25	9767	2792	9027	8225	9919	6004	17596
EIF3I	25	14532	6645	14512	8832	18578	3512	29070
ILK	25	17166	15318	10632	6316	24409	3855	56552
MSLN	25	113098	32043	101627	97443	124296	74368	204334
NNT	25	18856	4373	17217	16302	20698	11483	29211
SNTB2	25	17624	7600	15808	13177	19128	8759	42059
PPIG	25	68111	60052	54971	38854	65424	3566	316102
TCOF1	25	101521	26565	92516	87535	106905	74086	183972
SF3B2	25	27808	13549	25439	20442	29884	13527	79889
GOLGA4	25	245027	36554	235106	223871	265233	176662	322806
PDAP1	25	18168	8592	17288	13443	21616	6181	40581
ADAM9	24	108148	25935	101403	87836	126577	65305	156955
LSAMP	25	157872	97244	151451	100998	183502	54790	531880
FKBP5	25	17591	7910	16321	12084	19692	5890	35793
MYO9B	25	20008	5914	18662	16816	21617	11819	39663
ROCK1	25	37804	5624	36749	34513	39913	30001	56809
IL18R1	25	73533	30678	70069	60513	77065	41656	200446
SMAD4	25	4746	3968	3790	2600	5065	600	20402
SNAPC2	25	363983	341427	282899	114922	449689	31719	1265159
SQSTM1	25	7824	2831	6716	6262	8638	3723	15304
TUBB3	18	4231	3715	3284	2171	5852	404	16482
PPP1R1A	25	34830	18605	29727	23793	36413	12506	96112

PG.Genes	n	mean	sd	median	q1	q3	min	max
PRPF4B	25	111686	16686	113662	107673	125262	72583	130048
PIN1	25	13174	4984	13418	8938	15642	5887	23258
EIF4EBP1	25	8228	5256	7383	5506	9409	2700	30253
HDAC1	25	40997	12904	39220	30417	46748	24565	71503
DCTN2	25	24230	11562	20764	18157	32120	7372	55183
ITPK1	23	4984	2482	4726	3651	5962	297	12792
SNW1	25	198268	48214	200961	161829	232708	117396	272074
IQGAP2	25	45747	20299	44394	30946	56053	22827	96452
GPR50	25	608934	276934	520354	446411	704901	264859	1475611
STIM1	25	102055	18359	102349	89753	112307	56709	146675
TRA2A	25	53519	20989	48650	38798	59018	27865	115162
SNX1	25	50372	14360	49473	40297	55167	28161	86866
KRR1	25	16515	7171	14750	13581	16415	9310	40414
CUL1	25	23756	8392	23084	18419	26386	11110	48658
CUL2	25	107338	34954	105959	89240	118422	52036	222253
CUL4A	25	43161	14477	44985	30469	49734	23086	73796
GFUS	25	41524	33131	36001	24202	42631	12555	171041
RAB32	25	31487	19045	25712	19510	37514	11096	78195
FHL3	25	57568	34491	50466	36521	66686	20583	188844
AAMP	25	880687	713006	712301	515959	877451	268233	3807225
ALCAM	25	427849	565807	241111	160226	360417	84234	2682291
LAMB3	25	59056	9139	56968	53764	65368	43863	75250
THOC5	25	11726	3048	11722	9591	13356	5643	19278
APOF	21	5501	3720	3965	2740	7392	487	15819
SPTAN1	24	4200	1608	4136	2847	4923	2049	7840
AUH	25	36345	12224	35631	24716	44935	20335	61129

PG.Genes	n	mean	sd	median	q1	q3	min	max
DDX39B	25	73963	32475	74603	52257	92082	20051	149052
BLMH	25	17390	7870	16722	11816	24310	5705	31707
EXOSC2	24	5237	2117	4607	3650	6703	2332	10011
SNTB1	25	131358	28308	122280	111733	148151	96856	201976
TUBB2A	25	34053	16374	33672	19781	49294	9463	69110
BYSL	25	9051	5632	8286	7118	9650	2155	33370
RAPGEF1	25	89442	36148	78458	64601	99787	47539	171493
CAPS	25	312659	304209	198534	111734	437765	2026	1418100
CBFB	25	8958	3672	9060	6077	10924	3147	16269
IL16	25	57379	32532	45838	34951	67552	17850	130141
CAMK1	24	2531	1509	2030	1707	2775	880	6698
COTL1	25	94230	42320	90754	69316	106731	37796	221979
CYLC2	25	17666	9704	14202	11718	21453	6743	46909
HNRNPD	16	4953	8858	1714	1250	2633	407	30180
SCARB2	20	5185	5676	2444	1985	4883	1130	20574
IL18	25	13932	14383	10045	6582	12631	2191	71744
DPYS	25	37203	10979	34135	29210	47857	20437	60934
DAG1	25	23446	10841	21151	15083	26758	10768	51890
VEZF1	25	10015	2786	9239	8356	11053	5364	17026
DSG2	25	19973	7744	18409	12961	24619	11394	40853
SEPTIN6	25	8898	3722	7641	6310	11101	4151	17489
MORC3	25	78080	18077	75477	67613	85393	51382	135478
SAFB2	25	10534	3260	9816	8305	11948	4571	19080
EIF3A	25	16415	7336	15733	10573	20972	5131	35937
MLEC	25	8523	7476	7136	5703	8260	4475	43437
TTLL12	25	15316	6708	15127	9531	18726	4960	31380

PG.Genes	n	mean	sd	median	q1	q3	min	max
DOC2B	25	715799	384397	635401	582159	769556	373269	2398204
WRN	25	80090	21505	74943	64637	91500	46205	119768
FHL2	25	28593	14342	21097	18216	37732	12570	62906
CRMP1	25	18477	7671	17478	13102	20189	7347	36238
DPYSL3	25	63919	55546	42349	23488	88038	8250	225318
MRPL58	25	14649	11702	10823	6416	17176	2616	44033
DYNC1H1	25	475091	201125	469354	292907	648498	178715	795728
NPAT	25	238148	111938	208728	180099	239154	142582	698824
EIF2B1	25	9444	4413	9668	5465	11560	1887	18596
ADGRE1	25	17200	7232	17207	13524	18757	930	31440
CTTN	25	39840	14800	35732	31065	43240	24748	82833
FLOT2	25	20799	8778	18424	16004	24639	9178	51597
TRIM25	25	48869	21030	44139	31202	53764	25158	108443
PTK2B	25	27382	6960	25909	24405	29842	15614	50257
FGL2	25	26227	20116	24038	20380	24940	14932	120842
FAM50A	25	15267	6298	15433	10177	18884	3799	31026
FRG1	22	2863	1223	2723	2096	3755	1012	5502
GAMT	25	21276	5573	22048	17061	23565	11862	36542
LRRC32	25	14630	10697	10916	7165	20704	2555	41472
GK2	25	7096	2951	6288	5366	7748	3602	16827
PDE3A	25	45751	13071	45928	38933	48672	19364	76733
SLBP	23	4729	3428	3659	2022	6630	931	13023
RBM39	25	302886	103365	287394	247433	329280	122146	552499
WFDC2	24	89240	97638	62647	14567	108983	2747	327445
SPARCL1	25	41195	43667	19573	6844	69794	686	125368
HABP2	25	12036	8426	9870	7972	12315	4605	46544

PG.Genes	n	mean	sd	median	q1	q3	min	max
HNF4G	25	20866	7959	16786	14508	27021	11235	37142
PDIA5	25	16693	16637	11415	7216	20554	4165	87834
PRPSAP1	25	31146	27075	24741	20834	31557	10680	152860
MCM6	25	16941	9275	14524	11007	18958	6263	44947
ITPR2	25	31562	11334	28220	25028	34755	20014	73738
ZNF268	25	73660	20589	67604	62263	88318	43946	120384
IHH	25	106348	35074	100935	86282	126604	47868	179994
ITIH4	25	116436	85528	83907	66369	131586	45384	408174
PLS1	25	54652	16476	55094	43163	62294	26282	86789
LAGE3	24	10313	4421	9802	7345	12273	3204	20687
KIAA0100	25	27782	6963	26902	24805	28968	17586	55013
MDC1	25	16231	3785	15657	13866	17730	10728	25424
KANK1	25	109848	70160	85849	71888	117321	59853	393598
SMC1A	25	35508	8218	34329	29084	36630	24386	56383
RRP1B	25	17030	9081	14908	11468	18420	9224	50435
DIP2A	25	49767	20735	42687	37602	47766	29783	113837
BMS1	25	45822	7701	45386	39230	52507	29935	60007
USP10	25	22791	20103	16573	13811	21304	4504	85062
MESD	25	14181	16139	10628	6308	15723	2651	84933
GANAB	25	38295	34553	30506	19423	48627	10143	187533
RFTN1	25	19979	8416	20371	14127	25455	2684	34420
KCNAB1	25	583868	267248	497328	381856	716963	296753	1345786
LBR	23	11075	5278	10007	7982	11833	6319	27420
MVP	25	23174	19248	18252	9974	29973	2727	90644
LTBP1	25	113759	51278	102239	79373	119550	58927	259542
LTBP2	25	9480	2967	9362	6791	11415	5495	15383

PG.Genes	n	mean	sd	median	q1	q3	min	max
CHD4	25	71154	30397	58050	51293	80632	42255	175787
CRYM	25	14873	3056	14182	13184	16177	10085	21994
KIR2DS1	20	8717	8585	4850	3669	10434	1554	38990
KPNB1	25	47440	22975	41097	29054	65070	14128	93485
PSME4	23	6826	7858	4435	2977	7041	1560	38302
NAA25	25	33402	11740	29562	27402	31831	23940	71816
NCAPH	25	21986	6723	22419	18591	24884	7448	33328
PCLAF	17	4433	3026	3064	1860	6106	1013	10499
WTAP	25	11742	3586	11504	9212	13571	4523	20048
PSMD6	24	9952	4947	10107	6957	14332	2089	18091
MAD2L1BP	25	20189	8587	19002	14233	23550	8837	43713
ABRAXAS2	25	14360	4411	14480	13006	15996	4860	21271
SART3	25	37798	13967	34868	33066	39763	19680	88840
NCAPD2	25	17350	2962	16932	15795	18474	10766	25393
SUZ12	25	23452	6168	22332	20061	25277	13357	40371
ACAP1	25	6204	1816	6484	4804	7325	2909	9861
SNX17	25	25491	8370	23850	19486	27830	9894	47637
KARS1	25	44127	19412	43692	30474	54686	14047	81834
LRRC14	25	82859	45914	69337	44055	109274	32909	219501
ACAP2	25	16438	3815	16248	13838	19091	9637	25971
WDR43	24	31427	10577	30515	24888	37824	7828	50356
POSTN	25	13362	4249	12119	10751	14276	6533	24680
EEA1	25	134513	35114	125702	106181	148175	92632	224772
NCF4	25	3172103	1895784	2465428	1985928	4133400	962838	8678755
PAFAH1B3	25	67624	30430	61357	47080	98442	14474	123698
PLCL1	25	10741	2540	11198	8827	11999	7403	18408

PG.Genes	n	mean	sd	median	q1	q3	min	max
PCOLCE	25	9544	6993	7021	5427	13021	3334	35742
PGM5	25	23308	24963	12569	9903	33801	4036	118194
PMVK	25	233916	249551	194967	111609	245480	44919	1295447
PRKD1	25	48169	31575	38572	34529	49625	21004	183909
PLEC	18	2413	1464	2274	1186	2810	841	6723
PPP2R5A	25	5161	1779	5323	3733	6146	2114	8521
PPA1	25	162793	121733	149819	95479	185229	26570	568484
PDGFRL	24	6448	2806	5070	4562	7668	3531	12851
NONO	25	36539	15704	36992	22585	48734	4787	69958
QPRT	25	7453	4167	6863	5277	9144	1802	19356
RABEP1	25	55169	11839	56046	45234	61991	35266	84470
RAB35	25	6799	2338	6273	5682	8105	3005	12635
RCN1	25	13290	13215	9139	7125	12333	4707	60751
RP1	25	62509	15518	59378	53038	66861	37579	98372
ANKRD1	24	9231	5819	6854	5947	10853	2907	24452
TTF1	25	91539	30377	84870	78437	101698	37478	197725
PCBP1	25	83296	38257	83210	53081	110380	23402	164564
PCBP2	25	167997	75252	176851	117340	216727	39917	347749
ELOC	25	11740	7697	11391	7179	13871	1146	42489
RHEB	24	6899	5367	5272	3380	8105	1626	20009
UBE3C	25	19840	7013	18803	16292	21857	9731	45510
SF3B3	25	230965	136303	200907	150923	263533	88452	740941
PUM3	25	155311	49674	142196	118925	172810	84922	288921
RSU1	25	123403	54624	107186	77815	162288	54250	253247
CNN3	25	73236	43166	66706	35113	87714	11867	160851
SAFB	25	26852	16700	21455	18702	27374	10431	80832

PG.Genes	n	mean	sd	median	q1	q3	min	max
SF3B4	25	13262	7078	10949	9019	14284	6121	35359
SF3A2	25	22514	23044	14473	11957	18558	6198	103355
SEC23A	25	8014	4075	7896	5153	10378	2076	16171
SEC23B	25	22886	13489	21239	11874	29173	3987	56541
SF3A1	25	13380	6764	12384	9241	15635	5721	33867
SKIV2L	25	15142	3214	14326	13037	16586	10712	22997
RGN	25	184445	90456	165994	129849	194170	62612	424799
CDSN	25	16826	14306	9709	8359	16970	5424	63458
TCEA2	25	10606	2924	10185	8971	11550	5768	18669
TGFBI	25	81963	53309	61123	49372	94831	24835	256127
DIXDC1	25	193136	80659	181241	151719	202820	117660	525055
TRADD	24	8058	3572	7492	6629	8947	3161	20410
TSN	25	51848	17838	50342	38244	56223	26953	99136
TRIP10	25	19704	13804	16620	14752	19346	8430	82917
TRIP4	25	11054	6070	9371	7892	11079	2434	28146
TRIP6	25	22061	8983	20554	16979	27783	8410	47688
MAPRE1	25	33263	12381	31089	24461	39174	8377	60652
TSC22D1	25	15087	13838	11662	8011	18092	6170	76826
ELAVL1	25	57171	25483	55993	38423	66968	18961	122387
INPP5J	25	37035	12257	36083	31219	39560	19144	71974
HERC1	25	124056	20533	120585	109310	135864	91629	186459
TOMM34	25	9458	5206	9241	4646	11959	2050	19916
TBCC	25	9424	2606	10032	7918	11061	3577	13597
UBE2V2	25	46248	19855	41209	32622	62664	11211	82598
CST6	25	53968	15618	57405	45281	61435	19862	88926
VAMP3	25	14736	16269	8876	4061	15225	1488	65368

PG.Genes	n	mean	sd	median	q1	q3	min	max
NEDD8	25	40170	15525	40013	27692	48196	4894	73153
ADIRF	23	19434	20241	11667	5331	25609	1137	81327
ADIPOQ	25	197710	140697	166041	114840	214385	23617	546642
ATP6AP1	25	5155	2951	4343	3230	5566	1990	14417
RAB11B	25	48771	19256	51764	35171	56794	20355	111139
ZYX	25	138778	81410	120052	78648	172122	33790	333356
ADRM1	25	33021	11477	31297	25647	40346	11653	59007
CCDC6	25	23707	10874	21546	17366	27308	7878	49751
ENOX2	25	42759	13523	43224	32760	48913	22719	76404
LAMA4	25	10293	2295	10491	8184	11883	6887	15420
SSX1	25	13267	5557	12686	8833	16346	3874	26868
CSRP2	25	5269	3469	4908	2638	6084	1734	16668
DDB1	25	73891	34375	67921	45529	86412	32565	157340
MAPK14	25	5707	2098	5541	4019	6944	2051	10414
CDC37	25	60148	17757	57526	46414	70950	31672	100761
DPYSL2	25	47069	25812	35270	32411	58648	12958	120673
SYPL1	25	5600	1395	5698	4678	6637	3465	8907
RBBP7	25	14871	8883	10821	8310	18397	4551	37277
FXN	25	7498	10127	4666	2372	6372	1823	42921
ZNF239	25	26368	18601	19859	13458	29974	7946	73108
CALCRL	24	59357	142148	27238	8663	35318	1108	703599
SRSF7	25	17540	5075	16235	14132	20636	9429	30323
PRSS8	24	12467	15588	7622	3627	13328	1844	74929
FSCN1	25	92503	57831	64024	57755	112775	25608	278213
IFI16	25	257187	109320	272086	165051	337815	109332	554542
DECR1	25	16481	14550	12262	5501	18942	2994	61320

PG.Genes	n	mean	sd	median	q1	q3	min	max
MAN2A1	25	14070	6044	12515	10937	15038	3921	29228
CLPP	23	13552	5801	11968	9623	16977	5636	26147
TST	25	22275	17001	16958	11028	29934	5056	79880
RTN1	25	21472	6480	20646	15950	24560	12556	37280
UPP1	25	270313	158817	227168	196332	265483	145311	953322
UGP2	24	6839	3987	5956	4074	8220	2141	18153
UGP2	25	65480	24970	61586	48961	77066	27502	128273
TXNRD1	25	12586	6076	11217	8743	16230	4687	26982
IMPG1	24	25588	9170	24368	20624	30776	7990	45348
LONRF1	25	33067	11188	29974	25698	39358	19377	60594
NKPD1	25	18568	9741	15580	12998	20344	10011	54056
HNRNPUL2	25	23107	12740	23059	14243	26926	5727	59716
INF2	25	68608	18925	64453	56265	78309	46442	140271
PDS5A	25	76256	23594	70523	61928	87933	43585	155044
C5orf64	25	21972	11571	18545	15228	22681	11625	56125
CCDC96	25	47568	19478	41115	37659	54640	30860	126373
WASHC4	16	1075	490	1172	635	1495	210	1811
SHROOM1	25	7325	2373	6859	5386	8931	4189	14286
TSR1	25	15607	4790	14642	12153	17424	9250	28350
IAH1	25	13585	5738	13600	9278	15438	4781	30375
SMU1	24	5109	2695	4811	3170	7018	508	10090
SMU1	25	98998	33245	96172	75915	123775	44318	153163
ASTE1	22	84401	110118	44428	17892	118366	10991	495408
HKDC1	16	11657	10856	10408	3779	14217	1585	46404
PRTG	25	101873	24856	103445	82679	117529	59278	153334
DEFB115	22	22700	25664	13599	8108	20797	2318	108919

PG.Genes	n	mean	sd	median	q1	q3	min	max
MYLK3	25	52695	21235	44707	40005	56211	30531	132032
LRRFIP1	25	82285	29487	74803	65523	90650	48904	158947
LRRFIP1	25	11960	4511	11571	9502	13931	5423	26914
P3H1	25	43796	34357	34043	28931	45297	25888	201283
TRMT5	25	13797	5499	12399	10363	14602	8069	30746
C2orf76	25	5790	1864	5259	4859	6457	3359	11755
TKFC	25	34849	13971	31725	24402	46521	15306	59306
OTOGL	25	82448	25909	73630	67222	85474	51559	148233
LGALSL	25	6097	2515	6085	4031	7139	2906	13011
AMZ1	25	23277	8816	19987	16827	30058	8532	43755
SV2C	25	25748	9955	24262	20153	27880	11819	61051
ZFP69	25	45195	12822	45386	37713	56777	21919	68253
MAP9	25	19752	6968	17031	15802	24353	10217	39344
VPS26B	25	10158	3033	10430	8606	12427	4449	14959
HYDIN	25	359548	53081	342514	321931	380879	308249	538056
TBC1D10B	25	86083	50343	67357	57870	84493	46609	268537
PLCH1	25	7996	2457	7309	6658	9012	4489	13521
FILIP1L	25	78196	15833	78194	67424	86404	43684	111749
CCDC58	25	9054	5379	7234	6062	9098	3975	25159
AMOT	25	26655	8426	23390	20711	29506	17858	53682
ATP13A5	25	52185	12949	50133	44971	60434	27763	78738
TIGD2	25	17038	5290	15842	13146	22071	8562	28243
GREB1	25	82154	46316	71304	54956	84572	40651	256249
TMEM259	24	5333	1853	5294	4455	6430	1532	8660
CCDC38	25	12175	5041	11277	8861	13632	6181	24619
ATP6AP1L	24	120580	188927	74194	25989	112087	3798	928474

PG.Genes	n	mean	sd	median	q1	q3	min	max
CCDC184	25	36446	31529	28208	11305	49060	3394	134171
SBK1	25	17594	4647	17307	13799	19802	11437	33429
PDCD4	25	30151	18089	26349	18631	35092	11572	90246
FNDC3B	24	12513	5025	11320	9678	14874	1745	26692
CRTC2	25	34637	18167	33633	22820	38893	6867	74414
NCBP3	25	50998	32688	44269	36058	56395	16946	185868
TP53I3	25	55642	48217	42922	25854	70983	8361	239922
HIKESHI	25	12682	5723	11710	8855	15062	4786	31586
SLC44A4	25	16069	3858	16222	13044	17815	11005	27755
SLC44A4	25	239904	288642	179517	149530	212667	14027	1579875
PDLIM3	25	6511	3481	5940	3709	8781	1907	14797
LACTB2	25	13056	7922	10791	9200	14498	4598	39428
EIPR1	22	3234	2198	2763	1397	4943	673	7717
SMUG1	25	12692	6875	10937	7940	15275	2939	29132
SOWAHC	25	206511	165676	149373	114318	207935	61362	729074
LBH	18	3643	1581	3430	2540	4695	1551	6450
ARHGAP15	25	33695	11932	32516	25782	40968	11938	65008
FASTKD1	25	33501	29951	27210	20842	30755	12120	152914
ASPRV1	25	38199	15069	38956	28921	46597	6305	66572
HS1BP3	25	12892	14018	10167	7426	12938	4652	78282
INO80D	25	16183	4860	15401	13283	18730	5294	27953
ACTBL2	25	193395	71566	184323	161125	221363	62598	385668
CCDC93	25	57237	9202	56598	50054	60277	42906	79325
OCIAD2	25	49412	48811	37153	22918	44980	8406	192130
HSP90AB4P	25	163906	50664	147668	131382	187230	99871	302295
HSP90AA4P	25	18589	4310	18989	15654	21059	9623	26274

PG.Genes	n	mean	sd	median	q1	q3	min	max
TTMP	25	10350	5302	9466	7030	11328	4572	29602
NOM1	25	23180	7448	23683	17991	27044	9389	40576
ZC3H12A	25	4996	1689	5163	4082	5789	1946	9711
PDZD11	21	9229	6346	7041	4484	12055	2648	25017
CMPK2	24	5646	6578	4302	3792	4905	2182	35998
FBXO48	22	19728	7480	20162	13399	25809	2729	31258
TNFAIP8L3	25	52743	59124	34939	22002	52246	8021	262567
FREM1	25	26014	6446	25495	21988	28394	15187	46600
RTL5	25	103198	67423	87187	64756	115014	40140	356979
PABPC1L2A	25	63698	36135	57733	41299	71313	12641	143443
PRRC2B	25	10678	6431	9080	6792	11327	4716	29960
KLF17	25	86277	83200	50714	35751	119423	17770	314758
COA6	25	248421	294541	158308	120347	234223	84353	1541535
AARS2	25	30604	18018	27786	21781	32514	7018	98004
SPIN3	25	7878	3852	8136	5057	9715	556	15195
GNAS	25	5224	1877	4685	3837	6264	2368	9824
KIAA1755	25	38096	9529	36381	32294	40441	26415	67288
PRSS36	25	10262	8386	7526	4675	13565	312	37432
SAMD9	25	282978	109492	264557	219501	292371	192988	678057
DGKK	25	58647	30595	49474	39984	67720	22959	161663
SPECC1	25	13131	6209	10935	9277	15676	5669	33623
WDR45B	25	16782	5691	17672	12423	19899	8435	26948
EOGT	25	10146	6041	9695	6908	11175	3032	34106
NEXMIF	25	21257	8390	17495	15716	23589	10630	42048
TBCEL	25	4263	2868	3204	2287	4814	1721	14115
ELFN2	25	143769	31948	138339	119998	157262	98018	238050

PG.Genes	n	mean	sd	median	q1	q3	min	max
TTC38	25	84287	46745	80170	46638	113234	29869	178768
EXOSC6	23	5977	2250	6014	3705	7529	2770	10775
RBM48	25	3641	1685	3369	2459	4399	885	8148
LRRK2	25	53738	8045	51877	49775	54761	42908	83304
PHYHD1	23	3776	2155	2896	2205	4548	1473	9013
HP1BP3	25	15158	11397	11719	6973	19710	4131	55968
FRMPD1	25	73395	39376	64643	50338	78336	35554	232014
C6orf141	25	27000	11345	26987	17111	32361	8585	50990
GLYATL3	25	14936	6343	13993	10993	17647	5084	34870
FAM120AOS	17	2489	2549	1435	389	4082	146	8691
ZC3H13	25	53310	22228	47031	34798	69544	27974	108193
TRAPPC3L	23	7016	3254	6224	4604	8971	2843	14999
ARMH3	25	12964	7235	10732	9145	13335	6720	41778
IBA57	25	15735	10600	13005	8327	19655	4868	50960
HECTD3	25	16492	5311	15936	12769	19491	4938	29294
SFRP5	25	158018	82927	136646	91649	208767	51258	380772
UBR4	23	21702	21136	15034	11584	23689	6757	110653
UBR4	25	26104	4476	24912	23246	29326	15940	34068
ZNF684	23	7883	5427	6790	5932	7800	2890	30220
KIAA1217	25	1800468	775048	1564667	1269256	1900382	1139229	4280356
ADGRF1	24	17574	15838	13006	5566	26388	845	60841
UBAP2	25	67864	49970	56495	46888	65714	31431	290103
C9orf64	25	13136	4868	13507	9741	16376	4197	21690
KPRP	25	19967	6417	20262	17278	24862	7429	30401
XP32	15	7776	13739	3017	2309	5673	722	55147
L1TD1	25	465820	218437	376888	329603	535326	245282	1222692

PG.Genes	n	mean	sd	median	q1	q3	min	max
SYT6	25	272752	161175	197102	174277	310481	62837	801879
RNF187	25	10029	8076	7418	4708	11949	2023	32321
TTC22	25	7433	1462	7153	6790	8466	3824	10153
DCAF8	24	3250	1819	3006	2163	3661	843	8267
RNASEH2B	25	11311	4990	10147	8060	14020	4200	25137
RC3H1	25	13022	5818	11429	9452	15007	6299	28528
OGFRL1	25	137099	70954	137116	92152	175468	16440	301454
MAGI3	25	81285	19301	85831	72181	89765	41620	126878
SH3PXD2A	25	24572	3479	24071	21841	27305	19498	31655
RSPH4A	25	112152	35336	108038	89036	129526	57512	208054
DDI2	25	10069	4629	9145	7881	11237	4787	28209
C20orf194	25	36844	5852	36139	32490	40934	26045	49656
H3-2	22	6111	6092	3898	2132	6979	931	23851
C6orf163	24	7014	3484	5927	4517	8719	3142	18752
NT5DC1	25	20139	16571	16488	12497	20167	8345	87004
C1orf195	25	14294	8675	11084	9081	15955	5504	39311
AHDC1	25	49507	23805	41609	37152	52836	22332	140905
VPS13D	25	73180	10701	69910	64465	80378	55406	97045
CROCC	25	272529	42358	264359	242747	309363	205172	359266
AGBL2	25	128844	63367	102056	87965	151442	60516	354533
ATF7IP2	25	63087	29538	58120	43082	73307	35703	173984
RASIP1	25	16271	5321	14902	13616	16601	11196	33034
STRIP1	25	14377	2013	14292	13105	15881	10972	18794
SYDE2	25	123656	22153	115536	109681	141919	91059	175565
RNF220	25	7582	3651	6268	5430	8773	3307	19655
PRPF38B	25	27897	8913	24941	22727	31804	14214	58523

PG.Genes	n	mean	sd	median	q1	q3	min	max
RNF20	25	125382	18614	123641	112755	136986	86678	162133
ZNF318	25	31001	7858	29531	25810	33701	23135	60465
DIPK1B	23	6979	2385	6646	5155	8423	3467	12449
ATRNL1	25	16181	10322	13841	10633	19442	5883	59759
SPATA31A6	25	46533	46696	36351	31313	42824	20489	266788
BROX	25	12961	6642	11738	8736	14382	3789	35429
FOCAD	25	12847	6442	11630	9002	15191	6566	37328
TAF3	25	133731	70600	104601	86025	185088	43658	302819
PRAMEF8;PRAMEF7	25	11949	5125	9714	7700	15082	5436	26773
RSBN1	25	23659	7302	23552	17447	28429	14734	41782
LYPLAL1	25	17473	7258	17209	11656	21649	7820	37335
ECPAS	25	36922	9585	34629	30098	44581	22446	59869
TSHZ3	21	13818	6359	12253	8200	19267	5669	28234
KANK2	25	7998	6769	5367	4263	9217	1797	31361
WASHC2A	25	9315	8349	7107	6128	8999	4928	47695
EEF1DP3	25	67935	44391	54192	39063	87201	11990	189037
FAM91A1	25	12061	6351	10451	9042	12646	2004	30618
LARP1B	25	67213	23711	63455	53126	74957	30800	159432
CEP135	25	66277	17573	65132	54361	75279	39285	120772
TMEM198	25	155451	143899	110086	59573	187897	28502	536812
MAP1S	25	56479	16142	54936	40657	67411	33453	102708
E4F1	25	9153	6588	7272	5820	10105	3268	36879
PPP2R2D	25	31354	14694	28797	20103	38437	10605	64848
ATG9B	25	93609	20197	88842	83420	106663	56841	160732
NUGGC	25	357398	188622	312506	273469	357571	226603	1207557
TNS3	25	15286	4258	14246	12914	16522	9412	28283

PG.Genes	n	mean	sd	median	q1	q3	min	max
ANKS6	24	17751	9590	16672	11488	23181	2702	35512
LMBRD2	25	17918	11730	13486	11230	19266	7576	53470
ZFYVE26	25	966959	136368	930267	881786	1064440	714495	1266058
MSL1	25	11190	3453	10392	8812	12181	7367	20236
C18orf63	25	56293	14666	53494	45232	60912	37402	88464
CWF19L1	25	101703	50278	83768	70765	122224	52267	282490
CYB5R2	25	19138	13900	13751	9555	24296	2980	56214
UHRF1BP1	25	25095	9909	24163	18646	27957	12865	54853
SP5	24	28300	20657	22261	14392	28168	7339	81839
ATL3	25	58505	38411	49227	43156	68539	24144	228659
NXN	24	6620	5643	5160	4308	6959	1955	31320
ZNF470	25	15797	5626	14194	12781	18350	8673	35017
VASN	24	3823	2470	3640	2223	4557	993	12683
SFRP4	22	4111	4794	3183	1298	4892	506	23393
SMYD5	25	5666	2862	4872	4003	5767	2319	12816
PTRHD1	25	32660	13822	31944	24304	41734	10509	63087
NADSYN1	25	11533	4126	11579	8769	13208	5369	19147
LAMTOR1	20	2904	1302	2891	1729	3730	1278	6309
TWF2	25	9564	3852	9626	7585	11697	2782	18601
PHETA2	25	8154	5449	6562	4627	9049	2235	27070
TANGO2	25	14815	5086	14067	11686	16991	5309	27218
SPOPL	25	18795	12909	16710	12313	19521	7939	74520
RAB12	25	16291	4982	15541	12965	20286	5786	25153
KLHL10	25	9980	4255	9275	7354	12362	4430	22561
KRT80	25	44980	25263	35877	31063	50039	17537	143156
TET2	25	823401	129793	804762	753782	907239	573179	1096341

PG.Genes	n	mean	sd	median	q1	q3	min	max
NAA16	25	48285	12276	45686	39742	59739	26042	73058
PSAPL1	25	102019	21804	97970	85649	113741	65054	146290
TYW1B	25	14398	3769	13616	11829	16994	8846	25284
DPY19L2	25	93498	63681	67851	50572	120798	21743	300051
CRACDL	25	60952	23839	50323	45281	75566	36134	120846
NIPAL1	25	9590	10801	5837	5227	8991	2718	56435
HIBCH	25	19627	13943	14881	10943	22504	5468	67422
ZNF774	24	9541	7978	6677	4876	10296	3558	33573
ANKRD54	25	105188	90894	93370	53727	115026	14087	471629
PPP1R18	25	27556	7798	27266	20876	31458	16959	46874
CAVIN1	25	10958	7182	7787	6460	12308	3002	29884
ZCCHC8	25	26162	7824	24008	21567	28912	14957	49765
DHX57	25	64435	24236	55682	50748	75667	41610	137246
MRPL54	16	4090	4539	2372	2130	3305	704	15978
CDC73	25	11202	4857	9841	8529	13348	5956	26236
TATDN1	25	45591	27670	42684	30070	45340	13209	135614
METTL2B	25	10719	4309	10313	8045	13801	4390	19247
C8orf82	22	4956	5397	2875	1868	6239	782	24091
EDC4	25	46825	14882	47012	34629	55141	25481	80656
PRPF8	25	10516	2132	10206	9216	11541	7753	15364
NEK5	25	2629007	1113405	2407475	1931401	2893494	1539889	6679325
SCYL2	25	65984	19341	60986	53013	82203	31699	98777
TTC27	25	9320	2020	8654	8081	10052	6255	13315
PLBD1	25	294401	143803	263855	165026	400792	91522	571999
LMOD2	25	14201	3761	14190	11660	16058	5686	21368
NOTUM	25	10812	5318	9860	7694	14048	3918	26401

PG.Genes	n	mean	sd	median	q1	q3	min	max
GIMAP6	25	34968	22135	33811	23312	42143	11947	122840
VWA1	25	13478	7879	11330	8074	13023	6551	37835
PGM2L1	25	20107	10595	19704	13024	23853	7979	52277
CTR9	25	54796	13358	53953	49721	59563	26288	83218
TEX38	25	68505	38436	68463	39228	92729	12385	163959
TTC37	25	53324	10528	52556	47952	54905	38841	77973
DARS2	25	7174	2540	6708	5111	8046	4345	14544
FBXO46	25	21293	9378	19461	15516	25413	8056	46679
LARP1	25	18336	6458	16026	15413	20041	9857	37603
ATAD2	25	166993	62980	148613	118608	189705	101917	355392
CPLX2	25	6266	2509	5711	4496	6877	3442	13347
SLC25A47	25	24082	19724	17405	10983	31859	362	65593
SPAG17	25	51895	22040	45048	38799	62910	18584	109922
SPATA8	21	3576	4111	2421	1393	3791	882	19996
MTHFD1L	25	18924	3997	17388	16548	20885	13367	29146
CSPG4	25	42295	7122	41130	38139	44787	31744	66000
DHRS11	25	8231	4668	7221	5266	9771	2217	26502
SBSN	25	174494	29995	175526	148853	192086	125728	229500
ENPP7	25	17518	5386	16228	13485	19482	9841	32229
OLFML1	25	42931	24809	35184	25388	50558	16719	103565
CWC27	25	52226	14975	49734	44798	56342	22170	84573
OLFM4	25	65234	25661	55127	48635	80226	42134	143826
LAYN	25	38245	28825	29365	18520	49777	3007	120303
PLXDC2	25	83230	23070	79355	66895	95571	40143	151863
WDR82	25	23928	13395	22473	17007	26676	10122	79704
MUC6	25	56389	20850	51687	40770	66921	30165	103914

PG.Genes	n	mean	sd	median	q1	q3	min	max
RAB11FIP1	25	9009	3054	8455	7042	10440	5231	18417
ASCL4	25	54836	47566	42361	34592	55434	21643	265129
NAPRT	25	17554	9769	14638	12048	21164	4699	38774
GIGYF2	25	99926	26400	97886	82694	115566	64154	171071
CD109	25	452204	1368822	139963	122625	187208	94837	6996788
HSDL2	25	16040	8524	11663	9165	20941	8206	35094
KYAT3	25	8020	2698	7372	5730	9419	4611	13993
PPP1R21	25	7316	1717	6971	6241	8453	4349	10849
GLDN	25	12724	4511	11718	10136	14518	7404	30918
THSD4	25	130321	70110	113794	98405	144665	78215	443484
TMPRSS11A	25	93131	50941	87278	53976	126241	21074	227393
ZNF783	25	31970	21422	25287	20122	37455	12939	109489
TRIM72	25	62129	17818	58453	50961	69066	43922	129083
LEKR1	25	22081	11117	19010	16945	23755	10536	58374
ZNF782	25	30542	13746	27919	21255	34576	11929	67547
SPOCD1	25	55210	13862	53070	47820	62074	34926	97114
MEX3B	25	11165	6299	9730	6491	13214	3083	25741
BNC2	25	34375	13628	32134	25179	37045	17464	74922
GBP6	25	7944	2442	7275	5973	9360	5495	14789
CCDC81	25	109973	42046	93888	85313	106308	74513	260556
ZNF836	25	53547	9990	54589	47403	62076	35198	69691
RNF111	25	241876	94978	220428	167289	320065	107172	463695
FRRS1	25	218368	75788	194539	172905	273678	102706	363393
FGD5	25	36340	11616	32825	30056	38775	21767	77292
RBM44	25	6629	1689	6595	6164	7210	3292	10737
SLCO4C1	25	12229	7947	11965	7421	14263	2422	45255

PG.Genes	n	mean	sd	median	q1	q3	min	max
WDR87	25	40357	6393	41101	37164	43579	29518	61263
CATSPERG	25	16017	5177	14449	13045	19270	9090	31563
FAM83H	25	13585	3820	13328	11010	15838	7744	23069
LINC00696	25	99387	72799	82786	48784	137328	18144	354911
ZNF662	25	144928	68674	120262	95547	190072	60843	302844
LCNL1	25	6741	2462	6821	4737	7907	3078	12372
CDHR3	25	455652	118451	430496	390998	546574	243509	705785
CFAP47	25	293929	70904	276721	253329	322635	221612	581247
MSANTD1	25	8999	12155	5921	4142	6880	1943	62411
UBN2	25	34644	6676	33710	31358	39154	22231	52765
FAM205A	25	48672	45556	39198	29741	49733	10876	255419
CEP128	25	64985	9027	65164	61769	68818	47720	83870
MROH5	25	6886	2191	6558	5996	7353	4540	15853
SPATA31E1	25	649694	327874	587864	435205	705939	318953	1815755
CCDC121	25	7341	3391	6779	4700	9486	2141	14247
GPRIN3	25	10195	4852	9240	7750	11529	5995	29400
CFAP20DC	22	5534	2642	4738	3851	5793	3132	13095
NCCRP1	16	8610	14628	3108	1971	6118	1321	59589
MICALCL	25	6948	2446	6922	5332	8511	2326	13205
TMPRSS11F	25	50138	24424	40905	32091	57419	22782	101405
USP31	25	32667	11863	30316	24677	37847	13929	70072
RAPH1	25	77138	34198	68566	61362	83129	45750	216103
USP43	25	57149	17177	55230	48428	64768	30725	114184
UBE2R2	25	8506	3466	8233	6339	10405	3135	17070
CBLL1	25	23086	5373	23485	19861	27037	10412	30755
FBN3	25	155808	30977	151663	134633	172971	105679	239155

PG.Genes	n	mean	sd	median	q1	q3	min	max
MTSS2	25	76458	29627	70993	54331	94642	35008	156112
BRINP3	25	43279	17415	41354	29861	50200	17239	79429
SSH2	25	158157	165296	99949	78367	140957	61798	781593
CCDC80	25	17090	4472	16524	13712	20721	9316	27875
SUPT6H	25	177454	65630	165575	138135	194848	116174	399550
SND1	25	51521	24984	46133	33850	63374	9050	109787
DDX46	25	57421	16369	52702	47027	63371	36333	105639
TRIL	25	377109	211481	291923	235731	429466	90139	964390
TRMT10C	25	12842	3906	12314	10141	14668	7620	23290
CHST9	25	14172	6750	12122	10745	13481	7361	35807
ASRGL1	25	98135	80765	81760	33195	141848	2701	298599
KCTD9	25	18005	8571	15293	13090	21018	7276	45160
EIF3M	25	25106	11103	24100	17497	32853	8029	53803
MEPCE	25	5223	1389	4904	4237	6465	1989	8018
PARS2	25	118623	48384	103067	89281	131083	68047	300799
CYFIP1	25	79598	23852	78500	67770	97892	26698	116021
COPS6	25	13848	4831	13162	10217	16073	6187	24241
EPM2AIP1	25	2276	931	1977	1589	2813	1188	5179
TAOK1	25	107498	18521	106985	91095	122911	77332	141332
KDM3B	25	48651	14817	43480	39050	49897	35037	89034
CHMP1B	25	77158	34787	75122	51008	93518	23461	154640
CHST3	25	20142	15281	16241	14204	18207	9225	87543
OTOP1	16	826	518	709	420	1068	256	1949
MICAL3	25	238632	295152	193605	95303	254887	35315	1563681
PHF5A	25	3801	2259	3245	2326	4206	1397	11932
OVCH1	25	63920	23050	59267	52863	65804	35429	156012

PG.Genes	n	mean	sd	median	q1	q3	min	max
OVCH2	25	26887	11027	25696	20032	32109	7208	52507
TRMT1L	25	49204	20059	46886	35841	54721	24462	115003
ZC3HAV1	25	74174	34802	66760	56315	80905	37493	204523
GVINP1	25	24845	5484	23793	21615	26084	14738	40111
NEGR1	25	34581	26791	32049	17147	41663	6015	131569
NUP54	25	65339	17783	65079	55007	75043	33089	99873
DGLUCY	25	10395	3611	10104	7517	12632	4075	16882
CCDC186	25	34297	16257	27933	22235	45451	18104	75215
VPS35L	25	24569	9613	21148	19270	28495	13437	55056
ZFYVE16	25	44767	10550	44568	36018	50478	24922	64738
MYH14	25	62356	16900	60401	50490	72261	41848	104383
NUFIP2	25	12299	4773	10221	9029	13558	6537	24459
SZRD1	25	20876	11701	16953	13341	21194	8125	55203
MAVS	25	6139	3248	5285	3722	6968	1730	14602
PKD1L3	25	291367	60607	288238	253570	336826	160087	416406
DHX29	25	200181	45177	198187	164699	228519	125406	295484
NPHP3	25	11114	1682	11067	9821	12549	8615	14452
HDDC2	25	10243	4766	9400	6707	13105	3215	20384
POGLUT3	25	29176	11817	27488	19438	36219	13274	55488
LIMS2	21	4779	2656	3794	2953	6818	429	11649
LIMS2	21	19528	12785	18383	6454	28757	1264	43935
DCXR	25	47448	36238	35435	28238	46166	22879	177008
AKNA	25	67471	15223	65488	59661	76838	38923	100172
TAFA1	16	82228	87072	52276	31904	92442	13443	352404
POLN	25	91173	33340	84833	70935	97899	52608	194915
PPP1R32	25	101595	34899	96657	84663	121952	25202	195137

PG.Genes	n	mean	sd	median	q1	q3	min	max
SNX20	25	247972	103069	273051	213384	318136	877	404037
SPRED2	25	34336	25651	26342	17402	40819	12147	121692
ARPIN	24	6712	3505	6508	4677	7964	1629	15808
HUWE1	25	72319	26606	65301	55302	87244	39824	148278
YTHDF3	23	4372	2699	3748	2142	5516	1102	12480
CTU1	25	37598	11269	37589	30249	43661	10523	66019
ABI3BP	25	34271	22422	30974	21826	36557	4661	104100
ZNF467	25	36801	6977	36520	32084	42041	23190	49134
C11orf96	25	348710	167438	294427	252778	376732	203484	966466
MEGF8	25	31180	8635	26779	25087	35071	21674	54320
GALNT5	25	146136	66313	134527	111276	153193	70942	419477
GALNT7	25	33457	14784	28796	26768	35035	17409	92966
PHLDB2	25	151689	61363	133663	107392	182076	71115	316460
WDR86	25	9639	2116	9296	8534	11541	5768	13793
SETD3	25	363769	183231	331764	271643	377035	137796	933009
TTC7B	25	28826	16376	24352	20007	30822	15653	98367
ADCK1	25	126307	75287	111499	93898	126008	68401	460421
ACOT1	25	94382	29030	94093	73472	111795	45334	169368
NOP9	25	15279	11385	11401	9276	16869	3423	57391
METTL3	25	4894	2456	3996	3225	5491	2012	10254
PRPF39	25	20901	6228	20458	17069	24214	11658	39983
OAF	25	20357	7962	19733	15567	24699	9422	43435
ZNF546	25	18151	6471	18042	13219	21497	8635	29089
MTDH	25	24864	11623	22340	16599	30684	13474	67245
LRRTM1	20	5439	2761	4599	3843	6719	1349	12504
ABCA12	25	122317	23622	129116	105232	139907	76657	159213

PG.Genes	n	mean	sd	median	q1	q3	min	max
RTN4RL1	25	7683	3103	6949	6409	8235	3806	18003
RTN4RL2	25	4850	2160	4296	3250	6090	2164	8741
SMG6	25	87402	30191	86950	63482	99948	36652	156633
BCL9L	25	6475	1594	6383	5577	6895	4237	11214
ITIH5	25	13917	8046	11557	8331	16566	5800	37212
FERMT3	25	24882	23046	21361	13486	27284	7394	127409
LUZP1	25	43081	11976	39235	35641	44749	31966	88492
ALYREF	25	62462	33049	58135	45501	74861	10852	148594
FAM160B2	25	6803	2630	6034	4985	8317	2636	13149
ZC3H18	25	38684	26463	32129	28474	37964	19715	158893
VPS36	25	5191	1834	5157	3811	6903	2250	8525
CAND1	25	60822	24142	58300	44362	79224	17905	102195
GLCCI1	25	49963	23946	48063	37329	54383	16991	121816
TXNDC2	25	27469	15836	21376	17952	35024	6496	65781
HOOK3	25	112477	31723	114318	100490	120264	48749	230442
COMMD7	23	3852	1450	4023	2848	4606	1457	6476
METTL16	25	5130	5419	3761	3258	4285	2076	30003
ZC3HC1	25	28024	9513	26413	21415	34227	9820	49165
PKHD1L1	25	157208	95652	131584	105435	172745	85714	569413
CCDC25	25	11081	4882	10128	7883	13248	3968	22591
CARM1	25	9554	4665	9017	6572	12161	3107	20402
CIR1	25	11580	6019	8842	7617	12706	5491	27651
FAM131B	20	4472	7869	1926	1323	2859	554	35651
MICU3	25	29129	7709	27869	25282	31173	16231	48457
ZNF575	25	15750	5117	15377	11374	17883	8761	29650
PPP1R3B	25	22209	13629	16290	13144	31262	6235	58161

PG.Genes	n	mean	sd	median	q1	q3	min	max
ZSWIM9	25	63480	21656	60680	45144	76109	35528	116021
ANKLE2	25	66394	19866	66493	53219	80029	29651	99863
DZIP3	25	35778	13886	31840	27347	38304	23215	89462
STX12	25	15458	11158	13491	8205	16487	5766	52865
ZGRF1	24	533314	539133	369710	237346	516728	168772	2610354
ERO1B	25	24439	9063	21949	19066	28162	11966	56115
ZNF280B	25	12981	5013	12546	9500	15317	5365	27998
MARCHF9	25	37408	7191	37515	32813	42074	22639	51868
ANKRD13B	25	14256	5814	14104	9739	16692	7253	34909
TMPRSS6	25	51537	27251	42695	32707	65977	18057	135465
IRF2BP1	25	160848	71206	132188	107458	200955	65389	323877
RHPN2	25	30197	14240	26202	22200	39377	5236	61889
TGIF2LX	25	90397	87827	62302	46609	84409	27752	447725
H2AC21	25	71791	20212	72212	59169	82451	24496	126742
AEBP1	25	1479256	586601	1250830	1160965	1635505	373412	2977457
PHYKPL	25	264210	64516	249695	235735	280728	187187	469665
PLD3	25	31822	27349	22834	14718	36312	9804	132040
LIX1L	25	132791	34197	127141	109048	145519	88127	254155
ZNF584	25	237541	299852	174370	134517	210806	101846	1647518
NUDCD3	25	11623	5851	9521	7911	14969	4011	27737
MMAA	25	10882	5956	9892	7592	12195	3767	31713
P3H2	25	26440	15837	21351	19163	27074	14283	89372
P3H3	25	24307	9040	21923	18142	25723	14198	48661
MUSTN1	24	10741	7550	8807	5760	12703	2322	31074
MISP	25	43248	19889	37982	32522	44843	28488	129170
IGDCC3	25	69272	26387	61252	53837	76169	37183	163229

PG.Genes	n	mean	sd	median	q1	q3	min	max
CDKL3	25	14923	9010	11906	9623	14819	7682	46966
APLF	25	245645	48131	243053	211754	282274	167369	367090
MAPKAPK5	25	24399	6989	23463	20577	28209	8050	37675
NAXD	25	7617	2968	7119	5211	10283	3730	14039
NAXD	20	3738	2651	3345	1799	4468	628	12728
WDFY1	25	24676	9476	24357	17935	29388	10730	49650
TEX2	25	176177	52138	154964	140102	204990	112734	339102
FAM114A1	25	56960	25761	52238	41446	61106	26580	148912
GCC2	25	108952	30725	97178	88487	126102	74252	188663
HSCB	25	45855	15016	42164	35210	56670	23947	85667
CUL9	25	273125	112056	260621	215481	314125	137640	732402
LMTK2	25	45962	10245	44968	41005	50623	28147	81840
SULF1	25	4481	1282	4436	3682	5162	2346	7254
UBR1	25	23936	10275	21667	17104	24991	13364	58292
CHERP	25	188001	145031	155418	130718	181245	80543	843308
SCUBE1	25	16138	17501	12169	7714	15960	2954	91132
TRIM42	25	12415	3911	11423	10013	12908	7537	20845
SUGP1	25	174690	41821	178296	141340	205354	104205	252976
UEVLD	25	8291	5375	6571	6079	7894	2049	29271
CCAR1	25	43823	17822	40572	32033	49149	22572	102302
DNAJC10	25	27816	14663	25088	16076	36117	8870	70856
NELFCD	25	48945	31025	38087	33313	49206	18259	159126
ASXL1	25	38357	23515	33533	27626	37044	23349	142015
PHC2	25	37207	18117	36770	22075	49957	10612	76975
MSRB3	20	4097	4108	2401	1733	4484	830	17318
IQCF2	25	40064	23754	32697	27625	47054	6487	89751

PG.Genes	n	mean	sd	median	q1	q3	min	max
MRPL41	25	8834	5491	7205	5687	11467	1177	24975
GPALPP1	25	6552	6210	5402	3345	6958	1665	33516
ETFBKMT	25	365147	317138	267217	188264	414502	93590	1677466
FAM217A	25	38360	19008	32218	25988	45241	15365	94210
RBM12B	25	28798	12930	25583	20278	32983	14104	67682
ZC3H3	25	39334	12901	35478	31354	48646	9044	66819
DDX60	25	69675	18124	68074	59677	71577	47187	135144
MICALL2	25	24948	9113	21659	19726	26832	12084	51210
RAVER1	25	14911	4454	13911	12364	16602	6058	24267
FTSJ3	25	26144	11284	23822	17688	32536	9503	53371
MKX	25	29964	11830	26827	20853	35145	16056	58612
DIS3L2	25	68225	55396	53546	45972	69790	25498	319551
ZNF595	25	16067	5819	16787	12569	18792	6876	35195
LAS2	25	233509	98045	203145	162711	291142	105552	488663
EXOC8	25	13212	6385	11370	8785	14994	4544	26344
TRMT44	25	8612	4810	7499	5448	9551	3791	22032
THNSL1	25	75666	17612	71718	63020	85872	54572	124760
CFAP206	25	141440	53124	133130	109822	154500	80730	314632
PM20D2	25	10970	5726	9725	7300	14001	2431	27359
HACE1	25	488977	233255	443214	335259	517403	271113	1393121
MICU2	25	7315	2443	6694	5950	7458	4220	15000
CHPF	25	50613	12760	48300	41989	57534	32972	82029
NRSN1	25	26689	9132	23815	18744	32193	15927	48181
ALDH16A1	25	74517	28180	73249	53443	94008	20003	151015
RTKN2	25	121813	90580	92686	65614	146583	24773	382242
ADGRF3	25	28463	22466	22606	15821	33583	7891	112277

PG.Genes	n	mean	sd	median	q1	q3	min	max
PELP1	25	5943	1074	6037	5240	6727	3591	8185
ZNF654	21	13549	13229	8540	6116	17041	590	61722
C1orf87	25	31549	13308	29565	21686	36942	14604	69755
SPART	25	26612	7867	23705	21839	32036	15244	45416
PGAM4	24	10848	4896	9310	8213	10849	6935	30108
CNPY4	24	4404	3458	3246	2300	5145	1158	17904
ADSS1	25	22222	4148	20633	19435	24365	16062	34363
AHI1	25	62353	14631	58036	51387	66858	48650	106582
NAGS	25	219983	87190	218034	159684	243750	95178	448491
ZNF567	25	9016	8175	7521	6375	8687	3891	46582
CARF	25	53074	74359	30052	23022	44685	11371	379547
C4orf33	19	4072	2597	3651	1972	5037	321	8838
C1orf158	25	9487	3172	9937	6303	11366	4502	15501
STK11IP	25	10764	4820	9510	7668	13203	3329	23741
LRRC47	25	30500	9679	32008	23817	36961	11973	49781
VWDE	25	47414	8818	47399	38538	52861	34574	63566
ZCCHC24	25	7917	1676	7934	6765	9302	4542	11213
GHDC	25	11813	9563	8383	6790	15425	2914	49547
OSR2	25	10522	5740	8283	7025	12911	5508	30603
PIAS4	25	19392	7918	19174	12911	22288	7192	38354
GPD1L	25	49095	23354	42299	30765	64260	22874	128964
EHBP1L1	25	80230	27254	69561	61606	85627	50556	161849
MICALL1	25	15229	4381	14228	12367	18115	7887	25176
TXLNB	25	140623	92678	111012	80239	148830	52249	414068
PHTF2	25	110903	58327	102197	78047	118845	56346	348522
FNBP4	25	6242	2371	5942	4276	7813	1432	10116

PG.Genes	n	mean	sd	median	q1	q3	min	max
GUF1	25	910000	527702	743355	550351	1236665	436662	2714967
RIBC1	25	27670	17629	23473	15439	33316	10146	88554
SFRP1	25	28371	16424	24943	19675	32090	7768	79127
LIX1	25	7223	3463	7028	5269	7978	3056	18686
PNKD	25	17572	5179	16922	14419	19776	8313	29727
GALNT4	25	35336	7985	34639	31387	38027	20453	51784
BPIFB2	19	18052	31181	5712	3287	16871	1801	132568
MARVELD2	25	208903	177450	136208	101489	241482	56452	848769
CPA6	25	6464	2674	5926	5150	6459	4308	18251
CBR4	25	5482	2021	5315	4257	6232	1876	9904
AFAP1L2	25	14639	3338	14371	12481	15442	7081	21747
TTC39C	25	32188	12789	29482	24956	36501	16580	77498
FAM89B	25	188374	195740	122482	90747	238230	12763	953967
ARRDC1	19	2332	1957	1615	1186	2478	946	9137
SLC25A41	24	6068	4561	4389	3619	6937	1859	22437
CPSF7	25	106461	51870	95429	76740	114585	53546	323203
ESX1	25	22606	6097	23028	18157	24147	14880	43941
ARFGAP2	25	8291	2661	7768	6355	8982	4337	16277
OTUD6B	20	2604	2441	2023	1364	2680	893	12171
SIRT6	25	79459	69585	63764	40854	89396	26793	349667
CCDC185	25	21247	10095	17407	14412	26551	6660	48273
SLC66A3	25	71002	43139	60496	49857	84874	11965	214924
ZNF525	24	14785	4672	14316	11191	16268	8819	26029
PAF1	25	17345	7198	16564	12990	22168	7658	37233
PAF1	25	44092	21850	40211	31056	45402	19444	109676
ZNF283	25	99708	57266	82062	68132	104854	51014	306068

PG.Genes	n	mean	sd	median	q1	q3	min	max
ADGB	25	44084	8473	42493	39558	45396	34170	73518
ANKRD31	25	9097	2831	8491	7945	9279	5186	16642
UBR7	25	3086	1750	2522	1836	3491	1423	8852
ZNF614	25	30122	15551	28547	14187	43764	7372	58541
DDX51	25	36570	22431	35380	22300	45983	11089	123564
KIAA1958	25	7591	2615	6797	5395	9178	4423	12786
PTGR2	25	7091	4102	6560	3604	9017	2340	20911
SLC25A29	17	8754	4822	8066	5264	10900	2313	19573
PNPLA1	25	6296	2431	5912	4739	7208	834	10936
MAB21L3	25	40563	14730	37227	31435	47321	12027	71640
ANKFN1	25	6780	4030	6069	4633	7357	2483	19581
ZNF709	25	171264	35905	177295	154098	192783	83519	229257
IGSF22	25	63836	32004	52875	44500	60948	27560	160843
DTX3	25	230181	100832	198836	165116	269482	56395	424247
ASCC1	25	95141	97357	74163	64648	89391	28420	550895
LRRC57	25	101603	32853	98934	85387	114694	36164	174996
FSIP1	25	100156	50707	80174	62811	121880	39673	249406
WDR31	16	11913	9199	7410	5229	20665	2998	29919
C12orf50	25	32097	20164	24843	21041	34390	13916	106946
TEX45	25	39693	15997	40698	25760	50601	13158	74601
FAM47B	25	20706	6413	18755	16944	22313	13890	40673
MARCHF10	25	111546	28678	112630	90384	119582	63668	177143
ZBTB38	25	141993	55099	122969	110444	173103	75590	338561
PRPF38A	24	11722	44732	2566	2051	3083	45	221662
KDF1	25	144584	174527	108843	57454	147026	29130	918774
MLKL	25	29754	8466	26319	24391	32917	15843	48312

PG.Genes	n	mean	sd	median	q1	q3	min	max
NHLRC2	25	24208	9385	23627	18629	26132	6478	50344
AVL9	25	6277	2500	5898	4732	6688	3713	15393
COLGALT1	25	49819	22162	43549	38182	55483	22029	119769
POGLUT1	25	6498	2072	6323	4716	7668	3301	11532
SLC4A11	25	98705	118738	70460	44761	93386	16256	629578
TXNDC5	25	40036	43065	28108	14966	46670	5326	220603
RNF149	25	16065	12081	12393	11266	16208	7135	68283
LEMD2	25	16407	10357	12707	9679	22804	4518	46822
NOA1	25	41396	13237	40170	31970	47793	25233	80323
FAM98A	25	5690	1967	5362	4178	6351	3159	9957
MTMR14	25	20126	15326	14789	11297	20622	9387	75461
NFATC2IP	25	14462	5681	12936	10817	18997	6939	26062
GALNT6	25	13154	4923	12379	10545	14753	6057	25960
NAXE	25	30770	17998	26340	20660	39830	10368	84830
CARMIL3	25	123288	55913	113738	91159	134631	61429	350206
RNF214	25	15941	3977	16312	11863	18055	11256	23416
LSM14A	25	40800	23394	33626	29717	43130	18877	138387
C3orf20	25	8276	3133	7541	5810	9967	4279	14324
MROH1	25	34188	4714	34262	30051	36243	26554	45066
MAPK1IP1L	21	5495	4074	4421	2155	9763	569	12887
TNRC6A	25	345958	143201	295862	263217	357294	215842	793296
ZNF738	25	75711	24609	80998	55218	89644	34690	130343
ABCF1	25	16744	7305	14643	10572	21001	7697	34851
ACRBP	25	21382	17118	17370	11549	22983	2613	84309
PIK3C3	25	565406	206895	467943	444323	614371	401942	1207617
CATSPER1	25	21324	7427	20791	16930	24959	8091	44008

PG.Genes	n	mean	sd	median	q1	q3	min	max
TTC16	25	24439	8236	23355	21260	25896	16695	60693
NGDN	25	13869	4355	13576	11037	15531	6370	25446
RTL9	25	19032	4729	18424	15727	20450	13606	36205
SDR9C7	25	5564	1282	5845	5336	6168	3008	7795
SPATA4	20	1803	655	1704	1348	2252	682	2818
FBXO22	25	25516	9975	22195	20270	28400	14191	65640
BOD1L1	25	367086	66781	356410	329968	404257	255374	602315
ANKK1	25	161187	63261	139945	126899	171002	88625	365773
NUP37	25	5089	1976	4511	3998	6133	2312	8928
NUP35	25	78141	18974	75124	64742	84318	57157	134512
REPS2	25	12942	2832	11969	11410	14427	9193	22807
GPRC5A	22	6631	4241	5187	3200	9471	2073	16766
GJC3	25	12205	5120	12774	7345	15616	3985	20318
MDGA1	25	8551	6379	7230	5945	8382	3710	36769
NBEA	25	220903	25126	217240	204531	234089	178293	298252
CCDC148	25	51358	19486	48633	39967	59983	17291	96865
TSTD1	25	15038	9365	13305	8988	15662	5180	39320
TSTD1	24	26075	15456	23385	16562	30388	6654	83546
CMAS	25	26727	26642	18516	14383	33160	5432	142442
TRIM58	25	24562	8014	24636	19018	28229	12331	40654
KNL1	25	40572	7156	38658	35276	41836	31081	56620
OR56B2P	25	5030	1740	5093	3789	5786	2319	8441
OR13C9	24	8758	13497	5801	4564	7368	1720	71242
OR6N2	25	156735	136831	122211	67517	164508	39738	611944
OR2T3	24	3713	1317	3420	2916	4486	851	6574
OR51A7	25	50885	30250	44569	31660	58374	19882	164886

PG.Genes	n	mean	sd	median	q1	q3	min	max
AKR7L	25	62156	21414	60295	45044	76235	27118	120592
PLBD2	21	19012	14387	11852	8674	32069	1257	52651
SPATA22	25	16584	9832	13259	11321	16780	8191	55733
TDRD7	25	2505287	1177618	2179796	1819838	2729522	1033603	6965386
GIMAP7	25	16355	11259	14970	9422	18808	3862	54649
GAS2L2	25	73591	23404	65147	56386	82346	45271	141753
HUS1B	25	122679	67443	96033	75473	143413	57265	284960
THOC2	25	34398	9162	32845	28377	35615	25802	62119
WDR36	25	35046	11540	34174	26587	39972	21604	69554
COQ8A	25	5665	3416	4404	3674	6267	2719	18212
SLC30A5	25	17574	9369	16315	13024	19108	6592	55026
GADD45GIP1	25	17087	4526	15968	13983	19108	10999	28733
WDR48	25	17160	4876	16285	13800	18816	11049	28484
TYMSOS	24	38243	19379	37523	25192	48697	11513	81995
SMARCC2	25	11097	4188	10585	8510	12683	4420	23377
NPLOC4	25	302563	559512	189741	156413	233398	85596	2976020
NA	25	16812	7489	15244	12791	18973	3642	36259
ZNF519	23	12861	10892	10019	6226	13813	4183	54268
PIGX	25	11625	4613	10936	8213	14144	4185	19854
TBC1D16	25	14776	7556	13661	9856	17502	6323	36346
CIRBP-AS1	23	9173	4406	8423	6101	10655	3736	23118
MDM1	25	13449	3404	13442	11024	14228	9205	23940
FAM71B	25	29939	11540	25512	21682	38079	16397	62290
ACTL9	18	9504	5268	8539	6168	13653	412	19598
NT5C	25	35624	18065	30824	20440	46309	9981	74360
PNPT1	25	17769	3922	17218	14492	20085	13043	26245

PG.Genes	n	mean	sd	median	q1	q3	min	max
AGR3	21	15952	14901	12663	5294	21061	2103	65995
NEK9	25	20350	4897	20016	18122	24643	10612	32179
ZNF675	25	20726	5450	20783	18593	22942	10579	32211
GPT2	25	35810	13547	32280	28569	36704	16641	87357
PLEKHO2	25	6224	3076	6222	3353	7564	2116	12676
MAGEE2	25	40151	23314	30488	27997	43877	17595	126019
ACTRT1	25	30684	11815	31830	23041	35844	11373	64990
BPIFB1	25	65314	60991	45030	38215	68431	14663	325478
BRIX1	24	3463	2395	2550	2123	3904	832	12728
RNASEH2C	25	7467	4421	6895	4057	9235	1538	19157
GNPDA2	25	21713	9955	20168	16158	24531	9799	49267
FAT3	25	122362	21646	117416	107066	139535	84392	165544
NEK7	25	5524	2477	4990	3608	7885	1580	10984
IGDCC4	25	56792	14055	59820	50252	64939	25841	85972
MICAL1	25	104986	62845	81403	70599	109521	48032	283227
PANK1	25	30968	9052	29122	26249	33897	13759	58009
TAS1R2	25	20552	9849	17335	13827	22548	10057	53309
ADAMTS17	25	25049	9552	24318	19916	29931	13217	54891
ADAMTS15	25	131140	119748	101239	80380	126159	46253	624255
EPS8L1	25	14105	4597	13952	11440	16134	7402	26550
DNAH5	25	127406	42087	114543	102621	136933	92798	289357
SH3TC1	25	14166	2544	13667	12419	15171	10699	23197
NSUN6	25	53784	13909	53082	44704	59174	33374	95199
TBCK	25	295829	168706	237852	221232	319367	157723	1016940
DTD1	24	21066	17556	16783	13997	22326	8758	98950
IPO4	25	42129	17930	37623	27841	51893	17662	95456

PG.Genes	n	mean	sd	median	q1	q3	min	max
PNISR	23	4128	5136	2801	1921	3657	1548	25449
WHAMM	25	65826	30298	57507	48305	72812	35253	161960
ZNF483	25	9038	1825	8832	7552	10361	5643	12287
FNIP1	25	13191	3638	12722	10532	15700	6404	21690
SHROOM3	25	254818	70563	228825	200486	318305	149722	407281
SETD7	25	20650	8011	18995	15052	22530	6753	38419
DDI1	24	6030	3044	6165	3719	8138	1292	13738
STK32A	22	9184	6031	6762	5514	10172	4224	30037
C7orf33	25	7343	2627	7211	5057	9189	3047	13954
SCFD2	25	146357	70452	128349	98607	155927	63462	352036
ZC3H15	25	68782	28081	61607	49649	86123	19627	135672
PPIL4	25	81383	27662	77873	67406	92190	33933	155962
PPP1R13L	25	30635	19677	24887	17471	35174	10410	94360
NUP133	25	53424	18876	47467	43294	57907	34570	114758
PDCD6IP	25	55152	15250	54241	46162	65132	27767	88089
FBLIM1	25	94151	50936	79984	66516	94925	53277	277825
SDR42E1	25	7002	2879	6408	5522	7497	3686	17702
BRK1	25	6134	2663	5742	4506	7206	2472	13177
DEPDC1B	25	163904	64675	146313	114528	222747	53020	297444
LTO1	25	18368	12939	14163	12073	20378	7456	72565
PTCD2	25	65505	29906	50026	46682	75942	38986	155511
AFG1L	25	39923	35288	26991	15389	39427	7350	138299
LEO1	25	10027	5865	8388	6434	13319	3851	26453
OSCP1	25	17708	3880	17197	15381	19096	11501	25841
NUDCD2	25	7604	3875	7337	5070	9525	555	15183
SCFD1	25	7755	2521	7583	6211	9099	4347	16112

PG.Genes	n	mean	sd	median	q1	q3	min	max
C4orf3	25	34793	12001	31846	28680	38929	8506	58710
UBLCP1	25	140636	39159	136165	110141	177602	67945	207864
TEKT4	25	39244	25082	30573	26170	46122	16416	131732
ZFPM2	25	40468	20244	35267	27707	42978	23212	110550
SPRYD4	24	5736	5252	4467	3838	5358	2380	29263
CAPSL	25	31931	14596	31852	21345	34168	13113	76751
CYGB	25	11461	5460	9643	7037	15413	4070	22092
PHIP	25	105995	24072	101814	88931	115255	68723	163072
SELENOM	25	8298	4129	6810	5343	10078	3091	20331
PRPF31	25	15428	7603	14710	11189	17062	7134	46042
PALLD	25	43251	19543	37465	26802	59675	20017	84253
SREK1	25	5253	2582	4640	3968	5147	2510	13311
METTL21A	17	3591	996	3742	3111	4039	1560	6037
COPS9	25	6682	2223	6180	5501	7255	3389	14472
SCG3	25	19637	12755	18517	12320	21231	6173	74622
SRSF12	25	64145	13095	66064	56353	75193	38763	92509
PSPC1	25	15011	7469	14162	10147	18467	6236	38444
RSAD2	24	8234	3060	8012	5831	9931	3576	14859
JPH3	25	77974	26754	73341	57373	97228	31619	134568
MUC16	25	1251141	382983	1173950	1045249	1315872	842408	2896588
DNAJC9	25	5851	2181	5462	4143	7117	2770	11587
THAP4	25	26014	13321	21551	19316	29311	15640	79089
TTN	25	1225967	213110	1143941	1075104	1301510	1026950	1891973
OVCA2	25	2823425	3068512	2143782	1511930	2814977	916317	16876094
LZIC	25	51085	18570	47460	43114	52211	30170	122078
IRGQ	25	51313	24811	44965	40611	53155	24203	143657

PG.Genes	n	mean	sd	median	q1	q3	min	max
ST8SIA2	25	15208	5985	13754	10718	18973	6428	27327
DDB2	24	2935	1457	2968	1708	3645	492	5718
SMPDL3B	25	13106	9159	10071	8764	15042	4734	50973
DDX1	25	41695	14580	42858	34756	47697	15525	79063
HSD17B8	25	4709	2576	3727	2885	5629	1970	12176
PIEZO1	25	107035	47973	98976	80382	119336	45875	300408
FAM3C	25	10222	10509	7148	5386	9739	2730	55097
H1-10	25	94405	50999	81444	69348	96254	44230	291531
PSMF1	25	19400	5750	18098	15808	20395	13368	35563
GBF1	25	4538	2243	3691	3353	5059	2791	13172
NCSTN	25	7859	3937	6865	5854	8605	4079	24668
MRPS27	25	16645	4752	15876	12948	18807	9728	26422
ELMO1	25	35789	12035	32248	26688	43503	17614	66129
FIG4	25	45632	8747	45702	38131	48620	32360	74792
DCUN1D4	25	6585	3442	5988	4554	6987	3102	18230
AP3S1	25	13424	4756	12812	10457	14820	5706	24510
UBXN4	25	9333	2603	9079	7025	10725	6223	18152
CNOT9	18	2347	929	2229	1619	3027	1064	4521
DOCK2	25	42310	11581	39677	34395	46300	31362	79008
TBC1D5	25	18699	17117	16008	13705	16950	10098	99688
LARP4B	25	7045	2968	6827	5655	7845	3244	19069
GCN1	25	56020	13416	54997	44340	64621	33778	82198
ARHGAP45	25	22681	15369	18941	14689	24293	7489	81368
DHX38	25	166799	75580	146688	124668	190344	89192	486858
NUP205	25	29893	8775	28525	25858	32787	18591	66881
TTC9	25	9599	4910	8417	6657	11597	4539	26279

PG.Genes	n	mean	sd	median	q1	q3	min	max
PXDN	25	14291	6276	12620	10813	16435	7835	39914
PIGK	25	16378	5917	16076	12974	17858	8814	39543
GTF3A	25	25689	21366	18499	9048	37066	1446	73121
SORL1	25	50821	21616	45251	37227	53183	29201	121041
ANP32B	25	23603	13091	20504	15568	29955	2743	62038
RABGGTA	25	28482	11755	27438	19938	35455	10738	57377
USP6NL	25	22669	14643	19028	16879	23253	11860	89456
HTRA1	25	36657	8489	36503	30513	40872	20050	56721
ARPC1A	25	9435	5721	7823	5234	11864	2175	25355
ARPC1A	25	19878	10674	18182	12088	23094	6111	48345
TAF4B	25	11878	4157	11777	8278	14972	6022	19150
FRZB	25	15539	5996	14240	12072	20004	1879	27427
HDAC2	25	13867	5987	12440	9393	20063	5720	25841
DPF1	25	53333	34454	46550	28528	65012	22709	182023
STAM	25	21262	5984	20160	17324	23624	12790	36796
PROX1	25	40862	22567	32963	26286	48106	17328	93728
SYMPK	25	10372	3669	10218	8215	12548	4412	20227
SYMPK	25	15889	5635	15081	11577	20390	8747	29120
TAF15	25	11130	6554	9491	7083	12482	5309	35427
GGH	25	26519	57600	12234	6365	16795	2448	288141
DDX17	25	14021	10965	12360	7174	17201	928	43637
NEO1	25	29152	12098	25152	23188	30086	14473	64904
APBB2	25	25438	5825	24435	21951	28583	14327	43711
OSTF1	25	15982	8530	14512	10726	19816	2584	43395
ABCC2	25	77539	81364	53783	44831	80206	28417	450601
ERCC4	17	4508	2773	4265	2898	5408	1555	12916

PG.Genes	n	mean	sd	median	q1	q3	min	max
UFD1	25	15525	9548	13973	10594	15720	8098	56966
RPL3L	24	6649	1348	6875	6027	7298	2948	8908
COPS5	25	17858	8849	16850	12517	21834	7278	44387
GPKOW	25	21580	6610	19817	17776	24790	7800	36037
SMARCC1	25	4265	1381	3759	3285	5012	2498	7944
RAB8B	25	26404	8389	26067	21083	32486	11790	39293
BAD	24	9316	2935	9248	7408	10874	4884	14880
KHSRP	25	70556	38666	64361	42321	82842	13593	176621
KCNB2	25	26016	15616	21881	15170	32067	8903	78689
GLMN	25	46986	16641	42705	35002	53383	13893	83307
USP9X	25	76054	18460	73828	63267	84172	48967	130155
USP7	25	33506	10338	33433	25589	39402	17796	59569
CUL5	25	89048	26223	83069	72459	105004	40460	160196
LPP	25	101381	93953	56868	28612	136126	14243	330476
RBPMS	25	7631	3230	7126	6046	8347	3331	17036
HGD	25	40927	40738	32464	18971	42714	7958	207674
MR1	23	4488	2514	4327	2725	5944	985	11288
TCEAL3	25	9287	4597	8541	5572	12457	4033	21976
TSR2	25	46661	36291	37512	23124	46928	17425	184106
CAVIN3	25	67006	32029	54976	41644	85224	27950	132259
NKD1	25	20308	14162	17954	15053	20138	11587	86398
CNKSR1	25	23102	15129	18765	16461	24533	11541	88009
MYDGF	25	27830	20512	19052	17308	34108	10211	101572
OSBP2	25	103631	49384	91946	78268	110281	60591	317875
WBP2	25	5871	2574	5221	4291	6148	3150	12934
NCLN	25	24769	11455	24578	16954	31281	7772	53056

PG.Genes	n	mean	sd	median	q1	q3	min	max
NXPE3	25	26749	9151	26293	21602	31692	4008	42535
MYLPF	25	98392	49926	82154	70357	120602	42319	232423
SYAP1	23	8195	4579	7150	5115	10025	1492	21243
EXOC4	25	22800	3901	22910	19252	25320	14665	29699
NTAN1	25	50599	21488	45139	38895	53231	21847	106095
FUBP1	20	14392	14874	9620	7898	14377	2035	63628
FUBP1	25	57957	44610	47553	27091	69183	10540	191452
TTC17	25	9766	4962	8938	7164	9884	4697	28848
LRRC59	25	50553	41491	38828	32822	50659	15135	213395
CLUAP1	25	26326	13566	20896	16236	32054	13414	63300
ESAM	25	12754	5519	12410	8971	14979	4602	32353
FKBP10	25	149793	50836	143445	123903	160196	79492	353335
AKT1S1	24	11307	6582	9767	6909	12926	3522	31958
ZNF428	24	10240	5379	8823	6859	13593	3906	26270
TMEM186	25	13608	5167	13956	10610	15533	3198	26961
TMIGD2	25	117732	74888	90243	59306	168779	21294	262412
AIDA	25	33371	10155	31972	26209	39132	16567	57669
ARL8A	25	9076	3765	8613	5960	11009	3322	16732
OTULIN	24	3233	1073	3000	2495	3724	1824	5867
CHCHD1	25	44595	43406	34519	18927	46467	9830	219538
PPWD1	25	14541	5189	13142	11665	17481	6727	33831
COA7	25	3339	1287	3191	2690	3915	1322	6594
PTER	25	9435	4804	9805	5350	11551	2212	21781
MOB3A	25	6835	4550	5909	4349	8147	2479	25741
LENG1	25	17191	12223	14180	9478	18948	3146	59448
FAM136A	25	46990	35085	38640	29290	52111	17635	201011

PG.Genes	n	mean	sd	median	q1	q3	min	max
MIEF2	25	9071	2714	9026	7151	10255	4323	16470
DHX58	25	23858	10297	21123	15889	32353	8383	42125
EFHD2	25	17216	12693	15741	10739	18856	4166	72727
GALM	25	28059	14184	24633	19659	34374	9064	66269
SYTL4	25	20027	7773	18425	15942	21771	11801	51208
ULK4	25	77445	20079	74451	64821	81490	51783	136250
DCPS	25	15082	5153	12829	11371	19185	6823	23551
PPP1R14B	24	14696	7492	14610	8867	18445	3186	31178
FBXL8	25	2900	2316	2619	1554	3218	470	9712
PPCDC	25	10054	5647	8939	6706	11601	3937	32976
ZNF653	25	5845	3422	5466	3578	7898	441	15419
NXNL1	25	93561	75513	67698	47005	97508	20390	291489
ISOC1	25	24911	12169	24087	14551	31253	8341	50078
GCC1	25	7417	1853	7417	5834	8280	5081	11467
FLYWCH2	25	8028	2803	7631	6066	9740	3546	13643
FAF2	24	9114	4365	7681	5852	12660	4284	18899
CCDC124	25	12748	12438	8967	5998	13097	2968	66159
AP2M1	25	13050	4619	12341	9750	16185	5193	21141
KCTD12	25	36380	23247	30132	17065	53208	7928	88105
RCN3	24	9351	4920	8595	5936	11546	4209	27700
COQ8B	25	92740	27350	89602	78434	103050	47519	167749
ZG16B	25	19744	8667	17660	15406	23084	7325	48526
RMDN1	25	8502	4544	7010	5862	10297	2038	19118
LRRC39	19	11115	7574	9659	4929	14535	2062	28176
CMBL	25	20120	7718	21862	13187	25841	4237	35837
SNRNP40	24	3635	1496	3944	2397	4849	1206	6353

PG.Genes	n	mean	sd	median	q1	q3	min	max
RMC1	25	120661	60243	102236	75150	138328	42548	271183
ARHGEF26	25	44828	12391	47697	40003	51176	22045	69633
ATG4C	18	5761	2189	5752	4937	6270	1802	9447
ITPKC	25	15483	6045	16157	12106	19387	4740	30922
IQCD	25	12411	3021	11593	10299	15415	6862	17706
RBMXL1	25	9189	8254	7000	5153	8531	2328	39678
SIRT1	25	88493	40068	82822	66879	94272	20942	234427
HOOK2	25	29549	9092	27259	23352	33068	13365	56098
SEH1L	25	4440	2626	3886	2961	4759	1077	12323
TCEAL4	25	14867	11359	11834	10628	15435	3644	60564
GNPNAT1	24	23719	16316	18569	12725	34182	2885	59470
INKA1	25	9367	8660	6166	5011	9253	3038	44036
MRPL53	16	3689	1642	3430	2516	3852	2178	8553
L3HYPDH	25	36772	23726	29398	18615	48072	2496	93294
MOCOS	25	65477	39672	51525	29101	85904	14601	148886
DAZAP1	25	26280	18963	24229	11179	33007	4140	81705
SAAL1	25	40461	19738	34786	29985	45609	15919	97696
CCDC51	25	6248	2388	5862	4692	7350	3346	13651
RBM33	25	33593	5335	32936	30241	36446	25424	44164
MMAB	24	4433	4078	2898	1760	4936	613	16574
ADAT3	25	22981	12047	20808	15814	26438	6590	65630
SAT2	25	10484	5625	8946	7550	12205	3283	25769
CCDC97	25	161949	169437	106791	75339	148315	35091	731165
DISP1	25	11510	4310	11708	8621	12593	4559	21376
CNRIP1	25	14049	10277	9835	6999	15829	3766	38528
PHYHIPL	25	16423	9895	16309	8191	23516	3644	46414

PG.Genes	n	mean	sd	median	q1	q3	min	max
DYNLL2	25	6147	4866	4745	3459	6273	1664	22984
DTD2	25	11135	12789	6971	5732	9377	3201	67394
S100A16	25	18302	15163	14172	7386	20643	2302	60788
SIPA1	25	30400	6552	29547	26043	34718	20044	44966
LRRC46	24	8040	4675	6882	5462	9482	3593	26832
THOC1	25	155989	31363	156123	140911	174969	72060	214266
OTUB1	25	26904	9989	23489	19953	36269	7918	42761
TRMT61A	25	8023	4301	7034	5093	11376	2183	16803
HMCES	25	11691	4275	10368	8633	14276	5420	23124
PGM2	25	102802	35238	110401	80331	129217	33860	163025
KLHDC7B	25	204147	114546	177737	162914	205756	88206	650923
DUS3L	25	9018	5123	8217	6288	10370	3199	29833
SDSL	25	30474	18352	26926	21055	36407	6575	95908
PDXP	25	8813	2924	7527	7123	10301	5438	14473
DCUN1D1	25	15038	5192	14731	11934	16832	7272	27936
FAHD2A	23	4351	1203	4163	3557	5098	2613	7968
APIP	25	37144	21323	29155	20274	52457	13144	88687
ZC2HC1A	25	20829	6027	20461	17268	24899	11988	32818
SNF8	25	21287	5665	20181	17106	27113	12306	30948
ZC3HAV1L	24	3008	1973	2851	1684	3927	290	8311
PDLIM5	24	12004	8039	10068	6543	15491	1658	32064
PDLIM5	25	44797	25058	39132	25460	61980	10055	112886
PDLIM5	24	11392	10147	8199	3200	19018	657	37448
ACY3	25	56971	30578	50872	34192	62523	23531	133455
ERO1A	25	15584	13882	11708	5500	19666	2578	58251
PRR11	25	15424	10537	13113	8720	21515	1061	40503

PG.Genes	n	mean	sd	median	q1	q3	min	max
FMC1	25	20954	8217	19512	15087	23282	9139	46158
OXNAD1	25	41481	14334	40780	29691	45451	22694	78262
DIRAS2	25	13088	6369	10871	8869	15323	4477	32552
INTS4	25	15452	4548	15218	12197	16823	8955	30114
DDRGK1	20	6173	3689	4881	3787	7169	2178	17988
SCLY	25	18070	4569	18050	15327	20755	8871	28918
FUBP3	25	19508	6863	17038	14702	22028	10038	36558
RBM17	25	30445	13790	28460	21953	35814	14626	84161
NARS2	25	81224	24002	78384	66323	99180	44877	129754
GMPPA	25	16074	9045	17556	11622	19871	3543	46781
ABHD14B	25	68233	33954	55761	41243	84593	22173	151046
NGLY1	25	46559	34281	55553	9821	70174	6203	124581
FAXDC2	25	33548	15748	31168	22188	39048	7346	73447
CPB2	25	6338	5293	4441	3170	7498	1625	25691
PAWR	24	21643	10764	20368	13191	28275	4641	48765
THOC3	25	56121	29346	48220	36841	68257	25034	156339
COG3	25	291628	92511	286758	229439	333280	160667	585917
HIC2	25	15921	9523	12687	8979	20909	4790	43681
CDK5RAP3	25	3438	1599	3237	2167	3804	1362	7877
ELMO2	22	5404	4957	3413	1804	7157	472	17072
ZNF333	25	45974	12656	45700	38124	54105	23827	84251
CHAMP1	25	16780	5485	16504	12241	19322	10466	35386
MYO15B	25	30796	14056	27887	24023	33548	11449	81953
CLMN	25	80793	18478	80611	74182	86662	50408	129057
PDLIM2	25	19256	4148	18050	16655	21403	11404	28708
BTF3L4	25	14607	8173	12231	10729	15606	6548	44257

PG.Genes	n	mean	sd	median	q1	q3	min	max
BTF3L4	25	28054	20554	23058	18447	28602	5382	95649
DNAJC1	25	21063	43880	10637	8741	17819	2381	229256
CABS1	25	10033	3641	9976	7560	10811	4637	21449
BTBD6	25	29376	21054	21990	17590	35732	3704	88080
ZNF512B	25	53976	27976	46545	35496	70808	22440	146824
LRATD2	25	127440	25172	123243	106216	149042	94519	177133
CNDP1	25	71702	18491	67307	59751	85005	43192	108592
RPGRIP1	25	156977	45258	142919	128427	183266	104892	266938
EXOC2	25	37754	8330	35650	33574	42113	20200	58213
CNDP2	25	375665	269804	318206	187107	447541	76922	1086803
ZFR	25	42088	14926	36959	32808	46845	25645	92837
WDR90	25	33319	8102	31126	27894	36032	22630	51684
CAPZA3	25	99406	83447	82259	65659	100432	42576	484295
KCNH8	25	106117	15907	106207	96001	117964	80720	137357
CAPNS2	25	110656	32565	104877	92274	118419	64210	212605
FCRL2	25	16457	7344	14282	11245	19938	7180	36270
PRMT6	25	10223	5916	8154	6820	9908	2714	26388
TRIM47	25	14884	5551	14318	11486	16978	7121	32650
SENP8	25	11517	5947	9522	6995	13624	3892	25365
CXorf58	25	34210	13301	31283	24739	42084	17473	78438
C4orf45	25	32214	15107	31732	24144	37487	12133	93075
C12orf42	25	17038	17980	12629	10484	16584	5193	99934
PPP3R2	25	28343	11234	27904	22822	32013	6044	66231
TEX55	25	56025	33667	50598	42930	54894	35361	211814
TBATA	24	16320	14063	13556	10795	16481	4321	78594
NA	25	3216	1859	2861	2003	3694	1481	10024

PG.Genes	n	mean	sd	median	q1	q3	min	max
CCDC7	25	19501	3448	19544	17272	20765	13211	29257
DRC1	25	17005	6569	16386	12175	20496	4984	33725
MFSD14A	24	238846	405563	83104	33616	289668	5760	1831189
C5orf34	25	11051	3064	10429	9224	12212	5580	20937
COG8	25	56311	21137	50507	46840	56540	35437	138496
TTC14	25	3715	2145	3190	2166	4959	840	10879
PWWP2A	25	146256	54775	130672	111348	162086	88305	321315
RILP	25	5599	2091	5417	4303	6220	3089	13706
SCLT1	25	32419	23537	25894	21350	33209	14284	131024
WWC2-AS2	25	14887	15522	10546	7809	14470	2010	77153
SLC46A1	25	11321	9805	8278	6029	15331	2239	49607
CLIC6	24	9761	7847	5512	4923	12154	2199	26690
FOXN4	24	4549	2026	4483	3254	5760	387	8863
IPO9	25	10266	4784	8537	7423	12463	5223	25082
ARHGEF17	25	20775	3978	20598	18950	21760	13567	30549
MS4A10	25	11219581	5938734	10848904	7704033	13400214	2687038	33482446
RBM14	25	47804	15493	49852	36932	58442	21285	88986
ADCY10	25	428002	175710	385986	333107	446576	285941	1171537
GBP5	24	10471	2070	10806	9154	11980	6769	13853
GBP4	25	9626	4185	8515	6990	10929	5233	21094
FANCD2OS	25	6743	1474	6595	5941	7741	3851	10212
PSMG3-AS1	25	10449	6104	8238	6338	15054	3369	24885
LMTK3	25	61072	15783	56564	53381	65221	41254	118250
TRNT1	25	69280	16485	69928	57216	81850	38863	97156
FLACC1	25	9293	5068	8409	6116	10999	3232	23544
TMEM237	25	39840	31188	31646	24014	41426	8167	133615

PG.Genes	n	mean	sd	median	q1	q3	min	max
GSDMA	25	14424	5254	15144	11635	16974	5469	30671
PPP1R10	25	6483	2401	5797	4719	7173	4199	15417
PRAM1	25	4804	1748	4347	3607	5691	2197	9760
VPS35	25	44228	19852	47848	29401	58338	12057	76576
PURB	25	7810	5447	5533	4923	8816	2541	25150
PSKH2	24	3928	4256	2087	1391	4902	549	17991
PHF12	25	13572	5601	11551	9934	14871	7446	29521
RBP7	25	380717	177103	327830	254356	472604	140018	845741
PANX2	25	67436	49159	48775	39630	80634	24395	253446
NACC1	25	9279	2360	9566	8632	10369	3331	14332
ZNF300	25	47183	12117	43347	39501	52060	32273	84750
SNX18	25	21342	4962	21310	19025	24527	11532	32431
VPS13A	25	22198	4390	21038	19763	22633	15474	37109
MCCC1	25	6704	2142	6103	5301	7598	3526	12321
METTL26	25	8891	6605	7514	5924	9480	3272	38355
TP53RK	25	19832	4975	19447	16568	23236	12858	32240
HAPLN3	25	138004	57002	127356	98521	168161	71526	300001
PLEKHF1	25	33005	30083	21564	16827	35388	10675	151945
PPP1R9B	25	13162	4379	12106	10802	14339	7078	26455
SRPK1	24	6519	2308	5687	4943	7531	3297	11740
CPXM1	25	5899	2899	5274	3776	6533	2232	14263
CYP2S1	25	13184	5493	12266	9248	16112	4259	28518
IWS1	25	89863	14303	86853	81294	95694	62837	123856
SIN3A	25	2657581	799959	2417407	2145742	3018528	1517962	4865219
ADO	25	12143	3992	11432	9790	14351	3179	19796
RUFY1	25	114714	33326	114749	94050	129284	62640	201900

PG.Genes	n	mean	sd	median	q1	q3	min	max
PNKP	24	6653	2572	6232	4973	7524	3310	14048
NIBAN2	25	30086	14118	26937	19431	36492	12416	66155
RMDN3	25	6647	5870	5030	3736	6551	2694	30586
TCF12	25	5064	1355	4553	4252	5808	2894	8390
GAD1	25	10575	3522	10445	8099	12147	5340	20175
MYCBP	25	10572	6575	8754	7171	12376	2519	33569
TBCB	25	20427	9017	19856	15846	23492	5517	48491
PSMB7	25	11848	5641	10959	8296	14524	4787	28366
CNN2	25	20134	11068	16712	11068	27442	4485	48919
PCYT2	22	8164	5229	6366	4085	11291	1968	20362
PHOX2B	16	1763	773	1520	1183	2278	812	3400
CDC5L	25	38730	22571	33395	31003	39508	21568	142628
PSMD1	25	42430	18118	46293	28709	55078	13161	83319
PFDN5	25	47572	19029	44907	35789	54456	21969	110073
PARK7	25	293920	104435	282994	221438	338794	90071	556631
SORT1	25	5628	2224	5944	3925	7663	506	9271
VAT1	25	36047	11777	38958	28902	43996	12509	58005
NUP88	25	12631	5283	10882	8961	15303	7570	31055
PKP4	25	1249481	362768	1163945	1017507	1386689	721615	2352741
POP1	25	29218	9957	26411	24099	30390	20936	69966
S100A13	25	45248	30051	36813	27726	52649	7812	133421
SCAF11	25	62727	14479	57749	54061	68200	46095	111235
TSNAX	25	31274	7669	31079	26105	34516	20554	50579
SEPHS2	25	33336	13015	30650	25478	37326	15024	71679
TTC1	25	6541	2587	5497	4792	7951	3165	13978
DNAJC7	25	16995	25214	12035	9985	13489	7217	137093

PG.Genes	n	mean	sd	median	q1	q3	min	max
C12orf57	21	5111	2275	4795	3636	6519	2196	11160
COPS8	25	10451	3275	11427	8466	13117	3688	15974
CHP1	25	14722	7380	13356	9102	20574	5038	32484
MAP3K5	25	35614	8479	35573	29868	40464	22535	59224
MGLL	25	2959	1244	2894	2089	3705	1187	6182
KIR2DL4	25	6743	1968	6313	5264	7325	3955	12016
HSD17B10	25	15267	9833	13102	10916	19531	3243	41382
SEPTIN5	25	40509	29967	32224	25622	41361	19890	167121
CCL19	23	49798	88652	18910	11083	43896	2479	418348
NAP1L4	25	46106	15798	45895	32151	57609	15552	84095
NPAS1	25	6307	2059	5545	4956	7853	2046	11385
NAPG	25	51783	18572	43918	40657	63152	19688	98693
TXN2	25	42182	60704	24396	21035	32455	14432	316633
ABCA3	25	75944	31195	68306	60790	77171	50057	196979
MIPEP	25	14666	4970	13504	11609	14666	8381	27075
ACO2	25	92515	77692	62064	49316	114644	31012	406613
TM9SF2	25	216745	72852	204284	171024	236974	125379	400717
TSG101	25	11120	4779	9748	7139	14832	3711	20925
CPNE1	25	29334	15589	25818	17900	39780	8596	63521
CCT7	25	48020	23276	47319	32231	58763	14220	99860
SMO	25	17312	8947	14173	12253	19074	7319	48220
EBNA1BP2	25	18905	25967	11238	9264	17753	3735	137171
PKP2	25	16432	4665	17165	12887	19241	7321	28502
SH3GL1	25	58244	18117	55179	44247	64208	34309	100834
TEP1	25	22424	12213	20903	16557	22321	10166	75976
OMD	25	124275	40805	120637	95935	148513	64021	224641

PG.Genes	n	mean	sd	median	q1	q3	min	max
SEMA3C	25	12086	5349	10478	8447	15495	4384	26495
VRK1	25	41075	17966	34585	26308	57700	19208	85030
DPYSL5	25	19031	4532	18542	16278	21840	11167	31654
NIPSNAP1	25	27154	13366	24457	20469	30273	14518	85579
HSD17B14	25	6711	2328	6285	4847	8161	2958	11955
ARPC5L	25	15121	7987	15148	9405	15898	3802	44819
FAM118B	25	27376	9129	25372	20388	31480	15404	55860
KCTD14	25	36668	18540	30172	24248	44876	15813	87300
TRIR	25	3982	1535	4109	2535	4931	1769	6723
MACROD1	25	48421	13765	45523	40101	55038	27466	78762
KLHDC3	25	92330	39507	93519	64225	124717	8248	161577
WDR77	25	15306	6546	14654	10871	18834	4732	28951
KXD1	25	217206	152355	174274	117955	239594	45816	692390
ANTKMT	25	18095	7858	16686	12604	21083	7128	34699
TUBA1C	25	10496	5529	9574	7522	13953	1891	22651
APOL2	25	13090	7129	10974	8028	14672	5275	34524
PSD2	25	941807	331389	916591	714330	1052638	497966	2050303
NRIP2	24	35861	20085	31210	22497	49854	8365	87095
HEPH	25	128103	33269	129606	103101	148429	63371	194457
ACBD6	25	10454	5397	8726	7456	12141	4746	30987
CORO1B	25	35655	14680	32796	25716	45941	12694	73506
CCDC77	23	6986	2436	5774	5208	9029	2687	10925
NAA38	23	6723	8808	2764	1953	7700	1062	35720
TXNDC17	25	55523	24688	55142	44695	65914	12935	109553
PLCD4	25	28415	6132	28318	24811	30516	15752	45841
CPPED1	25	22745	11818	21104	15257	23608	5628	54524

PG.Genes	n	mean	sd	median	q1	q3	min	max
VPS25	25	6061	2401	5625	4093	8116	2152	10405
NUDT16L1	25	4798	1494	4735	3789	5601	2279	7941
ERP44	24	19351	24327	13109	9767	19946	4171	127372
LXN	25	42995	25615	36448	27721	47081	9427	123484
NTPCR	24	4735	3326	3851	2580	5553	1622	16038
HDHD3	25	12335	11854	10064	6533	11236	3535	53787
TUBGCP2	25	105714	32967	91436	86321	120661	60753	183358
ESYT1	25	58443	22674	53414	43876	69919	28469	127032
UBAC1	25	14251	3782	13639	12026	16092	8712	24833
CNPY3	25	110865	57499	94300	89110	121213	46670	345386
PSMG3	25	79839	79216	61283	54088	76018	32370	450427
COPS4	25	49056	11037	50392	41218	56807	30498	72513
WAC	25	194599	90744	153211	130148	251227	101133	427536
DIDO1	25	133179	37982	121594	113788	137975	96110	273506
RAMAC	25	119220	50409	114920	79356	154326	51926	225331
FUCA2	25	6381	2544	5706	4485	7793	2940	12482
HGH1	25	42457	15298	42813	29224	48873	23031	85096
THTPA	25	8882	3304	8097	7001	10578	2694	18432
DOHH	24	5406	2039	5187	3656	6962	1924	8986
SPINDOC	25	25138	7942	24661	17342	29151	13882	41164
TUBB6	25	8290	2465	8099	7048	9002	4353	16265
PAXX	25	24849	14653	20895	13132	35808	4049	67222
HNRNPUL1	25	31938	10426	31673	25866	39895	14012	51122
PDCD10	25	10781	2815	11699	8819	12631	4408	15351
EFHD1	25	9248	3210	9620	7109	10950	1458	16543
DDX23	25	6820	4520	5579	5052	6783	1650	23376

PG.Genes	n	mean	sd	median	q1	q3	min	max
BDH2	25	30160	18468	28188	16788	38529	1855	82359
BDH2	25	35383	55575	22187	12995	29024	4806	283037
ALG12	20	10143	7414	8258	5680	11007	1629	30321
C1orf50	25	9731	3921	9909	7549	12275	2016	18797
MRI1	25	4434	1408	4457	3636	5438	2055	7572
THUMPD3	25	7469	5508	6623	4501	8074	1663	30698
ADI1	25	11478	6315	9834	8269	13003	3126	25630
RNF126	25	4143	2727	3776	2593	4450	1444	15237
KATNB1	25	18133	3447	17944	15995	20288	11461	27040
PBDC1	25	13396	5733	13522	8982	17663	3234	26861
DUSP23	24	7387	6792	5623	2933	8780	1341	28897
TMED9	25	20166	5528	19266	17124	21277	10319	37778
NUP58	25	9869	6314	6306	5771	12138	4165	25773
SELENOO	25	55952	23544	50452	39888	68744	14418	103578
DPCD	24	6083	2908	5587	3785	7023	1905	13292
GNL3	25	5511	1462	5417	4468	6507	2913	8419
TPPP3	25	82789	75018	45599	38552	96141	21212	300541
HIRIP3	25	19697	6133	19722	16067	21950	10388	40371
RBM4	25	199453	115645	173123	127392	225351	105826	687106
SF3B5	25	31802	32198	20950	19590	32888	4292	163136
PRR14	25	271838	70664	262002	233943	290293	196996	536481
SSBP3	25	12526	10240	8255	6562	15074	5393	53231
SCRT1	25	15809	13197	12067	9397	16187	4141	70620
APOL6	25	13349	11450	8188	4342	16952	2101	41698
SYCP2	25	84172	17626	83411	71856	90348	53897	124601
SORBS1	25	80321	22350	72699	66708	93691	48355	150715

PG.Genes	n	mean	sd	median	q1	q3	min	max
HINT2	25	14006	10605	12763	8760	15002	4996	59607
ZNF471	25	50871	18574	49148	39383	56420	16238	101721
PLVAP	25	25224	5735	25413	23379	26458	14992	47543
SPATA16	25	18365	6175	17298	14589	20302	6134	36636
RAB11FIP5	25	20663	6282	18500	16271	23484	11383	36063
C1QTNF5	25	37253	10659	35410	31934	44674	15622	60348
TMEM120A	23	368521	542322	154910	104720	387365	45185	2615042
NAA15	25	57186	24067	55130	38728	71712	22274	119410
CDCA4	25	28133	12160	26273	23720	31527	9034	63956
ASPN	23	5911	4027	5030	2668	6548	1952	16360
PAPPA2	25	117466	41823	115601	82356	137692	61535	241485
QTRT1	25	5743	2142	6164	4664	6778	1076	10012
CFHR5	25	167639	43211	163664	136158	188935	108006	265383
CACNG6	25	31513	10162	28176	25647	34644	20050	66773
TEX12	25	78149	78587	49709	25995	85057	4225	303030
GON7	24	4348	2597	3951	2801	5276	544	12121
OSBPL1A	25	12655	3776	11496	10403	13752	6280	25389
EMILIN2	25	49324	10896	47668	41801	52067	33853	76402
ANKRD30A	25	33387	7579	31178	29274	34431	26554	59620
PACSIN1	25	17907	4434	17403	15836	19897	7488	28420
GPR87	25	22010	11500	18842	12541	26035	9015	53474
ITPA	25	20307	11614	18309	12634	27143	5422	46760
EIF2A	25	35845	15409	32092	22356	46633	14800	66264
CADM1	25	142824	194832	85925	32912	132971	3016	902608
POLDIP3	25	15780	9850	12384	10602	16545	4402	46941
GNB1L	25	27800	14320	26587	16904	36440	9670	69949

PG.Genes	n	mean	sd	median	q1	q3	min	max
PCGF6	25	27124	7969	25498	20784	31633	13132	44143
YTHDF1	25	18216	9277	15054	13446	19871	9693	56093
STK33	25	38512	7381	37594	33499	40843	28271	63123
NLN	25	95623	31991	87679	74601	112380	51447	199202
CEP41	23	6052	3839	5709	3754	6642	994	19091
POTEKP	25	198739	66986	175634	149101	252767	98771	309260
NUF2	25	74236	21965	65660	60431	84499	48450	141715
GTPBP4	25	58383	9759	55403	50481	65226	45393	79866
ASPSCR1	25	129288	41321	125671	103098	158645	43834	216647
DPH1	25	175890	228256	110901	83810	171294	51138	1217662
IRX2	24	8312	6192	5845	4267	10534	1994	27387
UPF3B	25	19071	12001	14788	11415	19543	8083	51048
TBL1XR1	24	14097	13517	10461	8292	16212	3743	73168
UBL5	25	7685	2912	7074	5768	9373	3273	15658
GSX2	25	538936	457678	412641	299733	583434	32463	1852789
NIBAN1	25	22635	10584	20384	14951	23942	12210	49813
UBXN6	25	7263	2813	6793	5454	8032	2540	15416
TM6SF2	25	27296	12306	25389	19644	32619	8486	64841
DPY30	25	11609	6904	10364	7681	13864	1580	33666
TRIM2	25	159593	79307	133318	97606	196357	68215	390518
SPEF2	25	21130	5025	20557	17583	23786	13324	35488
FTO	25	12407	8155	10840	8926	14273	4993	48874
CFAP74	25	15928	3068	15394	13700	17686	10516	22836
TNKS1BP1	25	44612	20255	38569	36635	44453	23291	115453
UBE2O	25	13128	3966	13096	10236	14929	7906	24700
ZNF518B	25	7702	2392	7228	6160	8317	5021	14664

PG.Genes	n	mean	sd	median	q1	q3	min	max
XPO4	25	74942	30487	67540	57460	77890	44066	167655
SRCIN1	25	58481	12163	54882	50449	66733	40801	87672
WDR33	25	41325	24323	34013	26097	51002	18411	113659
WDR12	25	11767	4244	11128	8792	14138	4453	20909
C20orf27	25	24272	10051	21679	17101	26813	9086	50532
PDGFD	20	3099	1421	2804	2374	3506	462	6898
PITHD1	25	24408	6274	23851	20594	26713	12190	40746
COMMD5	25	2965	847	2999	2305	3426	1486	5461
DNAI2	25	15953	5171	15627	12365	19862	5690	26649
WDR61	25	6106	2945	5803	4092	7745	1378	14003
NIF3L1	25	15567	5518	13858	11333	20940	8958	27976
EGLN1	25	11848	3368	10930	9843	12906	6809	21997
NYX	25	126096	142078	88877	72105	107278	57390	765055
NAA50	25	11201	6004	9990	6201	13825	2538	25908
UBA5	25	7090	3186	6846	5772	7949	2573	19695
LHPP	25	22778	8683	22758	16734	26404	7022	38238
PAIP1	25	19539	10859	19718	11479	23816	4356	58039
NAT10	25	109283	41426	96920	88043	119488	54353	231001
COMMD4	24	9089	2823	8149	7641	10848	4053	15320
IQCN	25	56981	17680	49733	44121	69952	34078	100465
KLC2	25	27094	11781	24733	18470	33057	14602	65273
ILKAP	25	6729	2955	6263	4945	8044	2704	16265
XRN2	25	9653	3383	9259	7274	11820	5350	18786
TOLLIP	24	8840	6830	7043	5710	9197	2668	33765
INTS2	22	7267	4450	6588	3501	10233	1883	17315
KLHL25	25	187006	65686	165025	137559	216443	96147	344658

PG.Genes	n	mean	sd	median	q1	q3	min	max
TKTL2	25	8207	2463	7896	6645	8801	4744	15028
QRICH2	25	91289	19382	88602	75597	95556	62990	142144
CSTF2T	25	57227	25991	55079	41918	64391	28072	160245
PCBD2	25	13963	6321	12993	10277	14562	5702	31341
CYRIA	25	33419	26896	28034	21113	32628	16679	155079
HDHD2	25	10047	3462	9613	7626	11013	4869	20106
RAB1B	25	8268	5240	6793	4969	9605	3113	27432
MRPL18	25	30479	19759	24757	15529	36684	8123	87685
C11orf54	25	30215	16799	27499	22312	32583	3167	83770
FAM234A	25	9315	6231	7219	6454	8755	4020	28755
NAPB	25	117811	45210	101307	86360	159531	46167	229045
CDH19	25	8279	3922	7422	6231	9290	3360	23118
SIL1	25	7495	3356	7000	5244	7718	4093	19954
IRF2BPL	25	52006	19286	47337	39081	54540	30691	99083
UNC93B1	25	160490	97022	127845	107830	175682	88525	567262
CYSTM1	19	3944	3214	2669	2190	4365	648	13195
POLR3F	25	7379	3476	6825	5799	8220	1416	17467
NUCKS1	25	57626	54195	36956	29759	67776	13129	270716
TMX4	25	4610	1783	4844	3336	5629	869	8905
MEGF9	25	26465	12807	21648	18756	32338	13191	68243
ATG5	25	8089	2900	7652	5928	10270	2663	13886
WDR13	25	19763	6224	19448	16183	23005	8237	35785
TSPAN10	25	21834	17335	15696	12500	22596	7619	75266
EPN3	25	83709	25596	82324	73220	95434	29615	151105
OR10A2	25	127450	151670	99821	58170	122675	38518	817399
EHD4	25	21164	8917	18906	14134	26482	8064	42669

PG.Genes	n	mean	sd	median	q1	q3	min	max
GBA3	25	28703	7074	28933	22584	33822	15295	43021
MMP28	25	5889	2071	5308	4609	6712	3313	13134
SPTBN4	25	80949	10113	83222	74801	87511	55475	94475
OR51E2	25	1761443	1590170	1125516	770931	1789908	598813	6598283
SH3BGRL3	25	80946	26217	83009	59037	97831	33904	131345
SLC25A32	24	16193	14462	9996	8357	21157	4069	74638
HSD3B7	25	172142	67481	149765	125767	207144	86803	330804
TSPYL2	25	40067	14264	41874	30523	46196	18672	79601
BLZF1	25	100410	32127	95430	81566	112785	43181	161950
PPIL3	25	7214	2658	6487	5570	9068	3096	13577
SLC38A1	25	26568	7437	24914	21479	29683	13383	42270
PDCL3	25	43826	21867	41695	30855	48746	22145	129118
RAB3GAP2	25	130102	43919	126312	97382	147958	78820	237843
ADNP	25	10342	3164	9670	7717	11737	6809	18174
ZNF106	25	154281	46188	140280	129177	159564	103530	322141
PNN	25	18262	4732	17858	15998	19590	10699	33201
OR52D1	25	33061	31718	24116	16653	35406	9561	160804
GGNBP2	25	67715	32861	58469	46734	84038	34088	171424
CPVL	25	5875	3678	5061	3493	7093	1307	15404
BOLA2	25	22660	10069	20736	16368	22429	11861	55183
TMX1	25	44542	153587	12256	7937	21214	3661	780755
ACBD3	25	10842	6292	8850	6921	12206	4119	28745
PTPN23	25	33994	9505	31670	28457	36468	13070	58378
UNC45A	22	11336	24129	5284	3492	7221	194	116730
CHMP4B	25	12947	6719	12856	8466	16148	2878	26629
FN3K	25	20880	5447	19850	16311	25093	11896	29231

PG.Genes	n	mean	sd	median	q1	q3	min	max
POFUT1	25	49703	20510	49024	36831	53544	24692	129542
RNPEP	25	83113	32227	79498	59391	98596	26658	163234
GOLPH3L	25	5122	1885	5183	3580	6235	1566	9264
GOLPH3	25	16622	14573	12287	10939	14781	9041	75316
TUBB1	25	63378	14318	62267	52419	70722	32713	96500
SMOC1	25	3883	1222	4132	2967	4679	1574	6159
EPB41L1	25	18457	6698	16177	14630	21514	9565	38759
GLIPR2	25	7322	5471	5310	3738	8443	1527	21444
PLEKHA4	25	160545	129346	133179	108910	145066	71290	747986
EHD1	25	20273	9389	17715	13560	25704	8828	39926
PCIF1	25	38997	23970	33881	27713	39654	19570	133595
ESF1	25	123810	29248	115994	103370	136286	86218	201443
MROH8	25	15790	11568	12425	10685	16018	8124	67371
ELOVL6	25	52007	59543	28813	20625	60366	17459	296943
CIAO2A	25	16011	7407	14162	11407	18424	6318	37542
SLITRK6	25	51077	15475	50464	40528	61503	15369	86426
STN1	25	54135	20787	53239	40534	60956	24253	118656
OPA3	25	18365	9494	17579	9935	24211	6820	37375
ACSS3	25	32316	17802	26884	19452	37899	16795	90629
YTHDC2	25	93827	33622	87854	69611	113767	56693	192069
CWH43	24	4108	2320	3313	2798	4068	1780	11321
SPRING1	24	12891	7919	11923	6648	15308	1997	31876
DCTPP1	25	61285	28951	55796	37204	72631	26342	155053
SMYD3	25	31650	20294	29055	18803	37919	7763	92345
AAMDC	25	13950	4851	13394	10289	17443	5896	22523
ATP13A3	25	99490	24832	97361	82162	111138	66203	181814

PG.Genes	n	mean	sd	median	q1	q3	min	max
SCAF1	25	22029	6918	19726	17172	24237	14555	37457
DCLRE1B	25	11635	4358	11548	8778	13755	4787	22809
UBE2Z	24	15250	8628	12435	8322	20623	5860	39179
NOL11	25	2761	1390	2447	1970	3502	643	5873
METTL7A	25	45630	15397	43940	32275	52099	25878	96289
MMRN2	25	27733	23393	20182	16692	29783	10315	129524
AKTIP	25	55759	21448	56049	42561	71642	18552	96744
ANKZF1	25	53364	17917	52753	38247	57241	34121	98154
RNF121	23	13544	9689	11580	6102	18241	819	38536
SLC25A22	25	6781	1733	6736	5830	7784	2765	9967
MED20	24	6200	2404	5880	4519	7771	3260	13058
QTRT2	25	17026	7806	18833	12317	20466	2090	35591
ARMT1	25	13187	9466	10678	5752	17658	4488	44187
LRRC40	25	25879	8545	25078	21114	30281	14223	50595
AGO3	25	27267	9858	23170	20195	32077	15194	51134
CHODL	24	9986	7390	8539	6110	10892	926	33749
NHEJ1	25	10386	9383	6809	5319	8158	3578	35204
CAB39L	25	16909	5745	16389	12650	20751	6035	31037
FN3KRP	25	6664	2374	6371	5286	8002	2064	12268
CARS2	25	26538	9618	24713	20118	32445	11203	51340
PPCS	25	34596	28686	27461	20139	40135	10047	159635
MLXIP	25	10440	4550	8884	7833	11259	5641	25003
C17orf75	20	2794	1298	2661	2095	3518	693	6160
SIAE	21	12572	7824	11336	8242	16165	955	31349
PLEKHA5	25	49299	17671	47750	36846	57491	21026	97352
UPF2	25	95581	38215	80835	71513	107981	57003	211309

PG.Genes	n	mean	sd	median	q1	q3	min	max
RNPEPL1	25	74161	11843	73288	64343	81276	53404	95484
GRPEL1	25	11682	12741	7704	6264	9856	4469	60690
ELOVL3	25	23234	5663	24329	18783	26899	13251	39534
MYG1	25	14010	6301	14094	9707	15714	4951	35475
KCNK13	25	12139	4895	10797	8810	16419	5557	26624
SEBOX	25	78571	45558	62044	47071	100619	33730	200458
SCPEP1	25	14798	7059	15091	9051	20226	4184	26741
CYP3A43	25	82578	26189	90699	58182	101117	40472	117520
PIDD1	23	3595	1267	3353	2799	4507	1554	5961
PARVB	23	71743	39967	70950	47325	80499	8369	187354
TNS1	25	95679	50052	82508	62836	106458	47815	236884
NMRAL1	25	35546	19223	34639	23151	42525	13868	101807
SLC38A10	25	5533	1536	5336	4540	6921	2909	8492
ZNF287	25	63172	15578	61968	51212	71514	40591	109888
EML4	25	262338	111233	250002	164079	316283	97728	497554
GLOD4	25	143196	50480	145271	109269	160853	52298	261797
CBX8	25	5960	2157	5897	4009	7080	2838	11773
MUC5B	25	181692	198762	94703	67200	161344	17994	712626
NEK6	25	58940	23247	53107	45702	69354	31452	143962
SPON1	25	9417	10148	6905	4213	9770	2342	51628
MCCC2	25	74699	59665	56425	43329	77767	29946	265331
NCOA5	25	6639	3665	6149	4553	7864	1430	17580
MOV10	16	1734	926	1570	1307	2272	286	3592
ANKH	25	39940	23920	31607	22865	43195	16729	105357
VAT1L	25	56140	30334	45088	35164	65318	23691	150156
GPAM	25	84587	27197	83576	68414	95886	27356	160558

PG.Genes	n	mean	sd	median	q1	q3	min	max
RESF1	25	148789	28505	142625	129236	161989	102356	230816
PLXNA4	25	14647	2681	14130	13339	15563	11311	23172
PGAP6	25	17485	20908	11659	6974	17577	3752	102190
EPB41L4A	25	49352	21991	45637	35315	58318	9156	109529
XAB2	25	91078	16751	89785	76860	102926	62207	135934
CD248	25	3191	2169	2356	1830	3834	1172	10745
PREB	24	6749	2915	6306	4653	8438	3025	15518
S100A14	16	2477	2425	1422	1028	2800	415	8570
CHMP1A	25	8065	3097	8951	5452	9957	2819	17391
RETN	20	6021	6913	3446	2015	7033	466	24643
APMAP	25	23841	12858	25147	12162	31833	3635	50234
TXNRD2	25	126622	42539	118204	100071	138947	64099	272555
SINHCAF	25	21042	28710	11721	7365	25643	1195	146234
BPIFA1	23	61471	102344	16556	8391	50546	3661	417369
ARFGAP3	25	47852	23379	43616	32299	59324	18152	124074
PALMD	25	21036	5885	21234	16945	24176	12278	39366
SSU72	18	4873	2230	4410	3662	6446	1149	9085
ABCB9	19	3343	1069	3414	2562	3732	1756	5687
VTA1	25	13077	6992	13038	7372	17345	2842	33280
DYNLRB1	25	7680	4454	8043	4429	9137	1475	21445
PARD6A	25	15143	10255	13520	9889	16563	6106	58491
GPCPD1	25	180971	71735	166086	121006	229554	78978	327968
CCNB1IP1	25	91335	18282	89261	76884	102525	53279	123063
A4GALT	25	95676	53263	82161	57902	141164	16910	212491
MYNN	25	8792	5048	7096	5223	10864	2831	22712
OSGEP	25	11204	4446	10934	8166	13465	4282	19383

PG.Genes	n	mean	sd	median	q1	q3	min	max
ACP6	25	9016	11190	6029	3927	10510	1510	58402
ISYNA1	25	79073	46445	66085	48222	116706	10766	182178
OBP2B	25	37294	36668	28183	12152	41230	7548	162047
NXT2	25	6567	2453	6006	4892	8590	2647	11335
CD93	25	17001	7009	16426	13112	18704	6005	32830
LZTFL1	25	19420	8032	18543	13523	25781	5723	33928
MEPE	25	11968	4357	10449	9134	12984	5978	25140
TIGAR	25	8534	2963	8375	6725	9310	4839	16742
RTN4	25	29062	8416	28721	23530	33674	9958	43612
CYLD	25	13510	5773	13001	9365	15056	5945	27064
RPRD1B	25	15333	17096	11334	9199	14139	4949	93722
PFDN4	25	18987	5724	18659	15371	22910	8110	31306
NIT2	25	36356	15977	31650	27935	45889	11261	83398
AVEN	25	17555	3341	17067	15468	20178	10491	23034
EXOSC5	25	15200	4598	14528	12188	19061	9002	25359
FSCN3	25	46250	121513	18699	10340	25185	1420	621012
KIF13B	25	120284	24829	118487	105241	128592	82216	189567
PAK6	25	16793	18359	12920	10844	13296	8124	103163
ANLN	25	7140	1674	6880	6051	7709	4223	11560
XPNPEP1	25	29427	14446	27996	18925	41332	7641	55538
GPHN	25	25255	8967	23334	20016	28313	11856	51948
MYO5C	25	107172	24141	101271	95568	109608	80330	195148
ITM2C	24	17404	7140	14765	13346	20011	7673	36182
BIN3	25	27813	20011	20914	16150	28277	12363	102095
DDX21	25	10945	4247	10101	8126	12179	6107	23795
MAN1C1	25	85083	34025	86369	62659	104693	34456	153753

PG.Genes	n	mean	sd	median	q1	q3	min	max
NANS	25	7329833	3035125	7255368	5909828	9084382	2208451	14957533
SH3GLB2	25	9618	3593	9207	7072	10817	4836	21230
EIF2B3	25	10271	11849	7891	5911	9407	2670	65229
FBXO6	25	29575	14765	27921	18704	38523	13615	75383
PICK1	17	19663	11270	17660	13384	20546	9232	56506
CTPS2	25	14735	6757	13653	10708	19361	4271	33803
CHRAC1	25	5491	3667	4238	3596	5445	2779	20945
PAPOLB	25	50723	30368	43781	29440	63024	14315	120835
STRN4	25	17062	7174	14502	12832	20072	8261	36966
ENAM	25	76532	34577	65923	58884	81763	41435	219817
AASDHPPT	25	6969	3015	5983	5219	7874	3361	15658
UBQLN4	25	26868	17264	21768	19006	27932	9901	81581
HEBP1	25	15784	4735	15265	13275	17669	7922	29162
RAB6B	25	10489	2211	10779	9125	12233	5927	14720
APOBEC3C	24	8106	3133	7844	5678	10359	1809	13598
PHPT1	25	21315	9296	20675	14823	25562	1973	42967
ARHGAP35	25	45171	13790	42212	38711	47915	29498	97926
FAM114A2	25	11374	3313	10515	9327	13578	5833	18712
LTBP3	25	60149	27968	52404	45700	65376	25737	158190
RGS18	25	24605	13216	20567	14606	28901	6341	58189
LANCL2	25	18921	8347	16630	15881	19019	9584	52345
KCND1	25	20957	16820	15940	11221	23779	6863	89241
CTNNBIP1	21	7676	2985	6976	5728	8758	4102	16694
ST6GALNAC1	25	97087	21280	97684	87243	110672	52769	154740
FARSB	25	31386	13952	32239	21448	42960	6636	55273
IARS2	25	488337	155505	457381	371132	638794	268584	753940

PG.Genes	n	mean	sd	median	q1	q3	min	max
STARD5	25	6862	1882	6527	5504	7349	3911	11423
ATG3	25	6889	3474	7071	3794	8395	2356	16118
PDS5B	25	54722	8839	52778	49809	60969	40828	72772
OLA1	25	22003	9469	19967	16657	27175	7876	41994
CUTC	25	7178	2273	6328	5494	8447	4523	11823
RBM12	25	73365	35342	67322	42289	90299	26032	152544
ANKEF1	25	19792	6716	18241	16878	19333	13550	43895
ZCCHC3	25	16913	4432	16075	14956	18730	10881	31166
ABHD10	25	6408	4107	4828	4051	6672	1741	17907
STAU2	25	2256235	967724	1969766	1540218	2529482	1199149	4876902
TMEM106B	15	1899	1103	1822	1338	1989	366	5177
LIN7C	25	11340	7111	9441	7324	13403	3275	32682
CYRIB	25	1857459	1214547	1755315	1208419	2035032	690125	6713092
DDX19A	25	19189	5844	19088	14104	23928	8077	32250
GIMAP4	24	10845	6477	10326	6841	11940	4150	36531
TDP1	25	34638	28329	23757	21227	32776	16085	123818
TBC1D23	25	3124	1042	3063	2539	3862	1255	4943
MRGBP	25	11273	8831	8588	7357	11883	4332	44959
SEPTIN11	24	3501	2230	2850	1909	4848	793	9929
SLC38A7	25	23195	10311	20959	15119	30591	6639	51788
MED17	25	75155	38007	72410	48842	88510	27351	202035
PARVA	25	11738	7726	9513	5260	17750	1102	27214
PANK4	25	5663	3677	4605	4071	5485	2621	19085
FBXO28	25	13552	3704	13322	10503	16132	8607	21713
TBC1D13	25	12308	3824	12224	9391	13689	4907	20538
ARL8B	25	58846	63762	52028	28668	61215	11384	346877

PG.Genes	n	mean	sd	median	q1	q3	min	max
DNAJC17	25	11078	3745	10964	8369	13218	3749	20622
DDX18	25	7304	2744	6403	5144	8639	3370	14789
ASF1B	20	6724	3829	5799	3893	8451	2146	15632
TBCCD1	25	11521	2989	10767	10113	12919	5545	18515
ARMC1	25	15278	10063	13244	11171	16954	5128	53992
MTPAP	25	7114	1564	7034	5833	7793	4094	10254
RLIM	25	51850	13482	52206	41187	61093	29832	80147
RBM22	25	3854	3877	3090	2561	3625	1618	21786
WDR70	25	21790	8322	18458	16875	22436	13827	51984
SLTM	25	89850	26077	86340	78729	91499	63901	202884
PAG1	25	12773	5868	10761	8912	16800	5200	28484
PARPBP	25	29686	6671	29938	23920	34260	17215	40594
PAK1IP1	25	8540	4203	7660	5969	10525	2120	23806
HIF1AN	24	5867	6020	4189	3651	5546	2414	32403
CZIB	25	53265	18625	51796	42391	61384	22292	91504
BABAM1	25	11776	5534	9136	8169	12876	6486	27206
CLN6	24	26394	13469	24921	18550	32867	6732	63812
C2orf42	25	48795	16718	44082	38296	52427	32010	101665
THG1L	25	6279	1319	5984	5347	6950	3515	10172
IRAK4	25	71082	17414	69072	56826	83531	40162	97900
COMMD8	20	3200	941	3040	2603	3843	1652	5063
NHP2	25	8944	7305	5912	4539	7450	2804	26059
OCIAD1	25	6743	4554	5542	4388	6614	2378	20681
ADPRS	25	10518	2793	11020	9047	12310	5290	16779
HYPK	24	6522	3174	5927	4444	8211	1928	16121
CHCHD3	25	25670	7573	26342	18977	30641	12606	42669

PG.Genes	n	mean	sd	median	q1	q3	min	max
ZSCAN32	25	171632	53025	156660	136767	180449	120910	358473
ERVK13-1	25	16269	8809	14947	9353	17968	3528	43915
SIRT5	24	10413	4496	10822	6628	12075	4777	23316
THUMPD1	25	24264	15000	21605	15177	28216	7051	78721
FBXL12	17	6085	4515	4351	3022	8879	1528	18840
GIN1	25	37212	14406	34864	26876	43938	16015	69989
NDE1	25	27786	9357	28970	21967	31697	5936	49738
BABAM2	24	5413	1729	5629	4091	6411	2055	8970
CDKN2AIP	25	23993	12747	19832	16880	24904	11338	58060
GAR1	25	5284	2584	4649	4048	5883	2160	13998
STAB1	25	14563	2251	14593	12765	16069	10456	19196
PPP4R2	25	11261	2619	10890	9751	12229	7734	17488
SLC5A4	24	6881	11351	3736	2618	5717	1422	57718
TERF2IP	25	11836	5207	10407	8773	13066	3258	26480
BCLAF1	25	11691	4778	10014	9023	12607	6584	25730
COA4	22	8908	10815	5131	4060	7571	856	52040
TLR7	25	121130	28274	122166	103292	132460	74179	173629
MAP3K20	25	25021	15321	19897	14222	27709	11819	75580
TMOD3	25	12823	9721	11124	7949	14228	3167	49431
CELSR1	25	31280	8613	28791	26152	33375	21778	55049
FAT2	25	136808	25340	125232	118468	152660	103777	199757
NKIRAS1	18	2573	1193	2545	1881	2841	542	5303
UGGT2	25	134656	30750	130264	110848	142572	93241	228077
UGGT1	25	97489	89446	78376	56948	100305	31715	494167
RBAK	25	296648	166455	233380	191636	363378	130248	831586
ERAP1	25	62313	29366	57066	37327	75583	24220	146878

PG.Genes	n	mean	sd	median	q1	q3	min	max
ACTR10	25	6831	2245	6699	5555	8244	2473	11132
C9orf78	24	9474	10350	7132	5230	9166	707	52458
FAM120A	25	18146	5248	17242	14911	21222	9540	29971
SMARCAL1	25	79408	27599	79406	62032	86442	38670	142374
GLTP	25	21049	12012	20190	13151	27125	3748	47260
AHSP	25	13617	13331	7570	3892	17494	2237	43514
GPRC5B	15	7411	5391	7109	3081	10410	1244	19030
NUDT4	23	4281	1396	4247	3473	5064	1077	7487
EHD3	25	14267	6557	13314	9797	19314	4841	29269
EHD2	25	51528	56599	24112	18108	60502	5101	187038
CNOT2	25	24283	15052	21458	14481	34209	2728	59201
C1RL	24	5158	3802	4041	3214	5584	1853	20389
TMOD2	25	21985	8478	20147	16849	24019	11174	45946
CALML5	25	12529	5105	11817	8976	13871	6393	27964
OGFR	25	21994	7025	20698	16933	24583	13865	39260
LMCD1	25	170401	96579	144929	93156	228584	38691	366784
COMMD9	25	27084	10838	25434	19567	29224	13508	65023
THYN1	24	12444	4503	11894	9124	15118	4466	21310
NDUFAF4	15	9634	3853	9282	6993	9778	5448	20217
HACD3	19	4975	11185	1827	1323	2696	716	49509
PDP1	25	16399	7894	12735	11378	16885	9257	39152
VAPA	25	53687	41461	35976	25080	56274	20139	156393
MACROH2A2	25	15230	13284	12008	8053	16003	3953	67408
RNF181	25	7824	4083	6850	5691	7752	3731	21446
MTRES1	20	4937	14127	2010	920	2304	210	64751
GSKIP	20	4011	2117	3216	2623	5240	1545	9490

PG.Genes	n	mean	sd	median	q1	q3	min	max
ZNF581	25	3729	2548	3004	2709	3657	582	12756
SPATA7	25	14884	7056	12984	10492	16567	5338	31679
ABRACL	25	22926	11671	23851	14977	28278	3878	52299
MDFIC	25	14195	3045	13823	12624	15344	8603	22260
ACTR3B	25	56554	16387	53910	48813	59175	32267	112362
HCN3	25	25239	5092	23482	21072	29052	18478	34039
WHRN	25	7526	4915	6293	4581	8583	3086	26902
PLCE1	25	33336	6855	30939	28146	37281	25308	48373
VPS18	25	5598	1881	5769	4221	6549	3080	10517
RCC2	25	56104	27299	61809	29602	71972	12539	113285
SLAIN2	25	21230	5437	20436	17828	22394	11905	38560
USP36	25	139030	43355	130738	112938	142682	92565	308366
FNIP2	19	2350	1025	2188	1573	3086	1130	4400
BAHCC1	25	58179	11768	56383	49612	60098	43700	88645
STK26	25	224422	62688	214879	184512	262239	136691	433167
PTGFRN	25	10379	1949	10525	9213	11746	5820	14452
RRBP1	25	29516	13217	28349	23245	31421	9852	82713
MAP10	25	67165	40220	59536	44649	67671	34965	233561
KLHL8	25	19249	5742	17945	14558	22174	9130	35501
CEP126	25	20656	6675	19243	15512	23399	13247	37966
CPSF2	25	16707	3766	16312	14484	17576	10993	28235
LARS1	25	74034	50214	60249	34874	104653	13558	194455
CC2D2A	25	26082	10210	23692	19628	30848	11295	54815
RBM27	25	11024	2241	11112	9778	11974	7578	18149
SUCLA2	25	60437	29710	56599	44864	68014	23259	176798
WRAP73	25	10229	2476	9786	8256	11354	6787	16592

PG.Genes	n	mean	sd	median	q1	q3	min	max
STX18	25	18602	7617	17765	14956	19006	12614	51618
DELEC1	25	33728	37464	24039	18963	29818	11544	206106
ATXN10	20	8371	7892	6279	3322	11410	798	36628
TFIP11	25	41513	10998	39440	33408	47197	28079	65293
MYO1A	25	48603	23406	43564	34140	53590	23362	127792
SPRR3	23	12138	20333	2452	1432	12061	410	77550
HSFX1	17	8820	5507	8774	4185	11899	881	19115
SAE1	25	15185	5387	15259	11928	17376	6586	32227
NLK	24	4588	3063	3772	2945	5221	1142	15900
COPG2	25	119016	45499	110601	76964	154323	59811	225528
MRC2	25	15681	4844	15801	12338	17460	8396	25660
IL36RN	25	20398	8770	18891	14755	24314	7870	46957
COMMD3	25	5003	1495	4684	4253	5891	2478	7961
GNG12	25	7891	4461	8214	4705	10633	1475	17785
HCST	25	18966860	14065296	15317523	10053916	25965428	4259258	65198428
CPNE7	25	27540	23597	23633	18022	27620	12361	134397
DKK3	25	151510	74453	136069	117688	191544	34048	387462
VPS29	25	7230	2918	6778	5060	9150	1845	13796
GRHPR	25	35602	14156	33482	25843	44736	10431	63938
CTSZ	25	53115	28255	51608	34044	65770	15969	135358
UBA2	25	16016	6146	15870	12469	19944	6514	32308
DKK4	22	50772	31083	42411	34664	52476	21549	165851
NXF1	25	52902	67218	40800	34112	46994	23300	373314
PEF1	25	6814	4199	5817	4133	7636	2630	22908
BIN2	25	49463	12677	47239	41557	53868	26379	84274
COPS7A	25	8609	2100	8434	6987	9879	3744	13423

PG.Genes	n	mean	sd	median	q1	q3	min	max
FBLN5	25	44742	28651	39845	25654	54198	12018	147264
APEX2	25	666294	341945	558850	475535	729250	233694	1966542
PPP1R1B	25	25062	27846	14566	9971	28720	4920	135876
AASS	25	13670	3636	13196	11196	16097	8093	20905
ZNF212	25	23953	6295	22401	20084	26334	16602	45345
CFDP1	25	8857	2456	8826	7045	10135	5313	14070
VTI1B	25	39119	8891	39484	33668	43245	24043	59075
STK39	25	10528	2426	10198	8764	11580	6332	16737
LRWD1	25	3030213	1648546	2241722	1858404	3748604	1420048	7071786
NIPSNAP3A	24	14240	20300	9079	5343	14153	2533	102763
CGGBP1	25	35722	18113	33653	24256	42043	4097	90350
ABCF2	25	8628	3076	7811	6670	10484	2898	17198
TES	25	17465	8460	13721	11182	22751	6363	34643
DMBT1	25	14800	21340	8361	5974	12583	1304	98434
FETUB	25	6389	3058	5497	5008	5897	3008	16581
LIMD1	25	32274	10801	28595	25724	38871	17520	56051
HMGXB4	25	9054	3387	8856	6192	12128	3688	16753
APOBEC3B	25	6315	2590	6486	3959	7325	2241	11776
SWAP70	25	84415	26701	77579	64058	91181	54373	156698
FBXO40	25	21875	6198	20229	17485	23900	14050	39135
LAMTOR3	24	12870	10009	9971	8540	11816	6161	47410
LIMA1	25	19878	7146	19140	15385	23147	10302	43571
SRP68	25	20292	6772	18770	14841	25608	11695	36650
CNTNAP2	25	154143	65472	134190	112642	191069	73980	392059
NPC1L1	25	68585	30861	59973	46345	88618	31205	161481
CHORDC1	25	17030	7993	17195	10345	21701	5656	30772

PG.Genes	n	mean	sd	median	q1	q3	min	max
UBQLN2	24	4895	2596	4285	3158	6006	1863	13325
EGFL7	25	12000	11734	9394	5822	13825	921	55837
IL20RA	25	36455	22037	28170	20837	45465	10432	93641
ADAMTS1	25	8852	4358	8250	5680	10563	3092	20909
SHPK	25	4537	2113	4316	3416	5374	1527	10435
DPP7	25	22678	12686	19107	12019	30641	6351	49435
SAP30BP	25	46420	22068	41971	31268	50984	15401	117081
ZNHIT2	25	2597515	1433941	2358350	1853549	2767377	876242	7478062
PFDN2	25	115833	37058	119576	88647	129764	60566	204076
PUF60	25	17108	10483	13706	9895	21721	5842	50671
NRBP1	25	5512	2840	5212	3858	6368	1860	16277
ENOPH1	25	19764	5543	20038	17275	22361	7678	37865
ATP6V1H	25	16696	8380	16456	9389	21821	4030	32137
TAGLN3	25	55719	23399	51068	44056	62429	23547	150250
TRMT112	21	6282	3186	5225	3996	8631	1946	12038
CPA4	18	29645	26760	22036	13908	34377	3546	107052
XPO7	25	9478	4121	8902	6376	11236	2771	19149
BAZ1B	25	104313	19899	101258	91499	120500	75750	154539
ATP5IF1	25	21893	19326	15321	12178	22936	1051	79834
AK3	25	12893	6481	12184	7224	15468	5071	31879
CNOT7	24	8207	2628	8482	6375	9663	3131	13545
SERPINB13	25	39090	26117	31273	23311	43122	13081	114169
GGT7	25	14599	4235	14449	11149	17160	6927	23016
RABGEF1	25	35280	9333	33905	27511	41674	20529	52159
MAGEL2	25	56932	45280	50627	33678	59902	13461	235207
NAGK	25	39284	17092	34526	31852	46541	14142	97913

PG.Genes	n	mean	sd	median	q1	q3	min	max
CRLS1	25	13083	12229	7319	4680	17589	2799	48323
SH3BGRL2	24	9426	7395	7005	5265	10255	773	25941
RASAL2	25	45105	12042	43355	36069	49546	30373	74928
DBNL	18	3746	2350	3925	1716	5534	531	8004
DCTN4	25	4435	949	4343	3851	4893	2526	6494
ZNF229	25	82866	33985	77757	62941	87995	41436	196211
CDC23	25	29163	11010	27027	22456	33991	14716	68855
ANAPC2	25	61943	22758	55378	49630	70150	37527	147548
VPS28	25	14272	6502	12021	10478	17262	5062	33577
LSM7	23	24424	14863	22037	15849	27372	6972	76942
SERPINA10	25	5980	1312	6172	5541	6826	3073	8438
DBR1	25	17031	4178	17005	14212	19142	10209	25811
TASOR	25	87260	17988	84370	73787	98245	53638	143873
FEM1B	25	13582	5370	12678	10486	16118	5168	26470
JPT1	25	16811	10880	13647	9486	20524	3179	47305
FBXO3	25	10089	20461	5931	4832	7667	2671	107807
AKAP11	25	58515	25708	49276	44571	58389	34864	147674
DNAJC12	25	347927	144919	306859	225463	507600	159784	629921
CPSF3	25	196362	68323	189569	148068	214236	84121	406790
PITPNC1	25	28044	32251	20494	16953	26484	11362	179674
APPL1	25	30501	12686	24945	21005	40217	13222	57885
PARP4	25	39211	7343	37613	34306	43764	30693	60380
NUDT5	25	18234	9253	17733	11651	22746	4239	39414
RCOR1	25	14187	4876	12663	10383	17397	7097	24920
MAN1B1	25	49814	26591	44553	32936	56049	18444	140743
MYO15A	25	163209	73564	132788	123103	176513	101336	380584

PG.Genes	n	mean	sd	median	q1	q3	min	max
GTF3C4	25	23620	9346	20608	17838	25494	12422	49085
ADAMTS6	25	32905	11043	31238	23690	39666	15491	61731
PACSIN3	25	55088	25830	49975	40677	60114	24782	148790
FBXO4	22	6291	3485	5841	3966	7915	1923	16108
FBXL21P	25	7065	4781	6101	3901	8569	3093	26625
ACAD8	25	32824	16161	28528	24264	35058	12361	81158
ACIN1	25	377052	144688	339009	299682	384671	249594	821651
AGO2	25	17053	4294	15912	14501	19783	9191	26887
MYH2	25	77805	12099	75141	69461	80918	62554	107308
MYH13	25	63740	14933	61007	55788	67295	44070	123923
NUP50	25	27544	10746	25020	20496	29886	13509	56251
ZHX1	25	50880	19781	43405	35446	59970	27330	105451
DSE	25	144450	65562	129395	105247	136360	80654	323076
AGO1	25	10617	4919	9226	7542	13200	4776	26972
RAB21	25	12728	6080	13135	8898	15088	1241	29646
RAB22A	25	5911	3273	5434	4244	6480	2642	20085
PSME2	25	104013	49835	90895	71575	130630	30957	217974
RAB23	25	27796	12900	24703	21098	34177	4965	58599
MCTS1	25	91519	28621	93058	65824	112504	52961	154930
MTUS1	25	177351	40838	173525	158842	190303	106000	280443
PALD1	25	125567	52741	112101	99077	134699	68534	342705
SLC39A10	25	127043	91512	86057	70989	154432	27165	422604
ZBTB21	25	63934	25175	63283	45482	75373	18497	121559
PLEKHG1	25	103890	19881	100046	90459	118672	74154	140363
CNOT6	25	107602	24763	104420	92446	121365	68283	170371
TBC1D24	25	7477	2456	6756	5850	8599	4664	13939

PG.Genes	n	mean	sd	median	q1	q3	min	max
PPM1H	25	48244	26060	42623	26605	74441	14783	107884
KCNS2	25	9860	5243	9127	7072	11242	2964	23983
ZNRF3	25	32093	9870	31662	28561	38978	11823	49479
HECTD1	25	10185	3541	9676	8043	10386	6510	20855
MYO5B	25	64050	10479	59007	57004	67965	51313	95073
FZD4	23	11284	5658	10192	8267	13550	316	26742
CORO1C	25	2134227	1271426	1811032	1273398	2798104	612344	4939795
NAP1L2	25	17603	14765	12844	10165	18084	6483	79572
PYCARD	25	70337	35769	62801	45340	87347	22868	166920
PADI4	25	8715	9707	6689	4914	8167	2720	53452
EPDR1	24	12199	17058	6851	4444	11480	1211	84387
MYO6	25	43524	10258	43252	36098	49936	25661	71808
PPT2	25	405819	320817	294023	181108	506590	89992	1477614
NFU1	25	44472	29730	35084	27977	49691	19682	164889
PRPF19	25	123067	57834	132780	75518	154252	26405	233031
SYNPO2	25	16544	3940	16505	13975	18060	10035	29516
NENF	25	13013	14305	7946	6477	10554	3541	59402
VPS4A	25	10196	3814	9089	7217	12623	5992	19082
ARHGAP26	25	90207	26677	74574	69188	110684	52836	139422
STUB1	25	54035	19537	48583	42053	63055	20975	109695
CDC14A	25	17702	5669	17014	14134	20025	7498	35352
SNX6	25	16376	5418	16101	12070	20008	8605	30773
DUSP12	25	12200	4343	11326	9815	13287	6530	25097
PSMD13	25	24601	11672	24999	15385	32972	5729	51077
FAF1	25	6761	2824	6028	5426	7185	1949	14306
PROCR	25	9709	3232	8923	7152	10925	6069	18184

PG.Genes	n	mean	sd	median	q1	q3	min	max
DIMT1	22	3120	2232	2564	1902	3061	1117	10993
TIMELESS	25	17199	3947	16335	13503	19601	11817	24925
WDR3	25	59735	30069	50523	42334	69627	20006	173674
NSFL1C	25	31522	14105	26768	23686	35765	16473	82426
ADAMTS8	25	21795	10614	20537	14627	26318	5116	49694
COG5	25	28390	7259	26944	24421	32997	14154	46239
MACF1	25	104720	37668	97269	86863	107347	71170	262714
SCAF8	25	30320	7915	29140	26448	30489	18366	60764
TRIM35	25	11661	10371	8020	6075	11694	4085	48792
ZC3H4	25	57845	10312	57702	50050	63277	40808	79733
SORCS3	25	88116	40686	73652	60179	102746	47864	207712
USP24	25	119200	44213	102905	92477	121015	77920	264502
SAMD4A	25	39876	16300	36273	30857	45013	18413	96040
PHF24	25	43397	15409	41845	33550	50348	17519	80125
TRAK1	25	78841	13789	78242	68383	88174	54224	108395
SHANK2	25	24560	9934	25735	17179	29956	6378	53391
SRRM2	25	147912	64443	128531	114858	144597	91372	316925
CNTN6	25	1542688	771265	1238889	1102666	1816524	767147	3767896
PA2G4	25	75139	22706	75121	56412	88265	32597	116197
SPG7	25	9747	5304	8111	6055	12584	3012	20552
CLCA2	25	50196	31918	36799	32991	59391	16274	158374
ERVW-1	25	15726	23375	8735	7481	14862	5584	124642
MAPK8IP1	25	91709	26933	90798	77982	101099	30550	143698
ZNF148	25	23869	6506	23776	19256	29027	12741	35563
RTRAF	25	20178	14884	16468	12619	21504	4450	77649
RUVBL2	25	12346	8336	11866	6497	14683	2764	40730

PG.Genes	n	mean	sd	median	q1	q3	min	max
LIPT1	25	31164	13569	29267	23496	35573	13388	66442
NOD1	25	10039	2092	9266	8446	11972	6610	13425
AKT3	25	32536	13920	33553	24434	39780	11944	78761
EIF3L	25	16454	9222	17342	9969	22653	3017	38869
PLAA	25	20673	6087	21160	14767	25050	12050	33979
RUVBL1	25	30711	14819	30591	18414	37866	9448	60304
NUDC	25	39065	18380	35874	23582	53626	8707	66877
ST3GAL6	25	32826	29964	21831	17927	34795	8001	134391
HS3ST2	24	9485	5886	9766	4584	12198	1937	26500
CFL2	25	1784856	692125	1633500	1357284	2042208	722960	3474131
FARSA	25	8853	2097	8832	7696	10016	5596	14470
ASF1A	21	4891	3264	3854	2891	6214	570	13208
DRG1	25	10608	4784	10526	7117	13771	3172	21445
NCKAP1	25	87618	45884	78773	68752	92064	55841	298929
CNPY2	25	71235	75138	48660	35161	89778	21700	405327
DTX4	25	17298	4638	15853	14033	21154	11398	28876
STK38L	25	10651	2895	9684	8706	11105	8250	19963
INPP5F	25	423138	87279	424699	367084	475372	268227	690769
PLEKHA6	25	24045	8376	22262	18164	28028	11539	45945
WDR37	25	4568	2127	4159	3162	6210	1437	9461
PADI2	25	9827	8199	6277	5448	12416	1876	41211
ZKSCAN5	25	39980	12483	41084	33712	47431	16234	75861
SLC27A6	25	35484	18989	30995	27670	39960	19316	120517
LAMTOR2	24	6611	5846	5876	3511	6721	1698	26180
MRPS7	25	64689	15456	63047	54232	74310	35967	93655
TMA7	25	34031	27419	28436	22552	34932	5328	126981

PG.Genes	n	mean	sd	median	q1	q3	min	max
AP3M1	25	6453	3255	5938	3391	8527	2000	12324
CARHSP1	25	32921	16264	29389	26147	34577	15035	95406
THRAP3	25	29407	13127	27258	22937	31515	11562	75904
WBP11	25	9852	3396	10197	6948	12180	3789	15861
NOP58	25	116561	33572	109594	95062	122395	66561	230566
DERA	25	14853	4210	13486	11677	18449	9380	24217
LSM2	25	24859	14035	26026	15914	29246	3163	79244
STARD10	25	9097	5715	7838	4744	13355	3083	27038
CAB39	25	127769	39919	124256	99743	162164	39694	188347
LUC7L2	25	12975	3782	12599	10903	15217	4787	21098
MRPS2	25	23926	8411	23033	19503	27699	8762	40756
SBDS	25	12147	5467	10711	8601	15261	2266	23173
EXOSC1	23	6420	7909	4612	3814	6160	2058	41786
SF3B6	24	8029	4692	6752	5456	9496	578	19433
REXO2	24	12980	6533	11841	9599	14998	4087	34847
RRP15	25	20630	19109	15741	11826	22975	4225	104901
WASHC3	18	4389	1737	4399	3221	4766	2126	8541
PPIL1	25	12462	5199	11696	9171	16096	3596	23802
UFC1	25	32030	14841	30794	21920	37648	10064	74039
FIS1	25	23291	10088	22881	14245	30035	7012	41960
AK6	19	51950	52137	34428	24419	52657	8893	229790
HDGFL3	25	12019	8319	10174	7856	12282	3767	41577
BOLA1	20	2640	1650	2302	1507	3146	643	7813
CHMP3	23	8047	3738	8035	5164	10030	1875	16157
CHMP3	25	6389	1944	6500	4922	7498	2892	10111
STRAP	25	10963	5674	11177	6210	13520	3412	29493

PG.Genes	n	mean	sd	median	q1	q3	min	max
RTCB	25	29853	10051	31291	24148	35643	11092	53725
RABGAP1	25	54442	10635	54013	47636	57903	35562	75455
TSC22D4	25	11683	4037	10632	9152	13119	7324	23393
RPL36	20	6750	6449	5259	3353	7979	240	28541
SAMHD1	25	46766	36769	30906	20164	68629	14324	161352
HBS1L	25	19023	3300	19202	16431	20692	12648	25760
SALL2	25	5682	2030	4921	4250	6635	2701	9974
PRKAB1	24	4871	1508	4901	4360	5479	1939	8807
TLN1	25	373268	173072	336053	261950	484752	110112	749328
MFHAS1	25	55103	17134	53227	45207	59734	35392	120942
ZNF451	25	82145	17967	78917	74522	89210	45148	131316
USP15	21	27725	20764	21767	14237	35734	2455	85831
TOGARAM1	25	18791	4273	18534	15865	20769	10529	32551
RIPOR2	25	54572	8735	56051	49982	60624	36601	68812
TLN2	25	82085	14427	82675	68842	92360	53655	111144
IRS2	25	144069	68094	116039	104157	173054	50483	321607
LOXL2	25	86802	33895	78085	68529	95268	48512	198249
CRYBG1	25	68438	15836	66940	55803	78318	46364	105527
MAP4K5	25	1379137	794313	1092542	896228	1576176	593727	3947095
HYOU1	25	20654	14086	15305	11736	25297	8183	67934
TBL2	25	11620	5733	9441	8089	12662	5851	32149
TELO2	25	84516	49211	67185	58314	92565	33115	269067
ARIH1	25	8794	3981	8311	7108	10388	939	23478
LSM4	25	22447	8660	24087	16372	28739	7378	42492
OARD1	24	5682	2451	5716	3882	7135	2052	13513
HSPB11	25	10476	4832	9688	6994	13230	2251	20557

PG.Genes	n	mean	sd	median	q1	q3	min	max
SUPT16H	25	91569	24106	90851	75469	108722	50770	144520
PCDHB2	25	36053	17899	31438	22787	40106	7870	80522
PCDHB14	25	13384	6152	13416	7824	17495	4796	27856
TIMM9	25	11994	10808	7085	4825	13997	3096	46742
TIMM8B	25	18165	4379	19049	14895	19837	10723	26895
PCYT1B	24	10896	22298	4687	3507	6534	2576	111458
CD2AP	25	18636	10441	14869	12105	23259	5489	48187
ATP6V1D	25	12544	19067	8216	7433	9182	5406	103129
TIMM13	25	11534	4642	9912	8433	13502	5484	25264
PPP2R3B	25	14236	5403	13177	10635	16129	7812	32702
MMP24	25	12501	9238	9716	8110	12470	5931	50320
DMRT2	25	32366	17090	27966	24139	31813	17638	102882
TRAPPC1	24	4339	1885	4239	2825	5156	1927	9877
CDC42BPB	25	189791	70351	167645	140620	246605	102603	350268
RBM8A	25	23918	14974	22681	16763	25535	7219	85874
ZNF706	25	26144	16123	28197	13771	32763	3077	64121
WIF1	25	5976	5561	4760	3446	5747	1846	28749
SNX13	25	42788	6573	43088	38245	46581	31149	54544
SNX9	24	8614	2900	8955	6433	10010	4055	16954
SNX5	25	13351	4420	13079	10822	15201	7013	25580
NUBP2	24	6133	2162	6080	4108	7862	2984	9775
ST14	25	64733	38685	51999	37701	76089	22712	175274
LYVE1	25	35391	24376	25048	20938	47843	4114	104884
HEBP2	25	19307	14571	15976	9905	23791	4533	76789
HEBP2	25	63907	50470	50246	34913	69635	17269	239273
PUS1	25	72566	64025	54211	47451	64919	16785	333595

PG.Genes	n	mean	sd	median	q1	q3	min	max
LRRFIP2	25	54598	15846	50362	44834	60946	36756	104860
FHOD1	25	276549	72529	261757	228707	295344	188311	438416
PSAT1	25	18034	16157	10488	8362	29004	2732	77729
F11R	18	17503	30311	5721	4193	13123	1215	103820
CPQ	25	54036	30576	50171	31929	68840	19187	158708
SPIN1	25	8962	12695	6727	5728	7539	3222	69361
COPG1	25	17357	8860	16881	11123	23417	5072	37816
CLIC4	25	142457	80511	131550	68502	193491	37694	338878
CFAP20	24	5263	1417	5148	4243	6220	2382	8528
SAR1B	24	7273	3233	6519	5740	8709	3138	18856
EMILIN1	25	25529	16719	19089	15633	28365	9504	81901
ARFGEF2	25	22467	9443	20164	17164	24814	14706	62921
ARFGEF1	25	60685	12496	58254	54116	71014	42972	82678
STK24	25	99291	41645	93033	75110	123846	35231	202117
BZW2	24	7606	5108	6752	3958	10270	1314	23190
COMMD10	25	174955	108687	131090	114532	173701	91492	578120
DYNC1LI1	25	11086	2472	10601	9489	12100	6621	16198
CHCHD2	24	8847	5336	6318	4800	13402	1446	17801
TEX264	25	407346	202351	368127	284450	485665	151386	1204033
DNMT3A	25	33007	13328	29009	23823	41493	17592	77935
OAS3	25	10235	7278	8576	6135	11099	4226	36389
AK5	25	280930	79885	285399	213022	328678	133448	468439
SQOR	25	10156	6301	9260	7578	10134	3924	37456
LAMC3	25	8929	1728	8645	8189	9782	5600	13292
CAPN6	25	8452	4796	8581	4404	10852	2151	19736
FCGBP	25	166645	212619	73874	39795	196983	19151	802496

PG.Genes	n	mean	sd	median	q1	q3	min	max
CAPN7	25	34001	9593	31549	28116	35427	22276	58481
WASF2	25	13974	3571	13652	10728	16165	8311	22682
FAM169A	25	11489	4606	10267	8615	12806	5272	23727
IVNS1ABP	25	13142	3430	12550	11337	13783	9263	26567
SEC23IP	25	52462	21061	50497	35958	65974	23572	118209
