# Supplementary material for: Data-Independent Acquisition (DIA)-Based Proteomics for the Identification of Biomarkers in Tissue Washings of Endometrial Cancer
Source: Int J Mol Sci. 2025 Nov 27;26(23):11498. doi: 10.3390/ijms262311498 (PMC12691889; doi:10.3390/ijms262311498)
Supplement: Supplementary file 1 [file ijms-26-11498-s001.zip › Supplementary Tables S3,S4,S5.pdf]

Supplementary Table S3. Proteins identified for having abundance values completely separated between EC cases and Controls.

Gene Symbol	EC	CTRLs	EC	CTRLs
	Median (Min – Max)	Median (Min – Max)	Mean (SD)	Mean (SD)
PDE2A	457337 (268190 – 1845667)	13536 (4796 – 83589)	478168 (232109)	18591 18901)
MAP2K7	157506 (66698 – 253808)	9423 (2313 – 27347)	149511 (49905)	9922 (5162)
**KIF3C**	**19654 (8251 – 58751)**	**890800 (474840 – 4782870)**	**22687 (10700)**	**1301452 (1018975)**
ARPC2	77215 (38796 – 183577)	13727 (3782 – 36999)	87952 (30822)	15500 (8947)
ZZEF1	197288 (83300 – 545310)	24176 (9630 – 65399)	208832 (98460)	25335 (10782)
EPB41L2	105871 (33483 – 454257)	4480 (3100 – 12175)	134473 (85752)	5386 (2153)
**PPL**	**185540 (108411 – 441262)**	**1012047 (527561 – 1.22e+07)**	**207149 (72131)**	**1526609 (2243822)**
EXOC3	108251 (22233 – 346982)	4628 (2064 – 21539)	111461 (71842)	5497 (3778)
PGK1	1117655 (309932 – 1950067)	62647 (9408 – 156987)	1111074 (363203)	69225 (40359)
GAPDH	1365258 (740269 – 2622601)	140366 (13778 – 373741)	1461974 (498254)	162814 (91993)
CAPNS1	55204 (22741 – 121297)	7885 (1392 – 13949)	56950 (21266)	7601 (3800)
GPI	193791 (46325 – 372204)	14552 (4893 – 41250)	191042 (66227)	16806 (10204)
TACSTD2	22403 (14342 – 40399)	5963 (2239 – 14175)	24190 (6389)	5970 (2750)
HSPA8	463309 (142066 – 879827)	38827 (4891 – 96427)	457437 (194108)	41996 (23030)
SPTB	164989 (101932 – 404586)	12034 (6693 – 83591)	190115 (70070)	15913 (15169)
PSMB1	65311 (24341 – 138635)	8730 (2671 – 23292)	68921 (26459)	9250 (5063)
COL5A3	184797 (21191 – 576371)	2776 (1382 – 6640)	139019 (124872)	2980 (1133)
ARID4A	38709 (22078 – 76824)	4216 (2263 – 8409)	40759 (12590)	4721 (1569)
HIVEP2	225366 (158842 – 405834)	21434 (13055 – 100676)	242939 (54064)	24432 (16834)
FBN1	389990 (265303 – 682135)	12151 (6490 – 107355)	403051 (81596)	17560 (19951)
PRRC2A	110319 (48792 – 355575)	20192 (9137 – 41354)	158848 (98388)	21545 (8485)
LRBA	311927 (119885 – 731353)	7761 (5016 – 34752)	362273 (180529)	9518 (6055)
RAB13	231333 (147156 – 485535)	11937 (5698 – 66880)	246733 (73142)	14136 (11617)
HSPA2	357804 (132668 – 803267)	27085 (10363 – 61019)	379501 (133149)	30033 (15709)
EEF1A1	4847758 (1484991 – 9373522)	95711 (6288 – 383717)	4690818 (1938483)	141216 (116936)
COG7	104850 (49637 – 385403)	7453 (3672 – 32803)	135050 (74470)	9704 (6751)
PRPF4B	113662 (72583 – 195723)	20402 (3939 – 72294	115676 (20435)	22418 (13859)
PDS5A	80568 (43585 – 259201)	21750 (10857 – 34899)	87354 (35074)	21631 (5657)
SYDE2	91059 (31232 – 175565)	7562 (1225 – 18275)	87261 (41327)	8332 (6023)
SCYL2	55567 (31699 – 98777)	9796 (5259 – 21252)	60610 (17032)	10117 (2808)
SUPT6H	147753 (101165 – 399549)	30071 (14825 – 67260)	164627 (56982)	32873 (12278)
PARS2	104327 (50520 – 300799)	10887 (3315 – 41320)	115443 (46268)	12268 (8605)
CYFIP1	43500 (16773 – 116021)	8613 (3433 – 16220)	55268 (30883)	9064 (3500)
NBEA	214453 (143834 – 298252)	32422 (12673 – 101036	215989 (33140)	37271 (19313)
RUFY1	142624 (62640 – 201900)	11540 (4872 – 55307)	137575 (35989)	13565 (9304)
PARK7	263845 (90071 – 556631)	21144 (3982 – 72031	276254 (101066)	24344 (15933)
COPS4	33290 (9639 – 72513)	1992 (806 – 7521)	33890 (17925)	2497 (1617)
ASPSCR1	158645 (43834 – 342802)	11908 (8265 – 36571	161581 (58534)	13749 (5773)
MYO15A	139885 (101336 – 380584)	12064 (6466 – 45964)	159804 (55896)	14135 (8471)
MYH13	73223 (44070 – 133739)	7920 (4014 – 38209)	75812 (19236)	9404 (6692)
SORCS3	73652 (47864 – 207712)	2905 (1313 – 28955)	83118 (31202)	4522 (5457)
MAP2K7	157506 (66698 – 253808)	9423 (2313 – 27347)	149511 (49905)	9922 (5162)

In bold characters, the proteins with lower abundances in EC Cases if compared to Controls

Supplementary Table S4. Proteins identified for having abundance values completely separated between High-grade EC cases and Low-grade EC cases.

Gene Symbol	High-grade EC	Low-grade EC	High-grade EC	Low-grade EC
	Median (Min - Max)	Median (Min - Max)	Mean (SD)	Mean (SD)
UHRF1BP1L	106236 (69227 - 288735)	29224 (20631 - 53184)	123511 (46057)	31753 (7862)
FBLL1	67980 (34275 - 194887)	6433 (3803 - 17548)	85845 (41556)	7760 (3413)
**KIF28P**	**18126 (12826 - 26996)**	**64417 (49928 - 123199)**	**18346 (3427)**	**67841 (15169)**
PROB1	45326 (21635 - 81842)	6801 (4254 - 14134)	47820 (15720)	7141 (1936)
STXBP3	111658 (61002 - 249711)	11430 (7316 - 24967)	115222 (40212)	12174 (3428)
EXOC5	45192 (28273 - 118056)	6204 (3536 - 21151)	56607 (24721)	6866 (3307)
USP9Y	53312 (34456 - 68447)	17120 (12454 - 30948)	53323 (7589)	18344 (5054)
LAD1	184152 (66846 - 559891)	16689 (10662 - 44798)	227664 (115770)	19279 (7477)
PIK3C2B	368991 (226223 - 703596)	50614 (32532 - 109409)	408820 (129581)	54433 (19337)
DYNC1LI2	54403 (29158 - 67830)	9055 (3595 - 15324)	52983 (9013)	9369 (3505)
SMARCA5	39414 (30719 - 66878)	16122 (10969 - 26427)	45184 (11930)	17527 (4014)
CUBN	20998 (16508 - 32033)	10032 (7085 - 14890)	21717 (3890)	9999 (1676)
SORBS3	308865 (152219 - 2352666)	56659 (21536 - 91242)	509183 (484679)	58364 (16423)
JAK2	83405 (49920 - 150951)	8702 (6470 - 10526)	88243 (22769)	8485 (1288)
ROCK2	110547 (62811 - 169078)	10567 (6359 - 17796)	113306 (25753)	10859 (2263)
CLASP2	250903 (199448 - 570856)	58436 (29718 - 108656)	282798 (82302)	61448 (19015)
FLNB	599824 (232106 - 1229552)	8021 (1082 - 50844)	604872 (266349)	10476 (10568)
NCOR1	519508 (417067 - 862176)	76126 (48654 - 276126)	540845 (91527)	87006 (43102)
**SF3B1**	**28596 (14927 - 72446)**	**402172 (264511 - 684316)**	**32233 (13038)**	**416631 (113528)**
KBTBD11	40973 (21300 - 113470)	5213 (3065 - 18453)	52135 (26047)	6409 (3377)
ATP10B	82835 (48230 - 141556)	17419 (12626 - 28058)	86714 (22241)	17872 (3566)
SASH1	280537 (203994 - 434866)	43750 (28499 - 80793)	289108 (72056)	45210 (12125)
COL3A1	92443 (75799 - 124956)	21681 (16600 - 60422)	95583 (14755)	24126 (8937)
COL4A1	96641 (79413 - 160473)	16439 (11853 - 36921)	102165 (21054)	18784 (6172)
POLR3D	224466 (171341 - 373440)	7370 (2276 - 11749)	242741 (60258)	7470 (2967)
COL5A2	174191 (130702 - 253019)	27588 (16655 - 79338)	182329 (30946)	31687 (13064)
GSN	244724 (101663 - 594082)	14398 (7754 - 45439)	249369 (116961)	17439 (9829)
RB1	18685 (14637 - 31464)	8115 (5418 - 14181)	20701 (4200)	8529 (2265)
ENO1	3529175 (1583154 - 5585988)	762439 (139729 - 1266001)	3478417 (1124775)	746821 (310163)
COL4A2	42404 (33950 - 56356)	9040 (5740 - 17603)	43529 (5721)	9654 (2364)
PARP1	350871 (163390 - 912346)	53148 (21188 - 104375)	401542 (225227)	55851 (21566)
SCG2	226735 (146685 - 361892)	31107 (15465 - 126528)	241844 (56354)	40352 (23360)
**HCLS1**	**259309 (147767 - 514773)**	**3484686 (1604714 - 8905937)**	**285195 (99836)**	**3496286 (1538529)**
PTPN1	44931 (34110 - 94514)	11542 (7319 - 31886)	49884 (14864)	12713 (5382)
PAX7	188165 (87979 - 344515)	59508 (37784 - 83059)	200671 (73633)	60204 (11493)
DPP4	462178 (290598 - 1210648)	22465 (7508 - 52424)	551313 (259881)	23630 (10144)
CAD	38352 (23267 - 49003)	8561 (6114 - 15422)	36594 (7621)	9009 (2037)
ITPKB	176048 (120235 - 256854)	42613 (26505 - 73666)	177872 (34182)	42811 (9658)
**PTPRM**	**27574 (21372 - 47056)**	**1768661 (1365065 - 3045492)**	**28890 (5991)**	**1970734 (538137)**
GTF2E1	44749 (28066 - 60544)	2234 (1781 - 5158)	45132 (8875)	2553 (763)
AKT1	67403 (34554 - 157536)	17311 (9765 - 25869)	76872 (27079)	17912 (4802)
GRK3	509439 (285195 - 839837)	56688 (20743 - 95828)	514487 (156960)	56707 (19951)
RPS19	104642 (75948 - 293622)	22108 (6485 - 64851)	123454 (50677)	24076 (14268)
CUX1	13925 (9534 - 21368)	3812 (2571 - 6313)	14863 (3429)	4070 (1019)
HTT	229304 (181593 - 452608)	37856 (30178 - 105952)	245667 (56802)	45303 (18209)
XDH	1016465 (801805 - 1667302)	24067 (11258 - 135016)	1108781 (243314)	28980 (23091)
PRRC2A	236649 (180035 - 355575)	66871 (48792 - 110319)	252044 (47467)	69380 (13329)
**NUMB**	**14523 (10647 - 25751)**	**343117 (146198 - 1147452)**	**15376 (3887)**	**397639 (222198)**

SMARCA2	44394 (30160 - 57200)	5759 (4020 - 10819)	44296 (6470)	6266 (1860)
GUCY2F	69773 (54611 - 134896)	18984 (13683 - 25011)	78014 (21594)	18876 (3091)
EPHB1	180446 (80827 - 318008)	24071 (17420 - 37431)	190964 (55965)	24895 (5024)
AFDN	5305009 (2851436 - 10592363)	66433 (39894 - 220985)	5604725 (1835478)	79913 (45272)
EPPK1	152469 (121100 - 244158)	76195 (59187 - 98768)	160164 (30301)	76979 (10827)
ACTA2	1591653 (943757 - 3298919)	153071 (60160 - 395185)	1689377 (553014)	180633 (95076)
MUC5AC	47985 (30575 - 109251)	13083 (8061 - 25139)	54226 (22895)	14065 (3866)
TCHH	160362 (123288 - 331736)	34464 (12969 - 65303)	175647 (48756)	35361 (13443)
CGNL1	475360 (383669 - 760627)	103255 (68224 - 166407)	499905 (102884)	103656 (21597)
ARHGEF5	48132 (34231 - 132445)	14032 (10218 - 28711)	53060 (19950)	16330 (4888)
**ABR**	**13883 (8454 - 19326)**	**52369 (33705 - 80840)**	**14548 (2904)**	**54075 (11630)**
DDX10	878452 (179970 - 1427013)	29780 (17349 - 76876)	883135 (261377)	33770 (14323)
EIF3A	140900 (62569 - 303366)	15733 (5131 - 35937)	159616 (68792)	16415 (7336)
CRMP1	58807 (36336 - 105632)	17478 (7347 - 36238)	63766 (19318)	18477 (7671)
PTK2B	89906 (58595 - 182275)	25909 (15614 - 50257)	92763 (27709)	27382 (6960)
KIAA0100	143572 (115924 - 183594)	26902 (17586 - 55013)	146256 (17859)	27782 (6963)
MDC1	2106224 (1535804 - 2958355)	15657 (10728 - 25424)	2139932 (381036)	16231 (3785)
WTAP	30311 (23308 - 47844)	11504 (4523 - 20048)	33171 (6646)	11742 (3586)
ACAP1	19470 (10607 - 33560)	6484 (2909 - 9861)	21022 (5917)	6204 (1816)
HERC1	959083 (688848 - 1283812)	120585 (91629 - 186459)	970109 (123159)	124056 (20533)
LONRF1	228310 (192742 - 360149)	29974 (19377 - 60594)	242379 (45386)	33067 (11188)
TSR1	55324 (29420 - 135958)	14642 (9250 - 28350)	61855 (25269)	15607 (4790)
ZFP69	158082 (108967 - 239299)	45386 (21919 - 68253)	161365 (35276)	45195 (12822)
PLCH1	216262 (134560 - 321002)	7309 (4489 - 13521)	221709 (44037)	7996 (2457)
HSP90AA4P	36443 (27939 - 54222)	18989 (9623 - 26274)	38251 (6814)	18589 (4310)
FREM1	121137 (99062 - 148356)	25495 (15187 - 46600)	120563 (14827)	26014 (6446)
SPECC1	127155 (79933 - 226687)	10935 (5669 - 33623)	137235 (37567)	13131 (6209)
NEXMIF	97544 (70716 - 153924)	17495 (10630 - 42048)	105725 (23315)	21257 (8390)
UBR4	227918 (139812 - 339056)	24912 (15940 - 34068)	229007 (45363)	26104 (4476)
**KIAA1217**	**53637 (38451 - 84899)**	**1564667 (1139229 - 4280356)**	**57654 (12661)**	**1800468 (775048)**
VPS13D	518236 (454481 - 817267)	69910 (55406 - 97045)	556728 (99869)	73180 (10701)
**SYDE2**	**48434 (31232 - 77354)**	**115536 (91059 - 175565)**	**49349 (10613)**	**123656 (22153)**
ZNF318	120758 (91389 - 170108)	29531 (23135 - 60465)	123181 (19702)	31001 (7858)
TNS3	159691 (105823 - 283690)	14246 (9412 - 28283)	167972 (39672)	15286 (4258)
NAA16	215887 (140692 - 319640)	45686 (26042 - 73058)	212543 (39788)	48285 (12276)
LARP1	56040 (40352 - 101420)	16026 (9857 - 37603)	61435 (16866)	18336 (6458)
SPAG17	160333 (125451 - 247042)	45048 (18584 - 109922)	168456 (33364)	51895 (22040)
CSPG4	3947071 (2823651 - 5488224)	41130 (31744 - 66000)	3958736 (622620)	42295 (7122)
GBP6	374441 (199723 - 702954)	7275 (5495 - 14789)	397977 (120832)	7944 (2442)
UBN2	122100 (70163 - 171111)	33710 (22231 - 52765)	123854 (26827)	34644 (6676)
TRMT10C	469805 (308760 - 854909)	12314 (7620 - 23290)	498960 (132745)	12842 (3906)
KCTD9	186470 (57682 - 515891)	15293 (7276 - 45160)	205518 (103515)	18005 (8571)
GVINP1	111047 (75932 - 164771)	23793 (14738 - 40111)	114434 (20382)	24845 (5484)
CCDC186	118266 (81996 - 219946)	27933 (18104 - 75215)	129527 (40342)	34297 (16257)
**PKD1L3**	**37936 (25757 - 89497)**	**288238 (160087 - 416406)**	**41132 (12891)**	**291367 (60607)**
POGLUT3	1554529 (894743 - 4953718)	27488 (13274 - 55488)	1712163 (816431)	29176 (11817)
BCL9L	280452 (181176 - 363398)	6383 (4237 - 11214)	279136 (53971)	6475 (1594)
ANKRD13B	305591 (121648 - 490368)	14104 (7253 - 34909)	309949 (91209)	14256 (5814)
CUL9	3021259 (2094879 - 3755502)	260621 (137640 - 732402)	2953277 (403363)	273125 (112056)
SUGP1	425189 (281350 - 641907)	178296 (104205 - 252976)	439720 (94174)	174690 (41821)
**HACE1**	**30982 (18843 - 68439)**	**443214 (271113 - 1393121)**	**30850 (9191)**	**488977 (233255)**

SPART	69699 (51055 - 117170)	23705 (15244 - 45416)	75327 (15942)	26612 (7867)
GHDC	226837 (96579 - 646444)	8383 (2914 - 49547)	239357 (128753)	11813 (9563)
AFAP1L2	104349 (58739 - 194118)	14371 (7081 - 21747)	107642 (28756)	14639 (3338)
ANKRD31	64381 (47072 - 95433)	8491 (5186 - 16642)	67570 (11850)	9097 (2831)
RTL9	91009 (77791 - 132178)	18424 (13606 - 36205)	94434 (13012)	19032 (4729)
SDR9C7	20897 (12435 - 33812)	5845 (3008 - 7795)	21431 (4586)	5564 (1282)
ADAMTS17	167397 (87749 - 300712)	24318 (13217 - 54891)	175850 (53106)	25049 (9552)
PXDN	67247 (40046 - 130334)	12620 (7835 - 39914)	74028 (22026)	14291 (6276)
HDAC2	86573 (53617 - 155754)	12440 (5720 - 25841)	98715 (33251)	13867 (5987)
GCC1	55862 (39958 - 70131)	7417 (5081 - 11467)	54996 (7933)	7417 (1853)
DISP1	43891 (32905 - 76880)	11708 (4559 - 21376)	48263 (11823)	11510 (4310)
KCNH8	3213510 (2073656 - 6299471)	106207 (80720 - 137357)	3434986 (938663)	106117 (15907)
CCDC7	101052 (84273 - 145187)	19544 (13211 - 29257)	108356 (16593)	19501 (3448)
TTC14	72187 (42181 - 139300)	3190 (840 - 10879)	78717 (26435)	3715 (2145)
ARHGEF17	96371 (74019 - 189258)	20598 (13567 - 30549)	109859 (34707)	20775 (3978)
**LMTK3**	**17690 (11899 - 24997)**	**56564 (41254 - 118250)**	**17686 (2994)**	**61072 (15783)**
FLACC1	74354 (42461 - 193601)	8409 (3232 - 23544)	91203 (43412)	9293 (5068)
SNX18	245338 (135016 - 578723)	21310 (11532 - 32431)	269134 (114621)	21342 (4962)
VPS13A	65109 (49033 - 150987)	21038 (15474 - 37109)	74345 (24303)	22198 (4390)
NUP88	3972965 (2386985 - 9792115)	10882 (7570 - 31055)	4409337 (1730803)	12631 (5283)
**ABCA3**	**9470 (7373 - 16594)**	**68306 (50057 - 196979)**	**9794 (1858)**	**75944 (31195)**
PKP2	60417 (45198 - 130413)	17165 (7321 - 28502)	65302 (19079)	16432 (4665)
**HEPH**	**31022 (22270 - 46409)**	**129606 (63371 - 194457)**	**31828 (6093)**	**128103 (33269)**
SYCP2	291047 (221836 - 389373)	83411 (53897 - 124601)	294434 (43514)	84172 (17626)
SPEF2	47998 (36110 - 71052)	20557 (13324 - 35488)	50051 (9376)	21130 (5025)
CFAP74	75668 (49700 - 119874)	15394 (10516 - 22836)	76809 (14429)	15928 (3068)
**XPO4**	**21490 (15735 - 38563)**	**67540 (44066 - 167655)**	**23816 (6518)**	**74942 (30487)**
SRCIN1	248883 (211155 - 371563)	54882 (40801 - 87672)	262320 (39905)	58481 (12163)
**NAT10**	**8591 (4038 - 15027)**	**96920 (54353 - 231001)**	**9119 (2922)**	**109283 (41426)**
NAPB	2013068 (1472177 - 4276504)	101307 (46167 - 229045)	2352368 (827785)	117811 (45210)
MROH8	878248 (282836 - 3252302)	12425 (8124 - 67371)	1023061 (602094)	15790 (11568)
**STN1**	**5219 (3183 - 10723)**	**53239 (24253 - 118656)**	**5638 (2086)**	**54135 (20787)**
ACSS3	378475 (204712 - 617112)	26884 (16795 - 90629)	391437 (110685)	32316 (17802)
DDX21	100845 (55519 - 189258)	10101 (6107 - 23795)	107545 (34918)	10945 (4247)
ARHGAP35	177527 (145644 - 234164)	42212 (29498 - 97926)	180582 (25601)	45171 (13790)
STAB1	230131 (149218 - 294842)	14593 (10456 - 19196)	229096 (39891)	14563 (2251)
FAT2	1336947 (958186 - 1548298)	125232 (103777 - 199757)	1328242 (154891)	136808 (25340)
PLCE1	1408686 (603561 - 3491920)	30939 (25308 - 48373)	1660931 (830730)	33336 (6855)
PTGFRN	127488 (75942 - 229550)	10525 (5820 - 14452)	139956 (42753)	10379 (1949)
CC2D2A	319799 (155023 - 652972)	23692 (11295 - 54815)	356238 (119884)	26082 (10210)
AASS	40388 (24050 - 72579)	13196 (8093 - 20905)	44513 (13375)	13670 (3636)
SWAP70	315902 (179269 - 431936)	77579 (54373 - 156698)	306717 (77755)	84415 (26701)
NPC1L1	436257 (163396 - 990744)	59973 (31205 - 161481)	475363 (220059)	68585 (30861)
ADAMTS1	50858 (24794 - 134391)	8250 (3092 - 20909)	58982 (28473)	8852 (4358)
**ACIN1**	**8794 (5047 - 14403)**	**339009 (249594 - 821651)**	**9029 (2234)**	**377052 (144688)**
AGO2	63118 (44777 - 113368)	15912 (9191 - 26887)	67536 (18377)	17053 (4294)
MYH2	329763 (219013 - 547980)	75141 (62554 - 107308)	338342 (84469)	77805 (12099)
HECTD1	55394 (29563 - 108787)	9676 (6510 - 20855)	58952 (19000)	10185 (3541)
NUDC	386507 (113937 - 679155)	35874 (8707 - 66877)	375822 (164699)	39065 (18380)
IRS2	1341997 (942664 - 2086938)	116039 (50483 - 321607)	1388941 (319779)	144069 (68094)
**MAP4K5**	**133406 (35817 - 229823)**	**1092542 (593727 - 3947095)**	**145565 (50228)**	**1379137 (794313)**
TBL2	2002690 (754806 - 4620120)	9441 (5851 - 32149)	2199942 (858629)	11620 (5733)

In bold characters, the proteins with lower abundances in High-grade EC Cases if compared to Low-grade EC Cases.

Supplementary Table S5. Proteins identified for having abundance values completely separated between High-grade EC cases, Low-grade EC cases and Controls.

Gene Symbol	High-grade EC	Low-grade EC	Controls	High-grade EC	Low-grade EC	Controls
	Median (Min - Max)	Median (Min - Max)	Median (Min - Max)	Mean (SD)	Mean (SD)	Mean (SD)
PRRC2A	236649 (180035 – 355575)	66871 (48792 – 110319)	20192 (9137 – 41354)	252044 (47467)	69380 (13329)	21545 (8485)
SYDE2	48434 (31232 - 77354)	115536 (91059 - 175565)	7562 (1225 – 18275)	49349 (10613)	123656 (22153)	8332 (6023)
