# Supplementary material for: Data-Independent Acquisition (DIA)-Based Proteomics for the Identification of Biomarkers in Tissue Washings of Endometrial Cancer
Source: Int J Mol Sci. 2025 Nov 27;26(23):11498. doi: 10.3390/ijms262311498 (PMC12691889; doi:10.3390/ijms262311498)
Supplement: Supplementary file 1 [file ijms-26-11498-s001.zip › Supplemetal file S1.pdf]

Descriptive statistics of all proteins identified with at least 60 % of values in the CTRL group (N=25)

PG.Genes	n	mean	sd	median	q1	q3	min	max
PPIAL4E;PPIAL4D;PPIAL4F	25	113359	72219	97915	53967	152770	25905	260552
CENPVL3	22	4227	4050	3033	1343	4768	693	14548
TEX48	23	8780	16689	6036	2897	7042	1042	84397
CPHXL	21	56335	165957	16196	5212	30469	893	775494
ERFL	25	86828	110917	62734	28408	86148	19273	583075
TRBV4-1	24	64462	43285	57471	23853	87038	10429	168081
UBA6	25	17856	8101	16931	12957	20594	6185	42818
ESYT3	25	76698	39911	59264	51819	88925	36885	192138
UHRF1BP1L	25	22860	11985	20879	15181	24534	10097	59163
SHTN1	25	108479	147597	60916	42697	99516	29942	716763
TLDC2	25	1531182	1172929	1256381	1030490	1635120	717693	6799682
BICDL2	25	13383	8468	11537	7198	17572	4300	33908
ARHGEF37	24	2980	2088	2534	1491	3930	224	10238
MEX3A	25	61792	57153	41784	27042	62118	11349	239656
ELOVL7	25	123066	83167	111767	64639	154614	16296	417366
SSC5D	22	2476	1443	2315	1760	2734	20	6803
SH3PXD2B	25	58763	37284	48761	42771	64421	22780	215989
PIPSL	23	14893	16429	10186	7095	15955	2145	81096
HYKK	25	9970	10217	7472	5796	11382	3523	56790
CRPPA	22	8173	5828	7607	4154	8962	199	20047
MEGF11	25	34383	81313	15920	11487	23273	3102	420578
FAM172BP	25	34734	48144	19616	13895	27701	4326	219319
VWA3A	25	13436	24351	7573	6284	10324	1925	128096
C2orf78	25	18078	5553	18039	14232	21370	7459	30715
ANKRD33B	25	215006	128783	214608	119225	286774	30378	545525

PG.Genes	n	mean	sd	median	q1	q3	min	max
IQCA1L	25	12056	8506	10006	8235	12553	4787	49148
PALM3	18	3389	2974	2271	1697	3633	618	11430
PGP	21	1866	836	1839	1211	2272	709	3607
TRIM49B	15	1703	819	1695	1279	2056	243	3348
C5orf51	16	2865	5282	1498	744	2508	456	22396
SOWAHB	24	7268	7318	4939	3867	6488	3055	33601
MROH2A	25	21076	12245	17689	12860	24899	8271	55251
ANTXRL	23	5644	3744	5223	2582	7619	1541	15479
WIPF3	20	3392	2925	2950	605	5577	180	9825
PCARE	25	14255	8005	11284	8281	17828	3971	34889
ANKRD61	18	324527	386568	224844	131938	395004	2589	1713948
OTOL1	23	15925	22242	8342	4736	17906	2609	108561
FBLL1	25	6095	8415	3310	2282	7091	1188	43946
SMCHD1	25	1209820	991691	950803	628528	1455361	306724	4687576
CASTOR2	24	5014	1786	5052	3940	6070	1639	8924
UNC119B	17	2458	2222	1854	1352	2406	214	8623
LRRC72	25	13597	17336	9380	7050	12632	1572	91043
SDR42E2	24	24804	58843	11957	5386	17398	1371	295983
CPSF4L	22	7344	4054	6621	5243	8532	3215	22253
ANXA2P2	22	36122	25986	32781	17748	50105	2739	115297
NA	23	175122	439118	41758	13200	163823	7900	2144014
FAM90A27P	22	6403	12234	2746	1501	4761	888	58493
ENO4	25	2897	1559	2202	1890	3689	916	6475
C4orf47	25	44379	34656	39584	24296	47943	13573	192107
SRRM4	17	3697	3925	3092	1608	3977	421	17680
A2ML1	25	59523	25182	52537	41265	71121	30476	134872

PG.Genes	n	mean	sd	median	q1	q3	min	max
NEURL1B	23	9553	12545	6130	4940	8646	2363	62156
PRSS47	24	7315	4505	5695	4078	8693	2998	19776
BBIP1	19	2694	1357	2410	1724	3543	880	6339
SERPINE3	16	3923	2138	3846	2186	4664	1299	7694
FADS2B	24	32617	22825	27587	17253	36346	5449	106207
CAPN14	23	18961	19891	9695	7000	26562	1318	86629
NA	24	9433	9983	7190	5174	10222	2994	53848
NUDT19	22	6685	10627	2974	1676	4668	506	40223
DCDC2C	25	7173	10216	4708	3055	7403	431	53376
ESPN	25	202698	815240	29802	16255	60073	7635	4111680
SNURFL	16	3936	3067	2554	1606	6214	852	11167
ZNF487	25	73294	91676	42352	36688	60600	21920	390245
OR11H12	23	53379	80993	22383	12049	40422	333	362591
ZNF732	24	24437	19752	18733	11639	31507	2550	93021
ANKRD66	24	61647	110325	37611	25118	46035	10324	565933
KIF28P	25	8524	4007	7604	5341	10323	3486	16888
C4orf51	23	11946	13387	7954	6284	13290	3305	69838
HSBP1L1	17	37955	34770	21707	11878	46827	5495	117262
AKR1B15	25	17841	9892	14049	9248	25256	6894	41567
MCIDAS	21	8197	12442	4579	2342	7621	646	58741
PROB1	25	79531	93420	41620	31694	71251	22750	400628
NACA	25	98223	73729	71718	65418	114003	37545	416791
C11orf98	19	1399	859	1534	860	1868	96	2808
CROCC2	25	36084	25567	30358	24560	40934	10439	145848
TRIM77	25	38863	58810	23053	19782	37264	4505	312669
ASDURF	25	101857	116921	62377	29807	159726	3834	544131

PG.Genes	n	mean	sd	median	q1	q3	min	max
OVOL3	25	135569	532989	19425	15757	34751	4345	2691057
AGPS	25	16249	51961	4481	3143	6026	931	263838
DDX39A	23	16306	14853	9661	5094	26189	865	57865
PDLIM1	24	12982	10843	9342	4729	20048	1386	45748
AIP	19	2950	2071	2263	1216	3979	700	8508
STXBP3	25	63136	103156	17404	10417	33116	4808	352086
LGALS8	25	132868	99449	104587	62662	176723	10775	398247
NFKBIE	25	90051	150730	58140	30875	86501	4259	780241
PSMD11	24	5476	5864	4022	2721	6254	1893	31320
PSMD12	24	5500	9465	3385	2402	4980	1358	49185
PSMD9	22	5817	3008	4941	4409	6327	3139	18345
RNF103	24	3362	1167	3214	2630	3987	1408	5932
ATOX1	20	2925	2528	2367	809	4175	425	9820
MEN1	25	13765	10079	9844	9456	14927	4049	56084
TAF4	24	337995	400526	216524	177405	324976	105814	2032171
DFFA	24	6452	12617	3473	2277	5745	626	64465
CLIC1	25	31044	26458	20939	11504	42830	2484	94309
EIF3F	18	1975	1453	1527	949	2539	332	5351
QSOX1	25	16044	15698	11772	8217	14214	3003	71336
DCTN6	16	3033	1612	2910	1766	4669	813	5234
WASL	19	2893	3770	1867	1417	2169	1014	17521
PDE2A	25	18591	18901	13536	8387	18208	4796	83589
FOXN3	17	2300	2303	1602	808	2389	252	9916
POLRMT	25	11366	15247	7745	5402	9513	2839	81139
EEF2K	25	32932	22535	23371	15452	55211	6734	74670
EML1	25	25788	29586	12935	9572	33940	4378	137244

PG.Genes	n	mean	sd	median	q1	q3	min	max
IGF2BP3	25	7946	7879	5942	3701	7948	1078	36802
GOLIM4	25	55833	34026	44008	39662	68431	9933	151464
EXOC5	25	17268	16244	13513	9634	16511	8330	85939
HMGN4	15	3166	3434	1397	983	5368	162	12365
PSMD14	23	3750	1921	3240	2162	4887	1411	8286
USP9Y	25	25697	29290	18154	13667	22571	8228	155828
LAD1	25	7249	4900	5379	4229	9690	318	18807
VWA5A	21	90717	387141	2630	1606	6424	594	1779845
NOP56	25	4299	3436	2948	2363	5085	857	16703
RNASET2	22	12186	12390	6742	3450	17493	1234	51577
GABRP	24	26820	18094	23180	13893	34433	4382	74242
PODXL	25	16605	18120	10251	3630	20200	1676	63620
FCN1	24	12032	24447	6545	3249	12147	990	124674
PIR	23	5673	11479	2415	1554	3144	802	43800
KPNA4	18	4030	3143	3407	1698	5411	798	13401
NFIB	25	218749	172875	151704	119414	199137	53880	735394
PIK3C2B	25	28693	30662	21548	18668	25430	10400	169762
FBP2	19	3272	2288	2190	1738	4055	444	8654
UBE2C	18	3783	2721	3159	2230	4092	715	11361
PDXK	25	135581	148004	94345	49195	177048	6548	737543
SCD	20	7838	4187	7541	5133	10797	914	15170
ISLR	25	21870	12691	18523	15898	24716	4269	57894
NCKAP5	25	44785	57337	27294	22954	30920	10590	254193
CUX2	24	8236	6545	6794	5215	8885	1987	34476
SOCS6	24	9696	7741	7936	3276	15170	1162	27111
TRAFD1	22	9371	10513	6861	2504	11737	695	47016

PG.Genes	n	mean	sd	median	q1	q3	min	max
HSPB6	21	23743	32059	13570	2021	35733	409	115703
UBFD1	15	2735	2449	2019	1335	3336	201	10140
COPE	22	3821	1924	3242	2758	4200	1141	8544
CCS	24	9580	18826	5481	4391	6924	1997	97319
ENPP3	25	73119	93200	41486	24606	90143	10029	470936
DVL2	25	5054	2220	5244	3089	6874	1447	8954
DNALI1	25	65754	66374	53372	35490	76833	11469	357855
ADAM10	19	1920	1839	1354	963	2162	327	7840
MAP2K7	25	9922	5162	9423	6818	11113	2313	27347
PDCD5	21	4436	5172	3190	1035	4721	290	23249
SLC9A3R1	25	25414	13010	22110	19409	27407	10199	71332
TPP1	23	10645	6909	8878	5784	13847	1069	24515
TCERG1	25	275548	747961	58608	34371	163980	13028	3768870
KIF3C	25	1301452	1018975	890800	673316	1796843	474840	4782870
UNC13B	25	112747	107160	80630	62911	120556	32528	537766
POLR3A	24	6957	9974	4061	3304	6177	2519	50857
PSMA7	25	12616	11944	9248	7586	13234	5351	66141
OPLAH	24	20613	14828	15945	12111	25527	9606	81326
SLC30A4	19	1855	2033	1608	491	2393	114	9035
IFIT3	25	10055	5735	8512	6974	10536	2326	27730
IRF6	25	12479	21959	7805	3384	12423	1404	109365
GIPC1	25	8033	4349	7514	5657	9604	2091	20174
HAT1	23	6659	12002	4196	3395	5168	1556	61207
MYL12B;MYL12A	22	5059	4551	4108	2294	6226	1044	22519
AURKA	24	41652	28491	36168	23150	52039	9834	142191
CLGN	23	6742	4206	5698	3793	9145	581	17199

PG.Genes	n	mean	sd	median	q1	q3	min	max
PPP1R12A	25	30617	27028	22894	17235	32409	10039	142775
AZIN1	24	24456	19598	17299	13420	29124	3952	90588
XPO1	23	6314	5367	4774	2523	8483	1521	24904
PLXNB2	24	6574	2394	6003	5131	7197	3723	13168
SEMA3E	25	127434	68260	129058	71646	157737	28621	305637
U2SURP	25	18504	19165	13666	10986	16975	8957	103944
SETD1A	25	81232	56526	61825	48057	97872	27678	294312
KIF3B	24	8256	11841	5066	3591	7262	2416	61371
PFAS	25	13006	7658	9679	7984	16428	6209	38496
NACAD	24	4897	5876	3820	2846	4799	1032	31772
ADAMTS3	25	29422	58775	15554	11464	21224	6238	307649
LSM1	23	10942	15492	5784	4017	11849	1151	77833
ARPC1B	23	46282	37721	36174	21307	60063	5144	137027
ARPC2	25	15500	8947	13727	8267	21845	3782	36999
ARPC3	23	11115	9276	6644	4079	18291	1219	36034
TBXT	25	22319	12476	19752	14223	30460	3166	53535
PFDN6	23	8946	6606	6664	4675	10184	3237	28861
LAMA5	24	5170	3403	4189	3487	5313	2708	19517
CLIC2	17	4150	4215	2575	1968	3928	9	13213
RTL8C	19	3416	3163	2300	1338	5046	262	12906
MAPK13	25	20014	12303	20563	10019	27664	2478	49735
SPTLC2	24	13829	14767	8572	4329	15313	846	53165
FANCG	25	14629	18657	8433	6336	16772	1454	96001
OGT	25	97008	77295	76432	64402	96194	46662	447459
PMM2	24	7863	4795	5812	4293	11840	1499	16896
HMGB3	25	7196	8228	4598	3170	5823	1712	34906

PG.Genes	n	mean	sd	median	q1	q3	min	max
PPM1G	25	34775	75611	13590	7741	16968	5572	311504
INPPL1	25	33503	27449	25911	18713	35125	9489	143795
EIF3H	24	14372	9353	11636	7473	20036	3651	41260
NVL	25	40818	31931	29053	22672	45436	16758	152678
BCAT2	15	3975	5592	1278	845	4878	475	21075
BCAT2	22	3124	3603	2475	1077	3434	567	17324
STX7	20	3591	2511	3110	1610	4924	621	10560
SLC16A6	24	24775	18109	19815	12613	32053	1176	81383
CAPN5	24	21512	41483	11301	8978	15907	5521	212481
YKT6	25	48563	108783	17415	15850	35559	6019	558124
ARPC5	23	19865	16935	15088	6657	31964	1908	70920
NKX2-8	19	2369	1381	2287	1479	2863	849	6050
DDX3Y	25	81548	219445	36444	23790	48781	15081	1131611
RGS5	25	109131	258733	55823	40045	60095	29793	1341926
DHX15	23	5152	3591	4634	2393	6717	1254	13746
ZZEF1	25	25335	10782	24176	20730	27545	9630	65398
FLRT2	21	4577	2053	4659	2979	5750	1381	8523
CYP26A1	25	104111	160356	66432	51645	81445	31674	849818
PHGDH	25	52870	31297	47285	34774	67825	17279	165469
GPR39	25	11412	6530	9394	7510	12281	4426	33032
SEPTIN4	24	45514	47713	34418	25622	47647	15808	261259
DYNC1LI2	25	7871	4180	6948	5823	9322	1234	21776
KLK10	19	3424	1673	3212	2346	4049	1209	8371
PSMD3	25	9957	4021	9167	6638	11961	5278	21947
PAPSS1	25	30253	44529	17861	13001	24260	2099	229255
SART1	25	112019	85227	90239	77425	111998	51970	500452

PG.Genes	n	mean	sd	median	q1	q3	min	max
TGFB1I1	24	24855	25859	18145	14701	28333	4528	136854
SRGAP3	25	209873	221010	138572	86059	263327	26533	1051151
ZNF264	18	4210	2438	3755	2586	5132	1198	10303
HSPA12A	23	40461	44916	29037	20097	42639	13349	235078
MSI1	23	13933	37194	5673	3533	9264	1563	183990
WDR62	25	198848	124591	165847	132067	229001	51498	701023
PRPF3	25	24352	36161	17561	10953	24129	1406	192717
TXNL1	25	9669	5494	7919	5096	13753	2875	20547
ERI3	24	26785	46527	12488	4703	23596	3444	219462
FIBP	25	31882	30430	23117	15785	30723	10601	155809
EIF4G3	24	12631	3543	11916	9244	15168	7382	18979
PPIH	23	5864	5768	4830	2716	6821	1046	26133
HTRA2	24	5320	2581	4859	3840	6624	1466	12324
AKR7A2	24	3479	2865	3407	1727	3741	721	14235
EPB41L2	17	9599	5150	7252	5880	13716	734	17844
EPB41L2	25	5386	2153	4480	3905	6680	3100	12175
WIPF1	25	78541	48836	58065	47192	125124	9287	194868
XRCC3	25	33841	10014	33411	24431	39907	18865	50864
DENR	22	10484	5051	8827	7447	14002	2832	20700
XPOT	23	37871	43370	22586	6081	57128	2628	191848
DNPH1	18	2615	1255	2524	1877	3642	731	4901
DCX	23	32798	24239	25637	16365	41539	3245	92901
TIMM44	24	61387	41255	52295	41107	67309	15527	228416
TRAPPC3	22	4348	6795	2914	2118	3365	1449	34299
CHMP2A	25	18401	21689	12297	9323	21131	5975	115826
NCK2	24	28474	27562	22969	18672	26525	12795	154868

PG.Genes	n	mean	sd	median	q1	q3	min	max
PSCA	25	485655	269195	442333	289508	694134	39080	1011010
PLRG1	24	10371	12542	7403	3558	10684	1479	63385
GET3	23	3979	1834	3827	2504	5015	1582	9357
ACTN4	25	18912	16556	14307	9185	22987	2847	69480
GSTZ1	24	11709	13638	8189	5487	11801	2424	71162
HTATSF1	21	5202	3313	4561	3351	6007	282	13725
STX6	23	11732	18183	6377	5281	9954	2495	89225
SYNGR1	17	8962	8536	6313	2291	11353	380	29000
SYNGR3	24	87623	50767	73250	61332	113444	7022	222522
SGTA	23	2802	1830	2497	1396	3695	548	7447
NARS1	22	6924	4383	7436	2511	10235	893	15551
NUDT21	20	9234	14122	3617	2366	9903	708	63912
LANCL1	23	7664	5377	7264	3020	11813	777	20079
RRP9	23	33360	89837	13090	8419	17174	562	442631
B3GALT2	23	7715	5623	6191	4874	8194	2502	29994
IDH3B	23	6504	4158	5408	4510	6922	2255	22926
AHCYL1	24	7008	3014	7326	4354	9069	2669	12685
CD5L	25	11597	10225	7104	5281	14312	1376	37121
PDE6D	18	2245	1945	1617	1343	2690	505	8536
RAD21	25	37588	21663	35797	24133	51208	1275	79583
DHX16	24	9100	9331	6549	4437	9416	1716	49251
ZNRD2	24	6617	2573	5605	4666	8178	3522	13089
GMFG	16	2260	1767	1728	1411	2469	780	8109
PPP1R12B	21	3373	3465	2100	1768	3019	838	13225
ADGRB3	25	41656	84674	8113	6040	18743	2750	353763
SMARCA5	25	965303	689410	705331	496931	1306143	208525	2975378

PG.Genes	n	mean	sd	median	q1	q3	min	max
KIF5C	25	14747	15562	11548	8323	14791	5101	84298
NUAK1	25	33921	42162	22696	17616	32063	6212	217930
ZNF862	20	82194	358541	1164	410	3556	12	1605440
SIPA1L3	25	4275	2021	3900	3175	4928	2154	12618
AQR	24	310575	192069	317177	137536	402600	20840	931360
MAST3	25	136679	178248	102207	73124	130654	23250	965551
OPA1	25	75399	83242	51446	46332	67176	37168	454559
PPL	25	1526609	2243822	1012047	908406	1320344	527561	12165718
PLXNC1	24	29304	64716	12572	10785	19155	3001	322418
ACSL4	25	202699	137025	162401	140224	192227	91892	785053
SNX3	22	5332	5287	2765	969	8702	280	16794
CUBN	25	22226	11878	18015	15519	28010	7765	57188
DOK2	24	20473	60593	6880	4552	10240	1407	304052
SORBS3	25	14611	9663	9852	8341	20726	6178	39323
CDC40	25	6227	6545	4672	3873	6295	1584	35763
RANBP6	25	41169	57949	27735	18546	35726	2091	305008
CCNT1	21	6182	7868	3843	2492	7580	662	37681
CCNT2	25	10721	14597	7049	5324	9789	2644	78076
TLR5	19	4455	2121	4112	3043	5601	456	8995
TLR2	25	27265	31492	19603	12765	25489	1988	149578
SELENOF	20	2038	2660	1293	1044	2072	594	12988
EXOC3	25	5497	3778	4628	4062	5283	2064	21539
JAK2	25	33709	122975	6220	4085	12888	1876	622696
MAFK	25	110771	104911	50248	33702	175487	2528	356428
SRPX2	19	9011	6872	8463	3754	10690	1746	25854
UGDH	21	7636	7688	4708	2107	11257	1191	28037

PG.Genes	n	mean	sd	median	q1	q3	min	max
SNX2	25	10775	6917	9823	6218	12357	3875	38979
DPM1	24	5382	8816	2941	2118	4501	833	44832
CCDC22	25	108291	269903	42367	31503	63938	9396	1380328
DKC1	17	2048	1747	1539	808	2346	192	6116
EIF5B	25	74290	114410	50068	41126	59502	22029	614600
MMP20	25	14726	10013	13356	9023	15788	5761	57030
DNAJA2	25	125050	241162	61632	47254	96823	15438	1242331
BRD4	24	7077	3613	6011	4705	8750	1915	17512
CUTA	24	16748	14322	12376	7679	18356	2154	53489
CTSV	23	7018	10518	3061	2429	5427	1016	42622
PFDN1	25	8411	9329	5013	3409	8544	1811	41573
NBN	25	34717	26632	30365	23916	35313	8156	139425
DTNB	25	88548	325638	18494	12061	26339	8407	1649357
KIF21B	25	7323	5248	5998	5021	6913	3348	30550
SRGAP2	24	24585	15999	23401	13326	31749	627	58949
WDR1	25	28273	29694	21370	6337	42409	2403	134963
N4BP1	22	5422	7289	2895	1784	5508	673	33254
ROCK2	25	171791	72953	153027	124483	192187	69273	394393
CLASP2	25	98309	99981	79025	68346	95380	43578	570285
CPNE3	23	5546	8264	2927	1971	4097	1031	35566
CLUH	25	17305	17000	13257	9725	16430	3415	91986
TSC22D2	23	16298	13017	12222	9248	19970	2042	60269
CNOT3	17	2785	2405	1940	1330	2559	433	8955
GGCT	23	4326	1940	3864	3099	5138	885	8846
ZPR1	25	8883	8128	6232	4380	10683	2265	43206
NIPSNAP2	25	19882	19412	14762	9597	21415	5835	105403

PG.Genes	n	mean	sd	median	q1	q3	min	max
CILP	25	22367	20488	16277	11509	21329	9544	101785
PDCD6	21	2515	1458	2276	1338	3245	936	6069
ZNF253	23	10819	16447	8059	4600	9871	1953	84538
TBCA	24	8689	5858	6465	4748	11233	2531	22982
ATP6V1G1	17	2241	2026	1347	854	2732	455	6240
VPS4B	24	8936	11550	6358	4335	8558	2889	61611
ZNF217	25	183884	83265	169795	135725	204089	77264	412165
SH3BGRL	24	91759	98253	33956	17611	163648	10640	368538
FLNB	25	47412	26283	40670	32437	60295	15508	119923
NCOR1	25	173670	89307	151382	126113	185032	98390	553920
NDUFS6	25	19988	37248	10976	8124	15437	4076	195748
ULK1	25	59974	28692	51590	45072	65580	27093	164445
CS	25	13139	13827	8261	6630	15827	2175	72882
SEC22B	25	12915	10964	8812	6144	16951	1451	50241
POLQ	25	83322	37488	71920	58629	95484	40104	186156
VPS26A	25	23596	12037	21381	13514	32856	7637	52020
PMPCB	25	75748	148788	34474	24458	67737	13473	768117
TECTA	25	24203	47736	12470	10178	15455	5831	244412
KATNA1	25	8437	6542	6750	5207	8076	2776	28516
RDH16	25	30478	20801	30572	14288	40077	2935	87769
ERN1	25	7009	7757	4329	3419	7310	1797	38789
PSIP1	25	29134	33662	23302	14975	28748	5086	181158
CLN5	22	11541	8835	10237	5306	16013	34	32586
HSBP1	25	6787	10171	3755	3141	5584	925	42962
KHDRBS3	24	6784	3329	6313	4319	8148	2614	14810
RBMXL2	21	4659	1624	4249	3659	4988	2277	7974

PG.Genes	n	mean	sd	median	q1	q3	min	max
BANF1	22	18814	20026	13484	3141	28390	450	83930
SF3B1	25	100822	85846	76498	56205	110528	34680	464479
SCGB2A1	15	10887	11355	5064	3850	14708	361	37109
SKAP2	21	10569	17845	3214	1395	11855	791	79455
LEFTY1	25	61758	132448	24868	14897	45523	3741	677374
CREG1	23	546485	744319	376392	275502	511415	48962	3787710
SNRNP200	25	133006	148663	93668	76833	127767	65666	827488
TIPRL	24	149956	286141	76530	57679	144096	11594	1473718
RFPL2	20	3572	3228	2833	1678	4320	303	15694
PPM1B	23	28132	11087	24711	19861	34937	7493	49231
UTP20	25	424675	184546	375117	312010	522438	235352	1113743
RP2	21	3899	3129	2923	1993	5166	759	14035
GJB3	24	26952	29412	17687	6578	29225	1787	104205
STK16	25	98954	74952	78592	47958	128250	4707	289244
CRTAP	22	1680	1420	1306	1016	1773	104	6572
B3GALNT1	23	27770	58940	9285	6670	15908	5240	284480
TCEA3	17	6313	8868	3149	1364	4361	271	27096
PALM	25	80593	95017	53163	32879	85803	23557	491147
RNASEH2A	24	5868	7707	3997	3050	5664	1989	41125
EIF3G	23	10753	32592	2308	1504	3616	155	156873
EIF3J	25	10943	12319	7581	6051	10538	911	64263
CBR3	23	8838	15963	5633	1982	7718	920	78612
PSMD10	21	2305	984	2088	1557	2382	1339	4929
ZMPSTE24	25	18250	11317	14767	12415	21006	6710	62120
PPP1R37	25	83612	168729	34038	21805	64136	11689	849780
IDH1	25	20256	17646	13973	9156	24017	3134	75787

PG.Genes	n	mean	sd	median	q1	q3	min	max
GATB	25	153909	407119	9165	5864	17409	1572	1764200
SCO1	23	4320	4650	2809	835	5734	282	16090
ATRN	25	20988	61061	7620	5357	9553	2771	311576
RBBP9	20	11509	12404	8418	3965	13056	1993	57619
STAM2	19	2831	2370	2023	1382	3300	436	8615
SULT1C4	20	18559	17871	12767	6941	19493	2896	61910
DCTN3	18	12527	35168	3771	2116	5831	463	152614
DNAJC8	15	4571	4101	3141	1829	5602	794	16659
ATP5PD	25	30330	24419	21424	19906	30185	10895	125439
GLRX3	24	21009	51496	10639	7206	13733	4508	262176
RSL1D1	21	8891	5242	6934	5320	13605	2442	19870
WFS1	24	88524	197286	43743	32467	68622	13679	1008052
SNCG	22	3144	2452	2164	1517	3772	470	11551
CIAO1	19	2748	1648	2180	1708	3602	508	5910
DFFB	20	1714	2078	978	764	2077	312	9618
SRP72	25	8202	7302	6031	4280	7987	3112	37293
DDAH1	25	12518	8730	11010	7738	15416	2200	46667
MTA2	25	12603	7845	11551	5805	16354	2129	28348
KBTBD11	25	23399	32694	16539	13505	21363	8978	178374
ATP10B	25	25155	35163	16786	12353	24209	8381	190178
TOMM70	17	4377	10713	1488	749	3105	231	45642
IPO13	21	2487	2564	1692	1016	2948	503	12225
TOX4	22	5463	5596	4052	2483	4898	23	21582
SEC24D	19	3779	7822	1547	1304	2467	348	35527
FCHSD2	25	15135	19107	6702	4974	14114	1310	73686
SASH1	25	41875	24650	31989	25161	50732	3857	115108

PG.Genes	n	mean	sd	median	q1	q3	min	max
TMEM63A	24	25039	10311	23960	19252	31639	9739	43249
UBXN7	24	30111	91517	11368	7580	14118	5102	459196
SUN1	25	23828	12642	20821	15409	28557	9015	64161
PLPBP	22	11006	4948	9351	7631	11896	4979	22706
ERLIN2	25	24099	28154	18110	13273	23908	2975	152679
PCF11	24	3396	6068	1783	1415	2522	781	31254
ENDOD1	25	90045	272084	35243	26481	42905	14899	1393692
GLCE	21	10193	15417	5701	4192	9052	737	73014
FBXO21	22	4228	3159	2991	2369	5309	927	14265
RHOBTB3	25	31298	40920	22219	12418	30827	7065	211553
USP19	25	8707	4515	8111	5645	10101	1849	20207
AP2A2	25	6407	3366	5315	4327	7417	2196	17962
CLSTN1	21	11160	10512	9392	4147	14475	531	47330
AGFG2	23	109655	75235	82726	57507	162926	1740	293014
ZFPL1	25	31247	26138	25515	16507	40602	7585	140215
ELP1	24	3841	5359	2624	1635	3613	768	27778
NDUFA7	18	2521	2066	2110	1633	2785	223	9730
ZNF205	25	10632	14845	6552	5460	8759	3168	70806
ZRANB2	25	8783	23194	3100	1745	5544	723	118738
SNX4	23	8083	7725	6534	2442	10593	1275	27902
OR6A2	22	29980	31834	16165	10674	35328	3260	138302
LRAT	21	10407	11620	7189	2161	15686	746	48581
GOSR1	20	3878	1769	3475	2605	4744	1652	8529
KCNH1	25	11828	13229	6624	5080	12868	3509	67769
LYPD3	24	32407	65855	16365	11888	25311	3430	336719
VAPB	17	9767	8286	7410	5088	10331	1086	34629

PG.Genes	n	mean	sd	median	q1	q3	min	max
PGLS	25	15157	11188	13245	6995	18715	2653	51457
PAPSS2	24	110373	146413	67455	56979	111806	4697	734826
ATG7	25	7890	10276	5394	4430	7037	1966	55647
LYPLA2	24	18101	20930	10332	7836	18821	3275	87568
IPO7	24	14655	28063	8456	4769	13485	774	144180
SLU7	24	3885	5371	2617	1937	3332	800	28074
CD2BP2	25	7065	4630	5956	3561	9541	1512	17180
AHSA1	24	8509	25714	3071	1111	4679	507	128608
ABCA1	25	12030	19400	7716	4687	10693	3736	101498
H6PD	22	2784	2464	2012	1016	3648	527	9498
SEC24B	25	90122	68565	63739	44869	125715	1288	270597
PRAMEF12	25	147335	81576	136182	109293	176906	2409	392942
ETHE1	25	20135	46086	9930	7888	12093	5147	240173
ZBTB11	25	111677	124898	55420	43526	148914	8020	588222
STAMBP	25	11263	21852	5882	4036	8110	3051	114226
ECEL1	24	5890	2624	5036	3904	7796	2480	13956
OXSR1	25	29958	69799	9147	4936	11769	2565	323371
GGPS1	22	6758	6023	5704	2928	8438	1289	28273
LSM8	25	27820	19613	23504	14046	36531	2749	82694
AP2A1	22	2171	1139	2220	1076	2929	726	5036
WIZ	24	10134	4441	8858	6909	11639	3203	22404
DDX58	25	8266	6403	5640	4505	9531	2872	28966
CAVIN2	25	12254	15246	8823	6036	11507	2357	82640
BAG2	18	2888	3819	1105	359	4020	99	14902
BAG3	20	2388	3142	1014	422	2592	179	11355
CRYZL1	21	7578	6572	6031	3431	10230	353	30644

PG.Genes	n	mean	sd	median	q1	q3	min	max
AIFM1	25	248268	134677	211797	165256	316730	79628	679021
EML2	22	8890	4233	8080	5939	11828	2718	16720
NUDT14	19	4872	2557	4070	3167	5816	1398	12072
TSPAN15	21	75017	67413	62695	48355	92980	2741	339790
BPNT1	25	95216	133029	57046	37718	110767	8416	686078
DDAH2	25	21466	27480	13231	7716	22452	2699	139890
ABHD16A	22	64107	122271	38512	18961	48699	695	598744
TXNDC12	22	6275	5111	4934	2259	10062	1010	20443
ECD	25	19719	11810	17700	11614	29114	2128	46952
EFEMP2	18	1987	1100	2102	1020	2956	458	4016
SCGB1D2	15	17321	26412	6287	2478	16358	382	100308
RECK	24	14031	23460	8812	5120	12367	2713	120826
NUDT3	23	6621	4622	4525	2538	10356	1213	15969
AGR2	25	11129	6988	9478	7065	13493	3060	37236
PAK4	24	23389	22737	14933	12718	24556	5278	108873
APBA3	25	9113	6159	7140	5896	11705	666	27626
ACTL6A	25	46436	49669	29893	27339	52324	5462	271902
CYB5A	16	2469	3084	1839	727	2820	142	13243
ADH1B	25	73176	65408	46367	30515	85140	17598	260235
ADH1C	23	7565	5848	6173	4000	8778	877	28358
ALDH1A1	25	51188	30813	46281	26852	72234	7257	114074
SOD1	25	55870	29938	45433	34978	78388	15737	130245
CP	25	90403	81724	64982	55727	81221	21200	365091
PNP	24	14530	7619	14615	10152	17833	3019	38047
HPRT1	24	8774	3834	7951	6582	9930	3946	18370
GOT2	24	7808	4967	6189	3461	10276	1784	21111

PG.Genes	n	mean	sd	median	q1	q3	min	max
PGK1	25	69225	40359	62647	36573	90063	9408	156987
AK1	25	26046	13971	23327	19368	30733	3459	72921
C1R	25	8811	4791	7481	6005	9684	4039	24140
CFD	20	3558	2153	2927	1930	5308	1078	10051
PLG	25	35711	34444	28436	16368	37588	5337	177943
PLAT	23	6551	4087	5610	3418	8631	2052	16126
CFB	25	86619	102847	56419	48672	91729	18342	558040
ADA	25	14507	9614	11908	9126	17897	4800	53851
CA1	25	1056876	917345	806023	365620	1741377	12766	3126398
CA2	25	107622	96210	71740	30636	168978	2085	376720
ASS1	25	12223	9615	9743	7981	11894	4261	53675
SERPINC1	25	99666	131745	79236	36114	104246	21239	707427
SERPINA1	25	983810	1181921	791834	522559	977871	222894	6492400
SERPINA3	25	186976	81215	189364	134635	230630	13851	351518
AGT	25	24271	19410	19427	12450	27833	3690	88001
A2M	25	219072	112755	211873	133881	304491	45202	463546
C3	25	248008	280860	206028	104421	248546	67962	1526068
C5	25	41531	23498	38994	23113	47980	16769	110208
TIMP1	23	12996	15982	7398	3939	11381	2390	71738
CST3	24	13403	16099	8665	5674	10762	3794	64646
CSTA	25	14909	25111	7760	5629	14011	2527	131897
KNG1	25	126103	53618	119648	97381	148329	41440	272164
HRAS	24	5850	3938	4419	3063	8062	1095	14831
KRAS	22	3643	3442	2072	1190	4877	368	11366
JCHAIN	25	68224	116394	35753	12805	62105	3209	592856
CD4	25	81665	118504	40941	35741	60147	17758	612021

PG.Genes	n	mean	sd	median	q1	q3	min	max
PIGR	25	157101	285437	79273	6636	146754	395	1270802
COL1A1	25	36798	31682	26128	18501	33851	11761	140149
COL3A1	25	47882	31731	37753	24030	58556	18104	140840
COL4A1	25	60575	70598	43823	30559	60875	15262	380305
CRYAB	17	3219	3279	1732	631	3554	426	12169
LMNA	25	22812	44866	6146	3302	13377	1436	165522
APOA1	25	714413	378637	583597	475697	998738	49103	1527399
APOE	25	14637	10729	10673	7669	20098	4182	52104
APOA2	25	192987	166922	140822	92842	199922	34389	777401
APOC1	23	6694	6032	5296	2484	8837	686	24238
APOC2	25	45665	48929	34733	20262	49947	5865	254643
APOC3	25	36336	64397	15916	10204	40104	5831	333543
FGA	25	105757	164568	53980	30081	83522	16070	802184
FGB	25	126586	179387	66534	35697	96896	6097	855402
PMP2	24	11611	19058	6336	4869	10629	1781	98058
SLC4A1	24	9259	7363	6752	4911	10407	2837	33800
APCS	24	21265	17937	15438	12959	25383	4692	90377
C1QA	24	7694	6047	6681	3693	8464	166	25360
C1QB	22	5201	4472	3679	1956	5591	889	16287
C1QC	25	11788	12443	9013	3123	12566	1118	51058
C9	25	24249	13203	21968	16088	29943	9208	66793
APOH	25	81405	76743	61523	40473	81166	26766	333215
LRG1	25	54358	22132	50643	40622	67765	18800	102853
FN1	25	16130	26775	5441	3033	17757	1214	124746
RBP4	25	17220	11356	15219	8465	21796	2855	45832
AMBP	25	40889	28096	36518	27383	45625	16101	158346

PG.Genes	n	mean	sd	median	q1	q3	min	max
ORM1	25	224318	241668	171874	127744	212196	51632	1293610
AHSG	25	80335	45051	76426	43219	102191	15049	183354
TTR	25	62484	37629	69624	29730	95448	7277	137589
AFP	23	2790	2919	2166	770	3355	123	13138
PPBP	24	10905	12161	7142	3591	11697	1221	49668
PF4	20	7213	7249	3290	2302	12930	255	21697
TFRC	25	18082	33394	10883	7486	13982	4397	173673
TF	25	1804137	2704258	1424847	880477	1624714	518104	14571432
LTF	25	21788	19085	18921	10640	26975	957	89989
HPX	25	472513	522456	411432	234943	484725	144147	2889521
FTL	23	136770	235287	51762	32808	94459	14917	887751
FTH1	22	9657	15277	3825	2251	7764	582	66776
ANG	18	3891	3471	3681	977	4947	524	12896
KLKB1	25	39885	23537	33490	26974	46339	14291	127488
SLPI	24	20212	36369	5444	2381	19210	18	145808
C4BPA	25	23716	19867	15985	9109	40453	1456	64084
VTN	25	33976	22257	32673	20404	38346	4981	107387
CAT	25	86746	68042	70237	37027	128072	9686	280687
FUCA1	24	8052	12257	4705	3715	7114	1919	63569
ALDOA	25	74875	42593	65813	42367	92393	6762	166485
CSTB	25	43555	27843	31356	21756	63717	11485	110091
ANXA1	25	34651	22048	31978	18409	45791	7212	99914
APOB	25	30520	21263	28905	12695	40600	7336	83218
HRG	25	23075	25348	14814	10210	24945	3818	131472
THY1	15	9575	16214	5947	1825	7226	808	66459
A1BG	25	44740	20111	47232	27106	56554	11649	78987

PG.Genes	n	mean	sd	median	q1	q3	min	max
CD74	24	257153	565016	27832	8578	245486	3440	2676078
VWF	25	12472	8638	10270	7847	12775	4978	45815
SHBG	16	1957	1213	1479	1283	2340	628	5492
GAPDH	24	409785	356239	225376	127752	747076	72388	1149404
GAPDH	25	162814	91993	140366	91415	211546	13778	373741
CAPNS1	25	7601	3800	7885	4401	11053	1392	13949
HSPB1	24	41019	34942	37018	11816	57168	7857	139136
CYBB	25	29724	37645	19518	14855	29247	5574	200398
RPN1	22	14062	15388	9673	3884	16600	681	62976
ATP1A1	24	9573	17493	4755	3830	7479	2294	89719
ARG1	20	4044	7487	2063	1322	2972	491	34481
APOD	25	40554	25499	30655	24399	51177	5438	96715
ALDH2	25	23166	21439	16216	12369	26074	7079	109469
ITGB2	22	12275	23327	6241	3952	9495	1681	114359
S100A8	25	108925	142780	56113	23472	147949	6883	637220
SERPINA5	21	6457	7350	3431	1792	8441	270	29685
CFI	25	19780	17259	15119	11992	20626	4268	94528
ISG15	25	56432	143971	21849	17567	38371	9506	742357
PCCB	22	23144	69566	2754	1379	7377	388	328696
ALPL	22	4095	2464	3900	2129	6027	543	9300
ALPP	23	14372	10952	12034	7510	19053	1227	51944
EIF2S1	23	5542	4078	4108	2087	8083	774	14384
ICAM1	24	8376	10520	4819	2915	8468	261	43492
RPLP1	24	8472	11017	4517	2959	7413	7	50022
RPLP2	21	15197	30323	3533	1848	10825	964	119289
RPLP0	22	4388	5469	2053	1172	5660	600	24499

PG.Genes	n	mean	sd	median	q1	q3	min	max
FABP3	24	75478	40723	70067	45362	101857	17032	171208
POLR3D	25	25542	22955	19822	17243	23984	7459	122501
CLEC3B	25	9998	6702	7059	5095	14019	3181	28148
SSB	24	9540	8406	7331	3864	12249	2152	40716
SERPINA7	22	5730	4921	3978	2876	6564	1633	23345
SERPIND1	25	21184	14721	16177	10184	30028	3106	59178
ITGB1	21	2472	2012	2014	1338	2594	633	9769
PRKCB	25	79050	124570	48815	40220	70715	18983	668228
C1	20	7840	13746	3383	2068	6950	535	62840
COL5A2	25	31581	16343	25918	20302	38210	17756	87630
UROD	23	4503	2871	3669	2954	4979	1562	14924
BCHE	24	16449	13933	13579	9648	17822	1484	75942
GLA	22	2060	1234	1874	1243	2544	616	5503
GSN	16	2216	1677	1648	1063	2773	698	6595
GSN	25	85163	59986	59729	55817	95992	23667	273492
RB1	25	11130	6979	8548	7397	13688	4712	37339
PGR	23	4838	3015	4213	2318	6366	1457	11441
PTMA	17	11337	26478	3866	949	9023	274	112365
ATP5F1B	25	13092	16425	8038	7327	13061	3462	87314
C2	25	16904	10288	14211	11456	17878	5226	58734
S100A9	25	66072	80685	40080	17226	69150	5742	361545
S100A6	25	56628	43613	41581	23489	85658	14335	165144
APOA4	25	33139	23728	28537	11867	48266	3856	97742
EIF4E	23	3896	2714	2265	1798	5241	949	10901
ENO1	25	154576	93676	147013	83082	183506	16148	398639
PYGL	24	36225	21989	31080	27095	36840	8420	123585

PG.Genes	n	mean	sd	median	q1	q3	min	max
GPI	25	16806	10204	14552	7912	22334	4893	41250
POLB	25	82007	48339	71205	58516	91553	22490	225237
TPM3	25	41206	128664	14901	5582	22671	2604	656153
HEXA	25	41981	110335	18698	12190	25589	6964	568942
EPHX1	24	15649	13481	11610	9085	17862	1583	68092
LDHB	25	101266	47479	105553	61480	126683	9594	210216
GPX1	24	22157	53412	9874	7441	13107	3460	268411
PGK2	25	60233	42078	49796	37688	75654	17025	232031
PROS1	25	6046	5362	5350	3205	6682	1716	29038
P4HB	25	20087	18611	13150	5558	28577	1306	78414
H1-0	20	20469	19091	13136	4468	30513	658	56386
ACYP1	21	6066	6168	3997	3327	6000	2022	25605
CSF1R	16	2963	3141	1652	505	4702	108	9420
CTSD	25	62291	30948	59042	39890	84753	13679	122846
ANXA2	25	34036	26475	28492	16905	49046	4089	116159
C8A	24	6304	2651	6028	4316	7908	1869	13428
C8B	24	6856	3465	6058	4667	9168	1956	14518
GP1BA	23	5923	4453	4458	3438	6853	1813	22887
C8G	24	7098	8512	5156	3845	6995	1816	45607
CAPN1	25	198504	93588	174661	126747	235509	67772	458774
TUBB	24	18988	15857	15343	6256	29440	2785	72862
CA3	24	22147	22791	15045	11328	22811	2754	113633
IVL	19	12400	36390	2774	1987	5043	46	161619
DCN	24	43655	47606	27104	9418	65048	2720	207927
PSAP	25	23921	14958	21905	12790	29160	4621	62182
HEXB	23	5295	3504	4446	3531	5927	1185	14259

PG.Genes	n	mean	sd	median	q1	q3	min	max
PFN1	25	215035	194602	105450	54475	422579	9241	593439
BPGM	25	32309	26837	20161	13414	49072	4666	121502
APRT	24	38588	67323	21042	13402	29759	5774	339685
EPRS1	25	101247	48173	88663	76501	102577	50909	296486
CTSB	25	59655	64158	40953	18729	67309	10879	250112
LDHC	25	94021	134479	64654	47858	79516	27641	728747
HSP90AA1	25	90308	91586	64707	43377	104552	30901	496803
GALT	24	4598	5023	2661	1812	4475	1343	18936
HNRNPC	25	21175	16567	17895	7468	30680	5049	60806
LAMB1	25	35651	16376	33005	23210	44776	9230	78806
YES1	25	18555	9323	19808	9752	25056	5013	37970
TPM2	22	53279	59081	23548	6276	109299	1486	187317
FH	22	6172	3848	5486	3127	8196	1368	14689
THBS1	24	12373	11932	10539	2900	16832	1323	48691
RNASE1	19	5861	6704	2639	1197	9294	569	23708
COL1A2	25	29310	27624	20067	11549	31251	8348	110764
ANXA6	25	31809	30500	21152	9478	43739	2871	120603
RHOC	24	23148	32546	11664	6460	32248	2451	161385
SERPINA6	25	91606	140775	49193	37362	69683	25498	719853
SLC3A2	25	56208	52046	38649	24140	54372	1822	210029
GUSB	18	5009	4191	4007	2382	6356	329	17398
PFKM	25	14503	22275	8279	6479	13352	4091	118094
HSP90AB1	25	202059	124462	137994	108222	292296	17811	485358
ELANE	20	8911	11788	4418	3035	9339	466	52588
MMP2	25	3339	1880	3274	2010	3827	1021	8972
SOD3	24	22698	31492	14082	7717	22522	1864	156470

PG.Genes	n	mean	sd	median	q1	q3	min	max
CTSG	20	3639	4658	2039	995	4324	444	21066
ITGA2B	25	54945	25296	50241	41141	63834	3561	109539
LPA	25	8369	6085	6811	5140	9619	1966	32848
PLEK	25	18666	33220	7775	4718	16484	1491	168769
CD14	25	7006	6634	4513	3887	6099	2115	29224
COL4A2	25	10037	6710	7907	6663	11788	4267	37390
SNRPB2	24	9886	18734	5741	3949	7847	2224	96493
CFH	25	51049	29714	45497	29773	53801	16669	152242
SNRNP70	24	25518	81139	7368	5366	13107	3107	405621
FCGR3A	21	9473	13304	4791	3908	6374	393	51370
ITGA5	21	10769	5738	10820	7200	12463	681	25442
NFIC	23	45597	105892	11882	5711	37034	390	508741
VIM	25	122322	144528	57886	30531	139580	4260	518037
SERPINF2	25	36465	27062	32397	17595	42440	11457	146121
RPS17	20	2941	3847	1599	667	3796	189	17160
GNAI3	17	1541	846	1586	795	1618	554	4137
ANXA5	25	263324	232974	209985	95181	355652	19499	996023
RPSA	24	14958	11711	11125	5692	23697	2754	38878
MRPL3	23	5821	7101	4006	2708	5458	833	32164
SNRPA	22	10349	8850	7017	4814	12928	1638	36226
ENO2	24	55127	44823	30707	19173	81521	8912	146621
GSTP1	25	100799	81004	79518	46285	152236	6695	276233
MMP7	17	8417	10506	4046	2670	9836	292	42950
MMP10	25	39128	84815	8749	6612	12920	4537	372379
LGALS1	25	81828	119272	53210	28279	71999	13379	537793
QDPR	22	6752	4787	4981	3484	8644	1584	20565

PG.Genes	n	mean	sd	median	q1	q3	min	max
HMGB1	24	37415	38519	23559	9888	54506	2493	130371
RBP1	25	80086	146769	33080	14696	84671	2372	707197
FBP1	19	13622	33979	5078	4265	7525	2124	153617
SPARC	17	3271	3346	2405	1137	3470	774	13419
GSTM1	24	34206	38804	21672	13239	30803	7669	162789
ANXA4	25	21558	18154	12123	7704	34201	4230	71144
DLD	15	2542	2112	1996	908	3082	463	7032
SNRPA1	25	17692	10050	15577	10737	20379	8960	55010
CTSH	22	4077	2713	2983	1938	6165	578	10249
COX6C	25	12694	12014	9456	7228	12795	4182	62280
TACSTD2	25	5970	2750	5963	3962	7551	2239	14175
C1S	25	7092	3388	6518	4585	9193	2877	17755
PARP1	25	11011	7794	9911	5145	14001	2091	32329
IFIT2	24	49660	109525	17443	10786	28464	895	518001
IFIT1	25	9857	11433	6027	4771	8420	2183	55864
ALPI	21	3268	3794	2493	1422	3078	844	18336
UCHL1	25	45182	53397	29737	15303	47962	8031	244865
ALDOC	21	4390	1979	3674	2975	5832	1581	8719
NUDT17	24	15077	15878	10421	6227	14644	1801	75691
C4A	25	108088	54048	89218	71324	116162	42376	267082
C4B	25	8939	4894	7636	5902	10034	2903	22679
WEE2	25	44788	112473	19575	10367	34527	3259	579861
OR4E1	20	3089	5334	1571	993	2309	163	24520
ANKRD34C	20	9822	10486	5939	3341	12450	1023	43284
ATXN1L	19	2120	1911	1662	999	2620	446	8702
DCAF8L2	23	17615	10959	14055	10033	23521	5931	49794

PG.Genes	n	mean	sd	median	q1	q3	min	max
PABPC4L	24	67495	60229	44886	30286	90337	4532	252458
POTEJ	16	4045	3940	2402	1551	5412	516	14832
THEGL	25	32337	18036	33675	20324	40477	3332	69001
SPATA31C1	25	5091	6302	3072	2552	4674	1520	32594
HSPA1A;HSPA1B	25	123626	75971	123032	58840	151667	19306	287012
CBSL;CBS;CBS	22	17602	17568	11572	6785	21047	1269	73203
CALM1;CALM2;CALM3	25	86771	48642	76549	50040	105050	9585	205123
SCHIP1	25	8072	3948	6985	5608	10559	2454	18372
AMY1B;AMY1C;AMY1A	21	16954	13437	12949	7652	18160	4448	53938
FDX1	24	11829	7145	9118	6169	15566	2814	28982
RNASE2	16	6298	6289	4911	1504	9131	888	23281
GAA	25	70558	174797	24711	15881	64440	9325	899888
RRAS	22	3365	3506	2041	980	4309	481	13588
H1-4	25	90165	100664	46684	19380	113414	6762	330271
SPP1	24	7159	9673	3223	2208	9480	596	45419
DLAT	21	5815	10097	3586	2756	4326	1226	49443
NR2F6	24	8598	6484	6742	4976	10255	1119	31372
TXN	25	94098	70297	75531	46723	107689	22538	295621
COX5B	23	67311	54615	47302	34797	87433	8778	209189
CTSA	18	5283	6362	3284	1678	6902	648	27945
MAPT	25	31989	68703	14931	12968	20421	4010	356464
C7	24	10042	7968	6614	5468	11390	1915	34747
PRKAR1A	22	4176	1969	4259	2583	5191	1321	9725
CHGA	22	15918	33248	3499	2155	5310	309	114330
ALPG	24	13961	9086	11777	8736	15018	3343	47852
UROS	19	1972	870	1696	1441	2459	217	3516

PG.Genes	n	mean	sd	median	q1	q3	min	max
ESD	24	22078	16252	15917	11090	29953	2615	73353
HSPD1	22	14983	9600	12061	7304	20631	5280	35599
CLU	25	259064	412124	115688	85660	210169	48949	2093116
HAPLN1	25	28188	11809	27550	17981	35415	8238	47364
HSPA5	24	15753	12872	12500	6062	21538	1745	51342
LAMC1	24	14728	19227	7226	5668	9895	3485	70346
ACP2	17	4354	5869	1703	634	5102	175	20614
HSPA8	25	41996	23030	38827	21762	58467	4891	96427
EPB41	25	85900	48626	72478	47505	106765	33606	210979
UMPS	21	3436	6444	1555	1228	3429	226	30852
DBT	25	18320	7506	15883	13092	23650	6038	35534
PYGB	25	11339	7121	10526	5624	15350	3383	25470
MBL2	25	80870	64728	60747	38702	113667	14777	253867
RALA	19	40660	54368	22095	8848	45855	2116	231531
NAT2	23	9454	5928	8089	3940	12381	1434	23234
SPTB	25	15913	15169	12034	10156	15297	6693	83591
LAMP1	22	3070	3630	1924	1221	3280	639	17499
TOP1	25	25578	13727	20954	18013	25744	12895	63692
GNAT1	25	53683	87822	33169	11872	41834	5169	434814
MTHFD1	25	5729	3064	5052	3362	7638	1930	14711
ADH5	25	15105	12997	11472	5181	20108	2084	50898
CDK4	25	141289	170107	78410	36556	158539	15842	716371
PABPC1	22	13298	19641	6229	3397	13394	1212	85649
PCNA	25	7847	14659	3836	2114	6030	935	63864
HARS1	25	10840	24076	5361	3481	9576	503	125199
COL6A1	23	13446	13948	8501	6920	14047	3739	67688

PG.Genes	n	mean	sd	median	q1	q3	min	max
COL6A2	24	7290	16430	3447	2384	4583	1849	83482
COL6A3	25	86022	152140	45114	27190	63409	19116	780314
IMPDH2	22	4817	6420	2985	2205	4844	252	30641
TPR	25	99692	90729	78519	65081	93545	48600	508520
CKB	25	51068	55571	22082	12787	69359	4215	203701
ANXA3	22	10242	5865	9397	7013	13922	720	20739
BMP4	25	15989	18076	10726	7159	15914	3473	83180
BCKDHA	24	19309	10245	16310	13230	24074	5514	45700
RNASE3	16	3336	2935	2462	1609	4163	599	12595
ACTN1	25	54954	72143	25372	16354	60036	6544	296402
CDH1	19	5134	5448	3408	1725	4989	527	19288
MYH7	24	2780	2426	2489	1366	3176	521	12764
SRC	24	12824	10468	10270	8059	12491	6603	59579
PEPD	24	5283	2718	3883	3234	7775	2407	11347
XRCC6	25	14540	9731	13545	6206	19193	3792	40836
XRCC5	23	17332	15786	9672	5380	26501	1396	60751
IFI30	21	5085	10661	2084	982	3621	489	50428
RNH1	24	25508	19545	17831	12155	31146	3114	83091
SCG2	25	7025	5538	5347	3725	7270	2717	27289
EEF2	25	39816	31283	26873	16138	53103	9270	112589
PDIA4	25	11104	7743	9379	4151	17583	1300	24011
C6	25	7551	4945	6587	4290	8279	2744	21750
TPT1	23	8873	8567	4256	2299	12789	1350	31267
ALAD	25	42848	21140	36638	31041	59278	14248	100476
LCP1	25	32076	34462	18758	13379	31781	10262	162558
PLS3	25	25709	15669	18008	12662	37984	7395	57501

PG.Genes	n	mean	sd	median	q1	q3	min	max
APEH	25	23838	10440	23181	15554	26659	10253	52322
ETFA	24	7924	8469	5517	3736	7306	1916	41736
GYS1	25	19221	12997	15042	9807	22834	5811	60658
GTF2F2	24	67529	83338	34489	18244	76817	6557	318243
MIF	25	140930	118762	84085	48343	218788	6302	398942
PRKCSH	24	9813	9163	7503	3707	11428	654	35908
HCLS1	25	50181	60607	31432	26582	35532	19797	255390
FDPS	25	10043	7881	7518	5589	10110	2755	39712
CPM	24	20085	19988	16890	11927	21807	1821	99310
NID1	20	4034	6643	1871	1162	2993	631	29752
AKR1A1	23	13211	9411	11515	5397	19218	1603	34142
PKM	25	124005	64443	99840	75968	172996	32160	258266
PKM	25	19129	14467	15244	12175	20871	6544	82150
ACYP2	24	138362	168036	95759	58811	177774	5643	868484
HSP90B1	24	45392	38544	26086	19173	79708	3269	144905
CCNB1	25	68277	59039	48121	34392	91352	7826	248689
IDE	25	9086	12649	5549	4631	8266	3566	66946
MMP9	20	2526	3523	1523	885	1931	11	14405
HNRNPL	24	9589	7210	6682	3635	12540	1667	23047
DARS1	24	13795	10214	11256	6174	20578	2542	44964
IDO1	20	13867	12359	8977	4914	18692	1585	39530
JUP	24	31610	35570	22169	13631	35923	3799	183662
FABP4	23	7767	7334	5355	2520	9700	670	26759
GLUL	23	3476	2284	3268	1859	4357	989	9741
AKR1B1	25	54947	54417	39142	20104	64448	11545	259378
RAC2	19	7258	9812	3419	2570	5238	853	34692

PG.Genes	n	mean	sd	median	q1	q3	min	max
CPN1	22	6143	5236	4617	2904	6322	1186	21285
GSPT1	25	17558	15612	13075	11277	17216	7026	81176
PGAM2	21	5420	4219	4170	3083	5252	888	17752
IFNGR1	25	32605	19797	27353	17652	42114	10488	80713
EZR	25	24234	13204	20927	14750	31530	7315	53860
UCHL3	24	9287	6564	6381	5424	10863	3818	33877
FOSL2	24	7747	5125	6098	4431	10086	1514	23143
NME1	17	2883	1525	3318	1591	3623	520	5443
GNS	24	28568	61469	14044	10652	19594	8735	314809
ARSB	25	40135	24009	34504	29847	42287	8378	138189
RPS2	23	5668	4410	4719	3368	6329	1478	23918
ST6GAL1	25	27758	45994	12409	8543	29955	4103	223056
DSP	25	74279	46536	61206	50962	77187	23491	250041
CBR1	25	181219	164220	106694	63581	283822	8393	671183
ACADS	25	7062	6481	5128	3531	7559	1705	31972
NCK1	24	13652	25978	6854	4746	8888	2040	126221
H1-5	24	10444	12645	6145	3273	10972	954	56446
H1-3	25	38462	99313	12466	8511	21894	3383	502178
H1-2	25	9014	5931	8283	5875	11166	1550	28365
EPCAM	21	4056	3391	3504	2127	5216	311	16321
POR	25	34532	64739	19629	12379	26781	6194	335626
PLCG2	25	119710	210659	56584	48908	79691	31777	998428
FAH	23	5078	2818	4486	3322	6071	809	12310
NAGA	22	7364	11486	4158	2456	5654	1942	55376
HSPA6	24	13478	6234	11113	9810	14841	7191	30379
GOT1	24	7576	3959	6824	4665	8068	2748	18834

PG.Genes	n	mean	sd	median	q1	q3	min	max
BPI	20	8711	5825	7298	4707	10979	1512	25624
PRKCA	21	2934	1675	2662	1506	3834	1003	6840
JUND	24	9317	15293	5390	3099	8522	1614	75780
CAPN2	25	12256	8224	11406	6446	15159	2920	39359
DES	25	17043	19798	6924	4221	20347	1741	66484
CTPS1	25	13196	13388	10376	8028	12267	3993	71133
DDX5	23	24035	21617	16931	10786	30759	4742	108015
PFKL	25	29100	20504	21602	16567	32469	8856	81567
GM2A	20	4303	4347	2400	1947	4558	1041	18908
CR1	25	10675	4192	9066	6848	13443	5151	18112
LGALS3	24	26593	27879	17498	10952	30161	5130	129640
FLT1	25	21413	45672	9530	6127	16253	2319	235485
PSMC3	25	7460	4222	5234	4447	9991	3116	17473
TCP1	25	58240	47213	45259	27767	70588	11978	243520
PTPN1	25	14802	14470	10858	8650	13594	5017	79042
IGFBP2	21	6325	8797	2349	1558	3945	820	32039
RPL35A	22	17728	18642	10889	5424	21164	2168	68520
ITGB5	24	6159	5110	4474	2196	9029	894	19324
ARF4	25	5914	3911	5338	3725	7283	651	19790
RPL7	17	2291	1121	2125	1676	3031	414	5234
VCL	25	63388	65472	35997	13767	89943	5112	217036
LBP	23	4204	3178	3178	2372	4912	975	14200
NAT1	21	8261	6761	7261	3961	10406	637	28771
PGAM1	24	13714	10073	12288	5662	17506	3113	41656
XRCC1	25	13940	13559	11135	3296	14913	810	61547
TNNI1	22	2837	2054	2257	1553	3628	434	9777

PG.Genes	n	mean	sd	median	q1	q3	min	max
NCL	25	15845	14625	11867	6038	19550	1912	63989
POLR2E	22	27142	13231	21972	18598	35246	11783	67040
TNFRSF1A	24	9909	18146	5781	4431	9067	1684	94264
TRIM21	20	27699	27719	21229	14749	30085	2016	135576
EIF2AK2	25	13361	12736	7698	5729	22732	846	54928
SRM	19	9394	8218	4897	2763	15974	1552	25388
ORM2	25	339936	680445	201368	150510	277165	85107	3585640
CSNK2A2	19	3175	1792	3316	1712	4222	476	7587
ITIH2	25	41498	23706	30510	25249	55511	15488	114918
ITIH1	25	24429	12467	21996	14077	32626	8679	54091
CEL	25	12746	7762	9861	8564	14114	6041	38996
NFKB1	25	3697	1543	3540	2733	4593	1318	6628
NCF2	25	12975	7605	11089	8665	13560	3762	42060
TYMP	24	16425	41545	4880	3681	8556	1728	207594
EIF2S2	22	5897	4255	5498	2998	7662	1074	19806
ANXA7	25	5912	4934	4506	2641	7341	1809	22832
AZU1	18	6395	10388	3901	2519	5445	892	46924
TPSB2;TPSAB1	25	21551	16954	17813	10012	28047	3177	84794
BTF3	23	4163	2939	3426	1854	5518	1148	13408
RAB6A	24	4172	3071	2802	2084	5834	1163	11661
MX1	25	117290	209962	49849	35553	96452	22552	1076219
PSMB1	25	9250	5063	8730	5617	11627	2671	23292
COX5A	19	3535	1398	3563	2291	4328	1223	6250
LMNB1	25	51853	37985	39251	32916	55864	17563	179546
ITGAX	25	69499	36813	54451	42607	83103	17755	165694
GZMH	17	8728	11573	5972	3750	8568	794	51248

PG.Genes	n	mean	sd	median	q1	q3	min	max
PZP	17	3449	5560	2041	1201	2309	463	23581
OGN	24	14010	16926	7277	4274	14114	1713	60483
CAST	25	17154	38169	6286	5209	10212	3696	194159
EFNA1	15	6536	8691	2356	1184	8652	536	26430
AGA	15	2648	1355	2188	1729	3122	1099	5663
RASA1	25	23545	20079	19682	11826	27428	2899	98931
PTMS	24	19953	16863	14495	8310	30760	1285	61611
PTN	25	235169	501034	63648	40494	170648	7300	2476275
GSTM3	25	56435	68149	23714	17874	55963	11220	285876
ATP6V1B2	25	4112	2276	3840	2304	5412	665	8360
ATP6V1C1	23	7546	9780	3793	2588	9286	430	46461
CSRP1	24	102466	149533	25109	4150	113008	1122	450948
ACO1	25	7343	10407	5439	3440	7343	1022	54939
S1PR1	24	65395	92745	35058	19464	69119	8385	432604
NT5E	23	33674	113027	4789	3033	8785	1032	546825
EPHA1	18	4456	5350	2535	1740	4642	1076	22325
VDAC1	23	12573	9822	10220	4801	15900	2124	34601
BGN	24	6186	7946	3852	2116	5986	1462	38293
CD9	17	18516	34854	2695	1730	12543	912	136000
BCKDHB	25	14770	9101	12627	9261	19446	2078	41500
COMT	15	2962	1726	2395	1802	3614	1377	8288
TGM2	25	24576	27172	17016	6818	27718	2721	106869
OSBP	24	31488	56910	17044	10834	27758	1573	290529
PCMT1	24	7518	3874	6272	5113	8749	2946	16192
FBL	25	37219	27668	30844	23401	39185	10908	145060
GART	25	7058	4666	5486	3153	10141	2281	18296

PG.Genes	n	mean	sd	median	q1	q3	min	max
PAICS	24	6593	4128	4656	3729	8702	2821	17915
GPX3	24	21292	28309	11279	7458	21512	4168	142322
NME2	25	48497	29243	41147	28606	61025	4189	147305
ENPP1	25	62175	30835	55936	42269	81065	16315	172925
SPRR1B	25	109342	85829	78737	56497	131793	34344	431223
HNRNPA2B1	24	35633	34110	27137	13088	44903	2935	163382
RFX1	25	35912	30135	29823	19934	38934	2268	153093
CBL	24	80907	175634	26100	16727	47038	3138	829716
IGFBP4	22	3130	2062	2397	1465	4431	872	7128
UQCRC2	23	13560	17477	5774	3873	15146	117	67175
CPN2	25	8472	4982	6133	4896	11315	1974	20636
MMP8	20	4088	5473	1849	1288	3508	458	20544
FBLN1	24	50178	61016	26961	19317	41250	10974	265617
FBLN1	24	15285	21295	7563	4187	14510	1397	97225
TCEA1	22	3169	1949	2625	1526	4365	801	7926
SFPQ	25	37128	28164	28184	17252	47111	9758	116485
PPIB	24	27569	21657	18312	10688	46177	2034	78176
S100A1	19	15084	16356	10311	4287	16212	1067	67955
HRC	23	7245	8135	5401	2378	7992	304	35927
ME2	20	3635	2128	3175	2109	4533	88	8060
WARS1	23	3442	1507	2917	2414	4425	975	6733
RPS3	23	8145	6811	5138	2974	10480	2158	27178
GCSH	15	2625	1496	2511	1491	3398	627	5659
SP100	25	40543	67671	21577	17807	32952	7448	351521
AHCY	24	22101	15786	18988	12448	24463	3121	79699
CFL1	25	116940	87018	99795	44400	131180	21185	334364

PG.Genes	n	mean	sd	median	q1	q3	min	max
ITPKA	24	8076	11980	5609	3731	7239	2640	63168
PAX7	25	13185	14096	8974	6467	13286	2065	71116
CPT2	15	3364	2877	2360	1816	4552	416	12328
DTYMK	24	40904	85919	5653	3682	17709	393	347617
RRM1	21	2543	1941	1785	1097	3207	378	7587
CMA1	24	157781	316473	82135	47605	133683	29752	1618458
LAMA2	25	48454	44510	35330	25546	46758	19882	216323
PRTN3	17	1961	1417	1348	973	2244	442	5271
MMP11	18	4620	6480	2084	1451	3761	985	25780
CHM	24	13333	11146	9759	6000	17042	1136	45339
EEF1B2	23	11934	11730	7774	3142	17280	11	51504
ACP1	24	19661	8109	16934	14044	24590	6538	38404
ACAT1	23	152925	115091	106741	86247	207132	28385	526250
TNC	25	13656	18110	6484	3743	13126	1907	79184
POLR2A	25	17303	29202	10653	7434	13839	5660	154344
CDK2	21	10809	20654	5723	3556	9302	1463	99584
GRK2	21	5382	3687	4683	3098	6605	1303	17148
AZGP1	25	41750	53991	27838	23713	35841	14126	291608
MPST	24	16547	11866	14282	9677	18730	4836	61051
LAMA1	25	101155	62948	84692	61242	116443	35023	325134
RPS12	24	9969	7708	6300	3833	16043	1880	25299
YY1	25	30597	31273	19467	13352	37949	577	147116
DNAJB1	25	80242	51884	66598	45005	109268	13019	264254
DNAJB2	25	433163	410836	342298	223985	460729	101515	2233316
ATP5F1A	24	7588	6724	4433	3319	9886	1447	23016
PSMA2	24	6970	3921	5995	4148	7687	2480	17774

PG.Genes	n	mean	sd	median	q1	q3	min	max
PSMA3	25	9413	4488	7695	5889	11088	4605	19421
PSMA4	23	15983	8856	12032	9594	20933	7130	35734
S100P	25	14150	17691	10485	6097	16069	2696	93673
COL5A3	25	2980	1133	2776	2237	3312	1382	6640
PTX3	24	16483	12983	12809	10130	19062	5597	69213
MSN	25	40629	35288	26891	17409	57404	8196	135809
DDX6	25	1013250	441793	904108	760310	1226046	476073	2668563
U2AF2	22	13867	21027	7400	4315	10115	1355	95863
RPL13	19	4122	3991	3050	1417	5580	224	16279
CHML	25	38819	40997	29313	23267	41467	12553	228121
IVD	25	217757	157969	171891	137383	218243	39711	724609
S100A4	25	26368	13276	23447	16299	34866	8588	68485
MGAT1	18	2516	2067	1840	1092	3370	385	7451
HMGB2	23	7530	5947	5544	2269	11749	757	21260
PTBP1	25	25655	19719	22552	13435	30750	7568	106549
VARS1	24	13731	9530	10319	7364	16562	4000	43954
EEF1G	25	18867	20004	11204	6747	27206	2568	98330
FKBP2	22	12709	10480	9795	4034	18192	1160	33610
MST1	21	8384	11661	4680	3040	6888	1125	56086
ACVR2A	25	1557823	2839802	859956	572370	1488209	30543	14802987
STOM	24	45532	55288	28267	16423	53544	514	263041
PON1	24	13098	10941	8985	4700	16309	2513	42429
YWHAQ	24	27270	21901	21160	10158	34657	2370	81056
MAPK3	25	23957	19995	19855	13365	31549	4288	101436
CALML3	25	210714	210187	121485	86251	248321	49588	903623
DPP4	25	17338	11071	15754	10828	22313	630	45080

PG.Genes	n	mean	sd	median	q1	q3	min	max
RPL10	24	72052	127558	27697	20652	80526	6964	644747
RPA1	25	19897	11040	16366	12747	28033	8008	55704
APEX1	24	24799	20479	16972	8359	40515	2074	59593
DCK	24	20187	20897	14222	4063	24471	836	73190
CAD	25	17648	14914	15261	11546	17970	8678	87561
CALR	25	51112	35810	35716	24978	72706	7135	131018
MAP4	18	5890	5553	4347	1832	8270	571	23852
CFP	25	9054	8599	6101	4419	9779	2150	40951
ITPKB	25	28210	23762	23609	14268	32378	10999	126374
PSMA5	24	25894	11380	23093	17098	32049	11884	56145
PSMA5	25	132037	62397	108435	92255	147897	66689	323724
HLA-DMB	19	3218	3390	1996	1214	4389	209	14093
PSMB4	23	6761	3919	5325	3775	7683	2772	15447
PSMB6	24	8338	5398	6742	4806	9356	3656	23838
GSTM2	24	14086	9208	9830	7850	19104	5109	35728
TMOD1	24	2740	4534	1509	1052	2227	204	22865
TEAD1	20	1432	2347	538	382	1115	96	10098
MAPK1	25	14773	17992	9334	7169	13251	4250	76846
GCA	24	7002	16600	3435	2231	4069	1355	84400
ERCC5	25	17376	11665	11586	8939	23280	3896	45522
PTPRM	25	998109	596846	956996	594780	1199645	161313	2670893
GTF2E1	25	3210	1022	2968	2702	3499	1390	5979
GTF2E2	21	2426	1457	2442	1243	3444	234	5638
PCSK1	25	17354	17608	13031	7306	17407	3677	77431
TPP2	24	7845	24865	2250	1739	3492	1035	124333
IMPA1	23	6082	5821	3533	1351	8779	1097	21050

PG.Genes	n	mean	sd	median	q1	q3	min	max
EPHA2	25	58052	93888	37604	25207	47706	8904	497770
EPHA3	23	3264	4735	2161	1142	3012	225	21486
CRABP2	23	9041	7416	6224	3508	11968	953	27919
ARID4A	25	4721	1569	4216	3829	5834	2263	8409
CASP1	17	1269	658	1315	828	1461	356	2615
SERPINB3	25	91173	126278	64065	35981	80343	6056	651224
LMOD1	25	4595	2796	3934	3070	4838	1584	13807
SERPINA4	25	24309	45277	14600	8422	20096	3229	238127
EEF1D	21	5766	4421	6315	2697	7707	740	19790
EEF1D	21	6385	4907	4756	2051	10780	682	17779
MARCKS	24	5513	4878	3433	1985	9106	721	16682
ALDH4A1	23	3716	1913	3389	2262	4203	1992	10544
PBLD	25	53222	36894	44351	33689	63404	7675	184402
ERP29	23	5087	3065	4128	3081	5625	1704	12172
PRDX6	25	144857	87899	128455	100079	153315	7044	378523
BLVRB	24	47128	31948	31051	23940	70961	6719	104441
DDT	24	18606	17079	12852	9029	22126	4422	86365
PRDX3	22	13228	14143	7355	4602	18936	200	57350
RPL12	23	10325	10222	7551	3628	10680	2150	44931
ECHS1	22	8116	4984	6082	4700	11109	2917	21129
CMPK1	23	15150	11898	10138	5333	23310	3584	42815
PEBP1	25	61336	47502	52192	21740	93865	4453	175297
PDIA3	25	29203	21010	22493	13148	46823	1932	88788
PPP2R1A	25	23510	14085	22247	13512	27069	6090	65166
CDC27	25	565707	600722	363363	164883	855554	47576	2852532
NKTR	25	206371	167830	155974	123061	206137	44667	860946

PG.Genes	n	mean	sd	median	q1	q3	min	max
NMT1	23	4913	2868	4584	2328	7045	1388	9944
ADSS2	24	52446	24551	48412	32819	64139	15413	115968
LRPAP1	24	90055	178787	30199	17643	57802	6929	730221
ADSL	24	4235	1643	4006	3204	4892	1490	9334
ADSL	24	4559	7097	2944	2513	3210	1261	36877
CLIP1	25	28214	20353	22262	14981	34072	5123	92883
GSTT1	23	6189	5395	4548	2601	8103	467	21456
SERPINB1	25	10307	5420	10442	6867	12777	2369	24346
ALDH3A1	23	27743	42469	11889	6262	30163	956	193953
POLR2B	25	28316	16768	23245	19236	29979	10261	77753
SDHA	25	164890	54307	154138	124453	209705	90155	289918
CORO1A	25	20338	41896	8651	4883	12213	3487	208658
GDI1	24	11107	8259	8731	3820	17041	1332	27226
S100A7	24	62756	31856	55950	39480	79719	24909	143653
MAT2A	24	5814	3647	5187	2641	7441	1500	13960
PRKAR1B	25	35052	44834	26430	15436	36138	4311	237744
PRKAR2B	23	7480	13540	3994	2872	5873	808	67706
CPS1	25	6832	4219	5268	4238	8364	2673	22429
HIVEP2	25	24432	16834	21434	15992	24988	13055	100676
DNAJA1	24	46210	33383	40466	24873	53367	5284	159020
AKT1	25	27444	42617	15559	10663	18262	5740	205243
UQCRC1	22	6828	8772	5086	3131	6472	1378	44673
HIBADH	22	4118	1708	3939	2813	5261	1076	7489
ATIC	25	16851	29089	9848	6444	16998	934	152906
HNRNPH3	19	5709	3826	5136	2366	7986	1615	15793
HNRNPH1	24	32426	48795	16087	10930	28035	2634	237144

PG.Genes	n	mean	sd	median	q1	q3	min	max
CASP14	21	6265	7614	3207	2546	6384	933	31872
SFN	16	3621	1795	3434	2436	4159	984	7421
STIP1	25	67281	39598	57244	37852	74404	25772	202328
S100A11	25	53731	39102	47092	26453	66233	9660	176969
CEACAM8	20	23978	27197	17602	12083	24576	3679	131714
PRDX2	25	260125	168274	209855	120080	393684	19216	577275
DCTD	22	6198	4951	5013	2702	8179	815	20994
GBP1	25	20281	26727	15010	8951	17959	5347	134181
GBP2	25	3543	1628	3447	2014	4384	1162	7420
RPL9	21	2880	1889	2789	1313	3577	616	8749
KIF5B	24	3815	2630	3185	2863	4057	1194	15155
CSTF2	23	25579	78721	6941	5863	10653	2500	385827
DUT	22	2647	2188	2233	877	3806	503	9080
S100A3	23	30969	26906	26423	15614	33604	10544	141270
MCM5	25	20664	9690	19590	13762	24075	7361	43389
GALNS	22	5198	5182	3630	1876	6752	750	22570
RNASE4	15	2162	1725	2008	872	2700	444	7262
NTF4	17	955	430	1112	568	1290	241	1612
SHMT2	16	1229	566	1207	859	1544	335	2209
EVI2B	25	62431	165652	20245	10989	33551	5432	838988
HSPA4	25	102567	50089	97127	65886	126114	47040	221996
GRK5	18	9976	7517	7625	3475	16456	1905	24242
MPI	24	2079	836	1900	1453	2429	885	3943
GPC1	24	72730	68145	40018	28421	103231	4295	280386
PFN2	25	971304	1192236	484816	398279	790525	53225	5308038
CTNNB1	25	294778	374872	115529	75886	258245	38313	1386168

PG.Genes	n	mean	sd	median	q1	q3	min	max
NOS2	25	71492	59662	57813	39416	81985	23808	331545
SERPINB6	25	20802	31232	11554	6550	18683	3843	137077
RPA3	18	3193	2005	3333	1475	4537	573	7821
RPL22	23	8599	6705	7707	3867	11116	596	29247
GTF2F1	23	19033	47679	7421	5622	12847	2847	236450
SPR	25	17595	15570	13509	10532	19003	4343	85977
THBS2	25	8399	8581	6106	3982	8211	523	43943
THBS4	20	2209	1142	1976	1315	3074	509	4121
HOXD13	23	13307	19624	7099	5210	11976	2286	96590
IDUA	25	13347	10175	11022	7342	15068	3418	43615
SAA4	25	7447	4763	6394	4925	9316	1367	25393
FBN1	25	17560	19951	12151	8896	16580	6490	107355
PCK1	25	9523	7439	8192	4486	11336	1097	27840
AGL	25	7495	3748	6989	5354	8244	1720	16605
MYH9	25	338522	328171	230917	195130	377943	81451	1644359
COPB2	25	29463	27398	20822	17367	28664	11359	147417
ADD2	23	5383	9977	3072	2180	4336	1379	50661
GRK3	25	316173	420794	183864	117160	338279	9285	2133700
FUS	24	11056	11499	8029	5526	10887	1993	57894
DEK	23	3700	2824	2889	1278	5711	226	8553
GLRX	22	3699	2228	3215	2347	4390	996	10945
HMGCL	20	4592	9214	1892	1529	2058	690	41024
PSMC2	20	1842	885	1605	1282	2413	680	3777
PSMC2	23	9056	12787	4389	3543	8769	1414	64299
CHI3L1	24	9950	16013	4334	3494	6990	1792	72481
ARL2	18	3694	6446	1799	1387	2341	350	27317

PG.Genes	n	mean	sd	median	q1	q3	min	max
ARL3	22	4959	4158	3590	1319	8578	424	11960
TRIM23	23	13401	6605	12755	8622	18278	4712	27697
MAP2K2	25	19470	9989	15660	10376	26826	6805	47085
ATP6V1E1	22	6446	4834	5064	3196	8113	750	19477
CPOX	25	46260	53547	30286	25417	39936	13612	264402
RPL4	22	5352	3962	3664	2438	7434	1560	16762
PGM1	24	12827	9715	11406	3678	18015	1504	36517
GNL1	17	2442	1298	1855	1727	3761	303	4661
SERPINB5	25	44989	62859	30931	21766	42848	6836	327927
SERPINF1	23	28481	28321	16958	13414	32189	8418	141549
DLST	24	7188	3539	6121	4784	9465	3045	16231
GMPR	23	14767	43114	2540	1720	3283	383	203532
CFHR2	24	6356	11563	3875	2760	4367	1127	59737
SRP14	19	6833	6251	4512	2657	10503	894	24823
TGFBR2	25	64672	112603	39095	27291	58908	12915	596831
HPCAL1	25	173097	387845	86798	56868	103418	39577	2009815
TALDO1	25	47358	29187	36243	20907	70362	12155	111276
SNCA	25	29013	29367	20280	15748	33303	6915	146102
COIL	22	16151	16040	12510	7441	15934	2610	71568
HSPA9	24	13506	8717	10313	8116	18177	5948	45828
EIF4A3	23	5873	12863	2053	1420	5551	760	63712
RPS19	25	266274	355195	191320	128628	282116	3851	1881455
RPL3	22	6143	4726	3952	2379	8393	1425	16721
COL15A1	25	188277	256663	135503	76135	201433	53641	1376322
ANP32A	23	21181	19810	12303	5610	32449	3067	71767
FEN1	24	13181	8226	12227	7885	15031	1670	39420

PG.Genes	n	mean	sd	median	q1	q3	min	max
CUX1	25	39738	46793	32665	21255	37506	9176	253480
MMP12	25	34200	12897	33020	29036	37919	14694	85196
CAPG	24	9068	7829	6333	3349	10595	1805	33453
CAP2	24	20063	23651	10977	6867	26498	4924	120726
CD96	25	27221	13767	24795	17940	35293	2649	52714
TXLNA	25	9036	6559	8097	5093	9036	2649	33637
CCT6A	25	8580	13539	4200	3093	7983	1907	66102
NNMT	19	19846	30479	5139	2057	18677	615	99308
RPL13A	16	1825	1261	1380	1022	2473	385	4961
STAT3	23	7654	3048	7638	5934	8513	1610	16327
USP8	25	178765	127933	161050	83355	212006	47159	570906
PEX19	23	10193	8155	6422	5433	13089	386	36437
MDH2	24	20314	14264	18070	8393	32284	1200	55194
HADHA	23	2542	1720	1953	1284	3790	396	6036
EIF2S3	25	2986	1996	2539	1379	4064	717	8757
OPRK1	23	15046	10733	12185	7685	18078	4326	45689
ETV6	24	6479	7018	4038	2955	6801	620	33944
EIF2D	23	6493	6515	4591	3446	6095	1996	32347
MNDA	24	17761	8411	16092	12077	20615	5361	38629
RGS2	16	16944	18910	11595	3605	20478	859	74208
PTGDS	23	9383	10407	6948	5725	7807	3117	54991
UBA7	23	6216	20605	2144	1073	2748	20	100592
NAA10	15	1514	929	1281	933	1914	441	4020
PPP1R2	20	3870	2495	3712	2481	4338	1516	13449
CSK	25	30139	23354	26360	17974	31591	14228	132724
GARS1	25	23361	45783	4295	2739	11080	825	187742

PG.Genes	n	mean	sd	median	q1	q3	min	max
IARS1	21	1844	1064	1647	1243	2564	320	3920
MAP3K8	24	42945	26531	38496	28452	51463	10582	143881
EIF1	24	2991	2810	2027	668	4625	376	8886
PRKCI	23	40596	31162	37124	13902	54464	2741	110500
ACTR1B	25	15097	8136	11568	9125	18156	6060	36064
TMPO	25	22857	39656	11984	8984	19012	5390	207831
STAT1	25	6133	3929	4510	3861	7035	2367	17137
STAT6	25	7798	9872	5702	3739	8932	734	53090
STAT5A	23	10188	7680	9919	5376	11320	2782	39719
MTREX	23	5422	3662	4333	3551	6032	2274	20412
AKR1C3	25	19142	16297	11879	9496	26409	3600	73273
EPS15	25	61077	22947	57121	47062	64722	19435	122819
CASP3	25	9226	7446	7876	6302	9354	823	42214
TEC	25	18591	30552	9189	7393	11358	2704	126232
NCAPD3	25	96820	170992	49864	37329	69390	16861	877162
RBM34	25	200678	136472	194952	117146	255703	4713	657298
LIFR	25	103600	109316	83441	56829	103692	30199	591760
LRPPRC	25	14060	6598	13770	8667	15485	6818	35391
CDKN2C	21	3333	3905	1884	1498	4068	341	18944
PRCP	19	2622	2204	2167	1153	3016	283	9467
HTT	25	153560	255874	85081	60782	125114	45499	1316489
PAFAH1B1	24	5820	4932	4396	2931	6931	2336	25599
PTGFR	20	19360	16405	14194	9343	23646	1921	61143
MCAM	25	52931	56006	21219	12038	74580	5710	177270
CRAT	25	13079	6188	10674	8850	16595	6909	31077
MATR3	25	12386	18771	7880	6102	9626	4752	98649

PG.Genes	n	mean	sd	median	q1	q3	min	max
ZAP70	24	7423	4149	6463	4797	8974	1472	21018
SYK	25	232988	278755	166602	114392	229935	76879	1518614
NAMPT	22	4808	4949	2754	1893	4172	904	16436
AFM	25	30397	15739	31968	17686	37649	10072	84700
PSMC4	24	9671	4685	8796	6190	11778	3061	20578
PPIC	16	2565	1663	2063	1504	3449	195	5536
CBX5	18	2201	1525	1733	1062	3509	191	5286
RANGAP1	23	2731	1525	2064	1753	3876	904	6401
RECQL	24	2742	2416	1603	1116	3441	587	9428
GPR4	20	5936	6052	4257	2087	6508	1058	21178
CRK	23	7973	5673	6322	3781	10398	2015	24879
CRKL	23	3260	3432	2175	1313	3710	744	16973
BAG6	24	3103	2272	2610	1811	3476	72	10366
GSTM5	23	7857	7164	5469	3089	8497	544	25979
NSF	25	8325	5373	5961	4265	10838	2540	25622
CDKN1B	25	50049	132097	16723	12489	29755	4066	670883
RPL5	23	11389	11223	8007	5811	12542	3796	57439
RPS9	25	5224	7272	3634	1986	4808	587	36991
RPS5	21	2288	945	2080	1686	2776	963	5175
MAP1B	24	12653	15271	9743	6691	11198	3437	78825
GNPDA1	25	23201	66407	7525	4420	13579	1964	339332
IQGAP1	25	16403	14704	11325	5685	21707	2927	64865
HAAO	24	9416	5010	7098	6065	10927	4516	22566
GYG1	19	4527	3922	2812	2272	5240	833	16855
RABIF	17	1044	781	841	496	1195	233	3107
PLA2G4A	21	2430	1496	1831	1339	3752	630	5719

PG.Genes	n	mean	sd	median	q1	q3	min	max
RAP1GAP	25	2643	1405	2252	1838	2996	914	6496
CAPZA2	23	21687	19935	17661	8665	25066	2985	89657
CAPZB	19	5525	6198	3140	1134	8174	312	21289
CAPZB	23	12863	10661	9895	5790	15906	3111	47925
EIF1AX	22	3195	2610	2062	1103	5279	570	10512
ALDH1A3	25	17227	24007	10350	6578	14772	5391	121756
QARS1	23	7509	7316	5740	3879	9746	1426	38175
HTR5A	22	14144	19421	5097	1985	11840	717	59757
CDX1	25	108291	241578	45548	31603	77945	2727	1246641
RPL29	23	8216	5511	6328	4043	11224	1724	22029
XDH	25	299853	382199	190250	171964	235448	96805	2069933
ATP5PO	25	9327	15052	4542	2098	6193	1066	72157
GRIA4	25	62969	115419	23411	16352	53438	6671	483339
LIMS1	16	5571	4610	3729	1839	10550	798	14293
PREP	23	6448	4783	4711	2924	8128	1667	20054
ME1	22	8693	10994	5958	3508	8676	1817	54026
IREB2	25	18947	13956	14045	12634	18722	5719	62488
RFX5	25	20962	16930	17158	10604	28501	1293	65014
ARCN1	25	13397	7370	10422	8063	16102	4612	35722
GCLC	24	8060	4866	6686	5720	9543	2523	27816
GCLM	24	7199	6783	4694	4265	7046	2443	33239
PCP4	17	8310	8188	5292	1964	13175	279	26365
NRIP1	25	33211	21540	28211	17389	44124	642	83410
PSMD8	23	2828	1275	2965	1589	3938	1179	4833
PRRC2A	25	21545	8485	20192	15286	26093	9137	41354
GSS	25	9943	4589	8938	5942	13137	2488	19212

PG.Genes	n	mean	sd	median	q1	q3	min	max
CCT5	25	10598	5740	9085	6327	14063	3298	29449
NES	24	4784	2165	4587	3190	5734	1636	11415
HSPA13	23	76945	46409	60747	48419	82076	30456	201511
IDH2	24	20845	18646	15061	10904	26124	7138	99220
LHX1	23	4099	6681	2661	1424	3310	726	33562
CCN3	25	34182	49048	20617	14649	32002	7540	247683
MARCKSL1	22	3431	1867	3255	2409	4208	478	8153
ALDH9A1	25	12812	8535	11093	7286	17562	2755	44135
RPL34	19	3695	1581	3356	2310	4789	1697	7109
RPIA	24	4660	6109	3710	2484	4527	770	32438
NASP	25	13514	14909	9023	6906	13898	3328	76103
FASN	25	43406	13556	39974	33657	49146	28128	77112
FNTA	20	2513	1872	1799	856	4255	425	6501
CCT3	25	16554	20677	9392	6984	17455	3858	98913
TUFM	21	10027	16029	5397	3174	9074	1781	76726
SRP9	21	5373	5104	3953	956	8269	369	17666
AARS1	25	11257	16186	6538	4755	8553	3554	74505
SARS1	24	5858	4336	4776	2587	7875	803	16563
PPM1F	25	7237	5434	4990	2946	11758	701	19143
PRIM1	25	6420221	3733725	5917376	3971865	8149112	1857817	15961835
PSMB3	23	5243	2423	4734	3325	6164	2389	12502
PSMB2	25	56627	63444	38874	32784	50647	21553	346366
MCM2	25	7486	9933	4993	3562	6409	1874	42348
COMP	25	19075	32763	9010	6026	12235	3625	161273
ACADVL	25	110143	142677	78186	49440	100934	22388	742337
YLPM1	25	78489	75363	58618	43388	93596	12826	356589

PG.Genes	n	mean	sd	median	q1	q3	min	max
ACOT2	25	21346	22140	16575	9196	23664	6966	119567
TMED10	23	15753	7832	14379	9580	19540	5102	32984
RBM25	25	21730	10439	19099	15534	26117	8290	55877
NUMB	25	34377	34985	21093	17722	37975	12037	145806
HINT1	24	13219	8114	11170	6773	19157	3357	29125
NUP153	25	35573	36542	24924	21494	31681	16705	190258
RANBP2	25	386814	302605	315015	226276	393582	140297	1354932
GSK3A	25	56106	33497	43904	36380	65044	18969	154343
GZMK	25	69440	154596	35268	20149	52386	9435	801892
SEPHS1	19	4732	4931	4081	1458	5800	302	19573
SELENOP	20	2760	1537	2343	1479	4092	711	5890
CAMP	22	3878	3503	3468	1634	4606	391	13579
GMPS	24	9088	16107	5213	3266	7048	2196	82494
LIG4	25	11580	27523	3106	1683	4903	644	134769
HNMT	23	3585	2594	2977	1804	4818	800	11748
GNAQ	25	38491	51344	24022	18890	31708	12140	257159
MEOX2	25	45947	35622	28858	22688	59991	5827	124445
SULT1A1	21	32316	73143	13912	10493	18748	7473	348637
CRIP1	24	25337	17039	20868	12489	34443	5506	58918
MMP14	24	38938	31996	30607	25173	42923	1346	162943
GDI2	25	29160	16198	25476	15258	41390	10038	71807
SERPINB8	25	12865	8215	10005	7667	16478	3630	35422
SERPINB9	25	18748	25775	10953	7503	16830	4671	119333
SERPINH1	25	33336	15944	32641	16784	43808	13753	70604
LHX2	23	5724	3567	4584	3675	5865	2124	15872
PDLIM4	25	46501	72785	25761	17725	38911	9493	372141

PG.Genes	n	mean	sd	median	q1	q3	min	max
ST13	24	24714	14117	20471	15615	28985	9471	71174
ERF	24	19248	39527	11539	4014	15048	2100	201966
VASP	18	3450	3126	2773	1389	3953	350	13101
METAP2	25	63882	104015	24698	15175	37163	1872	428713
METAP2	24	13133	11648	10102	4046	16885	968	38293
NUDT2	16	3304	2503	2494	1725	3946	572	10366
TNFSF10	25	52171	39271	37537	32427	55473	11229	177294
HLCS	22	28421	27047	23352	15420	30001	10535	144241
RASSF2	17	3766	10235	1422	884	1734	232	43407
LRBA	19	16586	13917	12611	5567	27231	2266	41339
LRBA	25	9518	6055	7761	6444	9122	5016	34752
BCAM	15	6433	9572	1838	1437	4520	512	29345
PPT1	25	13854	8536	12252	8966	15542	4984	48130
RPL14	25	21195	16390	16837	11222	22242	4060	65725
ANXA11	25	24569	9592	25471	16446	31878	10620	43734
FXR2	24	1908734	1033361	1706370	1271200	2110091	631781	4831378
RAB5C	25	65044	22021	64293	50665	78987	21949	117908
RAB7A	25	10925	12460	8714	4377	14125	1866	65058
RAB13	25	14136	11617	11937	9771	14646	5698	66880
PDE6C	25	23149	37549	10557	6187	20753	3567	187425
SCNN1G	23	47907	130759	3781	1830	9358	757	557096
DAP	24	3106950	3778057	967661	229836	5541432	46822	11425679
DUSP3	19	6368	7406	3768	1293	8777	625	24255
SMARCA2	25	8406	15062	4351	3174	6982	1361	76475
TPMT	25	4112	2370	4147	2363	5299	1039	10615
RENBP	23	10151	14029	3451	2303	8979	1495	56609

PG.Genes	n	mean	sd	median	q1	q3	min	max
MECP2	25	6486	8773	3929	3424	5371	1729	46217
IRAK1	25	299454	143152	272396	211751	327440	117247	719880
ALDH5A1	24	30324	104150	8299	6908	12748	918	518666
HSD17B4	24	2847	4052	1948	1238	2463	613	20656
PSMD7	22	2845	1341	2478	2011	3834	704	5566
SGSH	21	9596	5674	8663	5901	11116	1041	25550
STAT5B	25	7278	6405	5699	3432	8376	308	29557
USP11	25	133983	296098	58683	41074	82106	30326	1491511
KCNQ1	25	17929	15155	12221	9822	17479	3557	58459
PLXNA3	25	4595	3795	3687	2397	5134	1241	19835
DYNLT3	15	1221	632	1185	740	1493	397	2476
GUCY2F	25	10457	4963	9457	7077	11313	4673	22465
HDGF	25	12106	9467	8664	6040	13424	2535	38516
LUM	25	87666	114548	35295	17525	111824	7403	501358
PRELP	25	19910	13842	14085	11445	27818	2171	64108
CNN1	25	45676	52579	21946	11701	79168	4258	221696
CCNH	25	26280	12806	23326	19456	31837	7119	56327
NDUFA8	19	4951	4472	2893	1747	7572	516	13519
HNRNPA3	18	36319	28132	31614	14772	52383	1468	104675
HNRNPA3	25	12555	21673	5992	4627	9866	2327	95359
HNRNPM	25	22717	64806	9199	5648	14422	2194	332781
KPNA1	22	10071	11478	6397	3188	9985	992	42787
DGKE	20	2075	1864	1517	1068	2490	355	8635
POLR2H	19	2167	3683	1313	863	1515	2	16793
MAP2K6	24	9584	5073	7657	6905	10636	5135	27683
ARHGDIA	24	20768	13063	19267	9294	28962	5435	46495

PG.Genes	n	mean	sd	median	q1	q3	min	max
ARHGDIB	24	30839	16766	26620	17699	40444	9500	64970
AGFG1	20	2558	1006	2412	1750	3479	1162	4431
HNRNPF	24	5318	4269	4319	2327	6235	750	17687
GTF2A1	23	18037	26862	10520	8102	19796	119	137243
ZNF140	18	2387	1454	2421	1215	3103	242	4816
ZNF131	23	11828	12085	8158	5952	11277	3325	48581
ZNF142	25	66028	53817	48779	40333	69913	29127	286459
RIDA	15	1397	981	1477	452	1874	302	3829
SMS	25	5857	3162	4960	3095	7709	2069	13382
HK3	25	19152	10521	15309	11957	24295	7974	43519
STC1	18	4138	2795	3160	2264	4937	1420	12645
NDST1	25	205998	357684	109951	75098	186161	38486	1860299
THOP1	23	2415	2667	1934	798	2869	433	12514
AKR1C2	18	4956	2557	4288	3663	5566	1620	12354
CAPZA1	25	9355	9928	6658	3847	8511	2362	41939
CRIP2	15	9825	7727	7285	3659	15341	1162	24065
PDX1	23	2124	1639	1682	962	2457	323	6108
BLVRA	24	14318	6052	14832	9741	18089	3277	24164
PPP5C	25	19251	14790	14681	11840	16961	7376	76346
ARFIP1	25	55272	29113	51124	38195	60368	27212	168470
NUBP1	18	3312	3487	2308	1630	3216	536	15581
ACLY	25	19932	12275	15713	13037	24008	2926	51701
METAP1	25	15967	30513	8663	6890	12605	3102	160221
SUCLG1	25	18581	10109	15466	11561	21850	6360	43807
MVD	24	9865	5208	8460	6289	11936	4065	29081
COPB1	25	10909	5606	9822	6009	15101	2639	20434

PG.Genes	n	mean	sd	median	q1	q3	min	max
COPA	24	13512	9793	11999	7156	14695	4025	47812
CTSC	25	27299	60573	12589	6532	23228	4604	313220
LIMK2	25	8831	7276	6684	5035	9623	2823	38757
AP3M2	24	10006	3932	9176	7165	11923	4930	18586
AP2S1	23	7682	5268	6455	4777	8518	458	24855
TTC3	25	108832	114220	70186	51797	124123	26537	569750
SMTN	25	75739	76156	64594	33048	96336	9842	388681
SLC16A1	24	10834	22543	4453	3421	9073	1457	113356
SEC24C	25	6049	6473	4034	1897	7604	404	26342
SUB1	23	17483	10755	16212	8546	25432	2771	44652
CLNS1A	25	220420	139618	193978	144378	287032	8749	553925
CRISP3	20	7306	11485	2282	1137	6756	417	47223
BLM	25	7061	5654	5464	3705	7714	1892	28442
RARS1	23	8048	21623	2237	1090	4761	348	104131
ATXN1	25	6457	9432	4984	2782	6465	1378	50381
YARS1	22	4066	3546	2978	1810	5414	439	15599
HSPA2	25	30033	15709	27085	16593	40109	10363	61019
RAD23A	24	9650	4050	9042	8333	10149	4291	25745
RAD23B	24	10669	6794	10168	4988	14444	3342	28814
EPHB3	25	9585	13066	6122	4146	10754	1085	68020
EPHB1	25	6313	3328	6131	4697	7022	769	18616
AK2	24	27075	78133	10797	5676	14707	2330	392733
GAS1	25	309344	446446	233198	175281	300826	4944	2376445
ALDH18A1	25	14759	19138	11195	7331	13588	3047	103498
NAPA	22	4302	2357	3170	2635	5867	1448	9664
AIF1	24	6847	3168	5983	4558	7732	3070	14900

PG.Genes	n	mean	sd	median	q1	q3	min	max
EIF5	24	5786	5052	4363	2736	6435	1450	24073
PSMD4	20	3932	2719	3093	1849	5494	1113	12197
DRG2	25	29209	22290	23295	16167	33405	7778	109837
APOC4	25	269982	242944	230535	151818	319751	45061	1332684
PLTP	22	4043	3068	3208	2045	4860	614	11016
CSE1L	25	7911	22906	2895	1618	3846	508	116358
VCP	25	15301	9350	11600	9743	18317	7317	46001
MFAP1	16	4211	4481	2844	1438	4072	989	16801
HADHB	25	5804	2328	5991	4582	6848	1507	11305
MANF	24	110141	399376	18345	11694	28352	6836	1978524
AFDN	25	34698	23777	26438	23474	37113	14179	122602
CASP7	25	56926	26724	49766	37089	71901	23262	123327
CASP6	24	28692	27552	19309	13482	38049	355	132532
ADK	25	8409	6179	5907	4654	11687	2777	29246
LAMB2	23	5973	7145	3727	2785	5384	1060	34427
CDH13	22	13108	18793	7716	4445	12047	1415	92071
FOXG1	25	6517	2324	6369	5544	7583	1391	11513
SNU13	20	10030	9121	6602	2632	16796	1222	30469
NPEPPS	25	9395	7413	6745	3032	13260	1631	29548
HNRNPH2	18	5865	3834	5158	2847	8291	830	14053
XG	16	9550	20282	3361	1593	9028	383	84176
OXCT1	24	42404	63124	30304	18918	37626	8714	330512
EIF3B	23	4424	3208	4232	2632	5293	877	17263
NDUFV3	25	76188	106401	53447	28889	78426	8293	565722
MARS1	17	1804	933	1837	1001	2431	455	3369
ITGA1	25	21784	13216	18386	14359	25463	5919	62752

PG.Genes	n	mean	sd	median	q1	q3	min	max
P2RX3	25	96828	210906	55144	32844	71759	3638	1094262
EIF6	24	7415	4225	6992	4719	8706	2447	20853
CAV3	21	7711	4512	7401	3330	10327	1642	17806
CTBP2	25	21814	26135	19175	14041	23010	1012	140968
RP1	25	179219	253632	87499	79786	117624	48572	1135170
SLC37A1	25	44354	34214	35311	22062	51347	14347	174833
CFAP298	25	178668	132639	140472	63664	290374	22395	419993
GEMIN4	25	43195	102826	12356	9997	19172	5766	514922
EVC	25	96493	63711	75818	60385	106393	23569	306506
EPPK1	25	58241	35995	45386	40226	69301	25115	201663
MTPN	24	10048	6127	9083	5630	13074	1850	24242
NLRP6	24	7068	13398	2650	2194	5234	1423	67491
TAS2R39	25	20845	35299	7641	5326	12616	4209	125693
DEFA1	25	107733	112545	72249	37778	142461	1218	511288
GNG2	24	25954	45336	16659	12732	20790	1799	234896
ZNF445	20	4636	3232	3994	2370	6049	104	12096
ARPC4	25	70744	70638	59733	34140	78627	16559	375712
ARPC4	25	14120	11457	11376	8250	16652	2515	61341
CD81	16	2274	2150	1536	953	2794	306	7310
TPI1	25	111820	76642	100729	48140	149261	2051	294166
EIF3E	20	5783	3960	4960	1892	9284	671	12914
PTEN	22	8351	15936	5155	3605	6754	1390	79047
GABARAPL2	21	6764	4513	5011	3927	7825	2483	17883
MYL6	25	32778	49278	17555	10231	28851	2336	227063
PFN3	25	2004358	6078979	810315	417189	1153912	236377	31118472
ACTB	25	171342	96314	161046	95688	259760	19164	426493

PG.Genes	n	mean	sd	median	q1	q3	min	max
EIF4A1	25	74309	55478	62716	28069	111186	5829	216961
RPS20	24	16307	34262	6896	3681	9922	1474	168774
PRPS1	23	7602	3710	7459	4776	9283	2256	15681
PSMA6	24	18973	13268	13483	10625	19410	7812	64946
S100A10	22	10808	6562	8617	6263	15000	3119	24511
CDC42	25	9502	7857	6246	4511	12698	2770	32263
DSTN	25	59684	59033	30273	17410	89007	10256	234535
GMFB	24	15465	25231	10601	7309	14244	3112	132030
RAB5B	25	10381	5977	8342	5337	13601	1379	22244
RAB10	22	3689	2795	3217	1789	4426	850	13533
UBE2M	21	4681	3341	4271	1399	7982	785	10098
UBE2K	25	117801	264118	49968	38718	77643	20476	1356866
UBE2N	24	26195	17995	18926	11619	38060	6984	73765
RAB14	23	3760	1970	3777	2200	5020	788	8186
ACTR3	23	13757	10502	10932	4647	21769	2580	36417
ACTR2	24	9376	7155	7303	3180	13763	1950	23662
ACTR1A	22	4046	2583	4342	1639	5791	488	8197
ABCE1	23	9411	11259	6360	4460	10929	1975	57266
RAP1B	25	22406	18729	11611	9028	35854	3319	63533
RPS3A	23	4876	3998	3564	2403	6525	243	17684
PSME3	24	11401	25650	3282	1850	6133	1058	119667
MAGOH	18	4948	4460	3523	1810	6467	1368	19547
RPL27	25	36568	27543	28313	19462	43713	7165	131823
PCBD1	23	5864	3628	4825	2583	8256	963	13507
HSPE1	24	25839	22424	19305	11583	33277	2104	100709
LYZ	24	29907	54473	10369	7420	24250	1572	240676

PG.Genes	n	mean	sd	median	q1	q3	min	max
ST8SIA6	25	63474	57189	44178	36856	64725	21642	311266
VBP1	22	2953	2105	2639	1377	3316	368	9280
B2M	24	8070	7572	5362	3415	9007	872	26059
NPC2	24	8752	8635	5485	3671	9750	1367	37673
COPZ1	23	3291	2693	2776	1681	4046	616	13338
UFM1	23	7839	6655	7129	2525	10664	1498	26415
WDR5	18	2445	1691	1936	1549	2968	955	8120
NUTF2	24	15582	9911	12122	7077	21644	4042	40613
HNRNPK	25	74338	54440	69033	28989	93066	15371	238247
YWHAG	24	18652	11751	14893	10414	25361	4755	50703
TIMM10	25	32262	44248	20166	14447	39427	9192	237296
RPS7	22	9676	8213	8323	2912	13703	1156	31054
PPP1CB	24	8062	12538	5283	2922	7496	881	64468
PSMC1	24	2223	1246	2128	1237	2925	554	6102
PSMC5	24	6471	6073	3990	3223	6137	1581	26229
RPS8	25	6617	13559	3226	2371	4566	1542	70552
RPS15A	22	3058	1790	2762	1592	3683	779	7702
RPS16	23	3331	5154	2018	1143	3062	671	25731
UBE2H	20	2486	1453	2223	1540	3932	172	5246
YWHAE	25	33892	21858	28827	18423	43673	1809	97901
RPS14	24	193956	592852	58844	34739	98017	14626	2965518
RPS23	19	4367	3285	3434	1915	5827	621	14017
RPS18	22	5018	3914	4210	2420	6006	1299	15920
RPS11	22	4059	9187	1851	1045	3518	299	44686
SNRPE	23	17269	27793	7667	5200	14848	1513	127093
SNRPF	18	8902	12747	4693	2325	9238	459	48496

PG.Genes	n	mean	sd	median	q1	q3	min	max
LSM3	23	12383	12935	6776	3859	18231	805	54178
LSM6	21	11935	12205	7155	5066	13007	3059	57593
SNRPD1	21	6577	9466	2651	1550	7039	691	35552
SNRPD2	23	11327	8774	8683	5172	14513	3194	38363
TMSB4X	25	86688	110086	26629	11445	129166	1321	337041
ARF6	23	5048	5497	4199	2154	5400	764	26256
PSMC6	24	5507	4240	3976	3494	6283	1802	20834
RPL7A	23	4331	2356	3804	2797	5306	923	9675
ETF1	25	7847	3928	7179	4653	10148	3008	18519
RPS4X	25	29887	36278	23209	16407	32634	2529	196899
ACTA2	25	391971	306254	285056	164237	565444	45223	1232400
RPL23A	23	4618	5391	2968	1748	4701	327	24914
RPS6	23	12441	9385	9568	7412	12043	4528	45429
H4C1	24	64196	191820	25019	16426	34925	9676	963352
RAB1A	25	17588	11859	14183	9011	25021	2157	41178
RAN	24	38833	26396	31920	23822	46963	3659	96612
RPL23	19	3799	3789	3029	1172	4435	634	15143
UBE2D2	24	12742	12461	7059	3789	21741	933	48548
RPS15	25	13455	12401	7558	3787	20572	2227	42810
RPS24	17	5317	1899	4845	4589	6297	2228	9045
RPS25	20	6454	9886	3530	1721	6618	738	45694
RPS26	21	9318	13977	4177	2966	11373	641	66513
RPS28	16	3706	3892	2472	1387	4762	149	16344
GNB1	23	7242	6483	5022	3394	8655	1356	30237
RBX1	23	3371	1436	2970	2253	4171	1772	6611
GNB2	24	8427	6129	6291	4085	11563	1558	28827

PG.Genes	n	mean	sd	median	q1	q3	min	max
RPL30	21	2871	2167	2071	1338	3497	750	7961
RPL10A	25	28689	30097	18111	13119	35298	2693	159817
RPL32	21	48319	26288	52778	37039	67515	1254	106485
RPL8	17	2326	1257	1735	1619	3075	522	4852
PPIA	25	214668	138485	180855	100758	311604	15294	528088
PPIA	16	3069	2012	2609	1533	4527	533	7006
FKBP1A	24	24842	20013	18041	9967	34581	4153	75746
GRB2	20	5927	5649	5330	2333	6808	1006	26579
AP2B1	24	107179	193627	47233	33020	71075	25967	901399
PPP3R1	16	4611	5326	2434	1653	4483	976	21083
YWHAZ	25	79625	51256	80220	32994	111558	3163	188874
SUMO1	22	4492	4181	3380	1994	5466	831	19374
DYNLL1	23	6744	5733	4898	2478	8920	878	21692
RPL38	20	5039	5378	2844	1640	5151	462	17142
SKP1	24	27754	94507	7221	5181	9876	3536	470687
GNG3	22	8636	13880	3570	2858	7203	805	64227
RPS21	24	10066	8664	6830	4333	13140	1583	36717
RACK1	24	11408	10232	7867	3723	15216	1061	35845
ACTG1	19	4261	3065	3768	2660	5003	298	12168
UBE2I	24	48902	38514	33182	22879	70965	8513	166678
TMSB10	25	56014	68004	40234	35365	47070	20031	372795
PPP2CA	23	10491	6029	10246	5840	15056	2128	23949
YBX1	24	28452	26841	19741	7837	38530	2885	93213
CSNK2B	21	9087	4689	7973	6319	10793	1689	21925
TPM4	24	9118	13793	5032	3110	9041	764	68805
TPM4	25	297329	333828	134299	73194	450450	25818	1362429

PG.Genes	n	mean	sd	median	q1	q3	min	max
EEF1A1	25	141216	116936	95711	47952	220190	6288	383717
FKBP1B	25	3619	1791	3061	2614	4073	1319	9351
ACTA1	24	12556	7068	13724	6725	16766	1217	29769
TUBB4B	24	30261	24885	21361	9348	46930	3471	93390
CSNK2A1	24	53310	40192	45288	33273	57088	26561	229898
PAFAH1B2	23	19562	15777	12983	8028	24241	2151	54300
PSPH	16	2117	1127	2041	1257	2495	578	4785
RBM6	25	14460	8017	12179	10390	14547	7879	47804
RPP38	22	2388	1982	2083	1190	2696	280	9495
PIP4K2B	19	2326	2089	1934	1436	2398	894	10576
CCT2	24	6782	2787	6793	4420	8173	2094	12690
DENND2B	25	5564	2652	4870	3773	5973	1737	11214
ADAM17	23	6806	4889	6517	4386	6965	2061	25807
BLOC1S1	20	1365	637	1272	898	1838	458	2719
GPLD1	22	3950	2254	3413	2183	5021	1145	9275
LCN2	22	35565	66208	15721	6804	38580	300	318604
NUCB2	24	20976	45639	10883	8180	13741	4268	233694
S100A12	21	11219	12804	4826	2775	12858	641	43906
BASP1	25	4885	4813	3174	1905	5327	1549	23126
MRPS36	24	14326	8129	11968	9244	19867	4180	35921
MRPS15	25	18622	15705	12868	10198	16901	5748	65546
MRPS21	25	14069	24556	7289	3728	11500	1224	120236
MRPS34	24	9011	4446	7824	5635	12054	2808	17661
HMGN5	25	16046	27135	5450	3431	14078	1730	105036
SARNP	17	2617	1540	2179	1724	3137	668	7147
RBP5	25	81714	91900	68570	40407	85941	18699	499969

PG.Genes	n	mean	sd	median	q1	q3	min	max
LACTB	25	38205	35296	26225	21399	36574	6099	165215
COG7	25	9704	6751	7453	5523	12018	3672	32803
RPL24	25	79346	58946	66546	39244	94246	924	258941
CBX1	22	4847	6252	2724	1421	5487	277	28282
ARF5	16	2592	1176	2723	1658	3531	895	4284
ERH	20	12134	17223	6957	2245	11733	1220	67560
RHOG	19	1929	1125	1736	1306	2437	306	4813
RPL19	21	38974	96247	15096	13163	23670	6298	457502
SRSF3	24	12857	12435	9474	5340	14136	2163	57985
FOXK1	23	5292	3820	4270	3117	5927	1486	19006
CCZ1B;CCZ1	25	71452	171305	33042	26026	47881	15632	889646
TMPRSS15	24	23101	34160	9846	5340	23578	1810	153753
MUC5AC	25	35895	26578	30473	20550	46631	8757	137800
FBLN2	24	13879	12126	9573	6448	16878	1696	55148
VLDLR	25	3441114	2138412	2995816	1665610	4707903	956829	8014088
HSPG2	25	145491	157214	101995	64326	132428	46320	689427
CYCS	23	17870	19442	12943	4163	25144	910	89810
TFAM	22	4937	13744	1613	1017	2500	638	66169
PITPNA	24	5721	4126	4581	2915	6228	1789	18788
HDLBP	18	2726	2222	2499	833	3750	193	7709
HDLBP	25	8494	9345	6012	5137	7452	4174	51849
GTF2B	25	759904	356156	658898	472532	978942	251848	1547784
PURA	23	4757	4141	3173	2114	5280	1746	18715
CLTC	24	11879	13041	6840	5108	15441	2603	66290
HSF1	24	30597	26117	21759	10292	47350	774	104745
FKBP3	23	6561	6982	4977	1947	7884	449	31954

PG.Genes	n	mean	sd	median	q1	q3	min	max
REEP5	23	4785	4059	3247	1642	5938	652	13856
SORD	24	12076	10558	8042	4642	15493	2571	40470
HNRNPU	24	18633	14580	10794	7296	34232	1806	49179
SPTBN1	25	312364	172960	268959	226765	317315	167614	1012392
TIAL1	20	6724	6050	4394	3148	8948	947	25031
INSM1	25	10384	5792	9286	6506	12208	4122	25832
SET	24	7922	6813	6673	2995	10334	503	30024
SRSF2	24	15695	18374	10418	8911	13796	5861	97906
CTBS	20	1881	810	1723	1339	2176	1120	4787
FABP5	25	40981	62055	16441	9435	33037	5326	269387
ANK2	25	97183	91677	80905	64047	96524	33426	521508
PFKP	25	12269	19192	5757	4502	11973	2938	97965
EWSR1	25	484539	678205	302092	202579	510503	74746	3572268
OCRL	25	22991	17325	18910	13485	28350	6291	94987
TAGLN	25	579443	845432	96542	12631	834489	2092	2420018
OGDH	25	102585	46662	100642	80103	116345	14607	223437
COX6A2	22	8831	6077	7380	5399	8598	657	27965
ALDH6A1	24	13344	6357	13035	8396	18157	2043	24450
BDH1	25	979615	1460947	571827	413855	884097	263480	7696488
DSG1	25	17086	26030	10592	7428	15121	3444	135491
DSC2	24	13387	10860	9030	6758	16297	5080	52467
H1-1	19	1552	1144	1416	816	1892	501	5522
RPL18A	18	2626	2094	2107	1319	2698	638	7924
GHRHR	16	17184	23624	12928	9150	15038	3507	104160
GCNT1	25	29784	13480	25030	20618	39447	8512	62118
MAP2K1	25	62458	172120	16613	6763	43881	1469	871337

PG.Genes	n	mean	sd	median	q1	q3	min	max
FKBP4	25	33141	43906	19882	15256	33823	9894	234652
NUCB1	25	71394	64822	48221	40452	70339	23223	306869
RPL6	21	2361	2318	1577	969	3113	9	8249
TOP2B	25	31401	45619	15817	13699	21790	4418	212201
CREB5	24	61889	89186	44175	21893	60497	3113	448354
AKAP12	24	18469	9915	15536	11508	24874	5844	39476
DST	25	248890	295910	182754	148372	226880	91619	1627814
CAV1	21	13704	9528	11578	8014	17178	1923	45431
TGFBR3	22	4113	4105	2761	1183	5432	454	17682
TNFAIP2	24	12596	11709	8346	5053	16506	2562	54679
LMNB2	25	14976	9983	10987	8198	18147	6106	45647
CFHR1	25	7149	5230	5951	4143	8132	2062	28890
GBE1	25	7900	6218	6107	3417	9324	1709	24947
NOTCH2	24	9378	7345	7268	5571	10024	1245	31937
SSBP1	17	2888	2301	1777	1700	3658	969	9881
YWHAH	23	6662	3890	6634	3639	9313	1081	14341
CSTF1	25	14363	6994	13112	10378	16356	4104	36270
PTPN12	24	42342	17320	39486	34933	43802	14432	90050
CLC	22	5652	4648	3500	2756	8188	1513	17507
SRSF11	22	10380	8313	7380	4185	14579	2095	30696
CALD1	22	27711	42246	13902	5599	30186	1314	198380
CALD1	20	4038	4098	2511	1606	4173	783	16936
EML5	25	312648	328814	238389	213997	273950	157226	1865926
PTPN11	21	4269	3625	3161	1089	7652	321	11781
REG3A	24	134241	331374	41810	18780	97025	4101	1652016
PPP2R3A	25	78104	42810	70676	59358	91431	19925	247122

PG.Genes	n	mean	sd	median	q1	q3	min	max
PPAT	25	14674	15211	9781	5699	15974	4040	77001
EXOSC9	19	7404	5492	6389	3554	8835	2033	23533
PSME1	25	23808	12619	19324	14473	27397	11211	52556
FMOD	24	12874	12784	10817	4239	18034	1730	57524
PRDX1	25	209635	102483	202861	122884	262042	94087	511423
RPL18	18	2599	1769	2384	1256	3816	252	6339
C1QBP	24	11335	7811	9547	7402	11742	2650	38689
CKAP4	25	25618	26629	20431	9755	31455	2189	127669
TCHH	25	55371	86584	30109	13150	42095	3176	364780
TFF3	23	11711	8559	9461	6866	12888	4487	43777
KHDRBS1	23	9931	8425	8399	4509	11457	1728	37090
SOS1	25	15480	16025	11709	9603	15357	6552	89408
LRP1	25	179985	83313	154546	130738	210172	70191	430456
SRSF1	24	15618	42833	5158	3345	9015	2058	214350
ARHGAP1	25	28211	26246	15351	10274	36596	4926	95438
SRSF4	16	3527	4380	1853	1041	3816	417	14831
TGM3	25	15071	7398	14416	7921	20094	2758	30008
DHX9	25	28260	57194	13547	6723	23087	4504	295635
LGALS3BP	25	24034	37719	14663	6177	25772	2368	191746
EHHADH	24	4972	10717	2545	2139	3198	1164	54872
PPID	25	12238	5664	10347	8206	14338	6157	26599
SSRP1	24	43618	73549	30204	20953	38752	3439	385380
SLFN5	25	14946	10901	12170	10450	13519	7206	56005
ZNF616	23	45719	38992	38595	17611	56284	3682	174898
RBBP4	25	27189	28136	19936	13394	28422	6491	133160
NCBP1	24	43847	33813	36286	23491	50130	685	147679

PG.Genes	n	mean	sd	median	q1	q3	min	max
AHNAK	25	193412	165863	148837	99113	200858	39718	742578
EGFEM1P	24	39235	15233	38574	25972	50565	15114	69002
ABHD18	25	16648	12901	13581	8365	23599	1515	51370
LRRC74A	23	6253	4595	4663	2649	8623	1564	18029
HSPA14	25	89629	57301	74815	59258	102470	22045	304999
SCRN3	24	19483	28600	9266	5212	19642	3839	123035
CGNL1	25	437020	204880	398207	332357	508406	209512	1257779
CCDC173	25	40524	17678	35944	28588	42882	25045	97910
FABP9	25	15165	30955	2896	1577	14266	613	146286
NEXN	23	31116	62324	16864	11833	22123	6381	312971
GALNT2	20	8062	11316	3681	2211	7052	1431	45306
GALNT1	18	7037	10623	2735	1608	5820	396	44683
AP1B1	24	11376	19026	7162	3766	10604	2297	98423
BST1	23	47156	49110	35015	13440	59067	1986	182730
BST2	19	3280	7213	1358	1058	1829	258	32526
HMGXB3	25	18141	15608	12707	9087	21986	5565	79510
WASHC5	25	35940	57663	21639	18813	29513	11188	303454
NUP160	25	39639	129885	10994	7063	16027	5104	661513
SCAP	25	6546	5091	5396	3287	6921	962	25364
SREBF2	25	5131	3334	4619	3607	6792	156	13086
ARHGEF5	25	83101	220471	25854	20203	47396	14567	1126130
GTF3C1	25	21792	12974	18360	13002	25884	10413	72968
HYAL1	21	4969	4268	3891	1618	7618	45	15844
SMARCB1	25	7579	5893	5966	3799	8535	2031	30444
FSTL1	19	5111	9717	2753	1476	4120	746	44360
SF3A3	24	5830	4202	4709	3254	6558	1172	19398

PG.Genes	n	mean	sd	median	q1	q3	min	max
DPYD	25	54644	70500	28465	22402	43671	9248	271059
ILF2	24	15520	15205	9300	6007	17886	2979	61639
ILF3	25	40967	20472	34290	23505	53398	14401	82267
LMAN2	25	29198	17831	23257	19501	36432	4971	89429
IRAG2	24	4246	1951	3979	3021	4948	1750	9185
EPS8	25	77444	77677	47023	32308	88386	17387	382900
FOXF1	22	4592	4956	3046	1797	6114	6	22807
FOXD4	23	6248	4886	4543	3431	7076	2755	24872
ANK3	25	21104	11797	17378	15783	22903	11182	70082
MYO1E	19	44798	48283	27958	16937	51915	8142	211322
PPP1R8	23	7541	15450	3228	1826	4819	454	71524
ABR	25	13472	9298	10025	7782	14398	4571	42555
CSTF3	25	14463	14294	9971	7321	13378	3281	70464
ECH1	21	3260	1818	2575	1981	3845	954	6836
STRN3	25	4686	2885	3635	3161	5209	1387	13680
FLII	25	47618	26973	33997	27655	65443	17808	122087
LCP2	17	2319	1829	1969	1075	2573	417	7723
USP4	23	4630	5355	3121	2272	4185	952	23484
CHAF1B	25	17781	8501	19170	11490	24063	4846	34235
DUSP4	22	3371	2572	2348	1812	3846	1180	12334
KLF10	24	15195	23559	9012	2690	18979	428	117069
MTAP	23	6465	3956	4900	3650	9697	1492	14127
TARDBP	19	3748	5341	2053	1340	3617	645	24656
HNRNPA0	24	70069	85891	50613	32822	66018	16228	436625
PAK1	24	5108	3453	4235	2466	8179	790	12369
AIMP2	25	32124	56454	19583	14348	23754	3324	296632

PG.Genes	n	mean	sd	median	q1	q3	min	max
PRDX4	21	9158	16995	3395	1614	7751	980	78648
PAK2	23	2689	2023	2052	1189	3403	491	7349
CBX3	22	5421	3548	5086	2732	6827	932	14069
STK3	25	20767	23073	12107	9635	16554	6045	101977
PSMD2	24	7434	2845	8445	4864	9451	1442	12006
MMRN1	23	20272	49011	6940	3895	13270	647	238004
DDX10	25	16887	14178	13296	9458	16053	6081	70251
DNAJC3	25	6471	4302	5115	4706	8070	1542	24421
NME3	21	4004	3115	3403	2595	5214	287	15900
SRSF9	15	6455	7186	4242	2332	6222	1007	27272
SRSF6	24	12043	13712	6973	4281	12576	976	57624
TRIM28	25	11731	9428	7610	4396	17176	1699	33664
G3BP1	21	4516	4636	2499	1756	4528	110	17560
NMI	22	22751	84536	3746	2860	5517	1269	400861
SLAMF1	25	5704	8172	3199	2233	4887	713	36821
EIF3I	18	2323	1594	2238	923	2948	313	5983
ILK	24	10201	11426	4651	3048	13206	713	36486
MSLN	24	15241	21913	7528	4787	12627	1368	98781
NNT	25	77759	88916	59828	49768	71659	32403	495228
SNTB2	25	6208	2055	6112	4962	7140	2818	10971
PPIG	25	21389	19528	15600	10140	20535	989	75235
TCOF1	25	25433	54123	10869	8149	15303	5884	267865
TCOF1	23	91956	89322	51666	27296	167426	1783	295629
SF3B2	25	129188	103473	101666	79256	138626	39505	584246
GOLGA4	25	70903	52635	44528	34538	107231	24237	230532
PDAP1	25	20718	22091	13277	10871	23485	5153	116812

PG.Genes	n	mean	sd	median	q1	q3	min	max
ADAM9	22	11080	8600	8853	4119	14180	721	27866
LSAMP	25	11192	15028	8379	6565	9861	4118	82237
FKBP5	22	4203	2407	3722	2619	4518	1579	10002
MYO9B	25	30791	29438	24062	17062	28443	14503	159273
ROCK1	25	16169	20232	11801	10193	14691	4736	111164
IL18R1	24	4461	3448	3764	2111	6226	332	14909
SMAD4	24	5427	3740	4783	2636	6782	1031	17148
SNAPC2	25	1245615	899417	989777	658005	1596993	121165	4260828
SQSTM1	25	58314	20719	52651	44614	68076	16058	104858
TUBB3	17	7362	4603	6602	3403	10045	671	16862
PPP1R1A	25	70905	77230	43226	35400	66787	10351	378282
PRPF4B	25	22418	13859	20402	15865	24509	3939	72294
PIN1	24	5491	4183	4205	2934	7139	1068	18968
HDAC1	23	10389	7660	7484	4370	12969	2681	30863
DCTN2	25	17416	47589	7131	2880	11007	1886	243715
IQGAP2	25	36625	33575	24667	17861	39589	4414	152878
GPR50	25	49298	24131	42108	33178	58154	22085	125597
STIM1	21	37837	42881	22198	15157	46332	4810	194926
TRA2A	24	41676	48262	28564	20676	44399	6759	251146
SNX1	25	13136	41020	4097	2636	6094	1380	209176
KRR1	20	2244	2106	1564	950	2597	566	10030
CUL1	25	10937	9404	8909	7680	12667	1124	51430
CUL2	25	52441	42526	38486	31424	43370	21805	198833
CUL4A	25	14101	24359	9211	6674	10513	1979	128619
GFUS	23	6446	3043	6199	4536	7636	1470	13540
RAB32	19	3837	4449	2156	1127	4197	78	18215

PG.Genes	n	mean	sd	median	q1	q3	min	max
ALCAM	25	103214	51547	97917	62201	129393	30895	209407
LAMB3	25	40171	67636	21775	16239	26246	7782	301714
THOC5	22	2847	2120	2217	1561	3310	423	7519
SPTAN1	25	28775	15271	23915	20155	31985	13126	86162
AUH	24	163079	521432	18562	12100	39711	3631	2564383
DDX39B	24	151420	120408	97408	72747	211943	42915	498792
BLMH	25	12827	12252	9753	5749	12485	3383	61498
EXOSC2	24	33004	15272	27801	23052	38565	15842	73563
SNTB1	23	14339	6994	12374	9684	17111	4829	31045
TUBB2A	23	10254	7966	7632	3957	15161	1310	32340
BYSL	22	3095	1758	2712	1957	3982	767	7939
RAPGEF1	25	34514	38069	21053	13604	40555	2207	160920
CAPS	25	8774	6149	7338	4764	10732	2879	30720
CBFB	16	3868	1976	3158	2173	5041	1674	8366
IL16	25	15296	18841	9241	8025	14378	5694	100852
COTL1	25	26785	30623	14231	10039	35210	4227	130470
CYLC2	25	20601	22049	13845	9417	21056	4873	95366
HNRNPD	24	43758	56375	22494	10290	64385	2100	269205
DPYS	25	21930	12323	18081	15985	24953	11045	68888
DAG1	24	16110	26320	7583	5275	15489	2632	130714
VEZF1	17	20397	64115	1964	1052	6099	442	266965
DSG2	25	204748	393112	93947	61656	172608	34110	2038806
SEPTIN6	20	4519	4645	3655	1895	4712	262	21892
MORC3	25	38343	46234	27154	23254	31842	19003	254111
SAFB2	25	65163	52593	49436	40423	69196	10058	259468
EIF3A	25	12055	8046	10012	9004	12713	4942	47707

PG.Genes	n	mean	sd	median	q1	q3	min	max
TTLL12	21	8441	11965	5584	3225	7284	223	57816
DOC2B	25	796629	571253	744446	519190	963748	144765	3160702
DOCK1	25	18171	18803	10011	8117	20840	5578	91585
WRN	25	81681	47459	67198	60383	82996	43626	281780
FHL2	22	4053	2387	3269	2015	5949	963	8447
CRMP1	25	9116	3906	8529	5535	11174	4493	17108
DPYSL3	25	31835	37333	15604	8628	30125	4403	164989
MRPL58	25	5293	3700	4237	3278	5695	1487	18154
DYNC1H1	25	498458	446061	400983	331540	486216	179115	2500580
NPAT	25	65414	139761	32177	28128	43311	18259	730715
EIF2B1	25	9695	7889	6295	4299	12897	2588	30770
ADGRE1	23	58025	88150	21431	6648	63594	2286	361983
CTTN	25	143611	128618	106286	78311	154070	41032	604507
FLOT2	25	10356	3430	10698	7496	12985	3934	18601
TRIM25	24	34506	9949	33998	28944	40833	13315	57203
PTK2B	25	28859	15010	23009	19842	32410	11208	67278
FGL2	17	1880	647	1855	1527	2542	646	2922
FAM50A	25	35688	21607	32550	22604	44713	6931	109202
LRRC32	15	11156	15111	5092	3772	11533	2527	62922
GK2	25	61884	111186	21180	14277	46300	2789	483613
PDE3A	25	23858	13018	19235	14717	35510	4325	49050
RBM39	25	177623	55327	171780	149903	196960	91074	335787
WFDC2	20	57572	79460	40538	2630	69692	226	343680
SPARCL1	23	4915	6724	2522	1603	5009	670	30850
HABP2	24	6895	6884	4286	3064	9084	1344	33326
HNF4G	25	11983	21971	7767	5569	9967	3162	116005

PG.Genes	n	mean	sd	median	q1	q3	min	max
PDIA5	16	5228	6885	3666	1541	5774	406	29420
PRPSAP1	22	24691	27552	18639	11198	25633	4401	139905
MCM6	24	5766	6626	4257	3416	5214	1894	35761
ITPR2	25	9529	8514	7291	6441	9229	4631	48307
ZNF268	25	161781	120171	129181	73521	181961	19655	429552
IHH	21	3315	2584	3003	980	4651	94	9486
ITIH4	25	39568	16426	39619	26253	46176	11679	86870
PLS1	17	2814	4373	1466	940	2465	274	18867
LAGE3	23	5911	5086	4273	3481	5386	1905	24802
KIAA0100	25	13267	30491	6581	4504	9663	2380	158772
MDC1	25	2724	2557	1951	1616	2916	1146	14258
KANK1	25	78412	123200	46119	32741	53529	23697	596856
SMC1A	24	93078	269441	34023	26437	43633	21360	1355592
RRP1B	23	10569	6449	8890	5775	11655	4625	30310
DIP2A	25	53354	31992	46693	37975	60716	15018	167208
BMS1	25	9941	4030	8892	6900	13018	4989	20504
MESD	22	39084	160887	2622	2247	4976	378	758488
GANAB	25	8495	5659	6208	3363	13057	2217	21172
RFTN1	25	7248	7120	3949	1350	9888	541	22775
KCNAB1	25	62249	35062	62688	34762	75769	16850	163885
LBR	21	7000	4970	5473	4348	8793	1102	24940
MVP	23	8094	6465	5871	4487	7439	3379	31039
LTBP1	25	11503	10564	8560	7044	10563	4746	56923
LTBP2	25	8536	4166	7239	5508	11917	2415	15763
CHD4	25	13718	11480	9301	7294	13876	4787	56654
CRYM	20	5646	2566	5339	3563	7151	2560	11144

PG.Genes	n	mean	sd	median	q1	q3	min	max
KIR2DS1	23	4346	2890	3593	2639	4911	1527	15278
KPNB1	24	7357	5045	6086	3371	9064	2005	19112
PSME4	21	3419	5758	2409	928	3521	97	27793
NAA25	25	19463	23421	12798	8302	19586	4247	117326
NCAPH	25	80033	31217	73159	60410	95916	30408	151149
PCLAF	22	8072	7080	5047	3994	8867	1153	31342
WTAP	25	6816	3623	5967	4270	8365	1729	16834
PSMD6	25	3634	2279	3171	2667	3633	1624	13415
MAD2L1BP	24	7245	7721	4637	2621	9666	859	33982
ABRAXAS2	18	2220	1815	1545	830	3180	296	6041
SART3	25	41253	28209	32220	24744	39748	15464	127786
NCAPD2	22	16382	8823	13295	8897	21119	6924	34597
SUZ12	24	8552	6481	6382	4645	9920	1056	28095
ACAP1	25	23892	23471	15243	10625	23736	7322	105010
SNX17	22	16223	9554	14226	8161	21367	2831	40370
KARS1	25	7809	8590	5462	2826	9372	1823	41780
LRRC14	24	19465	57569	6794	5290	10740	1438	289232
ACAP2	25	26354	36009	14421	7960	26870	833	172799
WDR43	21	13609	14375	8883	4235	16224	1900	63974
POSTN	23	6715	7581	2702	2158	10709	981	29861
EEA1	25	42840	100252	9102	7956	27148	6244	427441
NCF4	23	4270	2063	4079	3285	5043	980	9641
PAFAH1B3	22	6608	2872	6826	4466	7946	1419	12177
PLCL1	25	125295	113074	89823	64455	131584	38559	481760
PCOLCE	24	13544	10867	9897	5786	18002	2688	44457
PGM5	25	16329	14975	7080	4265	29052	2220	43904

PG.Genes	n	mean	sd	median	q1	q3	min	max
PMVK	18	2103	2072	1309	807	2194	247	7078
PRKD1	25	7806	8166	5400	3615	7774	830	39720
PLEC	25	36705	21332	32726	20875	38128	14275	97221
PPA1	25	24127	44350	13261	7004	23767	2482	231695
PDGFRL	25	6399	5869	4542	3539	6974	2285	31239
NONO	24	9966	10979	7486	5789	11100	1123	58825
QPRT	25	32254	21534	25435	17119	45523	6237	84183
RABEP1	25	69410	58918	49809	34812	82186	23936	313932
RAB35	20	11944	14647	6421	5338	11511	1530	56684
RCN1	23	3333	2251	2243	1787	4880	408	8576
ANKRD1	17	4442	4080	3458	2180	4394	340	14770
TTF1	24	4667	6660	2996	1901	4735	1025	34474
TMED2	17	2192	3584	1438	745	2244	232	15815
PCBP1	24	28899	22999	26372	8064	42383	4744	77394
PCBP2	25	12219	8704	12946	3731	20334	2061	30578
ELOC	20	3154	1997	2880	1352	3994	434	6862
RHEB	24	4551	5874	3240	2503	4073	1675	31596
UBE3C	21	25369	72509	9946	6322	14361	2315	341168
SF3B3	25	204410	195195	137637	84050	234012	39818	893453
PUM3	25	90272	90773	76189	41472	88812	30594	495449
RSU1	25	16635	13372	12570	4316	25696	2295	47831
CNN3	22	30263	36262	16166	4155	42491	1031	150217
SAFB	25	25895	33199	16382	13383	20375	9715	170565
SF3B4	18	5205	3691	4099	1775	7579	337	11905
SF3A2	25	26616	25615	23105	10684	30997	1132	113578
SEC23A	25	9059	6142	7823	5717	10004	2005	34050

PG.Genes	n	mean	sd	median	q1	q3	min	max
SEC23B	20	2609	1442	2388	1363	3246	682	6065
SF3A1	24	11997	7258	9802	6850	15576	3711	33290
SKIV2L	25	86266	309453	18622	15965	24046	8208	1569011
RGN	18	20494	47689	2310	1051	4027	450	192736
CDSN	25	15880	11356	13098	8541	18049	3935	56521
TCEA2	23	11557	6735	9721	7724	14862	812	28075
TGFBI	25	21231	43068	6250	4978	17305	3862	216032
DIXDC1	25	30991	14749	30299	22812	34377	4288	78360
TSN	25	24037	34000	15046	10927	20670	2053	168294
TRIP10	25	112955	196095	53698	44703	99649	9215	1021955
TRIP4	21	7251	11421	3241	1307	6361	601	43814
TRIP6	21	8237	24185	3004	1765	4164	657	113548
MAPRE1	25	21463	13728	14860	10083	28507	6007	55626
TSC22D1	21	4427	5173	2715	1038	4974	271	21457
ELAVL1	24	19884	19002	12380	6305	26597	2733	77681
INPP5J	25	19700	9292	17481	14935	22927	5154	45255
HERC1	25	386594	272687	306467	266944	412381	198920	1610773
TBCC	24	7140	6501	4759	3003	8972	898	27469
UBE2V2	24	53269	191534	13785	7618	21686	3809	951673
NEDD8	16	4648	5218	3287	2441	4697	663	22919
ADIRF	16	12254	15503	3522	1887	19134	623	52363
ADIPOQ	25	81759	133621	46890	39072	66381	19140	708483
RAB11B	25	51190	40517	39897	21037	66757	15297	205459
ZYX	24	23694	41238	14038	3616	26324	1406	205833
CCDC6	25	32460	15854	29302	22191	43009	9306	75739
ENOX2	25	479292	670460	294945	192603	442384	157426	3520347

PG.Genes	n	mean	sd	median	q1	q3	min	max
LAMA4	25	88225	361760	12736	10651	16860	6763	1824110
SSX1	25	10629	7030	9863	5046	14384	1652	27554
CSRP2	19	7764	6000	6180	3522	8378	1140	22209
DDB1	25	7848	6518	5748	3470	10074	2062	29583
CDC37	25	25431	23435	19228	17180	23324	11254	127601
DPYSL2	25	22847	28255	14577	6640	30621	2616	142666
SYPL1	24	8203	6709	7186	2884	11136	793	28053
RBBP7	18	1865	824	1795	1350	2301	580	3293
ZNF239	19	13634	13570	9086	6321	13143	2501	52634
CALCRL	22	27241	40197	10492	7030	23698	1637	177366
SRSF7	21	4375	5351	2107	1081	5418	325	24100
FSCN1	25	25957	26183	17633	8843	31024	4540	111712
IFI16	24	11599	13861	8450	6336	12109	4126	74628
DECR1	19	2691	2382	1622	1000	4150	289	7992
MAN2A1	25	55742	37745	45774	30514	63937	15502	155946
TST	25	24273	29505	16541	13067	23309	10339	162431
RTN1	25	27553	36445	13113	9633	23134	4902	173216
UPP1	23	4496	4344	2546	2130	4699	830	16218
UGP2	23	36291	52792	20851	11602	33449	2677	260345
UGP2	24	10660	10612	6807	4729	10811	3212	52930
TXNRD1	25	21058	13947	16799	11135	24383	7471	59220
IMPG1	21	4238	5814	2827	1673	3543	506	27787
LONRF1	25	69953	106146	54217	35603	60420	21145	572381
NKPD1	24	16757	9485	14851	11425	18714	3542	50313
HNRNPUL2	24	9438	10641	6074	4156	9775	3116	53961
INF2	25	76175	75715	53582	41075	80617	10143	390908

PG.Genes	n	mean	sd	median	q1	q3	min	max
PDS5A	25	21631	5657	21750	18928	23907	10857	34899
C5orf64	24	17026	12683	15973	10754	17580	7002	71765
CCDC96	25	16803	9714	15105	10794	18578	7359	55996
WASHC4	23	2825	2504	2249	1745	2863	719	13512
SHROOM1	24	35755	47737	10215	7135	58201	1826	171111
TSR1	25	4255	5058	2580	1776	4210	564	25808
IAH1	21	3547	2548	2520	1460	5895	184	8177
SMU1	19	2587	1559	2152	1342	3593	607	5833
SMU1	25	41560	35091	24266	17928	58482	10019	135103
ASTE1	24	11225	8946	8301	5627	13509	1593	36434
HKDC1	24	46301	29240	39006	28592	50163	1247	120086
PRTG	25	435973	1153795	52101	37589	113252	8919	5430118
DEFB115	19	7597	6352	6047	2136	13565	812	19857
MYLK3	25	24858	13806	19937	16587	32694	10288	68256
LRRFIP1	25	60484	23074	53470	44613	78912	21204	103967
LRRFIP1	19	3625	3377	2100	1393	4781	198	10887
P3H1	23	29789	12536	26888	20781	35100	14993	69747
TRMT5	25	8395	5015	7584	5129	10185	1687	18289
C2orf76	15	2190	976	2108	1327	3005	870	3608
TKFC	25	456164	553406	333319	251905	424756	141619	2994933
OTOGL	25	21828	20001	16609	14783	20347	8200	114342
LGALSL	24	49505	31943	37371	23261	70517	9960	112910
AMZ1	25	14690	7577	13255	11143	15259	4125	37151
SV2C	22	2820	2043	1917	1515	4655	9	6998
ZFP69	25	33697	22163	27838	17038	46641	490	95843
MAP9	25	34436	26070	29116	21137	37691	13567	147845

PG.Genes	n	mean	sd	median	q1	q3	min	max
VPS26B	19	3913	7418	2123	1458	2803	873	34272
HYDIN	25	286295	220233	236804	197566	259018	166866	1274960
TBC1D10B	25	52570	37378	39379	23506	76395	6500	144032
PLCH1	25	399495	274380	327003	272697	447001	185197	1611520
FILIP1L	25	8874	4690	6271	5431	10930	4710	20396
AMOT	25	35151	36615	27198	17742	33404	8790	192355
ATP13A5	24	6233	2406	5978	4552	8248	2414	11446
TIGD2	25	30505	56178	14678	8620	31858	4454	289322
GREB1	25	26747	8023	26537	21281	30969	12809	45483
TMEM259	22	2457	1032	2380	1933	3130	469	4591
CCDC38	25	96586	134904	50011	35278	80353	14420	648225
ATP6AP1L	18	26563	49961	5285	2194	21759	301	202711
CCDC184	25	17223	11596	14250	9914	20456	4526	50200
SBK1	22	3110	2059	2859	2161	3576	255	10633
PDCD4	20	8412	7054	6415	3877	8600	1105	25742
FNDC3B	24	440866	790852	124659	99375	413037	11329	3496809
CRTC2	25	6974	3719	6225	5159	8582	1991	16442
NCBP3	25	26617	23885	22663	14790	28408	2836	128822
TP53I3	22	4108	3846	2287	1719	4435	829	15696
SLC44A4	22	4796	8325	2965	2015	4171	1385	41674
SLC44A4	17	35704	15727	33020	25523	44123	13946	68250
PDLIM3	17	12114	17142	8299	4541	11706	1054	75527
LACTB2	24	10584	30532	3480	2422	5566	1579	153307
SMUG1	20	34410	22416	25148	21459	37085	13209	93530
SOWAHC	25	13625	13945	9206	6231	12754	1739	52514
ARHGAP15	25	20667	18950	14494	8536	25279	1541	77598

PG.Genes	n	mean	sd	median	q1	q3	min	max
FASTKD1	22	13608	8155	13456	8106	16760	2459	32101
ASPRV1	19	2878	2098	2702	1119	3595	520	8500
HS1BP3	24	22596	47032	12649	9417	17225	4829	242195
INO80D	24	6322	6859	3758	2831	6499	1356	29350
ACTBL2	24	12948	7200	11413	7715	15618	3951	32617
CCDC93	23	16074	41130	7275	5498	9132	1520	203919
OCIAD2	25	12418	8825	10900	7542	14393	2441	46540
HSP90AB4P	25	280511	262101	198171	146957	327532	76467	1388836
HSP90AA4P	25	42636	27817	36962	23825	50670	6875	117926
TTMP	22	12997	24552	4804	3031	11671	1227	116244
NOM1	24	3539	2809	2751	2366	3169	1899	15264
ZC3H12A	23	9379	8941	6410	4531	10511	1971	44977
TNFAIP8L3	22	4562	3721	3274	1725	6038	691	13940
FREM1	25	44967	55244	26756	23871	37792	16866	278648
RTL5	25	75443	40792	70106	41622	98555	16619	157680
PABPC1L2A	24	4873	3839	3912	1926	6284	369	14945
PRRC2B	24	7650	3678	7301	5066	9487	3172	19499
KLF17	23	16463	29053	9738	6484	11695	1975	146361
COA6	21	4474	2649	4073	2084	5989	1029	10225
AARS2	24	7503	3282	7442	5550	8583	1168	15758
SPIN3	23	5718	5705	3622	2208	6824	1083	26482
GNAS	25	17872	29090	9927	5616	12184	2727	121571
KIAA1755	25	52164	82106	29860	24650	45641	13829	426640
PRSS36	20	8738	3851	8622	6334	10785	3122	16994
SAMD9	25	162995	133482	126282	110006	150107	41020	745426
DGKK	25	21377	34552	11940	11215	16798	8659	185213

PG.Genes	n	mean	sd	median	q1	q3	min	max
SPECC1	25	16995	15050	13281	11399	15165	8298	83367
WDR45B	25	5781	3502	5448	2998	6990	1292	12636
EOGT	23	14951	14531	11186	6837	18387	516	68980
NEXMIF	25	59056	65564	42658	32681	58865	28073	363446
TBCEL	25	34634	47184	17921	9448	30285	4275	204230
ELFN2	25	37342	18496	33434	22204	44604	8698	70675
TTC38	25	7444	12582	3688	2742	5785	1563	63169
EXOSC6	18	3623	2464	2693	1904	5788	543	9333
RBM48	23	7687	3788	6386	5526	8847	2174	16205
LRRK2	25	39201	37038	29886	22298	38663	13262	186098
PHYHD1	24	4195	5626	2785	1770	4592	654	29217
HP1BP3	20	7064	9602	3156	1983	8137	1104	43424
FRMPD1	25	58653	37283	48883	39392	63036	33954	216004
C6orf141	25	95139	150112	59822	46443	76872	28458	796504
GLYATL3	25	26840	21805	19831	14475	31699	5817	116731
FAM120AOS	23	13539	10264	10723	6711	17191	183	37615
ZC3H13	25	114112	83266	89202	76137	114610	31754	443927
TRAPPC3L	15	1458	1328	816	637	2039	276	4455
HECTD3	25	13122	11548	9391	7752	12979	3672	55223
SFRP5	24	20554	17744	15243	7518	25064	4300	70498
UBR4	24	9162	4941	7677	5405	12085	3796	24666
UBR4	25	35688	22387	28819	25631	34821	17764	124303
ZNF684	25	6544	4646	5176	4104	7810	771	23858
KIAA1217	25	52537	29121	41707	36515	54971	23296	137342
UBAP2	25	9026	8192	6910	5283	8403	1123	40297
C9orf64	23	3916	4636	2357	1362	4454	815	19144

PG.Genes	n	mean	sd	median	q1	q3	min	max
KPRP	25	13677	13601	9832	6621	12858	2099	67761
XP32	19	2273	2610	1186	770	2025	448	8709
L1TD1	25	268156	229524	225379	151208	277441	70651	1284637
SYT6	25	58482	111693	23358	11084	45881	4078	517262
RNF187	17	3266	2568	2316	1288	4879	605	8568
TTC22	24	5670	5993	3684	2648	5831	581	30369
DCAF8	24	3426	2136	2947	1644	4827	580	8887
RNASEH2B	21	3675	2376	3304	2165	4292	1133	11563
RC3H1	25	19655	18251	12292	9480	20158	5168	88835
OGFRL1	24	91623	213463	9842	6791	28498	1819	732504
MAGI3	25	51662	128843	24373	18277	34619	9683	667938
SH3PXD2A	25	63739	36010	54858	43081	73119	19580	172611
RSPH4A	23	105325	80087	81981	60829	127089	6149	390718
DDI2	23	4169	2580	3814	2367	4930	1593	11634
C20orf194	24	10117	9496	7604	4166	11028	2061	42815
C6orf163	25	49239	42561	37195	26291	47309	16273	204461
NT5DC1	20	53058	55907	39944	25950	47955	15103	212511
C1orf195	24	15556	16003	10401	4464	23217	725	70545
AHDC1	23	16323	15625	13026	7547	20463	2079	75902
VPS13D	25	24678	16328	19353	18231	24475	12417	79476
CROCC	25	124636	44683	123912	100020	136936	30802	283455
AGBL2	22	5886	6508	4477	2321	6222	515	30696
ATF7IP2	23	5043	9908	3023	1832	3555	955	49925
RASIP1	25	125172	227514	58776	39792	88425	23252	896815
STRIP1	22	3499	2326	2705	1809	4790	1051	8868
SYDE2	25	8332	6023	7562	2634	14101	1225	18275

PG.Genes	n	mean	sd	median	q1	q3	min	max
RNF220	25	19297	14147	17665	12805	21800	2419	68918
PRPF38B	25	106306	153120	75423	41298	106213	6926	804278
RNF20	24	11064	3258	10653	8895	12473	6385	20191
ZNF318	25	21003	24003	14094	11466	20613	6933	131433
DIPK1B	18	4801	4545	2487	1847	7096	498	17919
ATRNL1	24	14510	10257	9098	7221	21822	4161	44238
SPATA31A6	16	34915	117057	4847	3917	6175	556	473596
BROX	23	11471	6890	9547	7694	14782	2076	28888
FOCAD	24	283256	327585	199398	139064	272195	93167	1715273
TAF3	25	101231	56660	86250	72461	108973	49034	325096
PRAMEF8;PRAMEF7	24	9470	9238	6155	2317	13413	1060	35631
RSBN1	24	6208	5292	4026	2820	7466	1338	19992
LYPLAL1	24	7177	4686	6851	4506	8839	2079	25410
ECPAS	23	3720	2355	3436	2352	4349	1348	12706
TSHZ3	24	10241	6792	8260	6033	11662	4210	35058
AFMID	25	463167	482221	327472	84669	499255	7080	1698739
KANK2	16	5221	5127	3821	865	7759	212	18251
LARP1B	24	13816	13245	8992	6111	15816	1586	58800
CEP135	24	4623	2334	3995	3632	4985	2560	14403
TMEM198	25	107910	72074	89360	67709	132395	24415	356291
MAP1S	25	7968	7828	5229	4005	9697	1680	40090
E4F1	22	8874	16573	2364	1934	7268	1301	76859
PPP2R2D	18	5536	3613	4795	2954	7249	124	14058
ATG9B	24	18023	45487	9043	5091	10660	2690	230394
NUGGC	25	240297	238559	168449	137796	229874	70486	1201359
TNS3	25	58197	40306	43801	33887	66385	16092	216860

PG.Genes	n	mean	sd	median	q1	q3	min	max
ANKS6	23	10486	4112	8911	6856	14789	5858	17534
LMBRD2	21	2815	1868	2018	1678	3703	875	7545
ZFYVE26	25	33834	22732	26595	18525	34619	9899	89978
MSL1	19	8947	11833	5451	2367	7711	913	47817
C18orf63	25	14864	14133	11265	8842	13779	4708	71582
CWF19L1	25	205754	289872	144175	96806	200448	32888	1549070
CYB5R2	19	3855	3020	2823	1659	6003	591	10211
UHRF1BP1	25	19646	16503	15000	11874	20134	5290	80448
SP5	16	1651	1227	1060	610	2445	347	3683
ATL3	25	27628	39509	14539	8894	24408	4479	198047
ZNF470	24	37081	31675	28125	15893	43298	6899	145360
VASN	25	18710	13341	15400	10352	24144	1650	49502
CIAPIN1	22	8860	7956	7002	4338	8213	1491	32988
SMYD5	22	21474	63148	5435	3931	8968	1358	302290
NADSYN1	25	5756	2800	4906	4189	6463	2062	13661
TWF2	23	4358	3252	4096	1823	5840	839	14594
TANGO2	23	13575	32930	6934	5127	8300	2942	164280
RAB12	25	17932	14622	13764	11256	17967	6683	77689
KLHL10	25	5283	5624	3087	2205	4836	1567	26364
KRT80	25	11023	16787	6508	5569	8429	2631	87921
NIPBL	25	421273	145486	396466	322203	451830	200683	756341
TET2	25	16548	6697	15091	12614	21360	5489	32354
NAA16	25	21541	17331	17715	9271	30286	2359	76012
CHADL	25	59605	37127	50296	42188	71895	4635	171604
PSAPL1	22	6804	4832	6009	4131	8085	1791	23155
TYW1B	25	67041	76596	45510	31363	63174	1656	397285

PG.Genes	n	mean	sd	median	q1	q3	min	max
DPY19L2	25	760537	442176	734164	437681	958308	121886	1732865
CRACDL	24	22085	62745	9211	7704	12215	1788	316311
NIPAL1	25	27989	27855	23103	12815	31969	5995	147594
HIBCH	25	64424	190453	21798	14488	34536	2666	973408
ZNF774	21	9100	18194	5279	3432	6594	1003	87749
ANKRD54	25	62227	41143	60661	37728	78712	8351	202899
PPP1R18	24	43783	100070	21273	16775	29203	5760	510829
CAVIN1	25	17385	13630	13370	9180	23084	2661	63084
ZCCHC8	23	7733	5741	5763	3820	11014	1082	26450
DHX57	25	18264	26088	10211	8179	17151	4154	133426
MRPL54	21	3616	9042	1534	972	2515	518	42883
TATDN1	22	3338	2734	2577	2037	3032	1117	13656
METTL2B	24	2954	3142	1888	1314	2871	731	15404
C8orf82	20	9968	16750	5701	4501	8215	2245	80065
EDC4	25	206718	166286	181067	108262	246826	51507	931574
PRPF8	25	26650	19701	20801	18951	25722	15689	115842
NEK5	25	1423488	1150496	1080405	640297	2135532	53822	4547093
SCYL2	25	10117	2808	9796	8971	10679	5259	21252
TTC27	24	5157	2363	4428	3487	6489	2126	11339
PLBD1	23	112299	171105	16366	8109	217201	1614	610831
LMOD2	25	6048	7336	3068	2066	7149	1318	32378
NOTUM	24	27580	30948	15551	9784	22317	4507	125673
GIMAP6	23	3408	1816	2881	2052	5030	1041	6746
VWA1	21	9165	17390	4061	2698	7124	1141	82348
PGM2L1	22	3515	2157	2464	2132	4308	1515	10029
CTR9	25	41436	76379	24955	17103	28641	9562	401990

PG.Genes	n	mean	sd	median	q1	q3	min	max
TTC37	25	41454	32230	32115	22142	44080	8792	140540
DARS2	24	7764	8046	5422	3146	7728	1372	37097
FBXO46	23	12209	25680	5763	2989	8541	209	125881
LARP1	25	23462	18038	18000	12446	27522	8394	90106
ATAD2	25	198452	99932	168831	141358	210755	92261	534865
CPLX2	22	6450	4872	6160	2351	8374	463	20156
SLC25A47	24	10708	7654	7807	5629	12222	1064	30173
SPAG17	25	27525	19520	23083	14734	32740	5114	102753
SPATA8	21	2278	2133	1767	1168	2378	526	10486
MTHFD1L	25	22658	20935	14314	12593	19024	5426	102342
CSPG4	25	40664	22295	31193	25509	48434	18845	108391
DHRS11	25	20548	32894	13730	10381	16137	5153	173953
SBSN	25	37758	62036	16076	10056	37002	2843	299953
ENPP7	25	10055	6871	8808	6520	9832	3809	37681
OLFML1	25	32206	74485	13898	7959	25722	2358	384577
CWC27	22	22756	39943	10112	6229	19359	1569	190808
OLFM4	25	20219	35334	8499	6422	11428	4362	137275
LAYN	20	21760	21614	16184	8195	34958	961	83170
PLXDC2	25	69444	27878	65185	50202	80653	25153	147520
WDR82	19	7764	7002	5169	3716	8899	1660	29097
MUC6	24	17243	10530	14196	11722	19565	2826	50145
RAB11FIP1	25	9850	12128	6243	5084	8696	2309	64070
NAPRT	25	44972	14611	42357	37101	48598	27417	96055
GIGYF2	25	39138	47440	27719	21680	39988	13190	258455
CD109	23	3897	2695	3480	2239	4606	1250	14267
HSDL2	24	14588	19017	9826	7686	13331	3905	101227

PG.Genes	n	mean	sd	median	q1	q3	min	max
KYAT3	25	15037	6046	15152	10814	18963	2709	25222
THSD4	24	6675	10973	3810	3048	4934	2258	57062
TMPRSS11A	24	5704	3393	4397	2848	9099	1820	12081
ZNF783	25	20267	12746	17740	13485	21753	9004	74670
TRIM72	23	2336	925	2083	1738	2642	1340	5088
LEKR1	25	35522	99779	13665	9740	20189	5752	512620
ZNF782	25	11306	7242	9054	7436	14342	4952	41626
SPOCD1	25	7968	7407	5796	4535	8005	2230	39637
MEX3B	23	3587	1329	3247	2833	4238	1901	7405
BNC2	23	16271	13924	11638	8301	16353	3364	58643
GBP6	25	26692	36957	16113	8548	24928	1927	188113
CCDC81	24	4807	4145	3836	2700	4856	1696	21610
ZNF836	25	22267	18589	15567	7914	23506	5259	65063
RNF111	24	19674	24846	10381	6268	18334	2547	112970
FRRS1	25	156606	70219	143063	108663	178617	62641	361847
FGD5	25	15528	9981	12066	8460	20523	3974	40275
RBM44	24	15016	10701	11424	8048	15614	3940	41417
SLCO4C1	22	7187	6814	5512	4168	9089	1742	34630
WDR87	25	80748	30636	74385	65932	80617	49621	193030
CATSPERG	21	14415	15462	8977	5184	15958	1423	70253
FAM83H	25	318626	314359	229422	184883	305495	20864	1565944
LINC00696	25	66372	110667	25910	6121	46949	543	437109
ZNF662	25	93587	147267	65810	28076	118147	7845	768829
C1orf122	22	8038	5229	5941	4587	11839	1997	20001
LCNL1	25	10427	7101	9594	4945	12942	598	26340
CDHR3	25	63804	30708	55137	47770	64839	28440	153325

PG.Genes	n	mean	sd	median	q1	q3	min	max
CFAP47	25	937019	729875	713247	618619	978013	463839	4087969
MSANTD1	23	3826	1125	3877	3158	4234	1006	5816
UBN2	25	96243	87249	72314	48969	101343	35645	456335
FAM205A	25	18752	24434	10356	7621	17359	1550	112350
CEP128	24	5949	6610	3531	2665	5704	803	29667
MROH5	25	51077	41892	39097	31145	46952	15379	201518
SPATA31E1	25	60057	18278	58995	47155	74824	15343	93005
CCDC121	25	20897	21132	12123	9604	27284	2752	104312
GPRIN3	25	8097	8502	5453	4640	7288	2581	44298
CFAP20DC	21	5066	7036	3591	2588	4711	1075	34920
MICALCL	23	8826	10229	5929	4421	7523	1774	44664
TMPRSS11F	25	30392	37622	15035	12600	27523	4966	151191
USP31	25	19611	16963	13896	12007	22800	7109	94773
RAPH1	25	114473	55384	107588	91854	125872	44746	337068
USP43	24	37139	22958	30642	24372	45177	7898	126652
UBE2R2	25	28971	14051	28169	18631	35194	11377	63374
CBLL1	25	17089	34605	7903	6642	11826	4120	179621
FBN3	25	33097	13501	32112	22536	40700	14803	63955
MTSS2	24	11819	32056	5347	3380	7000	1300	161817
BRINP3	25	18502	13611	15879	9045	22218	3245	66532
SSH2	25	54773	100344	31656	27188	42037	11018	527873
CCDC80	21	11594	13352	7191	5422	11086	2496	64276
SUPT6H	25	32873	12278	30071	26813	33942	14825	67260
SND1	25	18577	12079	17212	9787	20872	5056	48202
DDX46	25	78349	125573	38404	32448	53245	20660	639004
TRIL	25	74304	82991	41136	25324	59117	4169	304497

PG.Genes	n	mean	sd	median	q1	q3	min	max
TRMT10C	25	11343	3891	11271	8687	12474	6441	19685
CHST9	24	7282	9792	3880	2224	6389	1092	37873
ASRGL1	25	89523	118087	78151	27539	103903	7657	619897
KCTD9	25	180017	226762	100071	47391	151983	24560	908330
EIF3M	23	12139	10874	8729	6177	12791	3334	53428
MEPCE	16	5511	4402	5391	2168	7543	666	18382
PARS2	25	12268	8605	10887	6283	16317	3315	41320
CYFIP1	25	9064	3500	8613	6758	11975	3433	16220
COPS6	22	4924	3072	4088	2657	6405	1654	14804
EPM2AIP1	25	12833	8872	12793	7046	16700	1250	34188
TAOK1	22	11049	4541	10239	7863	13244	5495	22824
KDM3B	19	2953	2094	2848	1670	3357	190	8829
CHMP1B	21	5761	6544	3926	1784	7958	890	29491
CHST3	25	10678	5319	9328	6589	15445	3138	24107
OTOP1	23	5025	8793	3121	2425	4232	1417	45069
MICAL3	25	6597	5571	4798	3609	7234	2098	29434
OVCH1	25	326322	124422	288810	250169	398869	67973	657353
OVCH2	20	8780	17313	3777	1164	7707	642	79004
ZC3HAV1	25	12788	8452	10675	7364	12976	4933	43332
GVINP1	25	8281	7749	5227	4067	8742	2905	35936
NEGR1	25	143954	85430	139716	92269	176549	12734	373297
NUP54	23	8087	12307	4981	2221	6311	1159	54745
DGLUCY	25	13920	6387	12181	10870	15649	6461	38403
CCDC186	25	38154	15747	33233	27816	47671	20160	83368
ZFYVE16	25	64354	102103	44877	30775	55077	15706	546955
MYH14	25	54655	32856	45377	38822	56658	35012	197815

PG.Genes	n	mean	sd	median	q1	q3	min	max
NUFIP2	25	28838	38284	21159	16288	29576	1546	206716
SZRD1	24	26722	31677	12953	7747	33394	2698	142117
MAVS	22	11142	19406	3956	2399	5864	495	71715
PKD1L3	25	203887	122374	190061	129541	258156	11588	593412
DHX29	25	53168	64176	36587	31701	44611	24017	345103
NPHP3	25	21768	17354	16174	10399	25333	2295	69512
HDDC2	17	8125	13746	3143	2238	7811	896	59755
POGLUT3	25	36395	116840	4976	4142	9868	2776	574167
LIMS2	21	11431	13905	4679	2070	16796	409	44994
DCXR	25	19033	17997	14516	12459	19074	10610	103096
AKNA	25	106839	116570	74414	61781	91070	40829	633058
TAFA1	24	77254	44429	59285	43990	121237	18500	147713
POLN	25	54015	48805	41325	30681	58782	13737	271674
PPP1R32	23	5609	4705	4362	2332	6439	1507	20192
SNX20	24	51948	58929	23746	15050	52008	7104	179878
SPRED2	24	22032	38106	13035	5419	20138	346	192199
ARPIN	25	18694	17516	11971	7471	26827	1730	80593
HUWE1	25	823057	227588	809646	607753	966691	530339	1432161
CTU1	25	32272	14536	29123	21573	40355	10205	66042
ABI3BP	25	15932	9044	15054	9689	18624	3212	46601
ZNF467	24	8917	12558	4831	3806	7902	1418	62151
C11orf96	16	3349	3882	2210	633	4542	3	13805
MEGF8	24	10346	4578	10036	6636	13682	3817	18249
GALNT5	25	57209	54976	44843	35859	52622	22064	299393
GALNT7	25	9858	9217	7896	5233	9013	2881	50431
PHLDB2	25	111615	62125	96273	76666	137936	14406	294142

PG.Genes	n	mean	sd	median	q1	q3	min	max
WDR86	24	17298	38990	6075	4434	9694	2125	193881
SETD3	18	9039	20892	3732	1906	7097	743	92060
TTC7B	25	31651	71663	15042	9599	23818	4706	372481
ADCK1	25	61340	26669	61680	38138	68323	25074	128535
NOP9	25	21192	56974	3814	2436	11229	1179	279570
METTL3	24	3443	2464	2639	1719	4346	1032	9689
PRPF39	25	122284	179958	52884	32500	166807	16207	897177
SAPCD2	22	9306	5523	7843	6372	11031	3681	28898
OAF	19	1937	2691	1279	813	1992	270	12661
ZNF546	25	14487	9853	12885	8557	17922	1111	39678
MTDH	25	9443	14285	6350	4881	8119	2572	77096
LRRTM1	22	5475	6157	3113	2150	6442	816	28853
ABCA12	25	23136	18895	17432	16834	21670	12647	110056
RTN4RL2	25	53391	38376	45278	35487	55328	8973	189764
SMG6	25	18846	25394	12707	9513	17013	4650	135683
BCL9L	25	16984	29887	8997	7542	11060	2794	148089
ITIH5	25	55550	35140	49230	27536	68654	20111	174585
FERMT3	23	5682	2941	5154	3405	7436	2377	12613
LUZP1	25	74837	72761	54307	40521	75788	27707	389311
ALYREF	22	11717	8096	9589	6870	12044	3733	38264
FAM160B2	19	9904	12510	4740	1603	15121	726	52219
VPS36	25	19025	26268	13274	12274	14392	4893	142643
CAND1	25	8572	4222	8701	4762	10541	3472	17213
TXNDC2	24	11252	11610	7772	4921	11519	2690	52364
HOOK3	15	2981	3389	1651	1176	3418	595	13937
COMMD7	24	21930	19192	16114	9511	24758	5439	92920

PG.Genes	n	mean	sd	median	q1	q3	min	max
METTL16	20	3522	6712	1663	1149	3414	278	31544
ZC3HC1	24	23792	16475	20281	13026	26879	4514	61402
PKHD1L1	25	10181	4797	10178	6936	12130	2865	24026
CCDC25	22	19577	22213	7716	3989	32842	1641	72914
CARM1	24	10471	13004	7366	5569	10048	2049	68193
CIR1	24	6808	6675	5463	2458	8605	1540	33844
FAM131B	15	2109	3359	1092	896	1460	436	13972
MICU3	25	38303	93365	19880	15150	23174	7444	485253
ZNF575	22	48663	57991	31702	23879	52298	11357	290062
PPP1R3B	25	17202	16437	12082	7951	24465	3777	84173
ZSWIM9	25	170884	97544	138008	112399	194251	90906	563315
ANKLE2	23	20204	15318	16143	9329	26763	540	62799
DZIP3	25	21429	74037	4276	2724	5652	1315	370925
ZGRF1	25	204925	192005	156461	128357	208951	70348	1084481
ERO1B	24	11503	10570	8201	6331	11901	2928	52875
ZNF280B	21	21260	16117	16745	12177	22968	764	60222
MARCHF9	22	21484	18600	12243	7859	33961	3217	71830
ANKRD13B	25	112989	232673	43648	22750	103609	8607	1178662
TMPRSS6	24	6804	5929	5319	3778	6822	2383	28113
IRF2BP1	25	77933	50752	63441	45726	124740	9287	178840
RHPN2	25	11522	5554	10395	6966	13616	5010	27019
TGIF2LX	18	3863	3249	3243	2443	4125	927	15878
H2AC21	17	4463	8216	1918	1092	5339	249	35376
AEBP1	25	43154	74439	25828	15390	39594	5035	385156
PHYKPL	25	77721	78623	55469	43241	81635	26902	428844
PLD3	23	3166	1572	2891	2035	4256	730	6844

PG.Genes	n	mean	sd	median	q1	q3	min	max
LIX1L	24	24707	31369	12783	10073	20814	4767	150739
ZNF584	25	317660	362811	217374	184780	338741	35097	1934862
NUDCD3	25	10921	11911	7483	5674	9812	3611	52062
P3H2	24	21076	24730	13770	9298	21612	4389	128366
P3H3	25	22970	43328	12711	9774	17393	2280	227857
MISP	25	4477	6707	2642	1904	4623	901	35686
IGDCC3	25	254326	354117	163241	92041	290056	51512	1857805
CDKL3	24	6253	12035	2793	1626	3895	630	58534
APLF	25	10217	9291	7713	4333	10858	1020	41678
MAPKAPK5	25	62717	20557	59410	50662	77180	27248	103556
NAXD	23	3314	1358	3066	2220	3694	1375	6417
NAXD	15	3475	2214	2630	1879	4453	1115	8391
TEX2	25	31845	31680	24274	21241	29954	7520	179259
FAM114A1	22	3776	4304	2889	1678	4079	699	21754
GCC2	25	83746	56593	83250	48364	92330	13013	304251
HSCB	25	70522	90457	39370	28244	47783	10579	341921
CUL9	25	181316	108591	150024	107012	218545	86005	549057
LMTK2	24	17115	8503	15852	13376	19351	4498	48587
SULF1	21	2952	5003	1377	1044	2548	280	23689
UBR1	25	11766	8723	10154	6799	11900	5160	39181
SCUBE1	22	10319	9555	8756	5156	11483	1411	47521
TRIM42	22	3961	5177	2869	2069	3784	864	26377
SUGP1	25	51928	43476	42698	35685	52738	28585	254889
UEVLD	25	15770	15665	10727	8551	16376	5775	82781
CCAR1	25	12725	5315	11059	9218	13424	6521	25000
DNAJC10	25	22286	21972	15212	10444	27684	2725	107758

PG.Genes	n	mean	sd	median	q1	q3	min	max
ASXL1	22	32729	26295	26268	12519	48689	3308	91895
PHC2	21	23677	21396	14918	11320	30323	2351	74973
MSRB3	23	40365	57901	10508	6854	41042	1250	191325
IQCF2	25	63495	52750	52595	44167	70357	12280	298218
MRPL41	22	18458	21614	11587	7492	19959	2650	99428
GPALPP1	25	11878	11789	7935	6453	9478	4179	55729
ETFBKMT	25	6446618	5365359	5414732	3130690	9986306	61445	22328706
FAM217A	24	32833	39307	18741	13838	35740	6890	193441
RBM12B	25	221861	133848	202015	121667	278255	46876	555534
ZC3H3	24	10353	13464	6632	4031	10360	2301	69300
DDX60	25	18045	13397	15435	10669	17969	4972	65742
MICALL2	25	8406	3162	8658	5515	10328	4032	16698
RAVER1	16	3949	4994	2198	1382	3710	297	18871
FTSJ3	24	13566	23089	8477	6425	10832	1062	119428
MKX	24	27278	54155	12706	9762	18954	4708	269234
DIS3L2	24	12136	7890	11162	8625	13076	6297	47284
ZNF595	24	4434	4974	3413	2693	4600	897	26636
LAS2	22	16380	10315	14194	9564	18727	4555	48688
EXOC8	25	35896	62306	21591	15298	29798	6236	327518
TRMT44	23	3158	3610	2287	1453	3502	688	18720
THNSL1	23	6749	5961	4462	3336	7948	2472	29873
CFAP206	25	34247	21353	28523	19483	42079	8575	97662
HACE1	25	220790	128740	192601	143861	283177	77319	710105
MICU2	22	8663	4934	7979	5189	10435	2052	21722
CHPF	23	12930	10067	9605	7703	14538	5500	52257
NRSN1	25	13212	10118	8894	4723	19429	1580	33877

PG.Genes	n	mean	sd	median	q1	q3	min	max
RTKN2	23	46390	93789	9606	6883	13909	2778	377191
ADGRF3	22	15930	23783	10376	7605	16200	1853	118644
ZNF654	18	5646	7269	3056	1995	5811	985	32255
C1orf87	22	13602	7597	13236	7321	19323	1980	29106
SPART	25	14335	29168	7059	5636	9749	3798	150890
PGAM4	24	10215	10831	6401	4463	9579	795	44994
ADSS1	23	10276	7315	9463	5394	12373	2859	31067
AHI1	25	19010	27639	12684	10416	16332	7527	148351
NAGS	24	5156	5772	3603	2695	5760	1312	30609
ZNF567	21	6825	5872	5220	2803	8088	78	19792
CARF	24	99039	268654	33041	24571	64315	4394	1351514
STK11IP	25	18375	11163	15467	11308	21580	2495	51489
LRRC47	24	11884	27755	4926	3995	8267	2465	140824
CRYBG2	25	190193	67262	187097	146819	204902	110764	402546
VWDE	25	16060	9981	13850	8882	22659	3651	41910
ZCCHC24	23	11108	6558	7815	6571	14813	3547	32005
GHDC	25	29173	19443	25253	16462	42521	4255	68922
OSR2	25	17601	37401	6845	4820	11977	2041	188117
LTBP4	24	7236	8535	3686	2395	9169	1567	39165
PIAS4	25	478139	253134	393745	321875	563844	212200	1265340
GPD1L	25	66826	96201	48088	30237	67720	16160	517368
EHBP1L1	25	1081938	1072698	804236	592177	1166111	422590	5906238
MICALL1	19	6411	6929	4288	2130	6953	1150	26193
TXLNB	24	35338	39042	27469	15439	35674	4987	202428
PHTF2	21	111158	487794	3823	2330	7443	870	2240010
FNBP4	23	7609	5905	5558	4029	8347	1383	23947

PG.Genes	n	mean	sd	median	q1	q3	min	max
GUF1	24	38287	37291	19486	12099	57221	2867	150997
RIBC1	24	5739	3151	4687	2853	8547	1553	12063
SFRP1	21	16216	12865	13817	4404	24354	1062	39035
LIX1	25	4569	3754	3744	2328	4839	565	18716
PNKD	16	2443	1265	2144	1538	3240	194	4965
GALNT4	25	34331	38593	17584	13663	35066	3952	162114
MARVELD2	25	139841	87025	116150	89055	153626	58541	468810
CPA6	25	10046	10722	6510	4089	10964	1107	49780
CBR4	20	7839	5394	6002	3658	11565	1338	20352
AFAP1L2	25	206222	211374	147016	63210	218947	27426	768184
TTC39C	25	19095	10395	16530	13486	21385	5708	49010
FAM89B	18	61385	52491	49938	22434	84525	3245	177094
ARRDC1	25	4873	2526	4165	3573	5549	1591	13167
SLC25A41	18	26953	57020	11841	8366	21286	195	252817
CPSF7	24	27034	26020	17254	13603	31592	2336	117471
ESX1	20	6935	7526	4398	2999	6430	925	33739
ARFGAP2	24	63222	149442	12136	7110	43100	3901	686555
OTUD6B	22	16219	29992	8980	6709	13377	1991	148500
SIRT6	25	34509	27050	33767	15630	40127	9054	141606
CCDC185	25	47260	21478	43871	32465	59753	15644	100000
PAF1	24	99579	100527	48753	28714	134883	6991	348589
PAF1	24	15081	12980	12743	8648	16145	5005	70148
ZNF283	25	19219726	55317466	5547709	3096851	9549393	356988	281816032
ADGB	25	42659	59833	28782	20228	35663	13792	319523
ANKRD31	25	8339	3675	7267	6171	10383	2746	19645
UBR7	20	3532	2804	2941	2024	3375	1262	13399

PG.Genes	n	mean	sd	median	q1	q3	min	max
ZNF614	25	24890	12462	19845	17319	32047	13212	71874
DDX51	25	9792	6837	8809	5512	12150	915	35058
KIAA1958	25	25614	52353	14930	10952	18986	4988	273972
PTGR2	23	6905	5988	4819	3481	7239	1836	26443
SLC25A29	23	36034	117279	12109	5138	17573	3142	572800
PNPLA1	25	16133	14569	10164	8686	19977	4268	74318
MAB21L3	20	48562	90647	26201	17270	41262	5388	427243
ANKFN1	18	6329	4740	4935	2836	7272	1498	16249
ZNF709	24	60605	64918	33308	21614	77261	7225	285624
IGSF22	25	43950	15676	41909	31831	49692	21557	97579
DTX3	22	6648	11963	2390	1759	5491	965	57197
ASCC1	25	1418474	863588	1321422	1082795	1661626	390397	4912804
FSIP1	25	138456	186594	83120	58906	143790	38117	972731
WDR31	21	16643	19500	10368	6070	19118	915	86267
C12orf50	21	25087	25091	17803	9459	34256	745	86370
TEX45	25	34366	19268	30555	19980	44613	6198	72655
FAM47B	24	19713	20650	13054	9593	19540	7662	107716
MARCHF10	24	4162	2921	3571	1980	4393	1451	13301
ZBTB38	24	4253	5311	2662	1957	4658	826	28038
PRPF38A	20	9621	10236	6649	4375	10181	1982	49175
KDF1	25	65265	85563	36745	22366	64475	4455	410780
MLKL	25	35128	18994	31187	23697	45864	9409	95038
NHLRC2	24	4854	5409	3775	2327	4718	1495	28480
AVL9	25	9423	5473	8439	5423	11424	2356	28450
COLGALT1	24	10707	8685	8729	3256	17588	981	31253
POGLUT1	25	490763	543199	302294	241679	527606	49373	2656765

PG.Genes	n	mean	sd	median	q1	q3	min	max
SLC4A11	25	34685	22107	32732	20271	40332	7260	114880
TXNDC5	20	7258	5227	5822	3073	11165	1020	17849
RNF149	25	85256	222926	39288	28173	49750	10219	1152257
NOA1	25	46750	18505	47964	32623	56689	15245	96008
MTMR14	24	9738	8464	8710	4632	12829	2116	44070
NFATC2IP	25	6570	6412	4923	3662	7198	2906	35826
GALNT6	24	31935	24884	22979	16021	34460	10955	112800
NAXE	25	96891	62930	78887	44104	146518	12501	239608
CARMIL3	25	76597	140507	39751	24626	78211	4389	725800
RNF214	24	44347	68142	23196	12376	38454	1222	320023
LSM14A	24	14747	32839	7685	4711	11473	2259	167318
C3orf20	25	19861	11718	18439	11343	24437	5860	57068
MROH1	25	10394	6396	8750	6815	10301	5155	36977
TNRC6A	25	54167	30838	42448	37460	54455	28660	150838
ZNF738	24	16971	15144	12321	9677	22049	4795	79382
ABCF1	25	9607	9198	6920	4934	8701	4355	45616
ACRBP	20	9422	8798	6783	3973	12113	1180	39154
PIK3C3	25	446092	237196	412363	328740	477159	224438	1445712
CATSPER1	23	31073	28402	23887	9509	41684	2916	117457
TTC16	25	27940	15594	26317	15069	36026	8672	75750
NGDN	25	137484	84513	149937	57125	195332	1319	267667
RTL9	25	68787	65433	58636	45218	64330	31457	376943
SDR9C7	25	36967	74045	15072	10525	21283	5699	364658
FBXO22	21	6414	7682	4375	2326	6396	514	34145
BOD1L1	25	1063984	1164115	877232	723419	946943	438965	6554891
ANKK1	25	57848	49956	34588	26147	75391	7181	184983

PG.Genes	n	mean	sd	median	q1	q3	min	max
NUP35	23	3833	3139	3045	2223	4153	1073	16294
REPS2	20	3273	2343	2422	1888	4356	335	8181
MDGA1	24	4039	3050	3207	2525	4255	799	16278
NBEA	25	37271	19313	32422	22947	47069	12673	101036
CCDC148	24	1226	726	1049	771	1388	477	3498
TSTD1	15	3983	2673	3025	1693	5921	632	8747
TSTD1	24	21641	12961	18856	13777	31830	3393	45362
CMAS	22	2173	1203	2091	1158	2822	615	4826
TRIM58	22	24341	30967	8831	6329	24670	573	101407
KNL1	25	47022	44332	38273	30022	51891	21507	251038
OR56B2P	21	10244	7819	7208	5683	13470	2846	39701
OR13C9	24	11627	11761	7700	3977	16422	613	53032
OR6N2	24	33647	19194	28750	18966	41746	12043	80556
OR2T3	22	15941	14909	11437	4920	20885	2281	60639
OR51A7	23	17681	12760	14917	10924	20043	6841	69965
AKR7L	23	9649	4782	9133	5472	12848	2826	21114
PLBD2	24	25872	27379	15552	10246	26713	2237	112734
SPATA22	24	5636	4776	4560	2745	5770	1351	23848
TDRD7	25	1244703	878060	1167796	741106	1343761	117373	4480724
GIMAP7	24	124420	124166	100161	27043	144943	3230	477257
GAS2L2	25	7518	11239	4530	4056	5995	2407	60133
WDR36	25	34142	16312	29317	23258	38114	14159	72527
COQ8A	17	10119	15754	3922	3607	5043	1284	60529
SLC30A5	24	4678	4767	3855	2776	4512	1827	26288
GADD45GIP1	25	134482	90866	93039	79010	150827	50267	411657
WDR48	25	27817	15183	22583	19130	32279	14349	89126

PG.Genes	n	mean	sd	median	q1	q3	min	max
SMARCC2	25	36545	45762	21072	17815	40091	8816	223536
NPLOC4	25	259616	432500	132529	92011	208697	52116	2128335
NA	22	15627	18845	9353	4988	17531	2209	84129
ZNF519	23	5458	3633	4138	3373	7367	459	16402
PIGX	23	4085	1719	3938	3296	4808	1189	8238
CIRBP-AS1	25	4862	3989	3611	2432	5736	1076	18832
MDM1	24	7353	14844	3494	2432	5004	664	74183
FAM71B	25	8553	5447	7453	6453	9743	489	29902
ACTL9	25	3824	1881	3479	3048	5449	372	6856
NT5C	15	3690	2106	2951	2080	4422	1514	7976
AGR3	23	16461	32785	8706	5085	11395	1250	161487
NEK9	25	12955	12341	10236	5382	13512	2073	58228
ZNF675	24	10094	25488	3550	2524	5620	1256	127334
GPT2	18	2983	4797	1697	1333	2594	504	21715
PLEKHO2	25	41597	32118	34258	22664	49766	13974	177849
MAGEE2	24	9576	16258	6771	3968	8356	1558	84800
BPIFB1	24	8647	6638	7133	2678	13757	786	24338
BRIX1	16	2000	1612	1430	1064	2257	450	5726
RNASEH2C	18	15099	19527	5800	805	18667	207	62786
GNPDA2	22	4781	4593	2974	2018	6010	592	17080
FAT3	25	24518	22277	17093	14797	21461	11399	103531
NEK7	24	5362	5574	3583	2909	5395	897	28699
IGDCC4	24	12450	25946	4242	3642	6216	2525	129075
MICAL1	25	40716	76470	20013	13733	27082	8058	347076
PANK1	22	2227	1581	2148	908	2790	344	6973
TAS1R2	25	11813	4926	11146	9088	14085	5076	27477

PG.Genes	n	mean	sd	median	q1	q3	min	max
ADAMTS17	25	27041	62721	12960	7162	18601	3623	324476
ADAMTS15	24	14816	13173	8688	6452	17052	4397	54351
EPS8L1	24	9614	9198	7552	6084	9442	4018	51000
DNAH5	25	88288	87522	60610	52069	86948	34452	485407
SH3TC1	25	29567	23301	23511	15382	33536	6933	116069
NSUN6	25	27831	17293	29139	16633	34163	0	80967
TBCK	22	13593	8474	11127	8864	16350	4086	41902
DTD1	24	17849	8755	16681	12954	19403	7116	45507
IPO4	20	11054	8204	9545	6324	12262	3327	39668
PNISR	25	15295	25327	8737	5385	15536	1601	132966
DYNLRB2	25	21909	22086	12460	9050	21392	3159	83615
WHAMM	25	115584	107732	89255	63453	113102	47123	583001
ZNF483	23	10131	9638	5390	4042	11730	1731	37584
FNIP1	25	79794	33132	69107	56731	92723	37151	166942
SHROOM3	25	24444	14812	20092	15152	32286	7861	73588
SETD7	20	4972	4991	2844	1449	6081	727	18266
C7orf33	22	1700	1764	1002	721	2045	255	7647
SCFD2	25	107688	51816	103032	85882	128289	23488	241719
ZC3H15	25	121396	229934	47251	34717	85861	6436	1128179
PPIL4	22	4329	1896	3996	2886	5040	1968	9862
PPP1R13L	25	72193	59540	48520	40612	82670	14323	256347
TGFBRAP1	23	10516	15250	6935	4743	10573	1300	78090
NUP133	25	16210	12554	11223	9294	15706	5774	59453
PDCD6IP	25	40928	27967	34243	24426	45690	15299	140785
FBLIM1	25	84920	109300	51354	13926	102340	268	458932
SDR42E1	24	5747	4934	4334	3218	6135	1408	25272

PG.Genes	n	mean	sd	median	q1	q3	min	max
DEPDC1B	25	58294	36725	58631	30422	73235	7554	151088
LTO1	21	25530	45536	13895	7871	24545	1486	217265
PTCD2	18	2661	2066	1962	1269	3625	209	8815
AFG1L	25	154630	102289	140121	100802	195746	11437	439839
OSCP1	25	63016	50635	38655	27507	101005	14114	193035
NUDCD2	21	1736	1019	1647	887	2133	492	4204
SCFD1	25	388229	207059	330246	280976	404582	163202	1063327
C4orf3	21	6929	12713	4007	2455	5048	1199	61520
UBLCP1	24	6803	4529	6110	3846	8620	1162	21761
TEKT4	25	25086	14420	22077	17294	27175	5875	68000
ZFPM2	25	25953	44021	16785	9323	22066	5453	231707
SPRYD4	19	2180	1677	1876	988	2373	272	6752
CAPSL	22	13177	15074	6353	4311	13470	923	56709
CYGB	19	2842	2286	2686	963	3673	113	7837
PHIP	25	161115	67152	148307	120790	181182	82130	399376
PRPF31	24	20101	19821	12937	5033	29694	1058	71282
PALLD	25	9603	5718	7891	5601	12808	2517	22980
SREK1	21	3251	3097	1973	1292	3978	657	14107
COPS9	24	10461	18206	3126	2038	7038	1019	79244
SCG3	22	9100	25488	3580	979	6217	101	122349
SRSF12	18	2174	1283	1979	1331	2976	400	5011
PSPC1	24	1865	1135	1435	957	2322	533	4515
RSAD2	25	16605	8127	13699	11807	21077	3021	35922
JPH3	21	5344	3018	4858	3839	5297	2730	16757
MUC16	25	312196	269511	230394	212208	273925	171779	1424097
DNAJC9	20	2635	2510	1796	1031	2955	570	9726

PG.Genes	n	mean	sd	median	q1	q3	min	max
THAP4	24	8636	8755	5978	4310	9507	2226	42285
TTN	16	45779	30836	40291	21421	65990	6436	109364
LZIC	25	8698	3192	8299	6269	10689	4435	16417
IRGQ	25	9903	3222	9828	6855	12401	5271	15929
ST8SIA2	24	7340	12271	4697	3448	5793	2377	64204
DDB2	25	67803	57268	50495	37774	77215	18165	312586
SMPDL3B	22	16811	28628	11577	6675	14940	3078	142945
DDX1	25	17672	6310	16111	14609	21096	6692	33523
HSD17B8	22	2828	1388	2778	1920	3697	598	5767
PIEZO1	21	3059	2642	2715	2011	3382	647	13479
FAM3C	24	21197	11057	18697	14073	25381	4262	45806
H1-10	24	7982	9076	5902	3903	8155	1467	48257
PSMF1	23	5370	1913	5342	4053	6500	1601	9645
GBF1	23	17825	62438	4196	3065	6715	1655	304027
NCSTN	25	17330	14791	13603	10192	16517	6543	80949
MRPS27	24	7234	4947	5850	4490	7930	2376	24445
ELMO1	24	22560	8282	21070	17716	24642	5411	40294
FIG4	22	23155	21036	17739	11657	25574	5260	106808
DCUN1D4	20	2241	1351	1851	1308	3195	673	6275
AP3S1	22	7726	7054	5055	3471	11626	636	28647
UBXN4	15	2219	2530	1250	757	2316	462	9469
DOCK2	25	28941	26025	21554	15653	32137	5556	138721
TBC1D5	24	8170	5525	6603	5627	8729	2888	23061
LARP4B	23	35294	23577	29809	24147	36916	5984	110239
GCN1	25	171782	99746	145359	128187	175450	64554	580180
ARHGAP45	23	6737	4426	6196	3709	8382	1922	21922

PG.Genes	n	mean	sd	median	q1	q3	min	max
DHX38	25	25573	39898	10078	8101	21315	5936	166104
NUP205	25	15040	4955	15280	11693	16676	6226	26129
TTC9	25	91514	133760	60675	38381	91737	5672	706437
PXDN	25	49863	25386	43911	35364	68632	12924	130556
PIGK	23	8625	10664	6088	4016	8399	2541	55128
GTF3A	24	17349	34921	7596	6178	10520	1806	171169
SORL1	25	44701	33957	33704	29470	40214	19793	178381
ANP32B	19	4230	2756	3626	1888	5640	1064	11061
RABGGTA	20	2054	1120	1749	1499	2203	394	4730
USP6NL	25	109568	414036	19866	17100	24446	11101	2093740
HTRA1	24	3675	3552	2821	1708	4112	686	15948
ARPC1A	19	4043	2630	3857	2058	5736	705	11224
ARPC1A	19	4546	3807	3142	1633	5750	612	11798
TAF4B	25	80375	89275	40476	27573	92949	12548	432724
HDAC2	25	39006	21688	35865	20704	50488	10801	100237
DPF1	25	62924	98466	41710	30743	50021	24236	530197
STAM	23	7739	8811	6213	4685	7452	2650	47282
PROX1	25	34959	15768	32542	27112	41450	14277	90065
SYMPK	25	14429	11762	10037	7594	17740	5825	61289
SYMPK	19	4746	6651	2832	1580	4431	139	30332
TAF15	23	11962	8881	9012	6128	13893	3373	43413
GGH	22	3886	3703	2756	1328	5088	389	13299
DDX17	23	10546	8438	7576	4930	13872	1063	30790
APBB2	24	4174	1483	3807	3201	5276	2101	8461
OSTF1	23	6163	3103	5668	3791	7901	1855	14787
ABCC2	25	64913	27193	64447	44824	80222	27406	138235

PG.Genes	n	mean	sd	median	q1	q3	min	max
ERCC4	25	123053	185469	89043	46832	130032	19470	983738
UFD1	15	3385	2929	2744	1555	3718	495	11127
RPL3L	25	24736	13626	21821	13747	27634	9062	63860
COPS5	23	15204	38727	6250	5317	9529	1834	192069
GPKOW	24	6269	2870	5557	4624	7571	1071	12759
SMARCC1	24	235843	1091027	11104	8593	15066	4142	5357893
RAB8B	25	114582	152732	68330	43401	108474	75	733970
KHSRP	25	26852	29983	14888	10183	33200	3486	129549
KCNB2	24	50456	38353	41239	29369	58959	9840	203135
GLMN	23	10235	5942	8062	5467	14348	2727	24014
USP9X	25	28624	13664	26370	21852	31295	16138	85765
USP7	25	14943	24115	7549	5627	9318	1711	96530
CUL5	25	10583	12444	6473	5353	9342	2829	64804
LPP	25	24995	27788	18070	7119	30414	2665	131151
RBPMS	20	2399	1737	1837	1021	3529	318	6139
HGD	23	3851	4441	2107	1432	3780	772	19407
MR1	16	11943	10321	7429	5583	14616	2160	40353
TCEAL3	18	8700	12452	5751	1966	8851	1155	52885
CAVIN3	23	16165	12339	14063	6794	21687	1254	43117
NKD1	21	2220	1827	1673	1184	2413	399	8117
CNKSR1	25	10239	4243	11020	8198	12882	1247	19025
MYDGF	25	17472	14376	12028	6066	27593	2658	50446
OSBP2	25	12123	20523	3747	2519	5120	1711	78144
WBP2	22	2895	1721	2387	1853	3694	1003	7317
NCLN	25	55179	86801	32810	26360	40310	14899	452535
NXPE3	24	36106	79689	20065	11590	28599	5434	406809

PG.Genes	n	mean	sd	median	q1	q3	min	max
MYLPF	25	66905	83646	40725	22045	87600	4092	390155
SYAP1	20	10404	13643	7139	3414	11387	958	64814
EXOC4	25	29370	17865	25415	19858	29139	11943	94156
FUBP1	25	16115	15140	9634	6646	19499	4264	63493
TTC17	25	79828	166568	27231	15565	35750	7490	754337
LRRC59	18	5079	7592	2822	1908	4206	549	32947
CLUAP1	25	19699	21039	13019	11147	16468	8705	89788
ESAM	19	2230	2936	1099	829	2013	416	12478
FKBP10	25	115651	51120	106104	81387	150555	29620	239179
TMEM186	25	1323752	1489972	862963	202422	2043598	34279	5611827
TMIGD2	24	16117	12565	11746	5208	22098	703	43207
RNF25	25	8830	11463	4117	2985	8430	1400	55805
AIDA	23	6677	12470	3561	1500	5600	1028	61933
ARL8A	23	13463	9849	9839	6326	18576	5011	40401
CHCHD1	25	60620	40129	53128	35954	81749	8220	154456
PPWD1	25	47622	53820	32315	23252	40183	6808	257591
COA7	23	4473	5145	3425	2096	4889	844	25487
PTER	16	1237	674	1303	667	1687	425	2534
MOB3A	23	5475	9841	3047	1806	3964	716	48880
LENG1	24	9794	3919	10845	7124	12714	3297	16685
FAM136A	16	1768	1379	1207	819	2556	480	5445
MIEF2	25	10334	16930	6997	3925	8437	1778	89050
DHX58	20	4815	9268	2523	1666	3709	982	43587
EFHD2	25	18254	11211	13446	11657	22476	2877	57110
GALM	24	8582	20331	3991	2979	6692	826	103366
SYTL4	22	8422	7160	6849	4584	9176	2625	37120

PG.Genes	n	mean	sd	median	q1	q3	min	max
ULK4	24	18884	19203	14468	10533	21335	6082	104555
DCPS	23	15147	9725	13499	8440	20195	1032	45156
PPP1R14B	15	4595	4653	2844	1523	5179	549	16924
ZNF653	24	168867	369012	89377	47972	135683	1219	1870533
NXNL1	24	65240	62449	49010	40442	69352	21822	345650
ISOC1	25	25076	28069	14957	7384	32703	2596	125523
GCC1	25	21730	16790	15137	11216	23000	7157	72020
CCDC124	19	6214	4677	6280	1999	8745	311	15612
AP2M1	25	31913	120591	6013	3917	9738	3325	610174
KCTD12	25	10601	9115	7851	4120	14963	1059	35179
COQ8B	25	111995	56305	87799	73492	127802	39978	250492
ZG16B	24	8923	7888	6413	4023	11020	1744	37072
RMDN1	19	3589	4327	2618	1569	3660	494	20525
LRRC39	17	8844	8846	6962	3473	9931	278	37847
CMBL	23	6378	3469	6265	3386	8192	2096	14348
RMC1	25	52891	27371	50677	40770	62326	6798	154854
ARHGEF26	25	58219	55708	40845	37225	51699	18457	267407
ATG4C	21	12086	19425	5708	3396	14701	2361	93418
ITPKC	22	9902	8572	6790	4072	12999	3051	39116
IQCD	25	11024	16761	6812	5016	10159	2617	89066
RBMXL1	23	18333	26151	8635	3382	19276	346	113002
SIRT1	25	7483	5165	6509	2817	12727	663	16286
HOOK2	24	130622	132526	100162	85782	126921	12628	725870
SEH1L	21	2835	1279	2894	1897	3615	912	5520
TCEAL4	19	3712	2238	2965	2189	4158	1190	9239
GNPNAT1	15	2808	1919	2311	1428	3803	578	6651

PG.Genes	n	mean	sd	median	q1	q3	min	max
INKA1	16	3191	1512	3171	1775	4498	1392	5306
MOCOS	24	13661	9442	10292	7467	15795	5537	48691
DAZAP1	21	2531	2197	2289	837	3775	285	8071
SAAL1	18	3913	4366	2633	1311	3657	714	17494
CCDC51	25	21390	10679	19381	15192	24022	8611	58800
RBM33	23	4937	5573	3893	2157	5543	825	28675
MMAB	20	4470	4231	3119	2220	3837	1370	16857
ADAT3	15	11391	12193	5676	2904	16058	955	37880
CCDC97	25	76241	156739	37813	20477	57049	8481	803893
DISP1	25	59162	71724	34596	23572	51762	17264	354912
CNRIP1	24	12686	47786	2665	2220	3238	1197	236926
PHYHIPL	24	31236	38333	16891	3774	35275	1670	126375
WDR89	25	6727	3430	6557	4460	8453	1147	16318
S100A16	20	5830	5398	4140	2064	7578	596	23857
SIPA1	25	9819	12764	6484	3813	10844	2041	66504
LRRC46	15	3085	2108	2614	1119	5073	419	6566
THOC1	24	22957	62525	3389	1780	7144	187	294593
OTUB1	25	22425	16438	13539	11777	31342	9540	84726
TRMT61A	22	5256	2666	4280	3743	6015	1276	11642
HMCES	25	20762	11869	15277	13431	21211	9756	55705
PGM2	25	16834	12935	11044	7705	22033	4582	50108
KLHDC7B	25	264707	772446	47201	19617	134518	4136	3895234
DUS3L	23	2554	2046	2383	1026	2905	512	9916
PDXP	17	926	399	895	592	1119	474	1929
DCUN1D1	23	5148	2769	4527	4065	5259	2285	16151
FAHD2A	23	21529	36891	10987	9710	18699	5036	188361

PG.Genes	n	mean	sd	median	q1	q3	min	max
APIP	25	34535	32943	25969	7168	46119	2099	118032
ZC2HC1A	21	11428	28829	5703	3249	7706	976	136858
SNF8	24	16621	18179	14271	8144	16594	4341	98737
ZC3HAV1L	24	7270	3559	6345	5072	10268	727	13232
PDLIM5	16	10245	15187	5895	3136	12678	313	63904
PDLIM5	23	5029	4426	3388	1668	6980	501	15748
ACY3	22	1641	1108	1303	1046	1959	458	5883
ERO1A	25	139665	100338	102442	65327	189584	8103	399169
PRR11	23	9528	25018	4163	2385	5681	1198	123305
FMC1	22	19764	47140	7079	5955	12894	4041	229057
OXNAD1	25	15857	10266	13145	8363	20927	1097	47767
DIRAS2	22	8330	12834	5663	2421	7792	1496	63630
INTS4	24	99386	104562	70546	53430	109132	21664	560318
DDRGK1	21	2806	2146	2218	1108	3947	538	7995
FUBP3	21	11179	26248	4608	3473	7606	1281	124875
RBM17	25	23840	12103	23307	17440	28682	2734	53148
NARS2	24	5234	5906	4128	1569	5564	659	27809
ABHD14B	24	14279	10282	12704	7636	16095	1354	49495
NGLY1	25	65675	76148	43957	23056	86161	5043	383803
FAXDC2	23	14403	12958	11379	6917	15168	1205	61155
CPB2	22	4031	1992	3588	2569	5015	1810	9271
PAWR	18	6513	2825	6461	3976	8612	2470	12460
THOC3	25	49183	69060	32619	23554	49205	14429	369273
HIC2	25	3445155	2370631	3028692	1745708	5008239	65452	9864547
CDK5RAP3	20	3138	1719	2614	1704	4897	762	6051
ZNF333	25	22672	18242	16719	11106	28579	2721	76156

PG.Genes	n	mean	sd	median	q1	q3	min	max
CHAMP1	25	4130	2896	3346	2291	4692	458	14802
MYO15B	25	8265	11246	5467	3731	8070	1427	60063
DCHS1	25	17621	16270	14173	10417	16893	8217	91866
CLMN	23	6052	4686	4179	3432	7491	894	20547
PDLIM2	24	6912	5291	5640	3680	7632	1610	25528
BTF3L4	22	5682	5028	4338	2412	6893	902	21673
DNAJC1	21	5321	4427	3833	2903	6976	664	20361
BTBD6	19	2128	898	1865	1444	2839	972	4056
ZNF512B	25	148991	90875	112042	91752	177025	54923	380030
LRATD2	23	7641	5204	6322	4607	10221	666	18169
CNDP1	24	28654	17852	25946	15705	41039	4623	73150
RPGRIP1	25	37492	59483	24528	18136	27419	12467	314500
CNDP2	24	36574	34273	19970	11612	44880	2625	120587
ZFR	23	3878	1721	3821	2662	5265	771	7985
WDR90	25	10316	6720	8932	7285	10914	4356	37664
CAPZA3	22	18913	11520	15435	10564	25641	1565	43277
KCNH8	25	112403	70208	95562	64842	117458	23152	327408
CAPNS2	24	19957	12253	17220	9177	27510	6377	51626
FCRL2	22	4713	4250	3464	2057	5281	210	16046
PRMT6	25	7387	8704	5894	4660	7153	204	47853
TRIM47	23	71164	23891	65394	55106	86013	41279	143049
SENP8	24	12488	14322	9367	5136	14746	2053	72739
CXorf58	19	7819	6211	5767	3680	9652	1559	26996
C4orf45	25	32915	17699	32550	22273	39717	9221	101127
C12orf42	25	6872	4298	5364	4492	7365	2268	21862
PPP3R2	22	108696	259000	49615	28465	80252	3451	1259024

PG.Genes	n	mean	sd	median	q1	q3	min	max
TEX55	23	5908	4875	4416	3095	7816	247	22692
TBATA	20	2318	2869	1586	830	2189	136	12258
NA	15	3375	3674	1923	951	4072	388	12851
CCDC7	25	9985	13365	6194	4725	9348	2976	71516
DRC1	25	30085	17100	28299	17376	36885	1129	66230
MFSD14A	19	48431	103701	18167	5426	29693	429	451757
C5orf34	20	9287	6793	8311	4861	12984	368	24388
COG8	25	9915	10984	6013	3888	11420	554	53896
TTC14	25	14755	21614	7692	6338	11208	2905	88337
PWWP2A	24	14913	28690	6589	5936	10322	3252	145198
RILP	15	29361	25525	17144	12865	51720	1067	73304
SCLT1	25	85083	57240	79897	45926	101903	26814	304759
WWC2-AS2	20	64711	185390	20004	16103	30433	3600	850015
SLC46A1	22	5339	7697	3246	2307	5725	597	38354
CLIC6	25	6036	8265	4070	1569	6868	20	40512
FOXN4	23	8589	7732	6666	5585	9301	981	38840
IPO9	22	7884	21162	3298	1588	5749	309	102074
ARHGEF17	25	32307	17223	26644	22440	34449	11244	82152
MS4A10	19	4777891	4943291	3226003	2510890	5067485	123517	21018588
RBM14	23	10334	10565	7976	5859	9828	2023	54399
ADCY10	25	13689	19308	7700	5915	10139	2871	92414
GBP5	25	54121	27206	52501	37499	61274	5511	121221
GBP4	24	18455	52757	6717	3205	10769	1821	264201
FANCD2OS	25	33386	35368	22047	16059	30737	637	146408
PSMG3-AS1	25	46251	118174	11722	6820	25440	862	576656
LMTK3	25	17914	6895	16029	13619	21998	8809	37750

PG.Genes	n	mean	sd	median	q1	q3	min	max
TRNT1	25	66576	66081	41501	31496	68526	13784	304206
FLACC1	25	42219	25545	32274	27611	42227	19857	127212
TMEM237	23	9218	17512	3997	2965	7252	1421	86784
GSDMA	24	8477	6102	6611	5786	10320	2616	32935
PRAM1	16	1706	1964	1372	889	1591	479	8805
VPS35	24	8413	6583	6299	3121	11752	1427	28842
PURB	19	3753	4880	2121	1164	3752	539	20433
PSKH2	25	264900	188645	183911	162954	327910	9591	750450
PHF12	20	7278	13383	4246	2086	6698	1146	63073
RBP7	25	369885	253655	323933	217838	452730	7111	1114245
PANX2	24	10508	7331	9110	6254	12893	2448	38724
NACC1	25	141959	190934	92731	74519	116682	35379	1029798
ZNF300	25	40117	26552	30662	26614	50806	9562	143304
SNX18	25	8709	12697	5079	3337	5945	2345	57978
VPS13A	25	23626	18739	17893	15727	22515	10436	94766
MCCC1	25	6623	4843	4989	3904	6600	1855	22102
TP53RK	22	4826	3429	3606	2823	5200	1685	16075
HAPLN3	23	14365	8060	14218	6383	19903	3769	31352
PLEKHF1	25	29519	60448	14961	11417	23474	5439	316644
PPP1R9B	25	12783	17136	8365	5708	10054	3726	77081
SRPK1	24	11323	13111	8306	5200	13059	3330	69509
CPXM1	20	5178	6826	2549	1804	4953	176	24630
CYP2S1	22	13435	25897	5433	3406	7843	830	119018
IWS1	24	29761	29412	22348	14587	34395	7826	148550
SIN3A	25	719604	490982	586026	455444	824267	252913	2583266
ADO	25	274792	690826	86761	39405	122736	6904	3028606

PG.Genes	n	mean	sd	median	q1	q3	min	max
RUFY1	25	13565	9304	11540	10257	13293	4872	55307
NIBAN2	24	6804	3386	5539	4446	7669	3494	15546
TCF12	22	27836	75828	3051	1779	8377	555	287378
GAD1	24	23167	16426	18524	14574	28167	4540	71838
TBCB	24	9331	10847	7345	4027	10210	623	56809
PSMB7	22	3688	1828	3255	2854	4377	1327	8749
CNN2	23	10196	15404	5111	3117	10599	744	74051
PCYT2	25	51485	50834	35871	26412	55086	3238	262752
PHOX2B	19	872	355	931	642	1093	196	1436
CDC5L	25	39940	28587	32337	26252	42919	15121	160173
PSMD1	24	2275	1232	2110	1245	3320	399	4248
PFDN5	25	27684	25799	23186	16643	27062	10426	143854
PARK7	25	24344	15933	21144	11074	34939	3982	72031
SORT1	22	4567	3063	4111	2740	5456	1686	15812
VAT1	24	17917	21654	8865	3753	26755	1034	101568
NUP88	25	9199	12924	4403	3064	6132	1602	47867
PKP4	25	35781	40664	26894	18117	35435	10627	210674
POP1	25	119533	95516	76546	61702	148947	22990	446491
S100A13	23	5252	3744	4066	2776	6466	382	14250
SCAF11	24	165987	106641	145344	106623	179345	64100	604127
TSNAX	25	148546	279271	51834	42713	106850	18936	1368083
SEPHS2	23	9611	13483	2923	1564	11963	322	44898
TTC1	24	11638	9908	8369	5875	12752	4292	47732
DNAJC7	24	12340	21137	8581	5912	10305	360	110183
COPS8	20	1898	996	1754	1212	2133	699	4891
CHP1	24	68914	56178	57480	24576	90982	6975	224247

PG.Genes	n	mean	sd	median	q1	q3	min	max
MAP3K5	25	50515	94954	27219	18275	37813	5291	487978
MGLL	23	4363	3086	4556	1679	6656	623	11368
KIR2DL4	24	20932	18188	13990	7061	29667	5491	80020
HSD17B10	25	24489	78015	6901	4065	11127	1496	397394
CCL19	24	89238	78689	71899	37563	100140	6253	324354
NAP1L4	23	14081	7167	12629	9117	16181	6366	32016
NPAS1	23	3955	2435	3265	2515	4278	958	11048
NAPG	25	25458	31697	20236	11918	28309	3472	168738
TXN2	25	64756	195142	23017	12205	32510	3307	997640
ABCA3	25	27471	40417	10148	6401	20376	2106	179956
MIPEP	23	3356	3539	2110	1424	3519	596	16696
ACO2	25	32239	16669	29610	17871	44978	12418	80494
TM9SF2	24	3741	2317	3131	2285	5143	316	9113
TSG101	21	2793	1545	2399	1998	3405	614	7208
CPNE1	24	8050	7829	4223	2443	11590	1082	29222
CCT7	25	10180	9065	7532	4421	12917	1735	47064
EBNA1BP2	24	29596	31274	18801	10759	41250	3701	123707
PKP2	25	50196	26136	42684	35744	54879	13493	137008
SH3GL1	24	37854	109594	11329	8985	19598	4577	549211
TEP1	25	43884	30320	36041	24093	55542	4536	154493
OMD	18	4871	5319	3340	1612	5056	625	22441
SEMA3C	25	21425	43290	10785	4317	20822	2985	225058
VRK1	25	8782	7495	6582	4828	9692	1990	37728
GDF15	22	1900	1194	1786	903	2691	454	4317
DPYSL5	24	10215	11211	7185	5938	9504	2605	58922
NIPSNAP1	25	12520	6067	12558	8797	13652	3971	34881

PG.Genes	n	mean	sd	median	q1	q3	min	max
HSD17B14	17	13820	13259	8786	1301	26773	325	36342
ARPC5L	24	7229	6300	6139	4892	7491	1668	35012
FAM118B	24	11796	8396	10458	6207	14516	2173	39886
KCTD14	25	34451	17265	30830	21512	43821	7327	69737
TRIR	25	9728	13048	6405	5203	8279	3260	70585
MACROD1	22	1685325	3138117	959168	708621	1455145	195842	15580083
KLHDC3	24	4143	3731	3349	1925	4311	581	18658
WDR77	18	1553	836	1516	784	1984	527	3216
KXD1	23	136347	427125	17825	15190	27361	5475	1962978
ANTKMT	23	16563	7697	15828	11023	19249	3340	33152
TUBA1C	16	3708	4823	1988	1397	3169	696	20101
APOL2	15	1890	1581	1410	749	2426	508	5277
PSD2	17	2396	1541	1894	1572	2493	927	7258
HEPH	25	6619	7003	4680	3374	6920	1592	28879
ACBD6	23	90158	138096	17445	5197	134955	1408	519158
CORO1B	24	32499	121856	7382	4492	12049	1412	604238
CCDC77	25	43413	37924	29292	19891	52487	6250	152984
NAA38	24	6658	5370	4574	2811	8252	1523	19544
TXNDC17	24	9720	6689	7589	5035	12386	2258	27856
PLCD4	22	14313	19838	9454	7127	13074	4718	99221
CPPED1	23	7175	5722	4659	3524	7909	1684	21516
VPS25	25	22362	18893	18624	14494	22376	884	102676
NUDT16L1	17	8494	8833	4066	2790	11101	1368	35452
ERP44	22	4136	7439	2461	1819	3464	973	37151
LXN	25	18814	26299	12885	10493	16964	6352	142560
NTPCR	18	8402	7579	4850	2566	15319	324	25075

PG.Genes	n	mean	sd	median	q1	q3	min	max
TUBGCP2	24	8484	8254	6445	4521	9751	2167	44356
ESYT1	24	9955	8172	6796	3234	14773	1385	25725
UBAC1	22	18716	41357	10655	6509	13011	2490	203086
CNPY3	25	129323	84283	105338	74078	155749	9846	369771
PSMG3	15	2032	1083	1754	1248	2291	618	4127
COPS4	25	2497	1617	1992	1607	2749	806	7521
WAC	25	11630	11406	8208	4915	12018	2578	56376
DIDO1	25	256429	237359	198670	155105	233969	132202	1343987
RAMAC	25	397134	323308	287472	218604	484255	94643	1619750
FUCA2	25	29650	21452	24894	17421	31542	13566	118567
HGH1	25	39779	18363	36803	31514	45873	763	88918
DOHH	21	23870	42929	6414	2793	22171	670	178631
SPINDOC	25	11870	5067	10457	8492	14492	3928	25406
TUBB6	25	2923	2246	2504	1776	3197	953	12556
PAXX	19	3548	2123	3002	1790	5162	976	8109
HNRNPUL1	23	17115	8278	15202	11004	19056	8099	35748
PDCD10	24	8546	22865	3589	2675	4979	1573	115579
EFHD1	25	114167	81692	93530	63586	128454	4360	381905
DDX23	24	19015	13894	14528	10176	23400	1911	49845
BDH2	25	9610	5426	8332	6912	10835	3483	28379
ALG12	24	7107	5018	5561	3584	10222	1628	21574
MRI1	24	15109	17361	9460	5591	13127	2590	74441
CCDC32	17	5324	9724	3052	2731	3479	500	42661
THUMPD3	25	27449	55641	14225	10044	19558	6322	285207
ADI1	24	9964	7946	7629	6002	11938	1831	38814
RNF126	24	81470	96804	54083	18010	98002	9341	451294

PG.Genes	n	mean	sd	median	q1	q3	min	max
KATNB1	25	137552	108040	79969	50813	232667	23729	403350
TMEM109	15	2218	3121	1113	538	2216	3	11995
PBDC1	25	14706	25436	7876	6687	12612	2859	134468
DUSP23	21	6919	12121	2043	1600	4347	780	48495
TMED9	19	10126	6375	8270	6019	12775	2217	28171
SELENOO	25	51870	43695	33330	25947	60178	6831	153283
DPCD	22	10206	8688	7762	5892	9871	2015	42574
GNL3	24	24727	8226	24500	18617	28341	13339	50785
TPPP3	25	9291	6263	7999	3976	14448	1938	21883
HIRIP3	21	6418	12530	3591	1945	5672	131	59900
RBM4	17	3361	2664	2416	1170	4933	526	9421
PRR14	25	111004	224112	19201	7467	94940	2568	1036893
SCRT1	25	8384	9087	5556	4328	8066	2324	45904
APOL6	23	20650	29184	11706	5627	21823	2423	141940
SYCP2	25	33963	16664	29622	23094	36991	6280	82426
LSM14B	25	307618	247110	232649	183299	336734	5140	1040075
SORBS1	25	8091	3091	7139	5879	10144	2628	14220
ZNF471	23	2151	2931	1063	774	2097	474	12398
PLVAP	23	14975	14313	10767	9262	17891	1517	73995
SPATA16	22	4278	2683	3469	2474	5240	1216	12472
RAB11FIP5	15	3513	3015	3011	2185	3577	298	13499
C1QTNF5	24	28003	24400	20347	11114	39566	725	83099
TMEM120A	25	137571	103869	102078	73548	184583	27569	520380
NAA15	25	23322	23785	18187	12095	23419	9552	128719
CDCA4	23	4914	3856	3968	2485	4878	1014	16205
ASPN	23	14783	24093	4827	2294	11037	267	93847

PG.Genes	n	mean	sd	median	q1	q3	min	max
PAPPA2	25	71978	68918	56613	42984	83466	16555	377313
QTRT1	25	92678	96147	54398	32311	104803	8878	437982
CFHR5	17	6781	5787	4620	2491	8037	344	20189
CACNG6	18	11180	7402	10482	4170	16908	1683	27356
TEX12	25	158342	260839	97076	69036	145155	9110	1369348
OSBPL1A	25	24070	11742	21889	16872	27265	10092	65706
EMILIN2	23	34435	100000	11154	6931	18299	4229	490739
ANKRD30A	25	31694	33725	21245	13135	31178	2094	138001
PACSIN1	25	18411	30708	9938	7518	13577	4730	158578
GPR87	24	12050	9124	8789	5273	17536	1279	39711
ITPA	21	5599	4918	4234	2690	5505	1102	20055
EIF2A	25	5752	2146	5755	3996	7691	1818	9506
CADM1	25	71321	108966	48458	34925	61711	18544	585745
POLDIP3	20	43717	146616	2117	1756	3924	961	656080
GNB1L	22	9115	14568	3327	1963	7654	798	51915
PCGF6	25	143393	203090	94340	74322	156012	4505	1076188
YTHDF1	25	77070	153761	29385	16110	52817	9173	754620
STK33	25	10781	8636	8813	5688	12619	784	42687
NLN	24	9025	9371	7276	5965	8422	2017	50963
CEP41	22	2427	1766	2177	1506	2700	473	8710
POTEKP	24	52033	30551	44464	31604	75256	6328	121320
NUF2	24	39314	13905	38473	27195	50219	14014	68191
GTPBP4	25	46739	66320	29907	24579	37551	14420	351720
ASPSCR1	25	13749	5773	11908	10269	15059	8265	36571
DPH1	25	36397	23985	24123	21068	57422	7147	89530
IRX2	18	6913	5009	6446	3712	8809	1498	23470

PG.Genes	n	mean	sd	median	q1	q3	min	max
UPF3B	24	54674	252366	3003	2042	4242	1087	1239473
TBL1XR1	25	17175	20241	8405	5407	23077	1650	90319
UBL5	16	1782	1134	1563	949	2252	473	4902
GSX2	25	262262	372065	151731	108199	262492	10608	1798400
NIBAN1	25	21086	14158	16741	12943	22418	7903	72691
UBXN6	22	3625	7221	1402	494	3464	193	34361
TM6SF2	22	5419	2098	4784	4127	6945	1319	8971
DPY30	23	4672	3522	4479	2243	5761	500	16900
TRIM2	25	174305	195104	126128	100492	172042	71967	1082277
SPEF2	25	13154	6389	11633	8298	14735	6835	30734
FTO	25	29915	29894	20030	15451	25287	11153	144928
CFAP74	25	26304	31816	16916	12285	23972	8155	164691
TNKS1BP1	24	11857	5634	10292	8496	13591	4875	31421
UBE2O	25	12939	7693	11089	8309	14971	5102	43118
ZNF518B	25	82493	38191	77762	54006	88456	31946	162743
XPO4	25	26163	44252	14199	10262	18125	3632	185938
SRCIN1	25	46213	18786	41411	34316	60629	16046	80156
WDR12	24	5743	6160	3989	2883	5568	985	25397
C20orf27	21	7605	5384	5145	3443	10418	2112	22351
PDGFD	25	57016	27821	52325	43679	70817	4088	133308
PITHD1	24	6084	3203	5501	4634	6417	3489	19977
DNAI2	24	4367	2111	4126	3440	4520	1186	12454
WDR61	15	3860	1742	3850	2201	4911	1397	6674
NIF3L1	24	3240	3676	2459	1661	3695	156	19679
EGLN1	20	9883	15664	4617	1871	8641	950	59467
NYX	23	406063	1051616	125799	103000	198503	60798	5073140

PG.Genes	n	mean	sd	median	q1	q3	min	max
NAA50	24	10013	12296	6837	4340	10415	547	57825
UBA5	24	13677	11329	9348	4393	26567	1780	33327
LHPP	22	7743	6231	4592	2944	10409	1912	21276
PAIP1	22	24454	21316	17566	8935	30032	4497	76319
NAT10	25	27004	40741	14912	7813	25476	3544	201662
COMMD4	16	1922	775	1839	1436	2474	610	3212
IQCN	25	72551	51593	64798	43311	80097	28912	294615
KLC2	22	11396	19858	4887	2788	6423	957	82814
XRN2	25	12808	6065	10892	8515	15592	4660	24750
TOLLIP	24	296250	227409	216449	163900	270830	77641	970158
INTS2	25	32578	14398	28929	20083	39905	15379	67026
KLHL25	25	98012	82497	84284	56766	114136	17402	442502
TKTL2	24	4089	1875	3501	2774	4927	1927	8013
QRICH2	25	13709	6850	11619	9494	15405	5545	31083
PARP12	24	58313	83710	32143	16877	67957	9553	419623
CSTF2T	25	73900	41611	71777	37183	92801	19163	195966
PCBD2	24	8652	6960	6960	3178	13866	1771	26723
CYRIA	24	108629	169391	66100	27019	88021	5950	760940
HDHD2	20	6166	4065	4941	4025	6732	3444	22020
RAB1B	25	22638	23841	15474	13594	23170	4653	131804
MRPL18	24	5277	4041	4956	2421	6297	479	17053
C11orf54	23	7972	4619	7717	4481	10003	1706	18658
FAM234A	23	13876	16803	9885	4706	13107	142	77717
NAPB	25	84256	22827	80813	75054	95495	32732	144250
CDH19	22	3728	2301	3210	2570	4259	1060	10939
SIL1	17	1479	1541	1015	705	1770	290	6954

PG.Genes	n	mean	sd	median	q1	q3	min	max
IRF2BPL	25	157860	72938	160888	114386	173811	59542	381315
UNC93B1	25	34173	22734	30363	18593	43707	8993	116080
POLR3F	17	8740	8383	6083	3323	8590	1583	28962
NUCKS1	25	13767	12691	10187	5052	21105	866	57058
TMX4	25	33017	107126	9781	4391	16170	1938	544776
MEGF9	15	2166	5343	890	514	1093	205	21441
ATG5	24	2394	1109	2147	1547	3001	1119	5072
WDR13	23	14377	13884	7736	5316	17348	1084	50402
TSPAN10	23	11752	10928	9278	7176	12936	2200	58414
EPN3	24	7880	8728	4557	2650	9352	1597	41302
OR10A2	25	61101	43655	54999	33863	78444	3579	193968
EHD4	18	6150	3672	5228	3629	7962	1548	16401
GBA3	22	8172	5675	6844	3641	10925	1628	24192
MMP28	25	35922	93422	15231	11485	18650	5158	480598
SPTBN4	25	24329	20944	19005	13062	25782	7277	103259
OR51E2	25	179776	277619	97468	62601	185579	23253	1430820
SH3BGRL3	24	41643	34376	29792	16546	55983	10680	137773
HSD3B7	25	22414	30294	15641	9470	23819	3038	161236
TSPYL2	20	7920	18563	3145	1828	4750	789	85448
BLZF1	25	14856	11622	10539	9115	15522	6312	53939
PPIL3	20	1597	582	1652	1296	1823	608	2595
SLC38A1	20	2537	2397	1686	834	3651	213	9716
PDCL3	22	5984	11037	2614	1972	6433	744	54238
RAB3GAP2	25	30797	67402	19500	8848	23569	1077	350980
ADNP	25	23786	8084	21906	18224	27945	11557	41330
MRPL46	18	4702	3776	3044	1983	6811	1251	13843

PG.Genes	n	mean	sd	median	q1	q3	min	max
ZNF106	25	38913	22450	30085	25282	49527	7009	101273
PNN	25	123423	96409	103385	53319	188099	15756	393684
OR52D1	24	17806	16876	13832	9141	22518	1488	84435
GGNBP2	25	46749	52168	34204	19642	56982	10094	265562
CPVL	25	23681	37428	6420	3017	15876	147	141336
BOLA2	23	10303	8019	8251	4620	12918	1997	27610
ACBD3	20	4654	5378	2899	1230	4969	431	21754
PTPN23	25	5771	5949	4118	2747	5749	1789	30029
CHMP4B	18	2163	1666	1994	906	2594	524	7157
FN3K	22	4214	6314	3062	2152	3596	1338	32172
POFUT1	25	17648	23201	13375	8750	16693	5791	126416
RNPEP	25	15928	18013	11942	6174	18785	2636	93415
GOLPH3L	23	18073	25341	12713	9914	16463	5479	132250
GOLPH3	20	8828	8526	5472	2138	13223	670	30368
TUBB1	20	5371	3912	3913	3091	5840	1776	17705
SMOC1	22	5825	3950	5206	3036	6927	1012	18160
EPB41L1	23	33859	14949	33145	23836	38878	9603	63042
GLIPR2	20	1653	1245	1238	854	2058	318	4650
PLEKHA4	24	217565	179829	157943	107949	242527	33672	818068
EHD1	24	9094	11412	6821	4361	8783	2487	60562
PCIF1	25	38019	29646	28015	19562	47344	14139	132358
ESF1	25	46496	23843	42303	32958	53773	16425	109907
MROH8	25	898025	1344494	580786	399248	837265	150520	7092710
ELOVL6	25	73300	109705	41867	23467	60825	5345	536782
CIAO2A	15	3228	2100	2793	1447	4470	697	8439
SLITRK6	24	3287	2031	2411	2013	4446	953	8500

PG.Genes	n	mean	sd	median	q1	q3	min	max
STN1	25	17906	26306	10732	7443	13982	3909	135543
OPA3	24	45549	38278	37027	22420	63621	1003	166272
ACSS3	25	26217	15507	21858	15137	31092	10228	79477
YTHDC2	22	12886	17037	8273	5972	12814	1542	85746
CWH43	22	8160	10722	4980	4097	7168	1410	53063
SPRING1	18	605	1037	266	104	370	4	4328
DCTPP1	23	54835	35731	58169	28440	81502	1384	122590
SMYD3	23	2747	2371	1654	1166	4584	530	9898
AAMDC	25	30959	78090	10507	5467	23106	2982	400367
DOCK5	24	11737	13899	8610	6419	11375	1695	73107
ATP13A3	23	22834	23744	17353	12663	25052	7836	126684
DCLRE1B	21	2761	2082	2157	1623	3002	497	8953
UBE2Z	19	5161	3337	4685	2287	7383	726	12777
NOL11	23	19581	36944	8227	4116	15701	1538	177060
MMRN2	25	19044	14363	16892	10327	20275	4502	75065
AKTIP	25	41834	62845	27746	17338	42016	1928	330973
RNF121	19	22788	12427	21429	16401	25921	6669	56603
SLC25A22	17	1909	1969	982	732	2353	404	6387
MED20	22	1751	2793	928	788	1229	567	13721
QTRT2	24	9243	11662	5909	3874	8761	336	55904
ARMT1	24	5329	14981	2115	1135	3142	477	75303
PANK3	19	4535	6603	3321	1678	4113	304	30381
LRRC40	24	9832	6901	6787	5146	13021	1297	29405
AGO3	24	35189	62571	19288	11880	26939	5843	317853
CHODL	23	7843	24669	2496	1503	4470	18	120684
NHEJ1	24	87130	39337	82265	60124	111320	18936	188098

PG.Genes	n	mean	sd	median	q1	q3	min	max
CAB39L	17	11617	20247	5186	3767	8710	629	84177
FN3KRP	24	4285	2155	3828	2849	4647	1660	10699
CARS2	25	27266	13615	25146	18908	34396	3210	60401
PPCS	22	2058	945	1958	1484	2463	436	4154
NMNAT1	25	117108	82280	99844	62693	137807	13059	305575
MLXIP	25	11478	6190	9454	7276	14153	5373	34707
C17orf75	21	17852	60544	2755	2456	6353	1646	281571
SIAE	17	4577	2985	4846	1724	6068	364	10668
PLEKHA5	25	16312	37065	7731	7084	10080	5261	193575
UPF2	23	7962	16306	3599	1973	6062	326	79883
RNPEPL1	18	13573	28721	3206	1651	5979	5	100080
ELOVL3	21	16505	12421	10307	8023	24333	5254	53586
MYG1	23	4244	2105	3628	2720	5042	1829	9003
KCNK13	25	22029	23297	15568	7273	25253	2779	107921
SEBOX	25	36690	17900	31233	25393	42889	5534	70814
SCPEP1	22	16991	12594	14632	10368	19498	1751	64858
CYP3A43	23	66115	148450	20647	10708	30670	1226	706370
PARVB	19	4231	2531	3716	2893	5140	805	9251
TNS1	25	122414	55055	119381	88795	142800	47204	292628
NMRAL1	23	11271	6492	9941	7566	14732	423	28200
SLC38A10	25	6927	15153	4019	3263	4482	1399	79438
ZNF287	24	505469	403613	422774	129958	818403	10216	1252848
EML4	24	15004	14448	12005	10957	14171	4910	80837
GLOD4	25	20623	17266	15481	8589	27758	3939	85346
CBX8	22	9640	13567	3943	2456	10799	1286	59919
MUC5B	25	33677	34606	20346	13198	40723	5417	151343

PG.Genes	n	mean	sd	median	q1	q3	min	max
NEK6	24	20135	42163	9545	5621	18683	568	213394
SPON1	25	10368	25681	4320	2906	6287	1634	131370
MCCC2	20	2687	3757	1742	1180	2494	410	17857
NCOA5	24	9734	8521	6246	4506	10504	3198	35204
MOV10	25	100109	374542	17425	12993	23977	8445	1888726
ANKH	25	21670	13349	17135	13657	23409	10763	74064
VAT1L	24	15010	31989	5429	3937	11339	2108	156259
GPAM	21	12875	14187	8369	4602	17393	1232	58058
RESF1	25	31673	36546	20513	17233	27776	10943	180926
PLXNA4	25	26252	48621	14235	10918	20805	8697	256052
PGAP6	20	3188	5186	1471	1059	2673	512	23935
EPB41L4A	20	30841	118000	4128	2751	5497	424	531955
XAB2	25	19886	15526	16747	12122	20435	5814	84077
CD248	25	52419	62540	33185	19842	54229	6135	290347
PREB	21	21632	30436	12120	8174	17282	2797	122277
CHMP1A	17	1906	1329	1514	1241	2588	325	5261
APMAP	23	22697	31622	15706	9105	21270	697	159730
TXNRD2	25	23876	30872	16468	11509	24076	2388	159880
ARFGAP3	24	3590	2343	2912	2082	3879	1396	10858
PALMD	25	46391	50018	36999	15584	58117	3781	231821
VTA1	20	6111	18057	1759	1591	2388	840	82718
DYNLRB1	16	2968	3058	2298	1263	3702	658	13292
PARD6A	24	237122	159170	207088	162210	268724	24405	741126
GPCPD1	25	66815	30974	55920	51262	76163	15753	152950
CCNB1IP1	23	22617	42121	12163	8589	17498	3820	211436
A4GALT	25	16557	17354	10767	6565	20299	2324	83993

PG.Genes	n	mean	sd	median	q1	q3	min	max
MYNN	21	6804	13238	3734	2284	5366	834	63579
OSGEP	22	5302	2597	4381	3401	7903	1301	10237
ACP6	24	10656	7603	9956	5405	12716	67	29548
ISYNA1	25	10852	4645	9548	7623	11482	3962	21817
OBP2B	25	47423	61790	25267	17100	50822	8413	305489
NXT2	18	3138	3156	2092	1309	4028	323	13543
CD93	20	7399	6492	3672	3046	10446	2476	23896
LZTFL1	24	13073	8246	11427	6739	16203	2526	37304
MEPE	24	37149	65041	15702	6266	37068	2598	252598
TIGAR	25	95448	43676	91805	62489	112948	28397	203240
RTN4	25	12232	14504	7208	6366	12779	460	70896
CYLD	22	4336	4011	2820	1854	5083	683	14454
HINT3	21	12769	41963	3086	2176	4925	907	195646
RPRD1B	23	4864	7070	3790	2256	4230	807	36378
RRAGD	21	5221	4566	3335	2379	6549	1147	16741
NIT2	24	7192	5263	5437	2744	11578	1260	17630
AVEN	24	12088	9967	10135	5907	13685	1351	48113
KIF13B	25	43872	24525	41161	30174	43535	25094	146551
PAK6	25	35179	51307	22103	15613	34697	7524	272884
ANLN	25	7893	7207	4828	4302	7215	1358	30342
XPNPEP1	25	87376	41949	75568	66182	101095	45149	254142
GPHN	23	1985	2294	1450	763	2044	505	11606
MYO5C	25	20935	27815	13409	12634	19820	8544	152512
ITM2C	20	4830	5093	3358	2594	5476	1026	25046
BIN3	25	28848	37837	18014	14302	27291	9240	202521
DDX21	25	77455	100534	51006	34083	64173	18473	470282

PG.Genes	n	mean	sd	median	q1	q3	min	max
MAN1C1	24	41589	42431	27778	22005	41478	16289	214887
NANS	25	3012420	1576759	2814446	2008579	3681133	857463	6970946
SH3GLB2	20	2418	1327	2266	1437	3215	418	5943
EIF2B3	18	3689	1762	3281	2336	4288	1931	7904
FBXO6	25	21918	11309	18969	16933	25624	2649	62028
PICK1	25	165025	141454	130391	105324	170065	68608	783083
CTPS2	21	11480	32612	4718	2335	5916	1190	153442
PAPOLB	19	6965	3325	6125	4105	9659	3031	15293
STRN4	25	43750	23818	36413	24820	53992	18289	99944
ENAM	23	13131	21352	7318	3112	11901	1129	102946
AASDHPPT	24	10090	10600	5457	4171	9953	2954	42321
UBQLN4	24	10331	25331	5122	3434	6476	1258	128649
HEBP1	25	29109	107464	6950	5450	10294	3262	544776
RAB6B	25	62800	46580	48840	34406	70346	26934	205503
PHPT1	24	3877	3183	2757	2286	3678	1605	15723
ARHGAP35	25	29921	18413	25370	15169	43051	5346	82285
FAM114A2	25	31405	20632	27274	20549	35424	7974	109687
LTBP3	23	9876	4324	9425	7580	12884	2054	21396
RGS18	25	21722	8140	21161	15201	26290	11588	41017
LANCL2	18	1866	927	1472	1235	2493	736	3738
KCND1	15	4322	3248	3011	2103	5629	271	10894
ST6GALNAC1	25	22988	26348	16824	12436	21691	7111	142346
FARSB	25	534035	2605218	12456	7710	16137	2613	13039022
IARS2	25	1043140	1075658	782158	721384	968284	501257	6059820
STARD5	23	13086	6953	10918	8097	16429	5144	28906
ATG3	18	1932	2375	954	644	2092	463	9959

PG.Genes	n	mean	sd	median	q1	q3	min	max
PDS5B	25	148919	94718	135355	111085	175837	27604	520232
OLA1	25	7051	3555	6132	4623	9404	1160	13966
RBM12	25	30031	47102	12943	11480	22958	8402	188051
ANKEF1	25	14321	7877	12455	10164	16015	3954	37582
ZCCHC3	25	25508	22173	20261	14769	28241	2499	118690
ABHD10	15	3524	6664	846	539	1953	4	21083
STAU2	25	34275	28103	29762	20972	34801	12375	160315
LIN7C	25	19474	11856	19279	11419	25814	2428	59924
CYRIB	22	7868	7066	4563	3174	10550	1726	26707
DDX19A	25	46371	13181	45794	36624	49134	22135	83619
GIMAP4	24	5436	2855	4191	3478	7467	1096	13057
TDP1	23	2978	1765	2804	1619	3207	581	7389
MRGBP	25	8175	9076	5369	3800	10365	289	45563
SEPTIN11	25	18348	57643	6540	3025	9770	1000	293748
SLC38A7	25	15183	7281	13860	10266	17628	5740	36819
MED17	24	20822	18048	17869	7614	27149	1828	75472
PARVA	24	52646	86235	33618	18129	53233	7471	446557
PANK4	25	31577	33048	27047	16589	31252	7688	183920
FBXO28	20	12982	20756	5944	2558	11149	1048	87677
ARL8B	20	48311	48159	30654	13381	96214	637	150501
DDX18	20	6240	16073	2060	1557	3208	878	74116
TBCCD1	21	6375	4549	4597	3785	8629	858	18639
MTPAP	25	260505	551594	144881	109712	192822	44346	2882300
RLIM	25	12158	7202	11425	6938	18289	1959	28956
RBM22	18	862	427	896	671	1018	1	1644
WDR70	25	26703	9126	28052	19550	32909	11800	41379

PG.Genes	n	mean	sd	median	q1	q3	min	max
SLTM	25	12864	11872	10254	8231	13024	5411	67845
PAG1	25	557111	640093	382985	143611	723119	57461	3200155
PARPBP	24	8943	19307	3710	3112	6001	924	97789
HIF1AN	23	1952	1394	1439	820	3058	516	5250
CZIB	23	11848	9980	7529	4426	18507	2105	44152
BABAM1	25	7193	11429	4161	2526	6731	1005	59416
CLN6	18	10685	16139	2648	1492	13778	292	65602
C2orf42	24	31068	24626	24477	11725	40064	6172	99458
THG1L	19	2191	1241	1720	1075	3372	734	4433
IRAK4	23	7636	6079	6060	4787	7522	1654	26124
COMMD8	15	1421	984	1427	612	1991	104	3419
OCIAD1	25	13656	25863	4298	2549	11533	813	104980
ADPRS	23	4863	6087	2998	2139	5690	593	30887
HYPK	19	9264	8614	4981	2713	16265	170	24252
CHCHD3	22	117866	186659	67124	48161	110176	18659	920196
ZSCAN32	25	69844	54470	53996	31305	88215	2210	204848
ERVK13-1	23	6237	12732	2091	1357	5376	779	62543
SIRT5	15	928	756	717	379	1285	89	2992
THUMPD1	25	7786	17117	4414	3321	5265	1852	89643
FBXL12	25	68085	100266	28268	18931	42350	2243	384830
GIN1	24	18214	16336	12769	10419	18980	5650	76893
NDE1	25	12320	8273	10065	7032	12241	4816	40690
BABAM2	21	11262	22915	3152	1358	7739	252	86698
CDKN2AIP	25	386195	432352	235715	154761	310476	38224	1972846
GAR1	23	310171	421227	201187	152949	257260	1422	2065055
STAB1	25	26736	15733	23871	17686	27570	16015	95460

PG.Genes	n	mean	sd	median	q1	q3	min	max
PPP4R2	23	15301	13879	9657	7983	14207	2770	55511
SLC5A4	24	6087	11118	2536	1910	4397	750	43533
TERF2IP	24	10863	8420	8349	5421	14733	2126	38606
BCLAF1	25	9405	5923	7518	6561	9557	5534	32406
TLR7	25	77538	205250	24336	12680	35224	3341	1006714
MAP3K20	22	13907	9143	11974	8318	16217	3032	41125
TMOD3	21	10041	12245	6343	4900	9618	2771	60829
CELSR1	25	11318	5340	10899	8072	13183	2310	24138
FAT2	25	79825	93584	61204	46440	80714	31514	519497
NKIRAS1	20	2804	2118	2499	1534	3233	214	9439
UGGT2	25	169888	600466	40565	33820	56715	25936	3048933
UGGT1	24	8960	3805	8058	6683	11657	4390	22204
RBAK	24	22390	21109	18444	11693	24447	8341	115486
ERAP1	25	42433	41987	33216	23896	49432	6870	223910
ACTR10	24	5266	5215	3972	3246	4726	1971	27818
C9orf78	18	7320	12838	3541	1340	8214	761	57086
FAM120A	25	7611	6625	5403	4185	7984	2475	35606
SMARCAL1	21	6149	7312	3564	1982	6194	667	27811
GLTP	24	3954	3037	2805	2034	4370	1135	12488
AHSP	24	12037	34229	4940	1764	7668	872	171699
EHD3	24	13561	22458	7136	4640	11864	1819	112287
EHD2	21	24500	30305	10792	2330	40320	1182	115104
CNOT2	19	14651	12354	10423	5873	21881	251	40635
C1RL	25	16044	15350	11624	7752	19047	2920	71688
TMOD2	20	15812	7495	13743	11574	17015	6252	33964
CALML5	25	10536	8869	8341	5692	9620	2625	42349

PG.Genes	n	mean	sd	median	q1	q3	min	max
OGFR	22	4258	5618	2357	1665	3489	1083	24742
LMCD1	25	3873	2270	3308	2260	5512	871	10897
THYN1	24	8654	18087	3860	2830	6962	1499	92369
NDUFAF4	18	6021	8610	3136	2036	4907	1039	35603
HACD3	16	4998	7077	2661	2274	3531	752	29689
VAPA	23	48729	35486	39073	23728	62536	1711	159213
MACROH2A2	18	4575	8595	1907	893	3981	490	37405
MTRES1	21	1925	1790	1395	948	1992	334	8237
ZNF581	18	7038	7843	3489	1298	12808	276	25488
SPATA7	24	6990	3309	6390	4867	8990	1827	16899
ABRACL	22	7223	4784	5932	3151	9957	1802	17592
MDFIC	25	15772	5916	15155	10200	19009	7479	27885
ACTR3B	23	5413	3826	4798	2771	6357	802	14157
HCN3	25	9376	9748	7043	4801	9180	2655	50125
WHRN	24	7029	6489	5975	3068	8205	120	30982
PLCE1	25	18253	18068	11690	8937	16591	3210	83918
VPS18	25	105260	127272	54067	37755	93764	5508	477519
RCC2	21	6775	4211	7523	3006	9264	1442	14913
SLAIN2	25	12774	22621	7456	5745	10231	4513	119708
USP36	24	25206	46068	15015	10549	19336	6618	239149
FNIP2	25	23059	12702	19751	14982	35271	3756	53071
BAHCC1	25	207922	83356	194814	153151	237386	86886	463291
STK26	25	181635	57070	170384	151490	202520	86028	373480
PTGFRN	25	37365	13336	36666	27708	45192	12236	64663
RRBP1	25	11344	9624	9353	6380	10953	4726	52475
MAP10	25	54461	40495	39756	34463	71904	4958	195562

PG.Genes	n	mean	sd	median	q1	q3	min	max
KLHL8	20	4190	2912	2931	2035	5786	826	12991
CEP126	25	30004	26207	22440	14767	36445	3125	129553
LARS1	25	27895	14347	25106	20490	31739	6480	72819
CC2D2A	25	45869	35630	37033	31430	43269	24293	185429
RBM27	24	14764	5635	13400	10684	17556	4538	29410
RERE	24	8534	6395	6579	4296	10250	3073	29695
SUCLA2	25	962897	552818	775788	643198	1048629	385251	2522556
WRAP73	25	14977	16200	11637	8625	14830	3514	88941
STX18	24	49042	83905	7588	3971	55438	1058	351305
DELEC1	15	14205	6834	11729	8866	19174	5253	26128
ATXN10	24	4511	6849	2960	2383	4179	1450	36253
MYO1A	25	45018	64113	27752	14457	45330	4941	332404
SPRR3	17	184103	300298	9741	4200	238635	725	830514
HSFX1	23	7144	4495	5258	4177	10187	1413	20307
SAE1	23	8041	5848	6788	3296	12647	2215	21024
NLK	24	24529	20932	19306	10516	27405	1727	78581
COPG2	24	4198	2560	3446	2660	5062	1040	10375
MRC2	24	83510	111341	51900	34567	89776	6895	460406
IL36RN	20	19420	18962	13626	5948	23906	413	59404
COMMD3	19	1133	696	959	694	1439	180	3085
GNG12	16	4199	6253	2222	1184	3677	408	24867
HCST	25	14547343	12722787	14436772	6650484	20295816	311607	60356292
CPNE7	25	21424	65373	5621	4736	12183	2205	334048
DKK3	15	1425	965	1333	769	1548	393	3847
VPS29	21	3843	2967	3915	1976	4976	532	12300
GRHPR	23	4484	3165	4011	2111	4869	646	12665

PG.Genes	n	mean	sd	median	q1	q3	min	max
CTSZ	23	5670	4469	4706	2157	7801	843	16757
UBA2	25	51851	30868	42652	29096	63100	14506	152026
NXF1	25	85716	49882	83146	50137	110445	24009	225662
BIN2	23	5655	4216	4777	3367	6146	2581	23171
COPS7A	25	16810	16227	11732	8401	19590	5028	82515
FBLN5	25	14704	14118	9390	4996	18349	752	59446
APEX2	21	2871	4071	1891	1391	2220	544	19602
PPP1R1B	24	84366	66929	67704	48462	110324	7552	341979
AASS	25	18641	21922	11282	9869	17327	6866	116277
ZNF212	25	16797	20383	10809	8502	16872	6099	109328
CFDP1	21	4247	3423	2667	2160	5185	644	12747
VTI1B	25	29447	39638	22075	17172	24568	4573	214557
STK39	23	5945	5643	4992	3186	6132	1882	29851
LRWD1	22	16006	54157	4144	2968	5118	663	258150
NIPSNAP3A	22	2593	1141	2753	1678	3413	8	4445
CGGBP1	25	18504	6760	17442	14785	22648	6591	34949
TES	22	5208	5269	3473	1202	8224	389	18572
DMBT1	23	13383	41091	5209	2669	6340	1428	201516
FETUB	25	49340	68970	32722	22120	44864	11563	360150
LIMD1	25	15002	7282	12592	10651	19244	6507	35540
HMGXB4	21	3077	2719	2292	1682	3104	867	13735
APOBEC3B	22	9002	9095	6581	4050	9549	2278	44214
SWAP70	25	93083	55955	74432	64067	93007	47544	299402
FBXO40	24	15867	39237	5721	3885	11508	411	197628
LIMA1	25	19067	17314	14206	11485	19544	8666	96257
SRP68	21	7641	17016	2632	1749	5775	837	80208

PG.Genes	n	mean	sd	median	q1	q3	min	max
CNTNAP2	24	177779	210798	90322	49544	256172	645	968630
NPC1L1	25	57558	46715	48480	42263	57441	21807	271012
CHORDC1	23	7389	9658	5516	4126	6833	1099	50912
UBQLN2	22	11830	8128	9776	6078	14427	3545	39780
EGFL7	24	5696	4173	4560	3047	7560	1054	17726
IL20RA	25	41660	33261	32427	23989	47275	3178	163282
ADAMTS1	25	31854	20117	27977	17381	46131	4915	86612
DPP7	22	4701	2787	4073	2634	5295	2018	11946
SAP30BP	16	1626	1597	1148	928	1596	295	7109
ZNHIT2	25	1466089	767973	1233749	1139955	1692244	682656	4322952
PFDN2	25	177264	311596	110613	79929	144329	61657	1655479
PUF60	24	5366	4933	3818	2910	5635	1564	24572
NRBP1	23	7671	4198	7880	5693	9044	166	22552
ENOPH1	24	9667	5950	8260	5591	11920	3914	29329
ATP6V1H	24	3736	1692	3450	2646	4506	1215	8250
TAGLN3	25	20557	40318	8910	6076	16488	3075	204928
CPA4	25	7560	5651	6524	3366	8739	1644	28107
XPO7	24	2966	1258	2656	2193	3300	1491	6646
BAZ1B	25	44170	37824	33147	29636	46549	11176	197426
ATP5IF1	21	5903	4064	4232	2231	8723	1345	14878
AK3	25	4834	1614	4981	3642	5857	2443	8044
CNOT7	25	3431	3505	2174	1506	4248	1131	18750
SERPINB13	25	32636	62921	14760	11145	28919	5070	326753
GGT7	23	18300	13740	14569	11107	22320	841	65539
RABGEF1	25	4528	3773	3300	2927	4880	1372	20797
MAGEL2	25	16648	36084	4583	4158	8428	2912	170389

PG.Genes	n	mean	sd	median	q1	q3	min	max
NAGK	24	13105	14529	10824	6338	13852	2954	77121
CRLS1	23	5997	7686	2921	1591	6197	1002	33325
SH3BGRL2	20	416467	379650	285677	190085	547482	37679	1722366
RASAL2	24	35202	48446	26023	18959	30449	5941	256038
DCTN4	22	3925	5010	1851	1336	4337	647	18620
ZNF229	25	16007	12191	12293	9409	17680	5008	65265
CDC23	25	32038	18280	26763	21683	38332	6926	83727
ANAPC2	25	35523	33925	23757	14397	36260	5585	126447
VPS28	23	128074	133444	88738	47507	107974	4351	465928
LSM7	20	9036	8595	5992	2072	13875	861	35660
SERPINA10	24	5320	6377	4139	2225	5723	560	32717
TASOR	25	119582	122627	77740	70367	100408	57414	623014
JPT1	25	27675	21060	18996	13029	33403	1512	80580
FBXO3	24	1018074	3440456	224940	159646	273718	102471	17091978
AKAP11	25	60445	34397	46467	36657	80082	14385	142565
DNAJC12	24	19725	11438	21731	11134	25981	1611	51474
APPL1	20	2920	1315	3078	1893	3664	657	5217
PARP4	25	693305	488580	508146	418652	662180	253949	2422403
NUDT5	22	5360	5010	3792	2190	5808	1139	22941
RCOR1	21	8275	4101	7322	5676	11204	1454	15652
MAN1B1	23	14196	18473	8536	5803	12149	2300	82354
MYO15A	25	14135	8471	12064	9388	13848	6466	45964
GTF3C4	24	33529	26355	28152	23703	33615	14448	149640
ADAMTS6	24	6782	4023	5199	4321	7764	2950	16886
PACSIN3	19	2920	2154	2907	1482	3385	690	8743
FBXO4	22	5415	2978	5354	2736	6449	2031	13529

PG.Genes	n	mean	sd	median	q1	q3	min	max
FBXL21P	24	24228	36619	16274	10967	23425	5242	191599
ACIN1	25	277957	117883	255035	214866	334627	78051	599826
AGO2	25	183724	226246	118502	111569	156490	58180	1217414
MYH2	25	55229	42642	36526	31111	53013	23088	217117
MYH13	25	9404	6692	7920	6364	10215	4014	38209
ZHX1	25	34943	57428	20708	10993	24727	5269	254496
DSE	25	255288	168722	213345	134162	336375	21910	730989
AGO1	25	9733	13759	6701	4828	8889	3395	74606
RAB21	18	5790	3272	4516	3331	7975	1950	14121
RAB22A	24	240969	1032459	28399	18065	35968	936	5087275
PSME2	23	10887	7893	7724	6207	10983	3463	34129
RAB23	23	4377	3485	2992	1468	6961	450	12071
MCTS1	24	13350	29439	5628	3041	12020	1203	149327
MTUS1	25	26397	68110	11534	8635	13774	6127	352094
PALD1	25	51187	76194	31606	18660	40798	7637	350257
SLC39A10	25	107864	95203	97056	52216	137054	6411	480432
ZBTB21	25	8925	7330	6700	5488	8009	3427	37364
KIAA1210	25	10891	5673	9675	7492	11662	5888	33130
PLEKHG1	25	38248	17413	36442	29710	44676	9586	99629
CNOT6	25	205580	178271	177958	64941	228665	28278	694512
TBC1D24	17	5425	10170	1408	873	3724	482	40411
PPM1H	20	4349	2630	4333	2077	5766	1279	11142
KCNS2	23	5978	5999	3166	2359	6939	1606	20365
ZNRF3	21	2597	3281	1613	939	2314	336	14759
HECTD1	25	33701	19484	27691	21614	35973	11122	90058
MYO5B	25	45294	37297	34136	28349	38175	20006	191731

PG.Genes	n	mean	sd	median	q1	q3	min	max
FZD4	25	33030	81323	14942	10941	21821	3478	420526
CORO1C	25	980768	792023	786810	356769	1570720	143834	2594009
NAP1L2	25	6265	4814	5064	2934	7255	1051	19666
PYCARD	24	4766	5989	3512	2230	4433	1089	31617
EPDR1	21	5604	3872	4722	3067	6307	974	14146
MYO6	25	69946	83376	55830	44788	59682	16158	454070
PPT2	22	31861	55541	18234	12056	31002	298	272309
NFU1	24	20557	39648	10945	9145	14049	4762	203921
PRPF19	25	778199	607817	748035	443872	836582	51150	2875515
SYNPO2	20	8750	10546	6231	4094	8069	1648	50022
VPS4A	24	57124	88746	31753	27118	41588	16524	448825
ARHGAP26	25	18231	16556	11694	7042	20065	3584	60822
STUB1	25	20767	14247	18478	10008	26608	4755	52842
CDC14A	25	45525	59224	26147	22048	39429	3268	298334
SNX6	25	79147	285280	17946	13742	37031	5257	1447098
DUSP12	16	4837	6666	2671	705	4540	376	26922
PSMD13	25	3251	2088	2277	1977	3644	1256	9790
FAF1	25	55762	59530	43509	14015	68346	3286	246634
DIMT1	21	5600	5675	4207	3201	5475	1647	29458
TIMELESS	23	37517	39949	19781	9900	54236	1867	152464
WDR3	25	29094	30055	18729	15868	24500	10797	131612
NSFL1C	25	6608	6618	5070	3732	6153	1991	33947
ADAMTS8	23	20359	50665	4054	2160	9191	452	242492
COG5	25	6481	5847	6025	3533	7621	1919	32330
SCAF8	25	21333	21091	16134	12436	23769	7061	118215
TRIM35	25	1046780	429348	1073853	738111	1241294	191623	1958996

PG.Genes	n	mean	sd	median	q1	q3	min	max
ZC3H4	25	20373	46609	9413	5851	15657	2432	241598
SORCS3	25	4522	5457	2905	2493	3871	1313	28955
USP24	25	85389	98594	63063	54830	72644	30489	542145
SAMD4A	25	10527	4125	9007	7981	14037	5606	22743
TRAK1	24	3508	2326	2795	1886	4680	576	8596
SHANK2	21	272089	448429	130270	112493	199625	3748	1900915
SRRM2	25	48630	51083	29682	25584	46267	17595	272855
CNTN6	24	23052	40006	14074	10529	20807	2702	206820
PA2G4	24	9567	5014	8060	5864	12796	3697	22612
SPG7	25	8946	17833	4760	4164	6320	1192	93606
CLCA2	22	27568	28603	14830	7165	38501	1145	86207
ERVW-1	25	172048	72248	159200	134114	203304	71429	374965
MAPK8IP1	25	38182	25070	36451	17053	47647	5468	97762
ZNF148	21	8235	7450	5641	3123	10908	1121	30763
RTRAF	21	2850	1512	2390	1729	3815	967	6007
RUVBL2	21	2276	1443	1814	1495	2311	629	6115
LIPT1	24	72764	77003	56286	21017	85723	11948	360192
AKT3	25	62208	42081	50038	40883	63759	19224	239135
EIF3L	25	5448	2276	5335	3899	6621	2117	11439
PLAA	25	8421	15760	5265	4667	6282	2026	83716
RUVBL1	24	16171	15353	11461	9193	16138	5779	81542
NUDC	25	50584	28395	41376	31669	67868	2754	114461
ST3GAL6	25	21307	40603	12569	9508	16822	6936	214564
HS3ST2	24	44520	35098	27760	17374	66955	7821	127147
CFL2	25	243347	248768	93351	55289	501131	1653	701862
NCKAP1	25	20334	51441	9165	8221	11045	5396	266631

PG.Genes	n	mean	sd	median	q1	q3	min	max
CNPY2	21	6523	4135	6571	3469	9316	508	16284
DTX4	21	8670	6767	6533	3693	8626	2439	25988
STK38L	19	1282	675	1271	665	1972	174	2443
INPP5F	25	13602	9081	11931	9214	13720	5816	48513
PLEKHA6	24	3530	2106	2669	2080	4635	1313	9999
WDR37	25	49003	102149	21509	11876	34564	6416	508230
ZKSCAN5	25	111783	155847	70431	56867	81734	36668	717717
SLC27A6	25	63632	88181	39651	30066	51486	15697	459017
MRPS7	25	5123	4469	3978	2950	4926	2307	23469
TMA7	21	11899	11758	9486	3589	14806	568	48007
AP3M1	22	8300	16081	3241	2182	5695	791	72135
CARHSP1	24	11353	6874	9082	7372	13618	1692	30546
THRAP3	25	137313	99884	116714	89219	159158	21714	548681
NOP58	25	12877	14910	8352	6562	14524	3031	79469
DERA	24	21450	18632	15710	11873	23320	5422	95710
LSM2	19	6386	5107	4397	2541	8997	1349	18836
STARD10	24	5430	3882	4638	3126	5924	1840	18558
CAB39	19	3442	2490	2929	1388	4458	650	9423
MRPS2	25	20145	61163	6473	5283	10399	2794	312905
SBDS	22	27935	19011	23374	10139	45329	5201	62583
SF3B6	16	3548	2317	2814	2097	4554	987	9973
REXO2	23	5712	5371	3009	1909	9136	857	19398
RRP15	25	21029	22628	13643	9188	18956	3526	102681
PPIL1	24	2938	3029	2297	1726	2643	1219	16501
UFC1	22	4623	4724	3782	3003	4856	1134	24990
FIS1	17	4668	5318	3276	2443	4578	335	22625

PG.Genes	n	mean	sd	median	q1	q3	min	max
AK6	19	14040	11259	10858	7077	20063	1775	39452
HDGFL3	20	5972	5299	4325	2870	6922	418	18327
BOLA1	20	11568	7772	10063	4771	18592	2728	27140
CHMP3	17	18172	32683	3566	2643	14746	903	123724
CHMP3	22	10884	7222	9711	5088	14488	1566	27661
STRAP	24	6316	2530	6294	4484	8068	1253	11599
RTCB	23	18586	10448	16194	10479	25095	3546	42644
RABGAP1	25	12022	10101	8782	7591	12997	3505	55300
TSC22D4	22	5740	3408	5085	3397	6282	1921	14706
SAMHD1	25	16224	43586	6554	5707	10240	3479	224966
HBS1L	25	30134	25262	25796	18021	29449	14193	140571
SALL2	24	51522	42823	37085	21723	69442	12966	198558
PRKAB1	20	3080	2680	2085	1189	4998	450	11278
TLN1	25	26012	24007	19724	5583	38642	2959	100264
MFHAS1	24	6664	2690	5889	4838	8425	2879	11990
ZNF451	22	9732	8286	5618	4702	12702	1018	31555
USP15	25	29155	18057	26383	21173	28907	16030	105961
TOGARAM1	25	69001	40367	62654	51030	74803	32639	235847
RIPOR2	25	23414	14653	18432	16430	24818	11140	84300
TLN2	25	44802	16286	40352	32641	50801	23952	92568
IRS2	25	18670	17798	12187	8472	21135	4693	85535
CRYBG1	25	11102	9780	8789	6792	9865	4363	47982
MAP4K5	25	883427	864388	727525	539111	985668	188028	4733414
HYOU1	25	9448	6120	7379	7005	9221	4609	34603
TBL2	25	95905	108103	67103	50753	86724	31573	568137
TELO2	19	4618	4904	2549	2263	4108	1348	21903

PG.Genes	n	mean	sd	median	q1	q3	min	max
ARIH1	24	7545	7165	5178	4034	6401	2751	34677
LSM4	24	8962	13116	5365	4405	7748	2138	68449
OARD1	24	23559	27276	12984	9766	21989	3833	105233
HSPB11	15	2188	1830	1622	1061	2673	320	6935
SUPT16H	25	69593	45973	61261	47901	77056	25432	271254
PCDHB14	18	3194	2192	2739	1294	4115	852	8215
TIMM8B	24	48746	52576	34336	21023	46784	5024	221232
PCYT1B	24	7999	5459	6281	4342	10404	2395	23993
CD2AP	25	40547	30311	29459	23676	46393	11630	143787
TIMM13	24	4843	6704	3002	2526	4448	1366	35471
PPP2R3B	25	561771	581577	350524	73565	788721	17129	2077325
MMP24	25	11835	9030	9812	7423	12289	4807	49064
DMRT2	25	51043	27346	39886	34254	67713	15779	126199
TRAPPC1	19	14433	6411	13895	9472	18136	4676	25972
CDC42BPB	25	2124427	2961575	1293919	1101938	1578819	602593	13310675
RBM8A	23	6973	7831	4404	2597	8826	1135	34860
WIF1	19	10767	14767	5737	3141	8742	1735	63559
SNX13	24	25255	25125	14719	12035	24325	6207	101040
SNX9	23	3344	2200	2437	1878	4631	696	9645
SNX5	23	3675	2249	2778	2100	5007	1103	8913
LYVE1	25	37253	22909	31110	20834	51981	6915	112319
HEBP2	24	5986	3798	5167	3622	7156	1546	17737
HEBP2	23	10561	8153	7348	4290	15365	1839	32787
LRRFIP2	24	4153	3633	3343	2819	4050	1454	20276
FHOD1	25	37304	33975	32782	20214	42796	11973	187480
PSAT1	23	3148	1901	2530	2072	3647	1543	10218

PG.Genes	n	mean	sd	median	q1	q3	min	max
CPQ	25	37044	21101	35015	23458	47059	8970	88364
SPIN1	20	9186	11406	4232	2971	6719	1181	41098
COPG1	25	15907	29221	7496	3302	9734	2304	128666
CLIC4	24	41554	39358	22434	8826	83421	4860	136715
CFAP20	18	6659	4395	5442	3535	7927	1920	16605
EMILIN1	23	6550	5999	4354	2448	8366	148	22888
ARFGEF2	25	39563	16507	40371	26449	47813	10705	78572
ARFGEF1	25	13585	17521	7866	6750	12882	3931	91347
STK24	25	8842	14449	4915	2826	6871	1486	73461
BZW2	24	6539	10630	4114	3324	5907	1620	55846
COMMD10	25	107872	77586	93345	47949	131240	7372	319312
DYNC1LI1	25	6932	3105	6582	5014	7848	2708	14845
TEX264	25	1106170	1248685	833496	113405	1361594	18434	5029439
DNMT3A	25	11191	6156	10142	7572	13851	3375	31190
OAS3	24	6108	6931	3634	2787	5318	1099	30644
AK5	25	271118	330761	171915	125539	210156	108384	1702316
SQOR	25	14862	14060	10337	8176	15999	5398	75931
LAMC3	24	3513	2736	2776	1867	4053	1019	13632
CAPN6	24	15301	34549	5355	3023	8614	1942	166528
FCGBP	25	18446	30008	8027	6973	13767	4306	127161
CAPN7	24	17421	10816	14894	10901	17429	7594	45008
WASF2	15	5456	10410	2970	1668	3389	878	42574
FAM169A	23	2498	1950	1986	1135	2979	386	8515
SEC23IP	23	9256	9048	5827	3462	11881	1869	43514
